# Diversified Fluoroalkylation of Alkenes Using Quaternary Fluoroalkyl Alcohols as the Fluoroalkylating Reagents

**DOI:** 10.1002/advs.202408781

**Published:** 2024-12-24

**Authors:** Heng Lu, Zhoulong Fan, Yike Zou, Ao Zhang

**Affiliations:** ^1^ Shanghai Frontiers Science Center of Drug Target Identification and Delivery School of Pharmaceutical Sciences Shanghai Jiao Tong University Shanghai 200240 China; ^2^ National Key Laboratory of Innovative Immunotherapy Shanghai Jiao Tong University Shanghai 200240 China; ^3^ Frontiers Science Center for Transformative Molecules Shanghai Jiao Tong University Shanghai 200240 China

**Keywords:** drug modification, fluoroalkyation, heterocycle synthesis, C─C bond cleavage, photoredox catalysis

## Abstract

Given the widespread presence of fluoroalkyl functionalities in bioactive molecules, the development of fluoroalkylation reactions with bench‐stable and easy‐to‐use fluoroalkylating reagents is highly desirable. In addition, realization of mono‐, di‐, tri‐, or polyfluoroalkyation usually requires distinct types of fluoroalkylating reagents under different or even harsh reaction conditions, and a universal method to accomplish different hydrofluoroalkylation of alkenes is lacking. Herein, the use of quaternary fluoroalkyl alcohols is reported as the universal fluoroalkylating reagents to readily facilitate mono‐, di‐, tri‐, or polyfluoroalkylation of a wide range of alkene substrates in high yields. Moreover, a cascade reaction of hydrofluoroalkylation followed by intramolecular fluoroalkylation facilitates the construction of a variety of high‐value complex heterocycles bearing diverse fluoroalkyl functionalities from alkenes. Mechanistic studies suggest that a proton‐coupled electron transfer (PCET) process may be involved through a radical‐generating pathway. The utility of this method is showcased by the late‐stage fluoroalkylation of various high‐value complex molecules derived from either natural products or drug‐like compounds. Of note is that a continuous‐flow system is amenable to this homogeneous photoredox conditions, thereby opening up a possibility of using this protocol to realize large‐scale manufacturing of fluoroalkyl products with industrial interests.

## Graphical abstract

A universal photoredox‐catalyzed fluoroalkylation approach by utilizing new (fluoroalkyl)diaryl methanols as versatile fluoroalkylating reagents is developed. This protocol facilitates the synthesis of mono‐, di‐, tri‐, and even polyfluoroalkylation of a broad scope of alkenes. Furthermore, various alkene substrates derived from complex natural products and bioactive compounds are well tolerated for the fluoroalkylation reactions as late‐stage functionalizations.

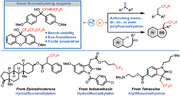

## 1 Introduction

**Figure 1:**
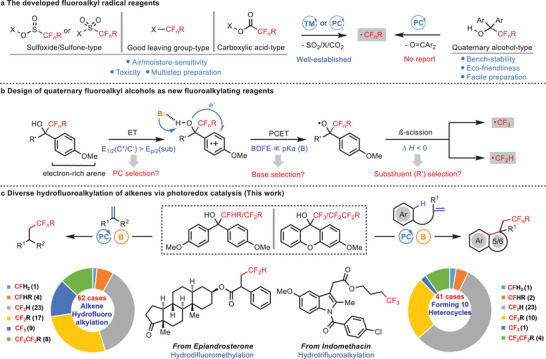
Design and development of fluoroalkylating reagents. a) The developed fluoroalkyl radical reagents. b) Design of quaternary fluoroalkyl alcohols as new fluoroalkylating reagents. c) Diverse hydrofluoroalkylation of alkenes via photoredox catalysis. TM, transition metal; PC, photocatalyst; B, base; BDFE, bond‐dissociation free energy; PCET, proton‐coupled electron transfer; HAT, hydrogen atom transfer.

The incorporation of fluoroalkyl groups, such as difluoromethyl (CF_2_H), trifluoromethyl (CF_3_), or polyfluoroalkyl (CF_2_CF_2_R) into a bioactive molecule may lead to essential improvements in the physicochemical property, target interaction affinity, lipophilicity, membrane permeability, and metabolic stability.^[^ ^1^, ^2^, ^3^ ^]^ As a consequence, development of efficient and practical strategies for fluoroalkylation is of great significance in drug discovery.^[^ ^4^, ^5^ ^]^ Among these, the radical fluoroalkylation represents a promising strategy because of its compatibility with various heterocyclic substrates and the use of mild reaction conditions.^[^ ^6^, ^7^ ^]^ Accordingly, a range of fluoroalkylating reagents bearing sulfoxide/sulfones,^[^ ^8^, ^9^, ^10^, ^11^, ^12^ ^]^ good leaving groups,^[^ ^13^, ^14^, ^15^, ^16^, ^17^, ^18^, ^19^, ^20^, ^21^, ^22^ ^]^ and carboxylic acid‐type counterparts^[^ ^23^, ^24^, ^25^, ^26^ ^]^ have been developed by Baran, Umemoto, Hu, Togni, and other groups (**Figure** **1**a). However, these fluoroalkylating reagents have certain limitations, including sensitivity to air or moisture, toxicity, and multistep synthesis. In addition, the conventional methods often require distinct types of fluoroalkylating reagents under different reaction conditions. Therefore, the development of a novel, practical, and universal fluoroalkylating source to allow for introducing all the mono‐ to polyfluoroalkyl (CFH_2_, CF_2_H, CF_3,_ CF_2_CF_2_R) functionalities under the same reaction system is highly desirable.

The generation of alkoxy radical from simple alcohols represents a common but challenging task.^[^ ^27^ ^]^ The O─H bond in alcohol generally has strong bond dissociation free energy (BDFE) (≈105 kcal mol^−1^), which is energetically unfavorable for direct generation of alkoxy radical through hydrogen atom transfer (HAT).^[^ ^28^ ^]^ However, the proton‐coupled electron transfer (PCET) strategy offers an option to overcome the energetic barrier.^[^ ^29^ ^]^ In this regard, we surmise that a quaternary fluoroalkyl alcohol might produce fluoroalkyl radical species through the PCET process followed by *β*‐scission of C─C bond (Figure 1b). More specifically, the arene radical cation might be first generated through oxidation by the excited state of an appropriate photocatalyst via an electron transfer (ET) event. A PCET process then occurs in the presence of a suitable base to afford an alkoxy radical which is subsequently converted to the fluoroalkyl radical through a *β*‐scission step by releasing a ketone side product. Photoredox‐enabled *β*‐scission has been well studied for cyclic alcohols via strain‐release,^[^ ^30^, ^31^, ^32^, ^33^ ^]^ however, the same strategy is rarely used for linear alcohols.^[^ ^34^, ^35^, ^36^, ^37^ ^]^ In addition, the generation of CF_2_H and CF_3_ radicals through *β*‐scission is typically exergonic, thus alcohol substrates bearing appropriate substituents might be helpful. Based on these analyses, we herein report the development of both acyclic and cyclic quaternary fluoroalkyl alcohols as novel radical fluoroalkylating reagents, exhibiting advantageous features such as bench‐stability, eco‐friendliness, and ease of preparation. More importantly, these reagents present broad applicability in introducing diverse mono‐, di‐, tri‐, and even polyfluoroalkyl groups to given molecules, including the formation of over ten kinds of fluoroalkylated heterocycles through photoredox catalysis (Figure 1c). Compared to alternative fluoroalkyl acetic acids, fluoroalkyl alcohol reagents do not require noble transition metal pho‐tocatalysts for the transformation reactions and can tolerate acid‐sensitive functional groups, thus rendering it more practical for late‐stage fluoroalkyl editing of a biologically interesting compound.^[^ ^24^, ^35^, ^38^ ^]^ As such, by setting up this approach in a continuous‐flow system, large‐scale manufacturing of valuable fluoroalkyl products is facilitated efficiently.

## 2 Results and Discussion

**Figure 2:**
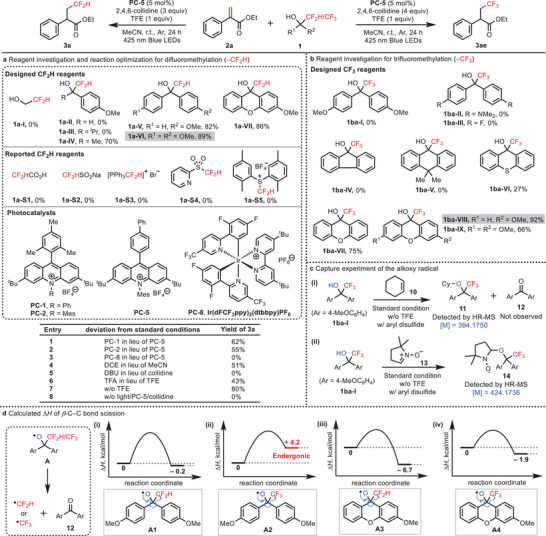
Reagent investigation and condition optimization. a) Reagent investigation and reaction optimization for difluoromethylation. b) Reagent investigation for trifluoromethylation. c) Capture experiment of the alkoxy radical. d) Calculated Δ*H* of *β*‐C─C bond scission. Reaction conditions: **1** (0.4 mmol), **2a** (0.2 mmol), **PC** (5 mol%), 2,4,6‐collidine (3.0 equiv. or 4.0 equiv.), TFE (1.0 equiv.), MeCN (2.0 mL), 425 nm blue LEDs, 24 h. Yields were determined by GC‐MS with *n*‐dodecane as an internal standard. Isolated yield in parentheses. n.d., not detected; w/, with; w/o, without; i/o, instead of; DCE, 1,2‐dichloroethane; DBU, 1,8‐diazabicyclo[5.4.0]undec‐7‐ene; TFA, trifluoroacetic acid; TFE, trifluoroethanol.

Our studies commenced with the investigation of difluoromethylation of alkenes (**Figure** **2**a). The model reaction was carried out with ethyl 2‐phenylacrylate (**2a**) as the substrate, 2,2‐ difluoroethanol (**1a‐I**) as the fluoroalkylating reagent, 5 mol % of PC‐5 as the photocatalyst, 3.0 equiv. of 2,4,6‐collidine as the base and 1.0 equiv. of 2,2,2‐trifluoroethanol as the proton source in MeCN under argon atmosphere and blue LEDs irradiation (see Supplementary Information for details). Unfortunately, the reaction failed to yield the desired product **3a**. The failure might be ascribed to the high oxidation potential of **1a‐I** (E_p/2_ = 2.19 V vs SCE, see Supporting Information), which prevents the formation of an arene radical cation through single electron transfer.^[^ ^29^ ^]^ Subsequently, the arylsubstituted difluoromethyl tertiary and quaternary alcohols **1a‐II** and **1a‐III** were subjected to the same reaction. The relatively lower oxidation potentials of these two reagents (E_p/2_ = 1.72 and 1.70 V vs SCE, respectively) facilitated the occurrence of oxidation by the photocatalyst through an electron transfer event. Further, the effective BDFEs of the oxidant/base combinations exceed the O−H BDFE of the substrate (≈ 105 kcal mol^−1^, see Supplementary Information), leading to the generation of alkoxy radicals via a PCET process. This was confirmed by the detection of 1‐(4‐methoxyphenyl)‐2,2‐difluoroethan‐1‐one as a side product. Unfortunately, the anticipated *β*‐scission to deliver difluoromethyl radical did not occur subsequently. Interestingly, methyl‐substituted quaternary alcohol **1a‐IV** can be used to obtain product **3a** in 70% yield. In addition, the use of α,α‐diaryl difluoroethanols **1a‐V** to **1a‐VI** afforded product **3a** in 82% and 89% yields, respectively, indicating that the α,α‐diaryl substituents assisted the *β*‐scission to form the desired difluoromethyl radical species. Subsequently, a range of previously reported difluoromethylating reagents, such as CF_2_HCOOH and CF_2_HSO_2_Na, were extensively investigated but did not generate the desired product **3a**. Various photocatalysts were then screened with **1a‐VI** as the difluoromethylating reagent. Ir‐based photocatalyst **PC‐8** (E_ox_ = +0.89 V vs SCE)^[^ ^39^ ^]^ failed to deliver product **3a** (see Supplementary Information), whereas the organophotocatalyst **PC‐5** was identified as the optimal choice. In addition, various reaction parameters, including bases, hydrogen donors, and solvents, were systematically evaluated (see Supplementary Information). A moderate yield was obtained in the presence of DCE as the solvent. No product was detected when DBU was used as a base. Switching the proton source from trifluoroethanol (TFE) to trifluoroacetic acid (TFA) reduced the yield. Moreover, control experiments revealed a slightly diminished yield in the absence of TFE. However, no product was detected in the absence of light, **PC‐5**, or collidine, suggesting that these components are indispensable in the photoredox reaction.

The success of difluoromethylation of alkenes using **1a‐VI** as the difluoromethylating reagent prompted us to explore the possibility of trifluoromethylation by using a similar quaternary alcohol‐type trifluoroalkylating reagent. As depicted in Figure 2b, with the 1,1‐diaryl 2,2,2‐trifluoroethanol (**1ba‐I**) as the fluoromethylating reagent, the reaction with alkene **2a** under the optimized condition as above failed to deliver the desired product **3ae**. In addition, the two trifluoroethanol variants **1ba‐II** and **1ba‐III** also failed to give the expected product. We envisioned that *β*‐scission of the alkoxy radical generated from **1ba‐I** is probably endergonic and needs higher activation energy. To overcome this challenge, a range of trifluoromethyltricyclic quaternary alcohols **1ba‐IV** to **1ba‐VII** were investigated, and only **1ba‐VII** was found capable of generating tr fluoromethyl product **3ae** in 75% yield. Delightfully, the quaternary alcohol **1ba‐VIII** bearing a methoxy substituent on the phenyl gave a higher yield of 92%, whereas the symmetric dimethoxy substituted alcohol **1ba‐IX** yield a lower outcome. Likewise, we were curious that whether the difluoromethylation reaction could be further improved by using a similar tricyclic difluoromethylating reagent **1a‐VII**. It was found that the reaction with **1a‐VII** indeed proceeded smoothly and afforded the product **3a** in 86% yield, which is comparable to that using **1a‐VI** as the difluoromethylating reagent (89%).

To rationalize the different reactivity of these trifluoro‐methylating reagents, we first examined whether the alkoxy radical from **1ba‐I** can be generated under the standard condition (Figure 2c). The two radical capture experiments were carried out and the radical adducts **11** and **14** were detected by HR‐MS, indicating that the alkoxy radical was successfully produced. In Figure 2c‐i, we failed to detect ketone **12** indicating that the subsequent *β*‐scission did not occur. We then calculated the enthalpy values associated with *β*‐C─C bond scission of alkoxy radicals **A1** to **A4** (Figure 2d). The C─C cleavage of the resulting radical **A2** is endergonic by ≈ 4.2 kcal mol^−1^ and cannot form CF_3_ radical, which is consistent with our experimental observation. In addition, the exergonic *β*‐scission of alkoxy radicals **A1**, **A3**, and **A4** led to the readily formation of corresponding difluoromethylated and trifluomethylated products. In addition, Potentials of hydrogenation on the PCET process, denoted E°(V vs H_2_), has been calculated, BDEs and BDFEs of the carbon‐fluorinated carbon bond have also been calculated to provide further insights (see Table S7, Supporting Information).^[^ ^40^, ^41^ ^]^

**Figure 3:**
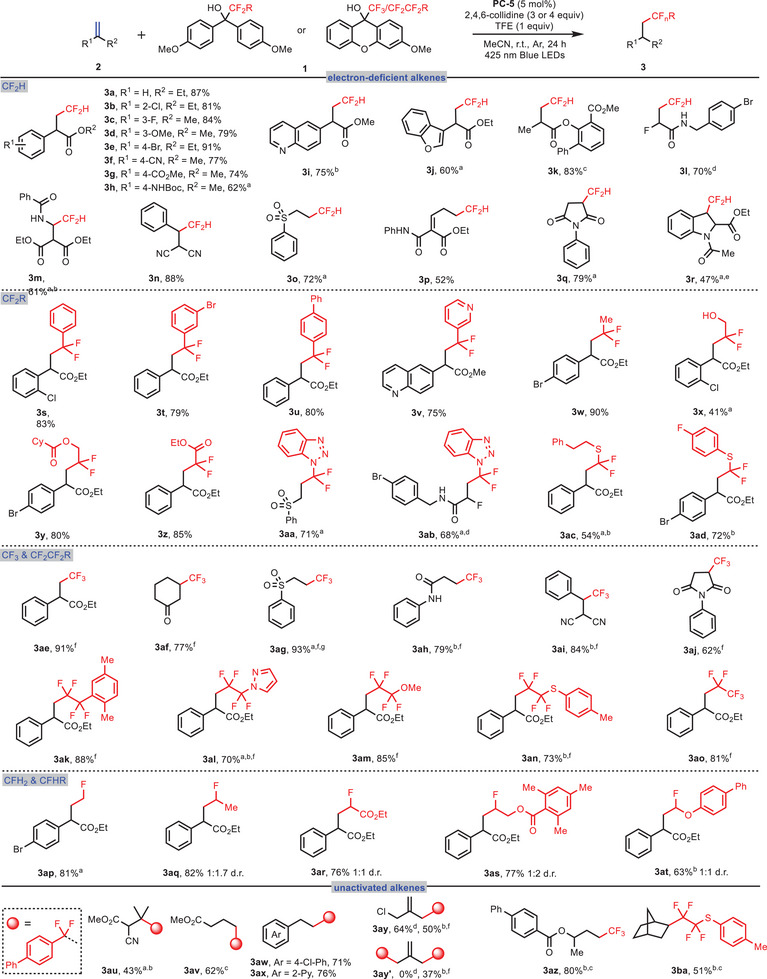
Substrate scope for alkene hydrofluoroalkylation. Reaction conditions: 2 (0.2 mmol), 1 (0.4 mmol), **PC‐5** (5 mol%), 2,4,6‐collidine (3.0 equiv.), TFE (1.0 equiv.), MeCN (2.0 mL), 425 nm blue LEDs, 24 h. *^a^* 48 h. *^b^* 10 mol% **PC‐5**. *^c^* **1** (0.2 mmol), **2** (0.1 mmol), **PC‐5** (5 mol%), 2,4,6‐collidine (4.0 equiv.), K_2_S_2_O_8_ (3.0 equiv.), MeCN (1.5 mL), H_2_O (0.5 mL), 425 nm blue LEDs, 48 h. *^d^* **1** (1.0 equiv.), **2** (2.0 equiv.). *^e^* 60 h. *^f^* **1** (3.0 equiv.), 2,4,6‐collidine (4.0 equiv.). *^g^* 50 °C.

With the optimal reaction conditions established, we sought to evaluate the substrate scope and tolerance of various alkenes (**Figure** **3**). First, we explored the hydrodifluoromethylation of 2‐arylacrylates bearing diverse electron‐withdrawing and electron‐donating substituents on the phenyl ring. The reactions went through smoothly, affording the corresponding difluoromethylated products **3a**‐**h** in 62–91% yields. In addition, quinoline‐6‐yl‐, benzofuran‐3‐yl, or simple methyl or fluoro‐substituted acrylates were also found well tolerated and the products **3i**‐**l** were obtained in good yields. Moreover, tri‐substituted alkenes, vinyl sulfones, and even 1,3‐dienes were also applicable to this protocol to provide the products **3m**‐**p** in 52–88% yields. Intriguingly, the maleimide and indole substrates as cyclic alkene variants were also suitable for this reaction and the corresponding hydrodifluoromethylated products **3q** and **3r** were generated in 79% and 47% yields, respectively.

In parallel with hydrodifluoromethylation, aryl/heteroaryl/alkyl difluoromethylation of alkenes were also realized by using appropriate difluoroalkylating reagents. As shown in Figure 3, the aryl/heteroaryl difluoromethylated products **3s**‐**v** were obtained in 75–83% yields. Interestingly, simple (methyl)difluoromethyl diarylmethanol was also applicable to the reaction and the product **3w** was obtained in 90% yield. The (hydroxymethyl)difluoromethyl diarylmethanol also survived in this reaction, although affording the corresponding product **3x** in lower yield (41%). Appealingly, more sophisticated difluoromethylation bearing esteric, benzotriazol‐, or alkylsulfane functionalities were also realized to provide corresponding products **3y**‐**3ad** in good yields.

The substrate scope for trifluoromethylation of various alkene substrates was then investigated. As shown in Figure 3, products **3ae‐3aj** were obtained in 62–93% yields by using appropriate trifluoromethylating reagents. Interestingly, valuable polyfluoroalkylated products **3ak‐3ao** were also achieved in good yields by using appropriate (fluoroalkyl)difluoromethyl alcohols as the fluoroalkylating reagents.

In addition, monofluoromethylation was also readily accomplished by using various monofluoromethylating reagents bearing diverse functionalities (**3ap‐3at**). To further demonstrate the versatility of the reaction, various unactivated alkenes, such as tetra‐substituted alkene, but‐3‐enoate, styrene, and norbornene, were subjected to the reaction conditions, and the corresponding difluoro‐, trifluoro‐, and polyfluoroalkylated products **3au**‐**3ba** were conveniently obtained albeit in somewhat lower yields (43‐80%). Notably, a further elimination of HCl occurred to provide mono‐ and difluoroalkylation products **3ay** and **3ay’** in the case of 3‐chloro‐2‐(chloromethyl)prop‐1‐ene.

**Figure 4:**
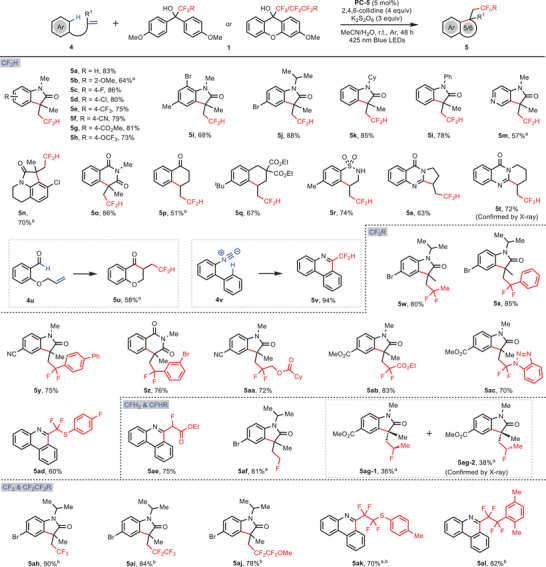
Intramolecular arylfluoroalkylation of alkenes to access diverse heteroarenes. Reaction conditions: **1** (0.2 mmol), **4** (0.1 mmol), **PC‐5** (5 mol%), 2,4,6‐collidine (4.0 equiv.), K_2_S_2_O_8_ (3.0 equiv.), MeCN (1.5 mL), H_2_O (0.5 mL), 425 nm blue LEDs, 48 h. *^a^* **PC‐5** (10 mol%). *^b^* **1** (3.0 equiv.).

Encouraged by the success of various hydrofluoroalkylation of alkenes, we then investigated the feasibility of intramolecular fluoroalkylation, which could build up a series of high value complex heterocycles bearing fluoroalkyl functionality (**Figure** **4**). To this end, a series of N‐phenylacrylamides or N‐(pyridin‐4‐yl)acrylamides were employed as the alkene substrates. After fine‐tuning the reaction conditions by replacing the proton donor TFE with an oxidant K_2_S_2_O_8_, the corresponding indolinone analogues **5a**‐**m** were obtained readily in 57–86% yields. In the case of N‐acryloyl 3,4‐dihydroquinoline as the fused *N*‐arylacrylamide substrate, a tricyclic difluoromethylated product **5n** was obtained in 70% yield. This cascade reaction was also applicable to construct other aryl‐fused bicyclic and tricyclic heterocycles **5o‐5t** in 51–86% yields. Interestingly, 2‐(allyloxy)benzaldehyde was also proved as a suitable substrate and the corresponding 3‐(2,2‐difluoroethyl)chroman‐4‐one **5u** was generated in 58% yield. Notably, biphenyl isocyanide also participated in the cascade reaction to afford the difluoromethylated phenanthridine **5v** in 94% yield.

To further extend the product diversity, various difluoroalkylating reagents were employed, leading to formation of difluoroalkylated heterocycles **5w‐5ad** in good yields. Meanwhile, intramolecular monofluoroalkylation was also easily realized by using diverse monofluoroalkylating reagents and the corresponding products **5ae**‐**5ag** were obtained in 74–81% yields. In the case of product **5ag**, a pair of diastereomers (**5ag‐1** and **5ag‐2**) were readily separated in 1:1 ratio, and the structure of **5ag‐2** was confirmed by X‐ray analysis. Similarly, trifluoromethylating and multifluoroalkylating reagents were also applicable to these substrates to furnish the desired indolinones **5ah‐5aj** and phenanthridine **5ak** and **5al** in good yields.

**Figure 5:**
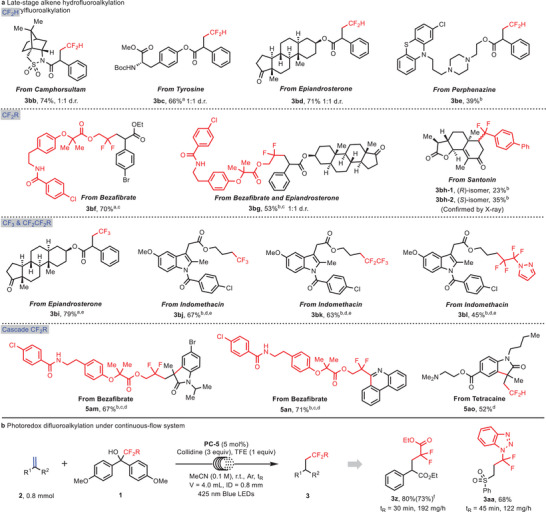
Synthetic applications. a) Late‐stage hydrofluoroalkylation and arylfluoroalkylation. b) Photoredox difluoroalkylation under a continuous‐flow system. Reaction conditions: **1** (0.2 mmol), **2** (0.1 mmol), **PC‐5** (5 mol%), 2,4,6‐collidine (3.0 equiv.), TFE (1.0 equiv.), MeCN (2.0 mL), 425 nm blue LEDs, 24 h. *^a^* 48 h. *^b^* **PC‐5** (10 mol%). *^c^* **1** (1.0 equiv.), **2** (2.0 equiv.). *^d^* **1** (0.2 mmol), **4** (0.1 mmol), **PC‐5** (5 mol%), 2,4,6‐collidine (4.0 equiv.), K_2_S_2_O_8_ (3.0 equiv.), MeCN (1.5 mL), H_2_O (0.5 mL), 425 nm blue LEDs, 48 h. *^e^* **1** (0.3 mmol). *^f^* For the yield of **3z** in parentheses, using 4.0 mmol scale, t_R_ = 30 min, 175 mg/h.

To further illustrate the practicability of the universal fluoroalkylating strategy late‐stage functionalization of diverse complex molecules was carried out, including derivatives of natural products and clinical drugs (**Figure** **5**a). First, the hydrodifluoromethylation was smoothly conducted on phenylacrylamides or phenylacrylates derived from the anticancer drug camphorsultam, tyrosine, epiandrosterone, and perphenazine to deliver products **3bb**‐**3be** in moderate to good yields. Interestingly, the complex fluoroalkylating reagent from lipid‐lowering drug bezafibrate^[^ ^42^ ^]^ also reacted with phenylacrylate or epiandrosterone analogues to afford the corresponding products **3bf** and **3bg** in 70% and 53% yields, respectively. The hydrodifluoroalkylation of santonin,^[^ ^43^ ^]^ a complex natural product with multiple biological activities, also performed well to provide a pair of diastereomers **3bh‐1** and **3bh‐2**. The absolute configuration of **3bh‐2** was confirmed by X‐ray crystal analysis (CCDC 2269262). The trifluoromethylating and polyfluoroalkylating reagents were demonstrated applicable as well to the reaction with epiandrosterone and indomethacin derivatives, and the corresponding products **3bi‐3bl** were obtained in good yields. In addition, bezafibrate‐derived fluoroalkylating reagent was also survived well for intramolecular cascade reaction to provided indolinone **5am** and phenanthridine **5an** in 67% and 71% yields, respectively. Meanwhile, the N‐arylacrylamide derived from the local anesthetic tetracaine^[^ ^44^ ^]^ also participated in the cyclization reaction, producing the corresponding derivative **5ao** in 52% yield.

To showcase the manufacturing potential of this protocol, we transferred our fluoroalkylation reaction to a continuous‐flow system^[^ ^45^ ^]^ (Figure 5b). In the flow reactor, the reaction time could be shortened to 30–45 min and the desired fluoroalkylated products **3z** and **3aa** were obtained in good yields. Furthermore, the difluoroalkylation reaction was successfully scaled up to 4 mmol by using this continuous‐flow technology without significant sacrifice of the yield, highlighting the large manufacturing potential of the present fluoroalkylation method.

**Figure 6:**
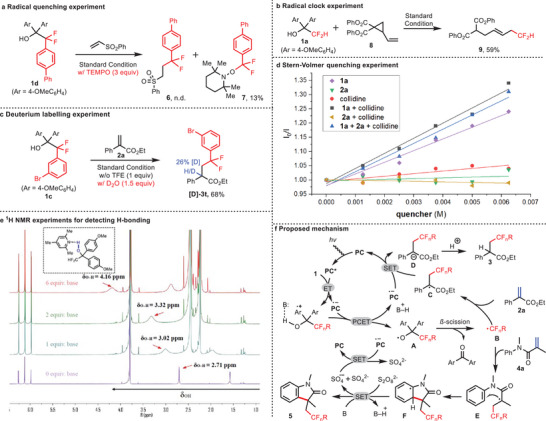
Mechanistic studies. a) Radical quenching experiment. b) Radical clock experiment. c) Deuterium labelling experiment. d) Stern‐Volmer quenching experiment. e) ^1^H NMR experiment for detecting H–bonding. f) Proposed mechanism.

To gain more insights into the fluoroalkylation protocol, a few preliminary mechanistic studies were performed. First, we carried out the reaction of (vinylsulfonyl)benzene with difluoroalkyl alcohol **1d** under the standard conditions in the presence of the radical scavenger 2,2,6,6‐tetramethylpiperidin‐1‐oxyl (TEMPO). Only the TEMPO‐difluoroalkyl adduct **7** was isolated, albeit in low yield, supporting that a radical process is involved in the reaction (**Figure** **6**a). Second, the radical process was further demonstrated through a radical clock experiment in the difluoromethylating reaction of 2‐vinylcyclopropane‐1,1‐dicarboxylate **8** (Figure 6b). During the process, difluoromethylation was followed by a cyclopropane‐opening event, leading to product **9** in 59% yield. Meanwhile, in the reaction of 2‐phenylacrylate **2a** with aryldifluoromethanol **1c**, deuterated water was used to replace TFE. The deuterated product **[D]‐3t** was obtained in 68% yield, indicating that the protonation process may involve a carbanion intermediate and TFE served as a proton source (Figure 6c). In addition, in order to determine which component could quench the excited state of photocatalyst **PC‐5,** Stern‐Volmer fluorescence quenching experiments were conducted in the mode reaction of **1a** and **2a**. Six curves were plotted for different concentrations of **1a**, **2a**, 2,4,6‐collidine and their various combinations. These outcomes clearly demonstrated that the excited state of **PC‐5** was quenched efficiently by fluoroalkylating reagent **1a** (Figure 6d).^[^ ^46^ ^]^ The PCET process is proposed by the formation of an intermolecular H–bonding interaction between the external base and quaternary alcohol reagent. This process was validated by treating the fluoromethylating reagent **1a‐VI** with incremental amounts of 2,4,6‐collidine and then detected the chemical shift changes of **1a‐VI** by ^1^H NMR spectra analysis. The observed downfield shifting of the hydroxyl of **1a‐VI** with the incremental amount of base suggested the formation of an H–bonding adduct between **1a** and 2,4,6‐collidine (Figure 6e).

Based on these mechanistic experiments and the precedent literatures,^[^ ^31^, ^35^ ^]^ a plausible catalytic cycle is presented in Figure 6f. The excited state of photocatalyst **PC** engages in a PCET process with the base‐alcohol adduct to generate the alkoxy radical **A**. A *β*‐C─C cleavage step then occurs leading to the formation of fluoroalkyl radical **B** along with the release of ketone **4**. The fluoroalkyl radical **B** then reacts with **2a** through a radical nucleophilic addition to deliver the radical species **C**. The carbanion **D** is subsequently formed via an electron transfer process, accompanied by the closure of the photoredox catalytic cycle. Finally, protonation of intermediate **D** provides the desired fluoroalkylation product **3**. Alternatively, treating fluoroalkyl radical **B** with substrate **4a** generates radical species **E**, which then undergoes cyclization to form intermediate **F**. Aromatization occurs in the presence of the oxidant K_2_S_2_O_8_, providing the cyclization product **5**.

## 3 Conclusion

In conclusion, we have developed a universal photoredox‐catalyzed fluoroalkylation approach by utilizing new (fluoroalkyl)diaryl methanols as versatile fluoroalkylating reagents. This protocol facilitates the synthesis of mono‐, di‐, tri‐, and even polyfluoroalkylation of a broad scope of alkenes. In particular, a cascade process encompassing fluoroalkylation followed by intramolecular cyclization is readily realized leading to formation of valuable complex heterocycles. Furthermore, various alkene substrates derived from complex natural products and bioactive compounds are well tolerated for the fluoroalkylation reactions as late‐stage functionalizations. Remarkably, such fluoroalkylating protocol is conveniently transferred to a continuous‐flow process, further validating the potential utility for industrial‐scale manufacturing.

## 4 Experimental Section

### General Procedure for the Synthesis of **3**

To a 4 mL vial equipped with a stir bar was added fluorinated alcohol **1** (0.4 mmol, 2.0 equiv.), PC‐5 (6.5 mg, 0.01 mmol, 5 mol%) and electron‐withdrawing alkene **2** (0.2 mmol, 1.0 equiv.). The vial was sealed, evacuated, and backfilled with Argon three times, then TFE (14 µL, 0.2 mmol, 1.0 equiv.) collidine (78 µL, 0.6 mmol, 3.0 equiv.) and 2 mL of dry MeCN (2 mL) were added. After degassing with Argon balloon for 8 min, the reaction mixture was irradiated with 10 W blue LEDs lamps for 24 h at ambient temperature. The reaction mixture was then concentrated and purified on a preparative TLC with petroleum ether/ethyl acetate as the eluent to afford the product **3**.

### General Procedure for the Synthesis of **5**

To a 4 mL vial equipped with a stir bar was added fluorinated alcohol **1** (0.2 mmol, 2.0 equiv.), PC‐5 (3.3 mg, 5 µmol, 5 mol%), K_2_S_2_O_8_ (81.0 mg, 0.3 mmol, 3.0 equiv.) and alkenes **4** (0.1 mmol, 1.0 equiv.) The vial was sealed, evacuated and backfilled with Argon three times, then collidine (52 µL, 0.4 mmol, 4.0 equiv.) and 1.5 mL MeCN and 0.5 mL H_2_O were added. After degassing with Argon balloon for 8 min, the reaction mixture was irradiated with 10 W blue LEDs lamps for 48 h at ambient temperature. The reaction mixture was then concentrated and purified on a preparative TLC with petroleum ether/ethyl acetate as the eluent to afford the products **5**.

## Conflict of Interest

The authors declare no conflict of interest.

## Author Contributions

H.L. developed the fluoroalkylating reagents, optimized the conditions, and investigated the substrate scope. Y.Z. per‐formed the calculation studies. H.L., Z.F., and A.Z. wrote the manuscript. A.Z. supervised the project. A.Z and H.L. are inventors on a patent application related to this work (CN Patent application 2024107554524) filed by Shanghai Jiao Tong University.

## Supporting information



Supporting Information

## Data Availability

The data that support the findings of this study are available in the supplementary material of this article.
