# Supplementary material for: Diversified Fluoroalkylation of Alkenes Using Quaternary Fluoroalkyl Alcohols as the Fluoroalkylating Reagents
Source: Adv Sci (Weinh). 2024 Dec 24;12(6):2408781. doi: 10.1002/advs.202408781 (PMC11809345; doi:10.1002/advs.202408781)
Supplement: Supplementary file 1 — Supporting Information [file ADVS-12-2408781-s001.pdf]

ADVANCED SCIENCE
Open Access

Supporting Information

for *Adv. Sci.*, DOI 10.1002/advs.202408781

Diversified Fluoroalkylation of Alkenes Using Quaternary Fluoroalkyl Alcohols as the Fluoroalkylating Reagents

*Heng Lu, Zhoulong Fan, Yike Zou and Ao Zhang**

Supporting Information

Diversified Fluoroalkylation of Alkenes Using Quaternary Fluoroalkyl Alcohols as the Fluoroalkylating Reagents

Authors: Heng Lu,[1,2,3] Zhoulong Fan,[1,2] Yike Zou,[1,2] Ao Zhang[1,2,3,*]

Affiliations: [1]Shanghai Frontiers Science Center of Drug Target Identification and Delivery, School of Pharmaceutical Sciences, Shanghai Jiao Tong University, Shanghai 200240, China.

[2]National Key Laboratory of Innovative Immunotherapy, Shanghai Jiao Tong University, 800 Dongchuan Road, Minhang District, Shanghai 200240, China.

[3]Frontiers Science Center for Transformative Molecules, Shanghai Jiao Tong University, Shanghai 200240, China.

*Correspondence to: ao6919zhang@sjtu.edu.cn

Table of Contents

1. General experimental information--S1

2. General synthetic procedure for starting materials---S3

2.1 General procedure for synthesis of fluoroalkyl reagents 1-------------------------------S4

2.2 General procedure for synthesis of alkenes 2---S28

2.3 General procedure for synthesis of alkenes 4---S32

3. Condition optimization---S35

4. General procedures for compound synthesis ---S50

4.1. Representative experimental procedure for synthesis of compounds 3----------------S50

4.2 Representative experimental procedure for synthesis of compounds 5----------------S83

5. Continuous-flow synthesis of fluorinated-compounds ---------------------------------S103

6. Preliminary mechanistic experiments--S104

6.1 Radical inhibition experiment--S104

6.2 Radical clock experiment--S105

6.3 Radical trapping experiment--S105

6.4 Deuterium labeling experiment--S108

6.5 Stern-Volmer Studies--S109

6.6 ^{1}H NMR experiment---S111

6.7. Light/dark experiment---S111

7. General Computational Procedure ---S113

8. References ---S119

9. Copies of NMR spectra data --S121

10. X-Ray crystallographic data --S361

SUPPORTING INFORMATION

1. General experimental information

Unless otherwise stated all reactions were set up under Ar atmosphere utilizing oven-dried glassware. Liquids and solutions were transferred with syringes. Solvents were purchased from J&K (Sure/Seal bottles) or dried with activated 4 Å molecular sieves. All other reagents were purchased from various commercial sources and used as received. A blue LEDs panel (λ = 425 nm, 10 W, brand: jiadeng https://m.tb.cn/h.5e890bV?tk=f7MZdyB5ArO) was used as the light source for all the photoredox catalyzed reactions. ^{1}H, ^{19}F and ^{13}C NMR spectra were recorded in $CDCl_3$ unless otherwise noted, on an Agilent 400MHz, Bruker 400 MHz or Bruker 700 MHz spectrometer. Chemical shifts in ^{1}H NMR spectra were reported in parts per million (ppm) on the δ scale from an internal standard of residual chloroform (7.26 ppm). Data for ^{1}H NMR were reported as follows: chemical shift, multiplicity (s = singlet, d = doublet, t = triplet, q = quartet, m = multiplet, br = broad), coupling constant in Herts (Hz) and integration. Data for ^{13}C NMR spectra were reported in terms of chemical shift in ppm from the central peak of chloroform (77.16 ppm). High resolution mass spectra (HRMS) were performed at Instrumental Analysis Center of Shanghai Jiao Tong University with electrospray spectrometer Waters Micromass Q-TOF Premier Mass Spectrometer. Flash column chromatography on silica gel (200 - 300 mesh) or RP-C18. The column output was monitored by TLC on silica gel (100 - 200 mesh) precoated on glass plates (15 x 50 mm), and spots were visualized by UV light at 254 nm. Cyclic Voltammetry Studies were recorded on a CHI 600E electrochemical workstation. Stern-Volmer luminescence quenching experiments were conducted on an INESA 930F fluorescence spectrophotometer.

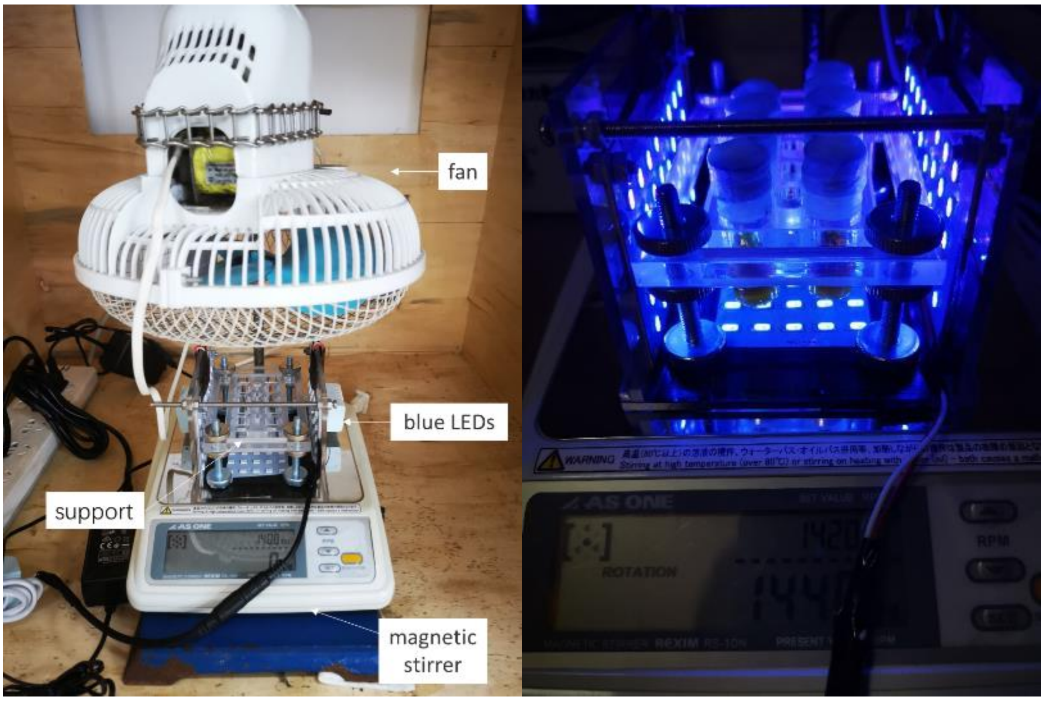

Figure S1. Photoreactor used in this manuscript

2. General synthetic procedure for starting materials

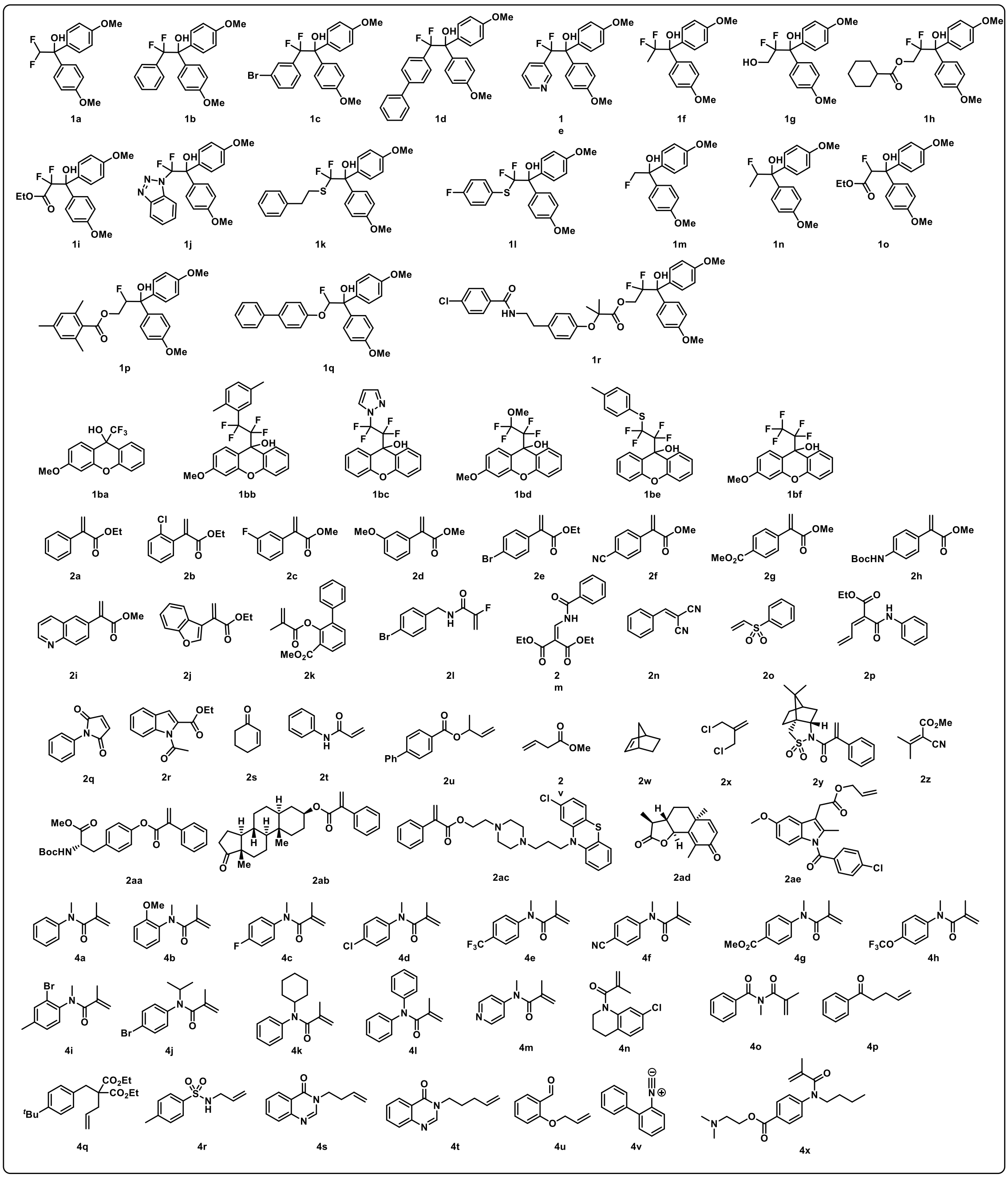

Materials: All reagents were used as received from commercial sources or prepared as described in the literature. All reagents were weighed and handled under air, and refilled with an inert atmosphere of Ar at room temperature. Compounds **2n, 2o, 2q, 2s, 2t, 2v, 2w, 2x, 2z** and **2ad**were commercially availabled. Compounds **1s**[1], **1j**[2],**2a**[3], **2b**[3], **2c**[4], **2d**[5], **2e**[3], **2f**[5], **2g**[4], **2i**[6], **2l**[7], **2r**[8], **2y**[9],**2ae**[10], **4a**[11], **4b**[12], **4c**[11], **4d**[11], **4e**[11], **4f**[11], **4g**[12], **4h**[13], **4m**[14], **4j**[15], **4k**[16], **4l**[12], **4s**[17], **4t**[17], **4q**[18], **4o**[19], **4p**[20], **4r**[21], **4u**[22] and **4v**[23] were prepared according to the reported literatures.

2.1 General procedure for synthesis of fluoroalkyl reagents 1

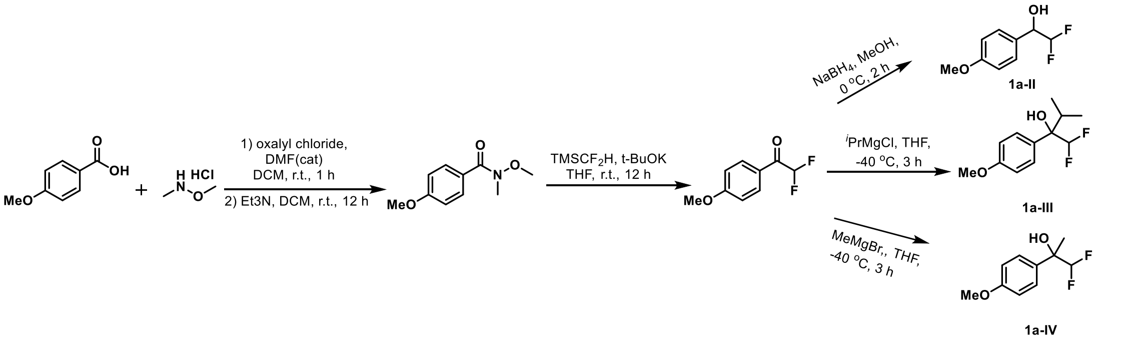

4-Methoxybenzoic acid (10 mmol, 1.0 equiv.) was dissolved in distilled DCM (30 mL) containing one drop of DMF at 0°C. Oxalyl chloride (0.93 mL, 10 mmol, 1.0 equiv.) was slowly added and the mixture was stirred for 1 h at room temperature. Then, the solvent was removed under vacuum and the resulting crude acyl chloride was dissolved in dry DCM (20 mL). N,O-Dimethylhydroxylamine hydrochloride (10.1 mmol, 1.01 equiv.) was added slowly followed by addition of Et_3N (30 mmol, 3.0 equiv.). The reaction mixture was stirred for 14 h at room temperature. Water (60 mL) was then added, and the organic layer was separated. The aqueous phase was extracted with DCM (2 x 30 mL), and the combined organic layer was washed with HCl 1M (2 x 50 mL), water (50 mL), dried over $MgSO_4$, filtered and concentrated under vacuum. The expected Weinreb amide was used without further purification.

The N-(4-dimethoxy)-N-methylbenzamide (10 mmol, 1.0 equiv.) was dissolved in distilled THF (100 mL) at 0°C under argon atmosphere. $TMSCF_2H$ (20 mmol, 2.0 equiv.) was added at 0°C followed by addition of a *t*-BuOK (0.9 M in cyclohexane, 18 mmol, 1.8 equiv.) solution over 15 minutes. The reaction mixture was stirred for 4 h at room temperature then NH_4Cl (40 mL) and Et_2O (100 mL) were added. The organic layer was separated and the aqueous layer was extracted with Et_2O twice (60 mL). The combined organic layer was was washed with brine (100 mL), dried over $MgSO_4$, filtered and concentrated under vacuum. The residue was purified by flash chromatography to afford 1-(4-methoxyphenyl)-2,2-difluoroethan-1-one (1.72 g, 92% yield) as a colorless solid. The collected analytical data are in agreement with those reported in the literature.[25] ^1H NMR (400 MHz, $CDCl_3$) δ 8.05 (d, J = 9.0 Hz, 2H), 7.02-

6.85 (d, J = 9.0 Hz, 2H), 6.24 (t, J = 53.7 Hz, 1H), 3.89 (s, 3H).^{19}F NMR (376 MHz, $CDCl_3$) δ -121.38 (d, J = 53.8 Hz, 2F).

1-(4-Methoxyphenyl)-2,2-difluoroethan-1-one (744 mg, 4 mmol, 1.0 equiv.) was dissolved in distilled THF (10 mL) at 0°C under argon atmosphere. $NaBH_4$ (190 mg, 5 mmol, 1.2 equiv.) was added over 5 minutes at 0 °C. The reaction mixture was stirred for 2 h at the same temperature, then HCl 1M (20 mL) and Et_2O (20 mL) were added, the organic layer was separated and the aqueous layer was extracted with Et_2O twice (20 mL). The combined organic layer was washed with brine (50 mL), dried over $MgSO_4$, filtered and concentrated under vacuum. The difluoroalkyl alcohol **1a-II** (720 mg, 96% yield) was obtained as a colorless liquid without further purification. The collected analytical data are in agreement with those reported in the literature.[26] ^{1}H NMR (400 MHz, $CDCl_3$) δ 7.33 (d, J = 8.5 Hz, 2H), 6.91 (d, J = 8.8 Hz, 2H), 5.72 (td, J = 56.1, 4.8 Hz, 1H), 4.75 (td, J = 9.9, 4.8 Hz, 1H), 3.80 (s, 3H), 2.38 (s, 1H).^{19}F NMR (376 MHz, $CDCl_3$) δ -127.60 (dd, J = 56.4, 7.5 Hz, 2F).

1-(4-Methoxyphenyl)-2,2-difluoroethan-1-one (744 mg, 4 mmol, 1.0 equiv.) was dissolved in MeOH (10 mL) at -40°C under argon atmosphere. iPrMgCl LiCl (4 mL, 5.2 mmol, 1.3 equiv. 1.3 M in THF) was added over 10 minutes at -40°C. The reaction mixture was stirred for 3 h at same temperature, then HCl 1M (20 mL) and Et_2O (20 mL) were added, the organic layer was separated and the aqueous layer was extracted with Et_2O twice (20 mL). The combined organic layer was washed with brine (50 mL), dried over $MgSO_4$, filtered and concentrated under vacuum. The residue was purified by flash chromatography (petroleum ether / ethyl acetate = 4:1) to afford **1a-III** (461mg, 50% yield) as a colorless liquid. ^{1}H NMR (400 MHz, $CDCl_3$) δ 7.37 (d, J = 8.6 Hz, 2H), 6.90 (d, J = 8.8 Hz, 2H), 5.97 (t, J = 56.2 Hz, 1H), 3.80 (s, 3H), 2.34 (dt, J = 13.6, 6.8 Hz, 1H), 2.21 (s, 1H), 1.02 (d, J = 6.7 Hz, 3H), 0.75 (d, J = 6.9 Hz, 3H). ^{19}F NMR (376 MHz, $CDCl_3$) δ -130.48 (m, 2F). ^{13}C NMR (101 MHz, $CDCl_3$) δ 159.0, 130.8, 127.2, 116.8 (t, *J* = 249.5 Hz), 113.5, 78.1 (t, *J* = 20.2 Hz), 55.1, 33.3 (d, *J* = 3.0 Hz), 16.6 (dd,

J = 50.5, 1.0 Hz). HRMS (ESI): m/z [(M+Na)$^+$] calcd for $C_{12}H_{16}F_2NaO_2$, 253.1011. found, 253.1016.

1-(4-Methoxyphenyl)-2,2-difluoroethan-1-one (744 mg, 4 mmol, 1.0 equiv.) was dissolved in MeOH (10 mL) at -40°C under argon atmosphere. MeMgBr (5 mL, mmol, 1.25 equiv. 1.0 M in THF) was added over 10 minutes at -40°C. The reaction mixture was stirred for 3 h at same temperature, then HCl 1M (20 mL) and Et_2O (20 mL) were added, the organic layer was separated and the aqueous layer was extracted with Et_2O twice (20 mL). The combined organic layer was washed with brine (50 mL), dried over $MgSO_4$, filtered and concentrated under vacuum. The residue was purified by flash chromatography (petroleum ether / ethyl acetate = 4:1) to afford **1a-IV** (493mg, 61% yield) as a colorless liquid.^{1}H NMR (400 MHz, $CDCl_3$) δ 7.41 (d, *J* = 8.8 Hz, 2H), 6.90 (d, *J* = 8.9 Hz, 2H), 5.66 (t, J = 56.0 Hz, 1H), 3.80 (s, 3H), 1.62 (t, J = 1.5 Hz, 3H). ^{19}F NMR (376 MHz, $CDCl_3$) δ -129.76 (dd, J = 275.4, 56.6 Hz, 1F), -130.10 (dd, J = 275.4, 56.3 Hz, 1F). The collected analytical data are in agreement with those reported in the literature.[57]

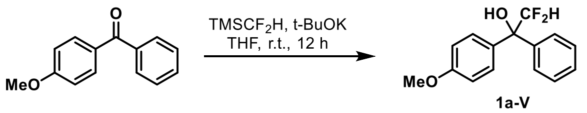

(4-Methoxyphenyl)(phenyl)methanone (10 mmol, 1.0 equiv.) was dissolved in distilled THF (100 mL) at 0°C under argon atmosphere. $TMSCF_2H$ (20 mmol, 2.0 equiv.) was added at 0°C followed by addition of a solution of *t*-BuOK (0.9 M in cyclohexane, 18 mmol, 1.8 equiv.) over 15 minutes. The reaction mixture was stirred for 12 h at room temperature then NH_4Cl (40 mL) and Et_2O (100 mL) were added. The organic layer was separated and the aqueous layer was extracted with Et_2O twice (60 mL). The combined organic layer was washed with brine (100 mL), dried over $MgSO_4$, filtered and concentrated under vacuum. The residue was purified by flash chromatography (petroleum ether / ethyl acetate = 4:1) to afford **1a-V** (2.0 g, 77% yield) as a white solid. ^{1}H NMR (400 MHz, $CDCl_3$) δ 7.44 (d, *J* = 7.6 Hz, 2H), 7.40-7.25 (m, 5H), 6.86 (t, *J* =

5.9 Hz, 2H), 6.16 (t, J = 55.3 Hz, 1H), 3.79 (s, 3H), 2.69 (s, 1H). ^{19}F NMR (376 MHz, $CDCl_3$) δ -127.50 (d, J = 52.6 Hz,2F). ^{13}C NMR (101 MHz, $CDCl_3$) δ 159.4, 140.7, 132.7, 128.5, 128.3, 128.2, 127.1, 117.0 (t, J = 251.5 Hz), 113.7, 77.9 (t, J = 19.7 Hz), 55.3. HRMS (ESI): m/z [$(M+Na)^+$] calcd for $C_{15}H_{14}F_2NaO_2$, 287.0854. found,

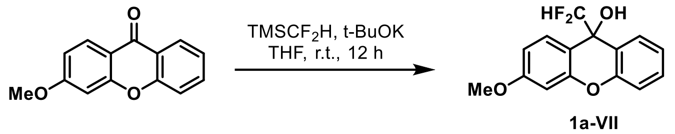

1a-VII

287.0857.

3-Methoxy-9H-xanthen-9-one (5 mmol, 1.0 equiv.) was dissolved in distilled THF (50 mL) at 0°C under argon atmosphere. $TMSCF_2H$ (10 mmol, 2.0 equiv.) was added at 0 °C followed by addition of a solution of *t*-BuOK (0.9 M in cyclohexane, 9 mmol, 1.8 equiv.) over 10 minutes. The reaction mixture was stirred for 12 h at room temperature then NH_4Cl (20 mL) and Et_2O (50 mL) were added. The organic layer was separated and the aqueous layer was extracted with Et_2O twice (30 mL). The combined organic layer was washed with brine (50 mL), dried over $MgSO_4$, filtered and concentrated under vacuum. The residue was purified by flash chromatography (petroleum ether / ethyl acetate = 5:1) to afford **1a-VII** (1.1 g, 82% yield) as a light-yellow solid.^{1}H NMR (400 MHz, $CDCl_3$) δ 7.65 (d, J = 7.8 Hz, 1H), 7.51 (d, J = 8.7 Hz, 1H), 7.42-7.33 (m, 1H), 7.22-7.15 (m, 1H), 7.14 (dd, J = 10.4, 2.2 Hz, 1H), 6.69 (dd, J = 8.7, 2.5 Hz, 1H), 6.61 (d, J = 2.5 Hz, 1H), 5.72 (t, J = 56.5 Hz, 1H), 3.75 (s, 3H), 3.10 (s, 1H). ^{19}F NMR (376 MHz, $CDCl_3$) δ -129.25 (m, 2F). ^{13}C NMR (101 MHz, $CDCl_3$) δ 161.1, 152.0, 151.1, 130.2, 128.4, 127.5, 123.4, 120.1, 116.4, 115.7 (t, J = 251.5 Hz), 112.0, 110.8, 100.9, 68.8 (t, J = 24.2 Hz), 55.4. HRMS (ESI): m/z [$(M+Na)^+$] calcd for $C_{15}H_{12}F_2NaO_3$, 301.0647. found, 301.0642.

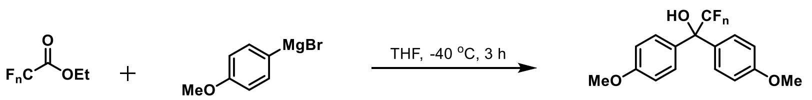

1

The corresponding fluoroalkyl ethyl esters (1.0 equiv.) was dissolved in THF (1 mmol / 4 mL) at -40 °C under argon atmosphere. A solution of 4-OMe-PhMgBr (2.4 equiv. 1 M in THF) was added over 10 minutes at -40 °C. The reaction mixture was

stirred for 3 h at the same temperature, then HCl 1M (1 mmol / 2 mL) and Et_2O (1 mmol / 2 mL) were added, the organic layer was separated and the aqueous layer was extracted with Et_2O twice (1 mmol / 2 mL). The combined organic layer was washed with brine (1 mmol / 2 mL), dried over $MgSO_4$, filtered and concentrated under vacuum. The residue was purified by flash chromatography to afford the fluorinated alcohol **1**.

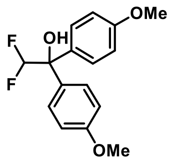

2,2-Difluoro-1,1-bis(4-methoxyphenyl)ethan-1-ol (1a-VI): The product was purified with silica gel chromatography (petroleum ether / ethyl acetate = 10:1) as light-yellow oil (15.2 g, 85% yield). ^1H NMR (400 MHz, $CDCl_3$) δ 7.35 (d, J = 8.7 Hz, 4H), 6.87 (t, J = 6.0 Hz, 4H), 6.11 (t, J = 55.4 Hz, 1H), 3.79 (s, 6H), 2.65 (s, 1H). ^{19}F NMR (376 MHz, $CDCl_3$) δ -127.25 (d, *J* = 52.7 Hz,2F). ^{13}C NMR (101 MHz, $CDCl_3$) δ 159.3, 132.9, 128.5, 117.1 (t, *J* = 251.5 Hz), 113.6, 77.6 (t, *J* = 21.2 Hz), 55.3. HRMS (ESI): m/z [$(M+Na)^+$] calcd for $C_{16}H_{16}F_2NaO_3$, 317.0960. found, 317.0958.

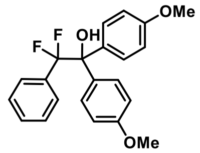

2,2-Difluoro-1,1-bis(4-methoxyphenyl)-2-phenylethan-1-ol (1b): The product was purified with silica gel chromatography (petroleum ether / ethyl acetate = 15:1) as white solid (3.4 g, 91% yield). ^1H NMR (400 MHz, $CDCl_3$) δ 7.34 (dd, J = 15.2, 8.1 Hz, 5H), 7.21 (t, J = 7.7 Hz, 2H), 7.10 (d, J = 7.6 Hz, 2H), 6.83-6.76 (m, 4H), 3.77 (s, 6H), 2.89 (s, 1H). ^{19}F NMR (376 MHz, $CDCl_3$) δ -101.31 (s, 2F). ^{13}C NMR (101 MHz, $CDCl_3$) δ 158.9, 134.45 (t, *J* = 26.3 Hz), 134.0, 129.5, 129.4 (t, *J* = 2.0 Hz), 127.5 (t, *J* = 6.6 Hz), 127.0, 123.4 (t, *J* = 257.0 Hz), 112.9, 80.5 (t, *J* = 29.3 Hz), 55.2. HRMS (ESI): m/z [$(M+Na)^+$] calcd for $C_{22}H_{20}F_2NaO_3$, 393.1273. found, 393.1270.

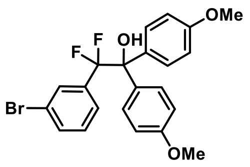

2-(3-Bromophenyl)-2,2-difluoro-1,1-bis(4-methoxyphenyl)ethan-1-ol (1c): The product was purified with silica gel chromatography (petroleum ether / ethyl acetate = 15:1) as white solid (4.0 g, 90% yield). ^{1}H NMR (400 MHz, $CDCl_3$) δ 7.43 (d, J = 8.0 Hz, 1H), 7.30 (d, J = 8.6 Hz, 4H), 7.22 (s, 1H), 7.04 (t, J = 7.9 Hz, 1H), 6.92 (d, J = 7.8 Hz, 1H), 6.78 (d, J = 8.9 Hz, 4H), 3.78 (s, 6H), 2.68 (s, 1H). ^{19}F NMR (376 MHz, $CDCl_3$) δ -106.99 (s, 2F). ^{13}C NMR (101 MHz, $CDCl_3$) δ 159.0, 136.5(t, *J* = 26.8 Hz), 133.6, 132.5, 130.7(t, *J* = 7.1 Hz), 129.3, 128.5, 126.1(t, *J* = 6.6 Hz), 122.6(t, *J* = 258.1 Hz), 121.0, 113.0, 80.3(t, *J* = 28.8 Hz), 55.1. HRMS (ESI): m/z [$(M+Na)^+$] calcd for $C_{22}H_{19}{}^{79}BrF_2NaO_3$, 471.0378. found, 471.0383.

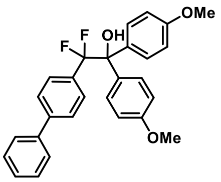

2-([1,1'-Biphenyl]-4-yl)-2,2-difluoro-1,1-bis(4-methoxyphenyl)ethan-1-ol (1d): The product was purified with silica gel chromatography (petroleum ether / ethyl acetate = 15:1) as white solid (3.8 g, 85% yield). ^{1}H NMR (400 MHz, $CDCl_3$) δ 7.58 (d, J = 7.4 Hz, 2H), 7.43 (m, 9H), 7.17 (d, J = 8.3 Hz, 2H), 6.81 (d, J = 8.9 Hz, 4H), 3.78 (s, 6H), 2.94 (s, 1H). ^{19}F NMR (376 MHz, $CDCl_3$) δ -100.96 (s, 2F). ^{13}C NMR (101 MHz, $CDCl_3$) δ 159.9, 142.5, 140.3, 134.1, 133.5 (t, *J* = 26.3 Hz), 129.5, 128.9, 128.0 (t, *J* = 6.1 Hz), 127.8, 127.2, 125.8 123.6 (t, *J* = 256.5 Hz), 113.1, 80.6 (t, *J* = 29.3 Hz), 55.2. HRMS (ESI): m/z [$(M+Na)^+$] calcd for $C_{28}H_{24}F_2NaO_3$, 469.1586. found, 469.1588.

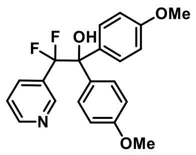

2,2-Difluoro-1,1-bis(4-methoxyphenyl)-2-(pyridin-3-yl)ethan-1-ol (1e): The product was purified with silica gel chromatography (petroleum ether / ethyl acetate = 4:1) as light red solid (1.8 g, 81% yield). ^{1}H NMR (400 MHz, pyridine-d5) δ 9.18 (s, 1H), 8.91 (d, J = 1.8 Hz, 1H), 8.70 (d, J = 4.8 Hz, 1H), 7.79 (d, J = 8.7 Hz, 4H), 7.71 (d, J = 8.0 Hz, 1H), 7.20-7.15 (m, 1H), 6.96 (d, J = 8.9 Hz, 4H), 3.64 (s, 6H). ^{19}F NMR (376 MHz, pyridine-d5) δ -100.93 (s, 2F). ^{13}C NMR (101 MHz, pyridine-d5) δ 159.2, 150.6, 149.0 (t, *J* =7.1 Hz), 135.4, 135.3, 131.6 (t, *J* = 26.8 Hz), 129.9, 124.0 (t, *J* = 257.6 Hz), 122.1, 113.2, 80.0 (t, *J* = 27.3 Hz), 54.9. HRMS (ESI): m/z [(M+H)$^+$] calcd for $C_{21}H_{20}F_2NO_3$, 372.1406. found, 372.1408.

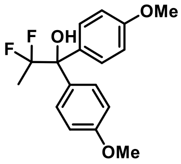

2,2-Difluoro-1,1-bis(4-methoxyphenyl)propan-1-ol (1f): The product was purified with silica gel chromatography (petroleum ether / ethyl acetate = 15:1) as white solid (1.0 g, 82% yield). ^{1}H NMR (400 MHz, $CDCl_3$) δ 7.44 (d, J = 8.9 Hz, 4H), 6.95-6.73 (m, 4H), 3.77 (s, 6H), 2.75 (s, 1H), 1.55 (t, J = 19.4 Hz, 3H). ^{19}F NMR (376 MHz, $CDCl_3$) δ -98.22 (q, J = 18.8 Hz, 2F). ^{13}C NMR (101 MHz, $CDCl_3$) δ 158.9, 134.2, 128.9, 125.9 (t, *J* = 251.0 Hz), 113.2, 79.5 (t, *J* = 26.3 Hz), 55.2, 20.3 (t, *J* = 26.8 Hz). HRMS (ESI): m/z [(M+Na)$^+$] calcd for $C_{17}H_{18}F_2NaO_3$, 331.1116. found, 331.1116.

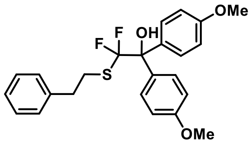

2,2-Difluoro-1,1-bis(4-methoxyphenyl)-2-(phenethylthio)ethan-1-ol (1k): The product was purified with silica gel chromatography (petroleum ether / ethyl acetate =

15:1) as colorless oil (1.8 g, 69% yield). ^{1}H NMR (400 MHz, $CDCl_3$) δ 7.48 (d, J = 8.7 Hz, 4H), 7.28 (t, J = 7.2 Hz, 2H), 7.24-7.10 (m, 3H), 6.85 (d, J = 8.9 Hz, 4H), 3.79 (s, 6H), 3.08-3.03 (m, 2H), 2.99-2.90 (m, 3H). ^{19}F NMR (376 MHz, $CDCl_3$) δ -78.84 (s, 2F). ^{13}C NMR (101 MHz, $CDCl_3$) δ 159.3, 139.8, 132.8 (t, *J* = 291.4 Hz), 132.5, 129.2, 128.5, 128.4, 126.5, 113.2, 81.1 (t, *J* = 24.2 Hz), 55.2, 36.6, 29.9. HRMS (ESI): m/z [$(M+Na)^+$] calcd for $C_{24}H_{24}F_2NaO_3S$, 453.1306. found, 453.1309.

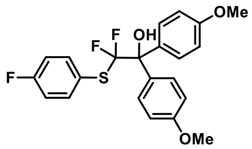

2,2-Difluoro-2-((4-fluorophenyl)thio)-1,1-bis(4-methoxyphenyl)ethan-1-ol (1l): The product was purified with silica gel chromatography (petroleum ether / ethyl acetate = 15:1) as white solid (1.5 g, 73% yield). ^{1}H NMR (400 MHz, $CDCl_3$) δ 7.55-7.46 (m, 6H), 7.02 (t, J = 8.7 Hz, 2H), 6.85 (d, J = 9.0 Hz, 4H), 3.78 (s, 6H), 3.08 (s, 1H). ^{19}F NMR (376 MHz, $CDCl_3$) δ -77.86 (s, 2F), -110.75 (m, 1F). ^{13}C NMR (101 MHz, $CDCl_3$) δ 164.0 (d, *J* = 251.5 Hz), 159.4, 138.8 (d, *J* = 9.1 Hz), 132.6, 131.3 (t, *J* = 293.4 Hz), 129.2, 121.8 (d, *J* = 2.0 Hz), 116.1 (d, *J* = 22.2 Hz), 114.9, 113.3, 81.2 (t, *J* = 24.2 Hz), 55.2. HRMS (ESI): m/z [$(M+Na)^+$] calcd for $C_{22}H_{19}F_3NaO_3S$, 443.0899. found, 443.0900.

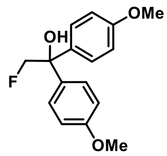

2-Fluoro-1,1-bis(4-methoxyphenyl)ethan-1-ol (1m): The product was purified with silica gel chromatography (petroleum ether / ethyl acetate = 20:1) as white solid (730.2 mg, 53% yield). ^{1}H NMR (400 MHz, $CDCl_3$) δ 7.31 (d, J = 8.8 Hz, 4H), 6.86 (d, J = 8.8 Hz, 4H), 4.78 (d, J = 47.9 Hz, 2H), 3.78 (s, 6H), 2.93 (s, 1H). ^{19}F NMR (376 MHz, $CDCl_3$) δ -217.16 (t, J = 47.0 Hz, 1F). ^{13}C NMR (101 MHz, $CDCl_3$) δ 159.0, 134.9 (d, *J* = 2.0 Hz), 128.1, 113.7, 87.6 (d, *J* = 181.8 Hz), 77.4 (d, *J* = 18.2 Hz), 55.3. HRMS (ESI): m/z [$(M+Na)^+$] calcd for $C_{16}H_{17}FNaO_3$, 299.1054. found, 229.1057.

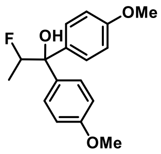

2-Fluoro-1,1-bis(4-methoxyphenyl)propan-1-ol (1n): The product was purified with silica gel chromatography (petroleum ether / ethyl acetate = 20:1) as white solid (1.1 g, 60% yield). ^{1}H NMR (400 MHz, $CDCl_3$) δ 7.43 (d, J = 8.8 Hz, 2H), 7.26 (d, J = 8.9 Hz, 2H), 6.83 (dd, J = 18.5, 8.9 Hz, 4H), 5.48 (dq, J = 46.6, 6.2 Hz, 1H), 3.78 (s, 3H), 3.76 (s, 3H), 2.51 (d, J = 2.0 Hz, 1H), 1.23 (dd, J = 24.5, 6.2 Hz, 3H). ^{19}F NMR (376 MHz, $CDCl_3$) δ -179.16 (m, 1F). ^{13}C NMR (101 MHz, $CDCl_3$) δ 158.7, 158.6, 137.0, 135.7 (d, *J* = 4.0 Hz), 128.2, 128.2, 127.2, 113.5, 113.5, 92.79 (d, *J* = 175.7 Hz), 78.6 (d, *J* = 11.1 Hz), 55.2, 55.2, 15.1(d, *J* = 23.2 Hz). HRMS (ESI): m/z [(M+Na)$^+$] calcd for $C_{17}H_{19}FNaO_3$, 313.1210. found, 313.1214.

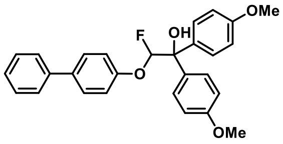

2-([1,1'-Biphenyl]-4-yloxy)-2-fluoro-1,1-bis(4-methoxyphenyl)ethan-1-ol (1q): The product was purified with silica gel chromatography (petroleum ether / ethyl acetate = 20:1) as white solid (364.1 mg, 41% yield). ^{1}H NMR (400 MHz, $CDCl_3$) δ 7.55-7.39 (m, 10H), 7.32 (t, J = 7.3 Hz, 1H), 7.09 (d, J = 8.6 Hz, 2H), 6.88 (dd, J = 9.0, 2.7 Hz, 4H), 6.11 (d, J = 60.7 Hz, 1H), 3.80 (s, 6H), 3.00 (s, 1H). ^{19}F NMR (376 MHz, $CDCl_3$) δ -131.84 (d, J = 60.2 Hz, 1F). ^{13}C NMR (101 MHz, $CDCl_3$) δ 159.1, 156.0, 156.0, 140.4, 137.1, 134.5, 134.11, 128.8, 128.8, 128.4, 127.1, 126.9, 117.7, 113.5, 113.4, 112.5 (d, *J* = 232.3 Hz), 78.4 (d, *J* = 23.2 Hz), 55.3. HRMS (ESI): m/z [(M+Na)$^+$] calcd for $C_{28}H_{25}FNaO_4$, 467.1629. found, 467.1633.

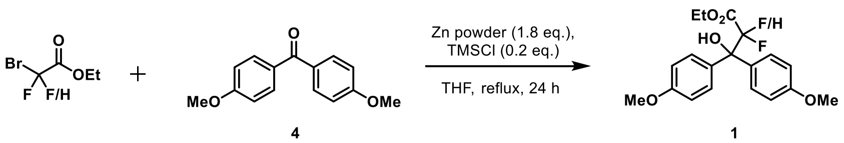

In a 250 mL 3-neck flask containing a stir bar, zinc powder (90 mmol, 1.8 equiv.) was

suspended in dry THF (100 mL) under Ar atmosphere. Then TMSCl (18 mmol, 0.2 equiv.) was added to the suspension, and the mixture was heated to reflux to active the zinc dust. To this vigorous stirred mixture was added dropwise a solution of bis(4-methoxyphenyl)methanone (50 mmol) and ethyl 2- bromofluoroalkylacetate (70 mmol) in dry THF (50 mL) . The resulted reaction mixture was stirred at reflux until the starting materials were consumed by TLC analysis. Then the mixture was cooled to 0 ºC, acidified with HCl (50 mL, 2 M) and exacted with ethyl acetate (3×80 mL). The combined organic layer was washed with sat. $NaHCO_3$ solution and brine, dried over anhydrous $MgSO_4$ and concentrated under vacuum. The residue was purified by flash chromatography to afford fluorinated alcohol **1i** and **1o**.

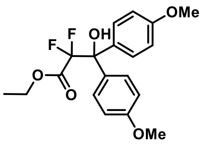

Ethyl 2,2-difluoro-3-hydroxy-3,3-bis(4-methoxyphenyl)propanoate (1i): The product was purified with silica gel chromatography (petroleum ether / ethyl acetate = 10:1) as colorless oil (13.0 g, 71% yield). ^1H NMR (400 MHz, $CDCl_3$) δ 7.42 (d, J = 8.9 Hz, 4H), 6.84 (d, J = 9.0 Hz, 4H), 4.19 (q, J = 7.2 Hz, 2H), 3.80 (s, 1H), 3.78 (s, 6H), 1.15 (t, J = 7.1 Hz, 3H). ^{19}F NMR (376 MHz, $CDCl_3$) δ -109.78 (s, 2F). ^{13}C NMR (101 MHz, $CDCl_3$) 164.1 (t, *J* = 32.3 Hz), 159.4, 132.0, 128.7, 114.4 (t, *J* = 264.1 Hz), 113.3, 79.1(t, *J* = 23.2 Hz), 63.2, 55.2, 55.2, 13.6. HRMS (ESI): m/z [$(M+Na)^+$] calcd for $C_{19}H_{20}F_2NaO_5$, 389.1171. found, 389.1173.

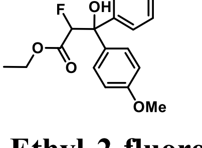

Ethyl 2-fluoro-3-hydroxy-3,3-bis(4-methoxyphenyl)propanoate (1o): The product was purified with silica gel chromatography (petroleum ether / ethyl acetate = 10:1) as colorless oil (11 g, 63% yield). ^1H NMR (400 MHz, $CDCl_3$) δ 7.45 (d, J = 8.8 Hz, 2H), 7.28 (d, J = 8.8 Hz, 2H), 6.91-6.76 (m, 4H), 5.37 (d, J = 46.9 Hz, 1H), 4.09 (m, 2H),

3.99 (d, J = 1.6 Hz, 1H), 3.78 (s, 3H), 3.76 (s, 3H), 1.06 (t, J = 7.1 Hz, 3H). ^{19}F NMR (376 MHz, $CDCl_3$) δ -188.30 (d, J = 45.1 Hz, 1F). ^{13}C NMR (101 MHz, $CDCl_3$) δ 168.9 (d, *J* = 24.2 Hz), 159.1, 134.8, 134.8, 134.2, 134.2, 128.2, 127.9, 127.8, 113.6, 113.4, 92.1 (d, *J* = 200.0 Hz), 78.0 (d, *J* = 20.2 Hz), 61.9, 55.24 13.8. HRMS (ESI): m/z [$(M+Na)^+$] calcd for $C_{19}H_{21}FNaO_5$, 371.1265. found, 371.1264.

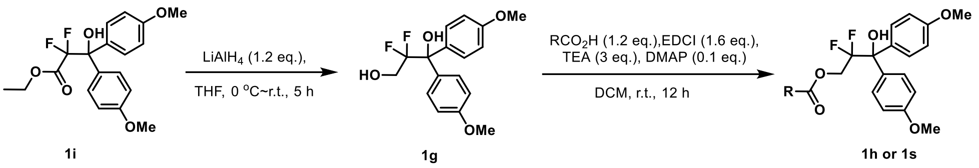

To a suspension of $LiAlH_4$ (1.1 g, 30 mmol, 1.2 equiv) in dry THF (50 mL) was added a solution of **1i** (9.1 g, 25 mmol, 1.0 equiv) in THF (25 mL) at 0 °C, and the mixture was stirred at room temperature. After complete consumption of the starting material, the mixture was cooled to 0 °C, quenched carefully with wet $MgSO_4$ and water and then exacted with ethyl acetate (3 × 40 mL). The combined organic layer was washed with brine, dried over anhydrous $MgSO_4$ and concentrated under vacuum. The difluoroalkyl alcohol **1g** (8.0 g, 99% yield) was obtained as a white solid without further purification. ^{1}H NMR (400 MHz, $CDCl_3$) δ 7.43 (d, J = 8.7 Hz, 4H), 6.83 (d, J = 8.9 Hz, 4H), 3.81-3.73 (m, 8H), 2.82 (s, 2H). ^{19}F NMR (376 MHz, $CDCl_3$) δ -114.42 (t, J = 13.2 Hz, 2F). ^{13}C NMR (101 MHz, $CDCl_3$) δ 159.1, 133.4, 128.6, 121.8 (t, *J* = 256.0 Hz), 113.5, 79.6 (t, *J* = 25.3 Hz), 62.9 (t, *J* = 28.8 Hz), 55.2. HRMS (ESI): m/z [$(M+Na)^+$] calcd for $C_{17}H_{18}F_2NaO_4$, 347.1065. found, 347.1071.

To a 100 mL of round-bottom flask were added an appropriate carboxylic acid (12 mmol, 1.2 equiv) in DCM (50 mL), then added **1g** (obtained above, 3.2 g, 10 mmol, 1.0 equiv), EDCI (2.9 g, 15 mmol, 1.5 equiv), TEA (4.2 mL, 30 mmol, 3.0 equiv) and DMAP (122 mg, 1 mmol, 0.1 equiv) at 0 °C. The reaction mixture was stirred at room temperature for 12 hours, then concentrated in vacuo. The crude product was purified by silica gel column chromatography to afford product **1h** or **1r**.

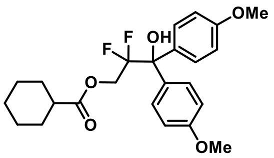

2,2-Difluoro-3-hydroxy-3,3-bis(4-methoxyphenyl)propyl cyclohexanecarboxylate (1h): The product was purified with silica gel chromatography (petroleum ether / ethyl acetate = 15:1) as white solid (3.4 g, 78% yield). ^{1}H NMR (400 MHz, $CDCl_3$) δ 7.45 (d, J = 8.7 Hz, 4H), 6.85 (d, J = 8.8 Hz, 4H), 4.38 (t, J = 15.0 Hz, 2H), 3.79 (s, 6H), 2.96 (s, 1H), 2.26 (dd, J = 9.1, 5.5 Hz, 1H), 1.85 (d, J = 12.9 Hz, 2H), 1.77-1.68 (m, 2H), 1.62 (s, 1H), 1.39 (m, 2H), 1.24 (m, 3H). ^{19}F NMR (376 MHz, $CDCl_3$) δ -113.04 (t, J = 15.0 Hz, 2F). ^{13}C NMR (101 MHz, $CDCl_3$) δ 175.3, 159.2, 133.2, 128.7, 121.9 (t, *J* = 257.0 Hz), 113.5, 78.7 (t, *J* = 24.2 Hz), 61.7 (t, *J* = 24.7 Hz), 55.2, 42.9, 28.8, 25.7, 25.3. HRMS (ESI): m/z [$(M+Na)^+$] calcd for $C_{24}H_{28}F_2NaO_5$, 457.1797. found, 457.1802.

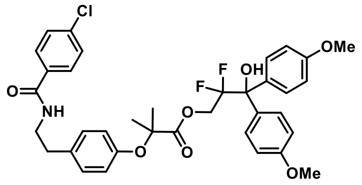

2,2-Difluoro-3-hydroxy-3,3-bis(4-methoxyphenyl)propyl 2-(4-(2-(4-chloro benzamido)ethyl)phenoxy)-2-methylpropanoate (1r): The product was purified with silica gel chromatography (petroleum ether / ethyl acetate = 2:1) as white solid (4.2 g, 63% yield). ^{1}H NMR (400 MHz, $CDCl_3$) δ 7.54 (d, J = 8.4 Hz, 2H), 7.40 (d, J = 8.6 Hz, 4H), 7.30 (d, J = 8.4 Hz, 2H), 7.02 (d, J = 8.3 Hz, 2H), 6.78 (t, J = 9.2 Hz, 6H), 6.24 (t, J = 5.4 Hz, 1H), 4.46 (t, J = 14.9 Hz, 2H), 3.74 (s, 6H), 3.56 (q, J = 6.6 Hz, 2H), 3.39 (s, 1H), 2.78 (t, J = 6.8 Hz, 2H), 1.53 (s, 6H). ^{19}F NMR (376 MHz, $CDCl_3$) δ -112.95 (t, J = 15.0 Hz, 2F). ^{13}C NMR (101 MHz, $CDCl_3$) δ 173.5, 166.4, 159.2, 153.9, 137.5, 133.0, 132.9, 132.7, 129.5, 128.7, 128.7, 128.3, 121.8 (t, *J* = 257.6 Hz), 119.9, 113.5, 79.3, 78.7 (t, *J* = 24.2 Hz), 62.7 (t, *J* = 24.7 Hz), 55.2, 41.3, 34.7, 25.4. HRMS (ESI): m/z [$(M+Na)^+$] calcd for $C_{36}H_{36}{}^{35}ClF_2NNaO_7$, 690.2041. found, 690.2043.

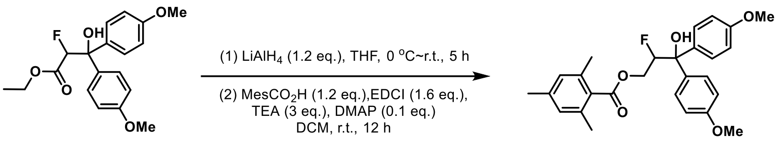

1o **1p**

To a suspension of $LiAlH_4$ (0.7 g, 18 mmol) in dry THF (30 mL) was added a solution of **1o** (5.2 g, 15 mmol) in THF (10 mL) at 0 °C, and the mixture was stirred at room temperature. After complete consumption of the starting material, the mixture was cooled to 0 °C, quenched carefully with wet $MgSO_4$ and water and then exacted with ethyl acetate (3×20 mL). The combined organic layer was washed with brine, dried over anhydrous $MgSO_4$ and concentrated under vacuum. The expected alcohol was used without further purification.

To a 50 mL of round-bottom flask were added 2,4,6-trimethylbenzoic acid (1.0 g, 6 mmol, 1.2 equiv) in DCM (15 mL), then added the alcohol obtained above (1.5 g, 5 mmol, 1.0 equiv), EDCI (1.4 g, 7.5 mmol, 1.5 equiv), TEA (2.1 mL, 15 mmol, 3.0 equiv) and DMAP (61 mg, 0.5 mmol, 0.1 equiv) at 0 °C. The reaction mixture was stirred at room temperature for 12 hours, then concentrated in vacuo. The crude product was purified by silica gel column chromatography (petroleum ether / ethyl acetate = 15:1) to afford the product **1p** as white solid (1.7 g, 75% yield). ^1H NMR (400 MHz, $CDCl_3$) δ 7.41 (d, J = 8.7 Hz, 2H), 7.32 (d, J = 8.8 Hz, 2H), 6.85 (dd, J = 8.6, 7.3 Hz, 6H), 5.60 (dd, J = 48.9, 8.7 Hz, 1H), 4.60 (m, 1H), 4.28 (dd, J = 35.6, 12.6 Hz, 1H), 3.77 (d, J = 5.0 Hz, 6H), 2.81 (s, 1H), 2.27 (s, 9H). ^{19}F NMR (376 MHz, $CDCl_3$) δ -191.90 (m, 1F). ^{13}C NMR (101 MHz, $CDCl_3$) δ 169.8, 158.9, 158.9, 139.4, 135.8, 135.4, 134.6, 134.6, 130.4, 128.4, 127.8, 127.8, 127.1, 93.5 (d, *J* = 184.8 Hz), 77.5 (d, *J* = 20.2 Hz), 63.9 (d, *J* = 21.2 Hz), 55.2, 55.2, 21.1, 19.8. HRMS (ESI): m/z [$(M+Na)^+$] calcd for $C_{27}H_{29}FNaO_5$, 475.1891. found, 475.1893.

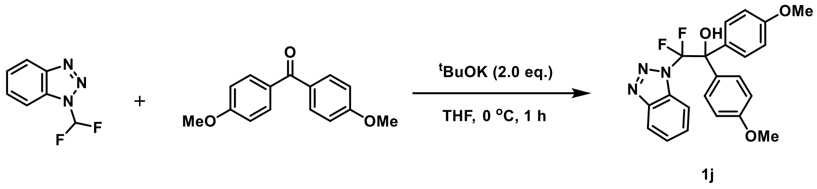

1j

To an oven dried 2-necked 250 mL round bottom flask containing a stirring bar was added 1-(difluoromethyl)-1H-benzo[d][1,2,3]triazole (3.4 g, 20 mmol, 1.0 equiv) and bis(4-methoxyphenyl)methanone (5.8 g, 24 mmol, 1.2 equiv) under Ar atmosphere. To the reaction flask was added anhydrous THF (40 mL) via a syringe at 0 ^{o}C. A solution of KO^{t}Bu (1.0 M in THF, 40 mL) was slowly dropped at 0 ^{o}C. After stirring under Ar at 0 C for 1 h, the reaction was quenched by ice-cold water (150 mL). The mixture was extracted with EtOAc (3 x 100 mL). The combined organic layer was dried over MgSO4, then filtered and concentrated in vacuo. The crude product was purified by silica gel column chromatography (petroleum ether / ethyl acetate = 4:1) to afford product **1j** as white solid (4.0 g, 49% yield). Known compound.[2] ^{1}H NMR (400 MHz, $CDCl_3$) δ 8.06 (d, J = 8.4 Hz, 1H), 7.66 (d, J = 8.5 Hz, 1H), 7.54 (t, J = 7.6 Hz, 1H), 7.43 (t, J = 7.7 Hz, 1H), 7.37 (d, J = 8.7 Hz, 4H), 6.78 (d, J = 8.9 Hz, 4H), 4.81 (s, 1H), 3.75 (s, 6H). ^{19}F NMR (376 MHz, $CDCl_3$) δ -85.20 (s, 2F).

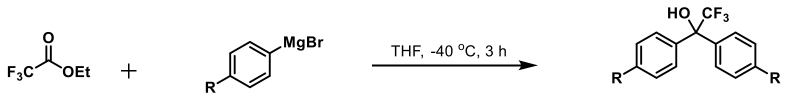

The ethyl trifluoroacetate (1.42 g, 10 mmol, 1.0 equiv.) was dissolved in 40 mL dry THF at -40 °C under argon atmosphere. An appropriate Grignard reagent (24 mmol, 2.4 equiv.) was added over 10 minutes at -40 °C. The reaction mixture was stirred for 3 h at the same temperature, then HCl 1M (20 mL) and Et_2O (20 mL) were added, The organic layer was separated and the aqueous layer was extracted with Et_2O twice (20 mL). The combined organic layer was washed with brine (20 mL), dried over $MgSO_4$, filtered and concentrated under vacuum. The residue was purified by flash chromatography to afford fluorinated alcohol **1**.

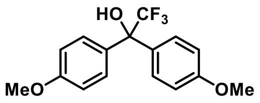

2,2,2-Trifluoro-1,1-bis(4-methoxyphenyl)ethan-1-ol (1ba-I): The product was purified with silica gel chromatography (petroleum ether / ethyl acetate = 10:1) as white solid (2.74 g, 88% yield). Known compound.[28] ^{1}H NMR (400 MHz, $CDCl_3$) δ 7.38 (d, J = 8.6 Hz, 4H), 6.91-6.81 (m, 4H), 3.79 (d, J = 6.1 Hz, 6H), 2.94 (s, 1H).^{19}F NMR (376 MHz, $CDCl_3$) δ -74.61 (s, 3F).

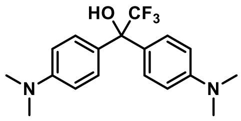

1,1-Bis(4-(dimethylamino)phenyl)-2,2,2-trifluoroethan-1-ol (1ba-II): The product was purified with silica gel chromatography (petroleum ether / ethyl acetate = 5:1) as white solid (2.33 g, 69% yield). ^{1}H NMR (400 MHz, $CDCl_3$) δ 7.32 (d, J = 8.6 Hz, 4H), 6.66 (d, J = 9.0 Hz, 4H), 2.94 (s, 12H), 2.72 (s, 1H). ^{19}F NMR (376 MHz, $CDCl_3$) δ -74.66 (s, 3F). ^{13}C NMR (101 MHz, $CDCl_3$) δ 150.2, 128.4, 127.4, 125.8 (q, J = 287.5 Hz), 111.6, 79.1 (q, J = 28.6 Hz), 40.4. HRMS (ESI): m/z [$(M+H)^+$] calcd for $C_{18}H_{22}F_3N_2O$, 339.1679. found, 339.1670.

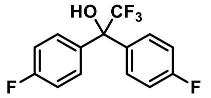

2,2,2-Trifluoro-1,1-bis(4-fluorophenyl)ethan-1-ol (1ba-III): The product was purified with silica gel chromatography (petroleum ether / ethyl acetate = 12:1) as colorless oil (2.13 g, 74% yield). Known compound.[27] ^{1}H NMR (400 MHz, $CDCl_3$) δ 7.45 (dd, J = 8.4, 5.4 Hz, 4H), 7.03 (t, J = 8.7 Hz, 4H), 3.29 (s, 1H). ^{19}F NMR (376 MHz, $CDCl_3$) δ -74.71 (s, 3F), -112.86 (m, 2F).

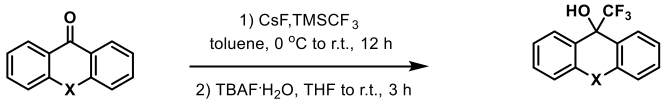

1

To an oven dried 50 mL round bottom flask containing a stirring bar was added an appropriate aryl ketone (10 mmol, 1.0 equiv) and CsF (0.3 g, 2 mmol, 0.2 equiv) under Ar atmosphere. To the reaction flask was added anhydrous toluene (20 mL) via a syringe at 0 ^{o}C. $TMSCF_3$ (3.0 mL, 20 mmol, 2.0 equiv) was slowly dropped to the solution at 0 ^{o}C. The cooling bath was removed and the reaction mixture was allowed to stir at room temperature overnight. The reaction was filtered and concentrated under vacuum. A solution of $TBAF{\cdot}H_2O$ (24 mL, 0.5 M in THF, 1.2 equiv) was added to the reaction flask. After stirring at room temperature for 3 h, the reaction was quenched with HCl (50 mL, 0.5 M). The mixture was extracted with EtOAc (3 x 40 mL). The combined organic layer was dried over $MgSO_4$, then filtered and concentrated in vacuo. The residue was purified by flash chromatography to afford fluorinated alcohol **1**.[44]

9-(Trifluoromethyl)-9H-fluoren-9-ol (1ba-IV): The product was purified with silica gel chromatography (petroleum ether / ethyl acetate = 12:1) as white solid (2.13 g, 85%

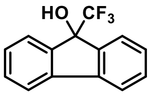

yield). Known compound.[28] ^{1}H NMR (400 MHz, $CDCl_3$) δ 7.61 (dd, *J* = 14.0, 7.6 Hz, 4H), 7.42 (td, *J* = 7.5, 0.7 Hz, 2H), 7.29 (td, *J* = 7.5, 0.6 Hz, 2H), 2.81 (s, 1H). ^{19}F NMR (376 MHz, $CDCl_3$) δ -78.51 (s, 3F).

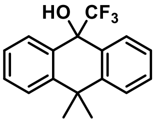

10,10-Dimethyl-9-(trifluoromethyl)-9,10-dihydroanthracen-9-ol (1ba-V): The product was purified with silica gel chromatography (petroleum ether / ethyl acetate = 15:1) as white solid (2.22 g, 76% yield). ^{1}H NMR (400 MHz, $CDCl_3$) δ 7.92 (d, J = 7.9 Hz, 2H), 7.63 (d, J = 8.0 Hz, 2H), 7.48 (t, J = 7.3 Hz, 2H), 7.35 (t, J = 7.5 Hz, 2H), 2.84 (s, 1H), 1.75 (s, 3H), 1.65 (s, 3H). ^{19}F NMR (376 MHz, $CDCl_3$) δ -78.73 (s, 3F). ^{13}C NMR (101 MHz, $CDCl_3$) δ 144.8, 130.9, 129.7, 127.9, 127.8, 126.6, 126.4, 124.4 (q, J

= 287.5 Hz), 72.5 (q, J = 29.3 Hz), 38.2, 34.2, 33.1. HRMS (ESI): m/z [$(M\text{-}OH)^+$] calcd for $C_{17}H_{14}F_3$, 275.1048. found, 275.1053.

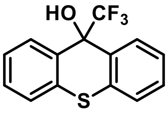

9-(Trifluoromethyl)-9H-thioxanthen-9-ol (1ba-VI): The product was purified with silica gel chromatography (petroleum ether / ethyl acetate = 10:1) as white solid (1.97 g, 70% yield). Known compound.[28] ^{1}H NMR (400 MHz, $CDCl_3$) δ 7.94 (d, J = 7.1 Hz, 2H), 7.39-7.28 (m, 6H), 2.90 (s, 1H). ^{19}F NMR (376 MHz, $CDCl_3$) δ -80.60 (s, 3F).

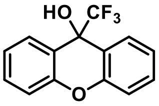

9-(Trifluoromethyl)-9H-xanthen-9-ol (1ba-VII): The product was purified with silica gel chromatography (petroleum ether / ethyl acetate = 10:1) as white solid (2.42 g, 91% yield). Known compound.[45] ^{1}H NMR (400 MHz, $CDCl_3$) δ 7.78 (d, *J* = 7.7 Hz, 2H), 7.42 (t, *J* = 7.7 Hz, 2H), 7.20 (dd, *J* = 11.5, 8.2 Hz, 4H), 2.96 (s, 1H). ^{19}F NMR (376 MHz, $CDCl_3$) δ -81.40 (s, 3F).

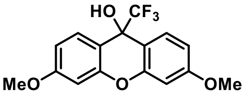

3,6-Dimethoxy-9-(trifluoromethyl)-9H-xanthen-9-ol (1ba-IX): The product was purified with silica gel chromatography (petroleum ether / ethyl acetate = 10:1) as white solid (0.43 g, 13% yield). ^{1}H NMR (400 MHz, $CDCl_3$) δ 7.66 (d, J = 8.7 Hz, 2H), 6.74 (dd, J = 8.8, 2.5 Hz, 2H), 6.64 (d, J = 2.5 Hz, 2H), 3.81 (s, 6H), 3.04 (s, 1H). ^{19}F NMR (376 MHz, $CDCl_3$) δ -81.42 (s, 3F). ^{13}C NMR (101 MHz, $CDCl_3$) δ 161.4, 152.2, 128.8, 124.4 (q, J = 286.8 Hz), 111.4, 110.8, 100.9, 69.2 (q, J = 31.3 Hz), 55.5. HRMS (ESI): m/z [$(M\text{-}OH)^+$] calcd for $C_{16}H_{12}F_3O_3$, 309.0739. found, 309.0741.

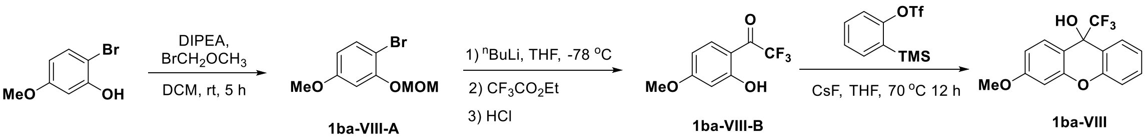

To a 500 mL round bottom flask containing a stirring bar was added 2-bromo-5-methoxyphenol (25.0 g, 124 mmol, 1.0 equiv), bromomethyl methyl ether (20.4 mL, 250 mmol, 2.0 equiv) and 200 mL of DCM. To the mixture was added DIPEA (47.5 mL, 273 mmol, 2.2 equiv) via a syringe at 0 ^{o}C. The cooling bath was removed and the reaction mixture was allowed to stir at room temperature for 5 h. The reaction was quenched with HCl (300 mL, 0.5 M), then, the organic layer was washed with HCl (2 x 300 mL, 0.5 M) and $NaHCO_3$ (200 mL, 1.0 M). The organic layer was dried over $MgSO_4$, then filtered and concentrated in vacuo to afford compound **1ba-VIII-A** as light-yellow oil (30.3 g, 99% yield), which was used for next step without further purification.

To an oven dried 2-necked 250 mL round bottom flask containing a stirring bar was added 1-bromo-4-methoxy-2-(methoxymethoxy)benzene (4.9 g, 20 mmol, 1.0 equiv) and 80 mL of anhydrous THF under Ar atmosphere. To the reaction flask was added ^{n}BuLi (8.7 mL, 21 mmol, 1.05 equiv, 2.4 M in hexane) via a syringe at -78 ^{o}C. After stirring under Ar at the same temperature for 1 h, CF_3CO_2Et (3.6 mL, 30 mmol, 1.5 equiv) was added via a syringe, and the reaction mixture was allowed to stir at same temperature for 3h. The reaction was treated with HCl (60 mL, 0.5 M) and then extracted with EtOAc (3 x 100 mL). The combined organic layer was dried over $MgSO_4$, then filtered and concentrated in vacuo. The residue was dissolved in HCl (50 mL, 3.0 M in EtOAc). After stirring under air at room temperature for 2 h, the mixture was concentrated in vacuo, and the residue was purified by flash chromatography (petroleum ether / ethyl acetate = 20:1) to afford compound **1ba-VIII-B** as white solid (2.9 g, 65% yield). Known compound.[46] ^{1}H NMR (400 MHz, $CDCl_3$) δ 11.58 (s, 1H), 7.71 (dd, J = 9.2, 1.8 Hz, 1H), 6.53 (dd, J = 9.5, 1.9 Hz, 1H), 6.49 (s, 1H), 3.89 (s, 3H). ^{19}F NMR (376 MHz, $CDCl_3$) δ -70.19 (s, 3F).

To an oven dried 2-necked 250 mL round bottom flask containing a stirring bar was added 2,2,2-trifluoro-1-(2-hydroxy-4-methoxyphenyl)ethan-1-one (2.9 g, 13 mmol, 1.0 equiv), CsF (5.9 g, 39 mmol, 3.0 equiv), 2-(trimethylsilyl)phenyl

trifluoromethanesulfonate (4.7 mL, 19.5 mmol, 1.5 equiv), and 130 mL anhydrous THF under Ar atmosphere. After stirring under Ar atmosphere at 70 ^oC for 12 h, the solvent was removed under reduced pressure and the residue was purified by flash chromatography (petroleum ether / ethyl acetate = 10:1) to afford product **1ab-VIII** as yellow solid (3.5 g, 91% yield).[45] ^1H NMR (400 MHz, $CDCl_3$) δ 7.80 (d, J = 7.8 Hz, 1H), 7.67 (d, J = 8.8 Hz, 1H), 7.42 (t, J = 7.7 Hz, 1H), 7.24-7.15 (m, 2H), 6.76 (dd, J = 8.8, 2.2 Hz, 1H), 6.67 (d, J = 2.1 Hz, 1H), 3.81 (s, 3H), 2.88 (s, 1H). ^{19}F NMR (376 MHz, $CDCl_3$) δ -81.43 (s, 3F). ^{13}C NMR (101 MHz, $CDCl_3$) δ 161.6, 152.1, 151.1, 130.7, 128.6, 127.8, 124.2 (q, J = 287.5 Hz), 123.5, 119.2, 116.5, 111.0, 110.9, 100.8, 69.4 (q, J = 31.6 Hz), 55.5. HRMS (ESI): m/z [$(M+Na)^+$] calcd for $C_{15}H_{11}F_3NaO_3$, 319.0552. found, 319.0564.

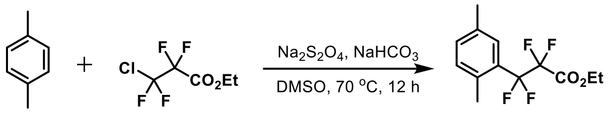

1bb-A

To a 250 mL round bottomed flask, equipped with a magnetic stirrer, $ClCF_2CF_2CO_2Et$ (10.0 g, 48 mmol, 1.0 equiv), p-Xylene (18.0 mL, 144 mmol, 3.0 equiv), $Na_2S_2O_4$ (12.9 g, 72 mmol, 1.5 equiv), $NaHCO_3$ (6.3 g, 72 mmol, 1.5 equiv) and 120 mL DMSO were added and the mixture was stirred at 70 ^oC. After 12 hours, the reaction was quenched with 600 mL of ice-cold water and the mixture was extracted with n-hexane (3 x 100 mL). The combined organic layer was dried over $MgSO_4$, then filtered and concentrated in vacuo. The residue was purified by flash chromatography (petroleum ether / ethyl acetate = 100:1) to afford the product **1bb-A** as colorless liquid (2.9 g, 22% yield). Known compound.[45] ^1H NMR (400 MHz, $CDCl_3$) δ 7.25 (s, 1H), 7.17 (d, J = 8.0 Hz, 1H), 7.11 (d, J = 7.8 Hz, 1H), 4.36 (q, J = 7.2 Hz, 2H), 2.39 (t, J = 3.0 Hz, 3H), 2.30 (s, 3H), 1.33 (t, J = 7.2 Hz, 3H). ^{19}F NMR (376 MHz, $CDCl_3$) δ -106.26 (m, 2F), -117.68 (m, 2F).

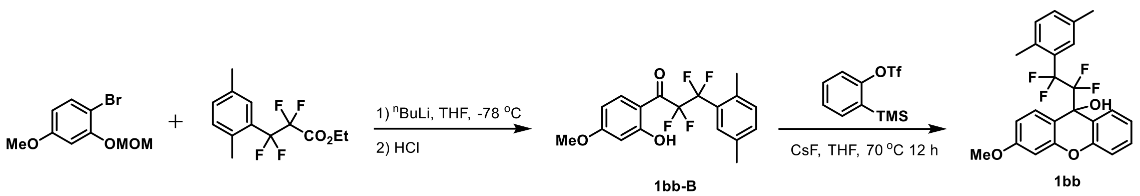

To an oven dried 2-necked 100 mL round bottom flask containing a stirring bar was added 1-bromo-4-methoxy-2-(methoxymethoxy)benzene (2.5 g, 10 mmol, 1.0 equiv) and 80 mL anhydrous THF under Ar atmosphere. To the reaction flask was added ^{n}BuLi (4.4 mL, 10.5 mmol, 1.05 equiv, 2.4 M in hexane) via a syringe at -78 ^{o}C. After stirring under Ar at the same temperature for 1 hour, a solution of ethyl 3-(2,5-dimethylphenyl)-2,2,3,3-tetrafluoropropanoate (2.9 g, 10.5 mmol, 1.05 equiv) in 3 mL anhydrous THF was added via a syringe, and the mixture was allowed to stir at same temperature for 3 hours. The reaction was treated with HCl (30 mL, 0.5 M), and then extracted with EtOAc (3 x 50 mL). The combined organic layer was dried over $MgSO_4$, filtered and concentrated in vacuo. The residue was dissolved in HCl (25 mL, 3.0 M in EtOAc). After stirring under air at room temperature for 2 hours, the mixture was concentrated in vacuo, and the residue was purified by flash chromatography (petroleum ether / ethyl acetate = 15:1) to afford the compound **1bb-B** as a gray solid (2.2 g, 63% yield).^{1}H NMR (400 MHz, $CDCl_3$) δ 12.17 (s, 1H), 7.87 (d, J = 9.1 Hz, 1H), 7.28 (s, 1H), 7.20 (d, J = 7.9 Hz, 1H), 7.13 (d, J = 7.8 Hz, 1H), 6.47 (m, 2H), 3.87 (s, 3H), 2.46 (t, J = 2.6 Hz, 3H), 2.33 (s, 3H). ^{19}F NMR (376 MHz, $CDCl_3$) δ -104.89, (s, 2F), -111.57 (t, J = 7.5 Hz, 2F). ^{13}C NMR (101 MHz, $CDCl_3$) δ187.7 (t, J = 25.7 Hz), 168.0, 167.76, 135.5, 134.7 (t, J = 2.0 Hz), 133.5 (tt, J = 17.7, 2.8 Hz), 132.4, 132.2, 128.9 (t, J = 8.6 Hz), 127.4 (t, J = 22.2 Hz), 117.7 (tt, J = 255.5, 33.6 Hz), 112,9 (tt, J = 263.6, 40.9 Hz), 110.9, 109.1, 100.8, 55.8, 20.9, 20.3 (m). HRMS (ESI): m/z [$(M+Na)^+$] calcd for $C_{18}H_{16}F_4NaO_3$, 379.0928. found, 379.0924.

To an oven dried 2-necked 100 mL round bottom flask containing a stirring bar was added 3-(2,5-dimethylphenyl)-2,2,3,3-tetrafluoro-1-(2-hydroxy-4-methoxyphenyl) propan-1-one (2.2 g, 6.3 mmol, 1.0 equiv), CsF (2.9 g, 18.9 mmol, 3.0 equiv), 2-(trimethylsilyl)phenyl trifluoromethanesulfonate (2.3 mL, 9.5 mmol, 1.5 equiv), and 60

mL anhydrous THF under Ar atmosphere. After stirring under Ar atmosphere at 70 ^{o}C for 12 h, the solvent was removed under reduced pressure and the residue was purified by flash chromatography (petroleum ether / ethyl acetate = 12:1) to afford product **1bb** as light-yellow solid (2.0 g, 73% yield).[45] ^{1}H NMR (400 MHz, $CDCl_3$) δ 7.86 (d, J = 7.8 Hz, 1H), 7.72 (d, J = 8.8 Hz, 1H), 7.42-7.36 (m, 1H), 7.19 (dd, J = 14.9, 7.9 Hz, 2H), 7.11-7.01 (m, 2H), 6.98 (d, J = 7.7 Hz, 1H), 6.75 (dd, J = 8.8, 2.5 Hz, 1H), 6.67 (d, J = 2.5 Hz, 1H), 3.81 (s, 3H), 3.30 (s, 1H), 2.25 (s, 3H), 2.14 (s, 3H). ^{19}F NMR (376 MHz, $CDCl_3$) δ -103.58 (s, 2F), -118.14 (d, J = 7.5 Hz, 2F). ^{13}C NMR (101 MHz, $CDCl_3$) δ 161.1, 152.2, 151.3, 134.9, 134.6 (t, J = 2.8 Hz), 131.9, 131.4, 130.2, 129.3, 129.1 (t, J = 23.2 Hz), 128.6 (t, J = 8.6 Hz), 128.4, 123.1, 120.9, 119.1 (tt, J = 256.5, 36.9 Hz), 116.5 (tt, J = 264.6, 36.9 Hz), 116.3, 112.9, 110.5, 100.6, 70.4 (t, J = 23.7 Hz), 55.5, 20.8, 20.0 (t, J = 3.5 Hz). HRMS (ESI): m/z [$(M+Na)^+$] calcd for $C_{24}H_{20}F_4NaO_3$, 455.1241. found, 455.1238.

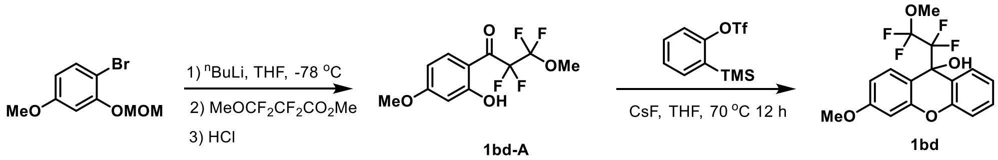

To an oven dried 2-necked 250 mL round bottom flask containing a stirring bar was added 1-bromo-4-methoxy-2-(methoxymethoxy)benzene (4.9 g, 20 mmol, 1.0 equiv) and 80 mL anhydrous THF under Ar atmosphere. To the reaction flask was added ^{n}BuLi (8.7 mL, 21 mmol, 1.05 equiv, 2.4 M in hexane) via a syringe at -78 ^{o}C. After stirring under Ar at same temperature for 1 h, $MeOCF_2F_2CO_2Me$ (4.3 mL, 30 mmol, 1.5 equiv) was added via a syringe, and the reaction mixture was allowed to stir at same temperature for 3 h. The reaction was quenched with HCl (60 mL, 0.5 M), and the mixture was extracted with EtOAc (3 x 100 mL). The combined organic layer was dried over $MgSO_4$, then filtered and concentrated in vacuo. The residue was dissolved in HCl (50 mL, 3.0 M in EtOAc). After stirring under air at room temperature for 2 h, the mixture was concentrated in vacuo, and the residue was purified by flash chromatography (petroleum ether / ethyl acetate = 15:1) to afford compound **1bd-A** as

light-yellow oil (4.4 g, 78% yield). ^{1}H NMR (400 MHz, $CDCl_3$) δ 12.01 (s, 1H), 7.84 (d, J = 9.2 Hz, 1H), 6.53-6.36 (m, 2H), 3.85 (s, 3H), 3.66 (s, 3H). ^{19}F NMR (376 MHz, $CDCl_3$) δ -89.78 (t, J = 5.6 Hz, 2F), -114.77 (t, J = 5.6 Hz, 2F). ^{13}C NMR (101 MHz, $CDCl_3$) δ186.5 (t, J = 25.2 Hz), 168.0, 167.8, 133.4 (t, J = 7.6 Hz), 117.7 (tt, J = 274.7, 31.8 Hz), 110.2 (tt, J = 265.6, 37.4 Hz), 110.1, 109.0, 100.9, 55.8, 51.5 (t, J = 6.6 Hz). HRMS (ESI): m/z [$(M+Na)^+$] calcd for $C_{11}H_{10}F_4NaO_4$, 305.0407. found, 305.0401.

To an oven dried 2-necked 250 mL round bottom flask containing a stirring bar was added 2,2,3,3-tetrafluoro-1-(2-hydroxy-4-methoxyphenyl)-3-methoxypropan-1-one (4.4 g, 15.6 mmol, 1.0 equiv), CsF (7.1 g, 45.8 mmol, 3.0 equiv), 2-(trimethylsilyl)phenyl trifluoromethanesulfonate (5.6 mL, 23.4 mmol, 1.5 equiv), and 150 mL anhydrous THF under Ar atmosphere. After stirring under Ar atmosphere at 70 ^{o}C for 12 h, the solvent was removed under reduced pressure and the residue was purified by flash chromatography (petroleum ether / ethyl acetate = 9:1) to afford product **1bd** as light-yellow solid (4.7 g, 85% yield). ^{1}H NMR (400 MHz, $CDCl_3$) δ 7.76 (dd, J = 7.9, 1.0 Hz, 1H), 7.63 (d, J = 8.8 Hz, 1H), 7.43-7.36 (m, 1H), 7.24-7.09 (m, 2H), 6.75 (dd, J = 8.8, 2.5 Hz, 1H), 6.67 (d, J = 2.5 Hz, 1H), 3.83 (s, 3H), 3.42 (s, 3H), 3.29 (s, 1H). ^{19}F NMR (376 MHz, $CDCl_3$) δ -86.7 (t, J = 5.6 Hz, 2F), -124.2 (q, J = 5.6 Hz, 2F). ^{13}C NMR (101 MHz, $CDCl_3$) δ 161.2, 152.3, 151.4, 130.2, 128.9, 127.9, 123.1, 120.5, 118.4 (tt, J = 274.7, 32.8 Hz), 116.2, 114.4 (tt, J = 265.6, 32.8 Hz), 112.4, 110.4, 100.6, 70.0 (t, J = 23.2 Hz), 55.5, 50.9 (t, J = 6.6 Hz). HRMS (ESI): m/z [$(M-OH)^+$] calcd for $C_{17}H_{13}F_4O_3$, 341.0801. found, 341.0797.

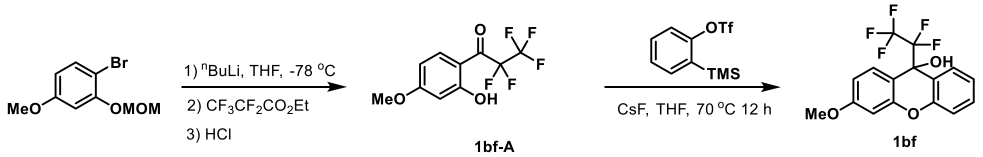

To an oven dried 2-necked 250 mL round bottom flask containing a stirring bar was added 1-bromo-4-methoxy-2-(methoxymethoxy)benzene (4.9 g, 20 mmol, 1.0 equiv) and 80 mL of anhydrous THF under Ar atmosphere. To the reaction flask was added ^{n}BuLi (8.7 mL, 21 mmol, 1.05 equiv, 2.4 M in hexane) via a syringe at -78 ^{o}C.

After stirring under Ar at the same temperature for 1 h, $CF_3CF_2CO_2Et$ (4.4 mL, 30 mmol, 1.5 equiv) was added via a syringe, and the reaction mixture was allowed to stir at the same temperature for 3 h. The reaction was quenched with HCl (60 mL, 0.5 M), and the mixture was extracted with EtOAc (3 x 100 mL). The combined organic layer was dried over $MgSO_4$, then filtered and concentrated in vacuo. The residue was dissolved in HCl (50 mL, 3.0 M in EtOAc). After stirring under air at room temperature for 2 h, the mixture was concentrated in vacuo, and the residue was purified by flash chromatography (petroleum ether / ethyl acetate = 20:1) to afford the compound **1bf-A** as light-yellow oil (4.7 g, 87% yield). ^{1}H NMR (400 MHz, $CDCl_3$) δ 11.61 (s, 1H), 7.69 (d, J = 9.3 Hz, 1H), 6.41 (dd, J = 9.3, 2.4 Hz, 1H), 6.36 (d, J = 2.3 Hz, 1H), 3.78 (s, 3H). ^{19}F NMR (376 MHz, $CDCl_3$) δ -81.69 (s, 3F), -114.01 (s, 2F). ^{13}C NMR (101 MHz, $CDCl_3$) δ 184.5 (t, J = 25.8 Hz), 168.3, 132.3 (t, J = 7.1 Hz), 118.0 (qt, J = 287.9, 33.8 Hz), 109.5, 109.2, 108.9 (tq, J = 268.7, 37.4 Hz), 101.1, 55.8. HRMS (ESI): m/z $[(M+Na)^+]$ calcd for $C_{10}H_7F_5NaO_3$, 293.0208. found, 293.0212.

To an oven dried 2-necked 250 mL round bottom flask containing a stirring bar was added 2,2,3,3,3-pentafluoro-1-(2-hydroxy-4-methoxyphenyl)propan-1-one (4.7 g, 17.4 mmol, 1.0 equiv), CsF (8.1 g, 52.2 mmol, 3.0 equiv), 2-(trimethylsilyl)phenyl trifluoromethanesulfonate (6.2 mL, 26.1 mmol, 1.5 equiv), and 150 mL anhydrous THF under Ar atmosphere. After stirring under Ar atmosphere at 70 ^{o}C for 12 h, the solvent was removed under reduced pressure and the residue was purified by flash chromatography (petroleum ether / ethyl acetate = 10:1) to afford product **1bf** as light-yellow solid (3.1 g, 52% yield). ^{1}H NMR (400 MHz, $CDCl_3$) δ 7.76 (d, J = 7.9 Hz, 1H), 7.61 (d, J = 8.8 Hz, 1H), 7.40 (t, J = 7.7 Hz, 1H), 7.18 (dd, J = 18.0, 7.9 Hz, 2H), 6.72 (dd, J = 8.8, 2.4 Hz, 1H), 6.64 (d, J = 2.4 Hz, 1H), 3.78 (s, 3H), 3.09 (s, 1H). ^{19}F NMR (376 MHz, $CDCl_3$) δ -78.36 (s, 3F), -124.13 (s, 2F). ^{13}C NMR (101 MHz, $CDCl_3$) δ 161.5, 152.4, 151.4, 130.8, 128.7, 127.8, 123.5, 119.5, 119.0 (qt, J = 288.9, 35.8 Hz), 116.6, 113.4 (tq, J = 264.6, 34.7 Hz), 111.4, 110.9, 100.9, 70.0 (t, J = 24.2 Hz), 55.5. HRMS (ESI): m/z $[(M-OH)^+]$ calcd for $C_{16}H_{10}F_5O_2$, 329.0601. found, 329.0601.

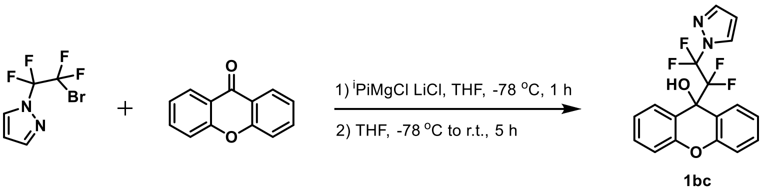

To an oven dried 2-necked 250 mL round bottom flask containing a stirring bar was added 1-(2-bromo-1,1,2,2-tetrafluoroethyl)-1H-pyrazole[50] (4.9 g, 20 mmol, 1.0 equiv) and 100 mL anhydrous THF under Ar atmosphere. To the reaction flask was added ^{i}PrMgCl LiCl (16.2 mL, 21 mmol, 1.05 equiv, 1.3 M in hexane) via a syringe at -78 ^{o}C over 10 min. After stirring under Ar at the same temperature for 1 h, a solution of 9H-xanthen-9-one (7.8 g, 40 mmol, 2.0 equiv) in anhydrous THF (50 mL) was added via a syringe, and the reaction mixture was allowed to stir at room temperature for 5 h. The reaction was quenched with 300 mL H_2O, and the mixture was extracted with EtOAc (3 x 100 mL). The combined organic layer was dried over $MgSO_4$, then filtered and concentrated in vacuo, and the residue was purified by flash chromatography (petroleum ether / ethyl acetate = 12:1) to afford compound **1bc** as light-gray solid (2.8 g, 38% yield).[49] ^{1}H NMR (400 MHz, $CDCl_3$) δ 7.82-7.68 (m, 4H), 7.42-7.33 (m, 2H), 7.25-7.04 (m, 4H), 6.40 (dd, J = 2.6, 1.9 Hz, 1H), 6.07 (s, 1H). ^{19}F NMR (376 MHz, $CDCl_3$) δ -89.10 (s, 2F), -118.61 (s, 2F). ^{13}C NMR (101 MHz, $CDCl_3$) δ 151.7, 142.8, 130.2, 129.7, 128.1, 123.2, 120.2, 116.5, 115.5 (tt, J = 267.7, 33.3 Hz), 114.4 (tt, J = 270.7, 35.4 Hz),108.3, 71.1 (t, J = 24.7 Hz). HRMS (ESI): m/z [$(M+Na)^+$] calcd for $C_{18}H_{12}F_4N_2NaO_2$, 387.0727. found, 387.0723.

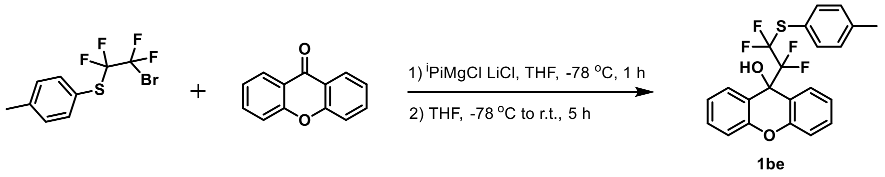

To an oven dried 2-necked 250 mL round bottom flask containing a stirring bar was added (2-bromo-1,1,2,2-tetrafluoroethyl)(p-tolyl)sulfane[51] (6.0 g, 20 mmol, 1.0 equiv) and 100 mL anhydrous THF under Ar atmosphere. To the reaction flask was added ^{i}PrMgCl LiCl (16.2 mL, 21 mmol, 1.05 equiv, 1.3 M in hexane) via a syringe at -78 ^{o}C over 10 min. After stirring under Ar at the same temperature for 1 h, a solution

of 9H-xanthen-9-one (7.8 g, 40 mmol, 2.0 equiv) in anhydrous THF (50 mL) was added via a syringe, and the reaction mixture was allowed to stir at room temperature for 5 h. The reaction was then quenched with 300 mL HCl (0.5 M), and the mixture was extracted with EtOAc (3 x 100 mL). The combined organic layer was dried over $MgSO_4$, then filtered and concentrated in vacuo, and the residue was purified by flash chromatography (petroleum ether / ethyl acetate = 18:1) to afford the compound **1be** as light-red oil (1.3 g, 16% yield). ^{1}H NMR (400 MHz, $CDCl_3$) ^{1}H NMR (400 MHz, CDCl3) δ 7.81 (dd, J = 7.9, 1.2 Hz, 2H), 7.41-7.36 (m, 2H), 7.34 (d, J = 8.1 Hz, 2H), 7.17 (m, 4H), 7.08 (d, J = 7.9 Hz, 2H), 3.14 (s, 1H), 2.30 (s, 3H). ^{19}F NMR (376 MHz, $CDCl_3$) δ -82.97 (t, J = 3.8 Hz, 2F), -117.34 (t, J = 5.6 Hz, 2F). ^{13}C NMR (101 MHz, $CDCl_3$) δ 151.4, 140.8, 137.2, 130.6, 129.9, 128.2, 125.0 (tt, J = 291.9, 36.9 Hz), 123.3, 120.5, 120.1, 116.5, 116.1 (tt, J = 266.6, 30.3 Hz), 70.7 (t, J = 24.2 Hz), 21.3. HRMS (ESI): m/z [$(M-OH)^+$] calcd for $C_{22}H_{15}F_4NaOS$, 403.0780. found, 403.0772.

2.2 General procedure for synthesis of alkenes 2

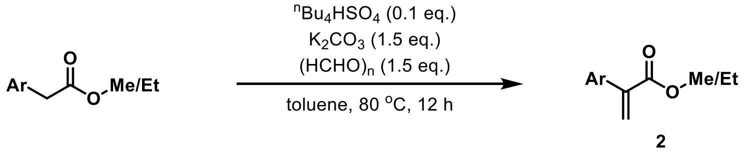

To a solution of an appropriate ester (1.0 eq.) in anhydrous toluene (10 mL), K_2CO_3 (1.5 eq.), tetrabutylammonium bisulfate (0.10 eq.), and formaldehyde (1.5 eq.) were added. The reaction mixture was heated at 80 °C for 12 h, then quenched with H_2O, and extracted with EtOAc. The combined organic layer was dried over $MgSO_4$ and concentrated under reduced pressure. The crude reaction mixture was purified by flash column chromatography to afford acrylates **2**.

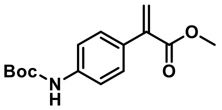

Methyl 2-(4-((tert-butoxycarbonyl)amino)phenyl)acrylate (2h): The product was purified with silica gel chromatography (petroleum ether / ethyl acetate = 8:1) as white solid (858 mg, 62% yield). ^{1}H NMR (400 MHz, $CDCl_3$) δ 7.33 (s, 4H), 6.52 (s, 1H),

6.27 (d, J = 1.1 Hz, 1H), 5.83 (d, J = 1.1 Hz, 1H), 3.79 (s, 3H), 1.50 (s, 9H). ^{13}C NMR (101 MHz, $CDCl_3$) δ 167.4, 152.6, 140.6, 138.4, 131.3, 129.0, 125.9, 118.0, 80.7, 52.2, 28.3. HRMS (ESI): m/z [$(M+Na)^+$] calcd for $C_{15}H_{19}NNaO_4$, 300.1206. found, 300.1210.

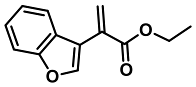

Ethyl 2-(benzofuran-3-yl)acrylate (2j): The product was purified with silica gel chromatography (petroleum ether / ethyl acetate = 25:1) as white solid (1.2 g, 70% yield). ^{1}H NMR (400 MHz, $CDCl_3$) δ 8.05 (s, 1H), 7.71-7.67 (m, 1H), 7.51 (d, J = 7.4 Hz, 1H), 7.30 (m, 2H), 6.51 (s, 1H), 6.23 (s, 1H), 4.31 (t, J = 7.1 Hz, 2H), 1.35 (t, J = 7.1 Hz, 3H). ^{13}C NMR (101 MHz, $CDCl_3$) δ 166.1, 155.1, 144.9, 132.0, 126.2, 125.7, 124.5, 123.0, 120.5, 116.3, 111.7, 61.3, 14.2. HRMS (ESI): m/z [$(2M+Na)^+$] calcd for $C_{26}H_{24}NaO_6$, 455.1465. found, 455.1468.

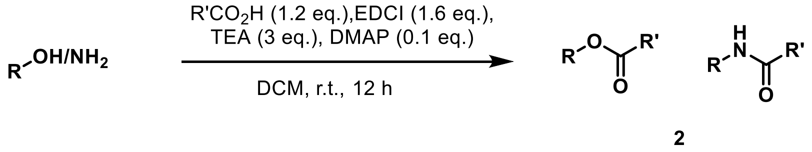

To a round-bottom flask were added a solution of an appropriate carboxylic acid (1.2 equiv) in DCM (5 mL), alcohols or amines (1.0 equiv), EDCI (1.5 equiv), TEA (3.0 equiv) and DMAP (0.1 equiv) at 0 °C. The reaction mixture was stirred at room temperature for 12 h, and then concentrated in vacuo. The crude product was purified by silica gel column chromatography to afford products **2**.

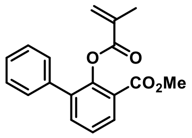

Methyl 2-(methacryloyloxy)-[1,1'-biphenyl]-3-carboxylate (2k): The product was purified with silica gel chromatography (petroleum ether / ethyl acetate = 20:1) as colorless oil (2.6 g, 88% yield). ^{1}H NMR (400 MHz, DMSO-d6) δ 7.97 (dd, J = 7.8, 1.7 Hz, 1H), 7.72 (dd, J = 7.7, 1.7 Hz, 1H), 7.52 (t, J = 7.7 Hz, 1H), 7.45-7.38 (m, 5H),

6.07 (s, 1H), 5.82-5.72 (m, 1H), 3.77 (s, 3H), 1.85 (s, 3H). ^{13}C NMR (101 MHz, DMSO-d6) δ 165.4, 165.0, 147.3, 136.6, 135.7, 135.3, 131.0, 129.7, 129.3, 128.8, 128.4, 128.2, 127.0, 124.3, 52.8, 18.3. HRMS (ESI): m/z [(M+Na)$^+$] calcd for $C_{18}H_{16}NaO_4$, 319.0941. found, 319.0945.

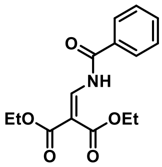

Diethyl 2-(benzamidomethylene)malonate (2m): The product was purified with silica gel chromatography (petroleum ether / ethyl acetate = 8:1) as white solid (1.2 g, 84% yield). ^{1}H NMR (400 MHz, $CDCl_3$) δ 12.00 (d, J = 11.5 Hz, 1H), 8.76 (d, J = 11.8 Hz, 1H), 7.97 (d, J = 8.0 Hz, 2H), 7.61 (d, J = 7.6 Hz, 1H), 7.52 (t, J = 7.7 Hz, 2H), 4.34 (q, J = 7.1 Hz, 2H), 4.26 (q, J = 7.1 Hz, 2H), 1.35 (m, 6H). ^{13}C NMR (101 MHz, $CDCl_3$) δ 167.9, 164.4, 164.2, 146.8, 133.5, 131.5, 129.0, 127.9, 102.7, 61.3, 60.8, 14.2, 14.1. HRMS (ESI): m/z [(M+H)$^+$] calcd for $C_{15}H_{18}NO_5$, 292.1179. found, 292.1180.

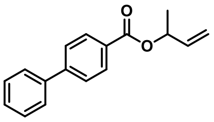

But-3-en-2-yl [1,1'-biphenyl]-4-carboxylate (2u): The product was purified with silica gel chromatography (petroleum ether / ethyl acetate = 20:1) as colorless oil (1.2 g, 95% yield). ^{1}H NMR (400 MHz, $CDCl_3$) δ 8.19-8.11 (m, 2H), 7.67-7.60 (m, 4H), 7.48-7.42 (m, 2H), 7.42-7.36 (m, 1H), 5.98 (m, 1H), 5.70-5.56 (m, 1H), 5.35 (dt, J = 17.3, 1.3 Hz, 1H), 5.20 (dt, J = 10.6, 1.2 Hz, 1H), 1.47 (d, J = 6.5 Hz, 3H). ^{13}C NMR (101 MHz, $CDCl_3$) δ 165.7, 145.6, 140.1, 137.8, 130.1, 129.4, 128.9, 128.2, 127.3, 127.1, 115.9, 71.6, 20.1. HRMS (ESI): m/z [(M+Na)$^+$] calcd for $C_{17}H_{16}NaO_2$, 275.1043. found, 275.1040.

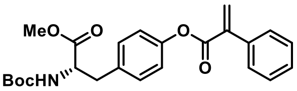

(S)-4-(2-((Tert-butoxycarbonyl)amino)-3-methoxy-3-oxopropyl)phenyl 2-phenylacrylate (2aa): The product was purified with silica gel chromatography (petroleum ether / ethyl acetate = 5:1) as white solid (2.5 g, 73% yield). ^{1}H NMR (400 MHz, $CDCl_3$) δ 7.49 (d, J = 6.9 Hz, 2H), 7.36 (d, J = 6.9 Hz, 3H), 7.15 (d, J = 8.2 Hz, 2H), 7.09 (d, J = 8.3 Hz, 2H), 6.58 (s, 1H), 6.06 (s, 1H), 5.00 (d, J = 8.0 Hz, 1H), 4.57 (dd, J = 13.3, 6.2 Hz, 1H), 3.69 (s, 3H), 3.09 (m, 2H), 1.41 (s, 9H). ^{13}C NMR (101 MHz, $CDCl_3$) δ 172.1, 165.0, 155.0, 149.8, 140.7, 136.2, 133.6, 130.2, 128.3, 128.3, 128.1, 121.5, 110.0, 80.0, 54.3, 52.2, 37.6, 28.3. HRMS (ESI): m/z [(M+Na)$^+$] calcd for $C_{24}H_{27}NNaO_6$, 448.1731. found, 448.1734.

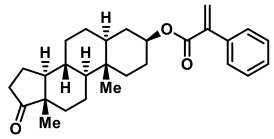

(3S,5S,8R,9S,10S,13S,14S)-10,13-Dimethyl-17-oxohexadecahydro-1H-cyclopenta[a]phenanthren-3-yl 2-phenylacrylate (2ab): The product was purified with silica gel chromatography (petroleum ether / ethyl acetate = 20:1) as white solid (3.0 g, 90% yield). ^{1}H NMR (400 MHz, $CDCl_3$) δ 7.51-7.39 (m, 2H), 7.39-7.27 (m, 3H), 6.30 (d, J = 1.2 Hz, 1H), 5.86 (d, J = 1.1 Hz, 1H), 4.91-4.79 (m, 1H), 2.43 (dd, J = 19.2, 8.7 Hz, 1H), 2.14-2.01 (m, 1H), 1.97-1.88 (m, 2H), 1.84-1.65 (m, 5H), 1.61-1.41 (m, 4H), 1.38-1.21 (m, 6H), 1.13-0.97 (m, 2H), 0.86 (s, 6H), 0.73 (td, J = 11.7, 3.9 Hz, 1H). ^{13}C NMR (101 MHz, $CDCl_3$) δ 166.4, 141.8, 136.8, 128.2, 128.0, 126.1, 74.4, 54.2, 51.3, 47.8, 44.7, 36.7, 35.9, 35.7, 35.0, 33.9, 31.5, 30.8, 28.3, 27.4, 21.8, 20.5, 13.8, 12.3. HRMS (ESI): m/z [(M+Na)$^+$] calcd for $C_{28}H_{36}NaO_3$, 443.2557. found, 443.2562.

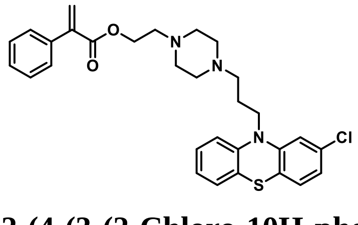

2-(4-(3-(2-Chloro-10H-phenothiazin-10-yl)propyl)piperazin-1-yl)ethyl 2-phenylacrylate (2ac): The product was purified with silica gel chromatography

(petroleum ether / ethyl acetate = 4:1) as white solid (1.7 g, 80% yield). ^{1}H NMR (400 MHz, $CDCl_3$) δ 7.50-7.27 (m, 5H), 7.13 (dd, J = 16.6, 8.0 Hz, 2H), 7.01 (d, J = 8.1 Hz, 1H), 6.96-6.82 (m, 4H), 6.34 (s, 1H), 5.89 (s, 1H), 4.34 (t, J = 5.8 Hz, 2H), 3.88 (t, J = 6.8 Hz, 2H), 2.68 (t, J = 5.8 Hz, 2H), 2.47 (m, 10H), 1.96-1.90 (m, 2H). ^{13}C NMR (101 MHz, $CDCl_3$) δ 166.7, 146.4, 144.5, 141.4, 136.6, 133.2, 128.2, 128.1, 128.0, 127.8, 127.5, 127.4, 126.6, 124.7, 123.4, 122.8, 122.2, 115.8, 115.8, 62.6, 56.5, 55.5, 53.4, 53.3, 45.3, 24.3. HRMS (ESI): m/z [$(M+H)^+$] calcd for $C_{30}H_{33}{}^{35}ClN_3O_2S$, 534.1977. found, 534.1976.

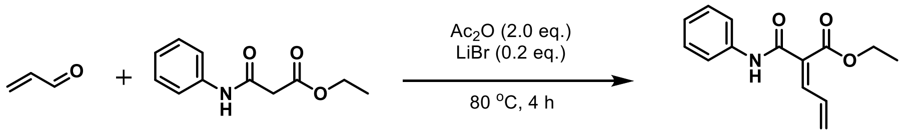

2p

To a round-bottomed flask (50 mL) equipped with a magnetic stirrer and a vapor condenser fitted with a septum-held gas-inlet tube were added Ac_2O (1.6 mL, 20 mmol), anhydrous LiBr (170 mg, 2 mmol) and ethyl 3-oxo-3-(phenylamino)propanoate (2.1 g, 10 mmol). The resulting mixture was stirred for 1 h at 80 °C under nitrogen atmosphere. Then, acrolein (1.7 mL, 30 mmol) was added in one portion through the vapor condenser and the solution was stirred at 80 °C until full consumption of malonate. The reaction mixture was allowed to cool to room temperature, and slowly decomposed in a solution of Na_2CO_3 (25 mL). The aqueous phase was extracted with Et_2O (2 × 20 mL) and the combined organic phases were washed with brine and, after dried over $MgSO_4$, were evaporated under reduced pressure. The residue was purified by flash chromatography to afford ethyl 2-(phenylcarbamoyl)penta-2,4-dienoate **2p** (1.4 g, 54% yield) as a white solid. ^{1}H NMR (400 MHz, $CDCl_3$) δ 9.93 (s, 1H), 7.83-7.69 (m, 1H), 7.60 (t, J = 10.9 Hz, 3H), 7.32 (t, J = 7.8 Hz, 2H), 7.10 (t, J = 7.4 Hz, 1H), 5.80 (dd, J = 22.8, 13.4 Hz, 2H), 4.30 (q, J = 7.1 Hz, 2H), 1.36 (t, J = 7.1 Hz, 3H). ^{13}C NMR (101 MHz, $CDCl_3$) δ 167.3, 161.6, 150.7, 137.8, 133.6, 131.5, 128.9, 124.3, 123.7, 120.3, 61.9, 14.1. HRMS (ESI): m/z [$(M+Na)^+$] calcd for $C_{14}H_{15}NNaO_3$, 268.0944. found, 268.0944.

2.3 General procedure for synthesis of alkenes 4

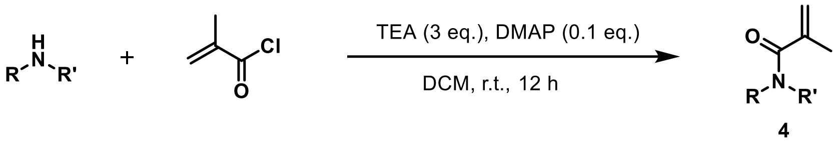

To a round-bottom flask were added corresponding amines (1.0 equiv) in DCM (1 mmol / 5 mL), then added alcohols or amines (1.0 equiv), EDCI (1.5 equiv). TEA (3.0 equiv) and DMAP (0.1 equiv) at 0 °C. The reaction mixture was stirred at room temperature for 12 hours, then concentrated in vacuo. The crude product was purified by silica gel column chromatography to afford the product **4**.

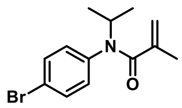

N-(4-Bromophenyl)-N-isopropylmethacrylamide (4j): The product was purified with silica gel chromatography (petroleum ether / ethyl acetate = 6:1) as white solid (2.0 g, 90% yield). ^{1}H NMR (400 MHz, $CDCl_3$) δ 7.48 (d, J = 8.6 Hz, 2H), 7.05-6.88 (m, 2H), 5.01-4.81 (m, 3H), 1.74 (s, 3H), 1.11 (d, J = 6.8 Hz, 6H). ^{13}C NMR (101 MHz, $CDCl_3$) δ 171.6, 141.3, 138.2, 131.9, 131.9, 121.7, 118.1, 46.8, 21.0, 20.7. HRMS (ESI): m/z [(M+H)$^+$] calcd for $C_{13}H_{17}{}^{79}BrNO$, 282.0488. found, 282.0486.

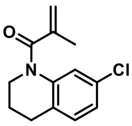

1-(7-Chloro-3,4-dihydroquinolin-1(2H)-yl)-2-methylprop-2-en-1-one (4n): The product was purified with silica gel chromatography (petroleum ether / ethyl acetate = 4:1) as white solid (2.1 g, 88% yield). ^{1}H NMR (400 MHz, $CDCl_3$) δ 7.32 (d, J = 1.1 Hz, 1H), 7.10-7.01 (m, 2H), 5.25 (d, J = 1.0 Hz, 1H), 5.16 (s, 1H), 3.82-3.75 (m, 2H), 2.75 (t, J = 6.7 Hz, 2H), 2.02-1.91 (m, 5H). ^{13}C NMR (101 MHz, $CDCl_3$) δ 171.5, 140.9, 139.8, 131.1, 129.5, 129.2, 124.7, 124.1, 119.2, 44.3, 26.5, 23.7, 19.8. HRMS (ESI): m/z [(M+H)$^+$] calcd for $C_{13}H_{15}{}^{35}ClNO$, 236.0837. found, 236.0840.

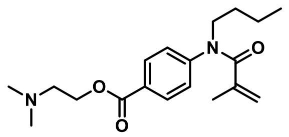

2-(Dimethylamino)ethyl 4-(N-butylmethacrylamido)benzoate (4x): The product was purified with silica gel chromatography (petroleum ether / ethyl acetate = 2:1) as white solid (756 mg, 38% yield). ^{1}H NMR (400 MHz, $CDCl_3$) δ 8.02 (d, J = 8.5 Hz, 2H), 7.17 (d, J = 8.5 Hz, 2H), 5.03 (d, J = 1.2 Hz, 1H), 4.95 (d, J = 0.9 Hz, 1H), 4.42 (dd, J = 8.1, 3.4 Hz, 2H), 3.86-3.76 (m, 2H), 2.72 (t, J = 5.7 Hz, 2H), 2.34 (d, J = 1.1 Hz, 6H), 1.78 (d, J = 0.9 Hz, 3H), 1.57-1.45 (m, 2H), 1.32 (m, 2H), 0.88 (t, J = 8.0 Hz, 3H). ^{13}C NMR (101 MHz, $CDCl_3$) δ 171.5, 165.8, 147.6, 140.7, 130.7, 128.4, 126.8, 119.8, 63.1, 57.8, 49.4, 45.8, 29.9, 20.3, 20.1, 13.8. HRMS (ESI): m/z [$(M+H)^+$] calcd for $C_{19}H_{29}N_2O_3$, 333.2173. found, 333.2177.

3. Condition optimization

Table S1. Screening of difluoroalkylation reagents[a]

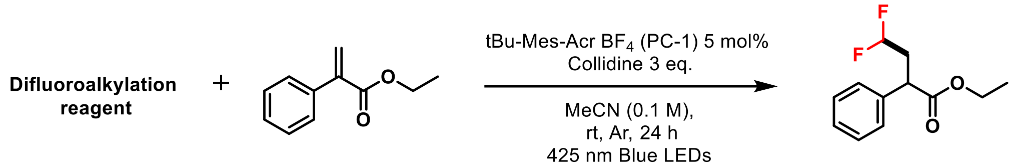

1 (2 equiv.) **2a** (1 equiv.) **3a**

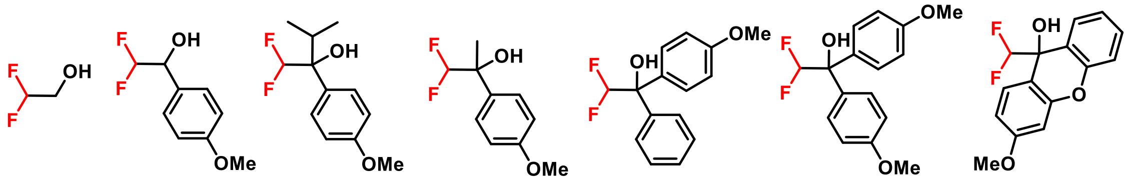

**1a-I**	**1a-II**	**1a-III**	**1a-IV**	**1a-V**	**1a-VI**	**1a-VII**
$E_{p/2}$ = 2.19 V vs SCE	$E_{p/2}$ = 1.72 V vs SCE	$E_{p/2}$ = 1.70 V vs SCE	$E_{p/2}$ = 1.69 V vs SCE	$E_{p/2}$ = 1.75 V vs SCE	$E_{p/2}$ = 1.70 V vs SCE	$E_{p/2}$ = 1.71 V vs SCE

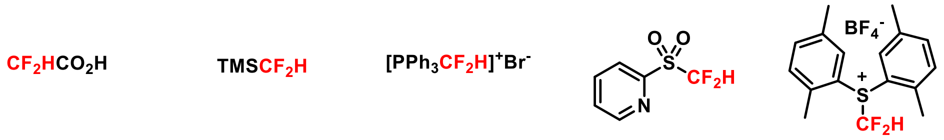

1a-S1 **1a-S2** **1a-S3** **1a-S4** **1a-S5**

Entry	Difluoroalkylation reagent	Effective BDFE (kcal/mol)	Yield (%)[b]
1	**1a-I**	117.2	0
2	**1a-II**	106.4	0
3	**1a-III**	105.9	0
4	**1a-IV**	105.7	70
5	**1a-V**	107.1	53
6	**1a-VI**	105.9	62
7	**1a-VII**	106.2	57
8	**1a-S1**	-	0
9	**1a-S2**	-	0
10	**1a-S3**	-	0
11	**1a-S4**	-	0
12	**1a-S5**	-	trace

[a] **1** (0.4 mmol), **2a** (0.2 mmol), PC-1 (5 mol%), 2,4,6-Collidine (3.0 equiv.), MeCN (2.0 ml), 425nm blue LEDs, ambient temperature, 24 h. [b] Yields were determined by GC-MS with n-dodecan as an internal standard. Effective BDFE = 23.06 * $Ep_{1/2}(Fc/Fc^+)$ + 1.373 *15 (pKa of 2,4,6-Collidine) + 54.9 kcal/mol in MeCN.

Table S2. Screening of solvents[a]

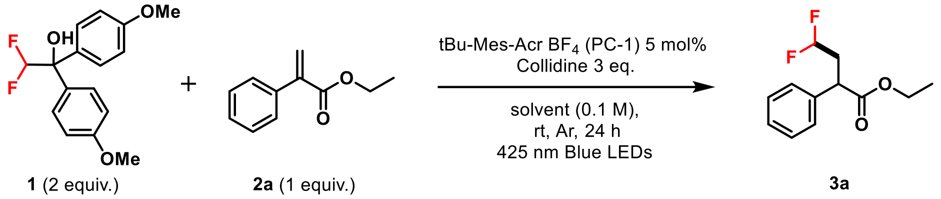

Entry	Difluoroalkylation reagent	Yield (%)[b]
1	**MeCN**	62
2	**DMSO**	0
3	**DMF**	0
4	**EA**	0
5	**Toluene**	0
6	**THF**	0
7	**DCE**	28

[a] **1a-V** (0.4 mmol), **2a** (0.2 mmol), PC-1 (5 mol%), 2,4,6-Collidine (3.0 equiv.), solvent (2.0 ml), 425nm blue LEDs, ambient temperature, 24 h. [b] Yields were determined by GC-MS with n-dodecan as an internal standard

Table S3. Screening of catalysts[a]

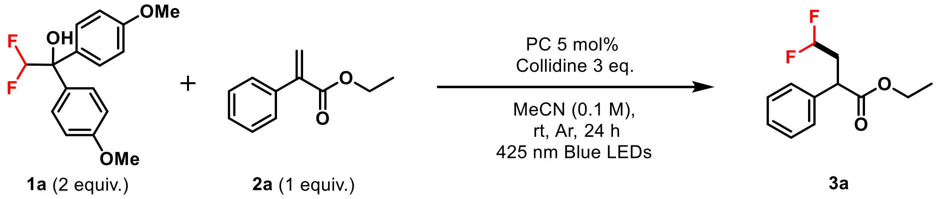

Entry	photocatalyst	$E_{1/2}(C/C^{-})$ (eV)	$E_{1/2}(C^{*}/C^{-})$ (eV)	Yield (%)[b]
1	**PC-1**[29]	**-0.59**	**2.08**	62
2	**PC-2**[30]	**-0.54**	**2.00**	55
3	**PC-3**	**-0.55**	**2.07**	53
4	**PC-4**	**-0.46**	**2.16**	47
5	**PC-5**	**-0.60**	**1.98**	80
6	**PC-6**	**-0.56**	**1.88**	68
7	**PC-7**[29]	**-0.69**	**1.68**	>5
8	**PC-8**[29]	**-2.01**	**0.89**	0
9	**PC-9**[29]	**-1.33**	**0.77**	0
10	**PC-10**[29]	**-1.21**	**1.38**	0

[a] **1a-V** (0.4 mmol), **2a** (0.2 mmol), PC (5 mol%), 2,4,6-Collidine (3.0 equiv.), MeCN (2.0 ml), 425nm blue LEDs, ambient temperature, 24 h. [b] Yields were determined by GC-MS with n-dodecan as an internal standard.

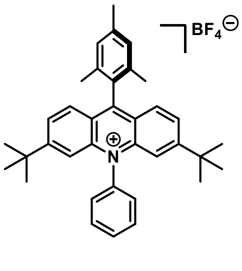

PC-1

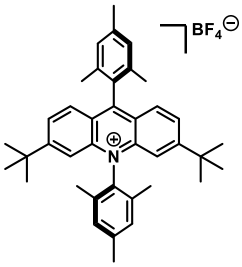

PC-2

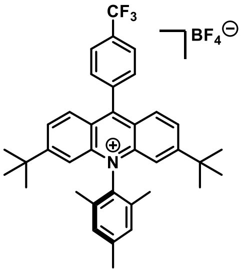

PC-3

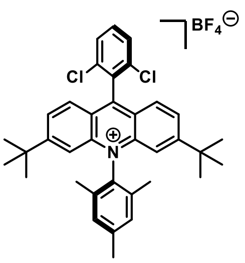

PC-4

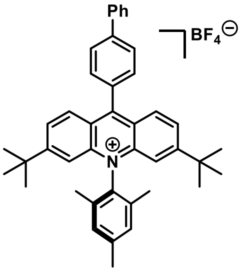

PC-5

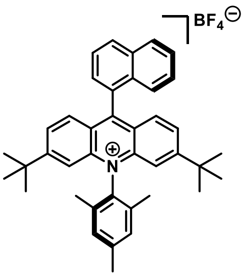

PC-6

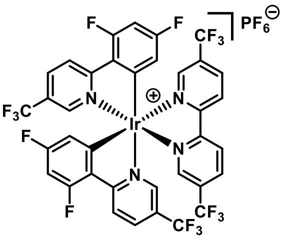

PC-7
Ir(dFCF$_3$ppy)$_2$(5,5'dCF$_3$bpy)PF$_6$

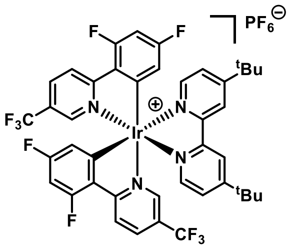

PC-8
Ir(dFCF$_3$ppy)$_2$(dtbbpy)PF$_6$

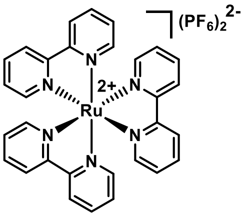

PC-9
Ru(bpy)$_3$(PF$_6$)$_2$

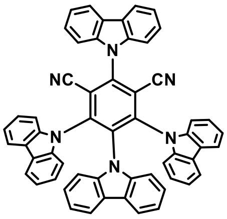

PC-10
4CzIPN

Table S4. Screening of bases[a]

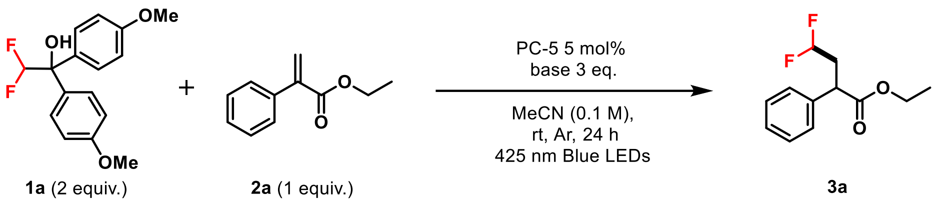

Entry	base	Yield (%)[b]
1	**collidine**	80
2	**DBU**	0
3	**TEA**	0
4	**Na_2CO_3**	0
5	**K_3PO_4**	0
6	**TBA^+ $(PhO)_2POO^-$**	6
7	**TBA^+ CF_3OO^-**	27

[a] **1a-V** (0.4 mmol), **2a** (0.2 mmol), PC-5 (5 mol%), base (3.0 equiv.), MeCN (2.0 ml), 425nm blue LEDs, ambient temperature, 24 h. [b] Yields were determined by GC-MS with n-dodecan as an internal standard.

Table S5. Screening of additives[a]

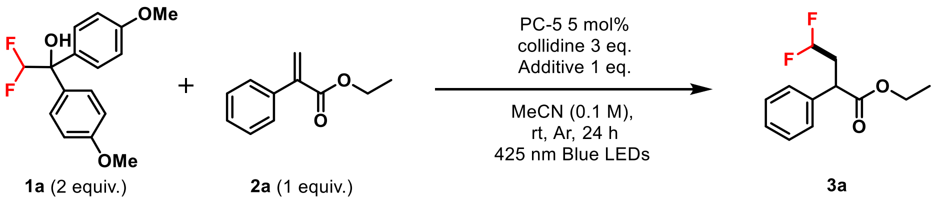

Entry	additive	Yield (%)[b]
1	**H_2O**	73
2	**CF_3CO_2H**	0
3	**$(CF_3)_2CHOH$**	84
4	**CF_3CH_2OH**	89
5[c]	**CF_3CH_2OH**	85

[a] **1a** (0.4 mmol), **2a** (0.2 mmol), PC-5 (5 mol%), 2,4,6-Collidine (3.0 equiv.), additive (1.0 equiv.), MeCN (2.0 ml), 425nm blue LEDs, ambient temperature, 24 h. [b] Yields were determined by GC-MS with n-dodecan as an internal standard. [c] additive (2.0 equiv.).

Table S6. Screening of trifluoroalkylation reagents[a]

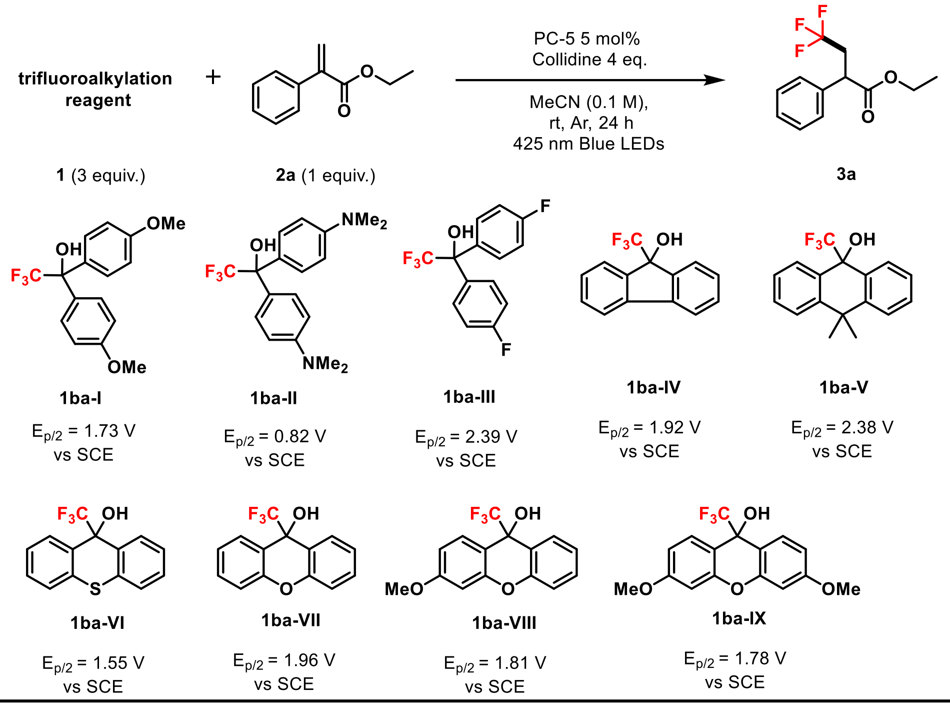

Entry	Difluoroalkylation reagent	Effective BDFE (kcal/mol)	Yield (%)[b]
1	**1ba-I**	106.6	0
2	**1ba-II**	85.6	0
3	**1ba-III**	121.9	0
4	**1ba-IV**	111.0	0
5	**1ba-V**	121.6	0
6	**1ba-VI**	102.5	27
7	**1ba-VII**	111.9	75
8	**1ba-VIII**	108.5	92
9	**1ba-IX**	107.8	66

[a] **1** (0.6 mmol), **2a** (0.2 mmol), PC-5 (5 mol%), 2,4,6-Collidine (4.0 equiv.), TFE (1.0 equiv.), MeCN (2.0 ml), 425nm blue LEDs, ambient temperature, 24 h. [b] Yields were determined by GC-MS with n-dodecan as an internal standard. Effective BDFE = 23.06 * $Ep_{1/2}(Fc/Fc^{+})$ + 1.373 *15 (pKa of 2,4,6-Collidine) + 54.9 kcal/mol in MeCN.

Cyclic voltammograms of starting materials

All voltammograms were taken at room temperature using a saturated calomel (SCE) reference electrode, a platinum (Pt) counter electrode, and a glassy carbon working electrode. The conditions of the experiments were the following: an acetonitrile solution of 100 mM tetrabutylammonium hexafluorophosphate (NBu_4PF_6)

and 5 mM starting material, a scan rate of 0.1 V/s, and a positive initial scan direction. The reported potentials were averages over segments, and were taken at half-height of the cathodic peaks ($E_{p/2}$) of the compounds, since all oxidations were nonreversible. To convert the potentials from SCE to Fc/Fc^+ reference, 380 mV were subtracted from the measured values.

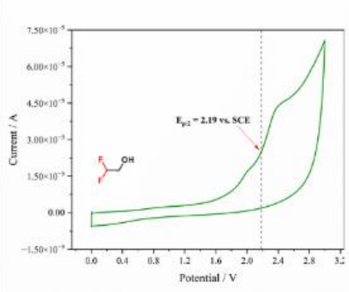

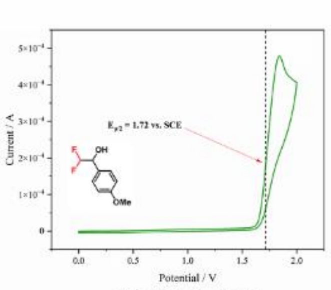

CV of 1a-II in MeCN

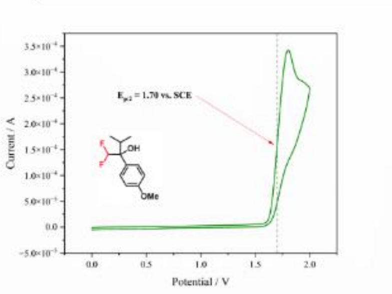

CV of 1a-III in MeCN

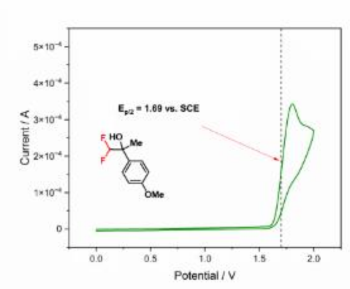

CV of 1a-IV in MeCN

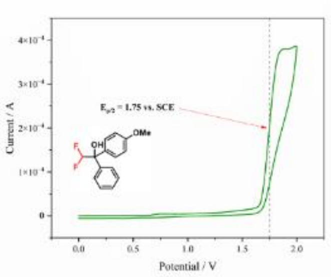

CV of 1a-V in MeCN

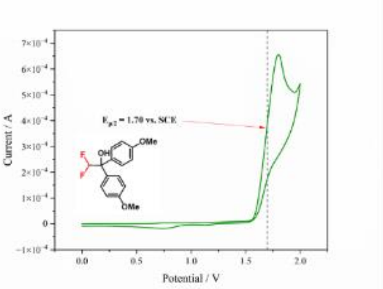

CV of 1a-VI in MeCN

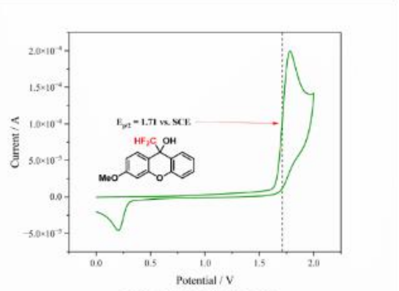

CV of 1a-VI in MeCN

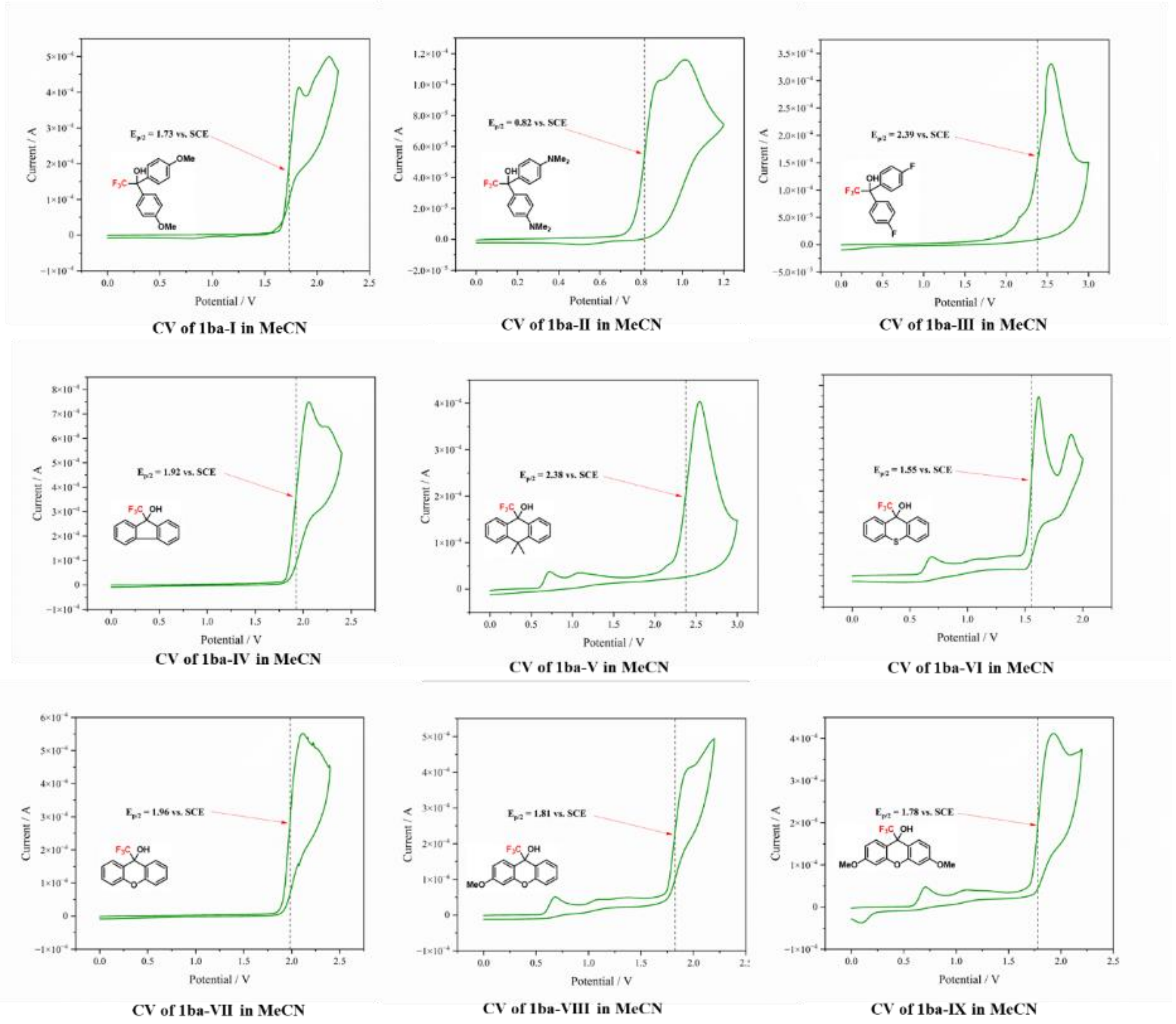

Figure S2. Cyclic voltammograms of fluoroalkylation reagents

Synthesis of photocatalysts

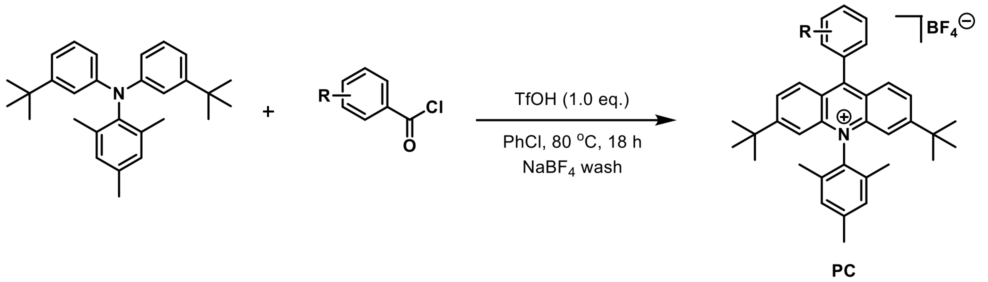

A dried Schlenk tube was evacuated and backfilled with argon three times. Under a stream of argon, dry chlorobenzene (1 mmol / 3 mL) was added, followed by N,N-bis(3-(tert-butyl)phenyl)-2,4,6-trimethylaniline (1.0 equiv) and acyl chloride (2.1 equiv), then, triflic acid (1.0 equiv) was added slowly to this mixture. The tube was sealed and the reaction was stirred at 80 °C for 18 h. After the reaction was cooled down, DCM (1 mmol / 6 mL) was added, the organic layer was washed with $NaBF_4$ (aq. sol., 1 M, 3 x 1 mmol / 10 mL). The organic layer was dryed with $NaBF_4$, filtered, concentrated and separated in Et_2O until a precipitate was formed. The precipitation was supported by further addition of Et_2O and stirring for 30 min. The solid was filtered off, washed with Et_2O and dried under reduced pressure to get the photocatalyst.[30]

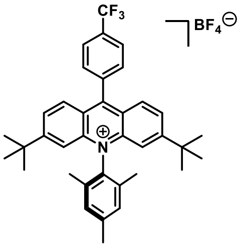

3,6-Di-tert-butyl-10-mesityl-9-(4-(trifluoromethyl)phenyl)acridin-10-ium tetrafluoroborate (PC-3): The product was obtained as yellow solid (250 mg, 39% yield). ^1H NMR (400 MHz, $CDCl_3$) δ 8.03 (d, J = 8.0 Hz, 2H), 7.96 (d, J = 9.1 Hz, 2H), 7.88 (d, J = 8.9 Hz, 4H), 7.36 (s, 4H), 2.57 (s, 3H), 1.78 (s, 6H), 1.31 (s, 18H). ^{19}F NMR (376 MHz, $CDCl_3$) δ -62.77 (s, 3F), -154.41 (s, 4F). ^{13}C NMR (176 MHz, $CDCl_3$) δ 164.9, 159.4, 142.2, 141.1, 136.5, 134.7, 132.52 (q, J = 33.4 Hz), 132.3, 130.9, 130.8,

129.8, 127.7, 126.1 (q, J = 3.5 Hz), 123.8 (q, J = 271.9 Hz), 124.1, 112.9, 36. 7, 30.4, 21.4, 17.1. HRMS (ESI): m/z [(M-BF$_4$)$^+$] calcd for $C_{37}H_{39}F_3N$, 554.3029. found, 554.3029.

Spectrophotometric and electrochemical data of PC-3

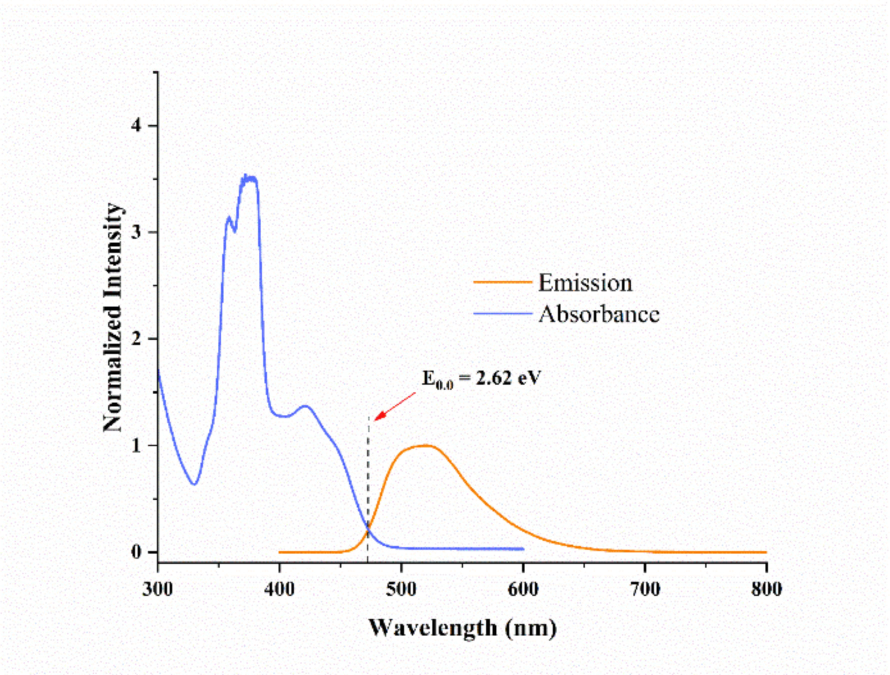

Figure S3. UV/visible absorption spectrum and emission spectrum of photocatalyst PC-3 in MeCN.

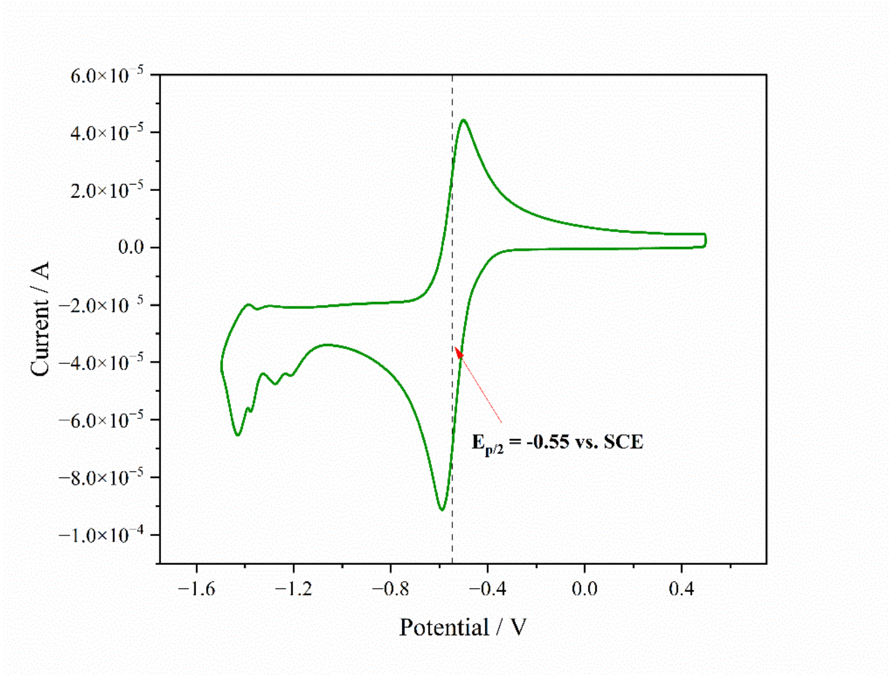

Figure S4. Cyclic voltammerty measurement of photocatalyst PC-3 in MeCN.

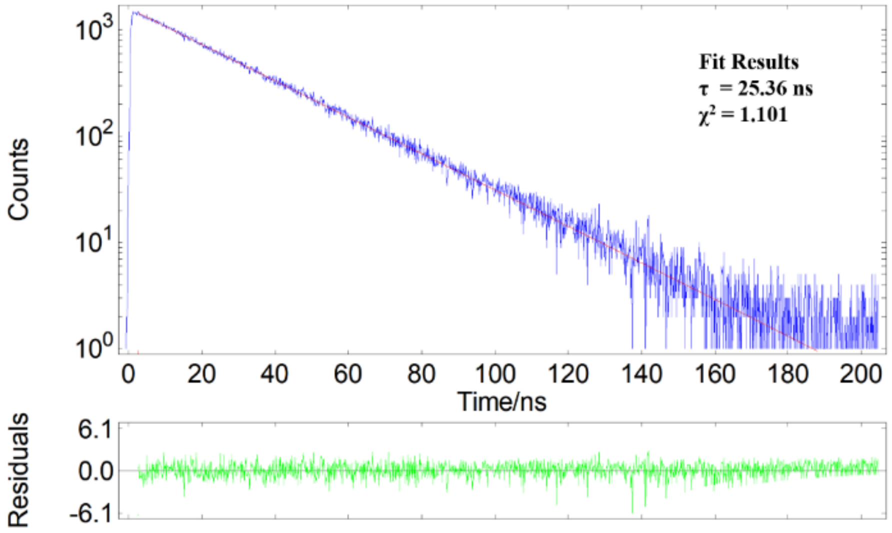

Figure S5. Lifetime measurement results of PC-3.

$E_{0,0}$ = 2.62 eV

$E_{1/2}(C/C^{-})$ = -0.55 V vs. SCE

$E_{1/2}(C^{*}/C^{-})$ = 2.62 + (-0.55) = 2.07 V vs. SCE

τ = 25.36 ns

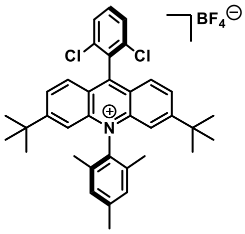

3,6-Di-tert-butyl-9-(2,6-dichlorophenyl)-10-mesitylacridin-10-ium tetrafluoroborate (PC-4): The product was obtained as yellow solid (300 mg, 47% yield). ^{1}H NMR (400 MHz, $CDCl_3$) δ 7.99 (dd, J = 9.1, 1.6 Hz, 2H), 7.86 (d, J = 9.1 Hz, 2H), 7.83-7.74 (m, 3H), 7.41 (d, J = 7.8 Hz, 4H), 2.60 (s, 3H), 1.75 (s, 6H), 1.34 (s, 18H). ^{19}F NMR (376 MHz, $CDCl_3$) δ -154.49 (s, 4F). ^{13}C NMR (176 MHz, $CDCl_3$) δ 165.9, 155.8, 142.8, 141.2, 134.1, 134.0, 133.4, 131.9, 131.2, 130.3, 129.3, 128.7, 128.4, 123.7, 113.2, 36.9, 30.4, 21.5, 16.8. HRMS (ESI): m/z [$(M-BF_4)^+$] calcd for $C_{36}H_{38}{}^{35}Cl_2N$, 554.2376. found, 554.2379.

Spectrophotometric and electrochemical data of PC-4

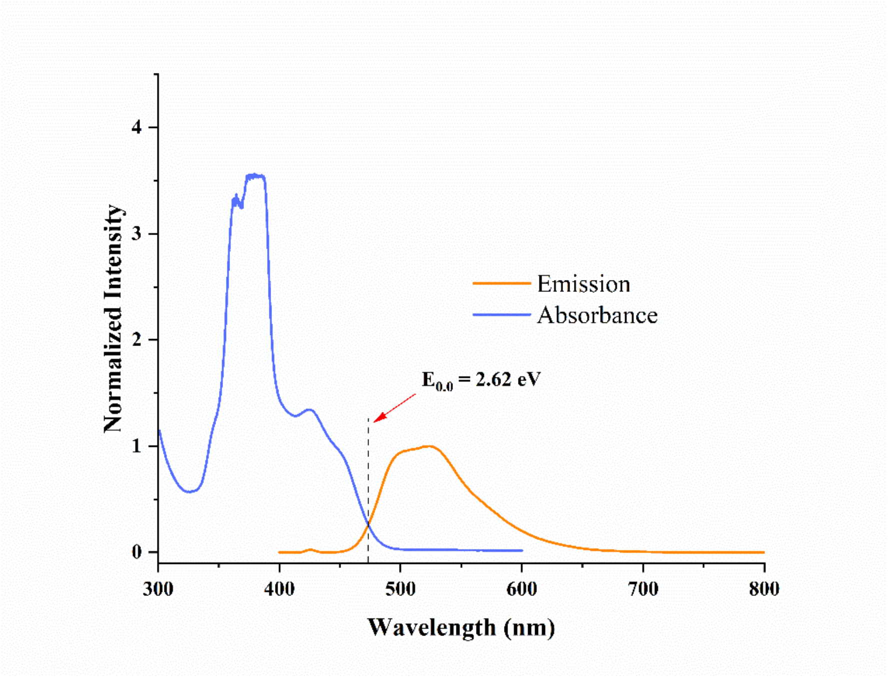

Figure S6. UV/visible absorption spectrum and emission spectrum of photocatalyst PC-4 in MeCN.

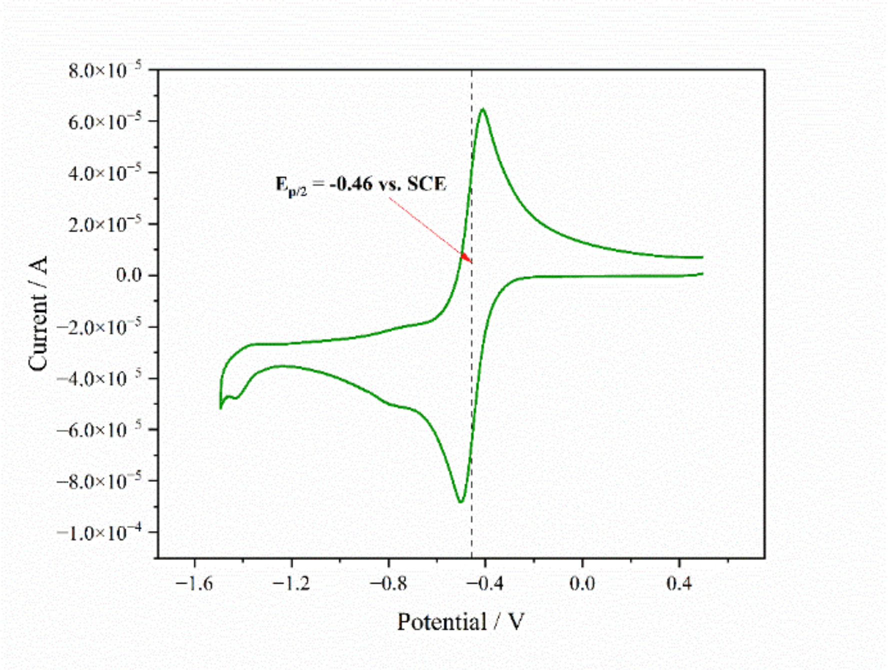

Figure S7. Cyclic voltammerty measurement of photocatalyst PC-4 in MeCN.

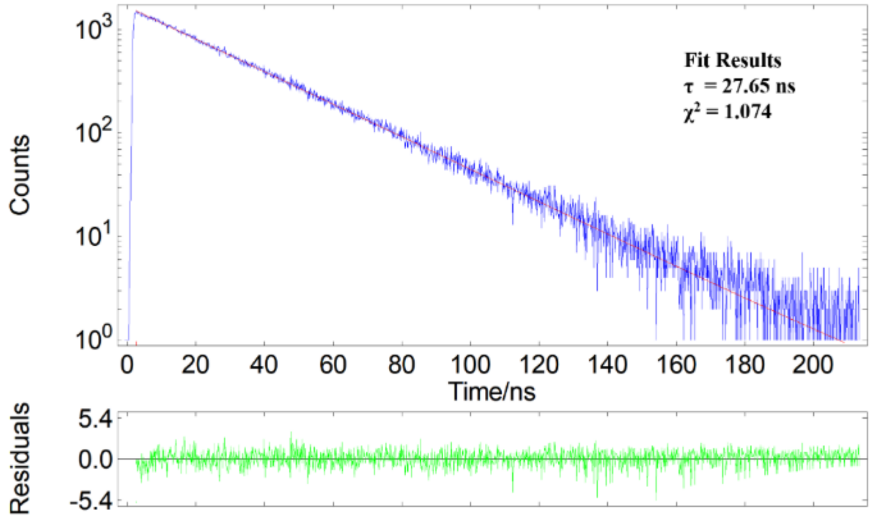

Figure S8. Lifetime measurement results of PC-4.

$E_{0,0}$ = 2.62 eV

$E_{1/2}(C/C^{-})$ = -0.46 V vs. SCE

$E_{1/2}(C^{*}/C^{-})$ = 2.62 + (-0.46) = 2.16 V vs. SCE

τ = 27.65 ns

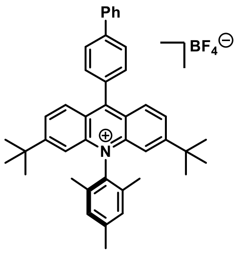

9-([1,1'-Biphenyl]-4-yl)-3,6-di-tert-butyl-10-mesitylacridin-10-ium tetrafluoroborate (PC-5): The product was obtained as yellow solid (5.1 g, 52% yield). ^{1}H NMR (400 MHz, $CDCl_3$) δ 8.16 (d, J = 9.1 Hz, 2H), 7.97 (d, J = 8.0 Hz, 2H), 7.88 (d, J = 9.2 Hz, 2H), 7.76 (d, J = 7.7 Hz, 2H), 7.72 (d, J = 8.1 Hz, 2H), 7.53 (t, J = 7.6 Hz, 2H), 7.44 (t, J = 7.4 Hz, 1H), 7.34 (d, J = 10.3 Hz, 4H), 2.56 (s, 3H), 1.75 (s, 6H), 1.29 (s, 18H). ^{19}F NMR (376 MHz, $CDCl_3$) δ -154.57 (s, 4F). ^{13}C NMR (176 MHz, $CDCl_3$) δ 164.8, 161.4, 143.6, 142.3, 141.1, 139.6, 134.6, 132.2, 131.3, 131.0, 130.8, 130.4, 129.1, 128.3, 127.7, 127.4, 127.4, 124.2, 112.8, 36.7, 30.4, 21.4, 17.1. HRMS (ESI): m/z [$(M-BF_4)^{+}$] calcd for $C_{42}H_{44}N$, 562.3468. found, 562.3467.

Spectrophotometric and electrochemical data of PC-5

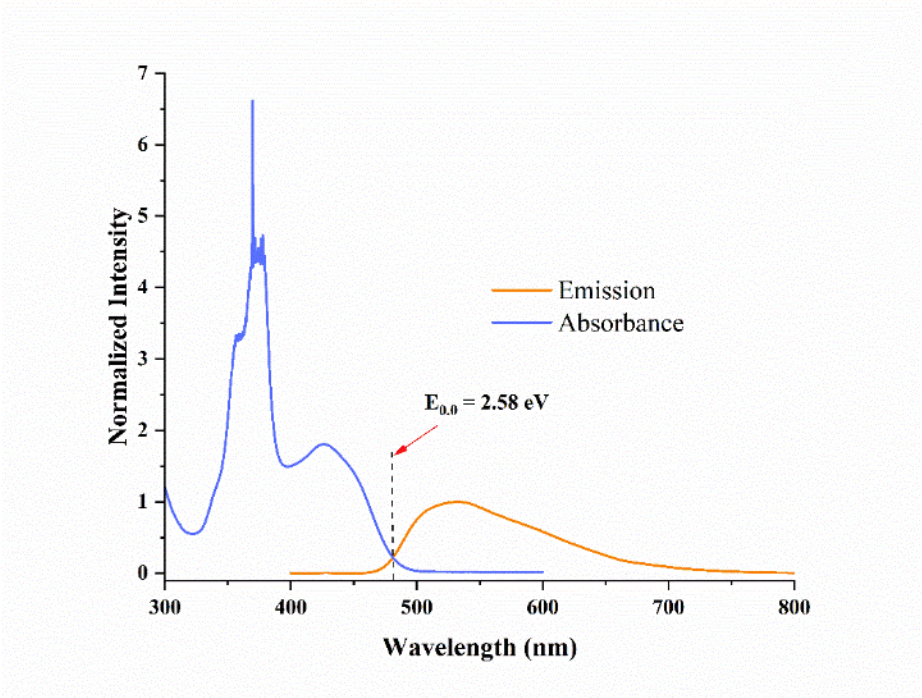

Figure S9. UV/visible absorption spectrum and emission spectrum of photocatalyst PC-5 in MeCN.

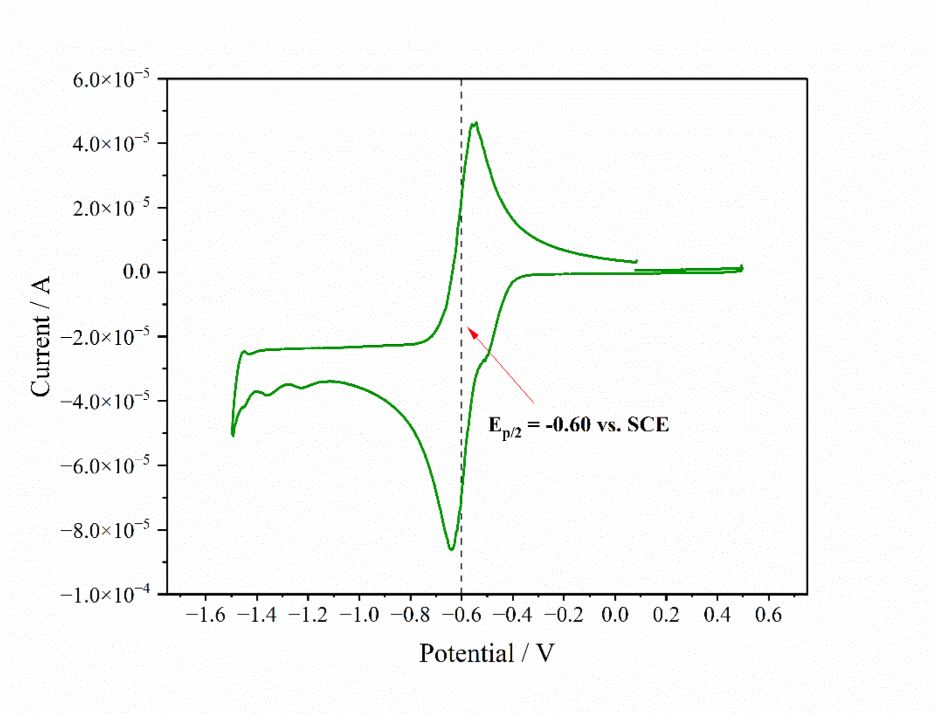

Figure S10. Cyclic voltammerty measurement of photocatalyst PC-5 in MeCN.

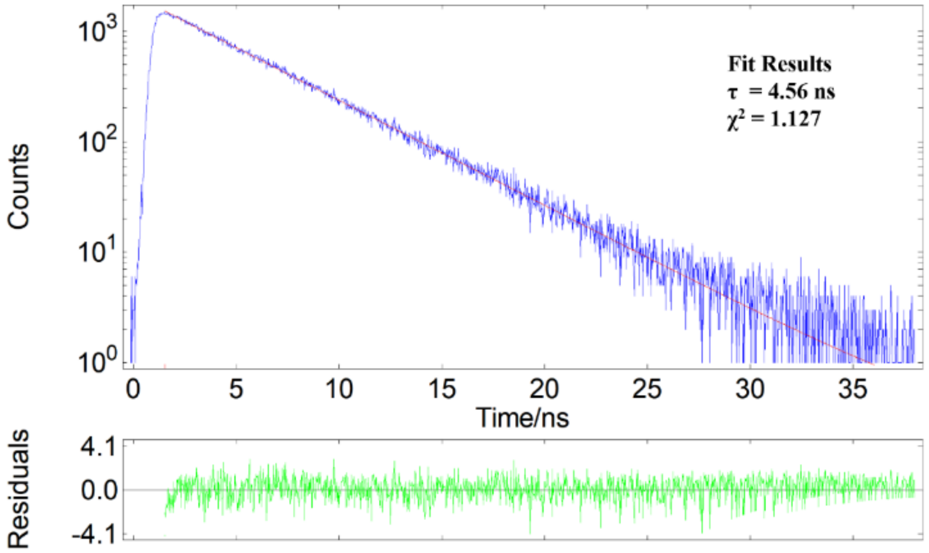

Figure S11. Lifetime measurement results of PC-5.

$E_{0,0}$ = 2.58 eV

$E_{1/2}(C/C^{-})$ = -0.60 V vs. SCE

$E_{1/2}(C^{*}/C^{-})$ = 2.58 + (-0.60) = 1.98 V vs. SCE

τ = 4.56 ns

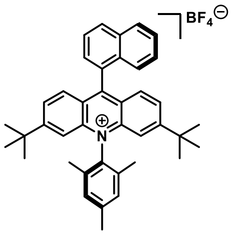

3,6-Di-tert-butyl-10-mesityl-9-(naphthalen-1-yl)acridin-10-ium tetrafluoroborate (PC-6): The product was obtained as yellow solid (286 mg, 46% yield). ^{1}H NMR (400 MHz, $CDCl_3$) δ 8.21 (d, J = 8.2 Hz, 1H), 8.08 (d, J = 8.3 Hz, 1H), 7.83 (t, J = 7.6 Hz, 1H), 7.74 (m, 5H), 7.59 (t, J = 7.6 Hz, 1H), 7.43-7.36 (m, 5H), 6.96 (d, J = 8.4 Hz, 1H), 2.57 (s, 3H), 1.83 (s, 3H), 1.81 (s, 3H), 1.27 (s, 18H). ^{19}F NMR (376 MHz, $CDCl_3$) δ -154.58 (s, 4F). ^{13}C NMR (176 MHz, $CDCl_3$) δ 165.2, 160.9, 142.4, 141.0, 134.7, 134.3, 133.4, 132.1, 131.6, 131.1, 131.1, 130.1, 129.9, 128.9, 128.9, 128.1, 127.6, 127.2, 125.6,

125.0, 125.0, 112.9, 36.7, 30.3, 21.5, 17.1, 17.0. HRMS (ESI): m/z [(M-BF_4)$^+$] calcd for $C_{40}H_{42}N$, 536.3312. found, 536.3319.

Spectrophotometric and electrochemical data of PC-6

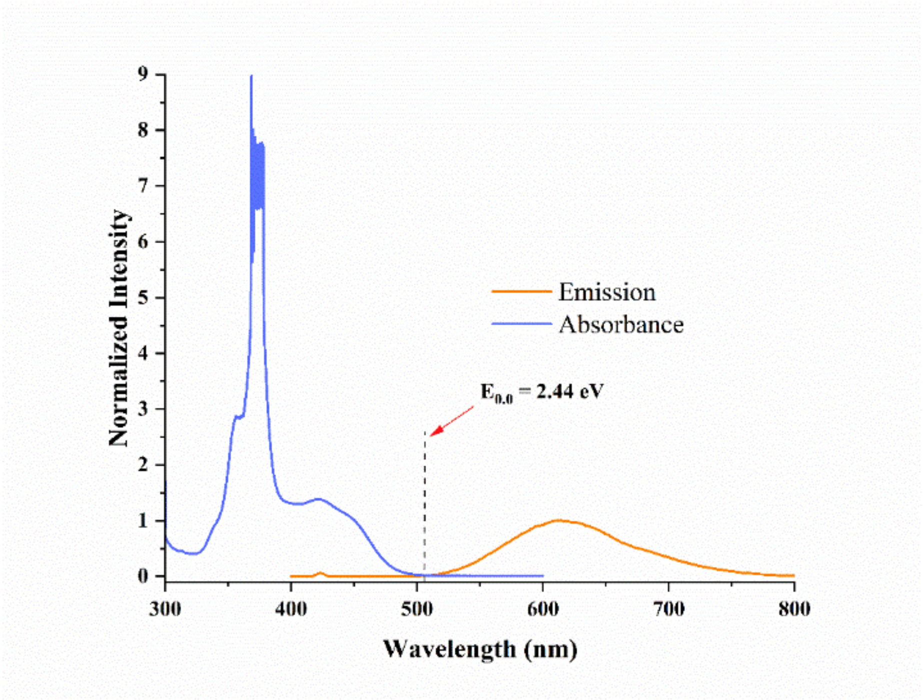

Figure S12. UV/visible absorption spectrum and emission spectrum of photocatalyst PC-6 in MeCN.

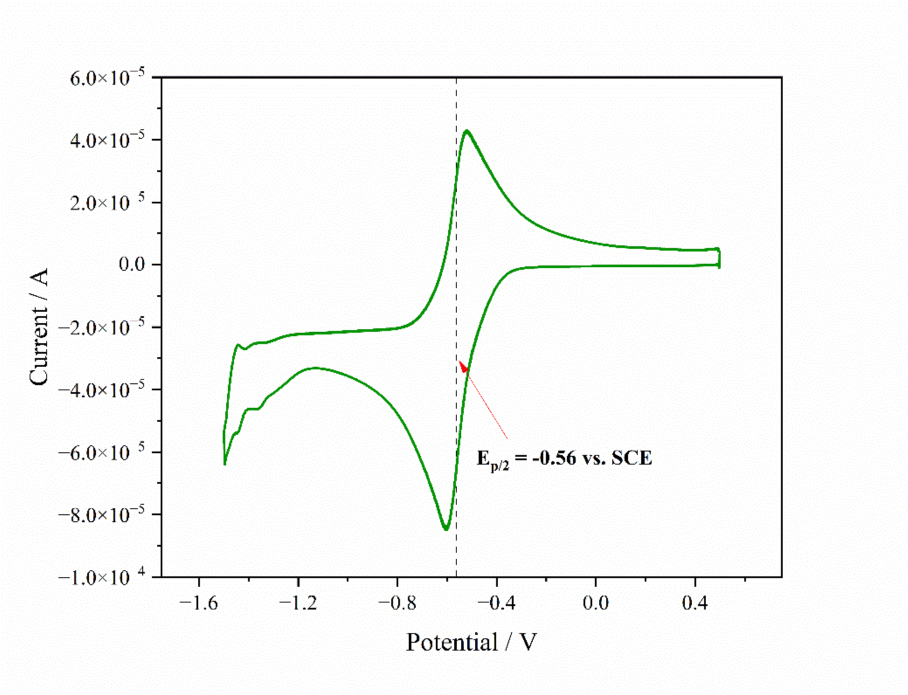

Figure S13. Cyclic voltammerty measurement of photocatalyst PC-6 in MeCN.

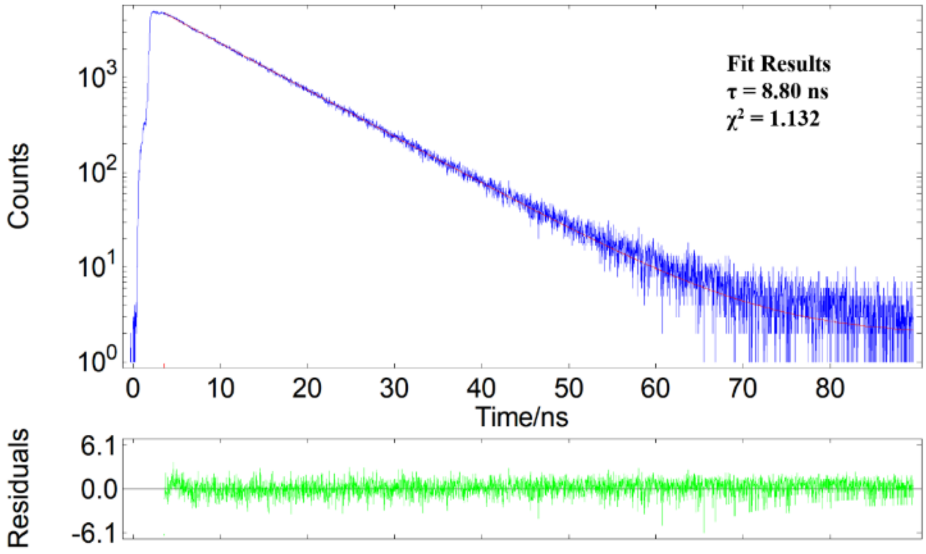

Figure S14. Lifetime measurement results of PC-6.

$$E_{0,0} = 2.44 \text{ eV}$$

$$E_{1/2}(C/C^{-}) = -0.56 \text{ V vs. SCE}$$

$$E_{1/2}(C^{*}/C^{-}) = 2.44 + (-0.56) = 1.88 \text{ V vs. SCE}$$

$$\tau = 8.80 \text{ ns}$$

4. General procedures for synthesis of compounds

4.1 Representative experimental procedure for synthesis of compounds 3

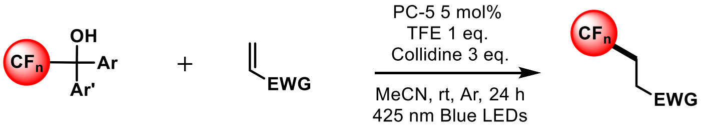

1 **2** **3**

To a 4 mL vial equipped with a stir bar was added fluorinated alcohol **1** (0.4 mmol, 2.0 equiv.), PC-5 (6.5 mg, 0.01 mmol, 5 mol%) and electron-withdrawing alkene **2** (0.2 mmol, 1.0 equiv.). The vial was sealed, evacuated and backfilled with Argon three times, then TFE (14 µL, 0.2 mmol, 1.0 equiv.) collidine (78 µL, 0.6 mmol, 3.0 equiv.) and 2 mL of dry MeCN (2 mL) were added. After degassing with Argon balloon for 8 minutes, the reaction mixture was irradiated with 10 W blue LEDs lamps for 24 hours at ambient

temperature. The reaction mixture was then concentrated and purified on a preparative TLC with petroleum ether/ethyl acetate as the eluent to afford the product **3**.

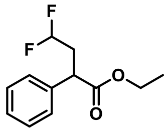

Ethyl 4,4-difluoro-2-phenylbutanoate (3a): The compound was according to the general procedure and was purified with silica gel chromatography (petroleum ether / ethyl acetate = 25:1) as colorless liquid (39.7 mg, 87% yield). ^{1}H NMR (400 MHz, $CDCl_3$) δ 7.40-7.26 (m, 5H), 5.93-5.55 (m, 1H), 4.40-3.97 (m, 2H), 3.79 (t, J = 7.7 Hz, 1H), 2.76-2.58 (m, 1H), 2.36-2.18 (m, 1H), 1.20 (t, J = 7.1 Hz, 3H). ^{19}F NMR (376 MHz, $CDCl_3$) δ -117.23 (m, 2F). ^{13}C NMR (101 MHz, $CDCl_3$) δ 172.5, 137.5, 129.0, 127.8, 127.7, 115.8 (t, J = 239.9 Hz), 61.3, 45.5 (t, J = 5.6 Hz), 37.6 (t, J = 22.2 Hz), 14.0. HRMS (ESI): m/z [$(M+Na)^+$] calcd for $C_{12}H_{14}F_2NaO_2$, 251.0854. found, 251.0855.

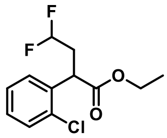

Ethyl 2-(2-chlorophenyl)-4,4-difluorobutanoate (3b): The compound was according to the general procedure and was purified with silica gel chromatography (petroleum ether / ethyl acetate = 25:1) as colorless liquid (42.4 mg, 81% yield). ^{1}H NMR (400 MHz, $CDCl_3$) δ 7.51-7.30 (m, 1H), 7.23 (m 3H), 5.81 (tt, J = 56.6, 4.7 Hz, 1H), 4.41-4.22 (m, 1H), 4.20-4.08 (m, 2H), 2.75-2.61 (m, 1H), 2.23 (m, 1H), 1.19 (t, J = 7.1 Hz, 3H). ^{19}F NMR (376 MHz, $CDCl_3$) δ -116.76 (m, 2F). ^{13}C NMR (101 MHz, $CDCl_3$) δ 172.0, 135.7, 133.7, 130.1, 129.0, 128.9, 127.4, 115.7 (t, J = 240.4 Hz), 61.6, 42.3 (t, J = 6.1 Hz), 36.7 (t, J = 22.2 Hz), 14.0. HRMS (ESI): m/z [$(M+Na)^+$] calcd for $C_{12}H_{13}{}^{35}ClF_2NaO_2$, 285.0464. found, 285.0467.

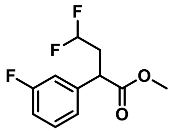

Methyl 4,4-difluoro-2-(3-fluorophenyl)butanoate (3c): The compound was according to the general procedure and was purified with silica gel chromatography (petroleum ether / ethyl acetate = 25:1) as colorless liquid (39.0 mg, 84% yield). ^{1}H NMR (400 MHz, $CDCl_3$) δ 7.32 (dd, J = 14.3, 7.6 Hz, 1H), 7.07 (d, J = 7.8 Hz, 1H), 7.00 (m, 2H), 5.74 (tt, J = 56.4, 4.6 Hz, 1H), 3.82 (t, J = 7.7 Hz, 1H), 3.69 (s, 3H), 2.74-2.59 (m, 1H), 2.34-2.20 (m, 1H). ^{19}F NMR (376 MHz, $CDCl_3$) δ -111.92 (m, 1F), -117.25 (m, 2F). ^{13}C NMR (101 MHz, $CDCl_3$) δ 172.5, 163.0 (d, J = 247.5 Hz), 139.6 (d, J = 8.1 Hz), 130.6 (d, J = 8.1 Hz), 123.5 (d, J = 3.0 Hz), 115.5 (t, J = 240.4 Hz), 115.0 (d, J = 12.1 Hz), 114.8 (d, J = 14.1 Hz), 52.6, 45.0 (t, J = 7.1 Hz), 37.4 (t, J = 22.2 Hz). HRMS (ESI): m/z [$(M+Na)^+$] calcd for $C_{11}H_{11}F_3NaO_2$, 255.0603. found, 255.0609.

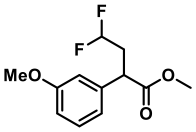

Methyl 4,4-difluoro-2-(3-methoxyphenyl)butanoate (3d): The compound was according to the general procedure and was purified with silica gel chromatography (petroleum ether / ethyl acetate = 20:1) as colorless liquid (38.5 mg, 79% yield). ^{1}H NMR (400 MHz, $CDCl_3$) δ 7.25 (t, J = 7.8 Hz, 1H), 6.92-6.76 (m, 3H), 5.71 (tt, J = 56.5, 4.7 Hz, 1H), 3.79-3.74 (m, 4H), 3.66 (s, 3H), 2.70-2.56 (m, 1H), 2.26 (m, 1H). ^{19}F NMR (376 MHz, $CDCl_3$) -117.37 (m, 2F). ^{13}C NMR (101 MHz, $CDCl_3$) δ 172.9, 160.0, 138.7, 130.1, 120.0, 115.8, 113.6 (t, J = 239.9 Hz), 113.2, 55.2, 52.5, 45.3 (t, J = 6.1 Hz), 37.5 (t, J = 22.2 Hz). HRMS (ESI): m/z [$(M+Na)^+$] calcd for $C_{12}H_{14}F_2NaO_3$, 267.0803. found, 267.0808.

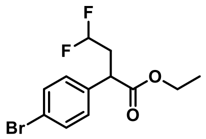

Ethyl 2-(4-bromophenyl)-4,4-difluorobutanoate (3e): The compound was according to the general procedure and was purified with silica gel chromatography (petroleum ether / ethyl acetate = 25:1) as yellowish liquid (55.7 mg, 91% yield). ^{1}H NMR (400 MHz, $CDCl_3$) δ 7.46 (d, J = 8.4 Hz, 2H), 7.16 (d, J = 8.4 Hz, 2H), 5.72 (tt, J = 56.4, 4.6 Hz, 1H), 4.17-4.03 (m, 2H), 3.74 (t, J = 7.7 Hz, 1H), 2.72-2.57 (m, 1H), 2.27-2.14 (m, 1H), 1.18 (t, J = 7.1 Hz, 3H). ^{19}F NMR (376 MHz, $CDCl_3$) -117.24 (m, 2F). ^{13}C NMR (101 MHz, $CDCl_3$) δ 172.0, 136.5, 132.3, 129.5, 121.9, 115.6 (t, J = 240.4 Hz), 61.5, 44.9 (t, J = 6.1 Hz), 37.4 (t, J = 22.2 Hz), 14.0. HRMS (ESI): m/z [$(M+Na)^+$] calcd for $C_{12}H_{13}{}^{79}BrF_2NaO_2$, 328.9959. found, 328.9951.

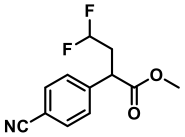

Methyl 2-(4-cyanophenyl)-4,4-difluorobutanoate (3f): The compound was according to the general procedure and was purified with silica gel chromatography (petroleum ether / ethyl acetate = 10:1) as white solid (36.8 mg, 77% yield). ^{1}H NMR (400 MHz, $CDCl_3$) δ 7.71-7.62 (d, J = 8.2 Hz, 2H), 7.42 (d, J = 8.3 Hz, 2H), 5.78 (tt, J = 56.2, 4.5 Hz, 1H), 3.90 (m, 1H), 3.70 (s, 3H), 2.79-2.65 (m, 1H), 2.32-2.19 (m, 1H). ^{19}F NMR (376 MHz, $CDCl_3$) -117.15 (m, 2F). ^{13}C NMR (101 MHz, $CDCl_3$) δ 171.9, 142.6, 132.8, 128.7, 118.3, 115.2 (t, J = 240.4 Hz), 112.0, 52.8, 45.2 (t, J = 7.1 Hz), 37.21 (t, J = 22.2 Hz). HRMS (ESI): m/z [$(M+H)^+$] calcd for $C_{12}H_{12}F_2NO_2$, 240.0831. found, 240.0831.

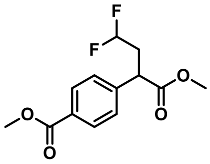

Methyl 4-(4,4-difluoro-1-methoxy-1-oxobutan-2-yl)benzoate (3g): The compound was according to the general procedure and was purified with silica gel chromatography

(petroleum ether / ethyl acetate = 10:1) as white solid (40.2 mg, 74% yield). Known compound.[31] ^{1}H NMR (400 MHz, $CDCl_3$) δ 8.02 (d, J = 8.3 Hz, 2H), 7.37 (d, J = 8.3 Hz, 2H), 5.75 (tt, J = 56.4, 4.6 Hz, 1H), 3.95-3.86 (m, 4H), 3.69 (s, 3H), 2.79-2.62 (m, 1H), 2.34-2.20 (m, 1H). ^{19}F NMR (376 MHz, $CDCl_3$) -117.29 (m, 2F). ^{13}C NMR (101 MHz, $CDCl_3$) δ 172.4, 166.6, 142.3, 130.3, 129.8, 127.9, 115.5 (t, J = 240.4 Hz), 52.6, 52.2, 45.2 (t, J = 6.1 Hz), 37.3 (t, J = 22.2 Hz).

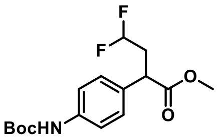

Methyl 2-(4-((tert-butoxycarbonyl)amino)phenyl)-4,4-difluorobutanoate (3h): The compound was according to the general procedure for 48 hours and was purified with silica gel chromatography (petroleum ether / ethyl acetate = 4:1) as white solid (40.8 mg, 62% yield). ^{1}H NMR (400 MHz, $CDCl_3$) δ 7.32 (d, J = 8.3 Hz, 2H), 7.18 (d, J = 8.5 Hz, 2H), 6.45 (s, 1H), 5.69 (tt, J = 56.5, 4.7 Hz, 1H), 3.74 (t, J = 7.8 Hz, 1H), 3.64 (s, 3H), 2.69-2.53 (m, 1H), 2.32-2.17 (m, 1H), 1.49 (s, 9H). ^{19}F NMR (376 MHz, $CDCl_3$) -117.28 (m, 2F). ^{13}C NMR (101 MHz, $CDCl_3$) δ 173.0, 152.6, 138.0, 131.6, 129.5, 128.3, 119.0, 115.7 (t, J = 239.9 Hz), 52.4, 44.6 (t, J = 6.1 Hz), 37.4 (t, J = 22.2 Hz), 29.7, 28.3. HRMS (ESI): m/z [$(M+Na)^+$] calcd for $C_{16}H_{21}F_2NNaO_4$, 352.1331. found, 352.1338.

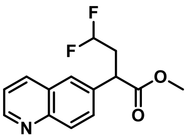

Methyl 4,4-difluoro-2-(quinolin-6-yl)butanoate (3i): The compound was according to the general procedure with 10 mol% PC-5 and was purified with silica gel chromatography (petroleum ether / ethyl acetate = 2:1) as white solid (40.0 mg, 75% yield). ^{1}H NMR (400 MHz, $CDCl_3$) δ 8.92 (dd, J = 4.2, 1.7 Hz, 1H), 8.13 (dd, J = 13.2, 8.6 Hz, 2H), 7.75 (d, J = 1.9 Hz, 1H), 7.66 (dd, J = 8.7, 2.1 Hz, 1H), 7.43 (dd, J = 8.3, 4.2 Hz, 1H), 5.79 (tt, J = 56.4, 4.6 Hz, 1H), 4.04 (t, J = 7.6 Hz, 1H), 3.70 (s, 3H), 2.79 (m, 1H), 2.46-2.30 (m, 1H). ^{19}F NMR (376 MHz, $CDCl_3$) -117.25 (m, 2F). ^{13}C NMR

(101 MHz, $CDCl_3$) δ 172.7, 150.8, 147.7, 136.0, 135.6, 130.5, 129.0, 128.3, 126.8, 121.7, 115.6 (t, J = 239.9 Hz), 52.6, 45.2 (t, J = 5.6 Hz), 37.5 (t, J = 22.2 Hz). HRMS (ESI): m/z $[(M+H)^+]$ calcd for $C_{14}H_{14}F_2NO_2$, 266.0987. found, 266.0991.

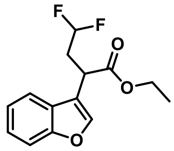

Ethyl 2-(benzofuran-3-yl)-4,4-difluorobutanoate (3j): The compound was according to the general procedure for 48 hours and was purified with silica gel chromatography (petroleum ether / ethyl acetate = 15:1) as white solid (32.2 mg, 60% yield). ^{1}H NMR (400 MHz, $CDCl_3$) δ 7.63 (d, J = 7.6 Hz, 1H), 7.58 (s, 1H), 7.48 (d, J = 7.8 Hz, 1H), 7.31 (t, J = 7.7 Hz, 1H), 7.26 (d, J = 7.6 Hz, 1H), 5.83 (tt, J = 56.4, 4.6 Hz, 1H), 4.24-4.11 (m, 2H), 4.02 (t, J = 7.6 Hz, 1H), 2.80-2.66 (m, 1H), 2.45-2.32 (m, 1H), 1.20 (t, J = 7.1 Hz, 3H). ^{19}F NMR (376 MHz, $CDCl_3$) -117.41 (m, 2F). ^{13}C NMR (101 MHz, $CDCl_3$) δ 171.8, 155.5, 142.3, 126.1, 124.9, 122.9, 120.0, 115.7 (t, J = 240.4 Hz), 111.8, 61.6, 36.1 (t, J = 22.2 Hz), 36.0 (t, J = 6.1 Hz), 14.0. HRMS (ESI): m/z $[(M+Na)^+]$ calcd for $C_{14}H_{14}F_2NaO_3$, 291.0803. found, 291.0800.

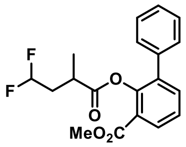

Methyl 2-((4,4-difluoro-2-methylbutanoyl)oxy)-[1,1'-biphenyl]-3-carboxylate (3k): The compound was according to the general procedure with $K_2S_2O_8$ (0.3 mmol) and MeCN/H_2O (2 mL, 3:1) for 48 h and was purified with silica gel chromatography petroleum ether / ethyl acetate = 15:1) as white solid (29.0 mg, 83% yield). ^{1}H NMR (400 MHz, $CDCl_3$) δ 8.01 (dd, J = 7.8, 1.7 Hz, 1H), 7.54 (dd, J = 7.6, 1.7 Hz, 1H), 7.43-7.31 (m, 6H), 5.66 (t, J = 56.0 Hz, 1H), 3.86 (s, 3H), 2.83-2.70 (m, 1H), 2.23-2.09 (m, 1H), 1.78 (m, 1H), 1.10 (d, J = 7.2 Hz, 3H). ^{19}F NMR (376 MHz, $CDCl_3$) -115.63 (m, 2F). ^{13}C NMR (101 MHz, $CDCl_3$) δ 173.1, 164.9, 147.5, 137.1, 136.7, 135.2, 131.0,

129.1, 128.3, 127.9, 126.1, 123.7, 116.0 (t, J = 239.4 Hz), 52.3, 37.2 (t, J = 21.7 Hz), 34.0 (dd, J = 7.1, 8.1 Hz), 16.9. HRMS (ESI): m/z [(M+Na)$^+$] calcd for $C_{19}H_{18}F_2NaO_4$, 371.1065. found, 371.1069.

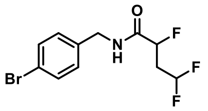

N-(4-Bromobenzyl)-2,4,4-trifluorobutanamide (3l): The compound was according to the general procedure with **1a** (0.2 mmol) and **2l** (0.4 mmol) and was purified with silica gel chromatography (petroleum ether / ethyl acetate = 10:1) as yellowish oil (43.2 mg, 70% yield). ^{1}H NMR (400 MHz, $CDCl_3$) δ 7.48 (d, J = 8.4 Hz, 2H), 7.16 (d, J = 8.2 Hz, 2H), 6.73 (s, 1H), 6.20-5.88 (m, 1H), 5.14 (m, 1H), 4.45 (d, J = 6.0 Hz, 2H), 2.73-2.55 (m, 1H), 2.48-2.30 (m, 1H). ^{19}F NMR (376 MHz, $CDCl_3$) -117.51 (m, 2F), -191.47 (m, 1F). ^{13}C NMR (101 MHz, $CDCl_3$) δ 168.2 (d, J = 19.2 Hz), 136.2, 132.0, 129.5, 121.8, 114.3 (td, J = 240.9, 3.5 Hz), 87.0 (dt, J = 188.9, 6.3 Hz), 42.6, 37.0 (td, J = 23.2, 19.2 Hz). HRMS (ESI): m/z [(M+H)$^+$] calcd for $C_{11}H_{12}{}^{79}BrF_3NO$, 310.0049. found, 310.0052.

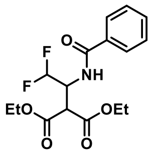

Diethyl 2-(1-benzamido-2,2-difluoroethyl)malonate (3m): The compound was according to the general procedure with 10 mol% PC-5 for 48 hours and was purified with silica gel chromatography petroleum ether / ethyl acetate = 4:1) as white solid (42.1 mg, 61% yield). ^{1}H NMR (400 MHz, $CDCl_3$) δ 7.91 (d, J = 8.7 Hz, 1H), 7.82 (d, J = 7.2 Hz, 2H), 7.54 (t, J = 7.3 Hz, 1H), 7.46 (t, J = 7.5 Hz, 2H), 6.02 (m, 1H), 5.20 (m, 1H), 4.36-4.27 (m, 2H), 4.25-4.17 (m, 2H), 3.94 (d, J = 3.2 Hz, 1H), 1.33 (t, J = 7.1 Hz, 3H), 1.23 (t, J = 7.1 Hz, 3H). ^{19}F NMR (376 MHz, $CDCl_3$) -125.71 (m, 2F). ^{13}C NMR (101 MHz, $CDCl_3$) δ 168.8, 167.0, 166.4, 133.2, 132.1, 128.7, 127.2, 114.1 (t, J = 248.0 Hz), 62.7, 62.4, 50.8 (dd, J = 27.3, 22.2 Hz), 48.0 (t, J = 2.0 Hz), 13.9, 13.9. HRMS (ESI): m/z [(M+H)$^+$] calcd for $C_{16}H_{20}F_2NO_5$, 344.1304. found, 344.1309.

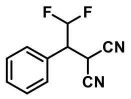

2-(2,2-Difluoro-1-phenylethyl)malononitrile (3n): The compound was according to the general procedure and was purified with silica gel chromatography (petroleum ether / ethyl acetate = 15:1) as colorless liquid (36.3 mg, 88% yield). Known compound.[33] ^{1}H NMR (400 MHz, $CDCl_3$) δ 7.55-7.45 (m, 3H), 7.40 (m, 2H), 6.37-6.07 (m, 1H), 4.30 (d, J = 6.4 Hz, 1H), 3.71-3.61 (m, 1H). ^{19}F NMR (376 MHz, $CDCl_3$) -120.13 (m, 2F). ^{13}C NMR (101 MHz, $CDCl_3$) δ 130.3, 129.8, 128.8, 114.5 (t, J = 248.0 Hz), 110.7, 110.5, 49.7 (dd, J = 22.2, 20.2 Hz), 24.0 (dd, J = 5.6, 4.5 Hz).

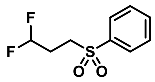

((3,3-Difluoropropyl)sulfonyl)benzene (3o): The compound was according to the general procedure for 48 hours and was purified with silica gel chromatography (petroleum ether / ethyl acetate = 12:1) as white solid (31.7 mg, 72% yield). Known compound.[32] ^{1}H NMR (400 MHz, $CDCl_3$) δ 8.01-7.80 (m, 2H), 7.67 (m, 1H), 7.57 (dd, J = 10.8, 4.6 Hz, 2H), 5.95 (m, 1H), 3.25-3.20 (m, 2H), 2.33-2.21 (m, 2H). ^{19}F NMR (376 MHz, $CDCl_3$) -117.45 (dt, J = 56.4, 16.9 Hz, 2F). ^{13}C NMR (101 MHz, $CDCl_3$) ^{13}C NMR (101 MHz, $CDCl_3$) δ 138.4, 134.2, 129.6, 128.1, 114.6 (t, J = 241.4 Hz), 49.2 (t, J = 5.6 Hz), 27.7 (t, J = 23.2 Hz).

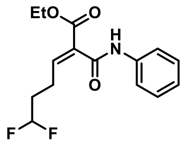

Ethyl 6,6-difluoro-2-(phenylcarbamoyl)hex-2-enoate (3p): The compound was according to the general procedure and was purified with silica gel chromatography (petroleum ether / ethyl acetate = 5:1) as yellowish liquid (30.9 mg, 52% yield). ^{1}H NMR (400 MHz, $CDCl_3$) δ 9.80 (s, 1H), 7.58 (d, J = 7.7 Hz, 2H), 7.33 (m, 3H), 7.11 (t, J = 7.4 Hz, 1H), 5.88 (m, 1H), 4.29 (q, J = 7.1 Hz, 2H), 2.97 (q, J = 7.6 Hz, 2H), 2.10

(m, 2H), 1.35 (t, J = 7.1 Hz, 3H). ^{19}F NMR (376 MHz, $CDCl_3$) -116.03 (dt, J = 56.4, 16.9 Hz, 2F). ^{13}C NMR (101 MHz, $CDCl_3$) δ 166.7, 161.5, 155.1, 137.6, 129.0, 127.5, 124.6, 120.4, 116.7 (t, J = 240.9 Hz), 62.1, 33.2 (t, J = 21.2 Hz), 23.3 (t, J = 5.6 Hz), 14.1. HRMS (ESI): m/z [$(M+H)^+$] calcd for $C_{15}H_{18}F_2NO_3$, 298.1249. found, 298.1253.

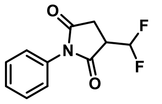

3-(Difluoromethyl)-1-phenylpyrrolidine-2,5-dione (3q): The compound was according to the general procedure for 48 hours and was purified with silica gel chromatography (petroleum ether / ethyl acetate = 8:1) as white solid (35.5 mg, 79% yield). Known compound.[32] ^{1}H NMR (400 MHz, $CDCl_3$) δ 7.57-7.44 (m, 2H), 7.44-7.35 (m, 1H), 7.32-7.14 (m, 2H), 6.49-6.20 (m, 1H), 3.48 (m, 1H), 3.09 (dd, J = 18.6, 5.0 Hz, 1H), 2.95 (dd, J = 18.6, 9.7 Hz, 1H). ^{19}F NMR (376 MHz, $CDCl_3$) -125.02 (m, 2F). ^{13}C NMR (101 MHz, $CDCl_3$) δ 174.0, 172.1 (d, J = 14.1 Hz), 131.2, 129.4, 129.1, 126.4, 113.6 (t, J = 243.9 Hz), 44.7 (t, J = 23.7 Hz), 27.4 (dd, J = 5.0, 1.0 Hz).

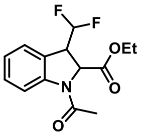

Ethyl 1-acetyl-3-(difluoromethyl)indoline-2-carboxylate (3r): The compound was according to the general procedure with 10 mol% PC-5 for 60 hours and was purified with silica gel chromatography (petroleum ether / ethyl acetate = 8:1) as white solid (26.6 mg, 47% yield). ^{1}H NMR (400 MHz, DMSO-d6, 363 K) δ 7.89 (s, 1H), 7.46-7.22 (m, 2H), 7.10 (td, J = 7.5, 0.8 Hz, 1H), 6.27 (td, J = 55.7, 4.0 Hz, 1H), 5.14 (d, J = 2.6 Hz, 1H), 4.34-4.14 (m, 2H), 3.95 (t, J = 14.7 Hz, 1H), 2.24 (s, 3H), 1.23 (t, J = 7.1 Hz, 3H). ^{19}F NMR (376 MHz, DMSO-d6) -122.54 (m, 2F). ^{13}C NMR (101 MHz, DMSO-d6) δ 169.7, 168.8 (168.4), 143.3 (142.1), 130.04 (129.9), 127.1 (125.3), 124.3 (123.7), 117.7 (114.0), 115.2 (t, J = 247.5 Hz), 62.7 (62.0), 61.6 (t, J = 3.0 Hz) (60.6 (t, J = 3.0 Hz)), 49.7 (t, J = 22.7 Hz) (47.5(t, J = 22.7 Hz)), 24.5 (23.7), 14.1. HRMS (ESI): m/z [$(M+H)^+$] calcd for $C_{14}H_{16}F_2NO_3$, 284.1093. found, 284.1097.

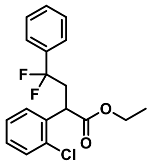

Ethyl 2-(2-chlorophenyl)-4,4-difluoro-4-phenylbutanoate (3s): The compound was according to the general procedure and was purified with silica gel chromatography (petroleum ether / ethyl acetate = 20:1) as yellowish liquid (55.8 mg, 83% yield). ^{1}H NMR (400 MHz, $CDCl_3$) δ 7.53-7.44 (m, 2H), 7.44-7.27 (m, 7H), 7.18 (m, 2H), 4.49 (dd, J = 8.6, 4.3 Hz, 1H), 4.18-4.01 (m, 2H), 3.23-3.08 (m, 1H), 2.52-2.37 (m, 1H), 1.18 (t, J = 7.1 Hz, 3H). ^{19}F NMR (376 MHz, $CDCl_3$) -95.97 (m, 2F). ^{13}C NMR (101 MHz, $CDCl_3$) δ 172.2, 136.6 (t, J = 26.3Hz), 136.2, 133.6, 129.9, 129.9 (t, J = 2.0 Hz), 128.8, 128.6, 128.4, 127.1, 125.0 (t, J = 6.6 Hz), 121.9 (t, J = 244.9 Hz), 61.3, 42.1 (t, J = 3.0 Hz), 41.9 (t, J = 27.8 Hz), 14.0. HRMS (ESI): m/z [$(M+Na)^+$] calcd for $C_{18}H_{17}{}^{35}ClF_2NaO_2$, 361.0777. found, 361.0780.

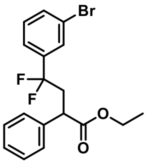

Ethyl 4-(3-bromophenyl)-4,4-difluoro-2-phenylbutanoate (3t): The compound was according to the general procedure and was purified with silica gel chromatography (petroleum ether / ethyl acetate = 20:1) as colorless liquid (60.1 mg, 79% yield). ^{1}H NMR (400 MHz, $CDCl_3$) δ 7.60 (s, 1H), 7.53 (d, J = 8.1 Hz, 1H), 7.39 (d, J = 8.2 Hz, 1H), 7.34-7.18 (m, 6H), 4.15-3.95 (m, 2H), 3.95-3.86 (m, 1H), 3.16 (m, 1H), 2.52-2.35 (m, 1H), 1.18 (t, J = 7.1 Hz, 3H). ^{19}F NMR (376 MHz, $CDCl_3$) -96.16 (m, 2F). ^{13}C NMR (101 MHz, $CDCl_3$) δ 172.7, 138.8 (t, J = 27.8 Hz), 138.2, 133.1, 130.1, 128.9, 128.3 (t, J = 6.6 Hz), 127.7, 123.8 (t, J = 6.1 Hz), 122.6, 121.1 (t, J = 244.9 Hz), 61.3, 45.6 (t, J = 3.0 Hz), 42.6 (t, J = 27.3 Hz), 14.0. HRMS (ESI): m/z [$(M+Na)^+$] calcd for $C_{18}H_{17}{}^{79}BrF_2NaO_2$, 405.0272. found, 405.0270.

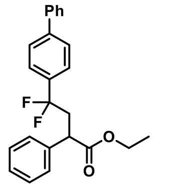

Ethyl 4-([1,1'-biphenyl]-4-yl)-4,4-difluoro-2-phenylbutanoate (3u): The compound was according to the general procedure and was purified with silica gel chromatography (petroleum ether / ethyl acetate = 20:1) as white solid (60.8 mg, 80% yield). ^{1}H NMR (400 MHz, $CDCl_3$) δ 7.61 (m, 6H), 7.47 (t, J = 7.5 Hz, 2H), 7.39 (t, J = 7.3 Hz, 1H), 7.36-7.21 (m, 5H), 4.17-4.02 (m, 2H), 4.00 (dd J = 9.8, 3.3 Hz, 1H), 3.36-3.20 (m, 1H), 2.59-2.44 (m, 1H), 1.20 (t, J = 7.1 Hz, 3H). ^{19}F NMR (376 MHz, $CDCl_3$) -96.16 (q, J = 17.5 Hz, 2F). ^{13}C NMR (101 MHz, $CDCl_3$) δ 172.9, 142.9, 140.2, 138.5, 135.6 (t, J = 26.8 Hz), 128.9, 128.8, 127.8, 127.7, 127.5, 127.2, 127.2, 125.5 (t, J =6.1 Hz), 122.0 (t, J = 244.4 Hz), 61.2, 45.8 (t, J = 3.0 Hz), 42.8 (t, J = 27.8 Hz), 14.0. HRMS (ESI): m/z [$(M+Na)^+$] calcd for $C_{24}H_{22}F_2NaO_2$, 403.1480. found, 403.1481.

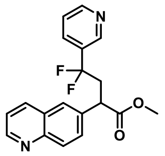

Methyl 4,4-difluoro-4-(pyridin-3-yl)-2-(quinolin-6-yl)butanoate (3v): The compound was according to the general procedure and was purified with silica gel chromatography (petroleum ether / ethyl acetate = 2:1) as yellow solid (51.3 mg, 75% yield). ^{1}H NMR (400 MHz, $CDCl_3$) δ 8.83-8.77 (m, 1H), 8.68 (s, 1H), 8.56 (d, J = 4.6 Hz, 1H), 8.00 (dd, J = 12.0, 8.8 Hz, 2H), 7.68 (d, J = 7.9 Hz, 1H), 7.63 (s, 1H), 7.56 (dd, J = 8.7, 1.8 Hz, 1H), 7.30 (dd, J = 8.3, 4.2 Hz, 1H), 7.24-7.18 (m, 1H), 4.10 (dd, J = 9.0, 4.2 Hz, 1H), 3.54 (s, 3H), 3.29-3.16 (m, 1H), 2.60-2.47 (m, 1H). ^{19}F NMR (376 MHz, $CDCl_3$) δ -96.31 (m, 2F). ^{13}C NMR (101 MHz, $CDCl_3$) δ 172.7, 151.3, 150.7, 147.6, 146.5 (t, J = 7.1 Hz), 136.0, 135.9, 132.8 (t, J = 6.1 Hz), 132.2 (t, J = 26.8 Hz), 130.3, 129.0, 128.2, 126.6, 123.2, 121.6, 121.0 (t, J = 244.9 Hz), 52.6, 45.3 (t, J = 3.0

Hz), 42.5 (t, J = 27.3 Hz). HRMS (ESI): m/z [(M+H)$^+$] calcd for $C_{19}H_{17}F_2N_2O_2$, 343.1253. found, 343.1255.

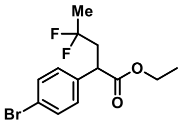

Ethyl 2-(4-bromophenyl)-4,4-difluoropentanoate (3w): The compound was according to the general procedure and was purified with silica gel chromatography (petroleum ether / ethyl acetate = 20:1) as colorless liquid (57.6 mg, 90% yield). ^{1}H NMR (400 MHz, $CDCl_3$) δ 7.45 (d, J = 8.5 Hz, 2H), 7.20 (d, J = 8.4 Hz, 2H), 4.24-4.05 (m, 2H), 3.87 (dd, J = 9.2, 4.4 Hz, 1H), 2.87 (m, 1H), 2.16 (m, 1H), 1.58 (t, J = 18.5 Hz, 3H), 1.20 (t, J = 7.1 Hz, 3H). ^{19}F NMR (376 MHz, $CDCl_3$) -90.99 (m, 2F). ^{13}C NMR (101 MHz, $CDCl_3$) δ 172.7, 137.6, 131.9, 129.4, 122.9 (t, J = 239.9 Hz), 121.6, 61.4, 45.1 (t, J = 3.5 Hz), 41.3 (t, J = 25.2 Hz), 23.9 (t, J = 27.3 Hz), 14.0. HRMS (ESI): m/z [(M+Na)$^+$] calcd for $C_{13}H_{15}{}^{79}BrF_2NaO_2$, 343.0116. found, 343.0115.

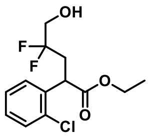

Ethyl 2-(2-chlorophenyl)-4,4-difluoro-5-hydroxypentanoate (3x): The compound was according to the general procedure with for 48 hours and was purified with silica gel chromatography (petroleum ether / ethyl acetate = 8:1) as colorless liquid (23.9 mg, 41% yield). ^{1}H NMR (400 MHz, $CDCl_3$) δ 7.41 (m, 1H), 7.37-7.30 (m, 1H), 7.28-7.20 (m, 2H), 4.46 (dd, J = 8.9, 4.2 Hz, 1H), 4.26-4.05 (m, 2H), 3.78-3.65 (m, 2H), 2.97 (m, 1H), 2.66-2.30 (m, 1H), 2.32-2.18 (m, 1H), 1.20 (t, J = 7.1 Hz, 3H). ^{19}F NMR (376 MHz, $CDCl_3$) -107.33 (m, 2F). ^{13}C NMR (101 MHz, $CDCl_3$) δ 173.0, 136.4, 133.6, 130.0, 128.8, 128.7, 127.3, 122.2 (t, J = 243.9 Hz), 64.0 (t, J = 31.8 Hz), 61.7, 41.7 (t, J = 4.0 Hz), 36.0 (t, J = 24.2 Hz), 14.0. HRMS (ESI): m/z [(M+Na)$^+$] calcd for $C_{13}H_{15}{}^{35}ClF_2NaO_3$, 315.0570. found, 315.0572.

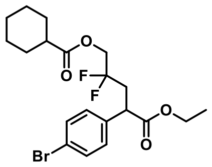

4-(4-Bromophenyl)-5-ethoxy-2,2-difluoro-5-oxopentyl cyclohexanecarboxylate (3y): The compound was according to the general procedure and was purified with silica gel chromatography (petroleum ether / ethyl acetate = 10:1) as colorless liquid (71.4 mg, 80% yield). ^{1}H NMR (400 MHz, $CDCl_3$) δ 7.46 (d, J = 8.4 Hz, 2H), 7.20 (d, J = 8.4 Hz, 2H), 4.26-4.04 (m, 4H), 3.90 (dd, J = 8.9, 4.6 Hz, 1H), 2.91 (m, 1H), 2.36 (m, 1H), 2.30-2.15 (m, 1H), 1.90 (d, J = 12.1 Hz, 2H), 1.79-1.71 (m, 2H), 1.69-1.61 (m, 1H), 1.44 (dd, J = 23.2, 11.6 Hz, 2H), 1.24 (m, 6H). ^{19}F NMR (376 MHz, $CDCl_3$) -105.32 (m, 2F). ^{13}C NMR (101 MHz, $CDCl_3$) δ 174.8, 172.3, 137.2, 132.0, 129.4, 121.8, 120.7 (t, J = 243.9 Hz), 63.5 (t, J = 33.3 Hz), 61.5, 44.3 (t, J = 3.5 Hz), 42.8, 37.4 (t, J = 22.7 Hz), 28.8, 25.6, 25.3, 14.0. HRMS (ESI): m/z [$(M+Na)^+$] calcd for $C_{20}H_{25}{}^{79}BrF_2NaO_4$, 469.0796. found, 469.0795.

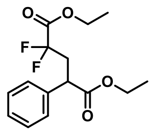

Diethyl 2,2-difluoro-4-phenylpentanedioate (3z): The compound was according to the general procedure and was purified with silica gel chromatography (petroleum ether / ethyl acetate = 15:1) as colorless liquid (50.9 mg, 85% yield). Known compound.[34] ^{1}H NMR (400 MHz, $CDCl_3$) δ 7.37-7.18 (m, 5H), 4.23-4.02 (m, 4H), 3.89 (dd, J = 8.6, 5.2 Hz, 1H), 3.12-2.96 (m, 1H), 2.48 (m, 1H), 1.27 (t, J = 7.2 Hz, 3H), 1.17 (t, J = 7.1 Hz, 3H). ^{19}F NMR (376 MHz, $CDCl_3$) -105.38 (m, 2F). ^{13}C NMR (101 MHz, $CDCl_3$) δ 172.4, 163.6 (t, J = 32.3 Hz), 137.6, 128.9, 127.8, 115.0 (t, J = 2.5 Hz), 63.0, 61.4, 44.9 (t, J = 4.0 Hz), 38.0 (t, J = 23.7 Hz), 14.0, 13.8.

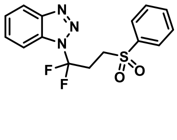

1-(1,1-Difluoro-3-(phenylsulfonyl)propyl)-1H-benzo[d][1,2,3]triazole (3aa): The compound was according to the general procedure with for 48 hours and was purified with silica gel chromatography (petroleum ether / ethyl acetate = 5:1) as white solid (47.8 mg, 71% yield). ^{1}H NMR (400 MHz, $CDCl_3$) δ 8.07 (d, J = 8.4 Hz, 1H), 7.97 (d, J = 8.3 Hz, 2H), 7.75 (d, J = 8.4 Hz, 1H), 7.70 (t, J = 7.4 Hz, 1H), 7.59 (m, 3H), 7.48-7.42 (m, 1H), 3.57-3.52 (m, 2H), 3.40-3.30 (m, 2H). ^{19}F NMR (376 MHz, $CDCl_3$) δ -77.2 (t, J = 26.3 Hz, 2F). ^{13}C NMR (101 MHz, $CDCl_3$) δ 146.2, 138.2, 134.4, 130.8, 129.7, 129.6, 128.2, 125.6, 120.4, 120.3 (t, J = 253.0 Hz), 111.4 (t, J = 3.0 Hz), 49.9 (t, J = 3.5 Hz), 29.7 (t, J = 26.3 Hz). HRMS (ESI): m/z [$(M+H)^+$] calcd for $C_{15}H_{14}F_2N_3O_2S$ 338.0769. found, 338.0770.

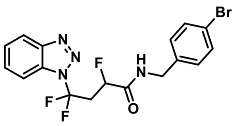

4-(1H-benzo[d][1,2,3]triazol-1-yl)-N-(4-bromobenzyl)-2,4,4-trifluorobutanamide (3ab): The compound was according to the general procedure with 10 mol% PC-5, **1q** (0.2 mmol) and **2l** (0.4 mmol) and was purified with silica gel chromatography (petroleum ether / ethyl acetate = 4:1) as white solid (57.8 mg, 68% yield). ^{1}H NMR (400 MHz, $CDCl_3$) δ 8.08 (d, J = 8.4 Hz, 1H), 7.79 (d, J = 8.3 Hz, 1H), 7.58 (t, J = 7.7 Hz, 1H), 7.45 (t, J = 7.8 Hz, 3H), 7.15 (d, J = 8.2 Hz, 2H), 6.89 (s, 1H), 5.45 (dd, J = 49.8, 9.4 Hz, 1H), 4.44 (d, J = 6.0 Hz, 2H), 3.79 (m), 3.44 (m, 1H). ^{19}F NMR (376 MHz, $CDCl_3$) δ -77.04 (m, 2F), -190.84 (m, 1F). ^{13}C NMR (101 MHz, $CDCl_3$) δ 167.6 (d, J = 19.2 Hz), 146.2, 136.3, 132.0, 130.9, 129.6, 129.5, 125.4, 121.8, 120.4, 120.3 (t, J = 255.5 Hz), 111.5 (t, J = 3.0 Hz), 86.3 (dt, J = 191.9, 3.0 Hz), 42.8, 38.2 (td, J = 25.0, 19.2 Hz). HRMS (ESI): m/z [$(M+H)^+$] calcd for $C_{17}H_{15}{}^{79}BrF_3N_4O$, 338.0769. found, 338.0770.

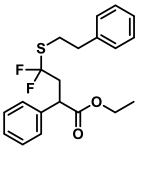

Ethyl 4,4-difluoro-4-(phenethylthio)-2-phenylbutanoate (3ac): The compound was according to the general procedure with 10 mol% PC-5 for 48 hours and was purified with silica gel chromatography (petroleum ether / ethyl acetate = 15:1) as white solid (39.3 mg, 64% yield). ^1H NMR (400 MHz, $CDCl_3$) δ 7.36-7.27 (m, 7H), 7.25-7.17 (m, 3H), 4.20-4.05 (m, 2H), 3.94 (dd, J = 9.4, 4.0 Hz, 1H), 3.18 (m, 1H), 3.11-2.99 (m, 2H), 3.00-2.90 (m, 2H), 2.45 (qd, J = 14.9, 4.0 Hz, 1H), 1.20 (t, J = 7.1 Hz, 3H). ^{19}F NMR (376 MHz, $CDCl_3$) δ -73.14 (td, J = 15.0, 7.5 Hz, 2F). ^{13}C NMR (101 MHz, $CDCl_3$) δ 172.6, 139.8, 138.1, 130.2 (t, J = 278.8 Hz), 128.9, 128.5, 127.7, 126.6, 126.1, 61.3, 46.2 (t, J = 3.0 Hz), 42.6 (t, J = 23.7 Hz), 36.5, 29.5 (t, J = 3.0 Hz), 14.0. HRMS (ESI): m/z [$(M+Na)^+$] calcd for $C_{20}H_{22}F_2NaO_2S$, 387.1201. found, 387.1206.

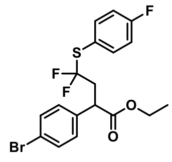

Ethyl 2-(4-bromophenyl)-4,4-difluoro-4-((4-fluorophenyl)thio)butanoate (3ad): The compound was according to the general procedure with 10 mol% PC-5 and was purified with silica gel chromatography (petroleum ether / ethyl acetate = 10:1) as white solid (62.2 mg, 72% yield). ^1H NMR (400 MHz, $CDCl_3$) δ 7.53 (dd, J = 8.4, 5.4 Hz, 2H), 7.43 (d, J = 8.4 Hz, 2H), 7.16 (d, J = 8.3 Hz, 2H), 7.04 (t, J = 8.6 Hz, 2H), 4.18-4.04 (m, 2H), 3.92 (dd, J = 8.6, 4.7 Hz, 1H), 3.17-3.05 (m, 1H), 2.42 (dt, J = 14.8, 10.9 Hz, 1H), 1.18 (t, J = 7.1 Hz, 3H). ^{19}F NMR (376 MHz, $CDCl_3$) δ -73.17 (m, 2F), δ -110.23 (m, 1F). ^{13}C NMR (101 MHz, $CDCl_3$) δ 172.0, 164 (d, J = 252.5 Hz), 138.5 (d, J = 9.1 Hz), 136.8, 132.0, 129.4, 128.3 (td, J = 280.8, 2.0 Hz), 121.8, 121.5 (d, J = 3.0 Hz), 116.3 (d, J = 22.2 Hz), 61.5, 45.5 (t, J = 3.0 Hz), 41.7 (t, J = 23.7 Hz), 13.9. HRMS (ESI): m/z [$(M+Na)^+$] calcd for $C_{18}H_{16}{}^{79}BrF_3NaO_2S$, 454.9899. found, 454.9895.

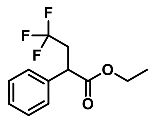

Ethyl 4,4,4-trifluoro-2-phenylbutanoate (3ae): The compound was according to the general procedure with **1ba** (0.6 mmol) and 2,4,6-collidine (0.8 mmol) and was purified with silica gel chromatography (petroleum ether / ethyl acetate = 20:1) as colorless liquid (44.7 mg, 91% yield). Known compound.[52] ^{1}H NMR (400 MHz, $CDCl_3$) ^{1}H NMR (400 MHz, $CDCl_3$) δ 7.43-7.23 (m, 5H), 4.23-4.04 (m, 2H), 3.88 (dd, J = 9.0, 4.9 Hz, 1H), 3.18-3.04 (m, 1H), 2.52-2.38 (m, 1H), 1.19 (t, J = 7.1 Hz, 3H). ^{19}F NMR (376 MHz, $CDCl_3$) δ -65.42 (t, J = 9.4 Hz, 3F). ^{13}C NMR (101 MHz, $CDCl_3$) δ 171.9, 137.2, 129.0, 128.0, 127.6, 126.1 (q, J = 278.4 Hz), 61.5, 45.4 (q, J = 2.7 Hz), 37.3 (q, J = 29.0 Hz), 13.9.

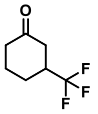

3-(Trifluoromethyl)cyclohexan-1-one (3af): The compound was according to the general procedure with **1ba** (0.6 mmol) and 2,4,6-collidine (0.8 mmol) and was purified with silica gel chromatography petroleum ether / ethyl acetate = 30:1) as colorless liquid (25.5 mg, 77% yield). Known compound.[38] ^{1}H NMR (400 MHz, $CDCl_3$) δ 2.59 (d, J = 14.1 Hz, 1H), 2.54-2.21 (m, 4H), 2.21-2.04 (m, 2H), 1.74-1.61 (m, 2H). ^{19}F NMR (376 MHz, $CDCl_3$) δ -73.84 (d, J = 7.5 Hz, 3F). ^{13}C NMR (101 MHz, $CDCl_3$) δ 207.2, 126.4 (q, J = 280.1 Hz), 42.2 (q, J = 28.3 Hz), 40.6, 39.9 (q, J = 1.8 Hz), 23.6.

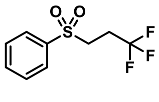

((3,3,3-Trifluoropropyl)sulfonyl)benzene (3ag): The compound was according to the general procedure with **1ba** (0.6 mmol) and 2,4,6-collidine (0.8 mmol) for 48 hours at 50 °C and was purified with silica gel chromatography (petroleum ether / ethyl acetate = 20:1) as colorless liquid (44.3 mg, 93% yield). Known compound.[39] ^{1}H NMR (400 MHz, $CDCl_3$) δ 7.92 (d, J = 7.6 Hz, 2H), 7.70 (t, J = 7.4 Hz, 1H), 7.60 (t, J = 7.5 Hz, 2H), 3.30-3.25 (m, 2H), 2.56 (m, 2H). ^{19}F NMR (376 MHz, $CDCl_3$) δ -66.01 (t, J = 9.4 Hz, 3F). ^{13}C NMR (101 MHz, $CDCl_3$) δ 138.1, 134.4, 129.6, 128.0, 125.4 (q, J = 277.8 Hz), 49.3, 28.0 (q, J = 31.6 Hz).

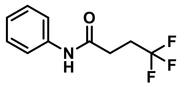

4,4,4-Trifluoro-N-phenylbutanamide (3ah): The compound was according to the general procedure with 10 mol% PC-5, **1ba** (0.6 mmol) and 2,4,6-collidine (0.8 mmol) and was purified with silica gel chromatography (petroleum ether / ethyl acetate = 10:1) as white solid (34.2 mg, 79% yield). Known compound.[39] ^{1}H NMR (400 MHz, $CDCl_3$) δ 7.91 (s, 1H), 7.44 (d, J = 7.9 Hz, 2H), 7.28 (t, J = 7.4 Hz, 2H), 7.11 (t, J = 7.3 Hz, 1H), 2.59-2.46 (m, 4H). ^{19}F NMR (376 MHz, $CDCl_3$) δ -66.77 (t, J = 9.4 Hz, 3F). ^{13}C NMR (101 MHz, $CDCl_3$) δ 168.4, 137.4, 129.0, 126.7 (q, J = 277.4 Hz), 124.8, 120.5, 29.6, 29.4 (q, J = 30.0 Hz).

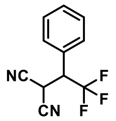

2-(2,2,2-Trifluoro-1-phenylethyl)malononitrile (3ai): The compound was according to the general procedure with 10 mol% PC-5, **1ba** (0.6 mmol) and 2,4,6-collidine (0.8 mmol) and was purified with silica gel chromatography (petroleum ether / ethyl acetate = 15:1) as colorless liquid (37.6 mg, 84% yield). Known compound.[40] ^{1}H NMR (400 MHz, $CDCl_3$) δ 7.50 (dd, J = 14.6, 8.3 Hz, 5H), 4.38 (d, J = 6.4 Hz, 1H), 3.93 (p, J = 7.5 Hz, 1H). ^{19}F NMR (376 MHz, $CDCl_3$) δ -66.86 (t, J = 7.5 Hz, 3F). ^{13}C NMR (101 MHz, $CDCl_3$) δ 130.7, 129.7, 128.9, 127.9, 124.0 (q, J = 282.8 Hz), 110.0, 109.7, 50.3 (q, J = 29.3 Hz), 24.8 (q, J = 2.7 Hz).

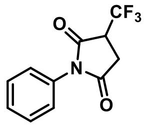

1-Phenyl-3-(trifluoromethyl)pyrrolidine-2,5-dione (3aj): The compound was according to the general procedure with **1ba** (0.6 mmol) and 2,4,6-collidine (0.8 mmol) and was purified with silica gel chromatography (petroleum ether / ethyl acetate = 15:1) as white solid (30.1 mg, 62% yield). Known compound.[53] ^{1}H NMR (400 MHz, $CDCl_3$) δ 7.54-7.36 (m, 3H), 7.23 (d, J = 7.1 Hz, 2H), 3.65 (m, 1H), 3.00 (m, 2H). ^{19}F NMR

(376 MHz, $CDCl_3$) δ -68.77 (d, J = 7.5 Hz, 3F).^{13}C NMR (101 MHz, $CDCl_3$) δ 172.6, 168.8 (q, *J* = 5.1 Hz), 131.1, 129.4, 129.3, 126.4, 123.8 (q, *J* = 280.1 Hz), 44.5 (q, *J* = 30.0 Hz), 29.6 (q, *J* = 1.0 Hz).

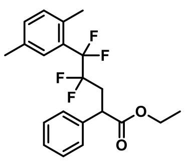

Ethyl 5-(2,5-dimethylphenyl)-4,4,5,5-tetrafluoro-2-phenylpentanoate (3ak): The compound was according to the general procedure with **1bb** (0.6 mmol) and 2,4,6-collidine (0.8 mmol) and was purified with silica gel chromatography (petroleum ether / ethyl acetate = 20:1) as light-yellow oil (67.1 mg, 88% yield). ^{1}H NMR (400 MHz, $CDCl_3$) δ 7.37-7.24 (m, 6H), 7.14 (d, J = 7.8 Hz, 1H), 7.08 (d, J = 7.8 Hz, 1H), 4.12 (m, 2H), 3.99 (dd, J = 9.6, 4.0 Hz, 1H), 3.24 (m, 1H), 2.58-2.38 (m, 4H), 2.30 (s, 3H), 1.17 (t, J = 7.1 Hz, 3H). ^{19}F NMR (376 MHz, $CDCl_3$) δ -106.98 (m, 2F), -112.96 (m, 2F). ^{13}C NMR (101 MHz, $CDCl_3$) δ 172.6, 138.3, 135.2, 134.7 (t, J = 2.0 Hz), 132.2, 131.7, 128.9, 128.8 (t, J = 11.1 Hz), 128.1 (t, J = 23.2 Hz), 127.8, 127.7, 118.9 (tt, J = 252.5, 38.9 Hz), 118.0 (tt, J = 253.5, 35.9 Hz), 61.3, 44.5, 34.3 (t, J = 22.2 Hz), 20.9, 20.3 (t, J = 3.0 Hz), 14.0. HRMS (ESI): m/z [$(M+Na)^+$] calcd for $C_{21}H_{22}F_4NaO_2$, 405.1448. found, 405.1454.

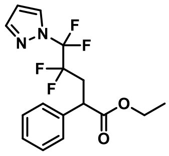

Ethyl 4,4,5,5-tetrafluoro-2-phenyl-5-(1H-pyrazol-1-yl)pentanoate (3al): The compound was according to the general procedure with 10 mol% PC-5, **1bc** (0.6 mmol) and 2,4,6-collidine (0.8 mmol) for 48 h and was purified with silica gel chromatography (petroleum ether / ethyl acetate = 10:1) as colorless oil (48.2 mg, 70% yield). ^{1}H NMR (400 MHz, $CDCl_3$) δ 7.79-7.70 (m, 2H), 7.33-7.26 (m, 5H), 6.42 (s, 1H), 4.17-4.05 (m, 2H), 3.96 (dd, J = 9.6, 4.0 Hz, 1H), 3.24 (m, 1H), 2.49 (m, 1H), 1.17 (t, J = 7.1 Hz, 3H). ^{19}F NMR (376 MHz, $CDCl_3$) δ -98.61 (d, J = 11.3Hz, 2F) -114.60 (m, 2F). ^{13}C NMR (101 MHz, $CDCl_3$) δ 172.2, 143.1, 137.7, 129.1, 128.9, 127.8, 127.6, 117.2 (tt, J = 256.0,

37.9 Hz), 113.6 (tt, J = 266.6, 33.8 Hz), 108.1, 61.4, 44.2 (t, J = 2.0 Hz), 34.6 (t, J = 21.2 Hz), 13.9. HRMS (ESI): m/z [$(M+Na)^+$] calcd for $C_{16}H_{16}F_4N_2NaO_2$, 367.1040. found, 367.1041.

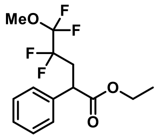

Ethyl 4,4,5,5-tetrafluoro-5-methoxy-2-phenylpentanoate (3am): The compound was according to the general procedure with **1bd** (0.6 mmol) and 2,4,6-collidine (0.8 mmol) and was purified with silica gel chromatography (petroleum ether / ethyl acetate = 20:1) as colorless oil (52.4 mg, 85% yield). ^{1}H NMR (400 MHz, $CDCl_3$) δ 7.41-7.15 (m, 5H), 4.13 (m, 2H), 3.97 (dd, J = 9.5, 3.8 Hz, 1H), 3.63 (s, 3H), 3.08 (m, 1H), 2.40-2.22 (m, 1H), 1.20 (td, J = 7.1, 0.9 Hz, 3H). ^{19}F NMR (376 MHz, $CDCl_3$) δ -94.45 (m, 2F), -117.07 (m, 2F). ^{13}C NMR (101 MHz, $CDCl_3$) δ 172.5, 138.1, 128.9, 127.7, 127.6, 118.6 (tt, J = 271.7, 32.3 Hz), 116.2 (tt, J = 252.5, 36.9 Hz), 61.3, 50.9 (t, J = 7.1 Hz), 44.3 (t, J = 2.5 Hz), 34.8 (t, J = 21.7 Hz), 13.9. HRMS (ESI): m/z [$(M+Na)^+$] calcd for $C_{14}H_{16}F_4NaO_3$, 331.0928. found, 331.0932.

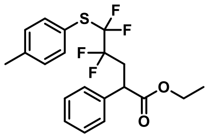

Ethyl 4,4,5,5-tetrafluoro-2-phenyl-5-(p-tolylthio)pentanoate (3an): The compound was according to the general procedure with 10 mol% PC-5, **1be** (0.6 mmol) and 2,4,6-collidine (0.8 mmol) and was purified with silica gel chromatography (petroleum ether / ethyl acetate = 20:1) as colorless oil (58.4 mg, 73% yield). ^{1}H NMR (400 MHz, $CDCl_3$) δ 7.50 (d, J = 8.1 Hz, 2H), 7.35-7.24 (m, 5H), 7.17 (d, J = 7.9 Hz, 2H), 4.13 (m, 2H), 3.99 (dd, J = 9.6, 3.9 Hz, 1H), 3.28-3.11 (m, 1H), 2.49-2.33 (m, 4H), 1.18 (t, J = 7.1 Hz, 3H). ^{19}F NMR (376 MHz, $CDCl_3$) δ -89.01 (m, 2F), -111.51 (m, 2F). ^{13}C NMR (101 MHz, $CDCl_3$) δ 172.4, 141.0, 138.0, 137.2, 130.1, 129.0, 127.8, 127.7, 124.2 (tt, J = 287.9, 35.4 Hz), 120.2, 118.4 (tt, J = 255.5, 32.8 Hz), 61.4, 44.4 (t, J = 3.0 Hz), 34.0 (t,

J = 21.7 Hz), 21.3, 14.0. HRMS (ESI): m/z [(M+Na)$^+$] calcd for $C_{20}H_{20}F_4NaO_2S$, 423.1012. found, 423.1013.

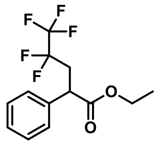

Ethyl 4,4,5,5,5-pentafluoro-2-phenylpentanoate (3ao): The compound was according to the general procedure with **1bf** (0.6 mmol) and 2,4,6-collidine (0.8 mmol) and was purified with silica gel chromatography (petroleum ether / ethyl acetate = 20:1) as colorless oil (48.0 mg, 81% yield). ^{1}H NMR (400 MHz, $CDCl_3$) δ 7.40-7.27 (m, 5H), 4.22-4.06 (m, 2H), 3.98 (dd, J = 9.3, 4.1 Hz, 1H), 3.22-3.06 (m, 1H), 2.44-2.30 (m, 1H), 1.20 (t, J = 7.1 Hz, 3H). ^{19}F NMR (376 MHz, $CDCl_3$) δ -89.01 (m, 2F), -111.51 (m, 2F). ^{19}F NMR (376 MHz, $CDCl_3$) δ -85,73 (s, 3F), -117.74 (m, 2F). ^{13}C NMR (101 MHz, $CDCl_3$) δ 172.0, 137.5, 129.1, 128.0, 127.5, 118.9 (qt, J = 286.8, 35.9 Hz), 115.0 (tq, J = 254.5, 38.0 Hz), 61.6, 43.9, 34.2 (t, J = 21.2 Hz), 13.9. HRMS (ESI): m/z [(M+Na)$^+$] calcd for $C_{13}H_{13}F_5NaO_2$, 319.0728. found, 319.0735.

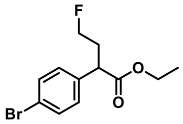

Ethyl 2-(4-bromophenyl)-4-fluorobutanoate (3ap): The compound was according to the general procedure for 48 hours and was purified with silica gel chromatography (petroleum ether / ethyl acetate = 25:1) as colorless liquid (46.6 mg, 81% yield). ^{1}H NMR (400 MHz, $CDCl_3$) δ 7.44 (d, J = 8.4 Hz, 2H), 7.16 (d, J = 8.4 Hz, 2H), 4.49 - 4.23 (m, 2H), 4.11 (m, 2H), 3.74 (t, J = 7.7 Hz, 1H), 2.53-2.39 (m, 1H), 2.10-1.96 (m, 1H), 1.18 (t, J = 7.1 Hz, 3H). ^{19}F NMR (376 MHz, $CDCl_3$) -221.75 (m, 1F). ^{13}C NMR (101 MHz, $CDCl_3$) δ 172.9, 137.2, 131.9, 129.7, 121.5, 81.3 (d, J = 165.6 Hz), 61.2, 46.7 (d, J = 4.0 Hz), 33.9 (d, J = 20.2 Hz), 14.1. HRMS (ESI): m/z [(M+Na)$^+$] calcd for $C_{12}H_{14}{}^{79}BrFNaO_2$, 311.0053. found, 311.0052.

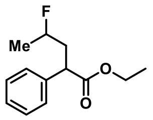

Ethyl 4-fluoro-2-phenylpentanoate (3aq): The compound was according to the general procedure was purified with silica gel chromatography (petroleum ether / ethyl acetate = 25:1) as colorless liquid (36.8 mg, 82% yield). ^{1}H NMR (400 MHz, $CDCl_3$) δ 7.37-7.22 (m, 5H), 4.79-4.58 (m, 0.36H), 4.51-4.30 (m, 0.65H), 4.21-4.04 (m, 2H), 3.83 (m, 1H), 2.46-2.29 (m, 1H), 2.06 (m, 0.36H), 2.01-1.91 (m, 0.65H), 1.32 (td, J = 24.2, 6.2 Hz, 3H), 1.19 (m, 3H). ^{19}F NMR (376 MHz, $CDCl_3$) -175.6 (m, 0.36 F), -176.42 (m, 0.65 F). ^{13}C NMR (101 MHz, $CDCl_3$) δ 173.7 (173.5), 139.1 (138.2), 128.8, 128.2, 127.4 (127.3), 88.2 (d, J = 166.7 Hz) (89.1 (d, J = 165.6 Hz)), 61.0 (60.9), 47.8 (d, J = 3.0 Hz) (47.4 (d, J = 3.0 Hz)), 40.4 (d, J = 20.2 Hz) (41.0 (d, J = 21.2 Hz)), 21.2 (d, J = 22.2 Hz) (21.2 (d, J = 21.2 Hz)), 14.1 (14.1). HRMS (ESI): m/z [$(M+Na)^+$] calcd for $C_{13}H_{17}FNaO_2$, 247.1105. found, 247.1109.

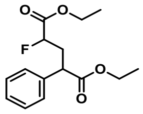

Diethyl 2-fluoro-4-phenylpentanedioate (3ar): The compound was according to the general procedure was purified with silica gel chromatography (petroleum ether / ethyl acetate = 15:1) as colorless liquid (42.8 mg, 76% yield). ^{1}H NMR (400 MHz, $CDCl_3$) δ 7.38-7.20 (m, 5H), 4.93 (m, 0.5H), 4.57 (m, 0.5H), 4.25-4.03 (m, 4H), 3.83 (m, 1H), 2.80-2.66 (m, 0.5H), 2.62-2.49 (m, 0.5H), 2.46-2.30 (m, 0.5H), 2.26-2.14 (m, 0.5H), 1.25 (q, J = 7.0 Hz, 3H), 1.17 (t, J = 7.1 Hz, 3H). ^{19}F NMR (376 MHz, $CDCl_3$) -193.40 (m, 0.5F), -193.88 (m, 0.5F). ^{13}C NMR (101 MHz, $CDCl_3$) δ 172.8 (172.7), 169.4 (d, J = 23.7 Hz) (169.2 (d, J = 23.7 Hz)), 138.0 (137.0), 128.9 (128.8), 128.1 (127.8), 127.8, (127.6), 87.0 (d, J = 184.8 Hz) (86.5 (d, J = 184.8 Hz)), 61.6 (d, J = 5.1 Hz) (61.1 (d, J = 2.0 Hz)), 46.9 (d, J = 3.0 Hz) (46.5 (d, J = 4.0 Hz)), 36.0 (d, J = 21.2 Hz) (35.6 (d, J = 21.2 Hz)), 14.0 (14.0), 14.0. HRMS (ESI): m/z [$(M+Na)^+$] calcd for $C_{15}H_{19}FNaO_4$, 305.1160. found, 305.1163.

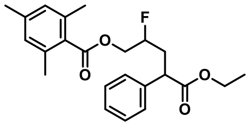

5-Ethoxy-2-fluoro-5-oxo-4-phenylpentyl 2,4,6-trimethylbenzoate (3as): The compound was according to the general procedure was purified with silica gel chromatography (petroleum ether / ethyl acetate = 10:1) as colorless liquid (59.6 mg, 77% yield). ^{1}H NMR (400 MHz, $CDCl_3$) δ 7.38-7.24 (m, 5H), 6.84 (s, 2H), 4.94-4.75 (m, 0.34H), 4.63-4.46 (m, 0.72H), 4.45-4.31 (m, 2H), 4.21-4.06 (m, 2H), 3.89 (dd, J = 10.9, 4.4 Hz, 0.34H), 3.84 (dd, J = 10.1, 5.4 Hz, 0.64H), 2.55-2.40 (m, 1H), 2.29 (s, 3H), 2.26 (s, 6H), 2.19-2.03 (m, 1H), 1.19 (m, 3H). ^{19}F NMR (376 MHz, $CDCl_3$) -189.40 (m, 0.33 F), -190.11 (m, 0.66 F). ^{13}C NMR (101 MHz, $CDCl_3$) δ 173.3 (173.1), 169.7 (169.6), 139.6, 138.6 (137.6), 135.5 (135.5), 130.30 (130.2), 129.0 (128.9), 128.5, 128.2, 127.7 (127.6), 89.4 (d, J = 174.7 Hz) (88.6 (d, J = 174.7 Hz)), 65.8 (d, J = 22.2 Hz) (65.7 (d, J = 22.2 Hz)), 61.1 (61.1), 47.3 (d, J = 3.5 Hz) (47.0 (d, J = 3.5 Hz)), 35.5 (d, J = 21.2 Hz) (35.0 (d, J = 20.2 Hz)), 21.1, 19.9 (19.9), 14.1. HRMS (ESI): m/z [$(M+Na)^+$] calcd for $C_{23}H_{27}FNaO_4$, 409.1786. found, 409.1785.

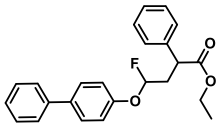

Ethyl 4-([1,1'-biphenyl]-4-yloxy)-4-fluoro-2-phenylbutanoate (3at): The compound was according to the general procedure with 10 mol% PC-5 and was purified with silica gel chromatography (petroleum ether / ethyl acetate = 15:1) as white solid (47.5 mg, 63% yield). ^{1}H NMR (400 MHz, $CDCl_3$) δ 7.53 (m, 4H), 7.42 (m, 2H), 7.38-7.30 (m, 6H), 7.09 (dd, J = 8.7, 2.6 Hz, 2H), 5.83 (m, 0.5H), 5.67 (m, 0.5H), 4.25-4.06 (m, 2H), 3.95 (dd, J = 13.5, 7.3 Hz, 1H), 2.90-2.75 (m, 1H), 2.50-2.35 (m, 1H), 1.21 (m, 3H). ^{19}F NMR (376 MHz, $CDCl_3$) -122.07 (m, 0.5F), -193.88 (m, 0.5F). ^{13}C NMR (101 MHz, $CDCl_3$) δ 173.0 (173.0), 155.9 (d, J = 3.0 Hz) (155.8 (d, J = 3.0 Hz)), 140.5, 138.0 (138.0), 136.7 (136.7), 129.0, 128.8, 128.4 (128.4), 127.9, 127.9, 127.7, 127.1, 126.9, 117.4, 108.9 (d, J = 220.2 Hz) (108.8 (d, J = 221.2 Hz)), 61.2 (61.2), 46.6 (d, J = 4.0

Hz) (46.5 (d, J = 4.0 Hz)), 38.2 (d, J = 24.2 Hz) (38.0 (d, J = 24.2 Hz)), 14.1. HRMS (ESI): m/z [$(M+Na)^+$] calcd for $C_{24}H_{23}FNaO_3$, 401.1523. found, 401.1526.

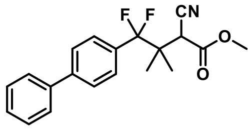

Methyl 4-([1,1'-biphenyl]-4-yl)-2-cyano-4,4-difluoro-3,3-dimethylbutanoate (3at): The compound was according to the general procedure with 10 mol% PC-5 for 48 hours and was purified with silica gel chromatography (petroleum ether / dichloromethane = 1:2) as light-yellow oil (29.5 mg, 43% yield). ^{1}H NMR (400 MHz, $CDCl_3$) δ 7.66 (d, *J* = 8.2 Hz, 2H), 7.60 (d, *J* = 7.3 Hz, 2H), 7.52 (d, *J* = 8.3 Hz, 2H), 7.47 (t, *J* = 7.5 Hz, 2H), 7.39 (t, *J* = 7.3 Hz, 1H), 3.87 (s, 1H), 3.84 (s, 3H), 1.36 (s, 3H), 1.25 (s, 3H). ^{19}F NMR (376 MHz, $CDCl_3$) δ -101.98 (t, J = 232.5 Hz, 2F). ^{13}C NMR (101 MHz, $CDCl_3$) δ 165.4, 143.3, 139.8, 131.9 (t, J = 27.3 Hz), 129.0, 128.1, 127.4 (t, J = 6.6 Hz), 127.2, 126.8, 124.2 (t, J = 253.0 Hz), 115.4, 53.5, 45.8 (t, J = 25.3 Hz), 43.0 (t, J = 3.0 Hz), 21.2 (t, J = 3.5 Hz), 20.1 (t, J = 3.5 Hz). HRMS (ESI): m/z [$(M+H)^+$] calcd for $C_{20}H_{20}F_2NO_2$, 344.1457. found, 344.1460.

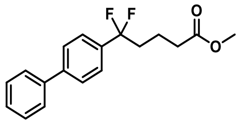

Methyl 5-([1,1'-biphenyl]-4-yl)-5,5-difluoropentanoate (3av): The compound was according to the general procedure with **1d** (0.1 mmol), **2v** (0.2 mmol), $K_2S_2O_8$ (0.3 mmol) and MeCN/H_2O (2 mL, 3:1) for 48 h and was purified with silica gel chromatography (petroleum ether / ethyl acetate = 20:1) as colorless liquid (18.8 mg, 62% yield). ^{1}H NMR (400 MHz, $CDCl_3$) δ 7.64 (d, J = 8.4 Hz, 2H), 7.59 (d, J = 7.1 Hz, 2H), 7.53 (s, 2H), 7.45 (d, J = 7.8 Hz, 2H), 7.39-7.36 (m, 1H), 3.67 (s, 3H), 2.38 (t, J = 7.4 Hz, 2H), 2.30-2.14 (m, 2H), 1.87-1.76 (m, 2H). ^{19}F NMR (376 MHz, $CDCl_3$) δ -95.26 (t, J = 16.9 Hz, 2F). ^{13}C NMR (101 MHz, $CDCl_3$) δ 173.4, 142.7, 140.3, 135.9 (t, J = 26.8 Hz), 128.9, 127.8, 127.2, 127.2 (t, J = 12.6 Hz), 125.4 (t, J = 6.1 Hz), 122.8 (t,

J = 242.9 Hz), 51.6, 38.2 (t, J = 28.3 Hz), 33.3, 18.2 (t, J = 4.5 Hz). HRMS (ESI): m/z [(M+Na)$^+$] calcd for $C_{18}H_{18}F_2NaO_2$, 327.1167. found, 327.1171.

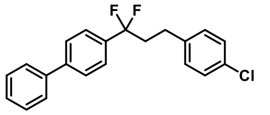

4-(3-(4-Chlorophenyl)-1,1-difluoropropyl)-1,1'-biphenyl (3aw): The compound was purified with silica gel chromatography (petroleum ether / ethyl acetate = 200:1) as light-yellow solid (48.6 mg, 71% yield). ^{1}H NMR (400 MHz, $CDCl_3$) δ 7.63 (d, J = 8.3 Hz, 2H), 7.60-7.51 (m, 4H), 7.44 (m, 2H), 7.39-7.33 (m, 1H), 7.25-7.20 (m, 2H), 7.07 (d, J = 8.4 Hz, 2H), 2.81-2.72 (m, 2H), 2.49-2.36 (m, 2H). ^{19}F NMR (376 MHz, $CDCl_3$) δ -95.82 (t, J = 17.0 Hz, 2F). ^{13}C NMR (101 MHz, $CDCl_3$) δ 142.8, 140.2, 138.9, 135.9 (t, J = 26.8 Hz), 132.0, 129.7, 128.9, 128.7, 127.9, 127.3, 127.3, 125.5 (t, J = 6.1 Hz), 122.5 (t, J = 243.4 Hz), 40.8 (t, J = 28.3 Hz), 28.3 (t, J = 4.0 Hz). HRMS (EI): m/z [M$^+$] calcd for $C_{21}H_{17}ClF_2$, 342.0981. found, 342.0980.

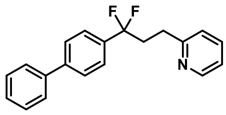

2-(3-([1,1'-Biphenyl]-4-yl)-3,3-difluoropropyl)pyridine (3ax): The compound was purified with silica gel chromatography (petroleum ether / ethyl acetate = 5:1) as white solid (47.0 mg, 76% yield). ^{1}H NMR (400 MHz, $CDCl_3$) δ 8.50 (d, J = 4.8 Hz, 1H), 7.63-7.53 (m, 7H), 7.44 (t, J = 7.5 Hz, 2H), 7.36 (t, J = 7.3 Hz, 1H), 7.12 (d, J = 7.8 Hz, 1H), 7.09 (dd, J = 7.4, 5.0 Hz, 1H), 3.03-2.97 (m, 2H), 2.66 (m, 2H). ^{19}F NMR (376 MHz, $CDCl_3$) δ -95.75 (t, J = 17.0 Hz, 2F). ^{13}C NMR (101 MHz, $CDCl_3$) δ 160.0, 149.4, 142.7, 140.3, 136.5, 136.0 (t, J = 27.3 Hz), 128.9, 127.8, 127.2, 125.5 (t, J = 6.1 Hz), 123.0, 122.9 (t, J = 243.4 Hz), 121.4, 38.7 (t, J = 28.3 Hz), 31.3 (t, J = 4.0 Hz). HRMS (ESI): m/z [(M+Na)$^+$] calcd for $C_{20}H_{17}F_2NNa$, 332.1221. found, 332.12216.

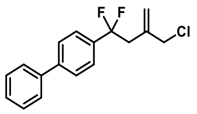

4-(3-(Chloromethyl)-1,1-difluorobut-3-en-1-yl)-1,1'-biphenyl (3ay): The compound was according to the general procedure with **1d** (0.6 mmol) and **2x** (0.2 mmol) and was

purified with silica gel chromatography (petroleum ether / ethyl acetate = 100:1) as colorless liquid (29.1 mg, 50% yield). When **1d** (0.2 mmol) and **2x** (0.6 mmol) were used, **3ay** was got with 64% yield. ^{1}H NMR (400 MHz, $CDCl_3$) δ 7.64 (d, J = 8.3 Hz, 2H), 7.61-7.58 (m, 2H), 7.54 (d, J = 8.3 Hz, 2H), 7.46 (dd, J = 10.2, 4.7 Hz, 2H), 7.38 (dd, J = 8.3, 6.3 Hz, 1H), 5.37 (s, 1H), 5.12 (s, 1H), 4.03 (s, 2H), 3.06 (t, J = 16.4 Hz, 2H). ^{19}F NMR (376 MHz, $CDCl_3$) δ -94.20 (t, J = 16.9 Hz, 2F). ^{13}C NMR (101 MHz, $CDCl_3$) δ 142.9 (t, J = 1.5 Hz), 140.1, 137.1 (t, J = 3.0 Hz), 135.6 (t, J = 26.8 Hz), 128.9, 127.9, 127.2, 127.2, 125.5 (t, J = 6.6 Hz), 121.8 (t, J = 244.9 Hz), 121.4, 48.5, 42.3 (t, J = 28.8 Hz). HRMS (ESI): m/z [(M+Na)$^+$] calcd for $C_{17}H_{15}{}^{35}ClF_2Na$, 315.0723. found, 315.0721.

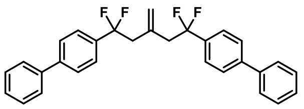

Meanwhile, di-addition product **3ay’** was purified with silica gel chromatography (petroleum ether / ethyl acetate = 60:1) as white solid (34.1 mg, 37% yield). ^{1}H NMR (400 MHz, $CDCl_3$) δ 7.60 (m, 8H), 7.53-7.41 (m, 8H), 7.37 (t, *J* = 7.3 Hz, 2H), 5.11 (s, 2H), 2.91 (t, *J* = 16.4 Hz, 4H). ^{19}F NMR (376 MHz, $CDCl_3$) δ -94.19 (t, J = 17.0 Hz, 2F). ^{13}C NMR (101 MHz, $CDCl_3$) δ 142.7, 140.2, 135.9 (t, J = 26.8 Hz), 132.8 (t, J = 3.0 Hz), 128.9, 127.8, 127.2, 127.1, 125.6 (t, J = 6.6 Hz), 123.6, 122.1 (t, J = 245.4 Hz), 45.6 (t, J = 28.3 Hz). HRMS (EI): m/z [M$^+$] calcd for $C_{30}H_{24}F_4$, 460.1809. found, 460.1808.

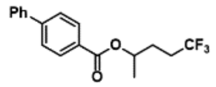

2-Fluoropent-4-en-1-yl 2,4,6-trimethylbenzoate (3az): The compound was according to the general procedure with 10 mol% PC-5, **2u** (0.1 mmol), **1ba** (0.3 mmol), 2,4,6-collidine (0.4 mmol), $K_2S_2O_8$ (0.3 mmol) and MeCN/H_2O (2 mL, 3:1) for 48 h and was purified with silica gel chromatography (petroleum ether / ethyl acetate = 25:1) as colorless oil (25.7 mg, 80% yield). ^{1}H NMR (400 MHz, $CDCl_3$) ^{1}H NMR (400 MHz, cdcl3) δ 8.09 (d, J = 8.1 Hz, 2H), 7.66 (d, J = 8.1 Hz, 2H), 7.62 (d, J = 7.7 Hz, 2H), 7.47

(t, J = 7.6 Hz, 2H), 7.39 (t, J = 7.2 Hz, 1H), 5.23 (m, 1H), 2.31-2.14 (m, 2H), 2.03-1.91 (m, 2H), 1.40 (d, J = 6.2 Hz, 3H). ^{19}F NMR (376 MHz, $CDCl_3$) δ -66.51 (t, J = 11.3 Hz, 3F). ^{13}C NMR (101 MHz, $CDCl_3$) δ 165.9, 145.9, 140.0, 130.1, 129.0, 128.2, 127.3, 127.1, 127.0 (q, J = 277.1 Hz), 69.9, 30.3 (q, J = 29.3 Hz), 28.5 (q, J = 2.5 Hz), 20.0. HRMS (ESI): m/z [$(M+Na)^+$] calcd for $C_{18}H_{17}F_3NaO_2$, 345.1073. found, 345.1070.

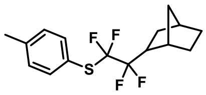

(2-Bicyclo[2.2.1]heptan-2-yl)-1,1,2,2-tetrafluoroethyl)(p-tolyl)sulfane (3ba): The compound was according to the general procedure with 10 mol% PC-5, **1be** (0.1 mmol), **2w** (0.3 mmol), 2,4,6-collidine (0.4 mmol), $K_2S_2O_8$ (0.3 mmol) and MeCN/H_2O (2 mL, 3:1) for 48 h and was purified with silica gel chromatography (petroleum ether / ethyl acetate = 100:1) as colorless oil (16.2 mg, 51% yield). ^{1}H NMR (400 MHz, $CDCl_3$) δ 7.52 (d, J = 8.0 Hz, 2H), 7.19 (d, J = 7.9 Hz, 2H), 2.60 (s, 1H), 2.37 (s, 3H), 2.29 (s, 1H), 2.22-2.09 (m, 1H), 1.74-1.67 (m, 1H), 1.55-1.45 (m, 4H), 1.23-1.13 (m, 3H). ^{19}F NMR (376 MHz, $CDCl_3$) δ -86.10 (m, 2F), -113.53 (m, 2F). ^{13}C NMR (101 MHz, $CDCl_3$) δ 140.8, 137.1, 129.9, 125.3 (tt, J = 289.4, 38.4 Hz), 120.8, 119.3 (tt, J = 255.0, 32.3 Hz), 43.4 (t, J = 21.7 Hz), 37.1, 36.8, 35.7, 32.2, 30.5, 28.0, 21.3. HRMS (ESI): m/z [$(M+Na)^+$] calcd for $C_{16}H_{18}F_4NaS$, 341.0958. found, 341.0963.

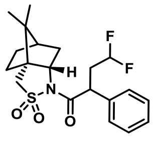

1-((3aR,6R,7aS)-8,8-Dimethyl-2,2-dioxidotetrahydro-3H-3a,6-methanobenzo[c]isothiazol-1(4H)-yl)-4,4-difluoro-2-phenylbutan-1-one (3bb): The compound was according to the general procedure at 0.1 mmol scale and was purified with silica gel chromatography (petroleum ether / ethyl acetate = 7:1) as white solid (29.4 mg, 74% yield). ^{1}H NMR (400 MHz, $CDCl_3$) δ 7.54-7.15 (m, 5H), 5.93-5.52 (m, 1H), 4.51 (dd, J = 8.9, 5.8 Hz, 0.5H), 4.43 (s, 0.5H), 3.99-3.75 (m, 1H), 3.59-3.31 (m, 2H), 2.83-2.59 (m, 1H), 2.42-2.22 (m, 1H), 2.17-1.99 (m, 1H), 1.96-1.68 (m, 4H), 1.30 (m, 2H), 1.16 (s, 1.5H), 0.95 (s, 1.5H), 0.85 (s, 1.5H), 0.66 (s, 1.5H). ^{19}F

NMR (376 MHz, $CDCl_3$) δ -115.95 (m, 2F). ^{13}C NMR (101 MHz, $CDCl_3$) δ 171.4 (171.2), 136.4, 128.9, 128.7 (128.6), 128.1, 127.9 (127.9), 115.8 (t, J = 240.4 Hz), 115.7 (t, J = 240.4 Hz), 65.6 (64.9), 53.0 (48.5), 47.8 (47.6), 45.9 (t, J = 6.1 Hz) (45.0 (t, J = 5.6 Hz)), 44.5 (44.4), 39.8 (t, J = 21.7 Hz) (37.3 (t, J = 22.7 Hz)), 32.9 (32.6), 26.4 (26.4), 20.6 (20.1), 19.9 (19.8). HRMS (ESI): m/z [$(M+H)^+$] calcd for $C_{20}H_{26}F_2NO_3S$, 398.1596. found, 398.1593.

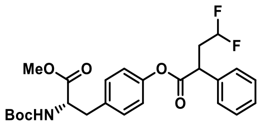

4-((S)-2-((Tert-butoxycarbonyl)amino)-3-methoxy-3-oxopropyl)phenyl 4,4-difluoro-2-phenylbutanoate (3bc): The compound was according to the general procedure at 0.1 mmol scale for 48 hours and was purified with silica gel chromatography (petroleum ether / ethyl acetate = 3:1) as white solid (31.5 mg, 66% yield). ^1H NMR (400 MHz, $CDCl_3$) δ 7.58-7.28 (m, 5H), 7.07 (d, J = 8.3 Hz, 2H), 6.89 (d, J = 8.4 Hz, 2H), 5.80 (tt, J = 56.4, 4.5 Hz, 1H), 4.98 (d, J = 8.0 Hz, 1H), 4.53 (dd, J = 13.6, 6.0 Hz, 1H), 4.04 (t, J = 7.6 Hz, 1H), 3.66 (s, 3H), 3.03 (m, 2H), 2.83-2.68 (m, 1H), 2.42-2.27 (m, 1H), 1.39 (s, 9H). ^{19}F NMR (376 MHz, $CDCl_3$) δ -117.19 (m, 2F). ^{13}C NMR (101 MHz, $CDCl_3$) δ 172.17, 171.2, 155.1, 149.6, 136.8, 133.9, 130.3, 129.3, 128.2, 127.8, 121.3, 115.7 (t, J = 240.4 Hz), 80.0, 54.3, 52.3, 45.5 (t, J = 5.6 Hz), 37.6, 37.4 (t, J = 22.2 Hz), 28.3. HRMS (ESI): m/z [$(M+Na)^+$] calcd for $C_{25}H_{29}F_2NNaO_6$, 500.1855. found, 500.1854.

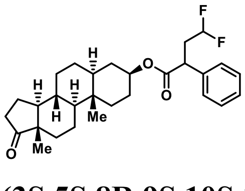

(3S,5S,8R,9S,10S,13S,14S)-10,13-Dimethyl-17-oxohexadecahydro-1H-cyclopenta[a]phenanthren-3-yl 4,4-difluoro-2-phenylbutanoate (3bd): The compound was according to the general procedure at 0.1 mmol scale and was purified with silica gel chromatography (petroleum ether / ethyl acetate = 12:1) as colorless oil

(33.5 mg, 71% yield). ^{1}H NMR (400 MHz, $CDCl_3$) δ 7.43-7.22 (m, 5H), 5.70 (tt, J = 56.6, 4.2 Hz, 1H), 4.68 (m, 1H), 3.73 (t, J = 7.6 Hz, 1H), 2.71-2.55 (m, 1H), 2.40 (dd, J = 19.2, 8.8 Hz, 1H), 2.31-2.16 (m, 1H), 2.10-1.99 (m, 1H), 1.91 (m, 1H), 1.79-1.60 (m, 5H), 1.55-1.41 (m, 3H), 1.37-1.10 (m, 8H), 1.04-0.92 (m, 2H), 0.82 (s, 3H), 0.78 (s, 3H), 0.66 (m, 1H). ^{19}F NMR (376 MHz, $CDCl_3$) δ -117.08 (m, 2F). ^{13}C NMR (101 MHz, $CDCl_3$) δ 172.0, 172.0, 137.6, 128.9, 127.7, 127.61, 115.8 (t, J = 239.9 Hz), 74.4, 54.2, 51.3, 47.7, 45.7 (t, J = 6.6 Hz), 44.6 (d, J = 6.1 Hz), 37.6 (t, J = 22.2 Hz), 36.6 (d, J = 10.1 Hz), 35.8, 35.6, 35.0, 33.7 (d, J = 32.3 Hz), 31.5, 30.7, 28.2 (d, J = 5.1 Hz), 27.1 (d, J = 33.3 Hz), 21.7, 20.4, 13.8, 12.2. HRMS (ESI): m/z [$(M+Na)^+$] calcd for $C_{29}H_{38}F_2NaO_3$, 495.2681. found, 495.2686.

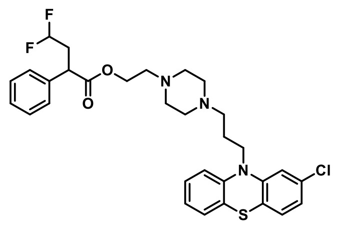

2-(4-(3-(2-Chloro-10H-phenothiazin-10-yl)propyl)piperazin-1-yl)ethyl 4,4-difluoro-2-phenylbutanoate (3be): The compound was according to the general procedure with 10 mol% PC-5 at 0.1 mmol scale and was purified with silica gel chromatography (petroleum ether / ethyl acetate = 2:1) as white solid (22.8 mg, 71% yield). ^{1}H NMR (400 MHz, $CDCl_3$) δ 7.35-7.21 (m, 5H), 7.17-7.07 (m, 2H), 7.00 (d, J = 8.1 Hz, 1H), 6.91 (t, J = 7.5 Hz, 1H), 6.86 (d, J = 8.1 Hz, 2H), 6.82 (d, J = 1.7 Hz, 1H), 5.75 (tt, J = 56.6, 4.7 Hz, 1H), 4.18 (t, J = 5.6 Hz, 2H), 3.86 (t, J = 6.7 Hz, 2H), 3.78 (t, J = 7.7 Hz, 1H), 2.65 (m, 2H), 2.51 (t, J = 5.6 Hz, 2H), 2.47-2.22 (m, 10H), 1.97-1.87 (m, 2H). ^{19}F NMR (376 MHz, $CDCl_3$) δ -117.15 (m, 2F). ^{13}C NMR (101 MHz, $CDCl_3$) δ 172.4, 146.5, 144.5, 137.3, 133.2, 129.0, 127.9, 127.9, 127.8, 127.6, 127.5, 124.8, 123.6, 123.0, 122.3, 115.9, 115.9, 115.8 (t, J = 239.9 Hz), 62.6, 56.3, 55.4, 53.0, 52.9, 45.5 (t, J = 6.1 Hz), 45.3, 37.4 (t, J = 22.2 Hz), 24.0. HRMS (ESI): m/z [$(M+Na)^+$] calcd for $C_{31}H_{35}{}^{35}ClF_2N_3O_2S$, 586.2101. found, 586.2103.

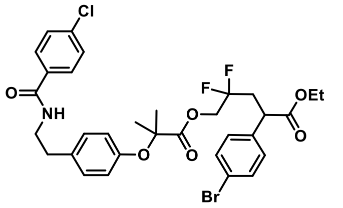

Ethyl 2-(4-bromophenyl)-5-((2-(4-(2-(4-chlorobenzamido)ethyl)phenoxy)-2-methylpropanoyl)oxy)-4,4-difluoropentanoate (3bf): The compound was according to the general procedure **1r** (0.1 mmol) and **2e** (0.2 mmol) for 48 hours and was purified with silica gel chromatography (petroleum ether / ethyl acetate = 3:1) as white solid (47.5 mg, 70% yield). ^{1}H NMR (400 MHz, $CDCl_3$) δ 7.61 (d, J = 8.5 Hz, 2H), 7.42 (d, J = 8.4 Hz, 2H), 7.34 (d, J = 8.5 Hz, 2H), 7.10 (d, J = 8.5 Hz, 2H), 7.05 (d, J = 8.5 Hz, 2H), 6.77 (d, J = 8.6 Hz, 2H), 6.33 (s, 1H), 4.34-4.20 (m, 2H), 4.16-4.02 (m, 2H), 3.83 (dd, J = 8.8, 4.9 Hz, 1H), 3.63 (dd, J = 12.9, 6.7 Hz, 2H), 2.83 (t, J = 6.8 Hz, 2H), 2.80-2.66 (m, 1H), 2.11 (m, 1H), 1.60 (d, J = 3.5 Hz, 6H), 1.17 (t, J = 7.1 Hz, 3H). ^{19}F NMR (376 MHz, $CDCl_3$) δ -104.95 (m, 2F). ^{13}C NMR (101 MHz, $CDCl_3$) δ 173.2, 172.3, 166.4, 153.9, 137.6, 137.0, 133.0, 132.8, 132.0, 129.6, 129.4, 128.8, 128.3, 121.8, 120.4 (t, J = 244.4 Hz), 119.3, 79.0, 64.3 (t, J = 33.8 Hz), 61.5, 44.2 (t, J = 3.5 Hz), 41.3, 37.1 (t, J = 23.7 Hz), 34.7, 25.4, 25.3, 13.9. HRMS (ESI): m/z [$(M+Na)^+$] calcd for $C_{32}H_{34}{}^{79}Br^{35}ClF_2NO_6$, 680.1221. found, 680.1216.

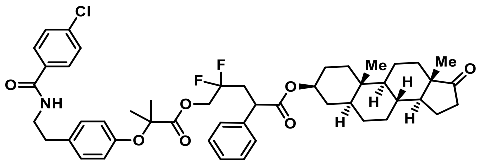

(3S,5S,8R,9S,10S,13S,14S)-10,13-Dimethyl-17-oxohexadecahydro-1H-cyclopenta[a]phenanthren-3-yl 5-((2-(4-(2-(4-chlorobenzamido)ethyl)phenoxy)-2-methylpropanoyl)oxy)-4,4-difluoro-2-phenylpentanoate (3bg): The compound was according to the general procedure with 10 mol% PC-5, **1r** (0.1 mmol) and **2ab** (0.2 mmol) and was purified with silica gel chromatography (petroleum ether / ethyl acetate = 2:1) as white solid (44.7 mg, 53% yield). ^{1}H NMR (400 MHz, $CDCl_3$) δ 7.58 (d, J = 8.2 Hz, 2H), 7.33-7.23 (m, 5H), 7.18 (d, J = 6.8 Hz, 2H), 7.02 (d, J = 8.1 Hz, 2H), 6.74 (d, J = 8.2 Hz, 2H), 6.36 (s, 1H), 4.63-4.50 (m, 1H), 4.33-4.15 (m, 2H), 3.80 (dd, J =

8.8, 3.9 Hz, 1H), 3.59 (d, J = 5.8 Hz, 2H), 2.80 (t, J = 6.5 Hz, 2H), 2.77-2.59 (m, 1H), 2.38 (dd, J = 19.3, 8.8 Hz, 1H), 2.18-1.97 (m, 2H), 1.88 (m, 1H), 1.72 (t, J = 11.5 Hz, 4H), 1.57 (s, 6H), 1.45 (m, 3H), 1.38-1.14 (m, 8H), 1.05 (m, 1H), 0.94-0.82 (m, 2H), 0.80 (s, 3H), 0.76 (s, 3H), 0.63 (m, 1H). ^{19}F NMR (376 MHz, $CDCl_3$) δ -104.84 (m, 2F). ^{13}C NMR (101 MHz, $CDCl_3$) δ 173.2, 172.3, 172.3, 166.4, 153.9, 138.1, 137.6, 133.0, 132.8, 129.6, 128.8, 128.7, 128.4, 127.7, 127.6, 120.6 (t, J = 244.4 Hz), 119.3, 79.0, 74.5 (d, J = 3.0 Hz), 64.4 (t, J = 33.8 Hz), 54.2, 51.3, 47.8, 44.9 (d, J = 2.0 Hz), 44.6 (d, J = 4.0 Hz), 41.3, 37.3 (t, J = 23.7 Hz), 36.6 (d, J = 9.1 Hz), 35.8, 35.6, 35.0, 34.7, 33.5 (d, J = 23.2 Hz), 31.5, 30.8, 28.2 (d, J = 6.1 Hz), 27.1 (d, J = 22.2 Hz), 25.5, 25.3, 21.8, 20.4, 13.8, 12.2. HRMS (ESI): m/z [$(M+Na)^+$] calcd for $C_{49}H_{58}{}^{35}ClF_2NNaO_7$, 868.3762. found, 868.3760.

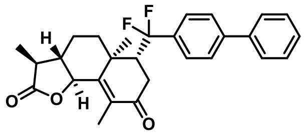

(3S,3aS,5aS,6R,9bS)-6-([1,1'-Biphenyl]-4-yldifluoromethyl)-3,5a,9-trimethyl-3a,5,5a,6,7,9b-hexahydronaphtho[1,2-b]furan-2,8(3H,4H)-dione (3bh-1): The compound was according to the general procedure with 10 mol% PC-5, **1r** (0.1 mmol) and **2ad** (0.2 mmol) and was purified with silica gel chromatography (petroleum ether / ethyl acetate = 8:1) as white solid (10.4 mg, 23% yield). ^{1}H NMR (400 MHz, $CDCl_3$) δ 7.63 (d, J = 8.1 Hz, 4H), 7.58 (d, J = 7.9 Hz, 2H), 7.47 (m, 4H), 7.39 (t, J = 7.3 Hz, 1H), 4.80 (d, J = 11.6 Hz, 1H), 2.66 (m, 2H), 2.60 (m, 1H), 2.36 (m, 2H), 2.08 (m, 1H), 2.01 (s, 3H), 1.98-1.87 (m, 1H), 1.75-1.63 (m, 2H), 1.58 (d, J = 2.0 Hz, 3H), 1.29 (d, J = 6.9 Hz, 3H). ^{19}F NMR (376 MHz, $CDCl_3$) δ -86.98 (d, J = 244.4 Hz, 1F), δ -105.51 (dd, J = 244.4, 22.6 Hz, 1F). ^{13}C NMR (101 MHz, $CDCl_3$) δ 196.8, 177.5, 153.1, 143.2, 140.0, 135.8 (t, J = 27.3 Hz), 128.9, 128.9, 128.0, 127.4, 127.2, 125.7 (dd, J = 8.6, 4.5 Hz), 124.3 (t, J = 251.5 Hz), 81.6, 52.4, 51.9 (t, J = 23.7 Hz), 43.8, 41.3, 41.2 (d, J = 10.1 Hz), 34.5 (t, J = 4.5 Hz), 25.0, 19.2 (d, J = 5.1 Hz), 12.4, 11.2. HRMS (ESI): m/z [$(M+Na)^+$] calcd for $C_{28}H_{28}F_2NaO_3$, 473.1899. found, 473.1897.

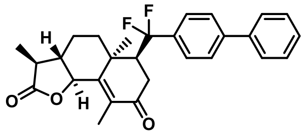

(3S,3aS,5aS,6S,9bS)-6-([1,1'-Biphenyl]-4-yldifluoromethyl)-3,5a,9-trimethyl-3a,5,5a,6,7,9b-hexahydronaphtho[1,2-b]furan-2,8(3H,4H)-dione (3bh-2): The compound was purified with silica gel chromatography (petroleum ether / ethyl acetate = 8:1) as white solid (15.8 mg, 35% yield). ^{1}H NMR (400 MHz, $CDCl_3$) δ 7.63 (d, J = 8.1 Hz, 4H), 7.58 (d, J = 7.3 Hz, 2H), 7.47 (t, J = 7.9 Hz, 4H), 7.39 (t, J = 6.1 Hz, 1H), 4.94 (d, J = 10.2 Hz, 1H), 2.64 (dd, J = 26.9, 12.9 Hz, 1H), 2.42 (m, 2H), 2.35-2.23 (m, 2H), 2.16 (m, 2H), 2.01 (s, 3H), 1.89-1.74 (m, 2H), 1.61 (s, 3H), 1.28 (d, J = 6.9 Hz, 3H). ^{19}F NMR (376 MHz, $CDCl_3$) δ -86.87 (d, J = 244.4 Hz, 1F), δ -105.91 (dd, J = 244.4, 26.3 Hz, 1F). ^{13}C NMR (101 MHz, $CDCl_3$) δ 196.9, 177.5, 154.2, 143.2, 140.0, 135.7 (t, J = 26.8 Hz), 128.9, 127.9, 127.4, 127.4, 127.3, 125.7 (dd, J = 8.1, 5.1 Hz), 124.6 (t, J = 252.0 Hz), 81.4, 54.0, 50.6 (t, J = 24.2 Hz), 43.5, 41.2, 34.4 (t, J = 3.5 Hz), 32.2 (t, J = 3.5 Hz), 27.5 (d, J = 8.1 Hz), 23.9, 12.5, 11.7. HRMS (ESI): m/z [$(M+Na)^+$] calcd for $C_{28}H_{28}F_2NaO_3$, 473.1899. found, 473.1902.

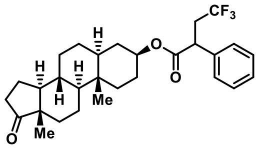

(3S,5S,8R,9S,10S,13S,14S)-10,13-Bimethyl-17-oxohexadecahydro-1H-cyclopenta[a]phenanthren-3-yl 4,4,4-trifluoro-2-phenylbutanoate (3bi): The compound was according to the general procedure with **1ba** (0.3 mmol) and 2,4,6-collidine (0.4 mmol) for 48 h and was purified with silica gel chromatography (petroleum ether / ethyl acetate = 15:1) as colorless oil (38.6 mg, 79% yield). ^{1}H NMR (400 MHz, $CDCl_3$) δ 7.34-7.24 (m, 5H), 4.68 (dt, J = 16.4, 5.5 Hz, 1H), 3.82 (dd, J = 9.3, 4.5 Hz, 1H), 3.15-3.00 (m, 1H), 2.45-2.34 (m, 2H), 2.07-1.98 (m, 1H), 1.92-1.85 (m, 1H), 1.78-1.67 (m, 3H), 1.64-1.58 (m, 2H), 1.46 (m, 3H), 1.33-1.06 (m, 8H), 0.97 (m, 2H), 0.82 (s, 3H), 0.79 (s, 3H), 0.66 (td, J = 11.5, 3.7 Hz, 1H). ^{19}F NMR (376 MHz, $CDCl_3$) δ -65.33 (m, 3F). ^{13}C NMR (101 MHz, $CDCl_3$) δ 171.4,171.4, 137.3, 129.0,

127.9, 127.5, 126.1 (q, J = 278.4 Hz), 74.7 (d, J = 1.0Hz), 54.2, 51.3, 47.8, 45.6 (t, J = 3.0Hz), 44.6 (d, J = 4.0Hz), 37.2 (q, J = 28.6Hz), 36.6 (d, J = 8.1Hz), 35.8, 35.6, 35.0, 33.5 (d, J = 25.2Hz), 31.5, 30.8, 28.2 (d, J = 3.0Hz), 27.1 (d, J = 24.2Hz), 21.8, 20.5, 13.8, 12.2. HRMS (ESI): m/z [(M+Na)$^+$] calcd for $C_{29}H_{37}F_3NaO_3$, 513.2587. found, 513.2591.

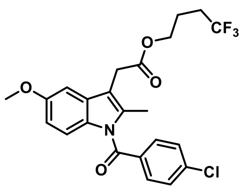

4,4,4-Trifluorobutyl 2-(1-(4-chlorobenzoyl)-5-methoxy-2-methyl-1H-indol-3-yl)acetate (3bj): The compound was according to the general procedure with 10 mol% PC-5, **1ba** (0.3 mmol), **2ae** (0.1 mmol), 2,4,6-collidine (0.4 mmol), $K_2S_2O_8$ (0.3 mmol) and MeCN/H_2O (2 mL, 3:1) for 48 h and was purified with silica gel chromatography (petroleum ether / ethyl acetate = 10:1) as white solid (31.3 mg, 67% yield). ^{1}H NMR (400 MHz, $CDCl_3$) δ 7.63 (d, J = 8.5 Hz, 2H), 7.44 (d, J = 8.5 Hz, 2H), 6.93 (d, J = 2.5 Hz, 1H), 6.84 (d, J = 9.0 Hz, 1H), 6.65 (dd, J = 9.0, 2.5 Hz, 1H), 4.13 (t, J = 6.2 Hz, 2H), 3.80 (s, 3H), 3.66 (s, 2H), 2.37 (s, 3H), 2.13-2.00 (m, 2H), 1.87 (dt, J = 12.9, 6.2 Hz, 2H). ^{19}F NMR (376 MHz, $CDCl_3$) δ -66.44 (t, J = 3.0 Hz, 3F). ^{13}C NMR (101 MHz, $CDCl_3$) δ 170.7, 168.3, 156.1, 139.3, 136.0, 133.9, 131.2, 130.9, 130.5, 129.2, 126.8 (q, J = 272.0 Hz), 115.0, 112.3, 111.6, 101.3, 63.2, 55.6, 30.5 (q, J = 29.3 Hz), 30.3, 21.5 (q, J = 3.0 Hz), 13.3. HRMS (ESI): m/z [(M+Na)$^+$] calcd for $C_{23}H_{21}{}^{35}ClF_3NNaO_4$, 490.1003. found, 490.1000.

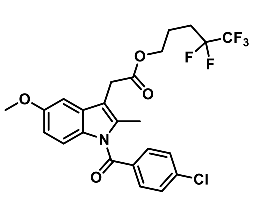

4,4,5,5,5-Pentafluoropentyl 2-(1-(4-chlorobenzoyl)-5-methoxy-2-methyl-1H-indol-3-yl)acetate (3bk): The compound was according to the general procedure with 10 mol% PC-5, **1bf** (0.3 mmol), **2ae** (0.1 mmol), 2,4,6-collidine (0.4 mmol), $K_2S_2O_8$ (0.3 mmol)

and MeCN/H_2O (2 mL, 3:1) for 48 h and was purified with silica gel chromatography (petroleum ether / ethyl acetate = 10:1) as colorless oil (32.6 mg, 63% yield). ^{1}H NMR (400 MHz, $CDCl_3$) δ 7.63 (d, J = 8.4 Hz, 2H), 7.44 (d, J = 8.4 Hz, 2H), 6.93 (d, J = 2.4 Hz, 1H), 6.83 (d, J = 9.0 Hz, 1H), 6.65 (dd, J = 9.0, 2.5 Hz, 1H), 4.15 (t, J = 6.0 Hz, 2H), 3.80 (s, 3H), 3.67 (s, 2H), 2.38 (s, 3H), 2.08-1.88 (m, 4H). ^{19}F NMR (376 MHz, $CDCl_3$) δ -85.54 (s, 3F), -118.50 (t, J = 18.8 Hz, 2F). ^{13}C NMR (101 MHz, $CDCl_3$) δ 170.7, 168.3, 156.1, 139.3, 136.0, 133.8, 131.2, 130.8, 130.5, 129.1, 119.0 (qt, J = 285.8, 35.9 Hz), 115.5 (tq, J = 252.5, 37.7 Hz), 115.0, 112.3, 111.6, 101.2, 63.4, 55.6, 30.2, 27.5 (t, J = 22.7 Hz), 20.1 (t, J = 3.5 Hz), 13.3. HRMS (ESI): m/z [$(M+Na)^+$] calcd for $C_{24}H_{21}{}^{35}ClF_5NNaO_4$, 540.0971. found, 540.0972.

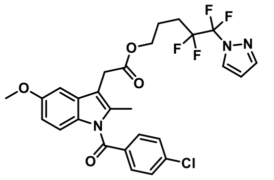

4,4,5,5-Tetrafluoro-5-(1H-pyrazol-1-yl)pentyl 2-(1-(4-chlorobenzoyl)-5-methoxy-2-methyl-1H-indol-3-yl)acetate (3bl): The compound was according to the general procedure with 10 mol% PC-5, **1bc** (0.3 mmol), **2ae** (0.1 mmol), 2,4,6-collidine (0.4 mmol), $K_2S_2O_8$ (0.3 mmol) and MeCN/H_2O (2 mL, 3:1) for 48 h and was purified with silica gel chromatography (petroleum ether / ethyl acetate = 10:1) as colorless oil (32.6 mg, 63% yield). ^{1}H NMR (400 MHz, $CDCl_3$) δ 7.77 (s, 1H), 7.72 (s, 1H), 7.66 (d, J = 8.6 Hz, 2H), 7.46 (d, J = 8.6 Hz, 2H), 6.95 (d, J = 2.3 Hz, 1H), 6.86 (d, J = 9.0 Hz, 1H), 6.67 (dd, J = 9.0, 2.5 Hz, 1H), 6.44 (s, 1H), 4.15 (t, J = 6.3 Hz, 2H), 3.83 (s, 3H), 3.67 (s, 2H), 2.38 (s, 3H), 2.17 (m, 2H), 1.92 (m, 2H). ^{19}F NMR (376 MHz, $CDCl_3$) δ -98.43 (s, 2F), -115.31 (t, J = 18.8 Hz, 2F). ^{13}C NMR (101 MHz, $CDCl_3$) δ 170.7, 168.3, 156.1, 143.0, 139.3, 136.0, 133.9, 131.2, 130.8, 130.6, 129.2, 129.1, 117.6 (tt, J = 255.5, 37.4 Hz), 115.0, 113.7 (tt, J = 266.6, 34.3 Hz),112.4, 111.7, 108.0, 101.2, 63.7, 55.7, 30.3, 27.9 (t, J = 22.7 Hz), 20.3 (t, J = 3.5 Hz), 13.3. HRMS (ESI): m/z [$(M+Na)^+$] calcd for $C_{27}H_{24}{}^{35}ClF_4N_3NaO_4$, 588.1284. found, 588.1288.

4.2 Representative experimental procedure for synthesis of compounds 5

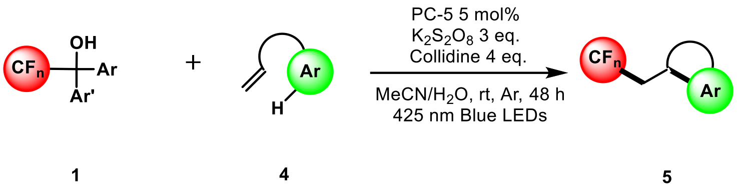

To a 4 mL vial equipped with a stir bar was added fluorinated alcohol **1** (0.2 mmol, 2.0 equiv.), PC-5 (3.3 mg, 5 μmol, 5 mol%), $K_2S_2O_8$ (81.0 mg, 0.3 mmol, 3.0 equiv.) and alkenes **4** (0.1 mmol, 1.0 equiv.) The vial was sealed, evacuated and backfilled with Argon three times, then collidine (52 μL, 0.4 mmol, 4.0 equiv.) and 1.5 mL MeCN and 0.5 mL H_2O were added. After degassing with Argon balloon for 8 minutes, the reaction mixture was irradiated with 10 W blue LEDs lamps for 48 hours at ambient temperature. The reaction mixture was then concentrated and purified on a preparative TLC with petroleum ether/ethyl acetate as the eluent to afford the products **5**.

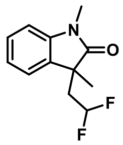

3-(2,2-Difluoroethyl)-1,3-dimethylindolin-2-one (5a): The compound was according to the general procedure and was purified with silica gel chromatography (petroleum ether / ethyl acetate = 15:1) as colorless liquid (18.7 mg, 83% yield). Known compound.[11] ^{1}H NMR (400 MHz, $CDCl_3$) δ 7.29 (td, J = 7.7, 1.2 Hz, 1H), 7.21 (d, J = 6.9 Hz, 1H), 7.07 (td, J = 7.5, 0.8 Hz, 1H), 6.86 (d, J = 7.8 Hz, 1H), 5.7 -5.42 (m, 1H), 3.21 (s, 3H), 2.48 (dt, J = 14.2, 8.0 Hz, 1H), 2.33-2.20 (m, 1H), 1.39 (s, 3H). ^{19}F NMR (376 MHz, $CDCl_3$) δ -114.33 (m, 2F). ^{13}C NMR (101 MHz, $CDCl_3$) δ 179.1, 142.9, 132.0, 128.5, 122.8, 122.8, 115.1 (t, J = 240.4 Hz), 108.5, 44.6 (t, J = 5.1 Hz), 41.4 (t, J = 21.7 Hz), 26.4, 24.4.

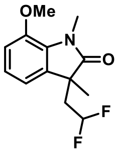

3-(2,2-Difluoroethyl)-7-methoxy-1,3-dimethylindolin-2-one (5b): The compound was according to the general procedure and was according to the general procedure with 10 mol% PC-5 and was purified with silica gel chromatography (petroleum ether / ethyl acetate = 12:1) as colorless liquid (16.3 mg, 64% yield). Known compound.[12] ^{1}H NMR (400 MHz, $CDCl_3$) δ 7.03 (dd, J = 8.2, 7.5 Hz, 1H), 6.85 (dd, J = 12.4, 7.8 Hz, 2H), 5.58 (m, 1H), 3.87 (s, 3H), 3.49 (s, 3H), 2.54-2.41 (m, 1H), 2.32-2.17 (m, 1H), 1.38 (s, 3H). ^{19}F NMR (376 MHz, $CDCl_3$) δ -114.26 (m, 2F). ^{13}C NMR (101 MHz, $CDCl_3$) δ 179.3, 145.6, 133.7, 130.7, 123.3, 115.3, 115.2 (t, J = 240.4 Hz), 112.2, 55.8, 44.6 (t, J = 5.1 Hz), 41.5 (t, J = 21.7 Hz), 29.7, 24.6.

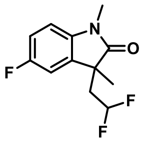

3-(2,2-Difluoroethyl)-5-fluoro-1,3-dimethylindolin-2-one (5c): The compound was according to the general procedure and was purified with silica gel chromatography (petroleum ether / ethyl acetate = 15:1) as colorless liquid (20.9 mg, 86% yield). Known compound.[11] ^{1}H NMR (400 MHz, $CDCl_3$) δ 7.01 (m, 2H), 6.80 (m, 1H), 5.63 (m, 1H), 3.22 (s, 3H), 2.56-2.45 (m, 1H), 2.35-2.21 (m, 1H), 1.41 (s, 3H). ^{19}F NMR (376 MHz, $CDCl_3$) δ -114.22 (m, 2F), -120.14 (m, 1F). ^{13}C NMR (101 MHz, $CDCl_3$) δ 178.8, 159.4 (d, J = 242.4 Hz), 138.8 (d, J = 1.0 Hz), 133.8 (d, J = 8.1 Hz), 114.9 (t, J = 240.9 Hz), 114.7 (d, J = 23.2 Hz), 111.2 (d, J = 25.3 Hz), 109.0 (d, J = 8.1 Hz), 45.0 (t, J = 6.1 Hz), 41.2 (t, J = 21.7 Hz), 26.5, 24.3.

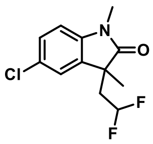

5-Chloro-3-(2,2-difluoroethyl)-1,3-dimethylindolin-2-one (5d): The compound was according to the general procedure and was purified with silica gel chromatography (petroleum ether / ethyl acetate = 15:1) as white solid (21.0 mg, 80% yield). Known compound.[11] ^{1}H NMR (400 MHz, $CDCl_3$) δ 7.29 (dd, J = 8.3, 2.0 Hz, 1H), 7.21 (d, J = 1.9 Hz, 1H), 6.80 (d, J = 8.3 Hz, 1H), 5.78-5.47 (m, 1H), 3.24 (d, J = 17.8 Hz, 3H),

2.56-2.45 (m, 1H), 2.29 (m, 1H), 1.41 (s, 3H). ^{19}F NMR (376 MHz, CDCl$_3$) δ -114.32 (m, 2F). ^{13}C NMR (101 MHz, CDCl$_3$) δ 178.6, 141.5, 133.8, 128.5, 128.2, 123.4, 114.8 (t, J = 240.9 Hz), 109.4, 44.8 (t, J = 5.1 Hz), 41.2 (t, J = 21.7 Hz), 26.5, 24.3.

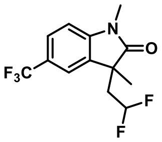

3-(2,2-Difluoroethyl)-1,3-dimethyl-5-(trifluoromethyl)indolin-2-one (5e): The compound was according to the general procedure and was purified with silica gel chromatography (petroleum ether / ethyl acetate = 15:1) as white solid (21.8 mg, 75% yield). Known compound.[11] ^{1}H NMR (400 MHz, CDCl$_3$) δ 7.60 (d, J = 8.2 Hz, 1H), 7.46 (s, 1H), 6.95 (d, J = 8.2 Hz, 1H), 5.61 (m, 1H), 3.26 (s, 3H), 2.53 (m, 1H), 2.40-2.27 (m, 1H), 1.44 (s, 3H). ^{19}F NMR (376 MHz, CDCl$_3$) δ -61.46 (s, 3F), -114.41 (m, 2F). ^{13}C NMR (101 MHz, CDCl$_3$) δ 179.0, 145.9, 132.7, 126.3 (q, J = 3.7 Hz), 125.1 (q, J = 32.7 Hz), 124.3 (q, J = 272.4 Hz), 119.9 (q, J = 3.7 Hz), 114.7 (t, J = 240.9 Hz), 108.25, 44.5 (t, J = 5.1 Hz), 41.1 (t, J = 21.7 Hz), 26.6, 24.3.

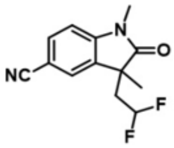

3-(2,2-Difluoroethyl)-1,3-dimethyl-2-oxoindoline-5-carbonitrile (5f): The compound was according to the general procedure and was purified with silica gel chromatography (petroleum ether / ethyl acetate = 15:1) as white solid (20.1 mg, 79% yield). Known compound.[11] ^{1}H NMR (400 MHz, CDCl$_3$) δ 7.65 (dd, J = 8.2, 1.6 Hz, 1H), 7.51 (d, J = 1.5 Hz, 1H), 6.96 (d, J = 8.2 Hz, 1H), 5.62 (m, 1H), 3.27 (s, 3H), 2.54 (m, 1H), 2.34 (m, 1H), 1.44 (s, 3H). ^{19}F NMR (376 MHz, CDCl$_3$) δ -114.40 (m, 2F). ^{13}C NMR (101 MHz, CDCl$_3$) δ 178.8, 146.8, 133.8, 133.2, 126.4, 118.9, 114.6 (t, J = 241.4 Hz), 108.9, 106.0, 44.4 (dd, J = 6.1, 4.0 Hz), 41.0 (t, J = 21.7 Hz), 26.6, 24.4.

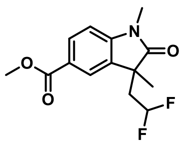

Methyl 3-(2,2-difluoroethyl)-1,3-dimethyl-2-oxoindoline-5-carboxylate (5g): The compound was according to the general procedure and was purified with silica gel chromatography (petroleum ether / ethyl acetate = 10:1) as white solid (22.9 mg, 81% yield). Known compound.[12] ^{1}H NMR (400 MHz, $CDCl_3$) δ 8.07 (dd, J = 8.2, 1.6 Hz, 1H), 7.91 (d, J = 1.5 Hz, 1H), 6.92 (d, J = 8.2 Hz, 1H), 5.58 (m, 1H), 3.92 (s, 3H), 3.26 (s, 3H), 2.55 (m, 1H), 2.42-2.25 (m, 1H), 1.44 (s, 3H). ^{19}F NMR (376 MHz, $CDCl_3$) δ -114.55 (m, 2F). ^{13}C NMR (101 MHz, $CDCl_3$) δ 179.4, 166.7, 147.0, 132.0, 131.2, 124.7, 124.1, 114.8 (t, J = 240.9 Hz), 108.0, 52.1, 44.4 (t, J = 5.1 Hz), 41.2 (t, J = 22.2 Hz), 26.6, 24.4.

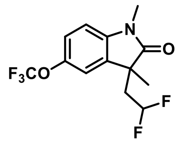

3-(2,2-Difluoroethyl)-1,3-dimethyl-5-(trifluoromethoxy)indolin-2-one (5h): The compound was according to the general procedure and was purified with silica gel chromatography (petroleum ether / ethyl acetate = 10:1) as colorless liquid (24.8 mg, 80% yield). Known compound.[13] ^{1}H NMR (400 MHz, $CDCl_3$) δ 7.19 (d, J = 8.5 Hz, 1H), 7.12 (d, J = 1.2 Hz, 1H), 6.86 (d, J = 8.5 Hz, 1H), 5.62 (m, 1H), 3.24 (s, 3H), 2.51 (m, 1H), 2.36-2.22 (m, 1H), 1.43 (s, 3H). ^{19}F NMR (376 MHz, $CDCl_3$) δ -58.42 (s, 3F) -114.25 (m, 2F). ^{13}C NMR (101 MHz, $CDCl_3$) δ 178.8, 144.9, 141.5, 133.6, 121.6, 120.5 (q, J = 256.9 Hz), 117.0, 114.8 (t, J = 240.9 Hz), 108.9, 44.9 (t, J = 5.1 Hz), 41.2 (t, J = 21.7 Hz), 26.5, 24.3.

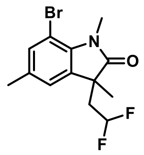

7-Bromo-3-(2,2-difluoroethyl)-1,3,5-trimethylindolin-2-one (5i): The compound was according to the general procedure and was purified with silica gel chromatography (petroleum ether / ethyl acetate = 15:1) as white solid (21.5 mg, 68% yield). ^{1}H NMR (400 MHz, $CDCl_3$) δ 7.23 (s, 1H), 6.94 (s, 1H), 5.77-5.46 (m, 1H), 3.57 (s, 3H), 2.49 (m, 2H), 2.39-2.03 (m, 5H), 1.38 (s, 3H). ^{19}F NMR (376 MHz, $CDCl_3$) δ -114.22 (m, 2F). ^{13}C NMR (101 MHz, $CDCl_3$) δ 179.5, 137.8, 135.1, 134.1, 133.9, 122.8, 114.9 (t, J = 240.4 Hz), 102.4, 44.4 (t, J = 5.6 Hz), 41.6 (t, J = 21.7 Hz), 29.9, 24.8, 20.5. HRMS (ESI): m/z [(M+H)$^+$] calcd for $C_{13}H_{15}{}^{79}BrF_2NO$, 318.0300. found, 318.0303.

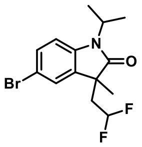

5-Bromo-3-(2,2-difluoroethyl)-1-isopropyl-3-methylindolin-2-one (5j): The compound was according to the general procedure and was purified with silica gel chromatography (petroleum ether / ethyl acetate = 12:1) as colorless liquid (29.1 mg, 88% yield). ^{1}H NMR (400 MHz, $CDCl_3$) δ 7.36 (dd, J = 8.4, 2.0 Hz, 1H), 7.31 (d, J = 1.9 Hz, 1H), 6.88 (d, J = 8.4 Hz, 1H), 5.51 (m, 1H), 4.56 (m, 1H), 2.47 (m, 1H), 2.21 (m, 1H), 1.42 (dd, J = 7.0, 4.5 Hz, 6H), 1.35 (s, 3H). ^{19}F NMR (376 MHz, $CDCl_3$) δ -114.64 (m, 2F). ^{13}C NMR (101 MHz, $CDCl_3$) δ 178.2, 140.7, 134.7, 131.0, 126.3, 114.9 (t, J = 241.4 Hz), 111.6, 44.4 (t, J = 5.1 Hz), 44.0, 41.4 (t, J = 21.7 Hz), 24.6, 19.2, 19.1. HRMS (ESI): m/z [(M+H)$^+$] calcd for $C_{14}H_{17}{}^{79}BrF_2NO$, 332.0456. found, 332.0461.

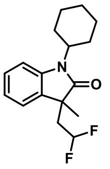

1-Cyclohexyl-3-(2,2-difluoroethyl)-3-methylindolin-2-one (5k): The compound was according to the general procedure and was purified with silica gel chromatography (petroleum ether / ethyl acetate = 12:1) as colorless liquid (24.8 mg, 85% yield). ^{1}H

NMR (400 MHz, $CDCl_3$) δ 7.29-7.24 (m, 1H), 7.24-7.19 (m, 1H), 7.11-7.02 (m, 2H), 5.50 m, 1H), 4.16 (m, 1H), 2.51 (m, 1H), 2.35-2.21 (m, 1H), 2.20-2.09 (m, 2H), 1.90 (d, J = 13.2 Hz, 2H), 1.78-1.72 (m, 3H), 1.48-1.37 (m, 5H), 1.32-1.23 (m, 1H). ^{19}F NMR (376 MHz, $CDCl_3$) δ -114.34 (m, 2F). ^{13}C NMR (101 MHz, $CDCl_3$) δ 179.0, 142.1, 132.4, 128.1, 123.0, 122.1, 115.2 (t, J = 240.9 Hz), 110.4, 52.3, 44.2 (t, J = 5.6 Hz), 41.6 (t, J = 22.2 Hz), 29.0, 28.9, 26.0, 26.0, 25.4, 24.7. HRMS (ESI): m/z [$(M+H)^+$] calcd for $C_{17}H_{22}F_2NO$, 294.1664. found, 294.1669.

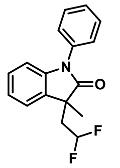

3-(2,2-Difluoroethyl)-3-methyl-1-phenylindolin-2-one (5l): The compound was according to the general procedure and was purified with silica gel chromatography (petroleum ether / ethyl acetate = 15:1) as white solid (22.4 mg, 78% yield). Known compound.[12] ^{1}H NMR (400 MHz, $CDCl_3$) δ 7.52 (dd, J = 10.7, 4.5 Hz, 2H), 7.45-7.36 (m, 3H), 7.29 (dd, J = 7.4, 0.6 Hz, 1H), 7.22 (dd, J = 7.8, 1.2 Hz, 1H), 7.12 (td, J = 7.6, 0.9 Hz, 1H), 6.84 (d, J = 7.9 Hz, 1H), 5.63 (m, 1H), 2.64 (m, 1H), 2.45-2.30 (m, 1H), 1.53 (s, 3H). ^{19}F NMR (376 MHz, $CDCl_3$) δ -114.64 (m, 2F). ^{13}C NMR (101 MHz, $CDCl_3$) δ 178.6, 143.0, 134.4, 131.7, 129.7, 128.5, 128.2, 126.6, 123.2, 123.1, 115.3 (t, J = 240.4 Hz), 109.8, 44.7 (t, J = 5.1 Hz), 41.8 (t, J = 26.8 Hz), 24.8.

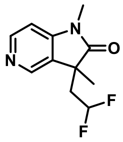

3-(2,2-Difluoroethyl)-1,3-dimethyl-1,3-dihydro-2H-pyrrolo[3,2-c]pyridin-2-one (5m): The compound was according to the general procedure with 10 mol% PC-5 and was purified with silica gel chromatography (petroleum ether / ethyl acetate = 4:1) as white solid (15.6 mg, 69% yield). ^{1}H NMR (400 MHz, $CDCl_3$) δ 8.52 (d, J = 4.9 Hz,

1H), 8.39 (s, 1H), 6.85 (t, J = 7.2 Hz, 1H), 5.65 (m, 1H), 3.24 (s, 3H), 2.55 (dt, J = 14.6, 8.5 Hz, 1H), 2.45-2.32 (m, 1H), 1.47 (s, 3H). ^{19}F NMR (376 MHz, $CDCl_3$) δ -114.55 (m, 2F). ^{13}C NMR (101 MHz, $CDCl_3$) δ 178.9, 150.4, 150.2, 143.1, 127.8, 114.7 (t, J = 241.4 Hz), 104.2, 43.4 (dd, J = 5.6, 4.5 Hz), 41.0 (t, J = 22.2 Hz), 26.5, 24.2. HRMS (ESI): m/z [$(M+H)^+$] calcd for $C_{11}H_{13}F_2N_2O$, 227.0990. found, 227.0995.

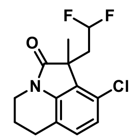

9-Chloro-1-(2,2-difluoroethyl)-1-methyl-5,6-dihydro-4H-pyrrolo[3,2,1-ij]quinolin-2(1H)-one (5n): The compound was according to the general procedure with 10 mol% PC-5 and was purified with silica gel chromatography (petroleum ether / ethyl acetate = 12:1) as colorless liquid (20.0 mg, 70% yield). ^{1}H NMR (400 MHz, $CDCl_3$) δ 7.00 (d, J = 8.2 Hz, 1H), 6.89 (d, J = 8.2 Hz, 1H), 5.50 (m, 1H), 3.75-3.68 (m, 2H), 2.81-2.74 (m, 2H), 2.73-2.50 (m, 2H), 2.05-1.97 (m, 2H), 1.52 (s, 3H). ^{19}F NMR (376 MHz, $CDCl_3$) δ -115.77 (m, 2F). ^{13}C NMR (101 MHz, $CDCl_3$) δ 177.4, 140.3, 128.7, 127.9, 126.5, 122.7, 119.1, 115.2 (t, J = 240.9 Hz), 47.0 (dd, J = 8.1, 3.0 Hz), 38.9 (t, J = 21.7 Hz), 24.4, 21.9, 20.9. HRMS (ESI): m/z [$(M+H)^+$] calcd for $C_{14}H_{15}{}^{35}ClF_2NO$, 286.0805. found, 286.0809.

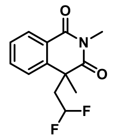

4-(2,2-Difluoroethyl)-2,4-dimethylisoquinoline-1,3(2H,4H)-dione (5o): The compound was according to the general procedure and was purified with silica gel chromatography (petroleum ether / ethyl acetate = 12:1) as white solid (19.0 mg, 72% yield). Known compound.[19] ^{1}H NMR (400 MHz, $CDCl_3$) δ 8.27 (dd, J = 7.9, 0.9 Hz, 1H), 7.66 (td, J = 7.9, 1.3 Hz, 1H), 7.49-7.41 (m, 2H), 5.51-5.20 (m, 1H), 3.38 (s, 3H), 3.04-2.92 (m, 1H), 2.53-2.41 (m, 1H), 1.64 (s, 3H). ^{19}F NMR (376 MHz, $CDCl_3$) δ -115.83 (m, 2F). ^{13}C NMR (101 MHz, $CDCl_3$) δ 175.2, 163.8, 141.3, 134.1, 129.4, 128.0,

125.3, 124.4, 115.0 (t, J = 241.4 Hz), 45.1 (t, J = 21.7 Hz), 43.8 (dd, J = 7.1, 3.0 Hz), 30.6, 27.4.

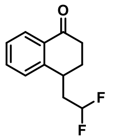

4-(2,2-Difluoroethyl)-3,4-dihydronaphthalen-1(2H)-one (5p): The compound was according to the general procedure with 10 mol% PC-5 and was purified with silica gel chromatography (petroleum ether / ethyl acetate = 25:1) as colorless liquid (10.8 mg, 51% yield). ^{1}H NMR (400 MHz, $CDCl_3$) δ 8.06 (d, J = 7.8 Hz, 1H), 7.53 (td, J = 7.6, 1.3 Hz, 1H), 7.37 (t, J = 7.6 Hz, 1H), 7.29 (d, J = 7.7 Hz, 1H), 5.93 (m, 1H), 3.37-3.26 (m, 1H), 2.80-2.62 (m, 2H), 2.38-2.10 (m, 4H). ^{19}F NMR (376 MHz, $CDCl_3$) δ -115.89 (m, 2F). ^{13}C NMR (101 MHz, $CDCl_3$) δ 197.3, 145.9, 133.9, 132.0, 128.02 127.8, 127.5, 116.3 (t, J = 240.4 Hz), 38.5 (t, J = 21.2 Hz), 34.5, 32.7 (t, J = 5.6 Hz), 27.2. HRMS (ESI): m/z [$(M+H)^+$] calcd for $C_{12}H_{13}F_2O$, 211.0929. found, 211.0931.

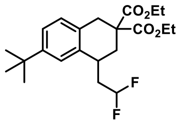

Diethyl 6-(tert-butyl)-4-(2,2-difluoroethyl)-3,4-dihydronaphthalene-2,2(1H)-dicarboxylate (5q): The compound was according to the general procedure and was purified with silica gel chromatography (petroleum ether / ethyl acetate = 15:1) as colorless liquid (26.5 mg, 67% yield). ^{1}H NMR (400 MHz, $CDCl_3$) δ 7.19-7.13 (m, 2H), 7.05 (d, J = 7.9 Hz, 1H), 5.97 (m, 1H), 4.20 (q, J = 7.1 Hz, 2H), 4.09 (m, 2H), 3.31-3.11 (m, 3H), 2.70 (dd, J = 13.6, 6.3 Hz, 1H), 2.52-2.37 (m, 1H), 2.12-1.92 (m, 2H), 1.29-1.23 (m, 12H), 1.11 (t, J = 7.1 Hz, 3H). ^{19}F NMR (376 MHz, $CDCl_3$) δ -115.00 (m, 2F). ^{13}C NMR (101 MHz, $CDCl_3$) δ 171.8, 170.6, 149.6, 136.1, 130.9, 128.8, 123.8, 123.4, 116.5 (t, J = 240.4 Hz), 61.7, 61.4, 53.7, 40.8 (t, J = 20.7 Hz), 34.7, 34.5, 34.5, 31.4, 30.9 (t, J = 5.1 Hz), 14.0, 13.9. HRMS (ESI): m/z [$(M+Na)^+$] calcd for $C_{22}H_{30}F_2NaO_4$, 419.2004. found, 419.2004.

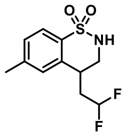

4-(2,2-Difluoroethyl)-6-methyl-3,4-dihydro-2H-benzo[e][1,2]thiazine 1,1-dioxide (5r): The compound was according to the general procedure and was purified with silica gel chromatography (petroleum ether / ethyl acetate = 7:1) as white solid (19.3 mg, 74% yield). ^{1}H NMR (400 MHz, $CDCl_3$) δ 7.69 (d, J = 8.1 Hz, 1H), 7.21 (d, J = 8.0 Hz, 1H), 7.06 (s, 1H), 5.97 (tt, J = 55.8, 3.9 Hz, 1H), 5.09 (dd, J = 8.8, 5.6 Hz, 1H), 4.07-3.93 (m, 1H), 3.69-3.57 (m, 1H), 3.23-3.12 (m, 1H), 2.40 (d, J = 13.2 Hz, 3H), 2.36-2.10 (m, 2H). ^{19}F NMR (376 MHz, $CDCl_3$) δ -115.74 (m, 2F). ^{13}C NMR (101 MHz, $CDCl_3$) δ 143.2, 137.8, 134.7, 129.4, 129.1, 124.5, 115.7 (t, J = 240.9 Hz), 45.5, 38.3 (t, J = 21.2 Hz), 31.6 (t, J = 4.0 Hz), 21.6. HRMS (ESI): m/z [$(M+H)^+$] calcd for $C_{11}H_{14}F_2NO_2S$, 262.0708. found, 262.0711.

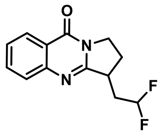

3-(2,2-Difluoroethyl)-2,3-dihydropyrrolo[2,1-b]quinazolin-9(1H)-one (5s): The compound was according to the general procedure and was purified with silica gel chromatography (petroleum ether / ethyl acetate = 5:1) as white solid (15.8 mg, 63% yield). Known compound.[17] ^{1}H NMR (400 MHz, $CDCl_3$) δ 8.28 (dd, J = 8.0, 1.2 Hz, 1H), 7.73 (m, 1H), 7.66 (d, J = 7.5 Hz, 1H), 7.51-7.43 (m, 1H), 6.44-6.14 (m, 1H), 4.36 (m, 1H), 4.03 – 3.92 (m, 1H), 3.53-3.42 (m, 1H), 2.842.71 (m, 1H), 2.62 (dt, J = 13.3, 7.3 Hz, 1H), 2.19-1.99 (m, 2H). ^{19}F NMR (376 MHz, $CDCl_3$) δ -116.26 (m, 2F). ^{13}C NMR (101 MHz, $CDCl_3$) δ 160.8, 159.7, 149.0, 134.2, 127.1, 126.5, 126.4, 120.8, 116.0 (t, J = 239.9 Hz), 44.8, 38.2 (t, J = 5.6 Hz), 36.3 (t, J = 21.7 Hz), 27.5.

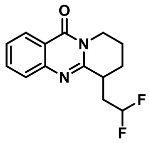

6-(2,2-Difluoroethyl)-6,7,8,9-tetrahydro-11H-pyrido[2,1-b]quinazolin-11-one (5t): The compound was according to the general procedure and was purified with silica gel

chromatography (petroleum ether / ethyl acetate = 5:1) as white solid (19.0 mg, 72% yield). Known compound.[17] ^{1}H NMR (400 MHz, $CDCl_3$) δ 8.26 (dd, J = 8.0, 0.9 Hz, 1H), 7.75-7.69 (m, 1H), 7.62 (d, J = 8.0 Hz, 1H), 7.45 (dd, J = 11.1, 4.0 Hz, 1H), 6.57-6.26 (m, 1H), 4.42 (dt, J = 14.0, 6.1 Hz, 1H), 3.92-3.83 (m, 1H), 3.12-3.04 (m, 1H), 2.77 m, 1H), 2.25-2.00 (m, 4H), 1.65 (m, 1H). ^{19}F NMR (376 MHz, $CDCl_3$) δ -115.80 (m, 2F). ^{13}C NMR (101 MHz, $CDCl_3$) δ 161.8, 156.0, 147.0, 134.1, 127.0, 126.7, 126.5, 120.4, 116.9 (t, J = 239.4 Hz), 40.8, 37.2 (t, J = 21.7 Hz), 35.2 (dd, J = 8.6, 4.0 Hz), 26.1, 20.8.

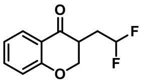

3-(2,2-Difluoroethyl)chroman-4-one (5u): The compound was according to the general procedure with 10 mol% PC-5 and was purified with silica gel chromatography (petroleum ether / ethyl acetate = 15:1) as colorless liquid (12.3 mg, 58% yield). Known compound.[22] ^{1}H NMR (400 MHz, $CDCl_3$) δ 7.89 (dd, J = 7.9, 1.7 Hz, 1H), 7.50 (m, 1H), 7.07-7.02 (m, 1H), 7.00-6.97 (m, 1H), 6.29-5.99 (m, 1H), 4.63 (dd, J = 11.4, 5.2 Hz, 1H), 4.24 (t, J = 11.7 Hz, 1H), 3.14-3.03 (m, 1H), 2.60-2.44 (m, 1H), 1.96-1.81 (m, 1H). ^{19}F NMR (376 MHz, $CDCl_3$) δ -115.00 (m, 2F). ^{13}C NMR (101 MHz, $CDCl_3$) δ 192.6, 161.6, 136.2, 127.4, 121.7, 120.3, 117.9, 116.1 (t, J = 240.4 Hz), 70.5, 40.5 (dd, J = 5.1, 3.0 Hz) 30.4 (t, J = 22.2 Hz).

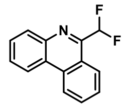

6-(Difluoromethyl)phenanthridine (5v): The compound was according to the general procedure and was purified with silica gel chromatography (petroleum ether / ethyl acetate = 10:1) as white solid (21.5 mg, 94% yield). Known compound.[23] ^{1}H NMR (400 MHz, $CDCl_3$) δ 8.64 (d, J = 8.4 Hz, 1H), 8.61-8.50 (m, 2H), 8.25-8.13 (m, 1H), 7.90-7.83 (m, 1H), 7.83-7.63 (m, 3H), 7.02 (t, J = 54.4 Hz, 1H). ^{19}F NMR (376 MHz, $CDCl_3$) δ -110.56 (dd, J = 52.6, 3.8 Hz, 2F). ^{13}C NMR (101 MHz, $CDCl_3$) δ151.4 (t, J = 26.8

Hz), 142.5, 133.8, 131.2, 130.6, 129.1, 128.6, 127.8, 126.4 (t, J = 4.0 Hz), 124.9, 122.4, 122.1, 118.4 (t, J = 244.4 Hz).

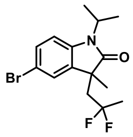

5-Bromo-3-(2,2-difluoropropyl)-1-isopropyl-3-methylindolin-2-one (5w): The compound was according to the general procedure and was purified with silica gel chromatography (petroleum ether / ethyl acetate = 12:1) as white solid (27.5 mg, 80% yield). ^{1}H NMR (400 MHz, $CDCl_3$) δ 7.36 (dd, J = 4.4, 2.4 Hz, 2H), 6.90 (d, J = 8.9 Hz, 1H), 4.61 (dt, J = 14.1, 7.0 Hz, 1H), 2.65 (m, 1H), 2.38 (m, 1H), 1.45 (m, 6H), 1.40 (d, J = 18.7 Hz, 3H), 1.33 (s, 3H). ^{19}F NMR (376 MHz, $CDCl_3$) δ -86.77 (m, 2F). ^{13}C NMR (101 MHz, $CDCl_3$) δ 178.7, 140.6, 135.1, 130.5, 127.1, 122.6 (t, J = 240.9 Hz), 114.6, 111.3, 44.9 (t, J = 2.5 Hz), 44.6 (t, J = 25.7 Hz), 43.9, 26.2, 24.5 (t, J = 27.3 Hz), 19.2, 18.9. HRMS (ESI): m/z [$(M+H)^+$] calcd for $C_{15}H_{19}{}^{79}BrF_2NO$, 346.0613. found, 346.0615.

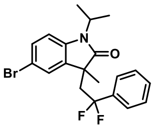

5-Bromo-3-(2,2-difluoro-2-phenylethyl)-1-isopropyl-3-methylindolin-2-one (5x): The compound was according to the general procedure and was purified with silica gel chromatography (petroleum ether / ethyl acetate = 12:1) as white solid (34.6 mg, 85% yield). ^{1}H NMR (400 MHz, $CDCl_3$) δ 7.34 (d, J = 7.1 Hz, 1H), 7.29-7.24 (m, 3H), 7.19 (d, J = 7.6 Hz, 2H), 7.11 (d, J = 1.9 Hz, 1H), 6.84 (d, J = 8.4 Hz, 1H), 4.59 (dq, J = 14.1, 7.0 Hz, 1H), 3.06-2.93 (m, 1H), 2.62 (dt, J = 19.2, 15.1 Hz, 1H), 1.43 (d, J = 7.1 Hz, 6H), 1.32 (s, 3H). ^{19}F NMR (376 MHz, $CDCl_3$) δ -88.67 (m, 1F), -95.60 (m, 1F). ^{13}C NMR (101 MHz, $CDCl_3$) δ 178.5, 140.7, 136.8 (t, J = 26.3 Hz), 134.5, 130.4, 129.8, 128.2, 127.3, 124.6 (t, J = 6.6 Hz), 121.5 (t, J = 24.9 Hz), 114.5, 111.1, 46.0 (dd, J = 28.8, 26.8 Hz), 45.0 (d, J = 4.0 Hz), 43.9, 26.2, 19.2, 18.9. HRMS (ESI): m/z [$(M+Na)^+$] calcd for $C_{20}H_{20}{}^{79}BrF_2NNaO$, 430.0589. found, 430.0592.

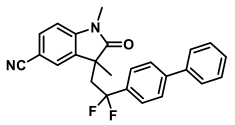

3-(2-([1,1'-Biphenyl]-4-yl)-2,2-difluoroethyl)-1,3-dimethyl-2-oxoindoline-5-carbonitrile (5y): The compound was according to the general procedure and was purified with silica gel chromatography (petroleum ether / ethyl acetate = 7:1) as colorless liquid (29.8 mg, 75% yield). ^{1}H NMR (400 MHz, $CDCl_3$) δ 7.60-7.54 (m, 3H), 7.51-7.43 (m, 5H), 7.39 (d, J = 7.0 Hz, 1H), 7.17 (d, J = 8.2 Hz, 2H), 6.81 (d, J = 8.1 Hz, 1H), 3.12-2.99 (m, 4H), 2.83-2.71 (m, 1H), 1.39 (s, 3H). ^{19}F NMR (376 MHz, $CDCl_3$) δ -88.91 (m, 1F), -92.29 (m, 1F). ^{13}C NMR (101 MHz, $CDCl_3$) δ 178.9, 146.9, 143.0, 140.0, 134.6 (t, J = 26.3 Hz), 133.3, 132.9, 128.9, 127.9, 127.5, 127.2, 126.9, 125.4 (t, J = 6.6 Hz), 121.3 (t, J = 245.4 Hz), 119.2, 108.5, 105.5, 45.7 (t, J = 28.3 Hz), 45.0 (t, J = 1.5 Hz), 26.4, 26.0. HRMS (ESI): m/z [$(M+Na)^+$] calcd for $C_{25}H_{20}F_2N_2NaO$, 425.1436. found, 425.1433.

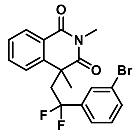

4-(2-(3-Bromophenyl)-2,2-difluoroethyl)-2,4-dimethylisoquinoline-1,3(2H,4H)-dione (5z): The compound was according to the general procedure and was purified with silica gel chromatography (petroleum ether / ethyl acetate = 8:1) as colorless liquid (30.9 mg, 76% yield). ^{1}H NMR (400 MHz, $CDCl_3$) δ 8.25 (dd, J = 7.9, 1.1 Hz, 1H), 7.57 (td, J = 7.8, 1.4 Hz, 1H), 7.49-7.41 (m, 2H), 7.37 (d, J = 7.9 Hz, 1H), 7.22 (s, 1H), 7.15 (t, J = 7.8 Hz, 1H), 7.08 (d, J = 7.9 Hz, 1H), 3.45-3.34 (m, 1H), 3.28 (s, 3H), 2.85 (m, 1H), 1.61 (s, 3H). ^{19}F NMR (376 MHz, $CDCl_3$) δ -91.09 (m, 2F). ^{13}C NMR (101 MHz, $CDCl_3$) δ 175.2, 163.8, 141.2, 138.3 (t, J = 26.8 Hz), 133.4, 133.0, 129.9, 128.9, 128.2 (t, J = 6.6 Hz), 127.7, 126.2, 124.4, 123.6 (t, J = 6.1 Hz), 122.4, 120.6 (t, J = 246.9 Hz), 49.4 (t, J = 26.3 Hz), 44.1 (t, J = 2.0 Hz), 31.8, 27.3. HRMS (ESI): m/z [$(M+H)^+$] calcd for $C_{19}H_{17}{}^{79}BrF_2NO_2$, 408.0405. found, 408.0409.

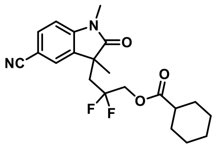

3-(5-Cyano-1,3-dimethyl-2-oxoindolin-3-yl)-2,2-difluoropropyl cyclohexanecarboxylate (5aa): The compound was according to the general procedure and was purified with silica gel chromatography (petroleum ether / ethyl acetate = 8:1) as colorless liquid (28.1 mg, 72% yield). ^{1}H NMR (400 MHz, $CDCl_3$) δ 7.63 (dd, J = 8.2, 1.6 Hz, 1H), 7.52 (s, 1H), 6.95 (d, J = 8.2 Hz, 1H), 4.13-3.92 (m, 2H), 3.26 (s, 3H), 2.70 (m, 1H), 2.57-2.43 (m, 1H), 2.37 (m, 1H), 1.92 (d, J = 12.7 Hz, 2H), 1.77 (dd, J = 7.3, 5.4 Hz, 2H), 1.69-1.63 (m, 1H), 1.50-1.38 (m, 5H), 1.34-1.24 (m, 3H). ^{19}F NMR (376 MHz, $CDCl_3$) δ -102.81 (m, 2F). ^{13}C NMR (101 MHz, $CDCl_3$) δ 179.0, 174.7, 146.8, 133.5, 133.3, 127.1, 120.3 (t, J = 245.4 Hz), 119.2, 108.8, 105.6, 63.7 (t, J = 33.3 Hz), 44.4 (d, J = 3.0 Hz), 42.7, 40.5 (t, J = 22.7 Hz), 28.9, 28.8, 26.6, 25.6, 25.6, 25.3. HRMS (ESI): m/z [$(M+H)^+$] calcd for $C_{21}H_{25}F_2N_2O_3$, 391.1828. found, 391.1832.

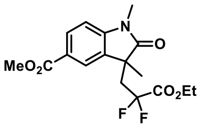

Methyl 3-(3-ethoxy-2,2-difluoro-3-oxopropyl)-1,3-dimethyl-2-oxoindoline-5-carboxylate (5ab): The compound was according to the general procedure and was purified with silica gel chromatography (petroleum ether / ethyl acetate = 8:1) as pink solid (29.5 mg, 83% yield). ^{1}H NMR (400 MHz, $CDCl_3$) δ 8.05 (dd, J = 8.2, 1.6 Hz, 1H), 7.87 (d, J = 1.5 Hz, 1H), 6.91 (d, J = 8.2 Hz, 1H), 4.06-3.98 (m, 2H), 3.91 (s, 3H), 3.25 (d, J = 10.0 Hz, 3H), 2.93-2.77 (m, 2H), 1.42 (s, 3H), 1.24 (t, J = 7.2 Hz, 3H). ^{19}F NMR (376 MHz, $CDCl_3$) δ -99.64 (dt, J = 135.4, 13.6 Hz, 1F), -105.45 (m, 1F). ^{13}C NMR (101 MHz, $CDCl_3$) δ 179.1, 166.7, 163.2 (t, J = 32.8 Hz), 147.5, 131.3, 130.9, 124.9, 124.2, 114.3 (dd, J = 254.5, 250.5 Hz), 108.0, 63.0, 52.1, 44.2 (d, J = 5.1 Hz), 41.1 (dd, J = 24.2, 22.2 Hz), 26.6, 25.4, 13.7. HRMS (ESI): m/z [$(M+H)^+$] calcd for $C_{17}H_{20}F_2NO_5$, 356.1304. found, 356.1308.

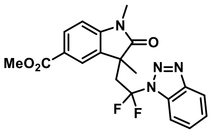

Methyl 3-(2-(1H-benzo[d][1,2,3]triazol-1-yl)-2,2-difluoroethyl)-1,3-dimethyl-2-oxoindoline-5-carboxylate (5ac): The compound was according to the general procedure and was purified with silica gel chromatography (petroleum ether / ethyl acetate = 3:1) as white solid (28.0 mg, 70% yield). ^{1}H NMR (400 MHz, $CDCl_3$) δ 7.98 (d, J = 8.2 Hz, 1H), 7.85 (dd, J = 8.2, 1.5 Hz, 1H), 7.71 (d, J = 1.0 Hz, 1H), 7.44-7.33 (m, 3H), 6.77 (d, J = 8.2 Hz, 1H), 3.88 (s, 3H), 3.75-3.56 (m, 2H), 3.24 (s, 3H), 1.52 (s, 3H). ^{19}F NMR (376 MHz, $CDCl_3$) δ -72.60 (m, 1F), -79.28 (dt, J = 210.6, 15.0 Hz, 1F). ^{13}C NMR (101 MHz, $CDCl_3$) δ 178.9, 166.4, 146.8, 145.8, 130.9, 130.7, 130.5, 129.0, 125.0, 124.5, 124.4, 120.6 (t, J = 258.1 Hz), 120.1, 111.1 (dd, J = 6.1, 3.0 Hz), 107.8, 52.0, 44.6 (d, J = 4.0 Hz), 42.5 (dd, J = 26.3, 23.2 Hz), 26.6, 25.7. HRMS (ESI): m/z $[(M+H)^+]$ calcd for $C_{20}H_{19}F_2N_4O_3$, 401.1420. found, 401.1422.

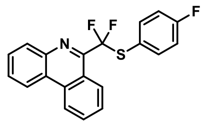

6-(Difluoro((4-fluorophenyl)thio)methyl)phenanthridine (5ad): The compound was according to the general procedure and was purified with silica gel chromatography (petroleum ether / ethyl acetate = 12:1) as white solid (21.3 mg, 60% yield). Known compound.[41] ^{1}H NMR (400 MHz, $CDCl_3$) δ 8.66 (d, J = 8.4 Hz, 1H), 8.56 (dd, J = 11.0, 9.2 Hz, 2H), 8.30-8.24 (m, 1H), 7.89-7.84 (m, 1H), 7.77 (m, 4H), 7.72-7.65 (m, 1H), 7.13 (t, J = 8.7 Hz, 2H). ^{19}F NMR (376 MHz, $CDCl_3$) δ -66.23 (s, 2F), -110.78 (m, 1F). ^{13}C NMR (101 MHz, $CDCl_3$) δ 164.2 (d, J = 251.5 Hz), 150.9 (t, J = 28.3 Hz), 141.7, 139.3 (d, J = 9.0 Hz), 134.1, 131.1, 130.9, 129.5 (t, J = 279.8 Hz), 129.1, 128.9, 127.7, 126.8 (t, J = 5.6 Hz), 125.0, 122.7 (d, J = 4.0 Hz), 122.5, 122.0, 121.9, 116.2 (d, J = 22.2 Hz).

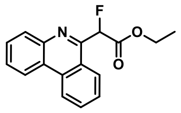

Ethyl 2-fluoro-2-(phenanthridin-6-yl)acetate (5ae): The compound was according to the general procedure and was purified with silica gel chromatography (petroleum ether / ethyl acetate = 12:1) as white solid (10.6 mg, 38% yield). Known compound.[42] ^{1}H NMR (400 MHz, $CDCl_3$) δ 8.64 (d, J = 8.3 Hz, 1H), 8.55 (d, J = 8.0 Hz, 1H), 8.37 (d, J = 8.2 Hz, 1H), 8.18 (d, J = 7.9 Hz, 1H), 7.85 (t, J = 7.7 Hz, 1H), 7.78-7.65 (m, 3H), 6.49 (d, J = 48.0 Hz, 1H), 4.42-4.24 (m, 2H), 1.23 (t, J = 7.0 Hz, 3H). ^{19}F NMR (376 MHz, $CDCl_3$) δ -180.72 (d, J = 45.1 Hz, 1F). ^{13}C NMR (101 MHz, $CDCl_3$) δ 168.1 (d, J = 25.3 Hz), 152.9 (d, J = 20.2 Hz), 143.0, 133.5, 131.0, 130.7, 128.9, 128.2, 127.9, 126.0 (d, J = 5.1 Hz), 124.3 (d, J = 47.5 Hz), 122.5, 122.0, 113.5, 91.3 (d, J = 187,9 Hz), 62.1, 14.1.

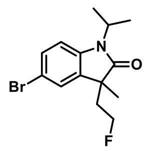

5-Bromo-3-(2-fluoroethyl)-1-isopropyl-3-methylindolin-2-one (5af): The compound was according to the general procedure with 10 mol% PC-5 and was purified with silica gel chromatography (petroleum ether / ethyl acetate = 15:1) as colorless liquid (25.3 mg, 81% yield). ^{1}H NMR (400 MHz, $CDCl_3$) δ 7.37 (dd, J = 8.4, 2.0 Hz, 1H), 7.30 (d, J = 2.0 Hz, 1H), 6.90 (d, J = 8.4 Hz, 1H), 4.60 (dt, J = 14.1, 7.0 Hz, 1H), 4.37-4.10 (m, 2H), 2.50-2.36 (m, 1H), 2.04 (m, 1H), 1.45 (m, 6H), 1.38 (s, 3H). ^{19}F NMR (376 MHz, $CDCl_3$) δ -218.91 (m, 1F). ^{13}C NMR (101 MHz, $CDCl_3$) δ 179.1, 141.0, 135.6, 130.7, 126.3, 114.8, 111.3, 80.5 (d, J = 166.7 Hz), 46.0 (d, J = 4.0 Hz), 43.8, 38.3 (d, J = 19.2 Hz), 24.3, 19.2, 19.1. HRMS (ESI): m/z [$(M+H)^+$] calcd for $C_{14}H_{18}{}^{79}BrFNO$, 314.0550. found, 314.0554.

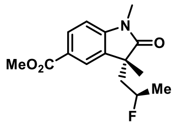

(±) Methyl (R)-3-((R)-2-fluoropropyl)-1,3-dimethyl-2-oxoindoline-5-carboxylate (5ag-1): The compound was according to the general procedure with 10 mol% PC-5 and was purified with silica gel chromatography (petroleum ether / ethyl acetate = 15:1) as white solid (10.4 mg, 36% yield). ^{1}H NMR (400 MHz, $CDCl_3$) δ 8.02 (dd, J = 8.2, 1.7 Hz, 1H), 7.90 (d, J = 1.6 Hz, 1H), 6.88 (d, J = 8.2 Hz, 1H), 4.55-4.36 (m, 1H), 3.90 (s, 3H), 3.25 (s, 3H), 2.29 (td, J = 14.3, 8.9 Hz, 1H), 2.14-2.00 (m, 1H), 1.40 (s, 3H), 1.18 (dd, J = 23.9, 6.2 Hz, 3H). ^{19}F NMR (376 MHz, $CDCl_3$) δ -170.53 (m, 1F). ^{13}C NMR (101 MHz, $CDCl_3$) δ 180.4, 166.9, 146.9, 133.8, 130.6, 124.6, 124.3, 107.7, 88.2 (d, J = 166.7 Hz), 52.0, 47.0 (d, J = 2.0 Hz), 44.3 (d, J = 20.2 Hz), 26.4, 24.8, 21.6 (d, J = 23.2 Hz). HRMS (ESI): m/z [$(M+H)^+$] calcd for $C_{15}H_{19}FNO_3$, 280.1343. found, 280.1340.

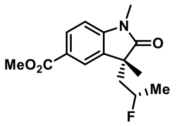

(±) Methyl (R)-3-((S)-2-fluoropropyl)-1,3-dimethyl-2-oxoindoline-5-carboxylate (5ag-2): The compound was purified with silica gel chromatography (petroleum ether / ethyl acetate = 15:1) as white solid (10.6 mg, 38% yield). ^{1}H NMR (400 MHz, $CDCl_3$) δ 8.03 (dd, J = 8.2, 1.6 Hz, 1H), 7.84 (d, J = 1.4 Hz, 1H), 6.88 (d, J = 8.2 Hz, 1H), 4.36-4.19 (m, 1H), 3.90 (s, 3H), 3.22 (s, 3H), 2.42 (dt, J = 14.7, 10.4 Hz, 1H), 1.94 (m, 1H), 1.39 (s, 3H), 1.19 (dd, J = 23.9, 6.2 Hz, 3H). ^{19}F NMR (376 MHz, $CDCl_3$) δ -174.26 (m, 1F). ^{13}C NMR (101 MHz, $CDCl_3$) δ 180.8, 167.0, 147.8, 132.8, 130.9, 124.2, 124.0, 107.7, 87.9 (d, J = 166.7 Hz), 52.0, 46.1, 44.8 (d, J = 20.2 Hz), 26.5, 24.4, 21.2 (d, J = 22.2 Hz). HRMS (ESI): m/z [$(M+H)^+$] calcd for $C_{15}H_{19}FNO_3$, 280.1343. found, 280.1345.

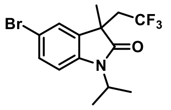

5-Bromo-1-isopropyl-3-methyl-3-(2,2,2-trifluoroethyl)indolin-2-one (5ah): The compound was according to the general procedure and was according to the general

procedure with **1ba** (0.3 mmol). The product was purified with silica gel chromatography (petroleum ether / ethyl acetate = 15:1) as white solid (31.3 mg, 90% yield). ^{1}H NMR (400 MHz, $CDCl_3$) δ 7.36 (dd, J = 8.4, 2.0 Hz, 1H), 7.33 (d, J = 1.6 Hz, 1H), 6.89 (d, J = 8.4 Hz, 1H), 4.56 (m, 1H), 2.87-2.78 (m, 1H), 2.61-2.53 (m, 1H), 1.42 (m, 6H), 1.35 (s, 3H). ^{19}F NMR (376 MHz, $CDCl_3$) δ -62.03 (t, J = 11.3 Hz, 3F). ^{13}C NMR (101 MHz, $CDCl_3$) δ 177.5, 140.6, 133.6, 131.1, 126.9 (q, J = 2.0 Hz), 126.5 (q, J = 279.8 Hz), 114.8, 111.5, 44.2, 40.7 (q, J = 28.6 Hz), 25.2, 19.1, 18.9. HRMS (ESI): m/z [$(M+H)^+$] calcd for $C_{14}H_{16}{}^{79}BrF_3NO$, 350.0362. found, 350.0361.

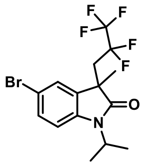

5-Bromo-1-isopropyl-3-methyl-3-(2,2,3,3,3-pentafluoropropyl)indolin-2-one (5ai): The compound was according to the general procedure with **1bf** (0.3 mmol) and was purified with silica gel chromatography (petroleum ether / ethyl acetate = 15:1) as white solid (33.5 mg, 84% yield). ^{1}H NMR (400 MHz, $CDCl_3$) δ 7.45-7.26 (m, 2H), 6.90 (d, J = 8.1 Hz, 1H), 4.58 (m, 1H), 2.79 (dd, J = 34.1, 15.3 Hz, 1H), 2.48 (m, 1H), 1.43 (m, 6H), 1.37 (s, 3H). ^{19}F NMR (376 MHz, $CDCl_3$) δ -86.35 (s, 3F), -115.60 (m, 2F). ^{13}C NMR (101 MHz, $CDCl_3$) δ 177.6, 140.6, 133.8, 131.1, 127.0 (d, J = 2.0 Hz), 118.5 (qt, J = 286.8, 35.9 Hz), 114.8, 114.7 (tq, J = 256.5, 38.4 Hz), 111.6, 44.2, 44.0, 36.9 (t, J = 20.2 Hz), 25.9, 19.1, 18.9. HRMS (ESI): m/z [$(M+H)^+$] calcd for $C_{15}H_{16}{}^{79}BrF_5NO$, 400.0330. found, 400.0332.

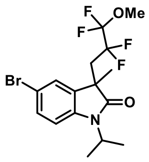

5-Bromo-1-isopropyl-3-methyl-3-(2,2,3,3-tetrafluoro-3-methoxypropyl)indolin-2-one (5aj):

The compound was according to the general procedure with **1bd** (0.3 mmol) and was purified with silica gel chromatography (petroleum ether / ethyl acetate = 12:1) as white

solid (32.0 mg, 78% yield). ^{1}H NMR (400 MHz, $CDCl_3$) δ 7.38-7.31 (m, 2H), 6.88 (d, J = 9.0 Hz, 1H), 4.59 (m, 1H), 3.59 (s, 3H), 2.76 (m, 1H), 2.52-2.37 (m, 1H), 1.42 (m, 6H), 1.34 (s, 3H). ^{19}F NMR (376 MHz, $CDCl_3$) δ -95.27 (m, 2F), -114.98 (m, 2F). ^{13}C NMR (101 MHz, $CDCl_3$) δ 178.08, 140.6, 134.5, 130.7, 127.1 (d, J = 2.0 Hz), 118.2 (tt, J = 272.7, 32.3 Hz), 116.0 (tt, J = 255.5, 36.9 Hz), 114.6, 111.4, 51.0 (t, J = 6.6 Hz), 44.1 (d, J = 1.0 Hz), 44.0, 37.5 (t, J = 20.7 Hz), 25.9, 19.2, 18.9. HRMS (ESI): m/z [$(M+Na)^+$] calcd for $C_{16}H_{18}{}^{79}BrF_4NNaO_2$, 434.0349. found, 434.0352.

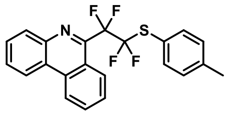

6-(1,1,2,2-Tetrafluoro-2-(p-tolylthio)ethyl)phenanthridine (5ak): The compound was according to the general procedure with 10 mol% PC-5 and **1be** (0.3 mmol) and was purified with silica gel chromatography (petroleum ether / ethyl acetate = 20:1) as white solid (28.1 mg, 70% yield). ^{1}H NMR (400 MHz, $CDCl_3$) δ 8.65 (d, J = 8.1 Hz, 1H), 8.53 (dd, J = 17.9, 7.7 Hz, 2H), 8.31 (d, J = 7.1 Hz, 1H), 7.88-7.83 (m, 1H), 7.80-7.67 (m, 3H), 7.54 (d, J = 7.5 Hz, 2H), 7.16 (d, J = 7.3 Hz, 2H), 2.34 (s, 3H). ^{19}F NMR (376 MHz, $CDCl_3$) δ -86.21 (t, J = 7.5 Hz, 2F), -104.02 (t, J = 7.5 Hz, 2F). ^{13}C NMR (101 MHz, $CDCl_3$) δ 148.20 (t, J = 25.8 Hz), 141.7, 140.6, 137.1, 133.9, 131.1, 131.0, 129.9, 129.2, 129.1, 127.8, 126.8 (t, J = 6.6 Hz), 124.8, 124.1 (tt, J = 290.9, 33.8 Hz), 123. 1, 122.4, 122.0, 121.9 (t, J = 3.0 Hz), 116.4 (tt, J = 258.6, 31.3 Hz), 21.3. HRMS (ESI): m/z [$(M+H)^+$] calcd for $C_{22}H_{16}F_4NS$, 402.0934. found, 402.0931.

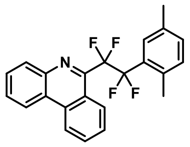

6-(2-(2,5-Dimethylphenyl)-1,1,2,2-tetrafluoroethyl)phenanthridine (5al): The compound was according to the general procedure with **1bb** (0.3 mmol) and was purified with silica gel chromatography (petroleum ether / ethyl acetate = 15:1) as white solid (33.5 mg, 84% yield). ^{1}H NMR (400 MHz, $CDCl_3$) δ 8.66 (d, J = 8.3 Hz, 1H), 8.60 – 8.54 (m, 1H), 8.50 (d, J = 8.5 Hz, 1H), 8.29 – 8.16 (m, 1H), 7.85 (t, J = 7.7 Hz,

1H), 7.81 – 7.71 (m, 2H), 7.68 (dd, J = 11.4, 4.1 Hz, 1H), 7.38 (s, 1H), 7.16 (d, J = 8.0 Hz, 1H), 7.10 (d, J = 7.8 Hz, 1H), 2.46 (d, J = 2.8 Hz, 3H), 2.28 (s, 3H). δ -104.40 (s, 2F), -105.87 (s, 2F). ^{13}C NMR (101 MHz, $CDCl_3$) δ 148.7 (t, J = 24.2 Hz), 142.1, 135.1, 134.8 (t, J = 2.0 Hz), 133.8, 132.2, 131.6, 131.1, 130.8, 129.4 (t, J = 8.6 Hz), 129.0, 128.8, 127.6, 127.2 (t, J = 7.6 Hz), 124.7, 123.8, 122.3, 122.0, 118.9 (tt, J = 256.5, 35.4 Hz), 116.6 (tt, J = 256.5, 37.9 Hz), 20.8, 20.4 (t, J = 3.5 Hz). HRMS (ESI): m/z [$(M+Na)^+$] calcd for $C_{23}H_{17}F_4NNa$, 406.1189. found, 406.1187.

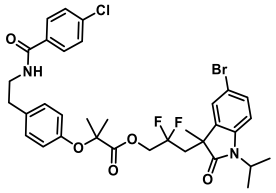

3-(5-Bromo-1-isopropyl-3-methyl-2-oxoindolin-3-yl)-2,2-difluoropropyl 2-(4-(2-(4-chlorobenzamido)ethyl)phenoxy)-2-methylpropanoate (5am): The compound was according to the general procedure with 10 mol% PC-5, **1r** (0.1 mmol) and **5l** (0.2 mmol) and was purified with silica gel chromatography (petroleum ether / ethyl acetate = 2:1) as white solid (47.2 mg, 67% yield). ^{1}H NMR (400 MHz, $CDCl_3$) δ 7.75-7.56 (m, 2H), 7.33 (m, 4H), 7.10 (d, J = 8.5 Hz, 2H), 6.88 (d, J = 8.4 Hz, 1H), 6.79 (dd, J = 8.9, 2.3 Hz, 2H), 6.58 (s, 1H), 4.54 (m, 1H), 4.21-4.06 (m, 1H), 3.95 (dd, J = 25.1, 11.6 Hz, 1H), 3.64 (m, 2H), 3.01-2.76 (m, 2H), 2.47-2.37 (m, 1H), 2.26-2.17 (m, 1H), 1.62 (s, 6H), 1.41 (dd, J = 7.0, 5.2 Hz, 6H), 1.24 (s, 3H). ^{19}F NMR (376 MHz, $CDCl_3$) δ -102.04 (m, 2F). ^{13}C NMR (101 MHz, $CDCl_3$) δ 178.3, 173.0, 166.5, 154.0, 140.5, 137.5, 134.6, 133.0, 132.7, 130.8, 129.7, 128.7, 128.4, 126.9, 120.1 (t, J = 245.9 Hz), 119.0, 114.6, 111.5, 79.0, 64.7 (t, J = 33.8 Hz), 44.3 (d, J = 4.0 Hz), 44.0, 41.4, 40.4 (t, J = 22.7 Hz), 34.7, 25.9, 25.7, 25.1, 19.1, 18.9. HRMS (ESI): m/z [$(M+Na)^+$] calcd for $C_{34}H_{36}{}^{79}Br^{35}ClF_2N_2NaO_5$, 727.1356. found, 727.1347.

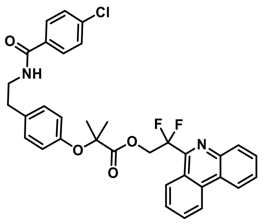

2,2-Difluoro-2-(phenanthridin-6-yl)ethyl 2-(4-(2-(4-chlorobenzamido)ethyl)phenoxy)-2-methylpropanoate (5an): The compound was according to the general procedure with **1r** (0.1 mmol) and **5v** (0.2 mmol) and was purified with silica gel chromatography (petroleum ether / ethyl acetate = 2:1) as white solid (42.7 mg, 71% yield). ^{1}H NMR (400 MHz, $CDCl_3$) δ 8.62 (d, J = 8.3 Hz, 1H), 8.59-8.49 (m, 2H), 8.13-8.04 (m, 1H), 7.84 (t, J = 7.7 Hz, 1H), 7.71 (m, 3H), 7.57 (d, J = 8.5 Hz, 2H), 7.28 (d, J = 8.5 Hz, 2H), 7.03 (d, J = 8.5 Hz, 2H), 6.87 (d, J = 8.5 Hz, 2H), 6.29 (t, J = 5.5 Hz, 1H), 5.35 (t, J = 14.1 Hz, 2H), 3.60 (q, J = 6.7 Hz, 2H), 2.81 (t, J = 6.9 Hz, 2H), 1.65 (s, 6H). ^{19}F NMR (376 MHz, $CDCl_3$) δ -98.61 (td, J = 15.0, 1.9 Hz, 2F). ^{13}C NMR (101 MHz, $CDCl_3$) δ 173.7, 166.4, 154.1, 150.6 (t, J = 29.8 Hz), 141.6, 137.5, 133.8, 133.0, 132.7, 131.1, 130.7, 129.5, 129.0, 128.8, 128.7, 128.3, 127.7, 126.7 (t, J = 5.6 Hz), 124.8, 122.4, 122.06, 120.1, 119.8 (t, J = 245.9 Hz), 79.5, 64.5 (t, J = 25.8 Hz), 41.3, 34.7, 25.5. HRMS (ESI): m/z [$(M+H)^+$] calcd for $C_{34}H_{30}{}^{35}ClF_2N_2O_4$, 603.1857. found, 603.1854.

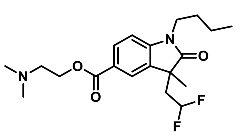

2-(Dimethylamino)ethyl 1-butyl-3-(2,2-difluoroethyl)-3-methyl-2-oxoindoline-5-carboxylate (5ao): The compound was according to the general procedure and was purified with silica gel chromatography (petroleum ether / ethyl acetate = 2:1) as white solid (19.7 mg, 52% yield). ^{1}H NMR (400 MHz, $CDCl_3$) δ 8.06 (dd, J = 8.3, 1.6 Hz, 1H), 7.93 (d, J = 1.4 Hz, 1H), 6.92 (d, J = 8.3 Hz, 1H), 5.56 (m, 1H), 4.46 (t, J = 5.8 Hz, 2H), 3.74 (m, 2H), 2.78 (t, J = 5.8 Hz, 2H), 2.64-2.52 (m, 1H), 2.39 (s, 7H), 1.66 (dd, J = 15.0, 7.4 Hz, 2H), 1.48-1.35 (m, 5H), 0.96 (t, J = 7.3 Hz, 3H). ^{19}F NMR (376 MHz, $CDCl_3$) δ -114.55 (m, 2F). ^{13}C NMR (101 MHz, $CDCl_3$) δ 179.3, 166.2, 146.7, 132.1,

131.2, 124.4, 114.9 (t, J = 240.9 Hz), 108.2, 62.7, 57.8, 45.7, 44.3 (t, J = 5.1 Hz), 41.1 (t, J = 22.2 Hz), 40.0, 29.1, 24.7, 20.0, 13.7. HRMS (ESI): m/z [(M+H)$^+$] calcd for $C_{20}H_{29}F_2N_2O_3$, 383.2141. found, 383.2144.

5. Continuous-flow synthesis of fluorinated-compounds

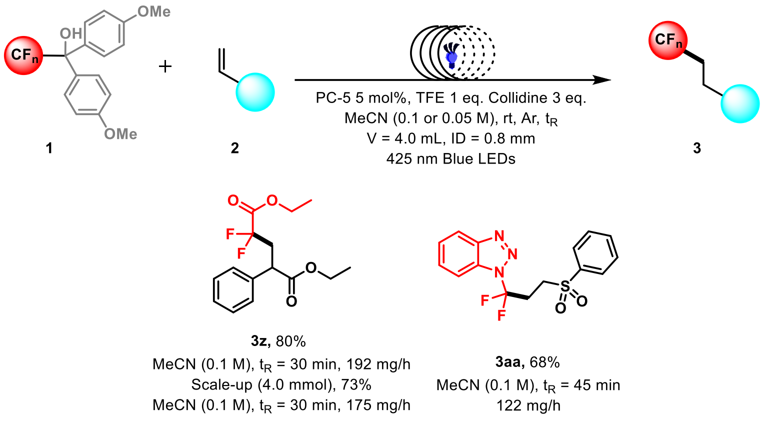

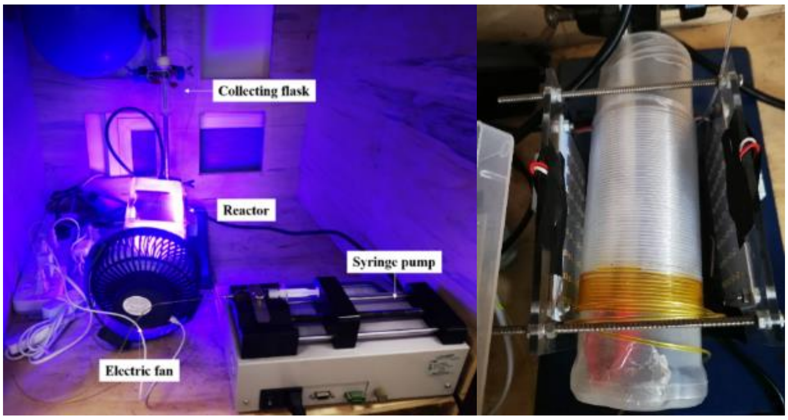

Figure S15. The continuous-flow photoreactor

To an 8 mL vial equipped with a stir bar was added fluorinated alcohol **1** (0.8 mmol, 2.0 equiv.), PC-5 (13.0 mg, 0.02 mmol, 5 mol%) and electron-withdrawing alkene **2** (0.4 mmol, 1.0 equiv.). The vial was sealed, evacuated and backfilled with Argon three times, then TFE (28 μL, 0.4 mmol, 1.0 equiv.) collidine (156 μL, 1.2 mmol, 3.0 equiv.) and 4 mL of dry MeCN were added. After degassing with Argon balloon for 8 minutes, the liquid was taken up with a syringe and mounted on a syringe pump. The syringe was connected to a 4.0 mL reactor (perfluoroalkoxyalkane (PFA) capillary tubing, 0.8 mm inner diameter) at ambient temperature. The liquid feed was pumped into the flow

reactor The liquid feed was pumped into the flow reactor at a suitable rate (corresponding to 30 or 45 minutes residence time), which was irradiated with 10 W blue LEDs lamps. The reaction mixture was then concentrated and purified via column chromatography on silica gel to afford the product **3**.

The reaction for **3z** was run for 30 minutes reaction time, which flow rates of 133.3 μL/min used. The product **3z** was purified with silica gel chromatography (petroleum ether / ethyl acetate = 15:1) as colorless liquid (95.6 mg, 80% yield). Analogous conditions for **3z** (same concentration) were used in the scale up reaction, allowing 880 mg of **3z** to be isolated in 73% yield after 5 hours.

The reaction for **3aa** was run for 45 minutes reaction time, which flow rates of 88.9 μL/min used. The product **3aa** was purified with silica gel chromatography (petroleum ether / ethyl acetate = 6:1) as white solid (91.5 mg, 68% yield).

6. Preliminary mechanistic experiments

6.1 Radical inhibition experiments

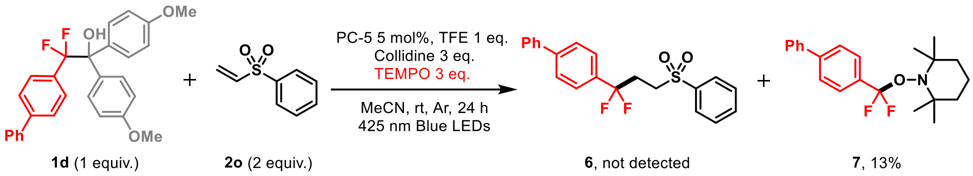

To a 4 mL vial equipped with a stir bar was added fluorinated alcohol **1d** (178.4 mg, 0.4 mmol, 2.0 equiv.), PC-5 (6.5 mg, 0.01 mmol, 5 mol%), TEMPO (93.6 mg, 0.6 mmol, 3.0 equiv.) and **2o** (33.6 mg, 0.2 mmol, 1.0 equiv.). The vial was sealed, evacuated and backfilled with Argon three times, then TFE (14 μL, 0.2 mmol, 1.0 equiv.) collidine (78 μL, 0.6 mmol, 3.0 equiv.) and 2 mL of dry MeCN were added. After degassing with Argon balloon for 8 minutes, the reaction mixture was irradiated with 10 W blue LEDs lamps for 24 hours at ambient temperature. Expected compound **6** wasn't determined by GC-MS. The reaction mixture was then concentrated and purified on a preparative TLC with petroleum ether/ethyl acetate (30:1) as the eluent to afford

the TEMPO-capture **7** as white solid (9.3 mg, 13% yield). ^{1}H NMR (400 MHz, $CDCl_3$) δ 7.71 (d, J = 8.4 Hz, 2H), 7.64 (d, J = 8.4 Hz, 2H), 7.60 (d, J = 7.2 Hz, 2H), 7.46 (t, J = 7.5 Hz, 2H), 7.38 (t, J = 7.3 Hz, 1H), 1.76-1.52 (m, 6H), 1.29 (t, J = 3.0 Hz, 6H), 1.22 (s, 6H). ^{19}F NMR (376 MHz, $CDCl_3$) δ -63.49 (s, 2F). ^{13}C NMR (101 MHz, $CDCl_3$) δ 143.3, 140.3, 133.9 (t, J =32.8 Hz), 128.9, 127.8, 127.3, 127.1, 125.9 (t, J = 3.5 Hz), 122.1 (t, J = 263.6 Hz), 61.1, 40.3, 34.2 (t, J = 6.1 Hz), 21.1, 17.1. HRMS (ESI): m/z [$(M+H)^+$] calcd for $C_{22}H_{28}F_2NO$, 360.2133. found, 360.2137.

6.2 Radical clock experiment

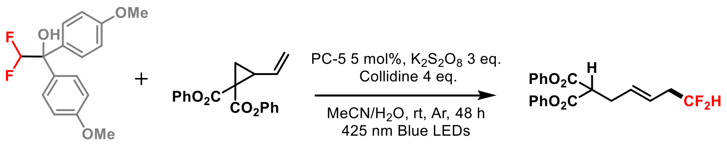

1a (2 equiv.) **8** (1 equiv.) **9**, 59%

To a 10 mL vial equipped with a stir bar was added fluorinated alcohol **1a** (58.8 mg, 0.2 mmol, 2.0 equiv.), PC-5 (3.3 mg, 5 μmol, 5 mol%), $K_2S_2O_8$ (81.0 mg, 0.3 mmol, 3.0 equiv.) and alkene **8** (0.1 mmol, 1.0 equiv.) The vial was sealed, evacuated and backfilled with Argon three times, then collidine (52 μL, 0.4 mmol, 4.0 equiv.) and 1.5 mL MeCN and 0.5 mL H_2O were added. After degassing with Argon balloon for 8 minutes, the reaction mixture was irradiated with 10 W blue LEDs lamps for 48 hours at ambient temperature. The reaction mixture was then concentrated and purified on a preparative TLC with petroleum ether/ethyl acetate as the eluent to afford the products **9** as light-yellow oil (21.3 mg, 59% yield). ^{1}H NMR (400 MHz, $CDCl_3$). δ 7.40 (t, J = 7.5 Hz, 4H), 7.26 (t, J = 7.5 Hz, 2H), 7.12 (d, J = 8.0 Hz, 4H), 5.93-5.62 (m, 3H), 3.92 (t, J = 7.3 Hz, 1H), 2.90 (t, J = 7.1 Hz, 2H), 2.67-2.51 (m, 2H). ^{19}F NMR (376 MHz, $CDCl_3$) δ -115.88 (dt, J = 56.5, 17.4 Hz, 2F). ^{13}C NMR (101 MHz, $CDCl_3$) δ 167.1, 150.5, 131.2, 129.6, 126.4, 124.1 (t, *J* = 6.6 Hz), 121.3, 116.1 (t, *J* = 241.4 Hz), 51.8, 37.6 (t, *J* = 21.7 Hz), 31.8. HRMS (ESI): m/z [$(M+Na)^+$] calcd for $C_{20}H_{18}F_2NaO_4$, 383.1071. found, 383.1067.

6.3 Radical trapping experiment

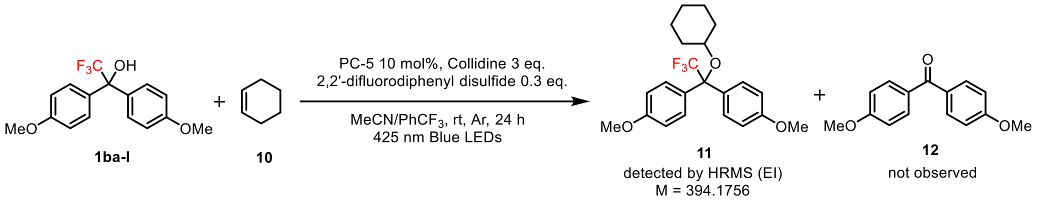

To a 4 mL vial equipped with a stir bar was added fluorinated alcohol **1ba-1** (62.4 mg, 0.2 mmol, 1.0 equiv.), and PC-5 (13.0 mg, 0.02 mmol, 10 mol%). The vial was sealed, evacuated and backfilled with Argon three times, then 2,2'-difluorodiphenyl disulfide (11.2 μL, 0.06 mmol, 0.3 equiv.), cyclohexene (61 μL, 0.6 mmol, 3.0 equiv.), collidine (78 μL, 0.6 mmol, 3.0 equiv.), 1 mL of dry MeCN and 1 mL of dry $PhCF_3$

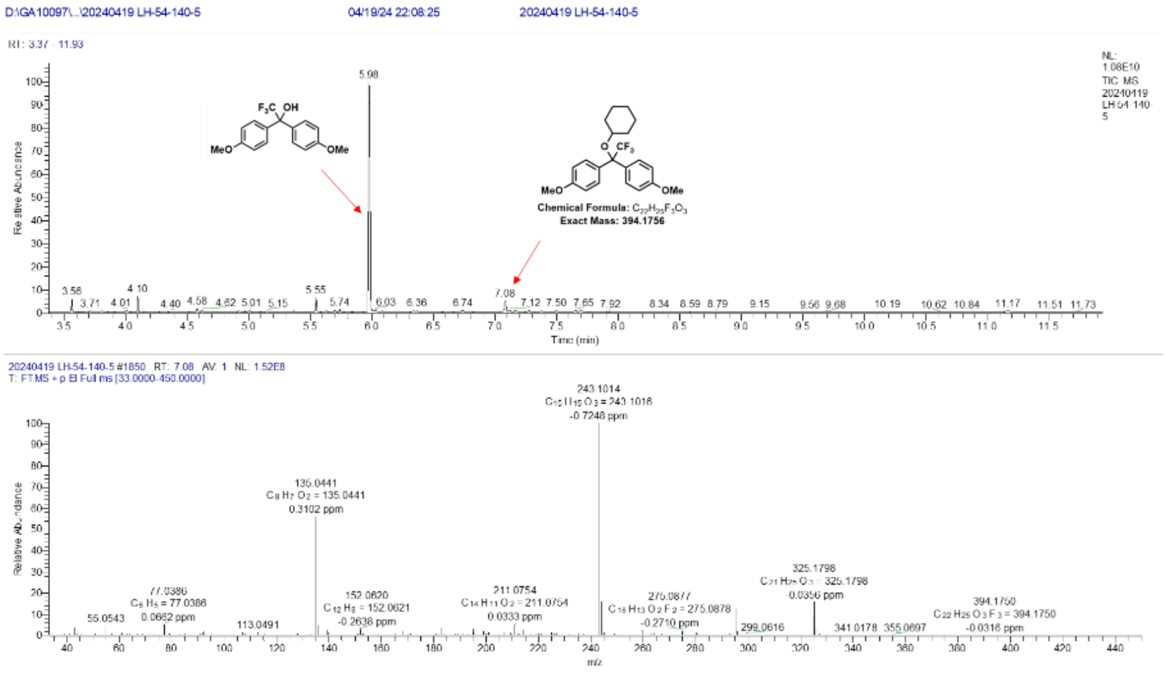

were added. After degassing with Argon balloon for 8 minutes, the reaction mixture was irradiated with 10 W blue LEDs lamps for 24 hours at ambient temperature. Compound **9** was detected by GC-HRMS, and byproduct 4,4'-dimethoxybenzophenone wasn't determined by GC-MS. HRMS (EI): m/z [M^+] calcd for $C_{22}H_{25}F_3O_3$, 394.1750. found, 394.1750.

Figure S16. HRMS (EI) spectra for compound **11.**

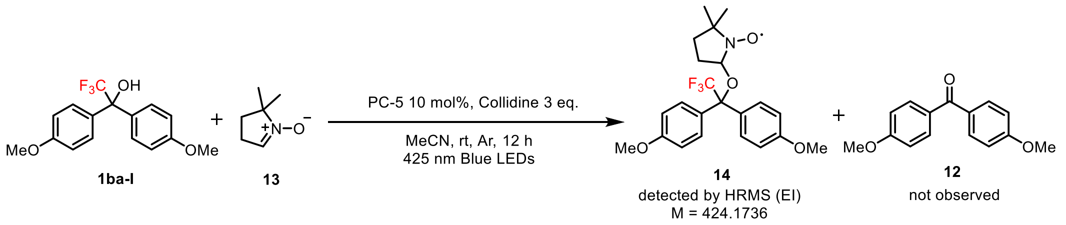

To a 4 mL vial equipped with a stir bar was added fluorinated alcohol **1ba-1** (62.4 mg, 0.2 mmol, 1.0 equiv.), and PC-5 (13.0 mg, 0.02 mmol, 10 mol%). The vial was sealed, evacuated and backfilled with Argon three times, then 2,2-dimethyl-1-oxido-3,4-dihydropyrrol-1-ium (34.0 mg, 0.6 mmol, 3.0 equiv.), collidine (78 µL, 0.6 mmol, 3.0 equiv.), 1 mL of dry MeCN was added. After degassing with Argon balloon for 8 minutes, the reaction mixture was irradiated with 10 W blue LEDs lamps for 12 hours at ambient temperature. Compound **14** was detected by HRMS, and byproduct 4,4'-dimethoxybenzophenone wasn’t determined by GC-MS. HRMS (EI): m/z [M^+] calcd for $C_{22}H_{25}O_4NF_3$, 424.1730 found, 424.1736.

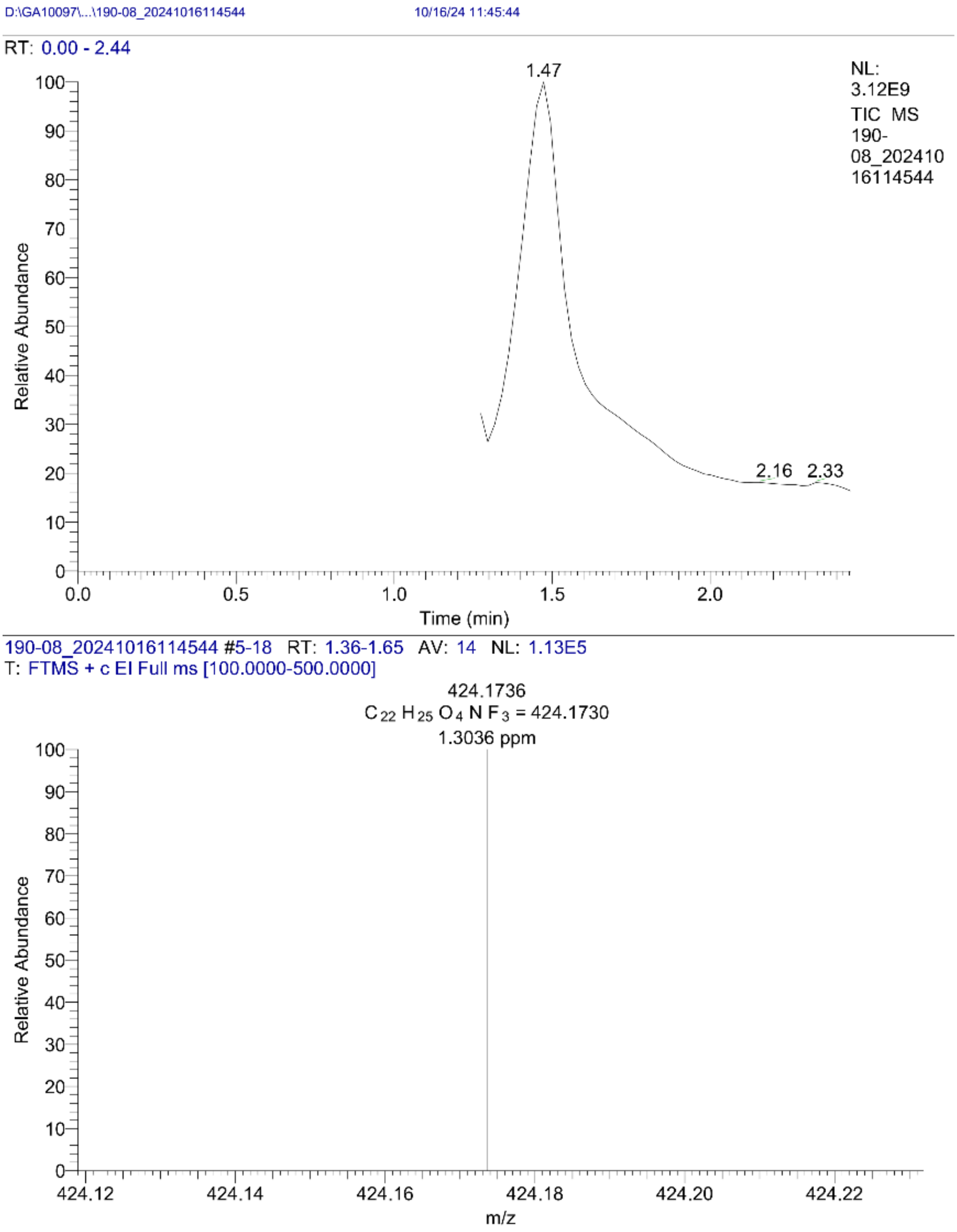

Figure S17. HRMS (EI) spectra for compound **14.**

6.4 Deuterium labeling experiment

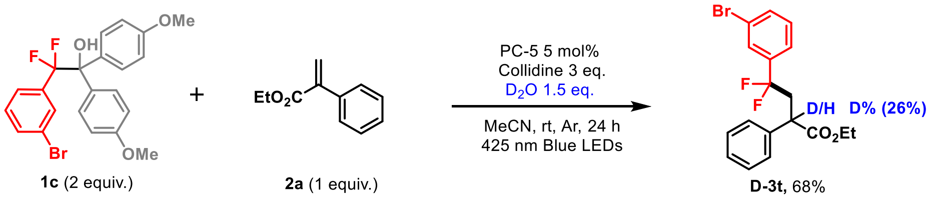

To a 4 mL vial equipped with a stir bar was added fluorinated alcohol **1c** (180 mg, 0.4 mmol, 2.0 equiv.) and PC-5 (6.5 mg, 0.01 mmol, 5 mol%). The vial was sealed, evacuated and backfilled with Argon three times, then alkene **2a** (35.2 mg, 0.2 mmol, 1.0 equiv.), D_2O (5.5 µL, 0.3 mmol, 1.5 equiv.) collidine (78 µL, 0.6 mmol, 3.0 equiv.) and 2 mL of MeCN were added. After degassing with Argon balloon for 8 minutes, the reaction mixture was irradiated with 10 W blue LEDs lamps for 24 hours at ambient temperature. The reaction mixture was then concentrated and purified on a preparative TLC with petroleum ether/ethyl acetate (20:1) as the eluent to afford the products **D-3t** as colorless liquid (52.2 mg, 68% yield). ^{1}H NMR (400 MHz, $CDCl_3$) δ 7.61 (s, 1H), 7.54 (d, J = 8.0 Hz, 1H), 7.39 (d, J = 7.8 Hz, 1H), 7.31-7.24 (m, 6H), 4.13-3.98 (m, 2H), 3.91 (dd, J = 9.6, 3.6 Hz, 0.74H), 3.24-3.09 (m, 1H), 2.50-2.36 (m, 1H), 1.18 (t, J = 7.1 Hz, 3H).

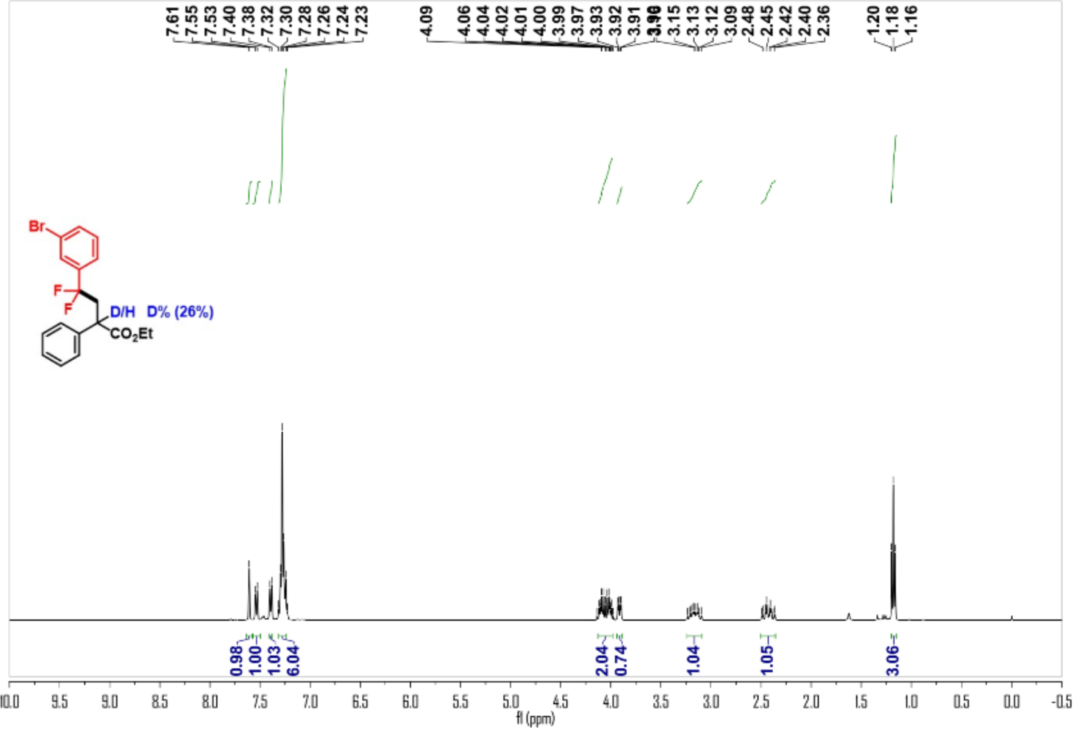

6.5 Stern-Volmer Studies

Stern-Volmer experiments tracking the quenching of the phosphorescence of PC-5 was conducted on a PerkinElmer Fluorescence Spectrophotometer. Fresh stock solutions of photocatalyst PC-5, 2,2-difluoro-1,1-bis(4-methoxyphenyl)ethan-1-ol (**1a**),

2a, and collidine were prepared in degassed MeCN and mixed together at varying concentrations in volumetric flasks. The solutions were loaded into quartz cuvettes, sealed under inert atmosphere, and shielded from light exposure before sample collection. The samples were irradiated at 427 nm, and three-scan average emission intensities at 533 nm were recorded. Each concentration combination was repeated three times, and the final average slope of I_0/I to concentration is reported here as the K_{SV}.

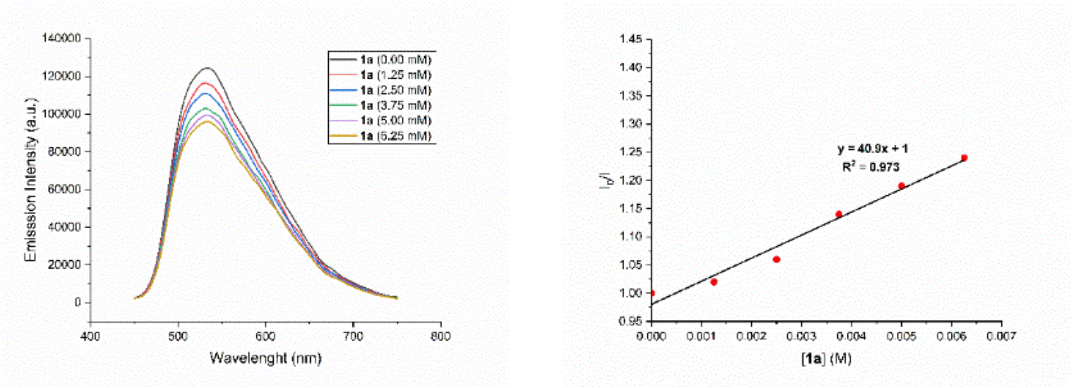

Figure S18. Stern-Volmer plot of **PC-5** (250 μM) with varied [**1a**] in MeCN at 23 °C.

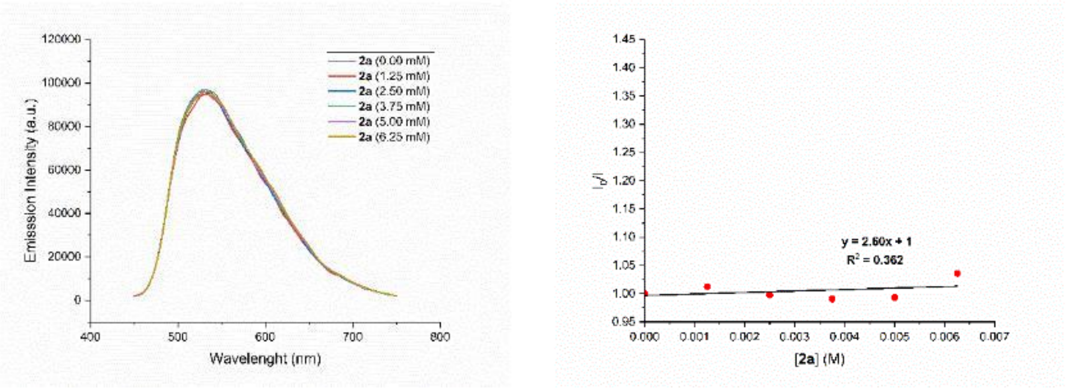

Figure S19. Stern-Volmer plot of **PC-5** (250 μM) with varied [**2a**] in MeCN at 23 °C.

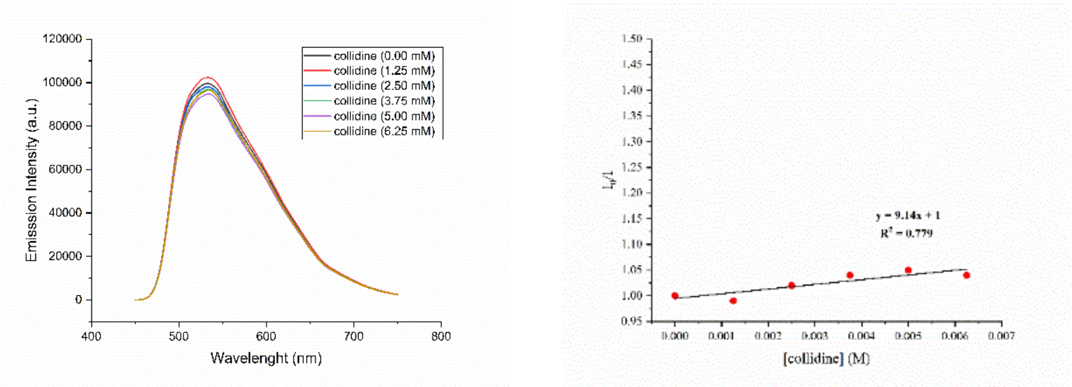

Figure S20. Stern-Volmer plot of **PC-5** (250 µM) with varied [collidine] in MeCN at 23 °C.

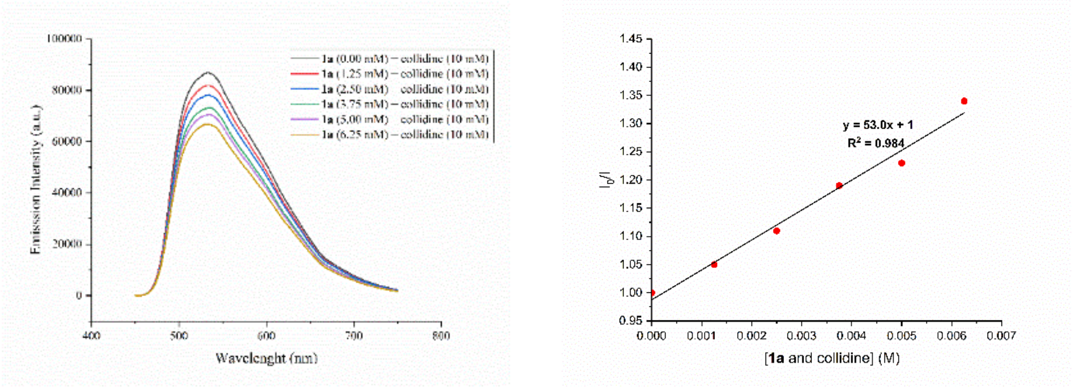

Figure S21. Stern-Volmer plot of **PC-5** (250 µM) with varied [**1a** + collidine] in MeCN at 23 °C.

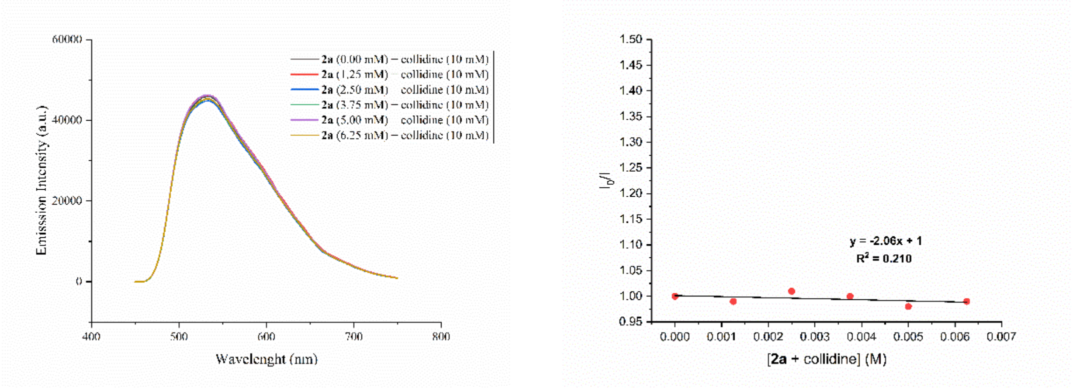

Figure S22. Stern-Volmer plot of **PC-5** (250 µM) with varied [**2a** + collidine] in MeCN at 23 °C.

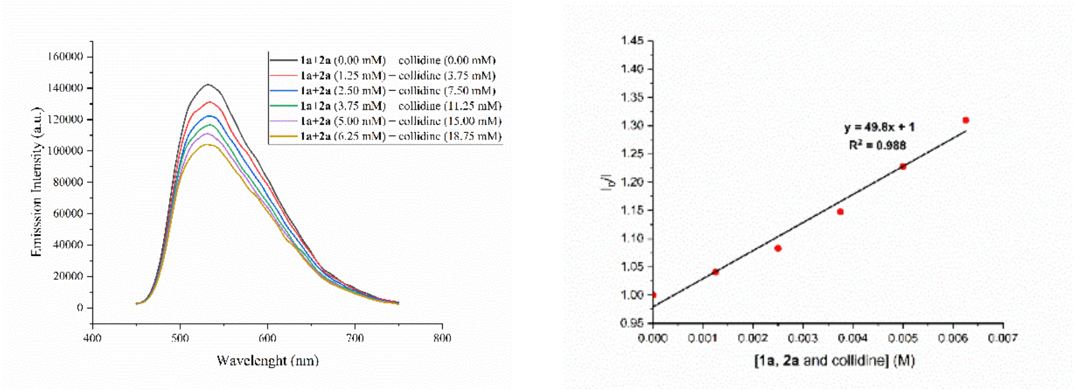

Figure S23. Stern-Volmer plot of **PC-5** (250 μM) with varied [**1a** + **2a** + collidine] in MeCN at 23 °C.

6.6. ^{1}H NMR experiment

To a 4 mL vial equipped with a stir bar was added fluorinated alcohol **1a** (50mg, 0.17 mmol) and 2.5 mL $CDCl_3$. The above solution (0.5 ml, 34 μmol) was added sequentially to five NMR tubes A-E. Then, 0.0 mg (0 μmol), 4.1 mg (34 μmol), 8.2 mg (68 μmol), and 24.6 mg (204 μmol) of collide was added sequentially to NMR tubes A-D. ^{1}H NMR spectra were recorded on a Bruker 400 MHz spectrometer.

6.7. Light/dark experiment

We applied the light/dark experiment to monitor the process of this reaction. To a 4 mL vial equipped with a stir bar was added fluorinated alcohol **1a** (117.6 mg, 0.4 mmol, 2.0 equiv.) and PC-5 (6.5 mg, 0.01 mmol, 5 mol%). The vial was sealed, evacuated and backfilled with Argon three times, then dodecane (0.2 mmol 1.0 equiv, internal standard.), electron-withdrawing alkene **2a** (35.2 mg, 0.2 mmol, 1.0 equiv.), TFE (14 μL, 0.2 mmol, 1.0 equiv.) collidine (78 μL, 0.6 mmol, 3.0 equiv.) and 2 mL of dry MeCN were added. After degassing with Argon balloon for 8 minutes, the reaction mixture was irradiated with 10 W blue LEDs lamps at ambient temperature. A little sample was obtained by syringe when we turn on or turn off the light. Next, we monitor corresponding yields by gas chromatography-mass spectrometer (GC-MS). The result was shown in Figure S24.

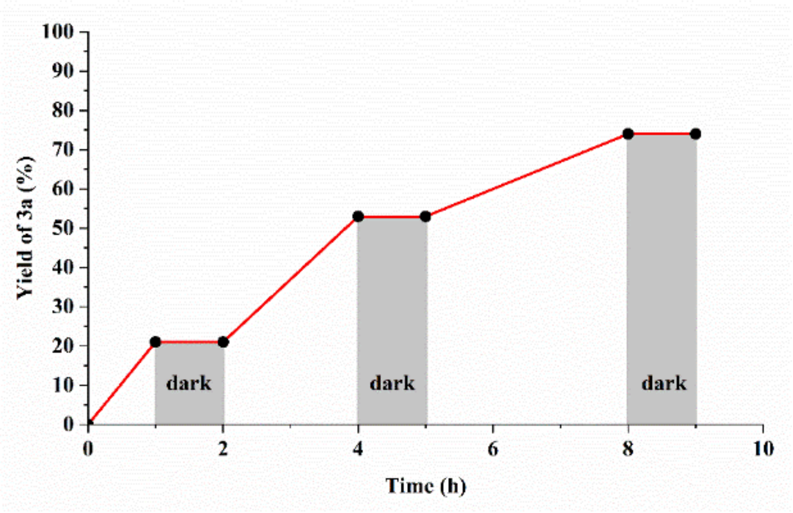

Figure S24. Successive intervals of irradiation and dark periods for reactions.

7. General Computational Procedure

Conformational search was performed using CREST of the XTB program.[54] DFT calculation of each low energy conformer was performed using Gaussian 16.[55] The structure of each species was submitted for geometry optimization at M06-2X/def2tzvp level with CPCM solvation model for acetonitrile and with the integration grid set to ultrafine level, followed by frequency calculation at the same theoretical level. All reported Gibbs free energies are for 298K and are after quasi-harmonic correction using the GoodVibes program.[56]

Optimized Coordinates and Calculated Energies

**12-A1**			
E[M06-2X/6-31G(d,p)/CPCM(ether)]= -805.667036364			
Zero-point correction= 0.258998			
Thermal correction to Energy= 0.274653			
Thermal correction to Enthalpy= 0.275598			
Thermal correction to Gibbs Free Energy= 0.215055			
O	0.119469	2.775819	-0.575497
C	0.124588	1.568771	-0.405865
C	1.409069	0.817178	-0.424275
C	2.574107	1.480260	-0.050342
H	2.506353	2.508340	0.281261
C	3.807111	0.849529	-0.085569
H	4.690106	1.386291	0.227775
C	3.885285	-0.470999	-0.529989
O	5.032814	-1.174450	-0.610763
C	6.238447	-0.533124	-0.226424
H	7.027490	-1.265121	-0.369301
H	6.203018	-0.232711	0.822438
H	6.431552	0.340621	-0.851659
C	2.725750	-1.141293	-0.928730
H	2.810850	-2.159215	-1.284965
C	1.503154	-0.506072	-0.866612
H	0.615920	-1.034602	-1.190246
C	-1.153094	0.839738	-0.180458
C	-2.325293	1.369579	-0.711763
H	-2.267973	2.272680	-1.305645
C	-3.552275	0.760985	-0.503415
H	-4.441044	1.187379	-0.944233
C	-3.616983	-0.393979	0.277263

O	-4.757517	-1.060223	0.547970
C	-5.970216	-0.554272	0.013215
H	-6.751967	-1.228178	0.350153
H	-5.940704	-0.543850	-1.077928
H	-6.169373	0.452815	0.384310
C	-2.450355	-0.923975	0.834737
H	-2.525082	-1.810716	1.449886
C	-1.233944	-0.318237	0.599407
H	-0.341261	-0.733968	1.048264

12-A3

E[M06-2X/6-31G(d,p)/CPCM(ether)]= -765.185200492

Zero-point correction= 0.208966

Thermal correction to Energy= 0.221719

Thermal correction to Enthalpy= 0.222663

Thermal correction to Gibbs Free Energy= 0.169566

O	-1.260216	1.919689	1.309128
C	-0.998459	0.859301	0.762826
C	-2.040722	-0.097444	0.353485
C	-3.395409	0.170890	0.576816
H	-3.654110	1.104476	1.059247
C	-4.363677	-0.729852	0.192055
H	-5.409232	-0.516881	0.367621
C	-3.986950	-1.925443	-0.428662
H	-4.743595	-2.636983	-0.732753
C	-2.657846	-2.213117	-0.660230
H	-2.348482	-3.132355	-1.138871
C	-1.687622	-1.294352	-0.266649
O	-0.397493	-1.635139	-0.521861
C	0.606270	-0.794129	-0.167249
C	0.367753	0.432024	0.460833
C	1.469694	1.227014	0.786399
H	1.283079	2.175773	1.272599
C	2.756809	0.831286	0.505100
H	3.586261	1.469076	0.769588
C	2.967382	-0.406497	-0.127173
O	4.182860	-0.885190	-0.444910
C	5.322708	-0.098934	-0.128530
H	6.181307	-0.667783	-0.471388
H	5.396965	0.063546	0.947931
H	5.288906	0.861295	-0.645601
C	1.890876	-1.217337	-0.462341
H	2.054944	-2.168935	-0.948409

A1			
E[M06-2X/6-31G(d,p)/CPCM(ether)]= -1044.00611526			
Zero-point correction= 0.281831			
Thermal correction to Energy= 0.300762			
Thermal correction to Enthalpy= 0.301706			
Thermal correction to Gibbs Free Energy= 0.232534			
O	0.016752	2.451303	-1.673126
C	0.029600	1.919263	-0.407480
C	1.324231	1.095083	-0.471351
C	2.485902	1.421665	0.215679
C	3.615709	0.617108	0.124033
C	3.596530	-0.511651	-0.690723
O	4.640054	-1.348675	-0.851268
C	5.838710	-1.063718	-0.147534
C	2.436738	-0.832198	-1.405255
C	1.319617	-0.037706	-1.294516
C	-1.184659	1.054360	-0.104847
C	-2.322014	1.143293	-0.888275
C	-3.451282	0.380679	-0.605527
C	-3.440502	-0.478345	0.488742
O	-4.482179	-1.262859	0.852636
C	-5.659057	-1.205483	0.065896
C	-2.297645	-0.568246	1.287439
C	-1.183410	0.189543	0.989443
C	0.096280	3.053028	0.628522
F	-1.077552	3.737785	0.598699
F	1.058629	3.950681	0.302204
H	2.537143	2.297266	0.846326
H	4.496746	0.884532	0.687950
H	6.538473	-1.849581	-0.414716
H	5.668977	-1.074812	0.930745
H	6.243756	-0.094893	-0.445539
H	2.439028	-1.711837	-2.034933
H	0.419773	-0.294706	-1.839215
H	-2.335103	1.813473	-1.738687
H	-4.321246	0.468323	-1.239460
H	-6.360298	-1.899828	0.519218
H	-5.457075	-1.509620	-0.963309
H	-6.085091	-0.199972	0.071599
H	-2.304245	-1.245611	2.131208
H	-0.296348	0.095165	1.606034
H	0.268060	2.700191	1.646389

A2

E[M06-2X/6-31G(d,p)/CPCM(ether)]= -1143.27528238
Zero-point correction= 0.273515
Thermal correction to Energy= 0.293068
Thermal correction to Enthalpy= 0.294012
Thermal correction to Gibbs Free Energy= 0.223701

O	0.037740	2.395287	-1.663012
C	0.030368	1.882276	-0.392245
C	1.333251	1.071422	-0.436005
C	2.510499	1.437345	0.205854
C	3.645445	0.643010	0.110498
C	3.618375	-0.514761	-0.663863
O	4.665966	-1.343732	-0.822548
C	5.881057	-1.021703	-0.162940
C	2.444081	-0.872938	-1.336171
C	1.321756	-0.087592	-1.223530
C	-1.190157	1.026451	-0.090946
C	-2.339837	1.174653	-0.847931
C	-3.476818	0.418651	-0.582304
C	-3.459714	-0.495138	0.466922
O	-4.507191	-1.278679	0.811306
C	-5.698569	-1.162454	0.052706
C	-2.303083	-0.646158	1.236616
C	-1.181236	0.106374	0.956715
C	0.077373	3.062308	0.600968
F	0.271504	2.657215	1.859956
F	-1.078604	3.733686	0.569482
F	1.037233	3.941947	0.305455
H	2.567386	2.330038	0.810412
H	4.537678	0.939367	0.641244
H	6.581357	-1.809504	-0.422493
H	5.740823	-0.997023	0.919190
H	6.266235	-0.060010	-0.506440
H	2.440482	-1.772782	-1.936365
H	0.412981	-0.370788	-1.739160
H	-2.356607	1.886243	-1.664264
H	-4.356555	0.552962	-1.194031
H	-6.402560	-1.866847	0.485635
H	-5.521975	-1.418308	-0.994057
H	-6.106270	-0.151550	0.117306
H	-2.306184	-1.365989	2.044386
H	-0.285228	-0.029280	1.550653

A3

E[M06-2X/6-31G(d,p)/CPCM(ether)]= -1003.51388956

Zero-point correction= 0.231867
Thermal correction to Energy= 0.247851
Thermal correction to Enthalpy= 0.248795
Thermal correction to Gibbs Free Energy= 0.187564

O	-1.000683	-2.077322	-0.813097
C	-0.963870	-1.128865	0.189111
C	-2.007890	-0.052029	-0.019631
C	-3.329058	-0.399308	-0.302878
C	-4.297285	0.572873	-0.476765
C	-3.951000	1.916942	-0.363571
C	-2.643686	2.280914	-0.100550
C	-1.678937	1.292969	0.060452
O	-0.403599	1.729027	0.288355
C	0.623962	0.844199	0.166389
C	0.434009	-0.536297	0.100456
C	1.556725	-1.348893	-0.037221
C	2.834403	-0.821738	-0.089373
C	3.001824	0.561941	0.004503
O	4.197379	1.181984	-0.024096
C	5.361172	0.381525	-0.164290
C	1.892389	1.391838	0.132822
C	-1.165960	-1.808464	1.555511
F	-2.366951	-2.445243	1.572878
F	-0.229167	-2.766711	1.750317
H	-3.589441	-1.447027	-0.382179
H	-5.317246	0.288449	-0.695719
H	-4.702517	2.684700	-0.491629
H	-2.345603	3.318211	-0.028125
H	1.424198	-2.419921	-0.097629
H	3.679374	-1.484373	-0.195081
H	6.200073	1.070602	-0.167043
H	5.340292	-0.174558	-1.103168
H	5.459410	-0.312428	0.672342
H	2.018998	2.463551	0.199754
H	-1.129316	-1.100549	2.384155

A4

E[M06-2X/6-31G(d,p)/CPCM(ether)]= -1102.78344116
Zero-point correction= 0.223359
Thermal correction to Energy= 0.240014
Thermal correction to Enthalpy= 0.240958
Thermal correction to Gibbs Free Energy= 0.178276

O	-1.100386	1.631574	1.555702
C	-1.051635	1.072894	0.299333

C	-2.065071	-0.039705	0.140888
C	-3.380052	0.140745	0.569152
C	-4.319417	-0.860772	0.405617
C	-3.948773	-2.059714	-0.197996
C	-2.645605	-2.260793	-0.613451
C	-1.708113	-1.251067	-0.431945
O	-0.433540	-1.528151	-0.837881
C	0.578310	-0.719802	-0.418819
C	0.359965	0.534104	0.151345
C	1.464081	1.276842	0.561993
C	2.754075	0.806325	0.400002
C	2.951331	-0.441173	-0.197813
O	4.160614	-0.990611	-0.415193
C	5.309148	-0.263203	-0.005587
C	1.859303	-1.201403	-0.606625
C	-1.306733	2.178879	-0.748088
F	-2.515075	2.727714	-0.587215
F	-1.245844	1.687111	-1.987858
F	-0.414559	3.165496	-0.656922
H	-3.659620	1.079511	1.030422
H	-5.335749	-0.709835	0.741956
H	-4.678589	-2.846369	-0.335824
H	-2.330176	-3.190421	-1.067476
H	1.309010	2.247865	1.011912
H	3.585628	1.409857	0.729092
H	6.163091	-0.880043	-0.267704
H	5.297044	-0.092172	1.072192
H	5.371800	0.691917	-0.529805
H	2.010539	-2.169602	-1.063404

CHF2-radical

E[M06-2X/6-31G(d,p)/CPCM(ether)]= -238.337466997

Zero-point correction= 0.019369

Thermal correction to Energy= 0.022463

Thermal correction to Enthalpy= 0.023408

Thermal correction to Gibbs Free Energy= -0.005642

C	0.143868	3.205077	0.526280
F	1.170315	3.981106	0.212138
H	-0.829524	3.657963	0.364493
F	0.312764	2.680330	1.730650

CF3-radical

E[M06-2X/6-31G(d,p)/CPCM(ether)]= -337.600630839

Zero-point correction= 0.012336

Thermal correction to Energy= 0.015750			
Thermal correction to Enthalpy= 0.016695			
Thermal correction to Gibbs Free Energy= -0.014337			
C	0.147060	3.186790	0.511764
F	1.141432	3.987283	0.207992
F	0.286793	2.694272	1.719814
F	-1.007959	3.794439	0.378319

Table S7. Calculated carbon-fluorinated carbon bond dissociation enthalpies

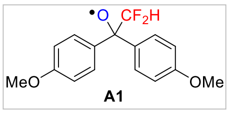

A1

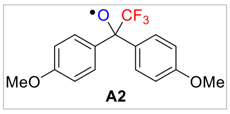

A2

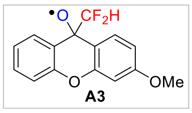

A3

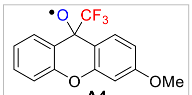

A4

Species	BDE	BDFE	EDFEiespi$_2$)
A1-OH	71.7	57.2	2.199
A2-OH	76.1	61.0	2.198
A3-OH	67.9	52.9	2.289
A4-OH	73.2	57.5	2.304

Note: potentials of hydrogenation, denoted E°(V vs H_2), of substrates at M062X/def2tzvp/CPCM(acetonitrile) level. BDE and BDFE are in kcal/mol. E°.V vs H_2) is in eV.

8. References

[1] Y.Wang, R.Mogi, J. Hu, T. Mathew, G. A. Olah, G. K. S. Prakash. *Org. Lett.* **2010**, *12*, 2932.
[2] J.-Y. Chai, H. Cha, H. B. Kim, D. Y. Chi. *Tetrahedron* **2020**, *76*, 131370.
[3] R. Feng, W. Yu, K. Wang, Z. Liu, Y. Zhang. *Adv. Synth. Catal*. **2014**, *356*, 1501.
[4] D. Liu, M. Ke, T. Ru, Y. N. F. Chen. *Chem. Commun.* **2022**, *58*, 1041.
[5] S. Park, D. Yang, K. T. Kim, H. B. Jeon. *Tetrahedron Lett.* **2011**, *52*, 6578.
[6] Q. Tang. et al. *ACS Med. Chem. Lett*. **2021**, *12*, 955.
[7] Y. Li, K. Li, Y. Wu, Q. Ma, X. Lei. *Tetrahedron* **2016**, *72*, 4845.
[8] Q. Miao. et al. Chem. Commun., 2019, 55, 7331.
[9] M. Oga, Y. Takamatsu, A. Ogura, K. Takao. *J. Org. Chem*. **2022**, *87*, 8788.
[10] C.Guo. et al. *J. Org. Chem.***2022**, *87*, 9232.
[11] M. Zhu, Q. You, R. Li. *J. Fluor. Chem*. **2019**, *228*, 10939.
[12] K. Lu. et al. *Tetrahedron Lett.* **2021**, *67*, 152864.
[13] Q.-W. Gui. et al. *Chin. Chem. Lett.* **2021**, *32*, 1907.
[14] Z. Cui, D.-M. Du. *Adv. Synth. Catal.* **2018**, *360*, 93.
[15] M.-Z. Lu, T.-P. Loh. *Org.Lett*. **2014**, *16*, 4698.
[16] G.-A. P, Y. Li, J.-H. Li. *Org. Chem. Front.* **2020**, *7*, 2486.
[17] X. Chen. et al. *Org.Lett.* **2021**, *23*, 7787.
[18] G. Darzens, L. Andre. *Compt. Rend*. **1934**, *199*, 1426.
[19] G. Zou, X. Wang. *Org. Biomol. Chem.* **2017**, *15*, 8748.
[20] W. E. Brenzovich. et al. *Angew. Chem. Int. Ed*. **2010**, *49*, 5519.

[21] M. Karikomi, M. Dhooghe, G. Verniest, N. D. Kimpe. *Org. Biomol. Chem*. **2008**, *6*, 1902.
[22] Y. Zhou, Z. Xiong, J. Qiu, L. Kong, G. Zhu. *Org. Chem. Front*. **2019**, *6*, 1022.
[23] Z. Zhang, X. Tang, W. R. Dolbier. *Org. Lett.* **2015**, *17*, 4401.
[24] M. Zhu. et al. *Org. Biomol. Chem.* **2017**, *15*, 9057.
[25] E. Schmitt. et al. *Org. Lett*. **2015**, *17*, 4510.
[26] Z. Deng. et al. *Org.Lett.* **2016**, *18*, 3206.
[27] T, Billard, et al. *Eur. J. Org. Chem.***2001**,1467.
[28] M. V. Riofski, A. D. Hart, and D. A. Colby. *Org. Lett*. **2013**, *15*, 208.
[29] Y. Wu, D. Kim, T. S. Teets. *Synlett* **2022**, *33*, 1154.
[30] L. Pitzer, F. Sandfort, F. Strieth-Kalthoff, F. Glorius. *Angew.Chem.Int.Ed*. **2018**, *57*, 16219.
[31] V. R. Yatham, Y. Shen, R. Martin. *Angew. Chem. Int. Ed*. **2017**, *56*, 10915.
[32] X.-J. Tang, Z. Zhang, W. R. Dolbier. *Chem. Eur. J.* **2015**, *21*, 18961.
[33] V. V. Levin. et al. *Org. Lett.* **2014**, *16*, 6256.
[34] X. Zeng. et al. *J. Am. Chem. Soc*. **2019**, *141*, 11398.
[35] Z.-P. Bao, Y. Zhang. X.-F. Wu. *Chem. Sci.* **2022**, *13*, 9387.
[36] K. Sato, M. Omote, A. Ando, I. Kumadaki. *J. Fluor. Chem*. **2004**, *125*, 509.
[37] G. Zhao, H. L. Sun, Z. Qian, W. X. Yin. *J. Fluor. Chem*. **2001**, *111*, 217.
[38] D. V. Sevenard. et al. *Tetrahedron Lett.* **2003**, *44*, 7623.
[39] Y.-F. Yang, J.-H. Lin, J.-C. Xiao. *Org. Lett*. **2021**, *23*, 9277.
[40] K. Sakavuyi, K. S. Petersen. *Tetrahedron Lett.* **2013**, 5*4*, 6129.
[41] J. Wei. et al. *Org. Chem. Front*. **2018**, *5*, 2568.
[42] D. Chowdhury, S. Dana, A. Mandal. M. Baidya. *Chem. Commun*. **2019**, *55*, 11908.
[43] X. Li. et al. *Chem. Asian. J*. **2020**, *15*, 1175.
[44] D. M. Rudzinski, C. B. Kellyza and N. E. Leadbeater. *Chem. Commun.,* **2012**, *48*, 9610.
[45] J. Zhang. et al. *Tetrahedron Lett.* **2017**, 5*8*, 2964.
[46] A. S. Golubev. et al. *Org. Biomol. Chem*., **2022**, 20, 6809.
[47] X. Xiao, Z. Long. and Q. Chen. *J. Fluor. Chem*. **2001**, *111*, 107.
[48] T. Fukuhara and S. Hara. *J. Org. Chem*. **2010**, *75*, 7393.
[49] A. Budinska, J. Vaclavik, V. Matousek, and P. Beier. *Org. Lett*. **2016**, *18*, 5844.
[50] K. I. Petko, T. M. Sokolenko, A. V. Bezdudny, and L. M. Yagupolskii. *J. Fluor. Chem*. **2005**, *126*, 1342.
[51] V. G. Koshechko, L. A. Kiprianova, L. I. Fileleeva, and L. I. Kalinina *J. Fluor. Chem*. **2007**, *128*, 1376.
[52] R. M. Moriarty, O.Prakash. *Organic Reactions*. **1999,** *54*. 277.
[53] L. Zhu. et al. *Chem. Commun.,* **2016**, *52*, 6371.
[54] S. Grimme. *J. Chem. Theory Comput.* **2019**, 15, 2847.
[55] M. J. Frisch. et al. Gaussian 16 Rev. C.01, Wallingford, CT, **2016**.
[56] G. Luchini. et al. *F1000Research.* **2020**, *9*, 291.
[57] A. L. Trifonov. et al. *Org.Lett.* **2016**, *18*, 3458.

9. Copies of NMR spectra data

^{1}H, and ^{19}F NMR spectra of compound 1a-II

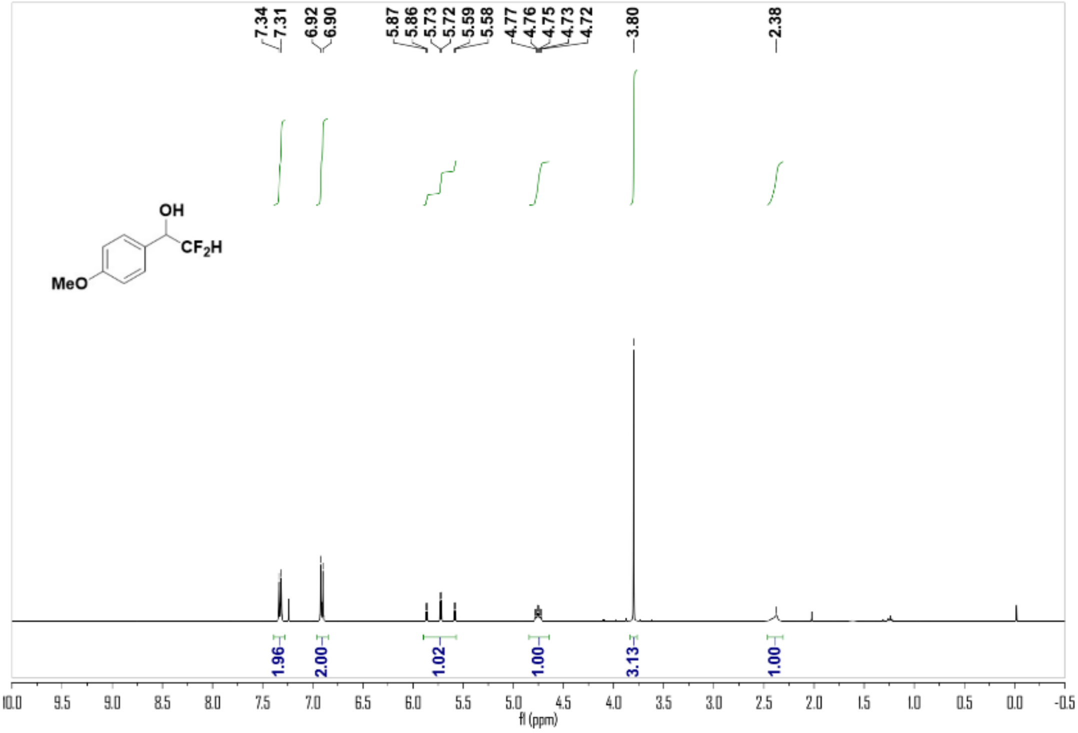

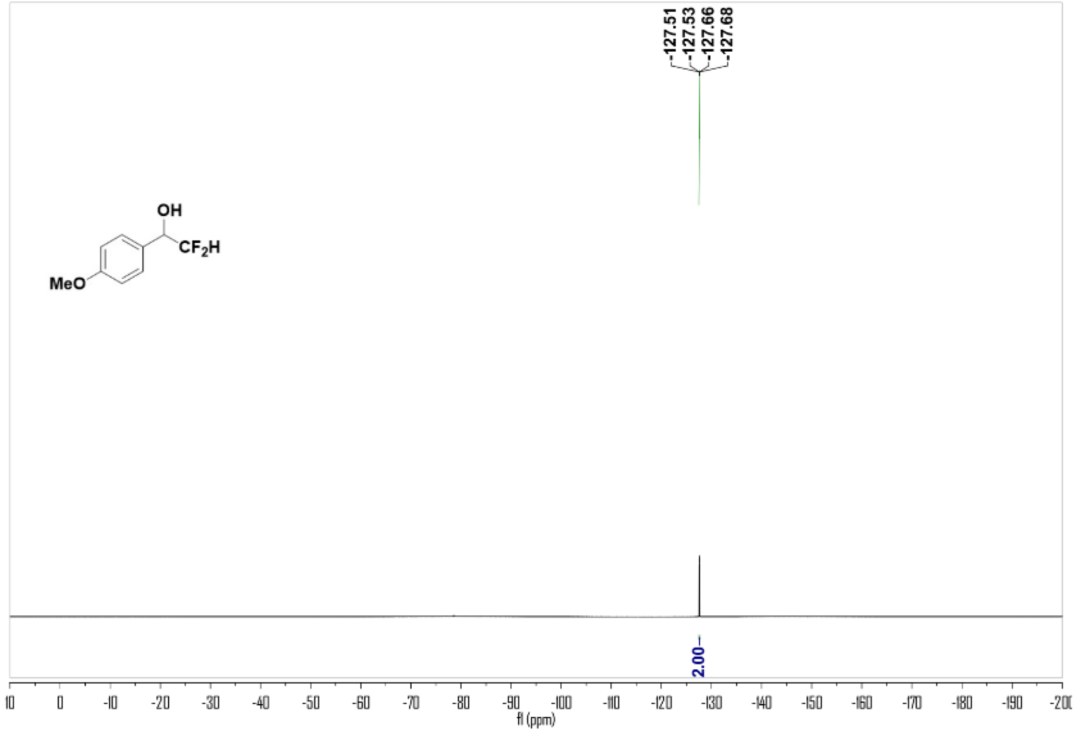

^{1}H, ^{19}F and ^{13}C NMR spectra of compound 1a-III

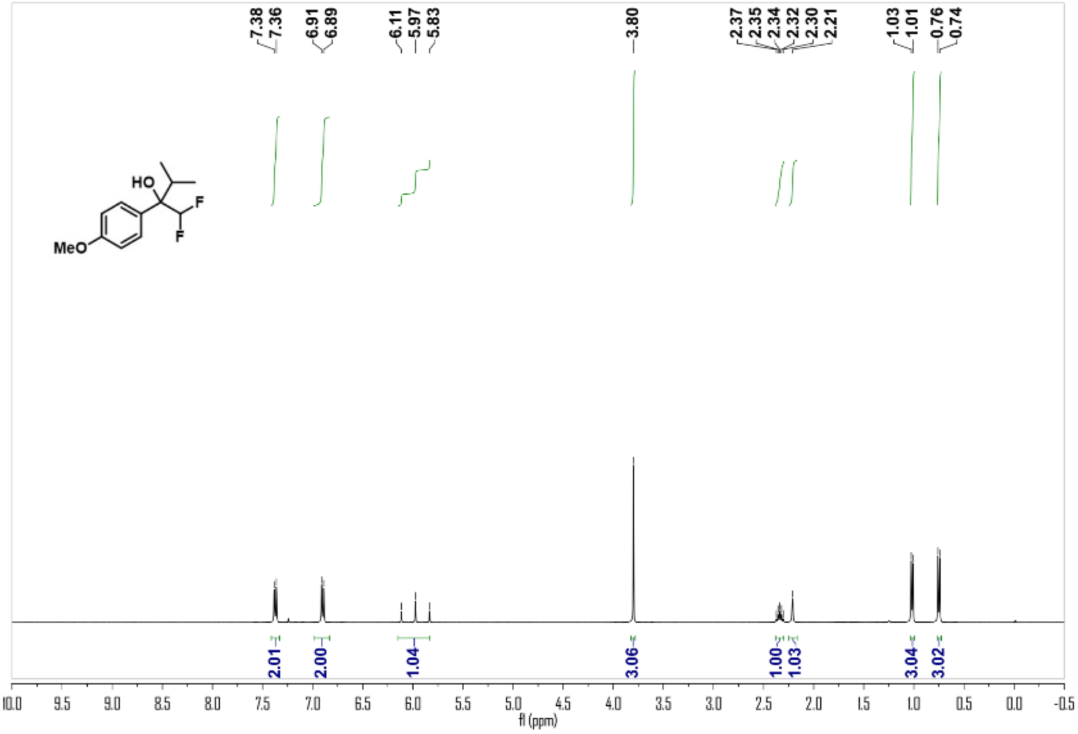

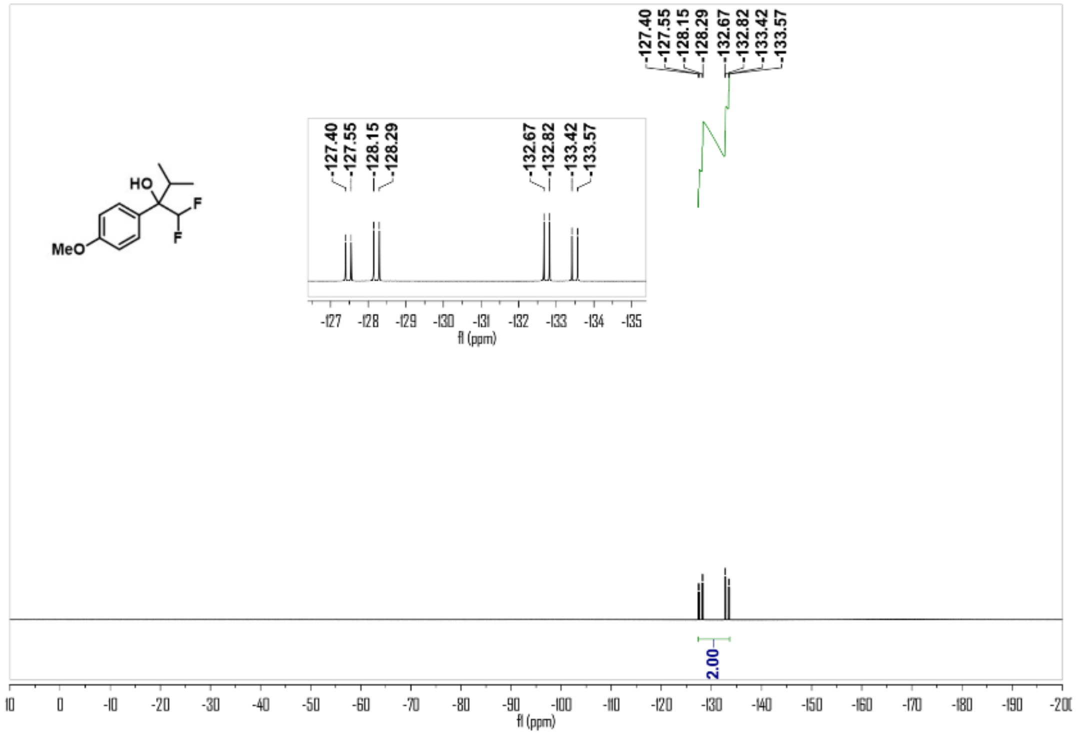

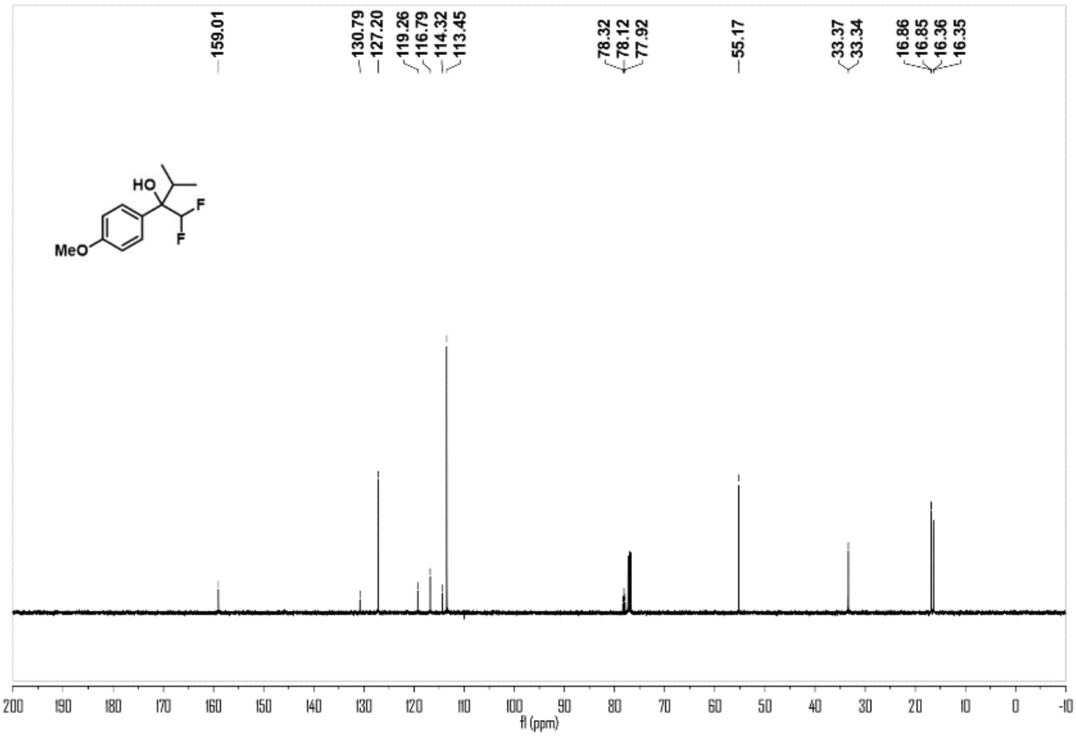

^{1}H, and ^{19}F NMR spectra of compound 1a-IV

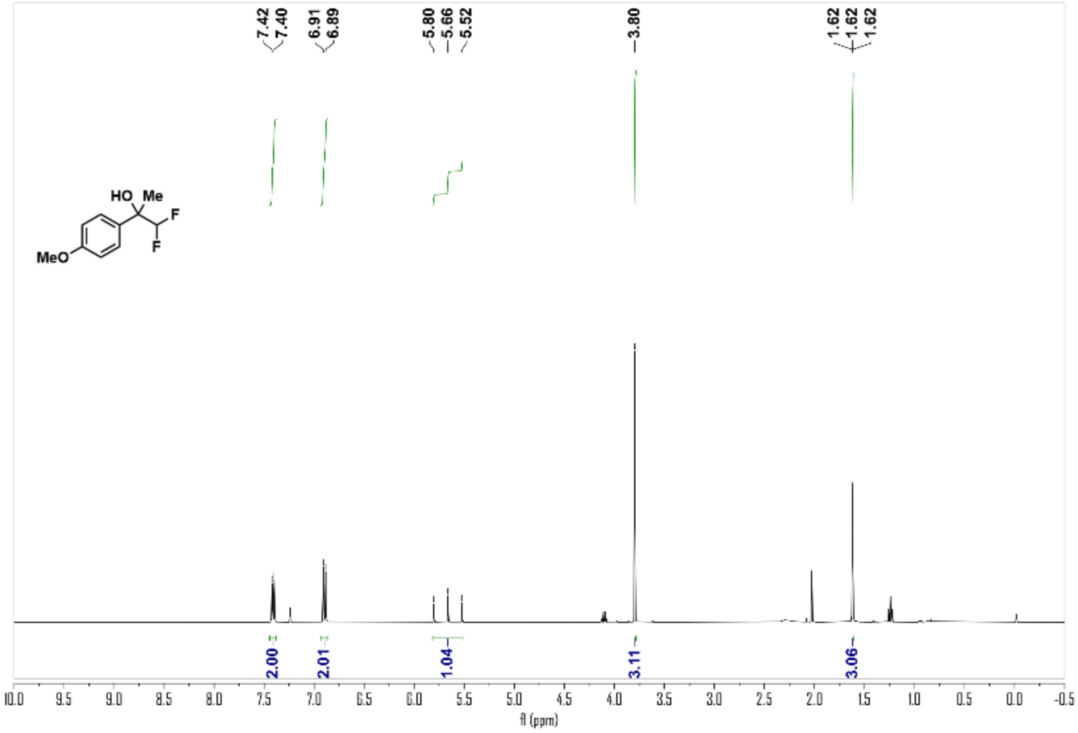

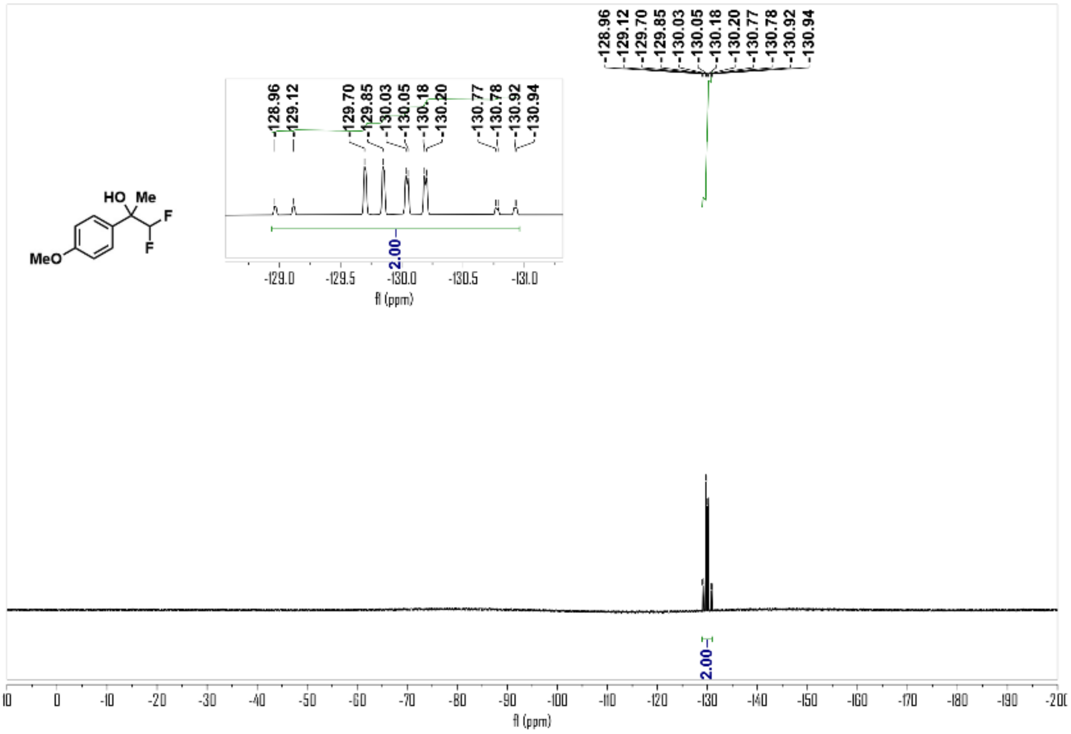

^{1}H, ^{19}F and ^{13}C NMR spectra of compound 1a-V

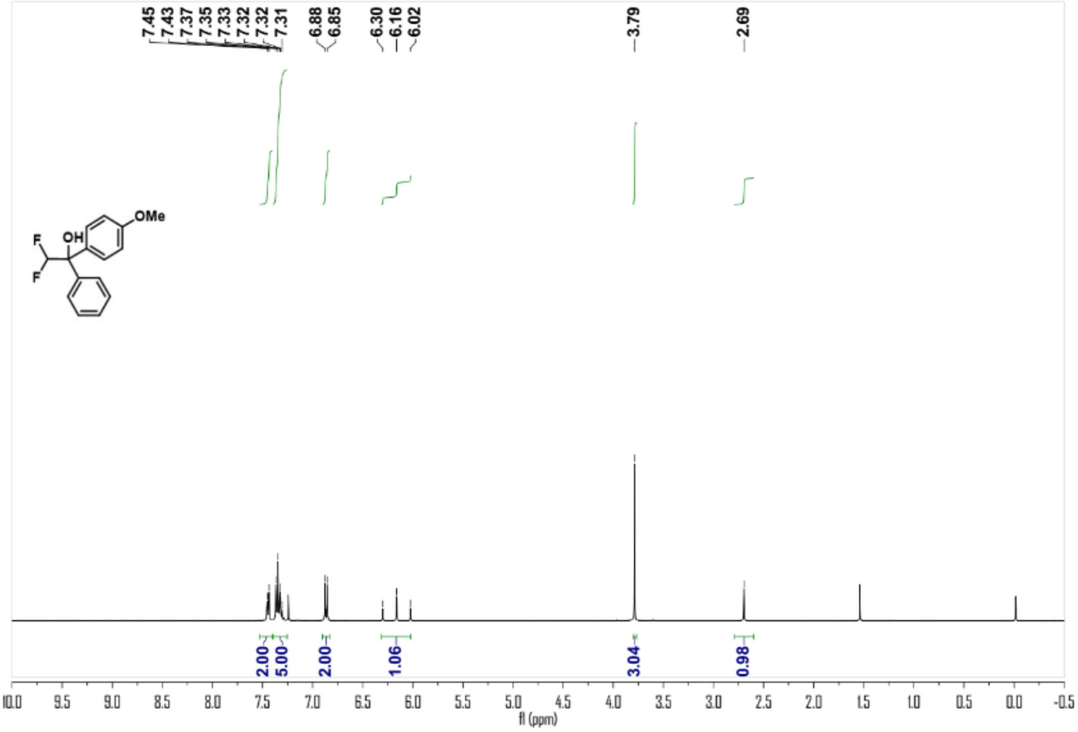

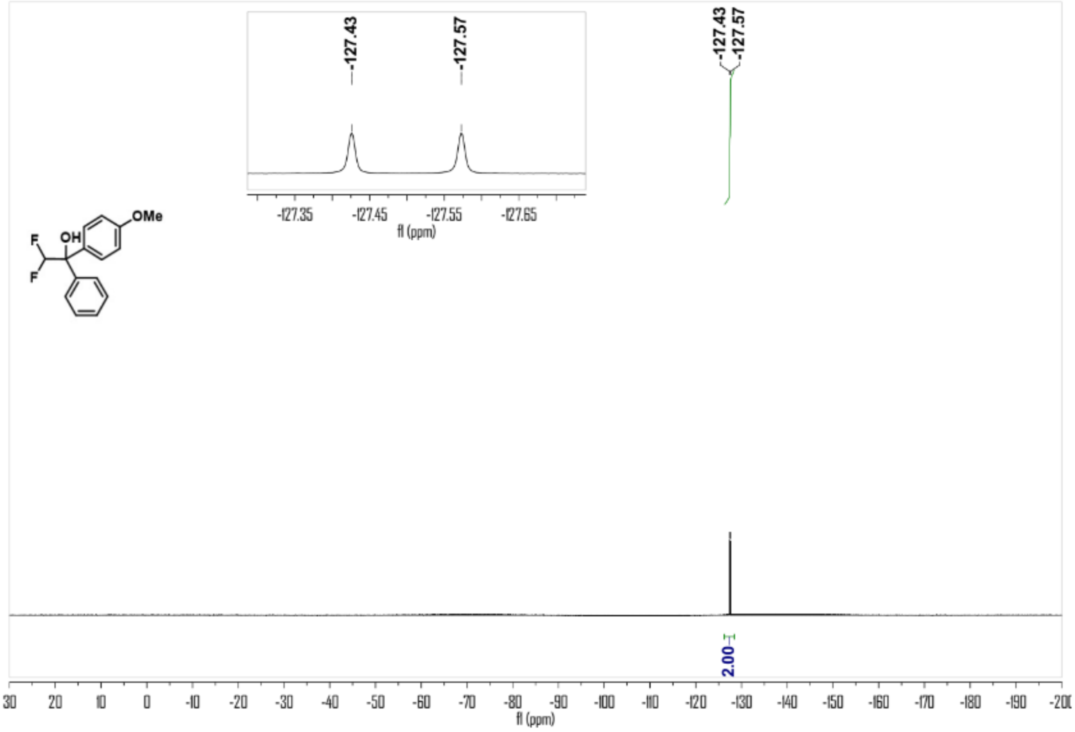
-127.43
-127.57
-127.35
-127.45
-127.55
-127.65
fl (ppm)
OMe
OH
F
F
2.00
30
20
10
0
-10
-20
-30
-40
-50
-60
-70
-80
-90
-100
-110
-120
-130
-140
-150
-160
-170
-180
-190
-200
fl (ppm)

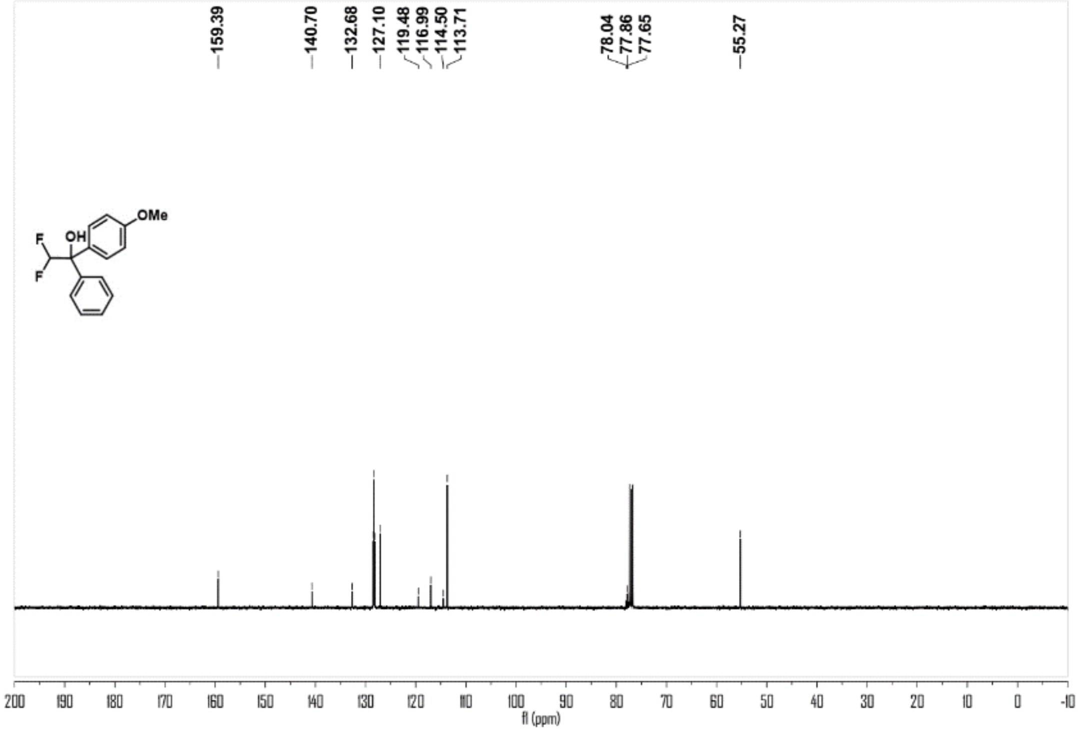
159.39
140.70
132.68
127.10
119.48
116.99
114.50
113.71
78.04
77.86
77.65
55.27
OMe
OH
F
F
200
190
180
170
160
150
140
130
120
110
100
90
80
70
60
50
40
30
20
10
0
-10
fl (ppm)

^{1}H, ^{19}F and ^{13}C NMR spectra of compound 1a-VI

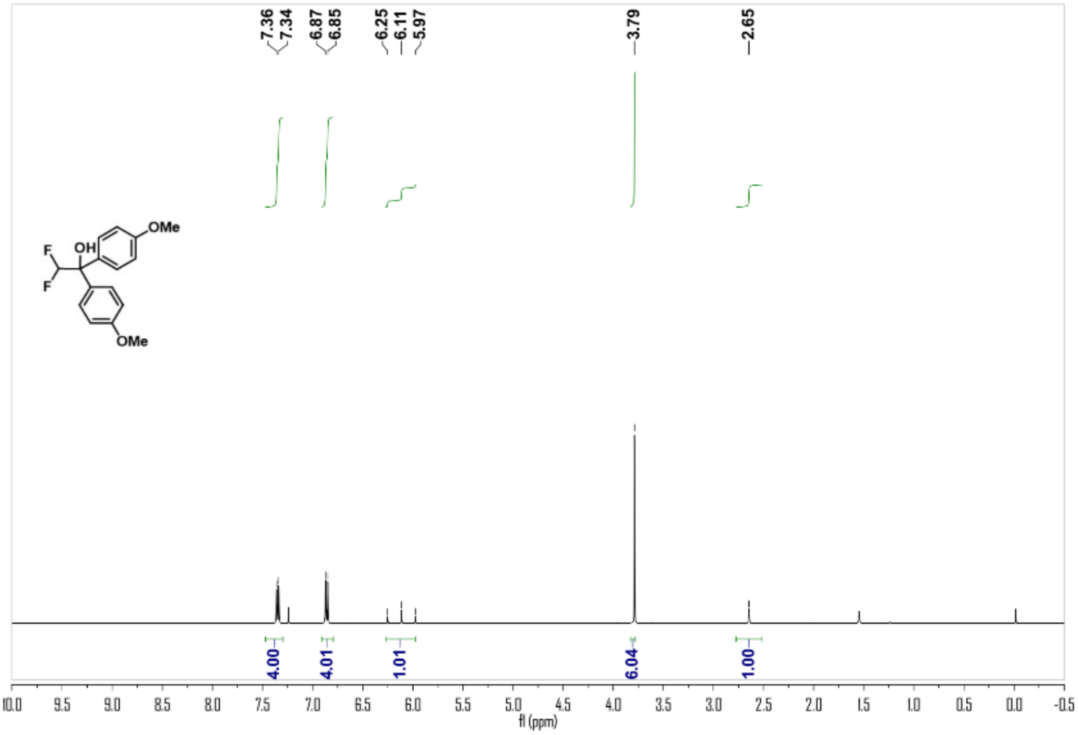

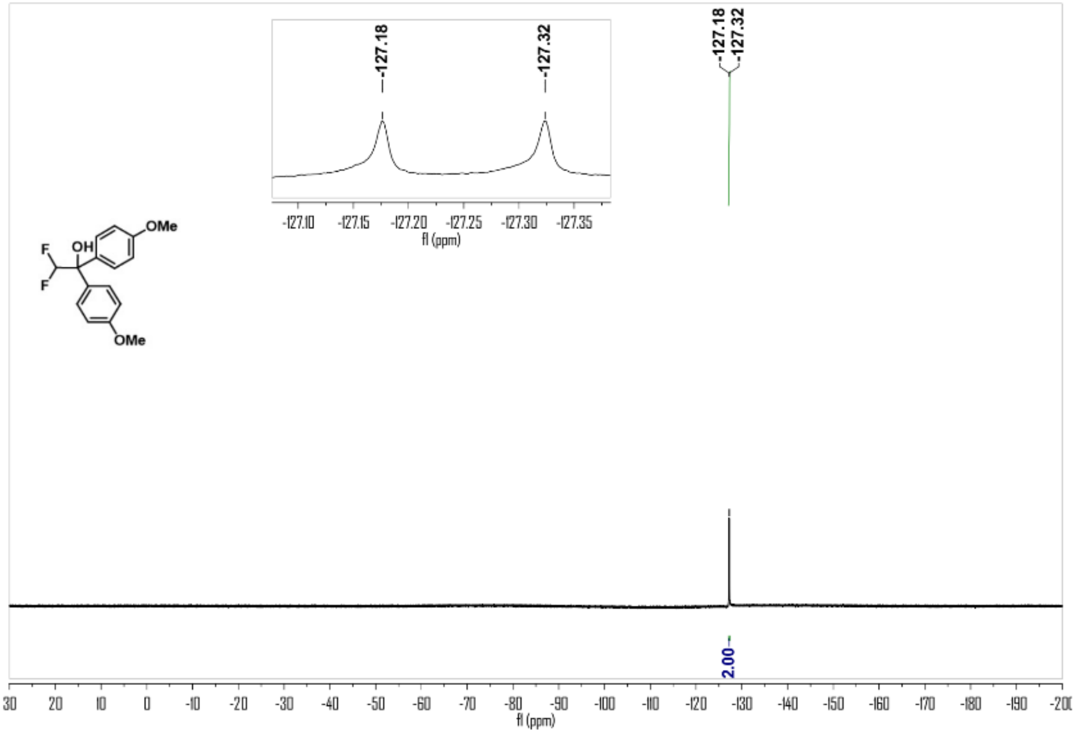

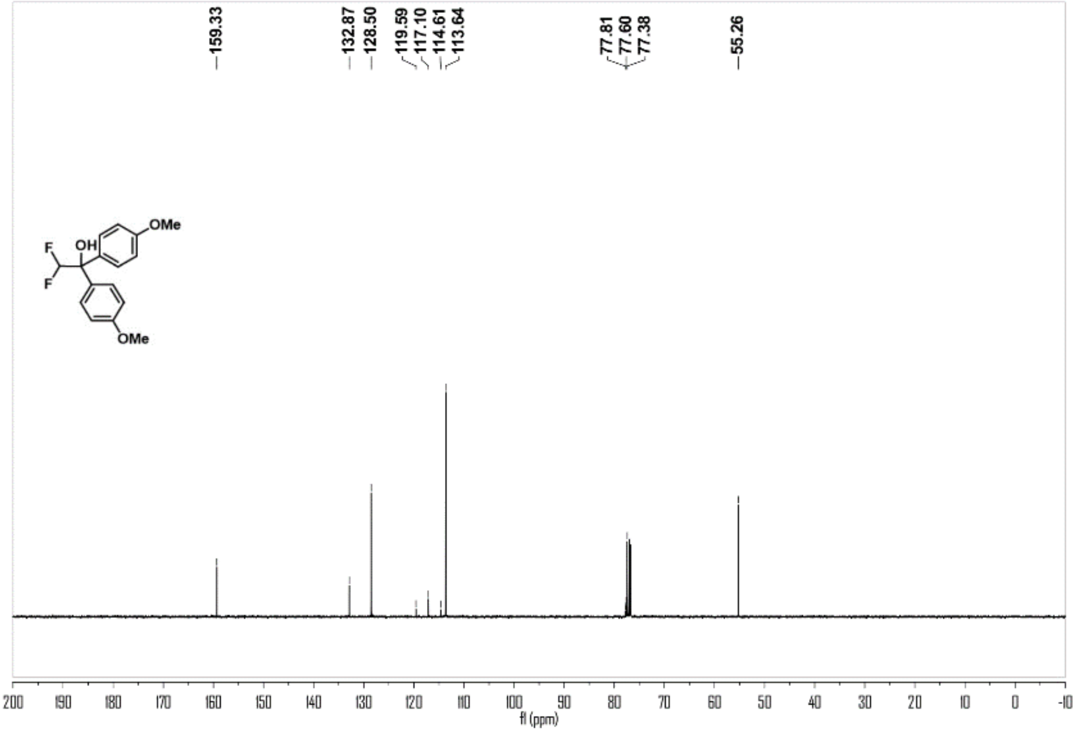

^{1}H, ^{19}F and ^{13}C NMR spectra of compound 1a-VII

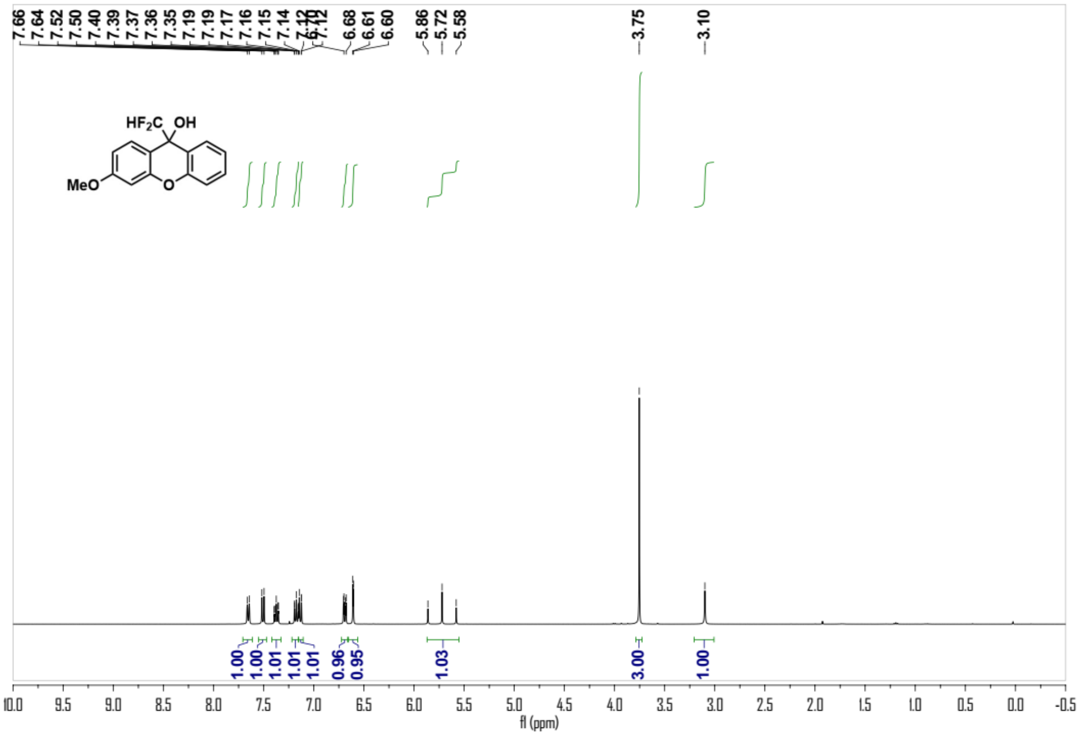

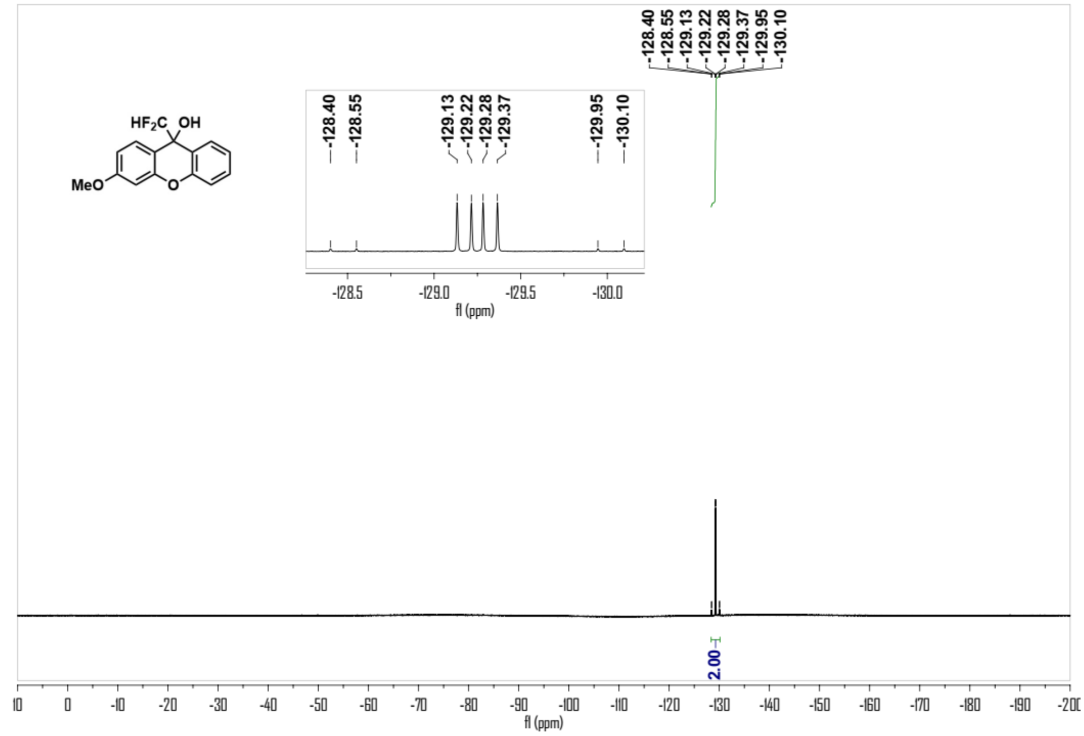
-128.40
-128.55
-129.13
-129.22
-129.28
-129.37
-129.95
-130.10
HF2C OH
MeO
O
2.00
f1 (ppm)

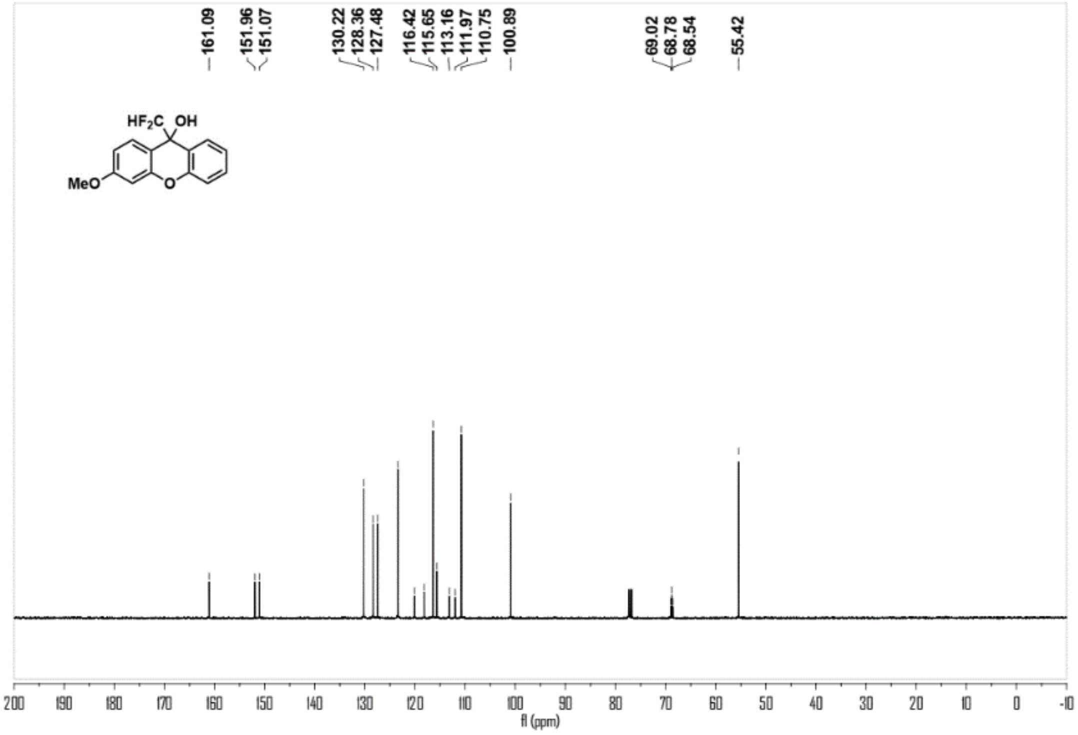
161.09
151.96
151.07
130.22
128.36
127.48
116.42
115.65
113.16
111.97
110.75
100.89
69.02
68.78
68.54
55.42
HF2C OH
MeO
O
f1 (ppm)

^{1}H, ^{19}F and ^{13}C NMR spectra of compound 1b

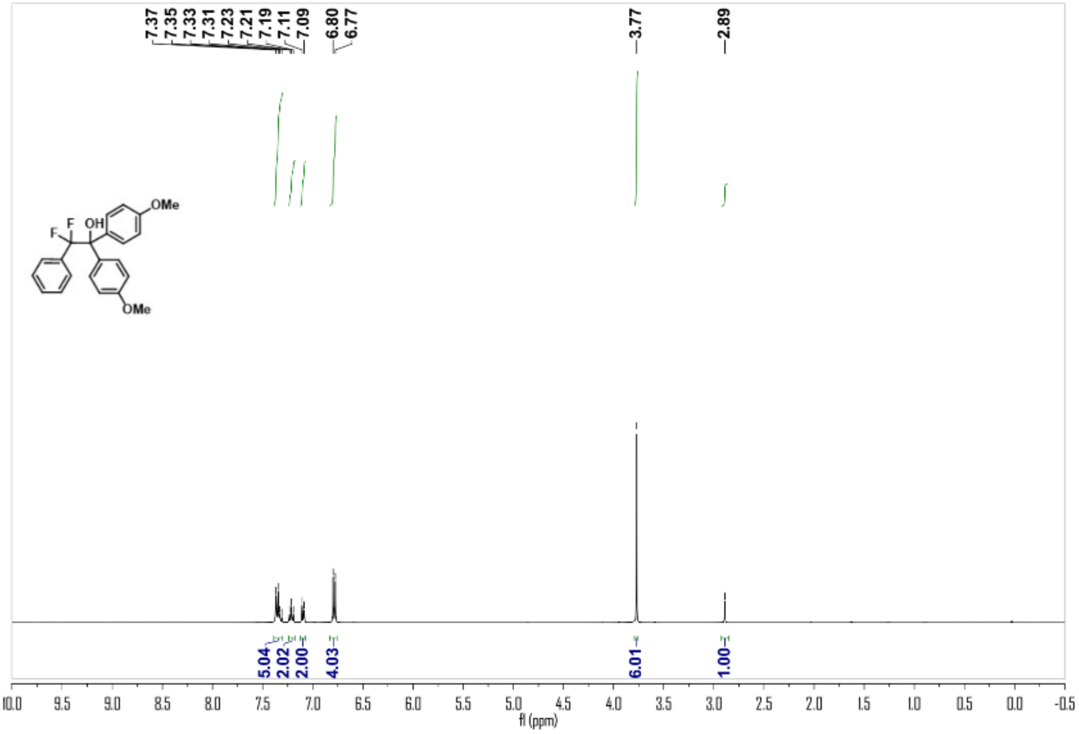

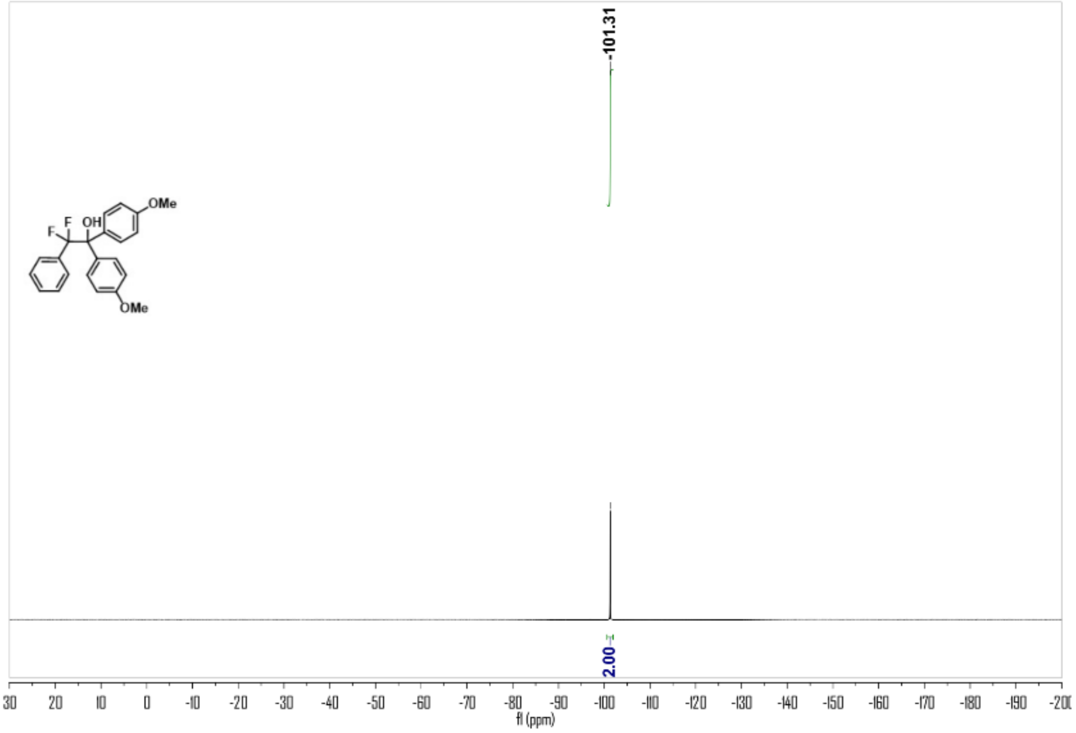

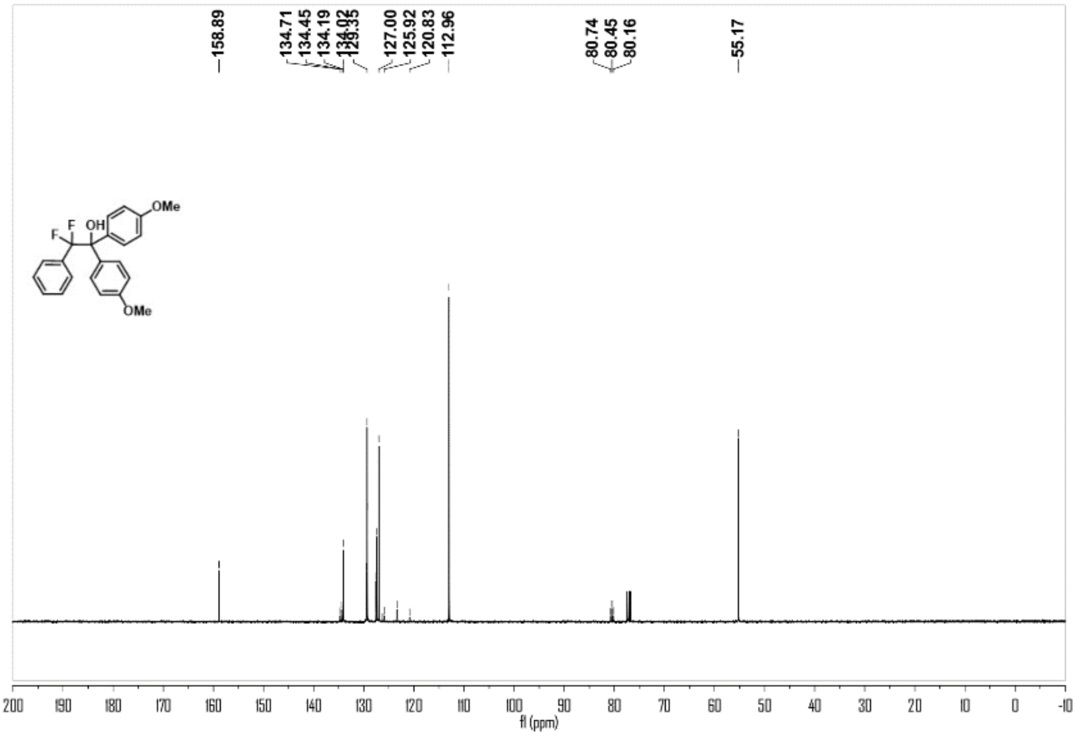

^{1}H, ^{19}F and ^{13}C NMR spectra of compound 1c

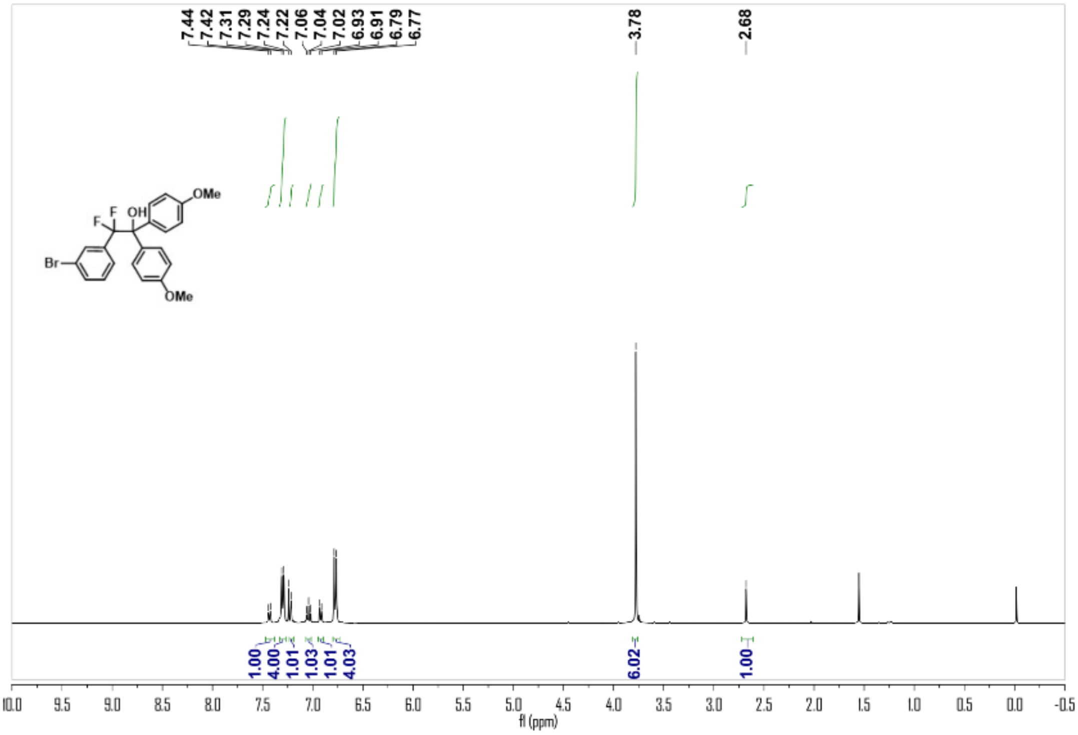

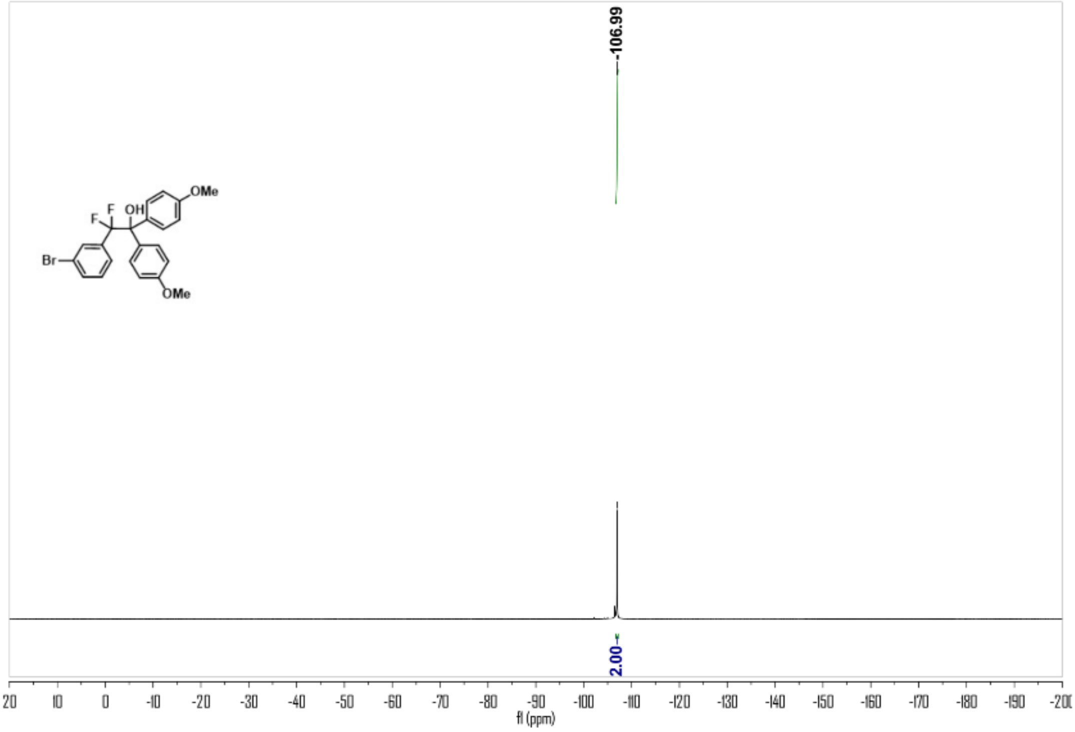
-106.99
OMe
F
F
OH
Br
OMe
2.00
20 10 0 -10 -20 -30 -40 -50 -60 -70 -80 -90 -100 -110 -120 -130 -140 -150 -160 -170 -180 -190 -200
fl (ppm)

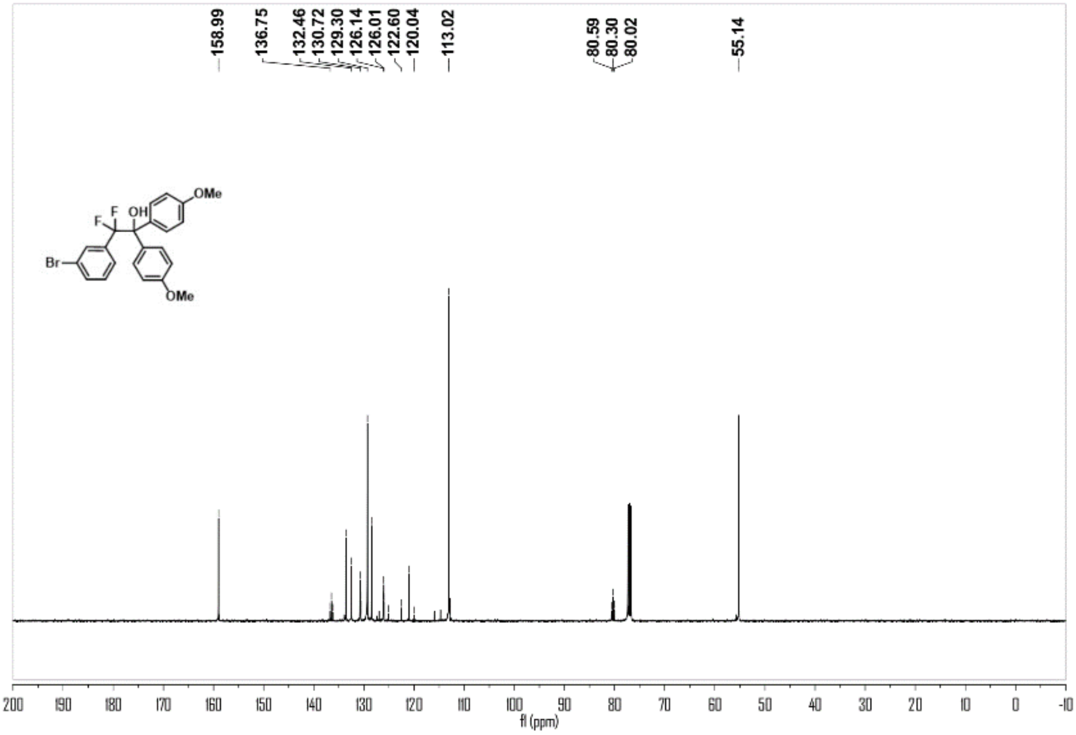
158.99
136.75
132.46
130.72
129.30
126.14
126.01
122.60
120.04
113.02
80.59
80.30
80.02
55.14
OMe
F
F
OH
Br
OMe
200 190 180 170 160 150 140 130 120 110 100 90 80 70 60 50 40 30 20 10 0 -10
fl (ppm)

^{1}H, ^{19}F and ^{13}C NMR spectra of compound 1d

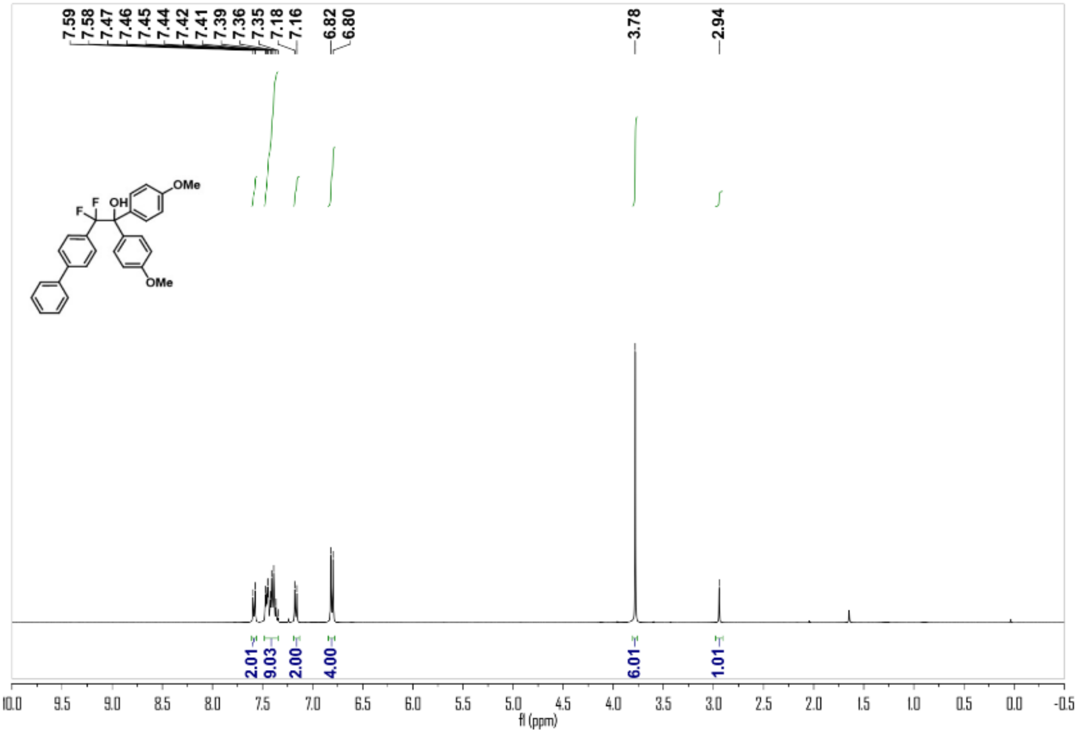

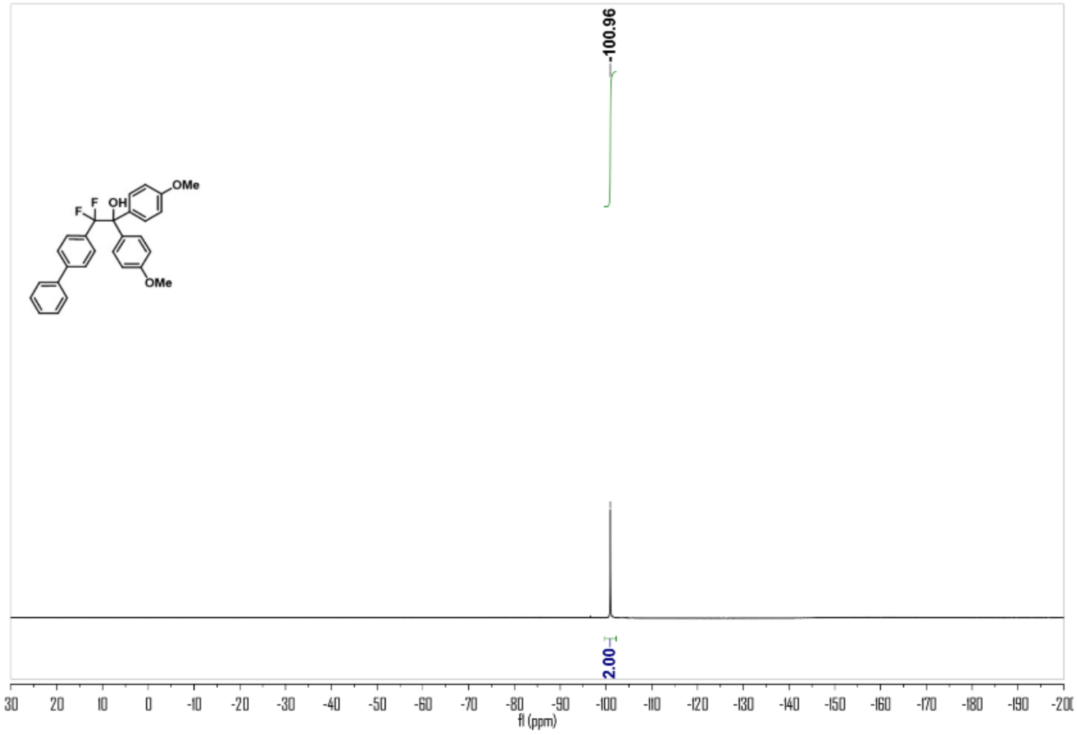

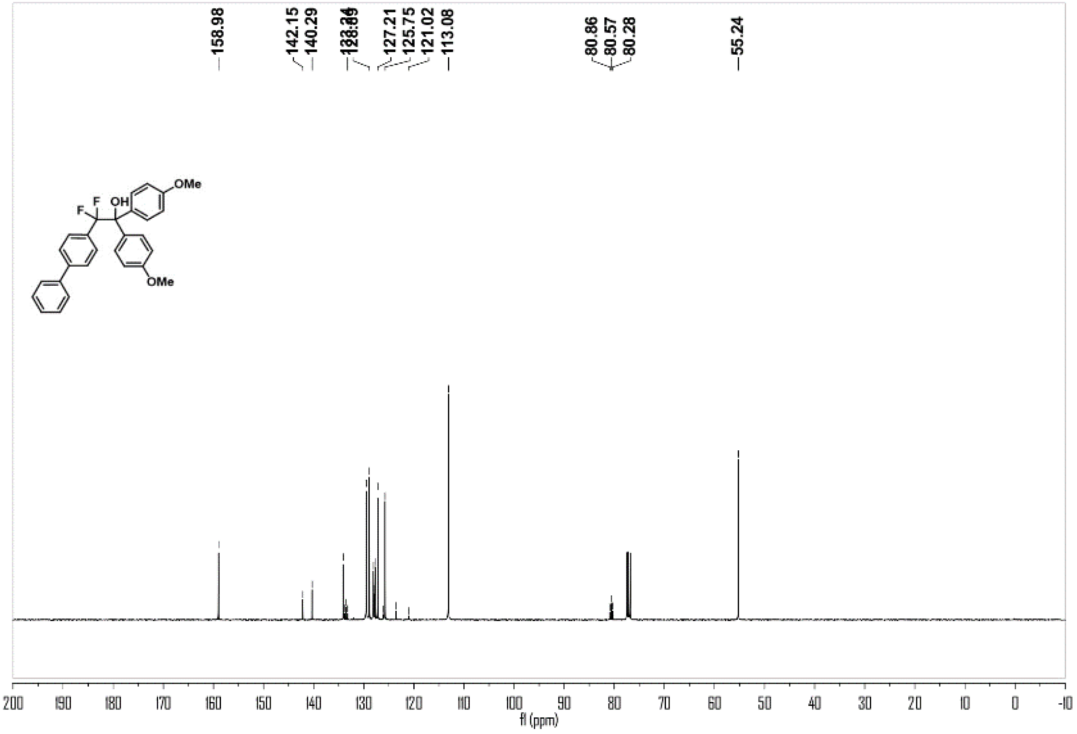

^{1}H, ^{19}F and ^{13}C NMR spectra of compound 1e

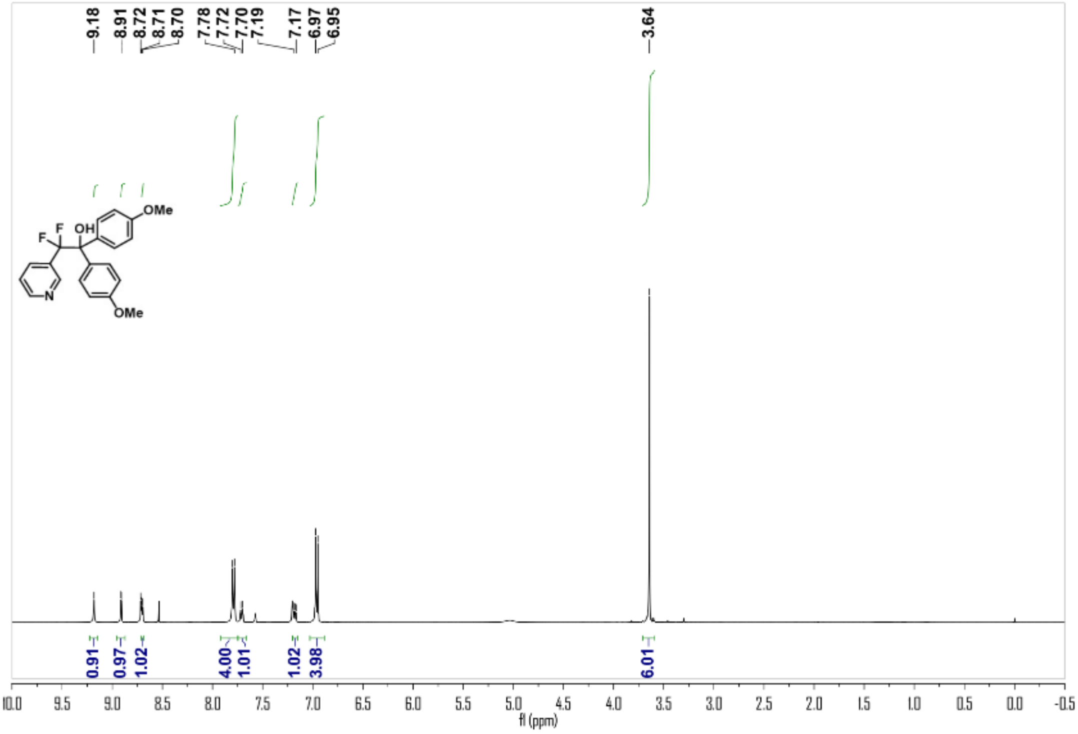

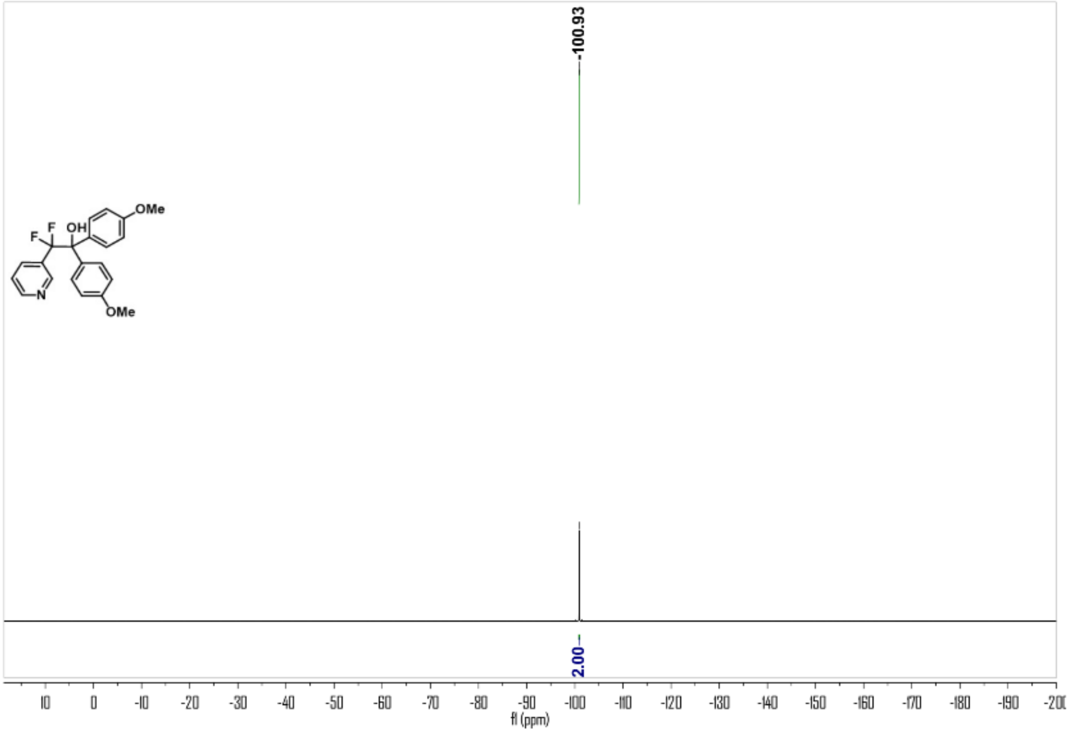
-100.93
OMe
F
F
OH
N
OMe
2.00
10
0
-10
-20
-30
-40
-50
-60
-70
-80
-90
-100
-110
-120
-130
-140
-150
-160
-170
-180
-190
f1 (ppm)

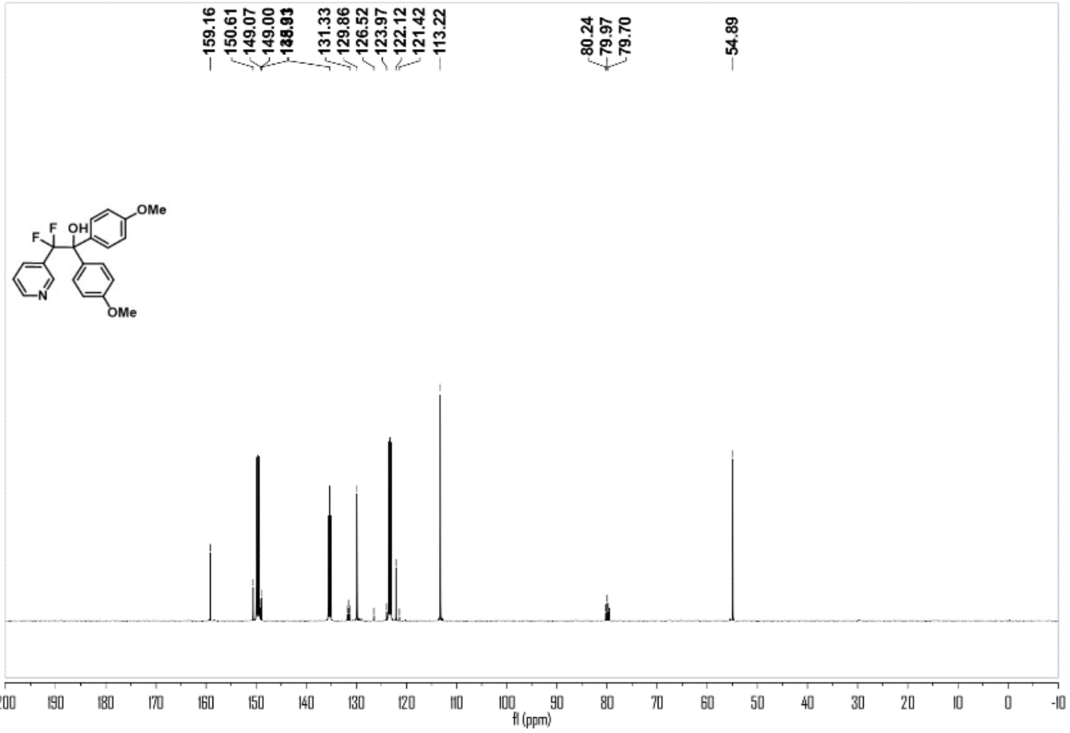
159.16
150.61
149.07
149.00
131.33
129.86
126.52
123.97
122.12
121.42
113.22
80.24
79.97
79.70
54.89
OMe
F
F
OH
N
OMe
200
190
180
170
160
150
140
130
120
110
100
90
80
70
60
50
40
30
20
10
0
-10
f1 (ppm)

^{1}H, ^{19}F and ^{13}C NMR spectra of compound 1f

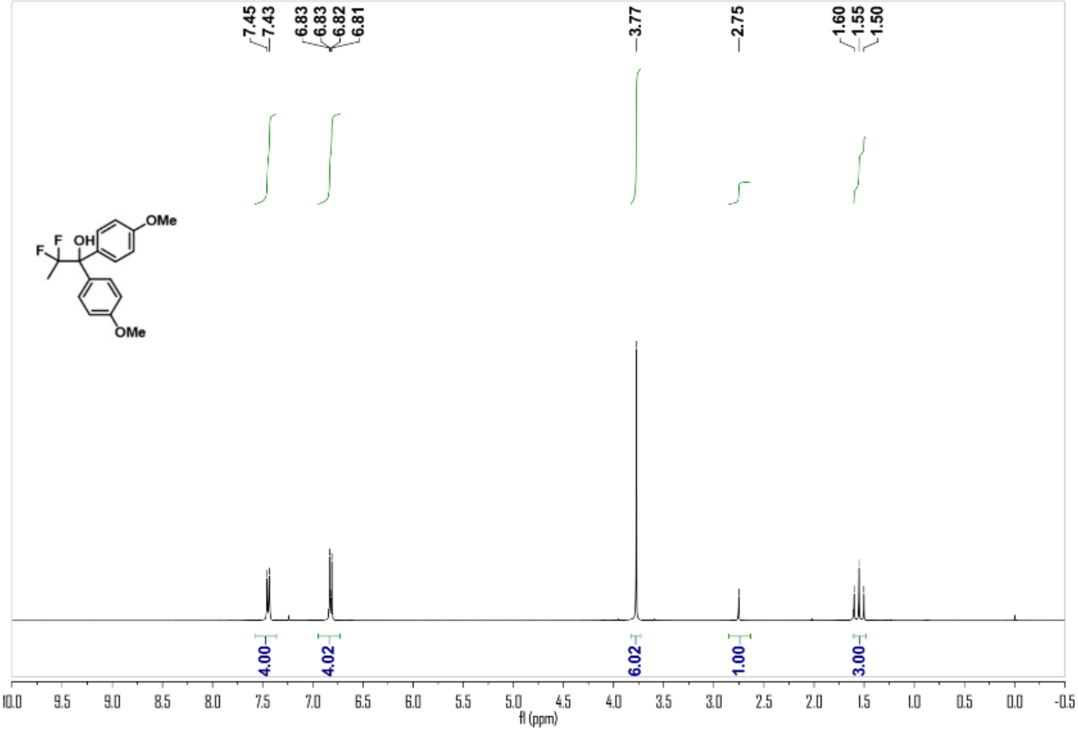

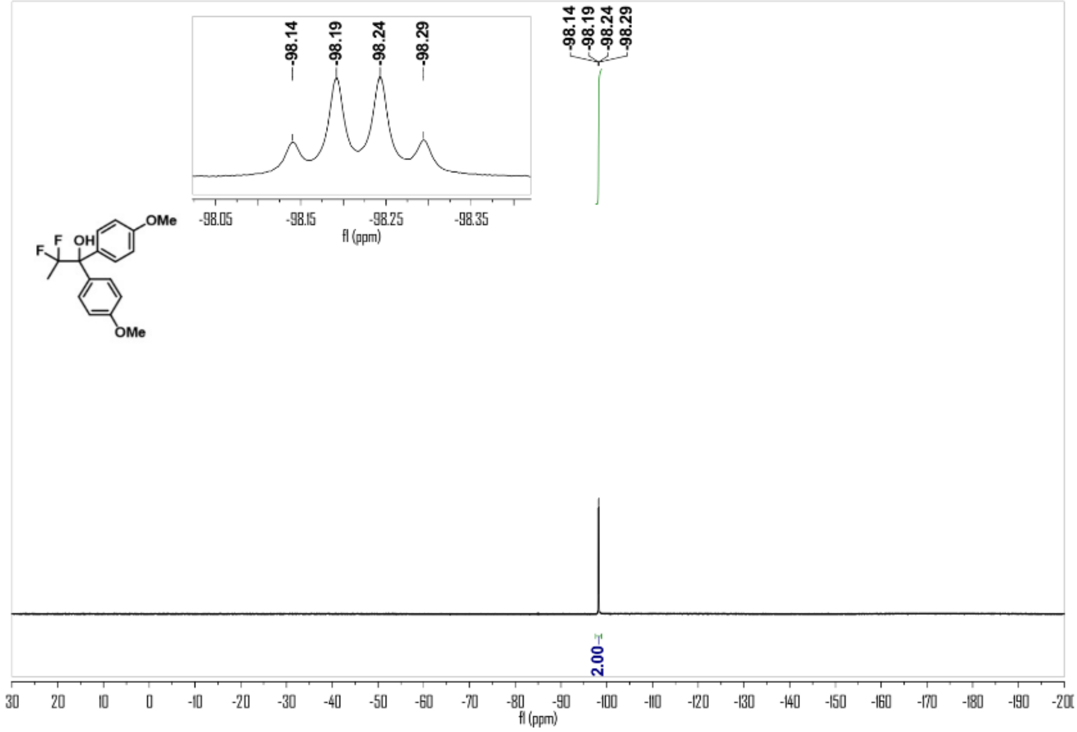

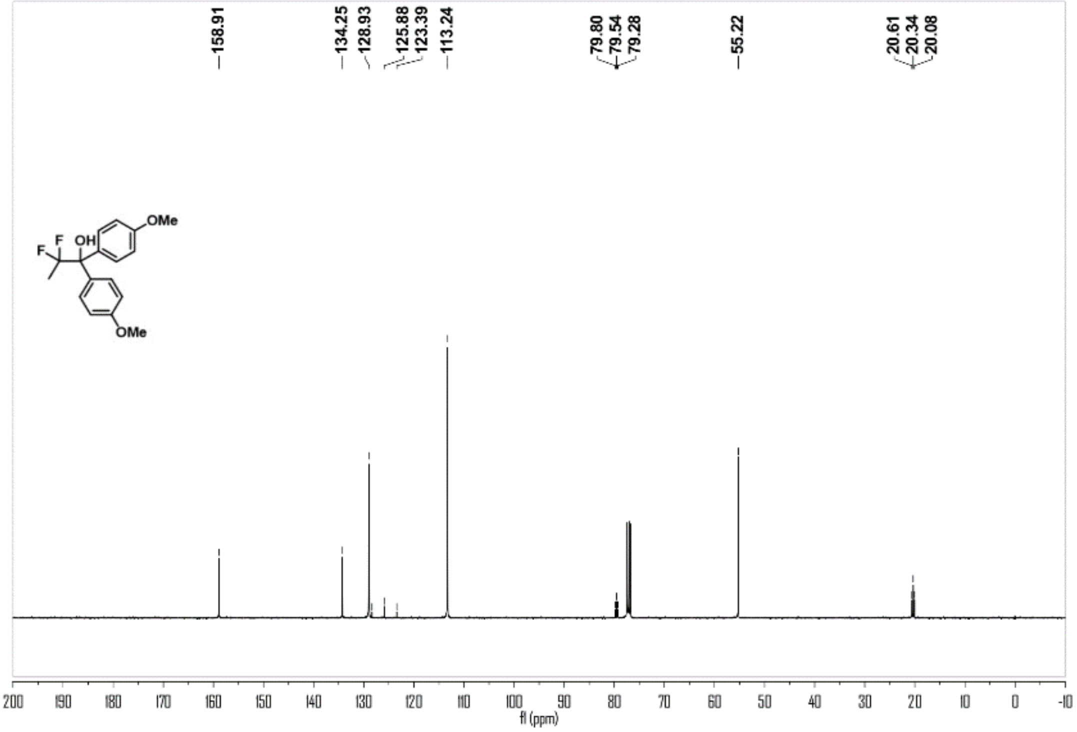

^{1}H, ^{19}F and ^{13}C NMR spectra of compound 1g

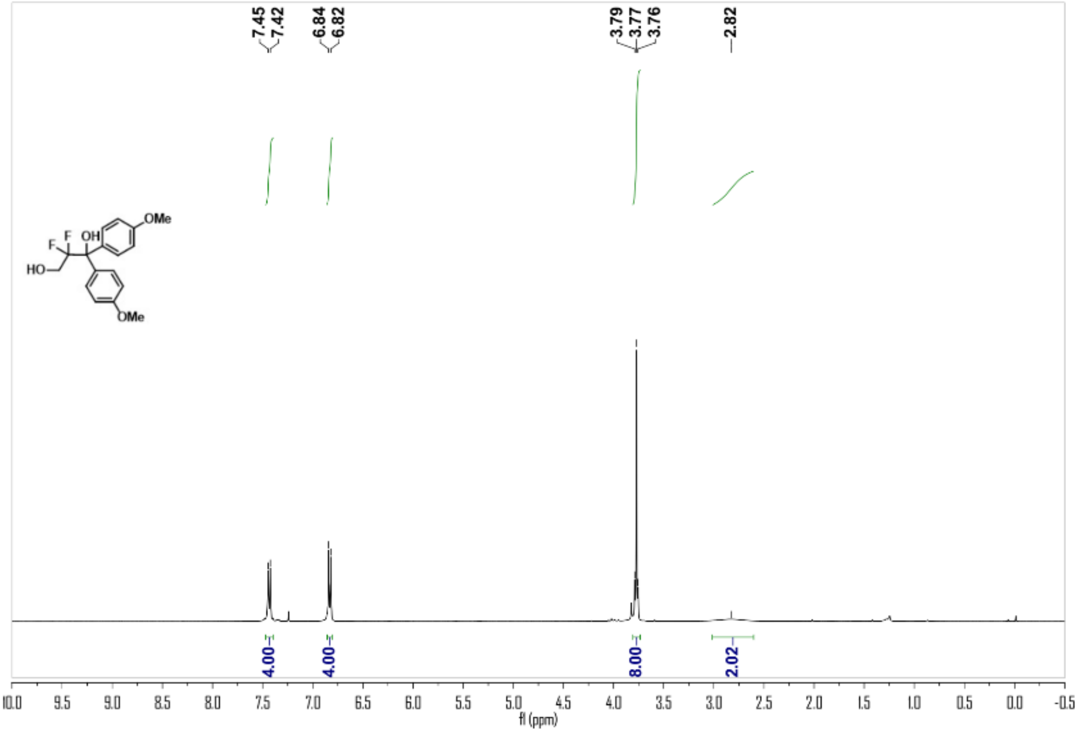

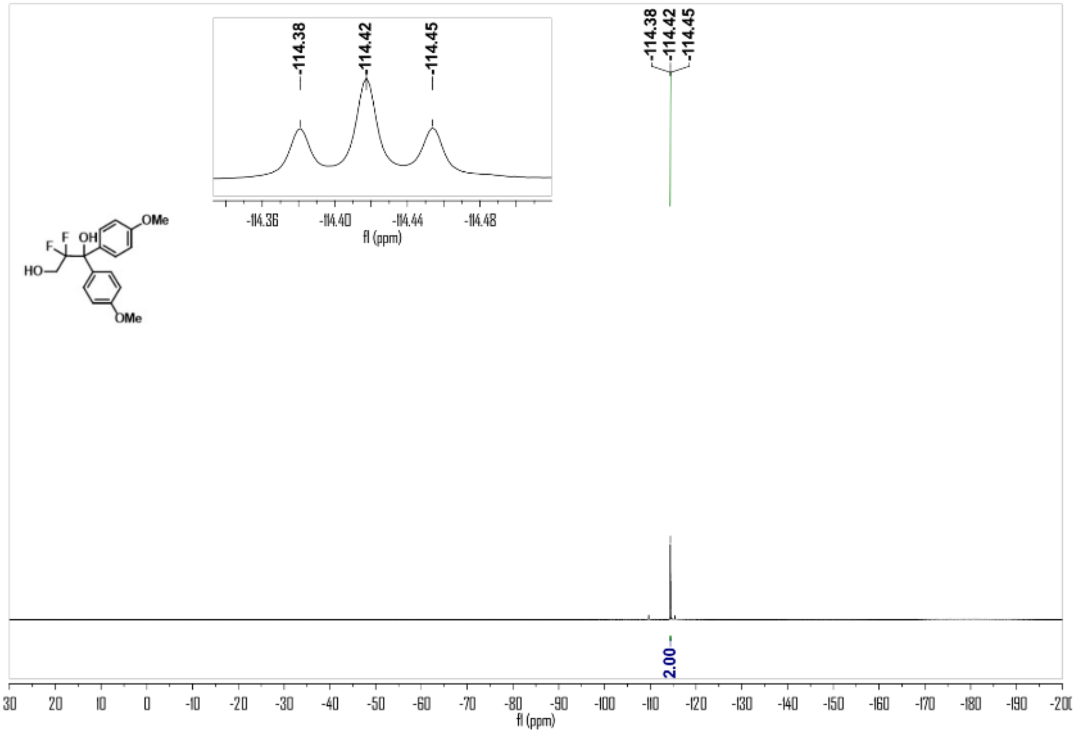
-114.38
-114.42
-114.45
OMe
F F OH
HO
OMe
2.00
fl (ppm)

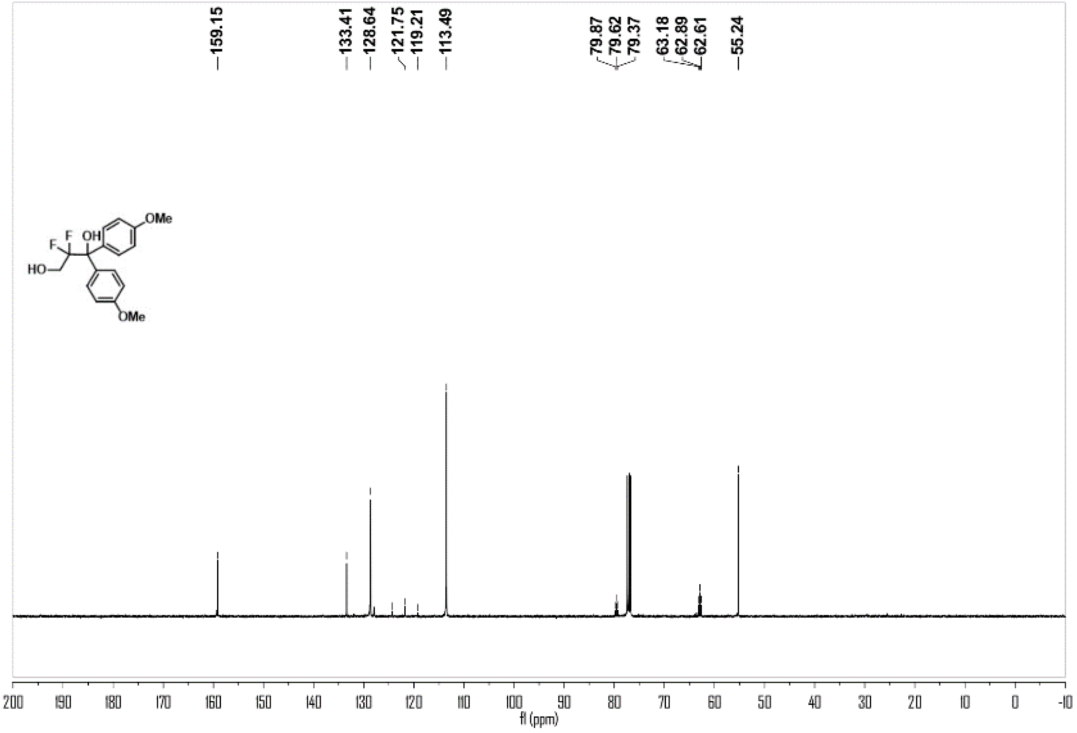
159.15
133.41
128.64
121.75
119.21
113.49
79.87
79.62
79.37
63.18
62.89
62.61
55.24
OMe
F F OH
HO
OMe
fl (ppm)

^{1}H, ^{19}F and ^{13}C NMR spectra of compound 1h

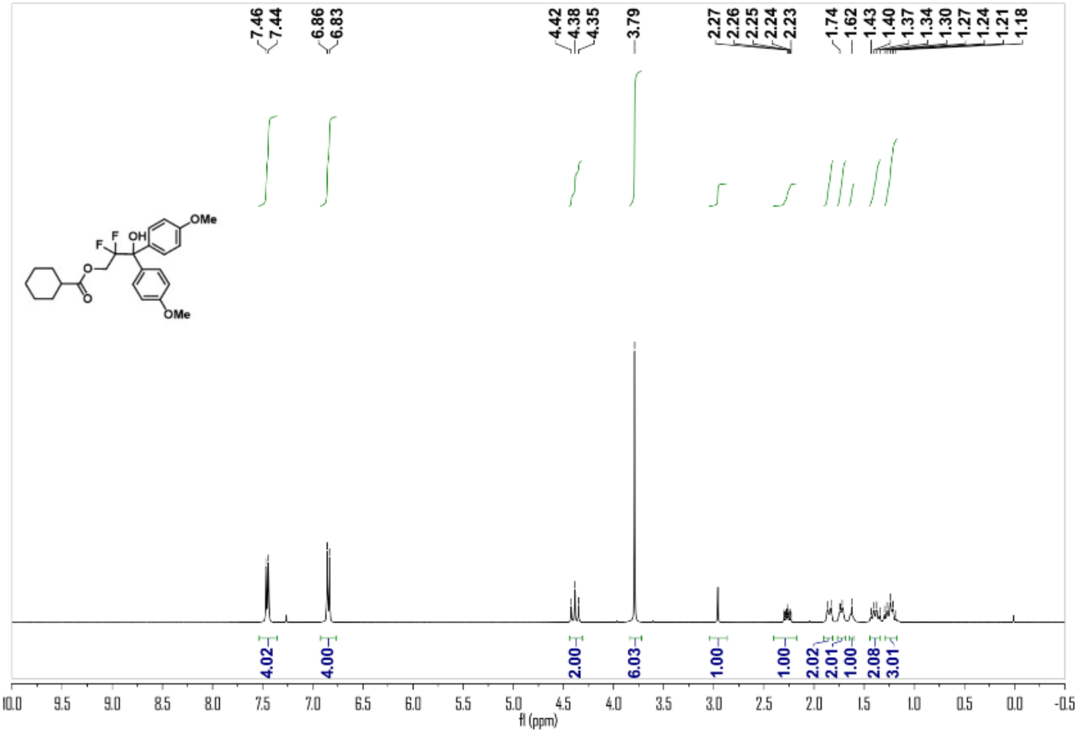

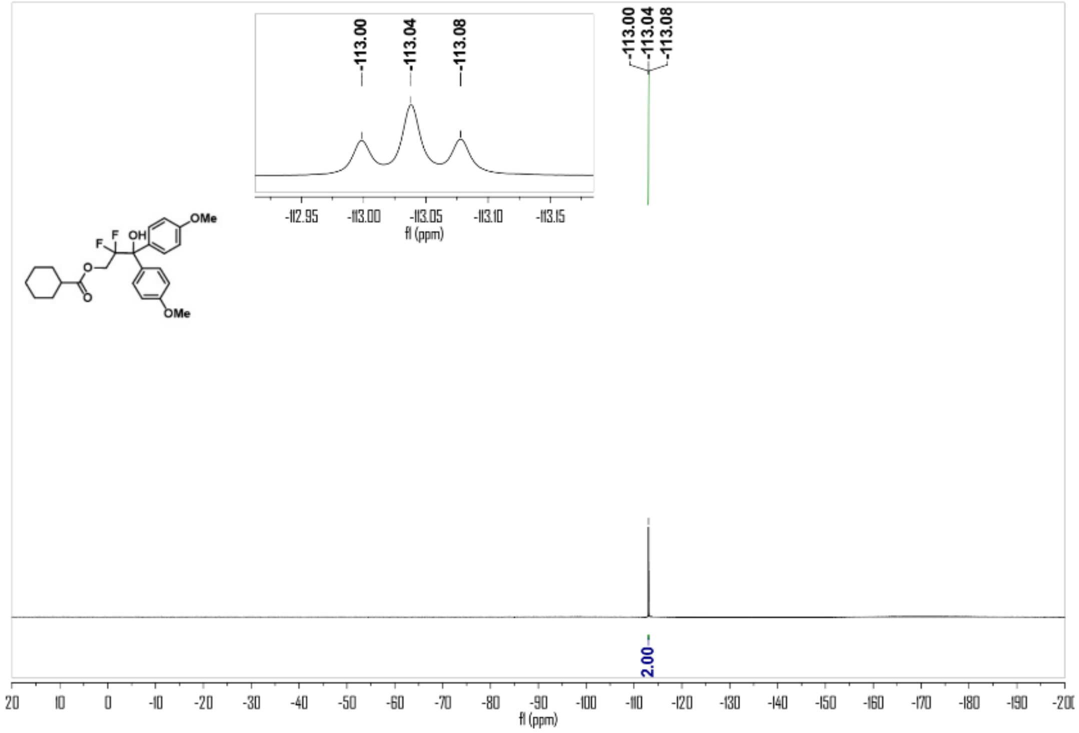

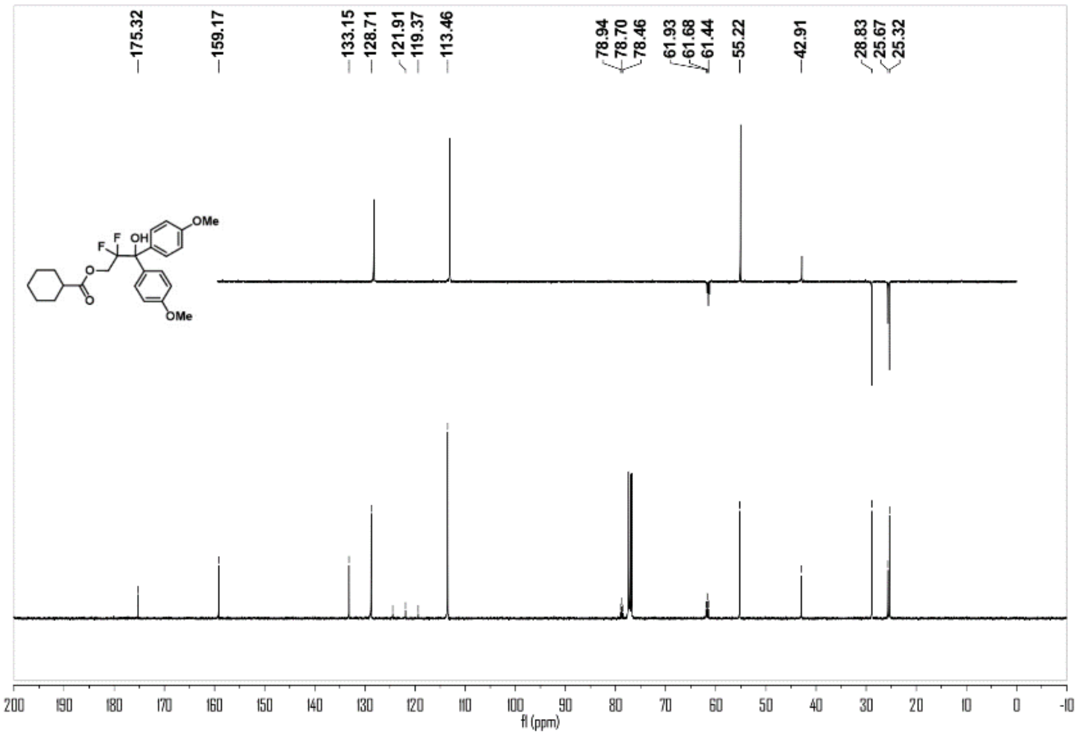

^{1}H, ^{19}F and ^{13}C NMR spectra of compound 1i

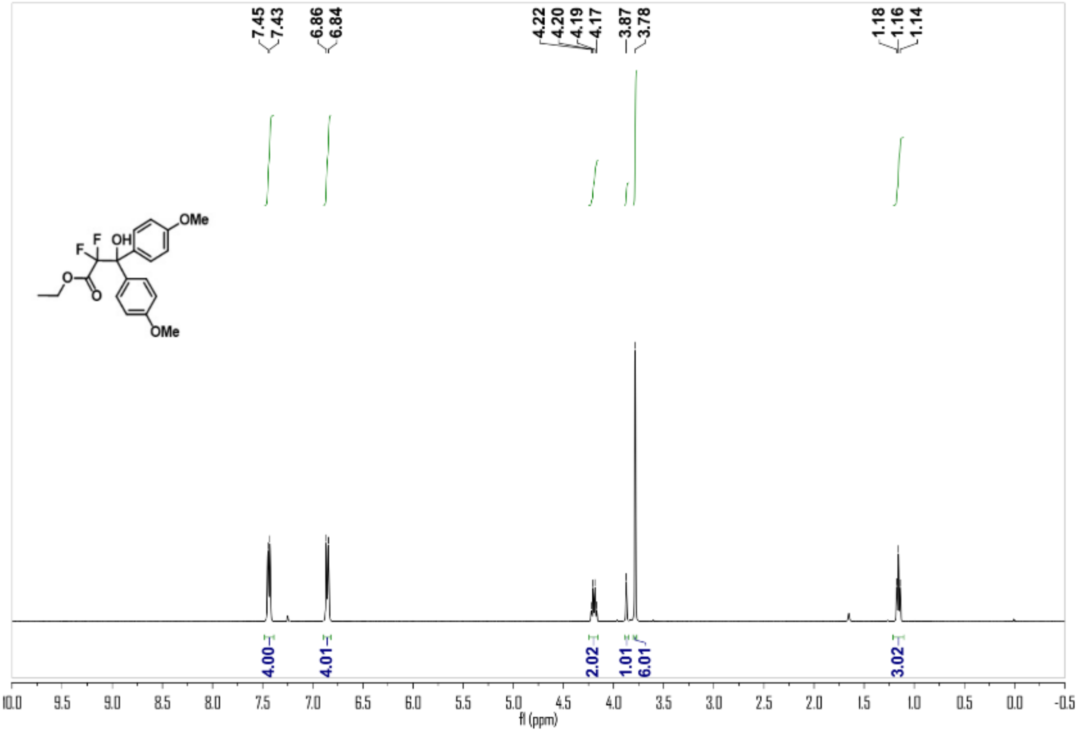

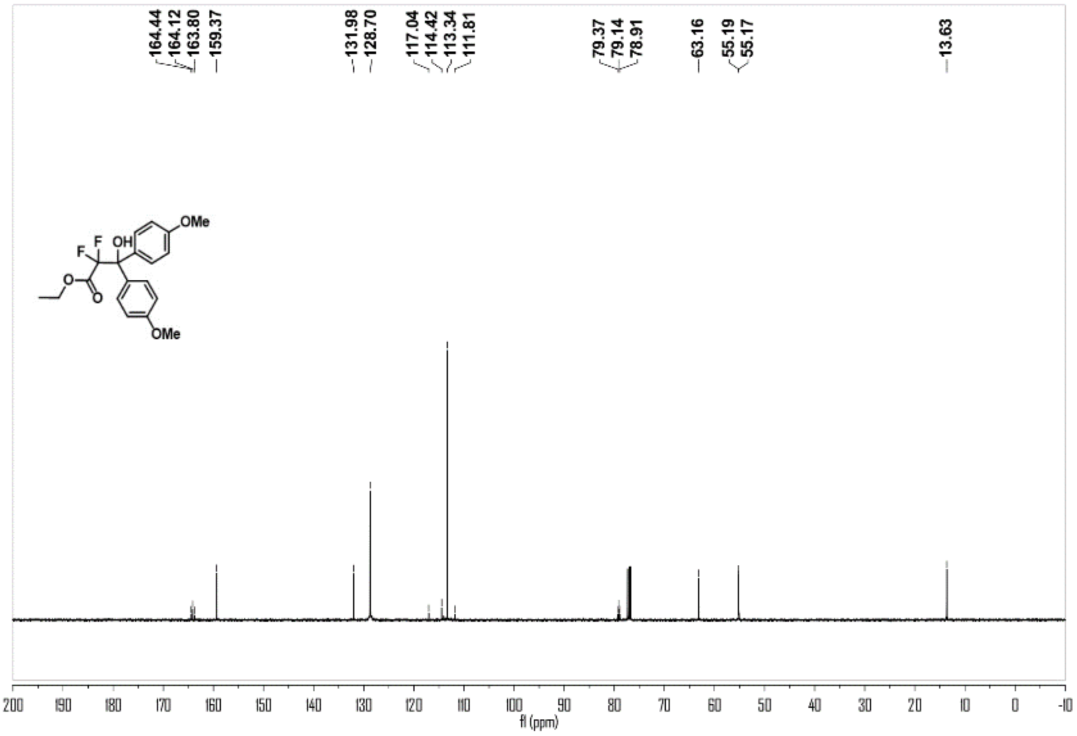
164.44
164.12
163.80
159.37
131.98
128.70
117.04
114.42
113.34
111.81
79.37
79.14
78.91
63.16
55.19
55.17
13.63
OMe
F
F
OH
O
O
OMe
200 190 180 170 160 150 140 130 120 110 100 90 80 70 60 50 40 30 20 10 0 -10
f1 (ppm)

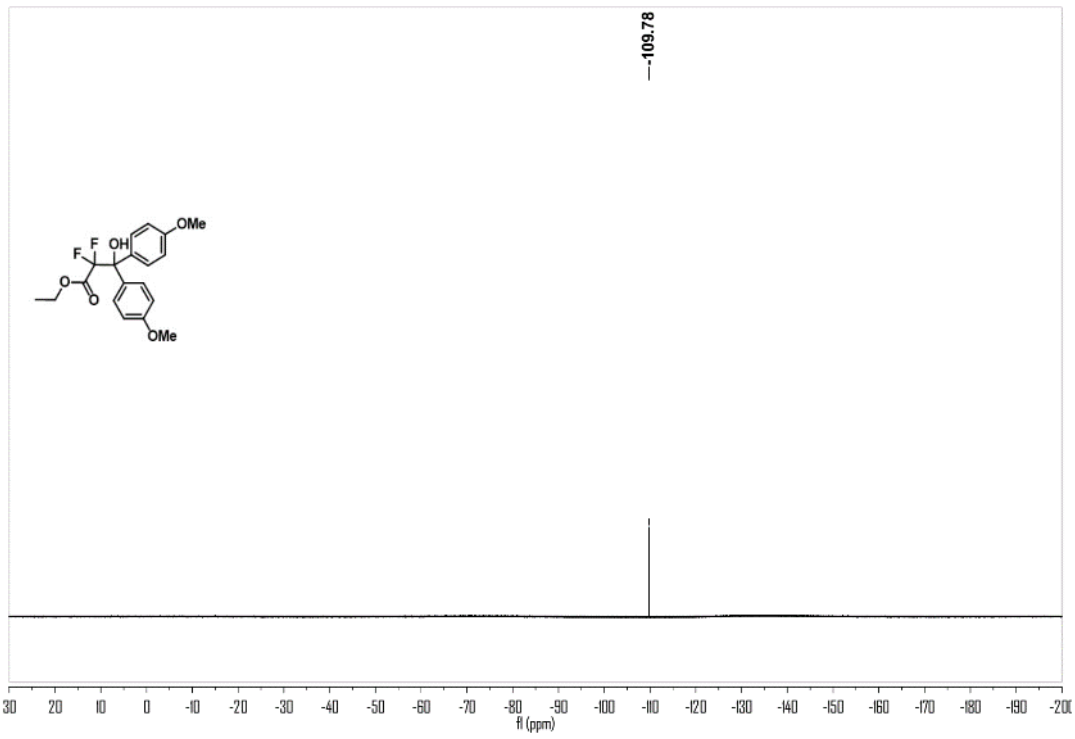
-109.78
OMe
F
F
OH
O
O
OMe
30 20 10 0 -10 -20 -30 -40 -50 -60 -70 -80 -90 -100 -110 -120 -130 -140 -150 -160 -170 -180 -190 -200
f1 (ppm)

^{1}H, and ^{19}F spectra of compound 1j

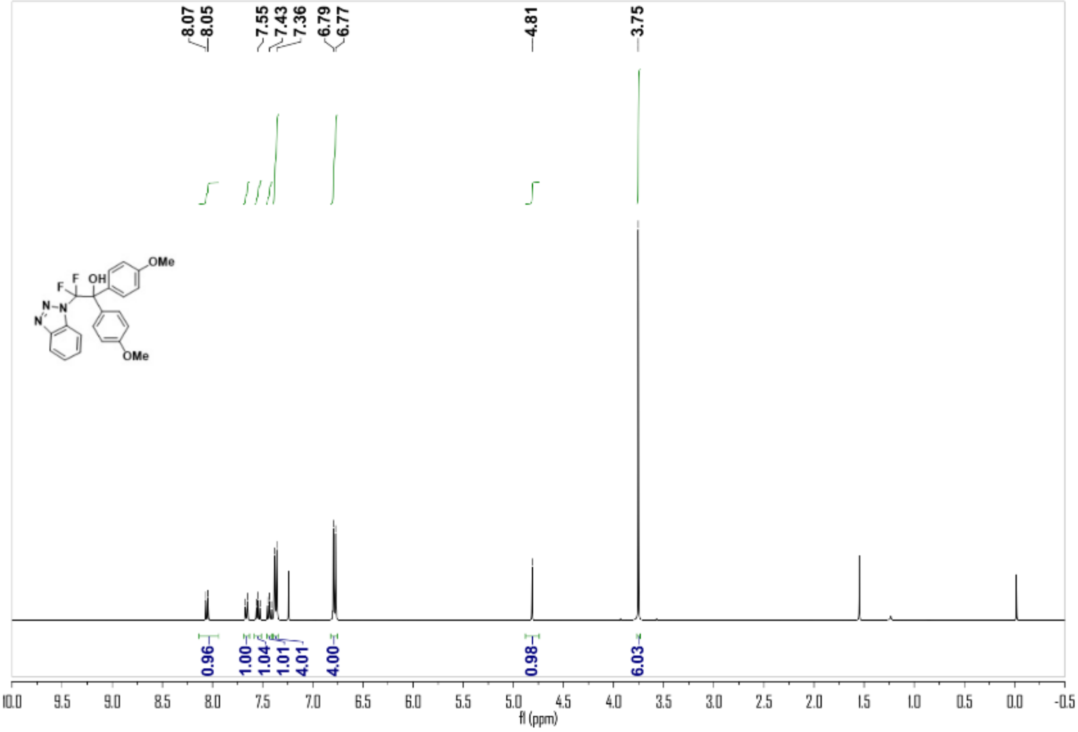

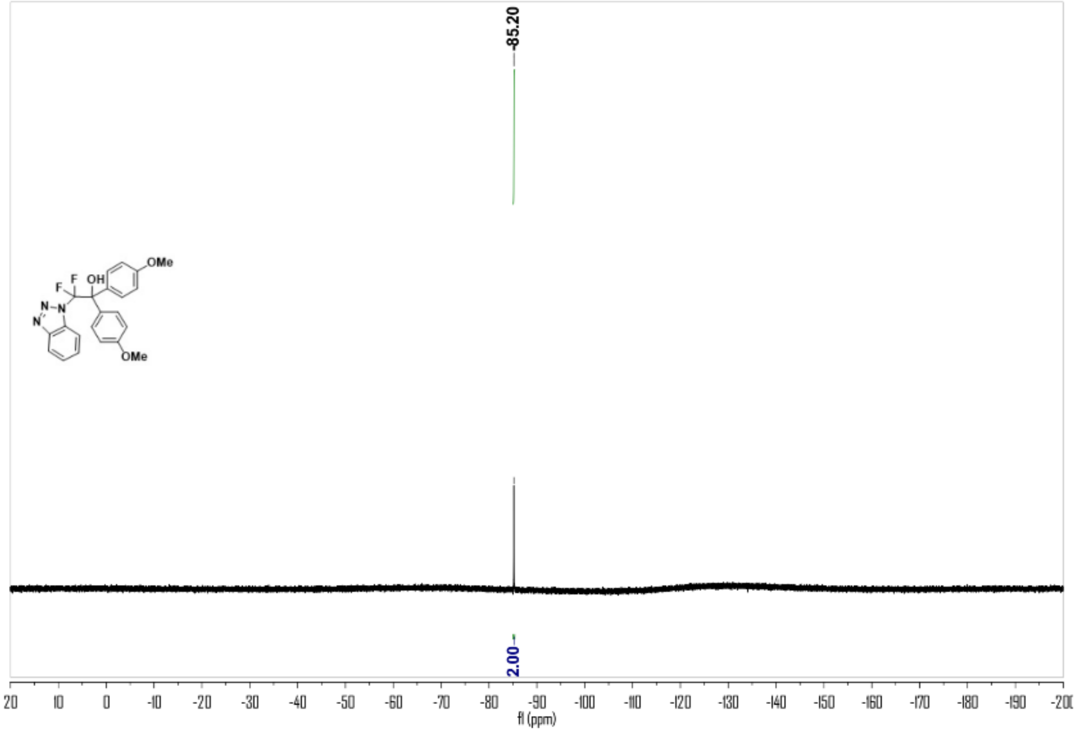

^{1}H, ^{19}F and ^{13}C NMR spectra of compound 1k

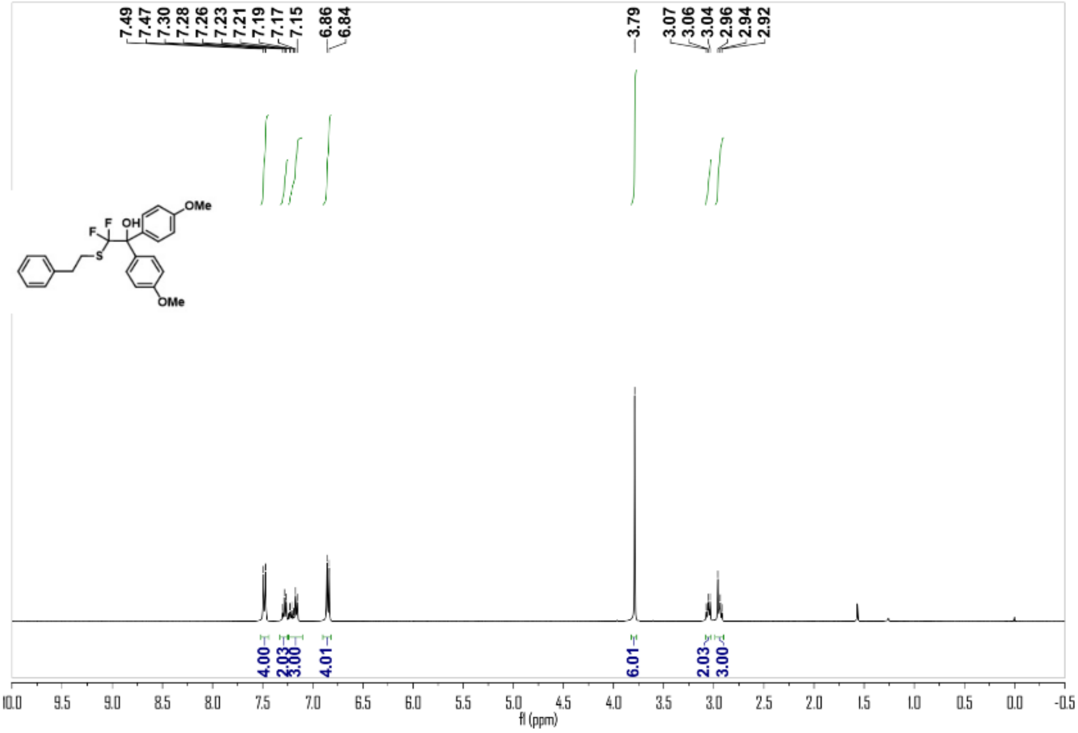

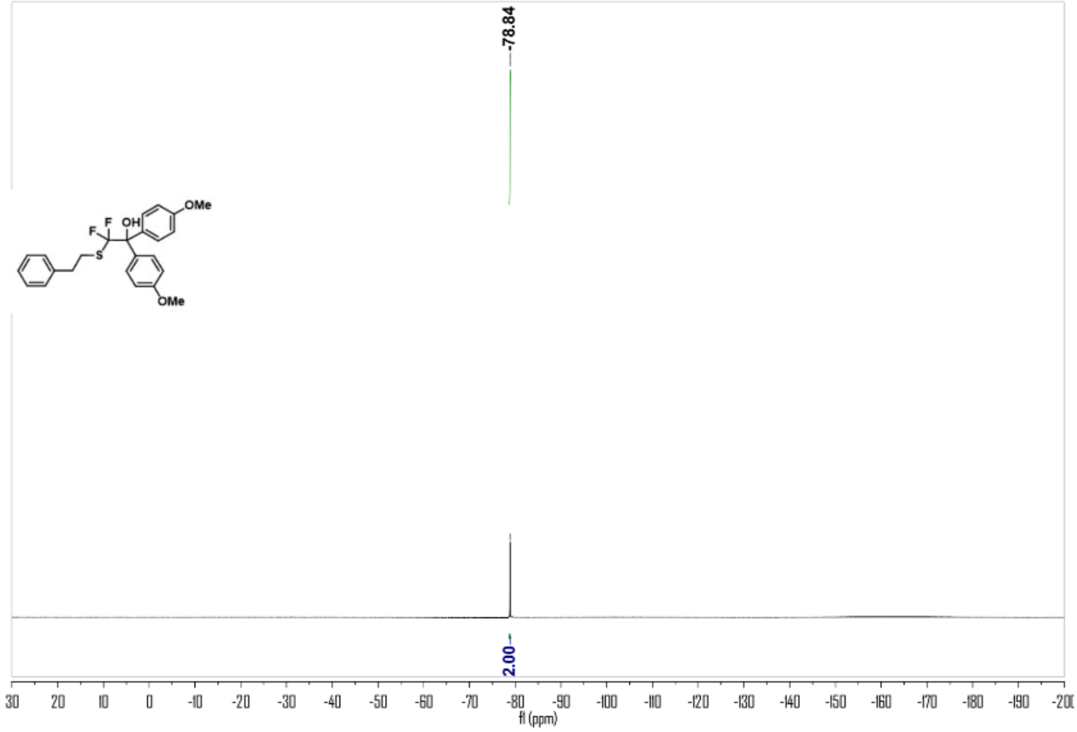

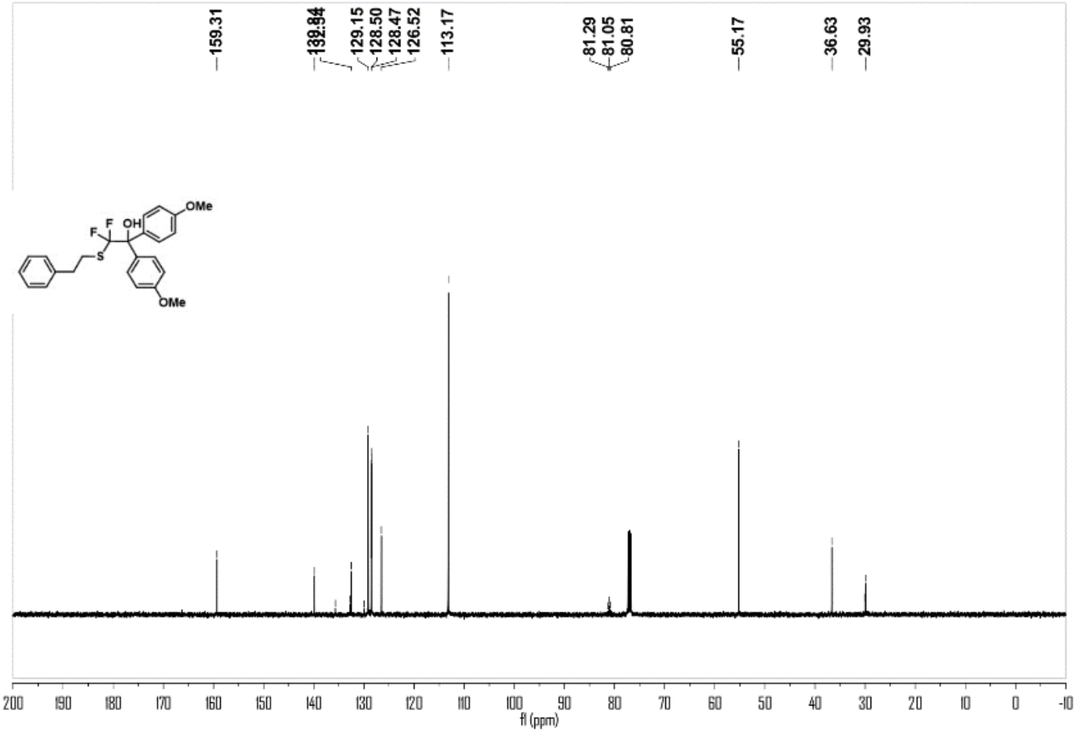

^{1}H, ^{19}F and ^{13}C NMR spectra of compound 1i

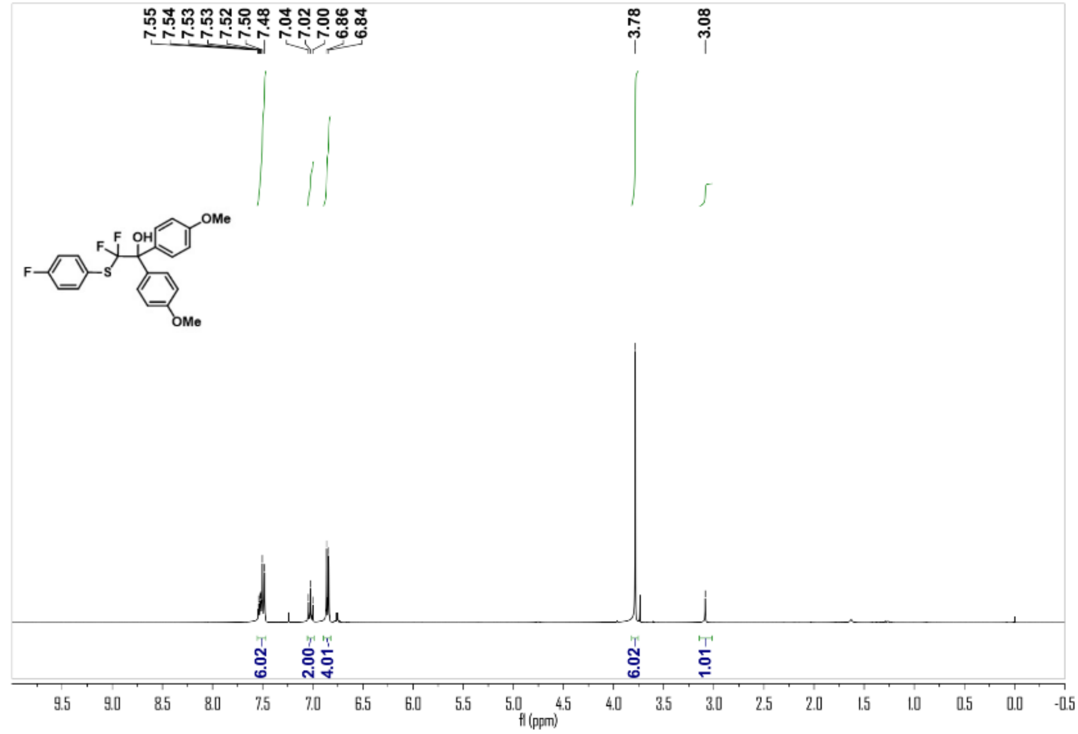

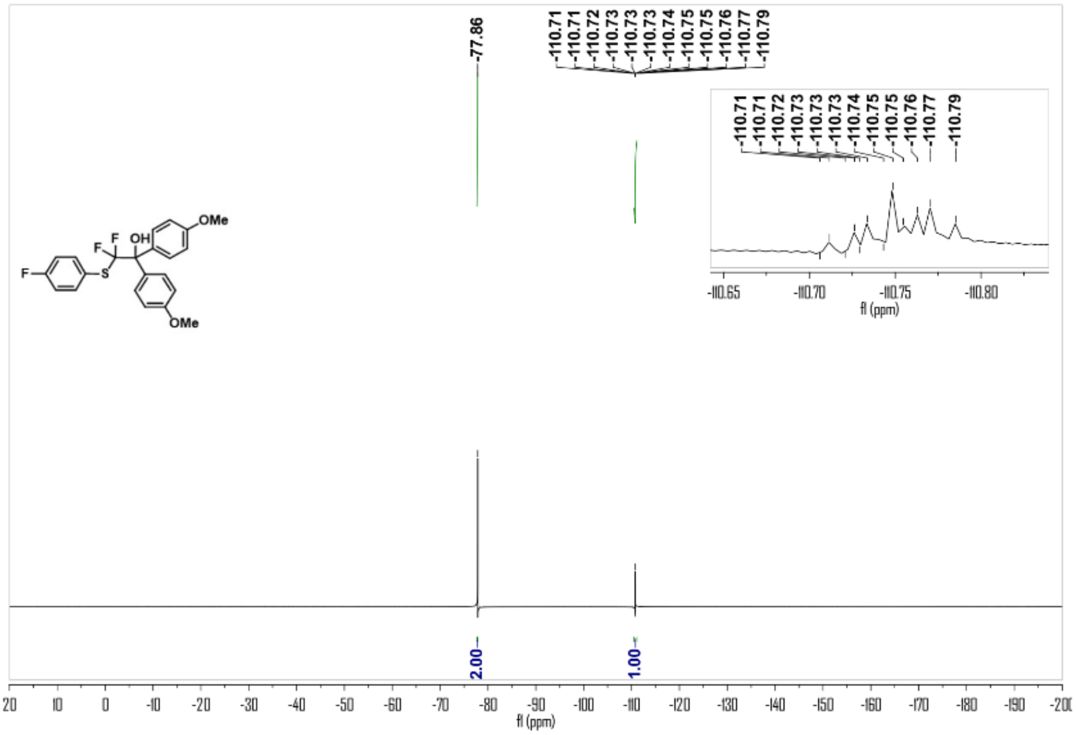
-77.86
-110.71
-110.71
-110.72
-110.73
-110.73
-110.73
-110.74
-110.75
-110.75
-110.76
-110.77
-110.79
OMe
F
F
OH
F
S
OMe
-110.65
-110.70
-110.75
-110.80
f1 (ppm)
2.00
1.00
f1 (ppm)

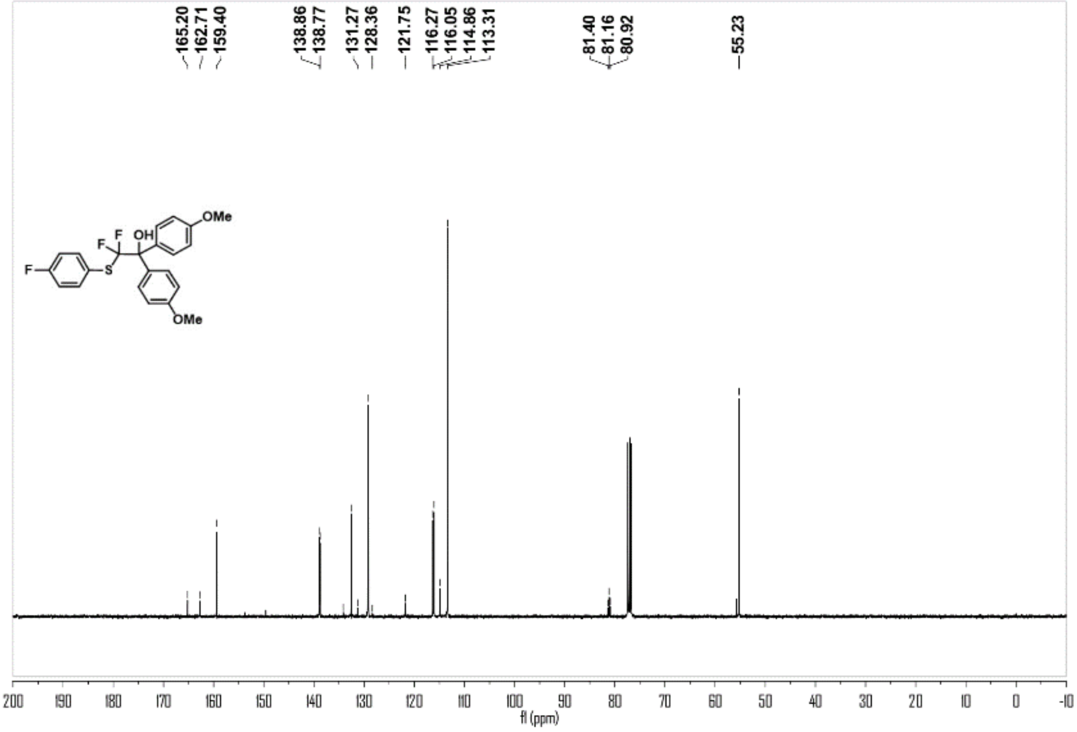
165.20
162.71
159.40
138.86
138.77
131.27
128.36
121.75
116.27
116.05
114.86
113.31
81.40
81.16
80.92
55.23
OMe
F
F
OH
F
S
OMe
f1 (ppm)

^{1}H, ^{19}F and ^{13}C NMR spectra of compound 1m

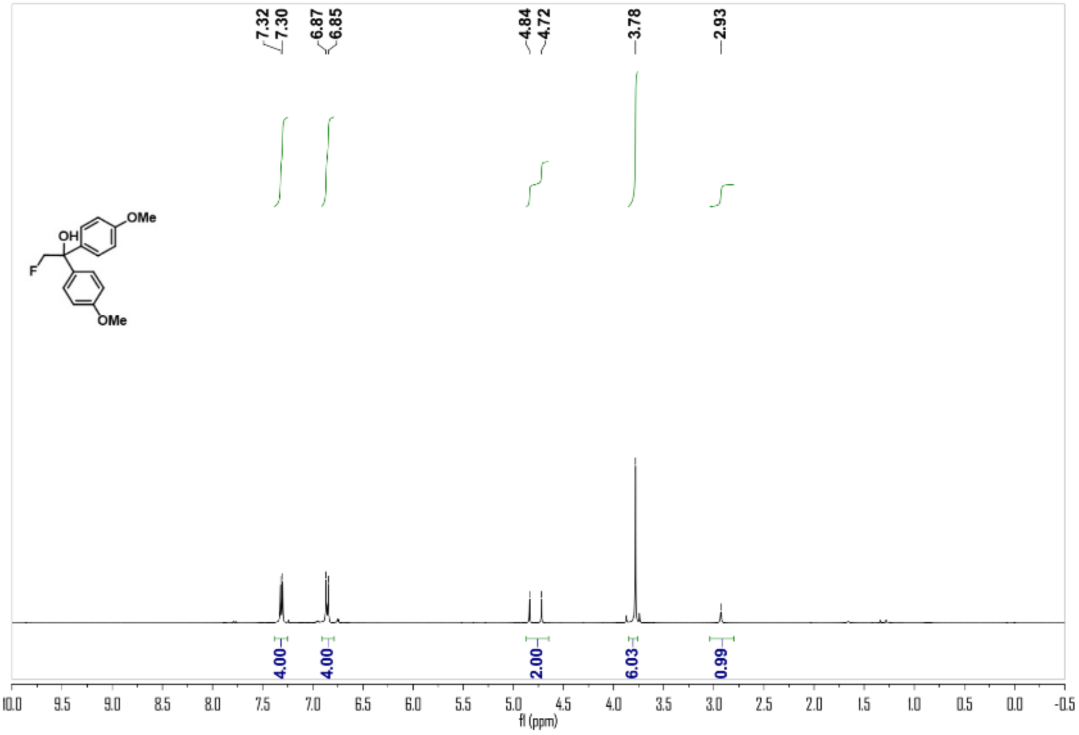

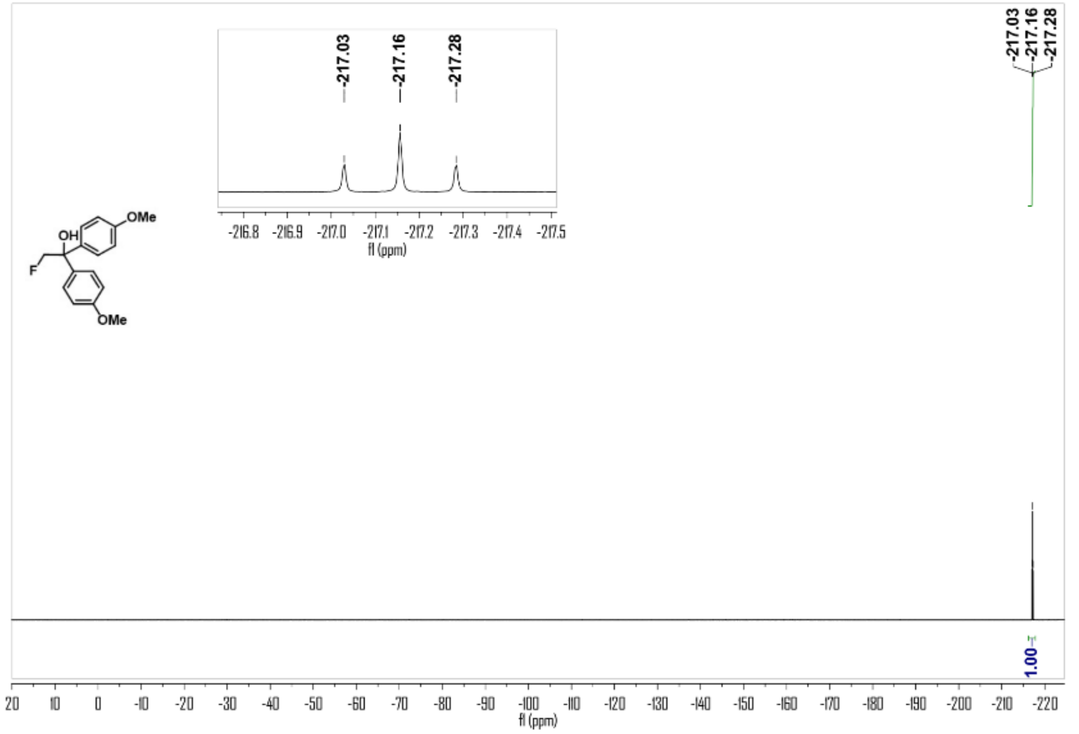

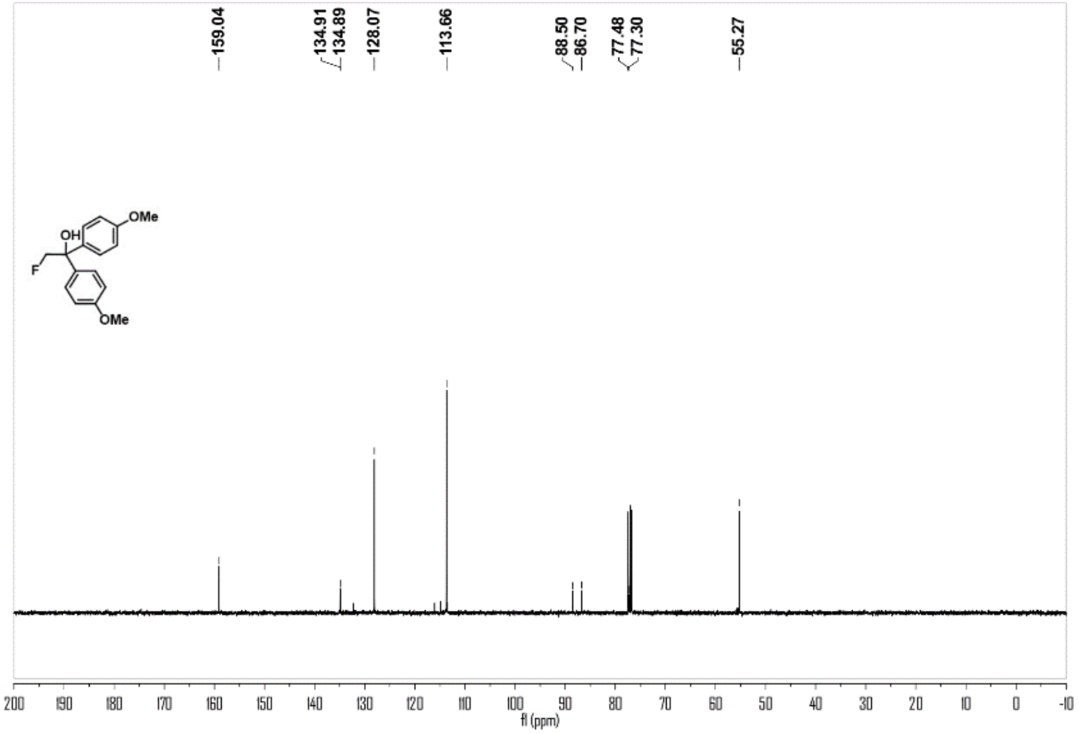

^{1}H, ^{19}F and ^{13}C NMR spectra of compound 1n

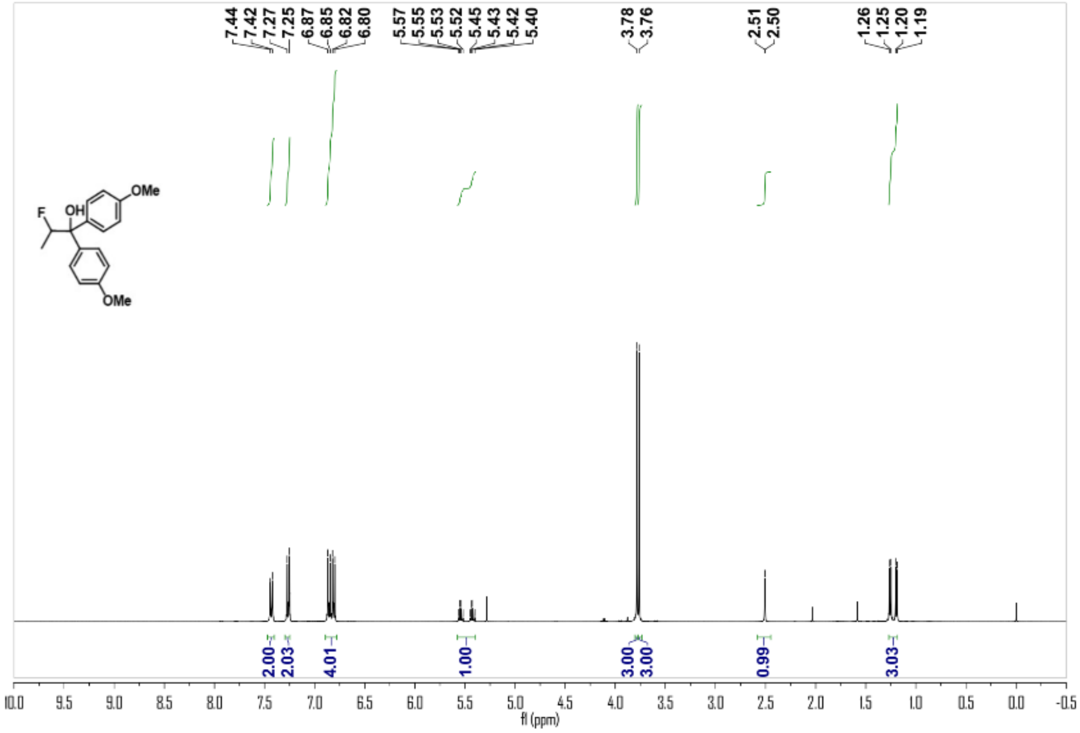

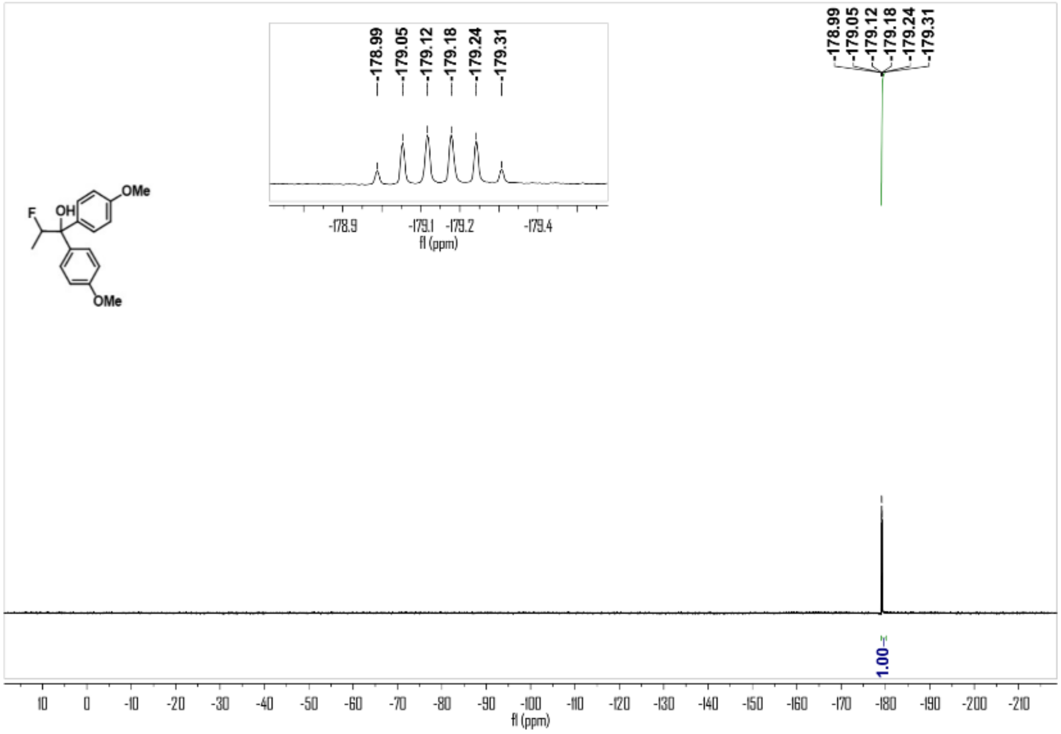
-178.99
-179.05
-179.12
-179.18
-179.24
-179.31
-178.9
-179.1
-179.2
-179.4
fl (ppm)
OMe
F
OH
OMe
1.00
10 0 -10 -20 -30 -40 -50 -60 -70 -80 -90 -100 -110 -120 -130 -140 -150 -160 -170 -180 -190 -200 -210
fl (ppm)

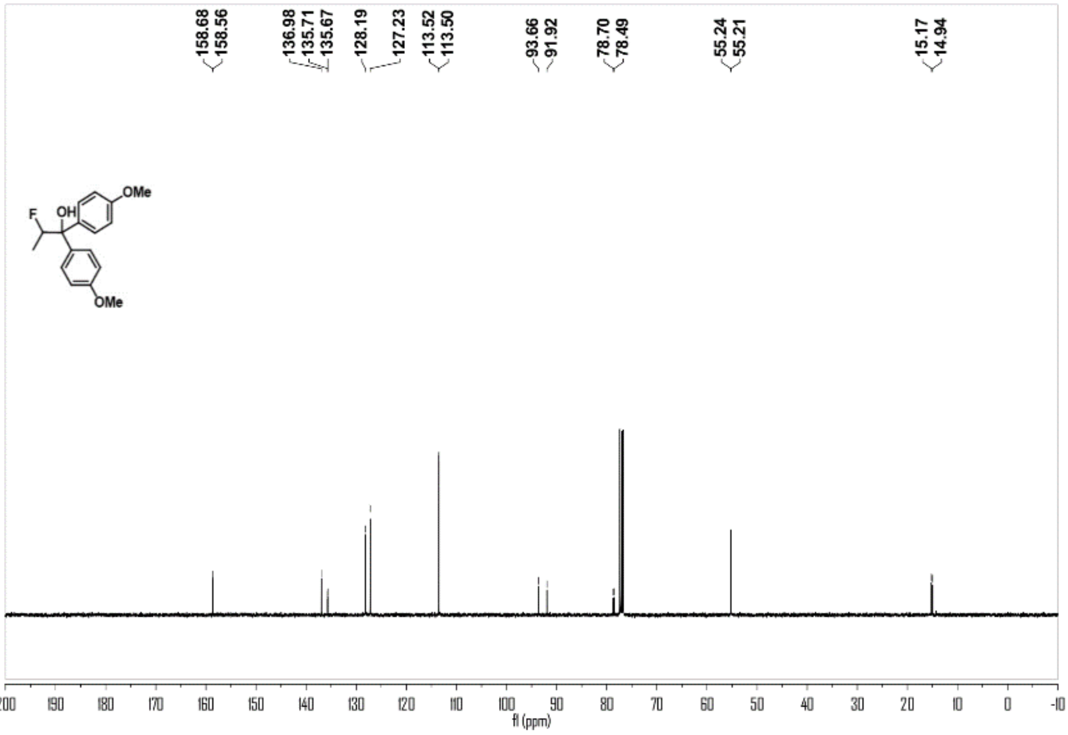
158.68
158.56
136.98
135.71
135.67
128.19
127.23
113.52
113.50
93.66
91.92
78.70
78.49
55.24
55.21
15.17
14.94
OMe
F
OH
OMe
200 190 180 170 160 150 140 130 120 110 100 90 80 70 60 50 40 30 20 10 0 -10
fl (ppm)

^{1}H, ^{19}F and ^{13}C NMR spectra of compound 1o

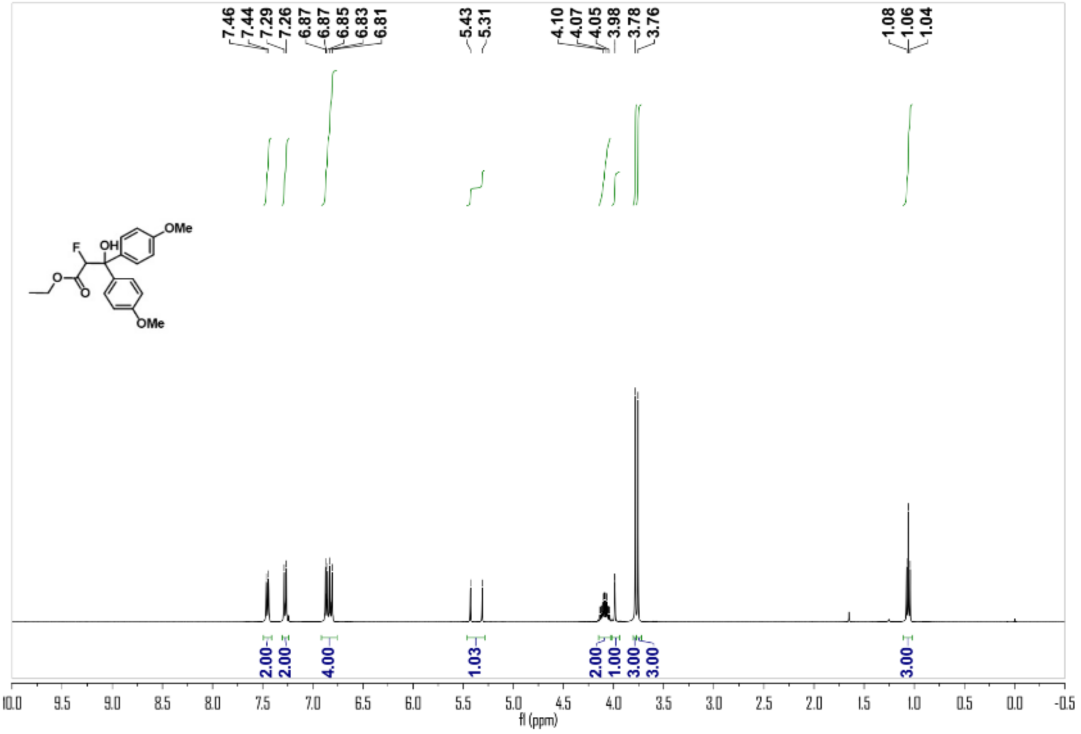

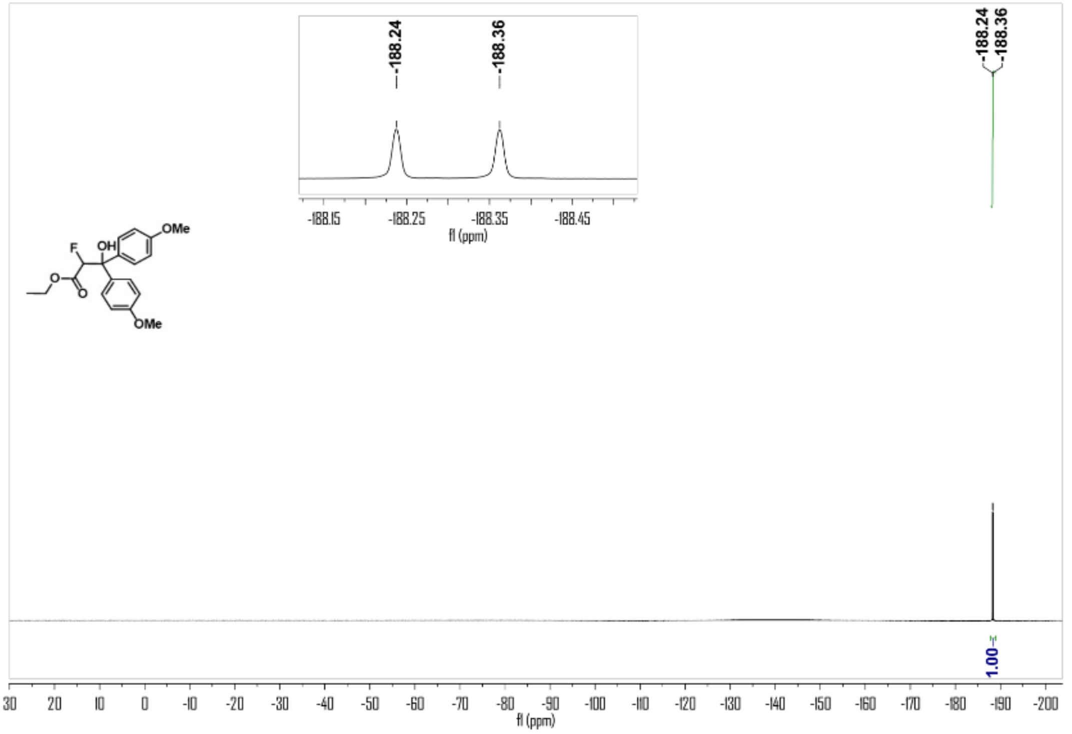

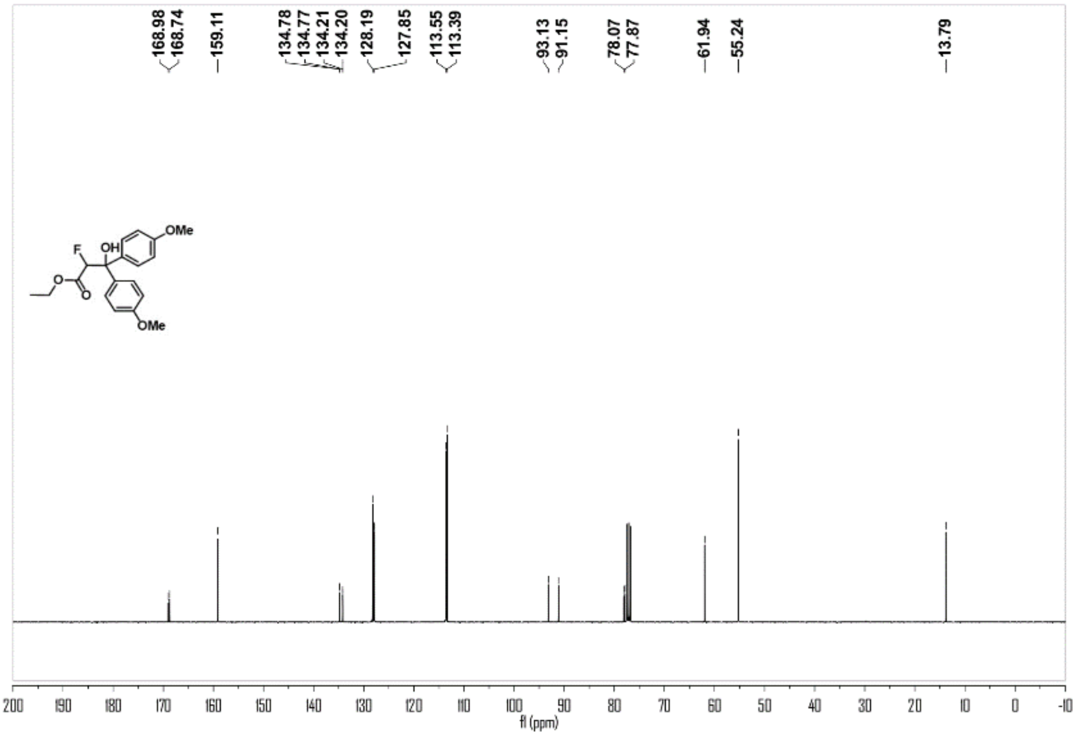

^{1}H, ^{19}F and ^{13}C NMR spectra of compound 1p

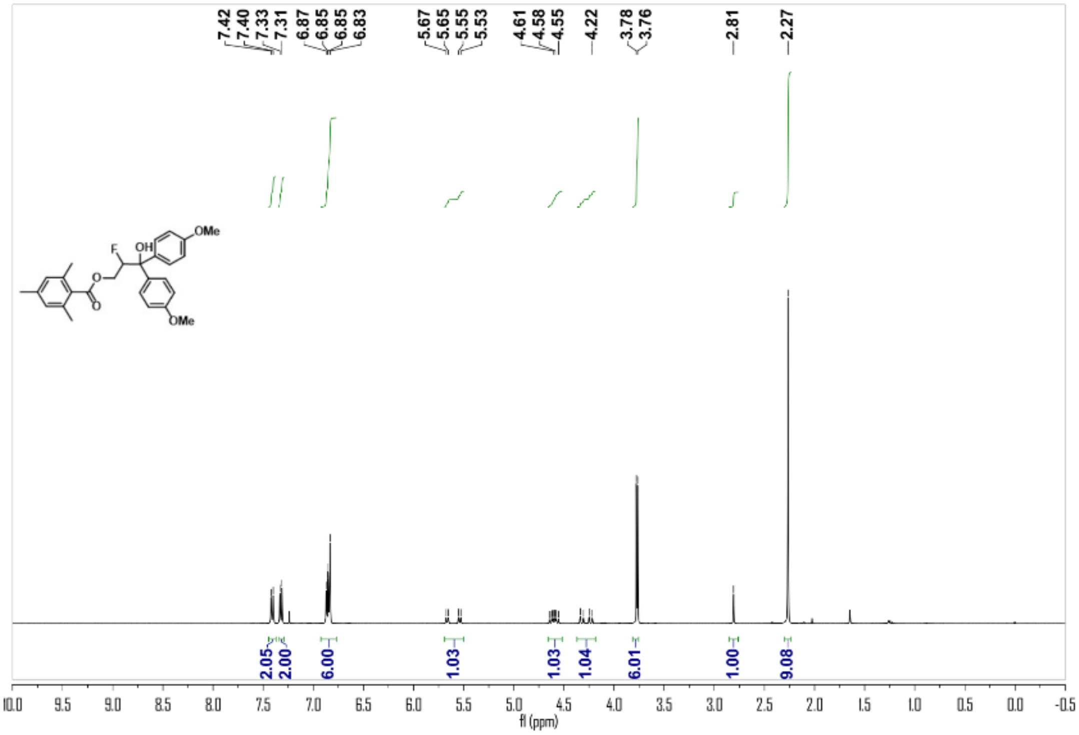

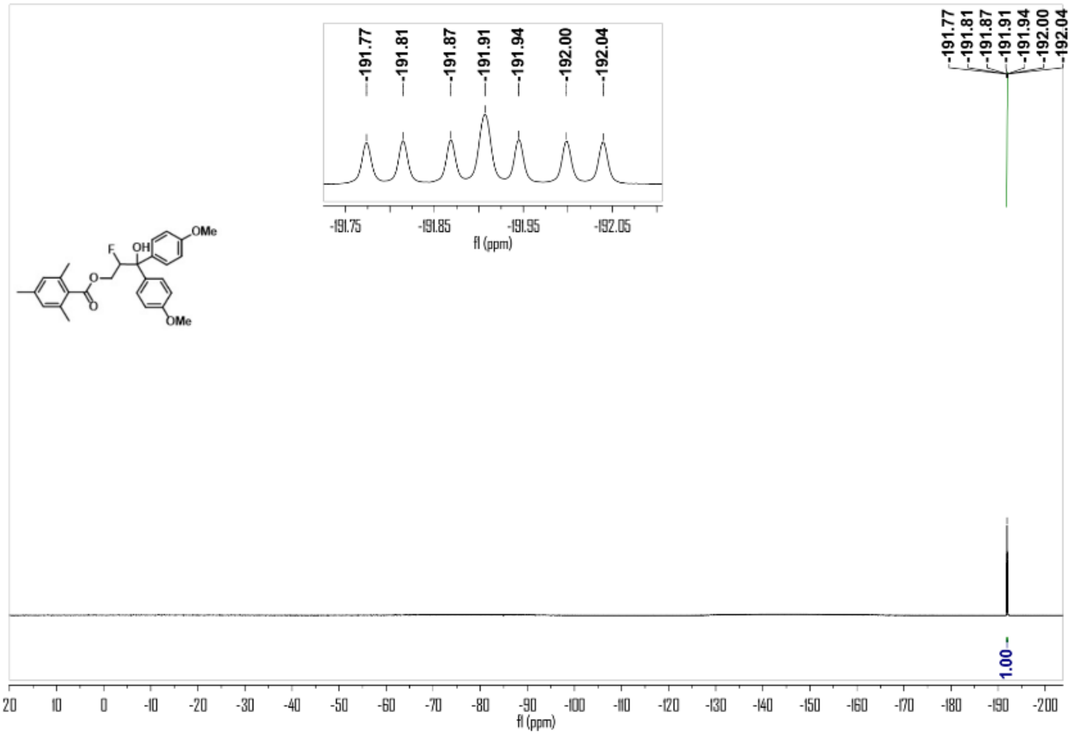

-191.77
-191.81
-191.87
-191.91
-191.94
-192.00
-192.04
-191.75
-191.85
-191.95
-192.05
f1 (ppm)
OMe
F
OH
O
O
OMe
1.00
20
10
0
-10
-20
-30
-40
-50
-60
-70
-80
-90
-100
-110
-120
-130
-140
-150
-160
-170
-180
-190
-200
f1 (ppm)

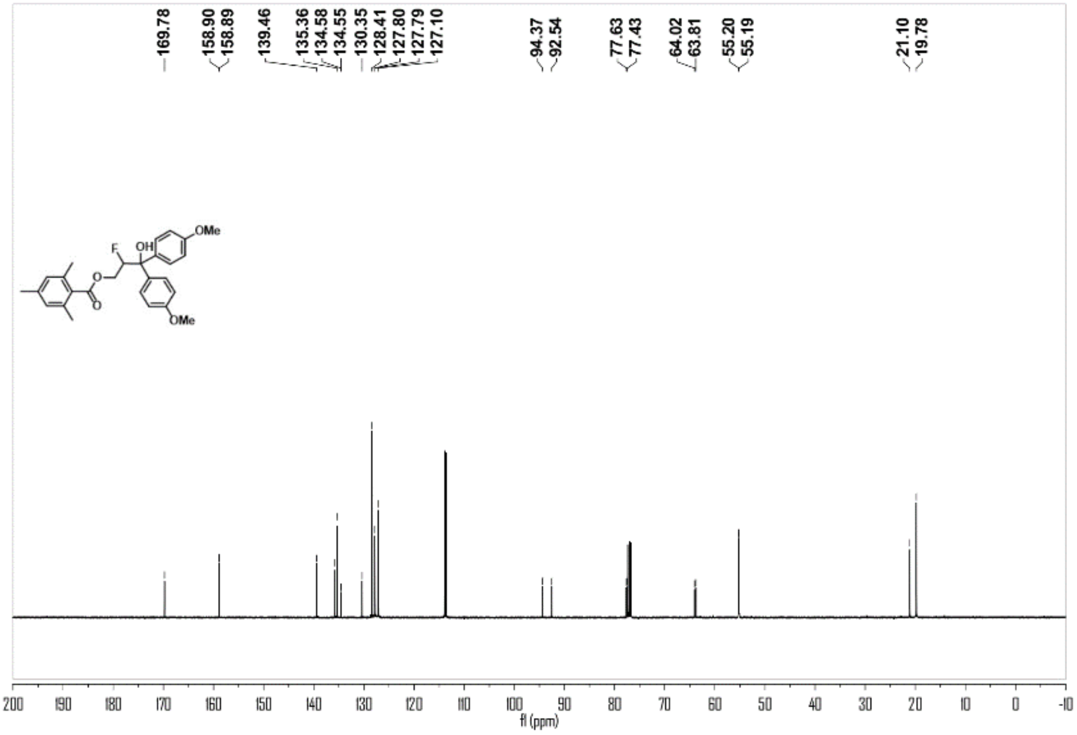

169.78
158.90
158.89
139.46
135.36
134.58
134.55
130.35
128.41
127.80
127.79
127.10
94.37
92.54
77.63
77.43
64.02
63.81
55.20
55.19
21.10
19.78
OMe
F
OH
O
O
OMe
200
190
180
170
160
150
140
130
120
110
100
90
80
70
60
50
40
30
20
10
0
-10
f1 (ppm)

^{1}H, ^{19}F and ^{13}C NMR spectra of compound 1q

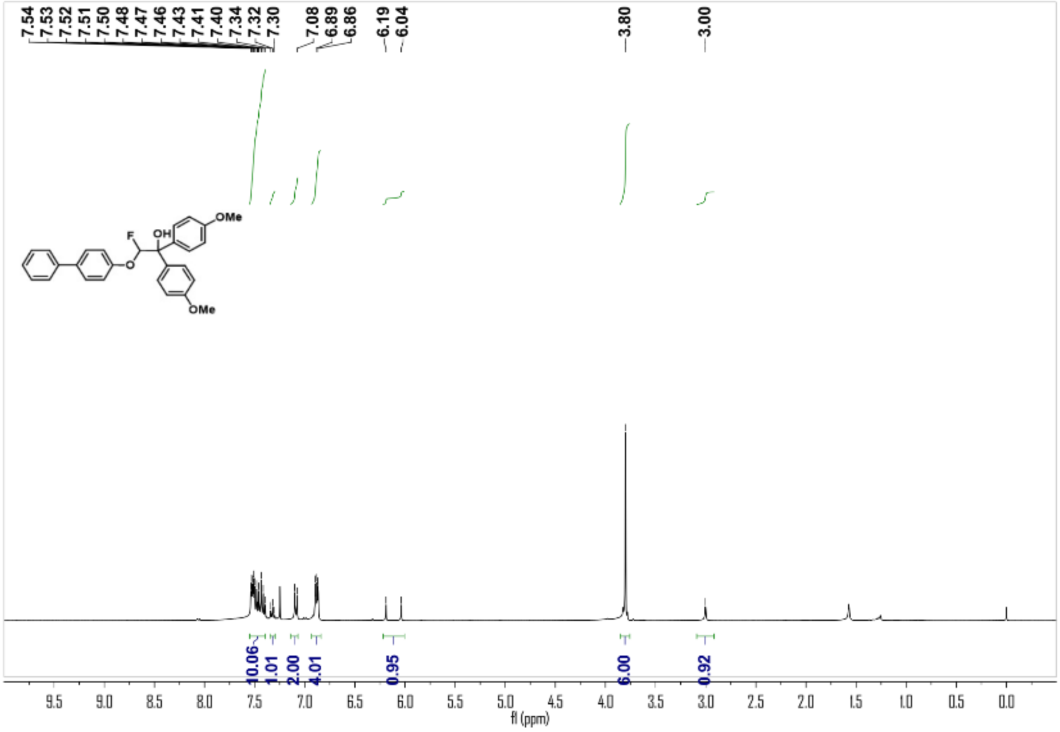

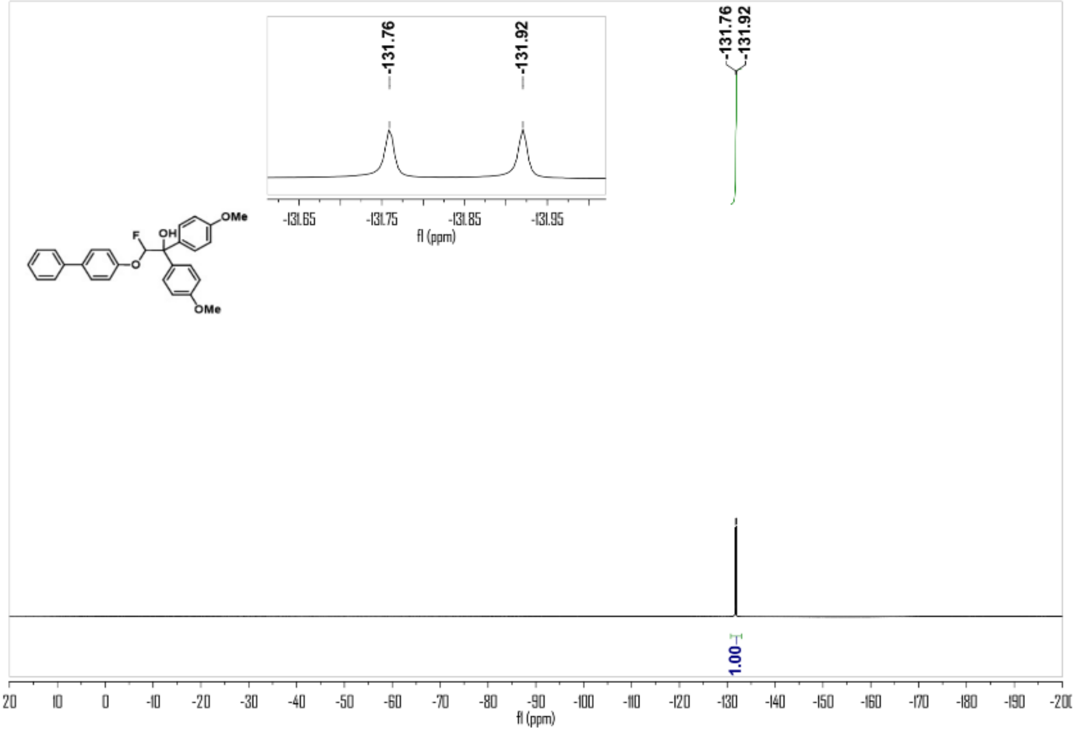

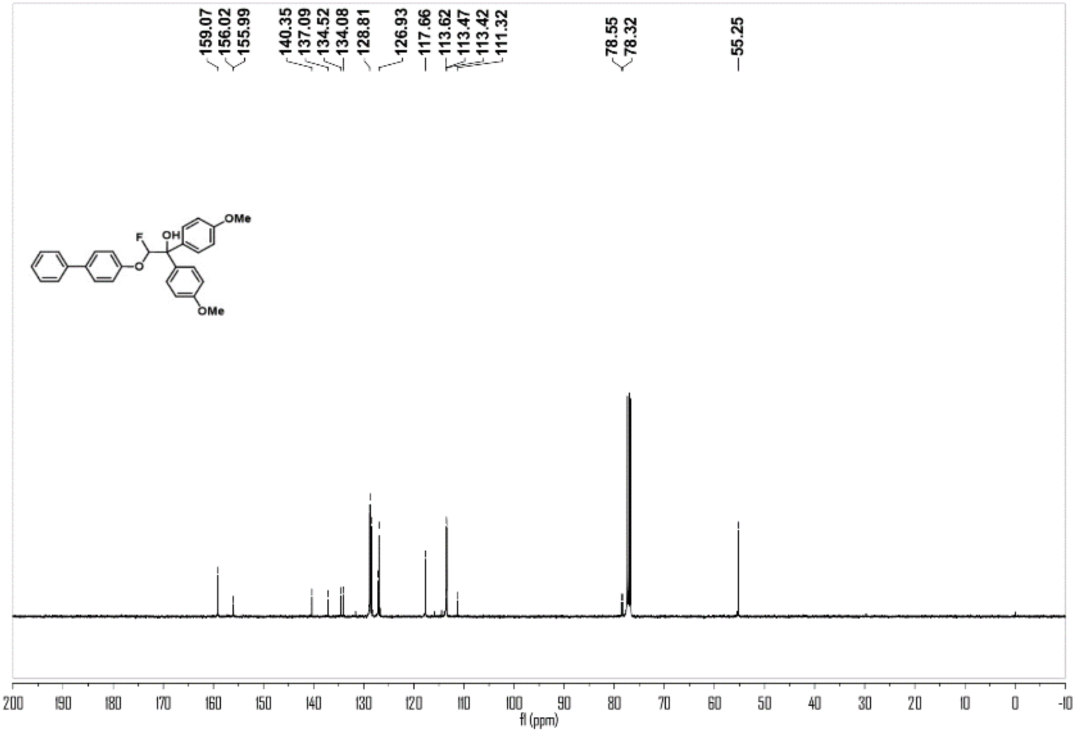

^{1}H, ^{19}F and ^{13}C NMR spectra of compound 1r

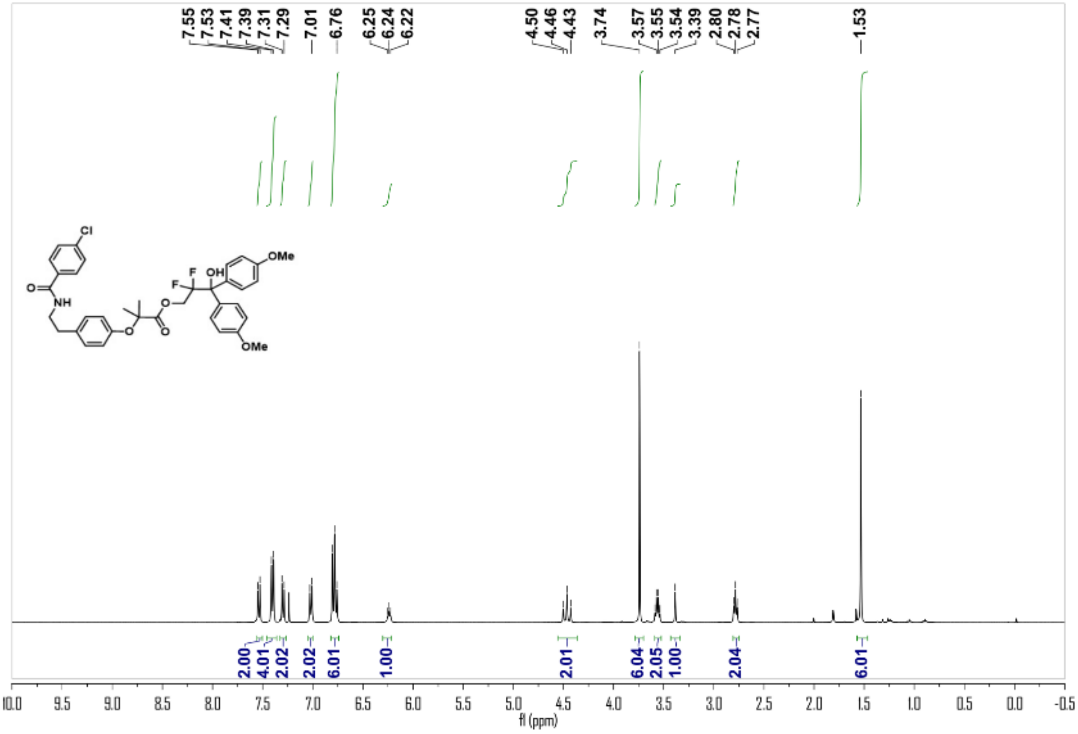

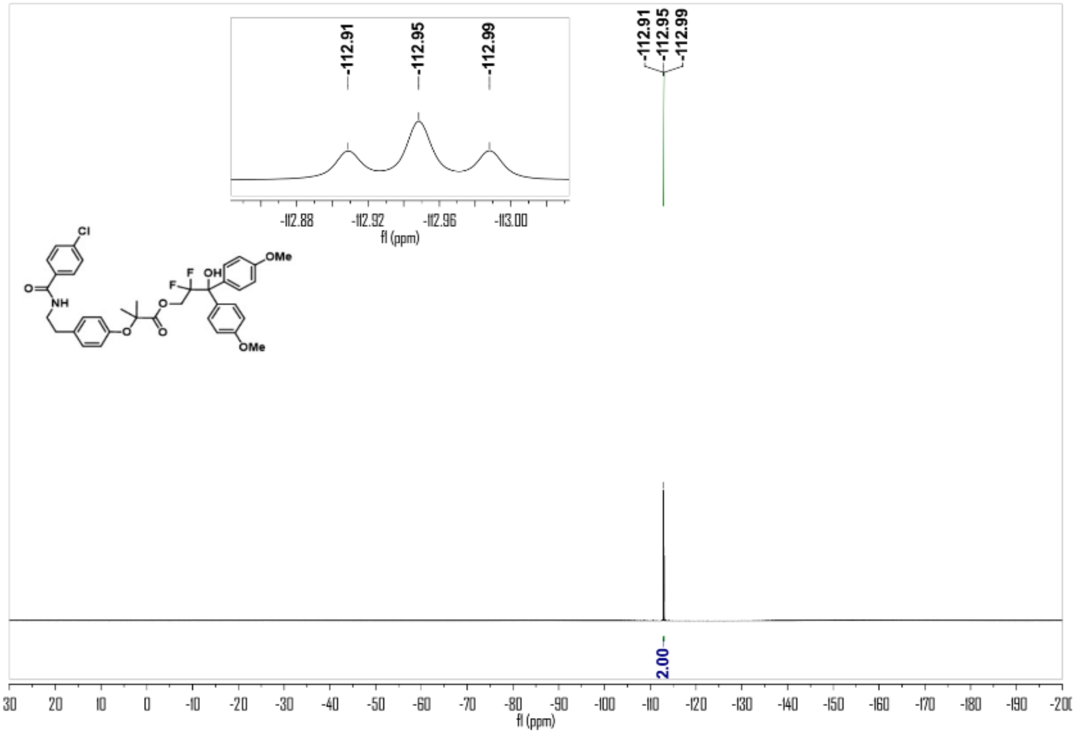
-112.91
-112.95
-112.99
-112.88
-112.92
-112.96
-113.00
fl (ppm)
Cl
O
NH
F
F
OH
OMe
O
O
O
OMe
2.00
30 20 10 0 -10 -20 -30 -40 -50 -60 -70 -80 -90 -100 -110 -120 -130 -140 -150 -160 -170 -180 -190 -200
fl (ppm)

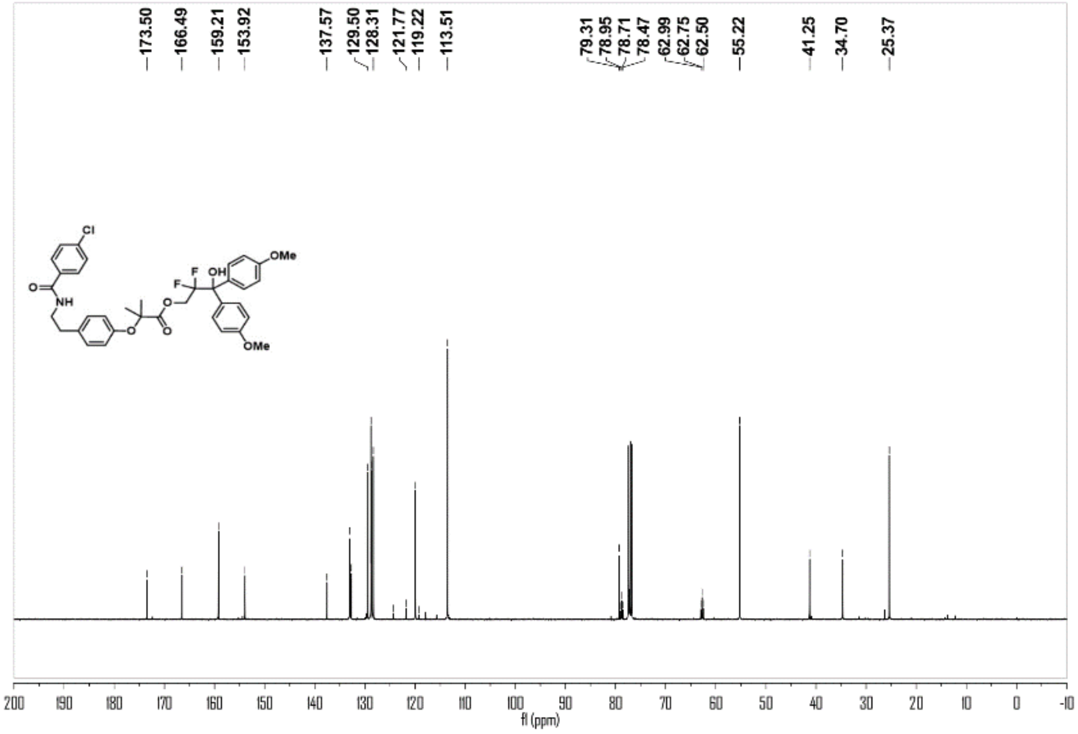
173.50
166.49
159.21
153.92
137.57
129.50
128.31
121.77
119.22
113.51
79.31
78.95
78.71
78.47
62.99
62.75
62.50
55.22
41.25
34.70
25.37
Cl
O
NH
F
F
OH
OMe
O
O
O
OMe
200 190 180 170 160 150 140 130 120 110 100 90 80 70 60 50 40 30 20 10 0 -10
fl (ppm)

^{1}H, and ^{19}F NMR spectra of compound 1ba-I

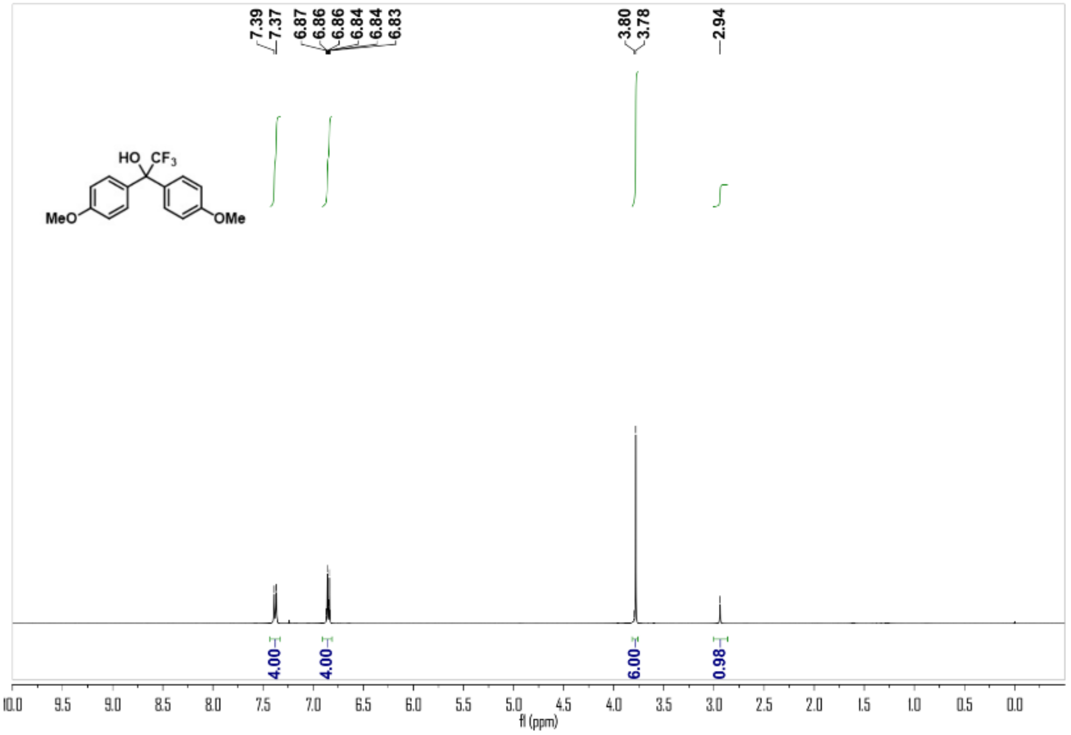

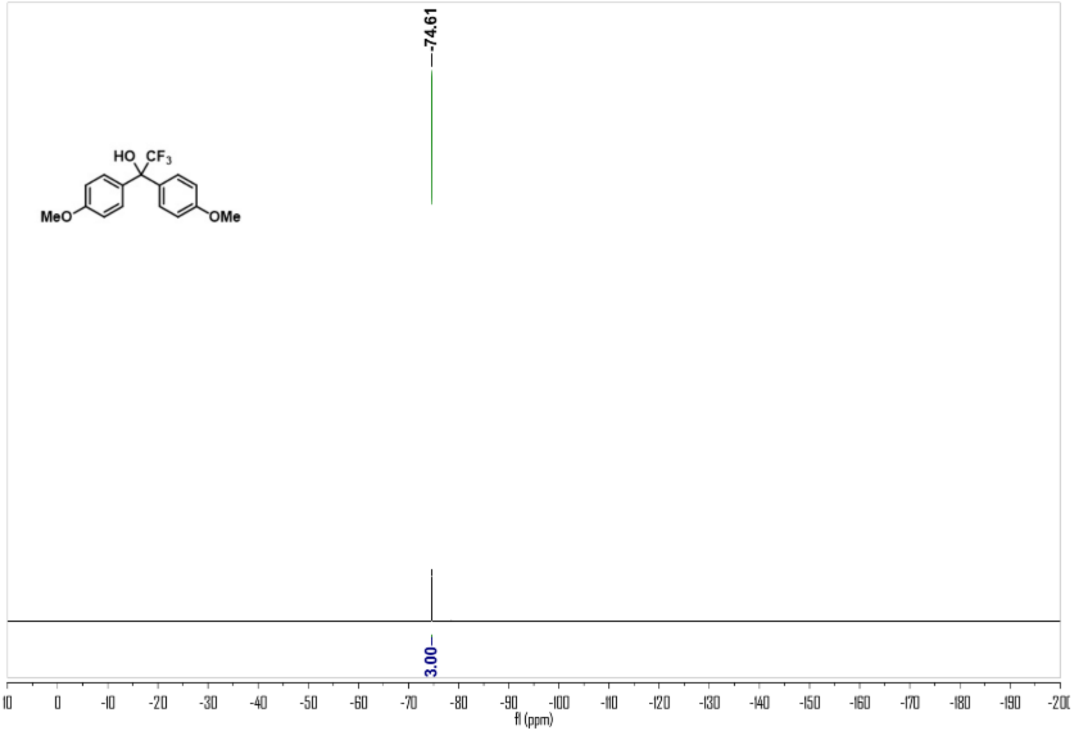

^{1}H, ^{19}F and ^{13}C NMR spectra of compound 1ba-II

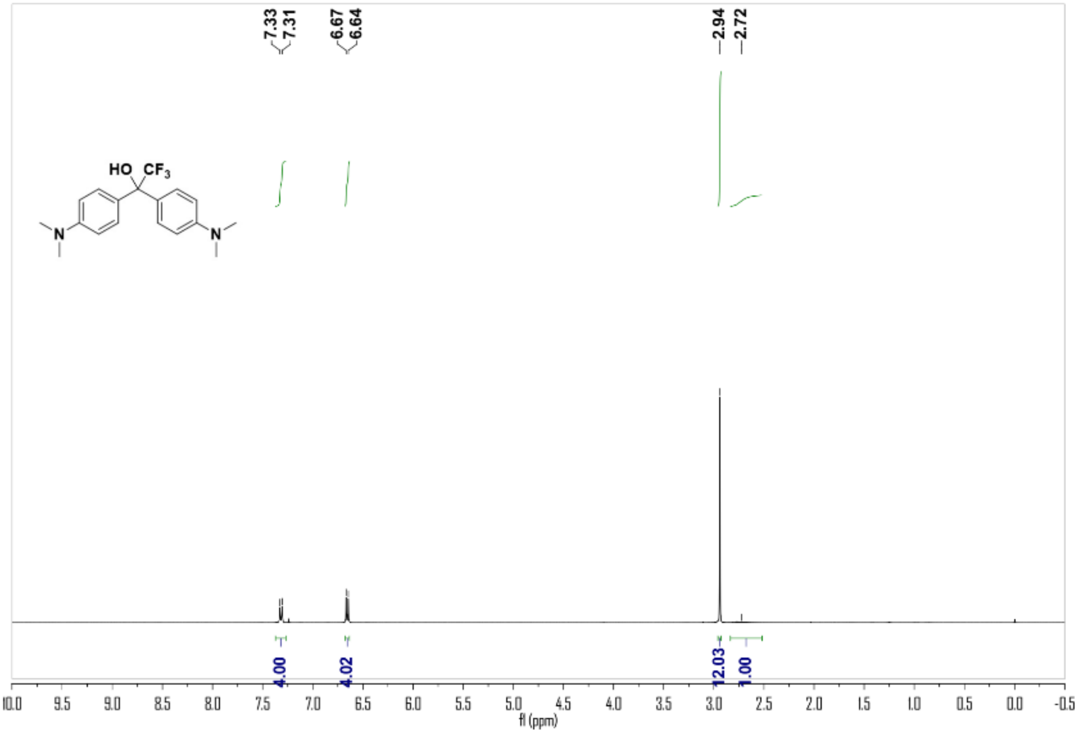

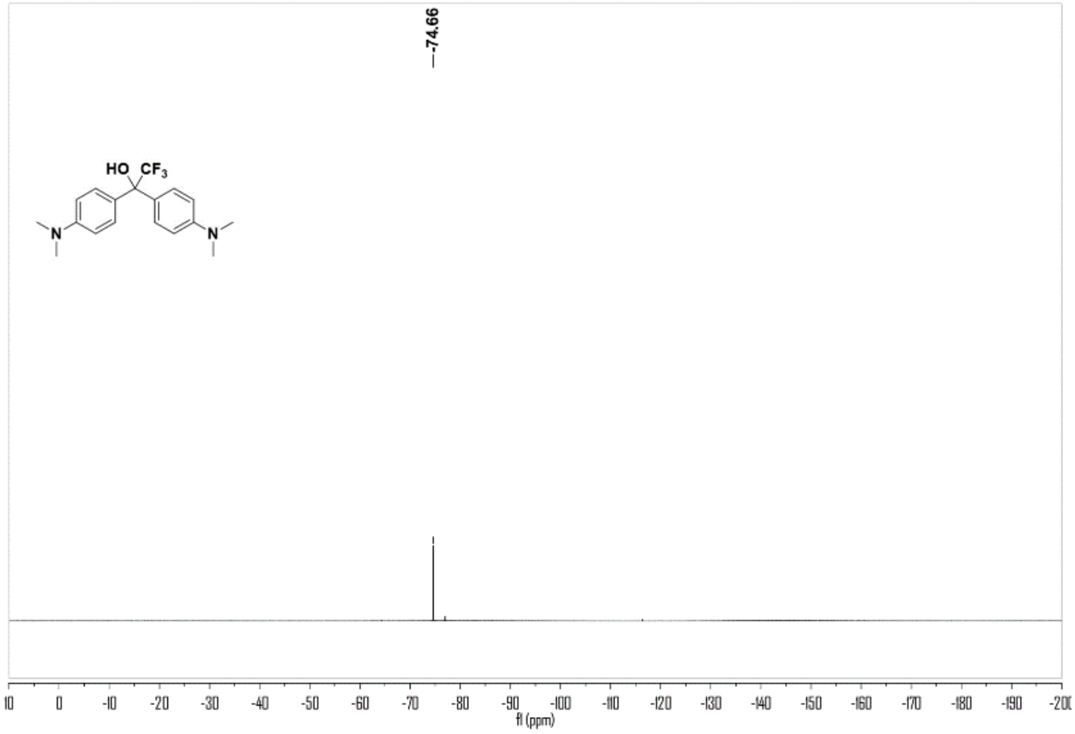

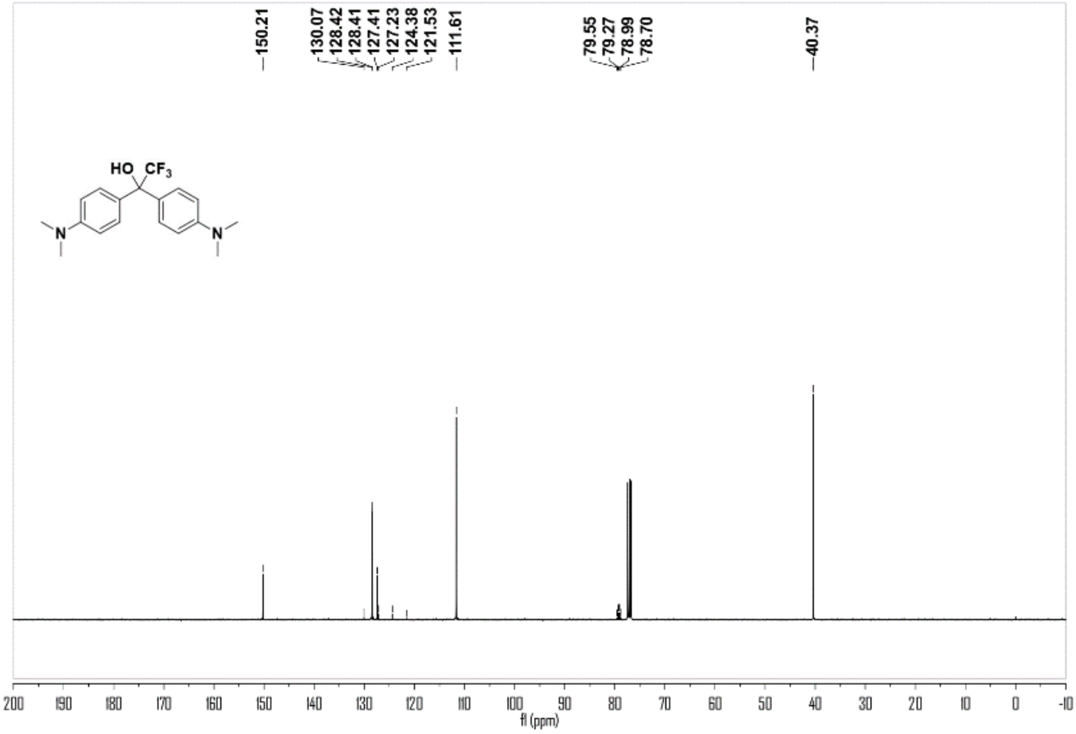

^{1}H, and ^{19}F NMR spectra of compound 1ba-III

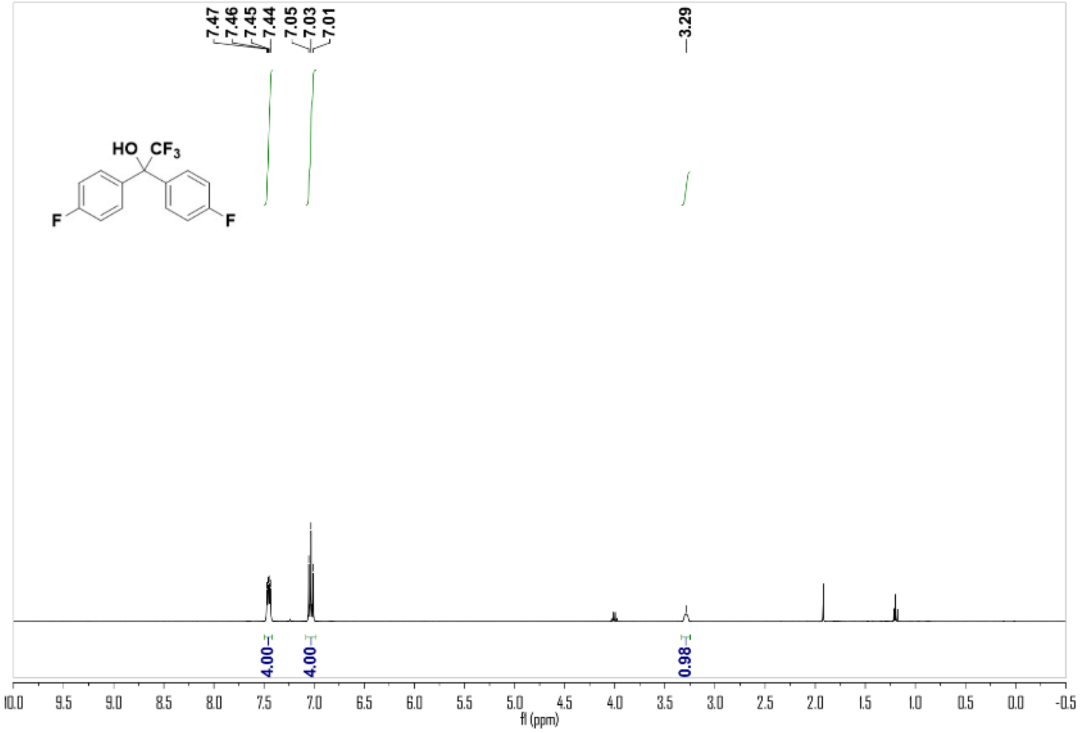

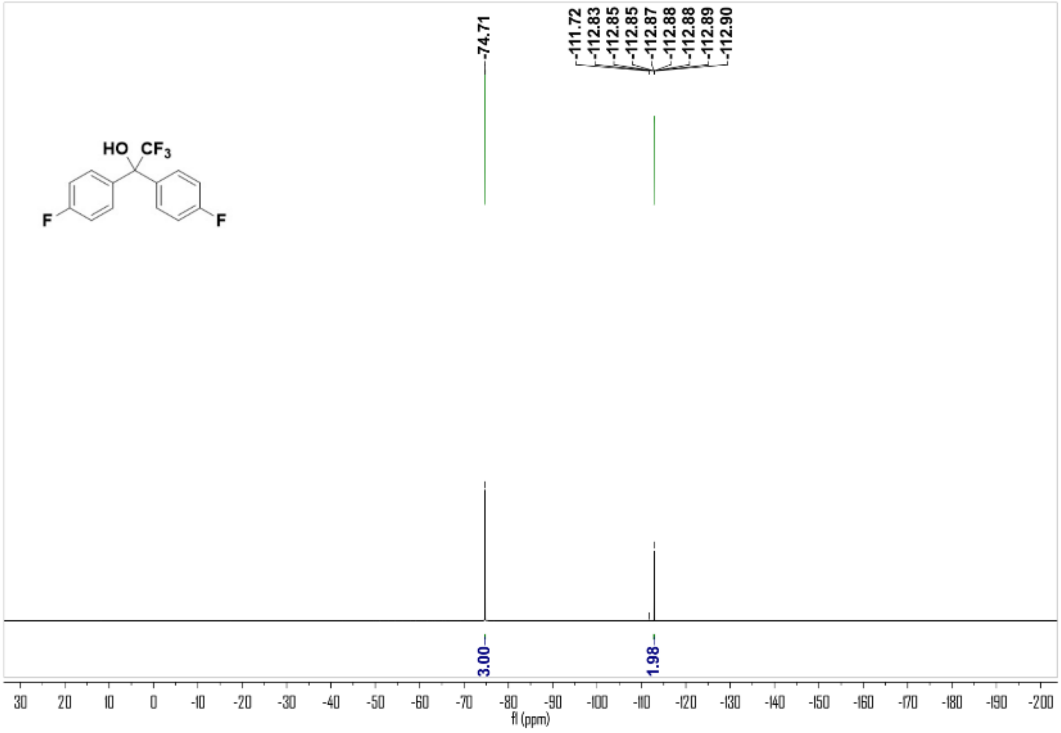

^{1}H, and ^{19}F NMR spectra of compound 1ba-IV

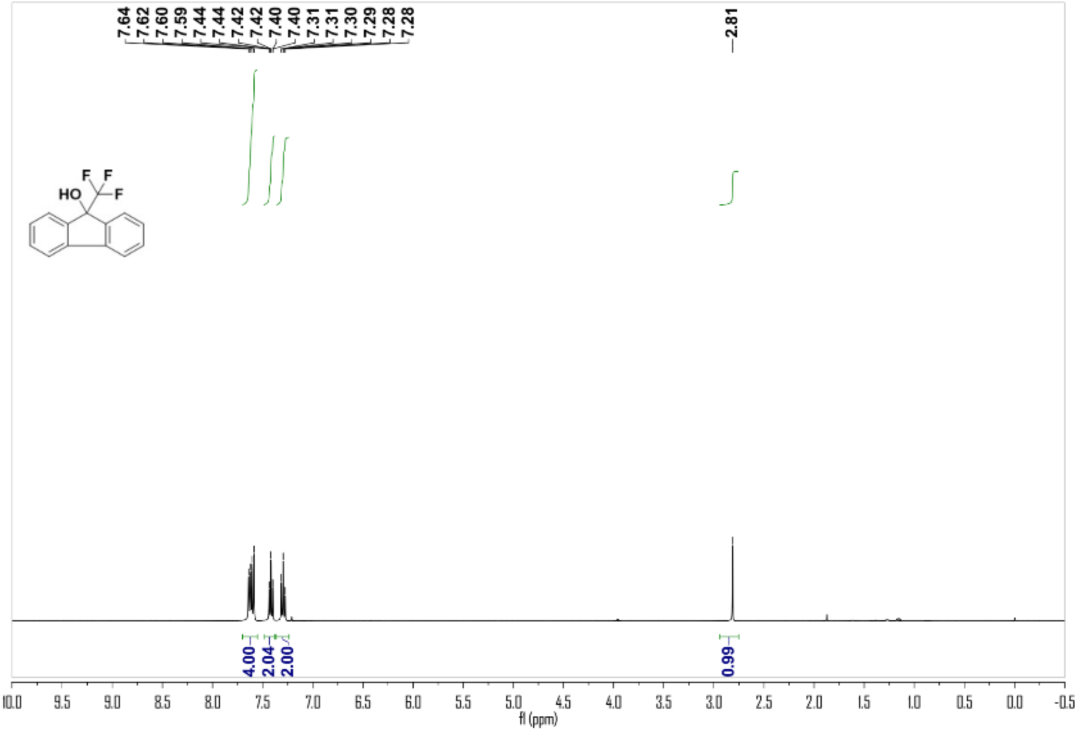

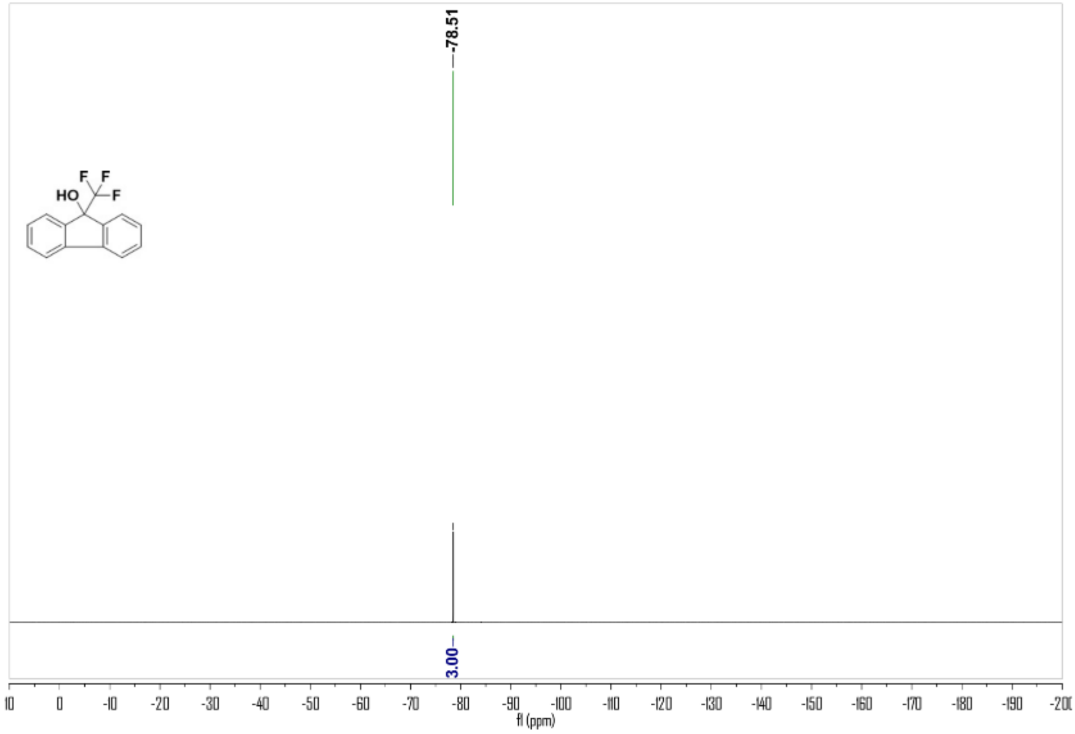

^{1}H, ^{19}F and ^{13}C NMR spectra of compound 1ba-V

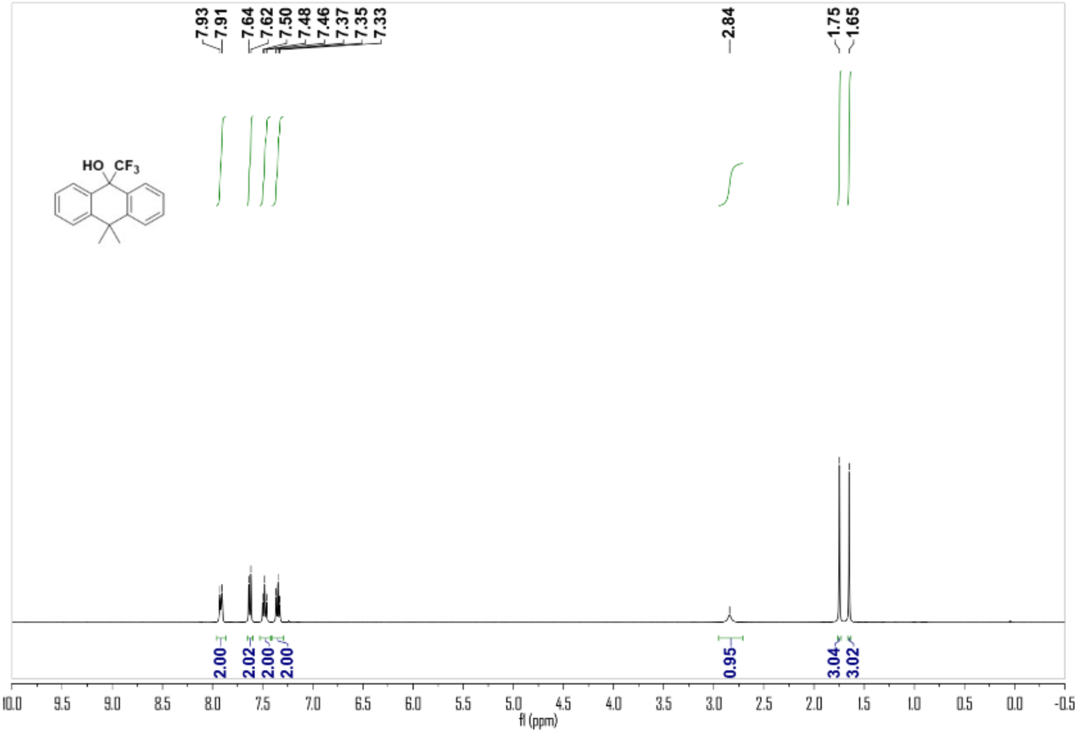

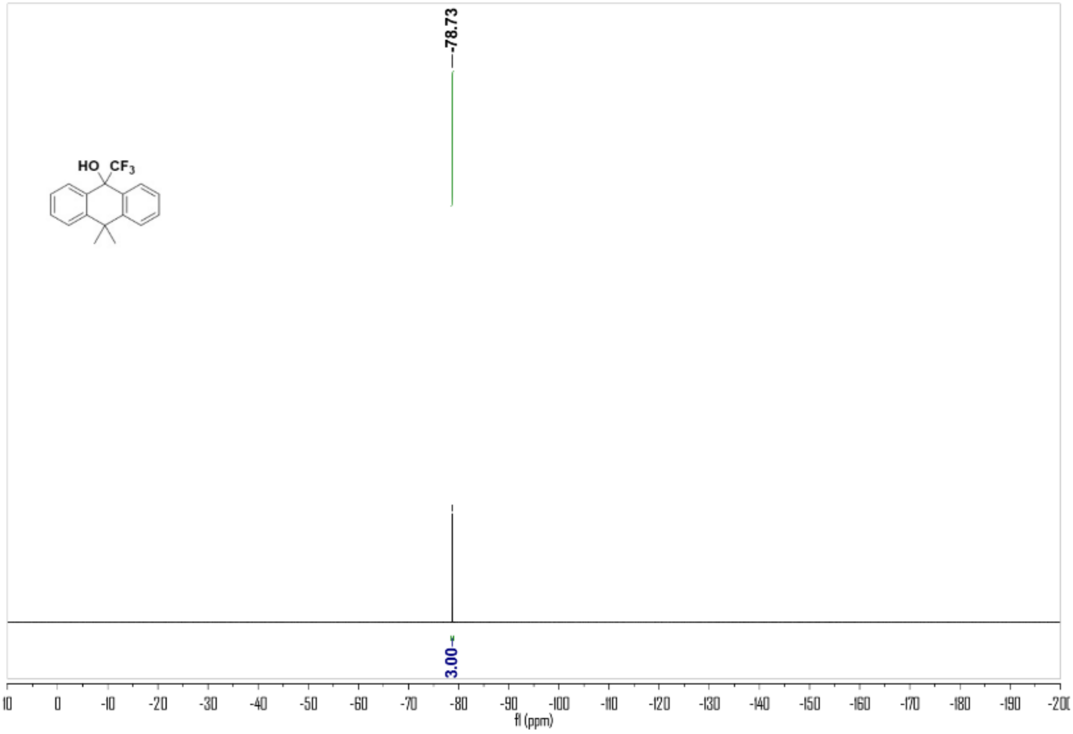

-78.73
HO CF3
3.00
10 0 -10 -20 -30 -40 -50 -60 -70 -80 -90 -100 -110 -120 -130 -140 -150 -160 -170 -180 -190 -200
f1 (ppm)

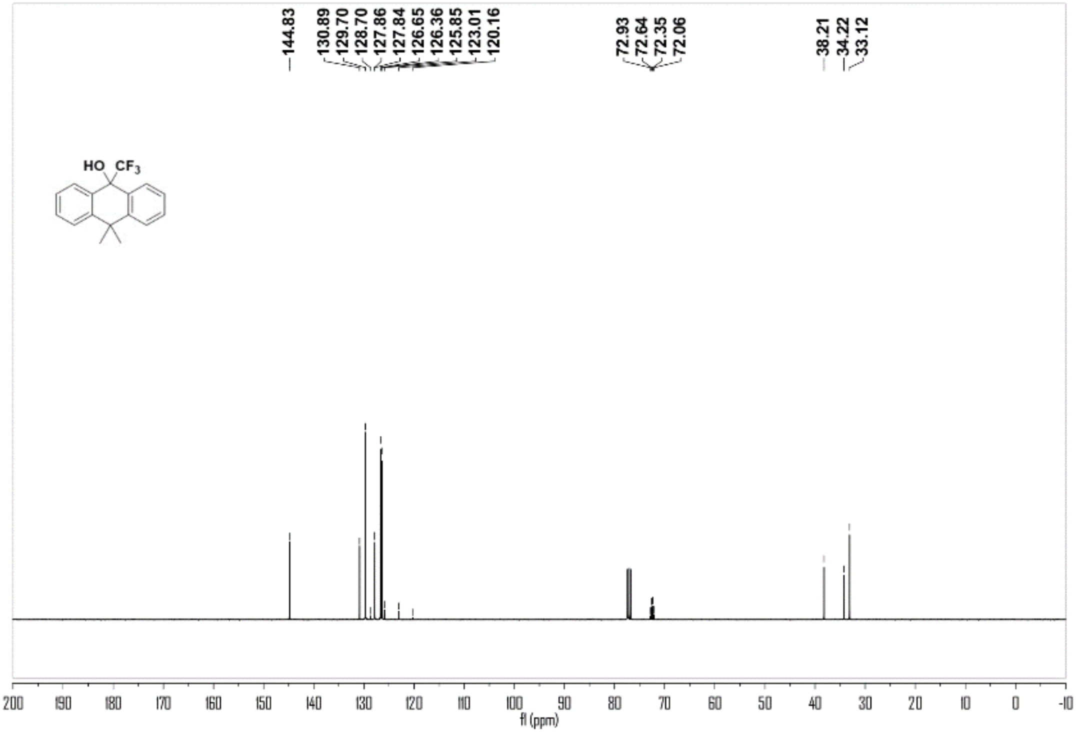

144.83
130.89
129.70
128.70
127.86
127.84
126.65
126.36
125.85
123.01
120.16
72.93
72.64
72.35
72.06
38.21
34.22
33.12
HO CF3
200 190 180 170 160 150 140 130 120 110 100 90 80 70 60 50 40 30 20 10 0 -10
f1 (ppm)

^{1}H, and ^{19}F NMR spectra of compound 1ba-VI

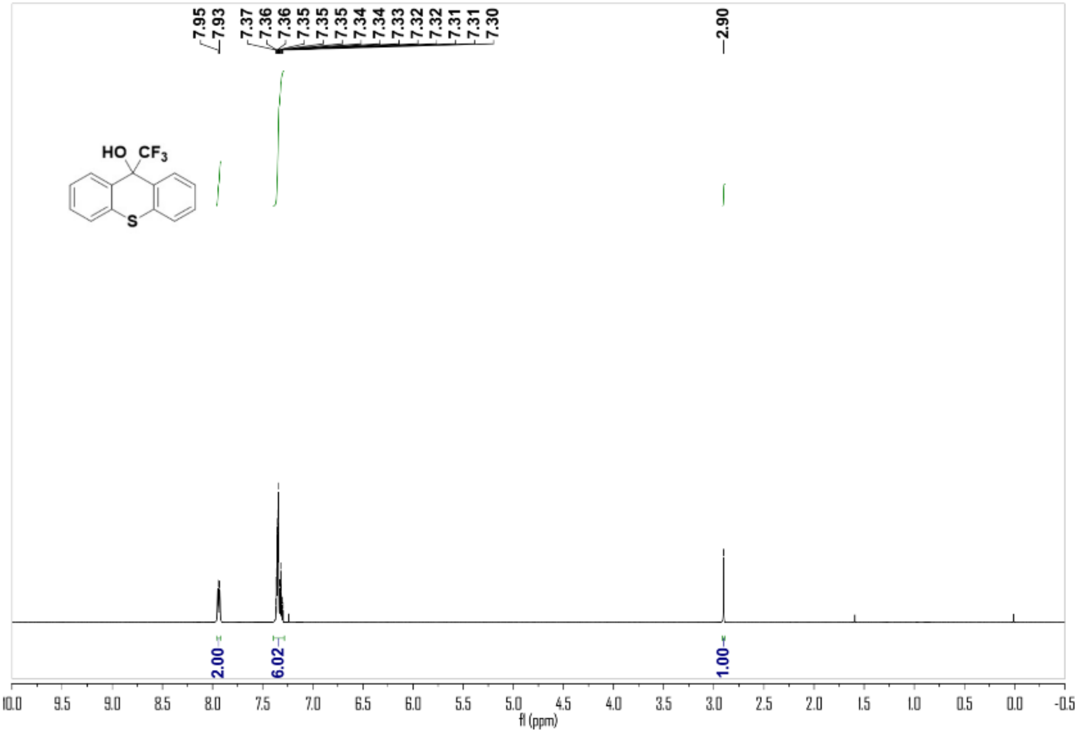

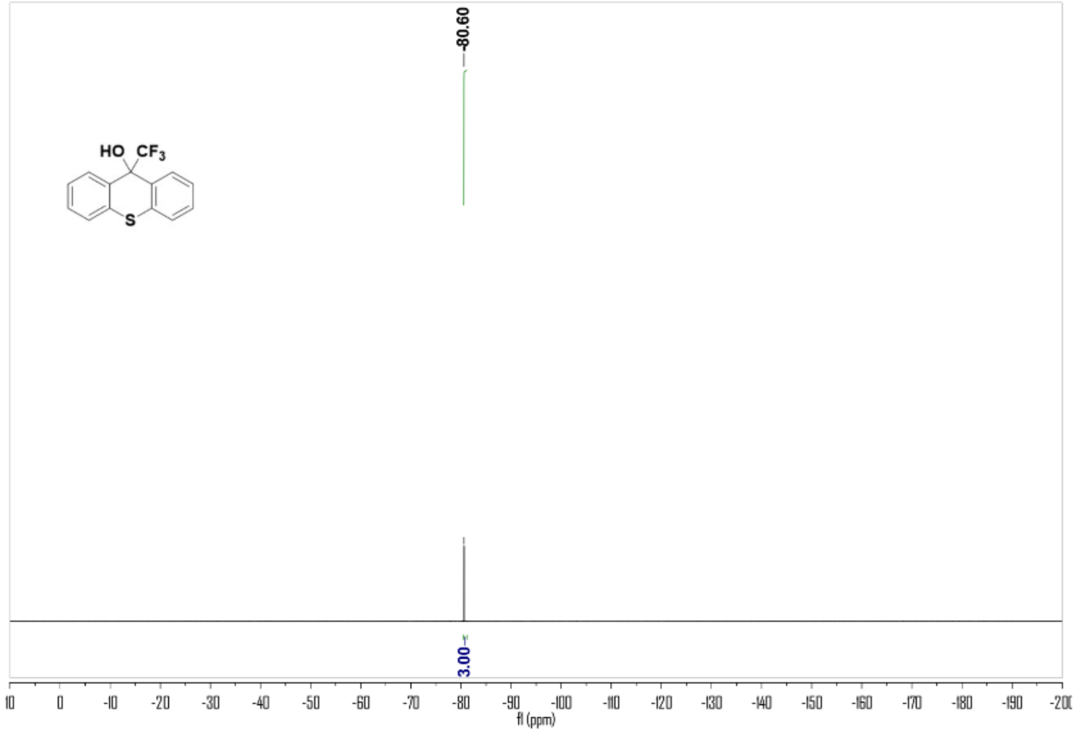

^{1}H, ^{19}F and ^{13}C NMR spectra of compound 1ba-VII

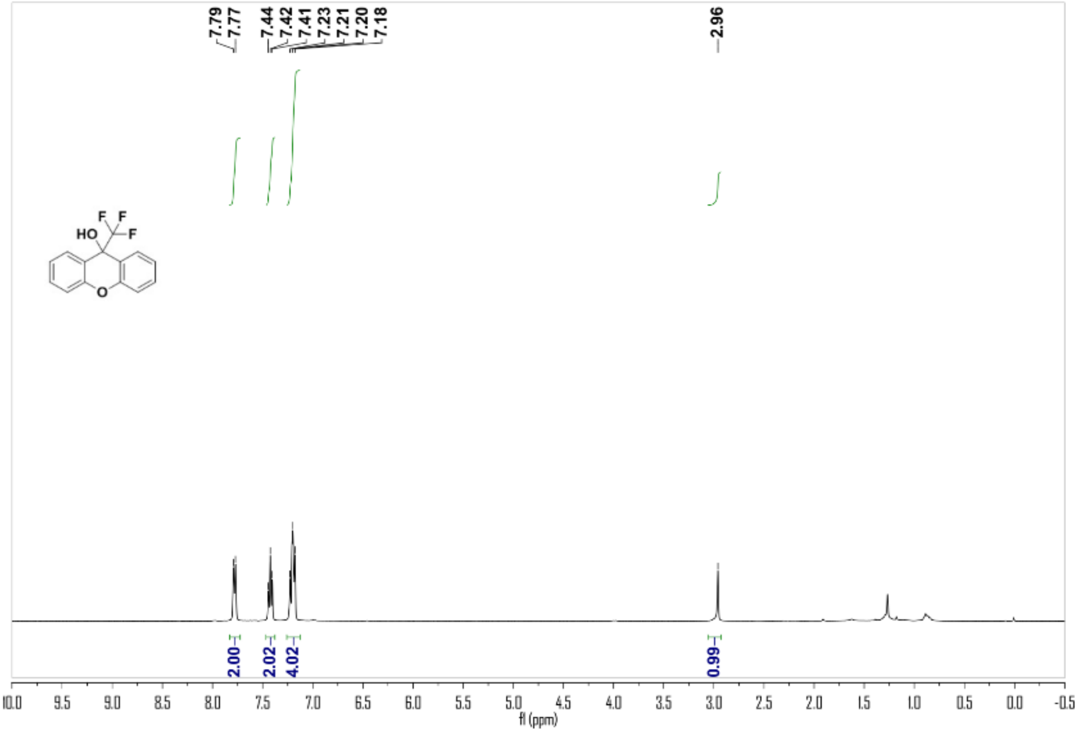

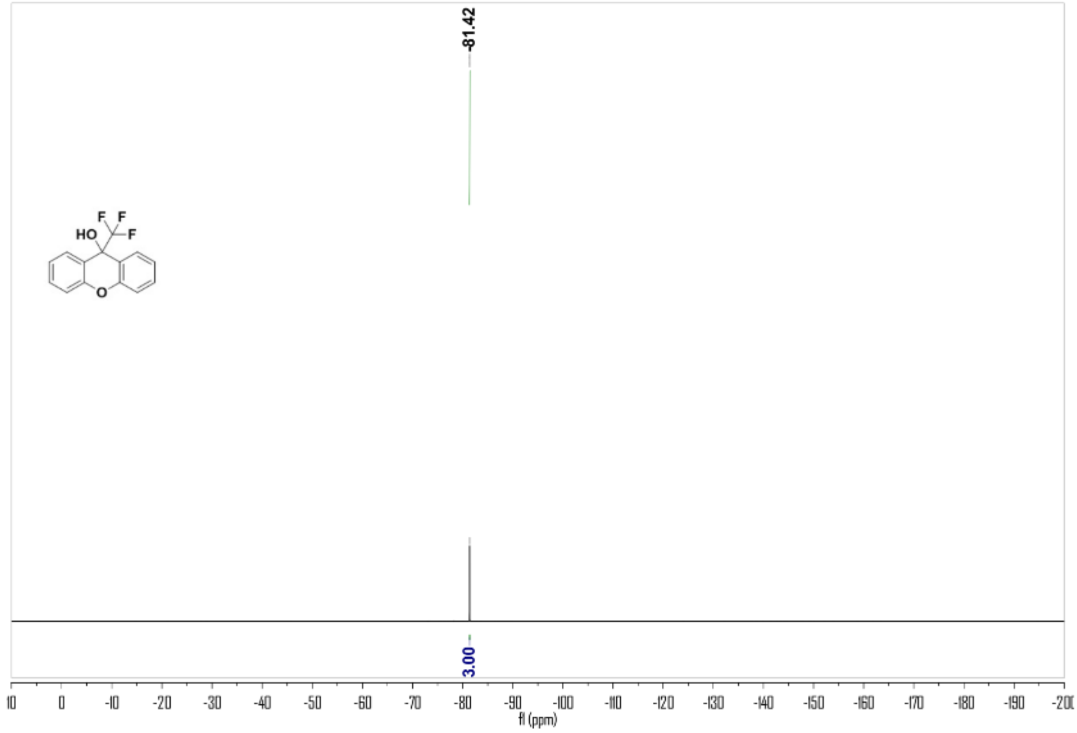

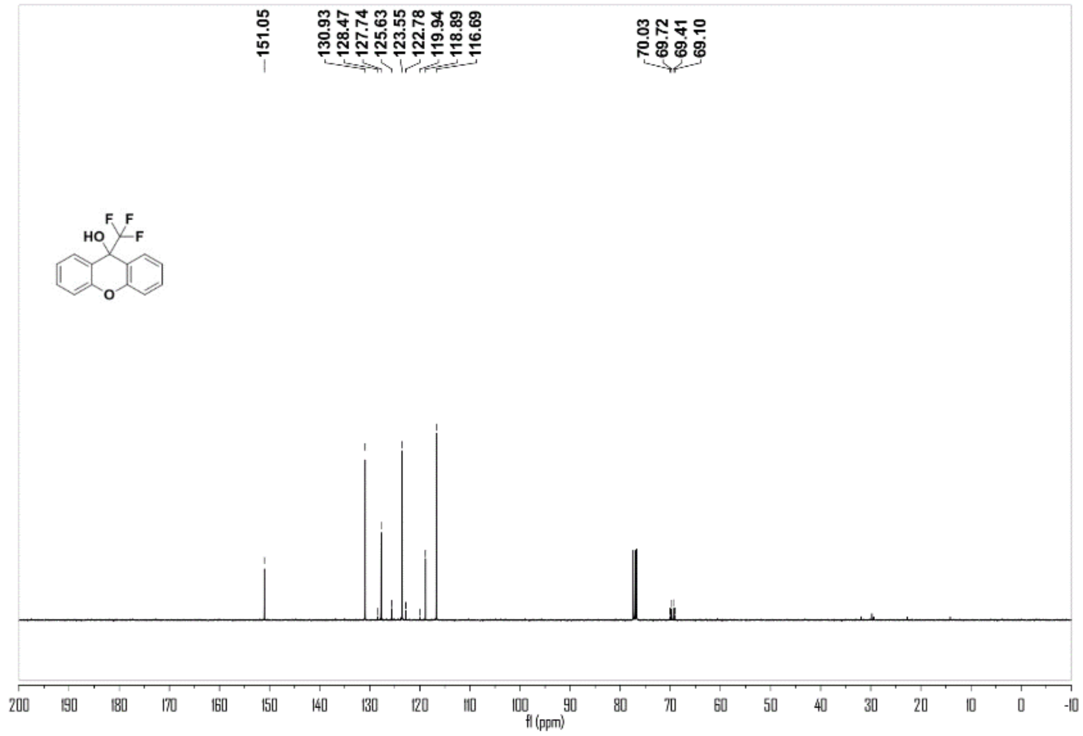

^{1}H, ^{19}F and ^{13}C NMR spectra of compound 1ba-VIII

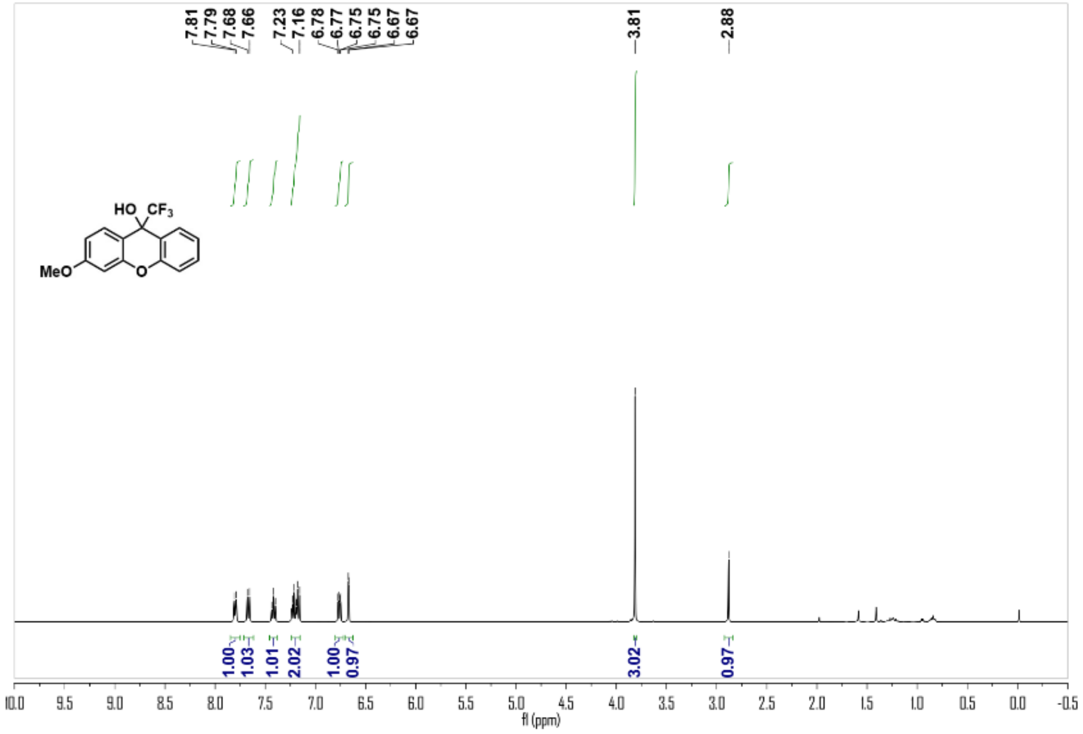

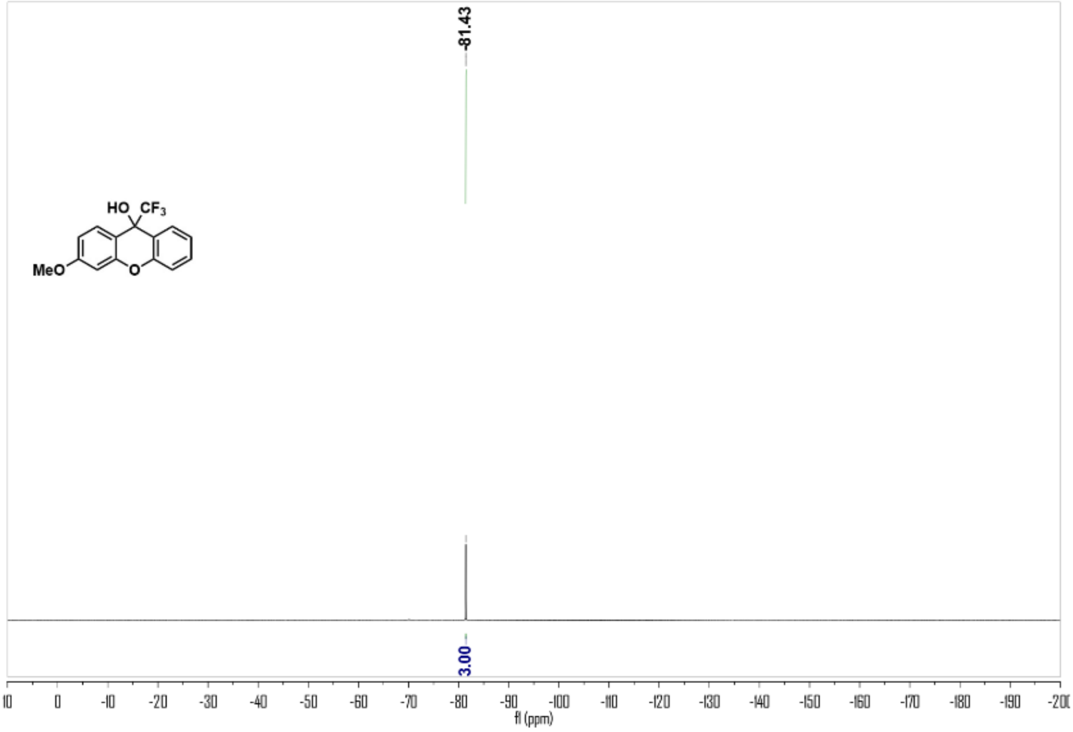
-81.43
HO CF3
MeO
O
3.00
10
0
-10
-20
-30
-40
-50
-60
-70
-80
-90
-100
-110
-120
-130
-140
-150
-160
-170
-180
-190
-200
f1 (ppm)

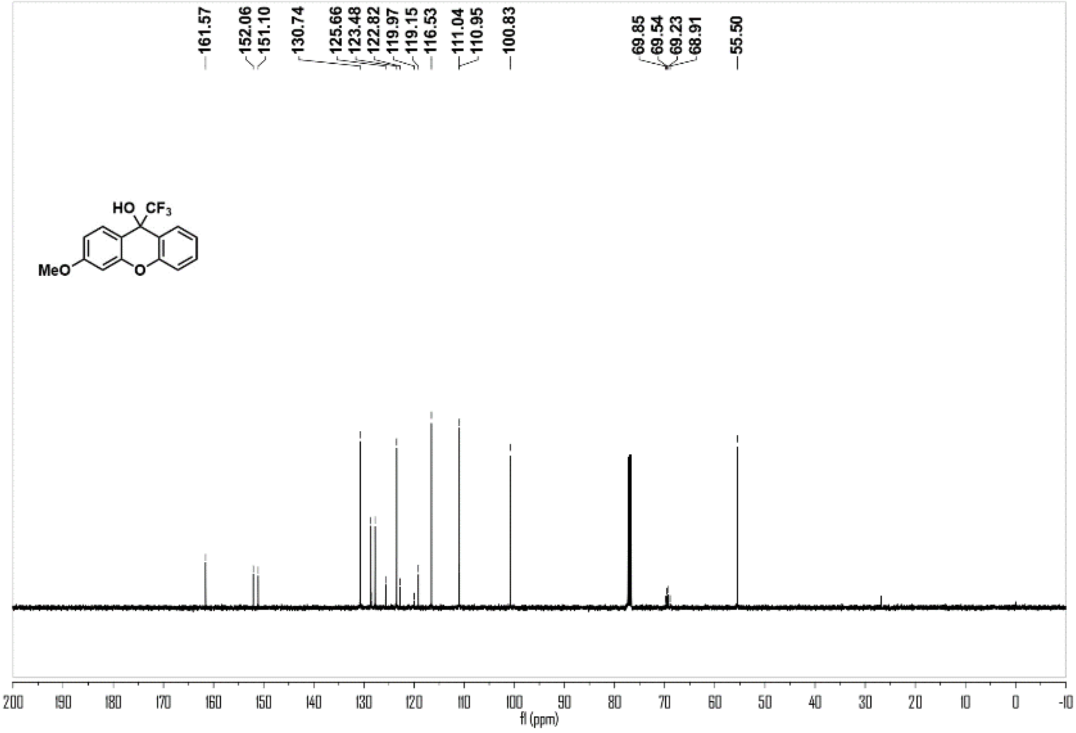
161.57
152.06
151.10
130.74
125.66
123.48
122.82
119.97
119.15
116.53
111.04
110.95
100.83
69.85
69.54
69.23
68.91
55.50
HO CF3
MeO
O
200
190
180
170
160
150
140
130
120
110
100
90
80
70
60
50
40
30
20
10
0
-10
f1 (ppm)

^{1}H, ^{19}F and ^{13}C NMR spectra of compound 1ba-IX

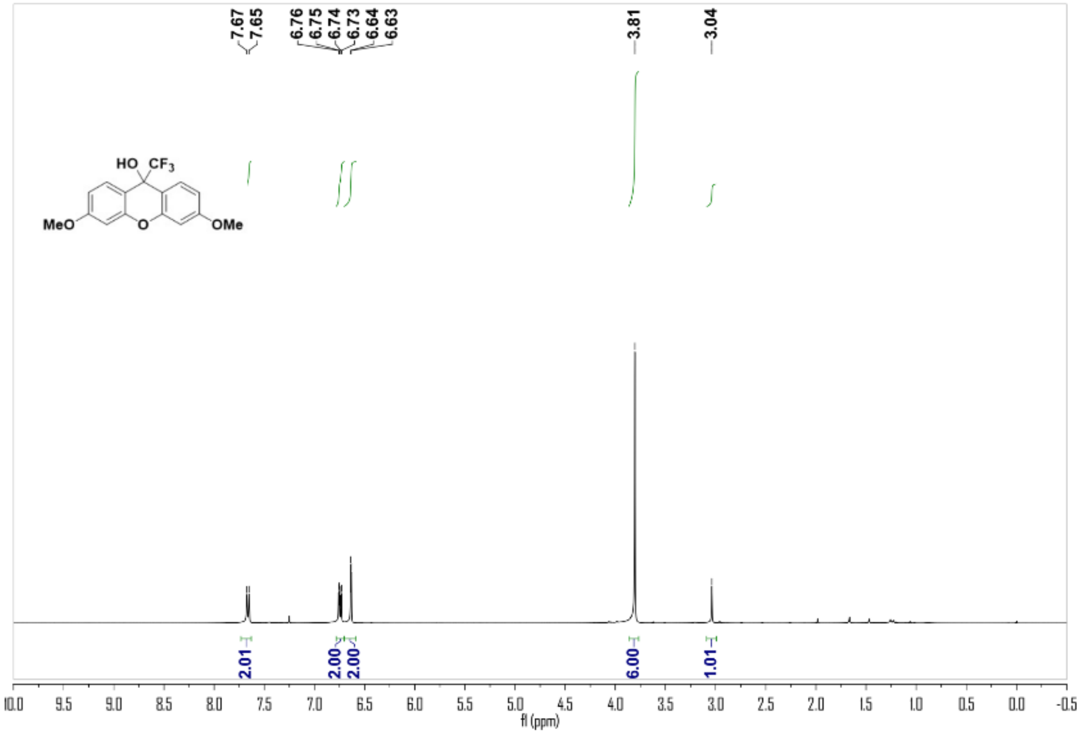

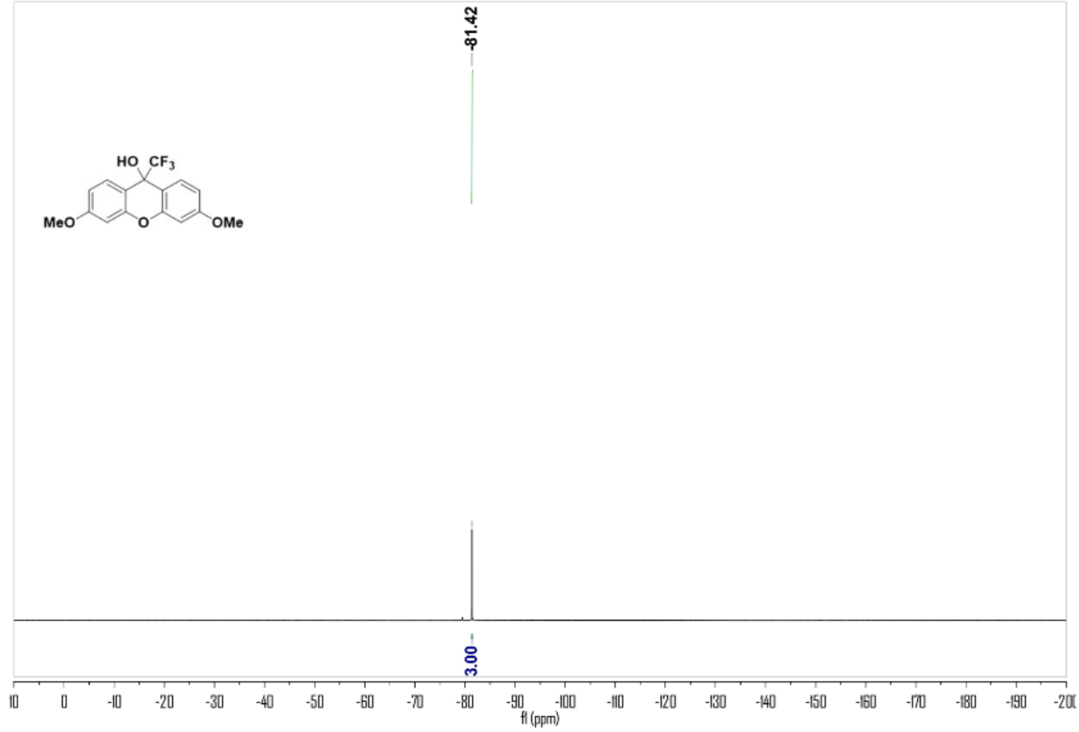

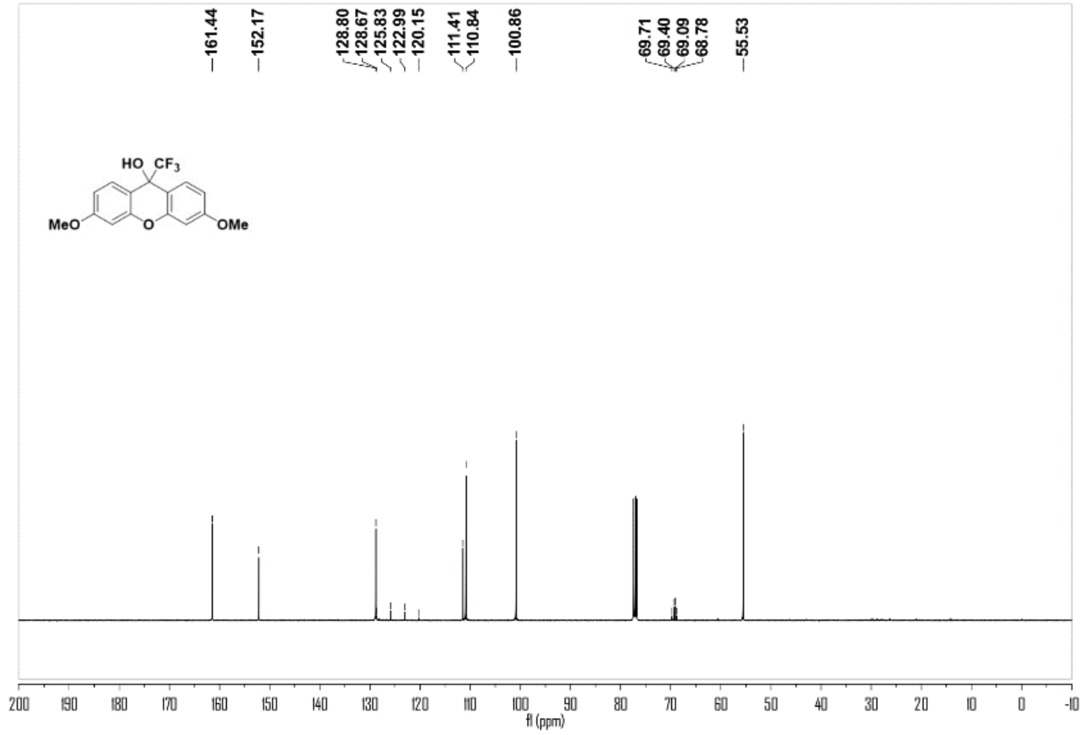

^{1}H, ^{19}F and ^{13}C NMR spectra of compound 1bb-B

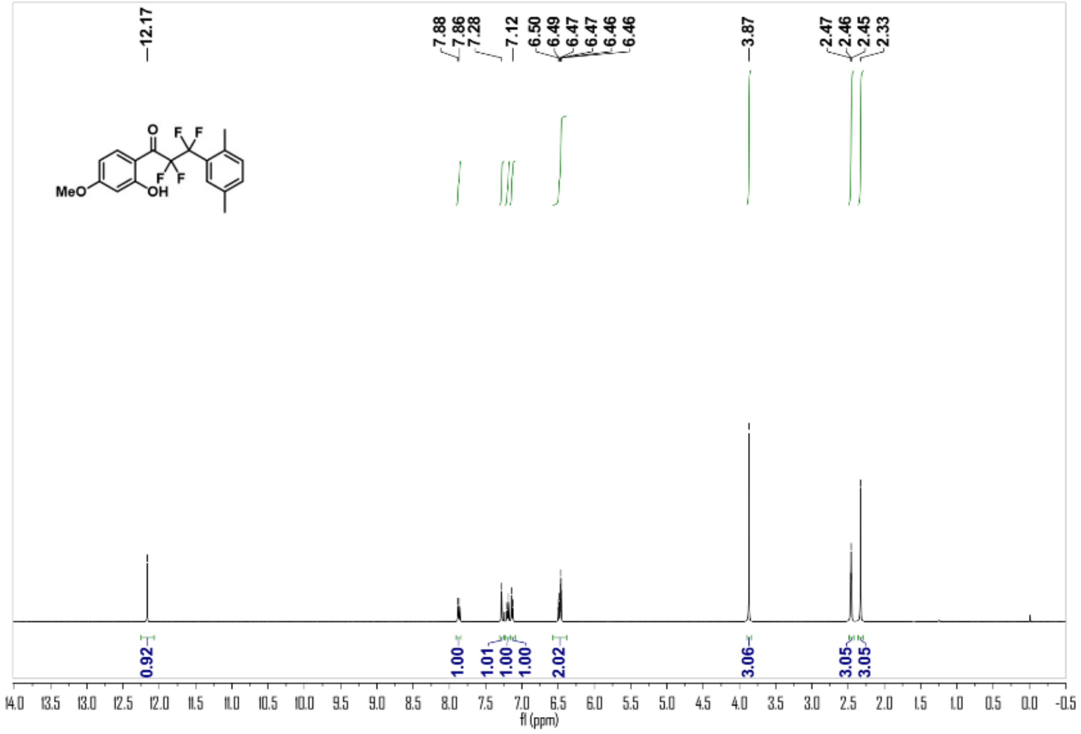

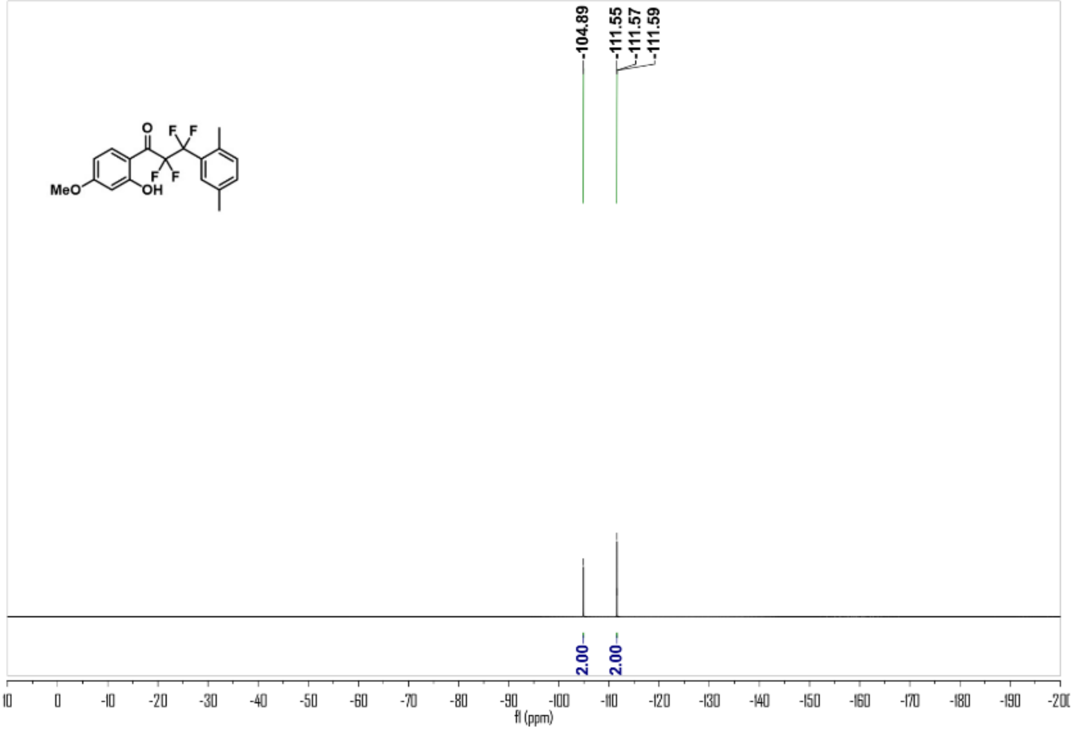

-104.89
-111.55
-111.57
-111.59
MeO
OH
O
F
2.00
2.00
fl (ppm)

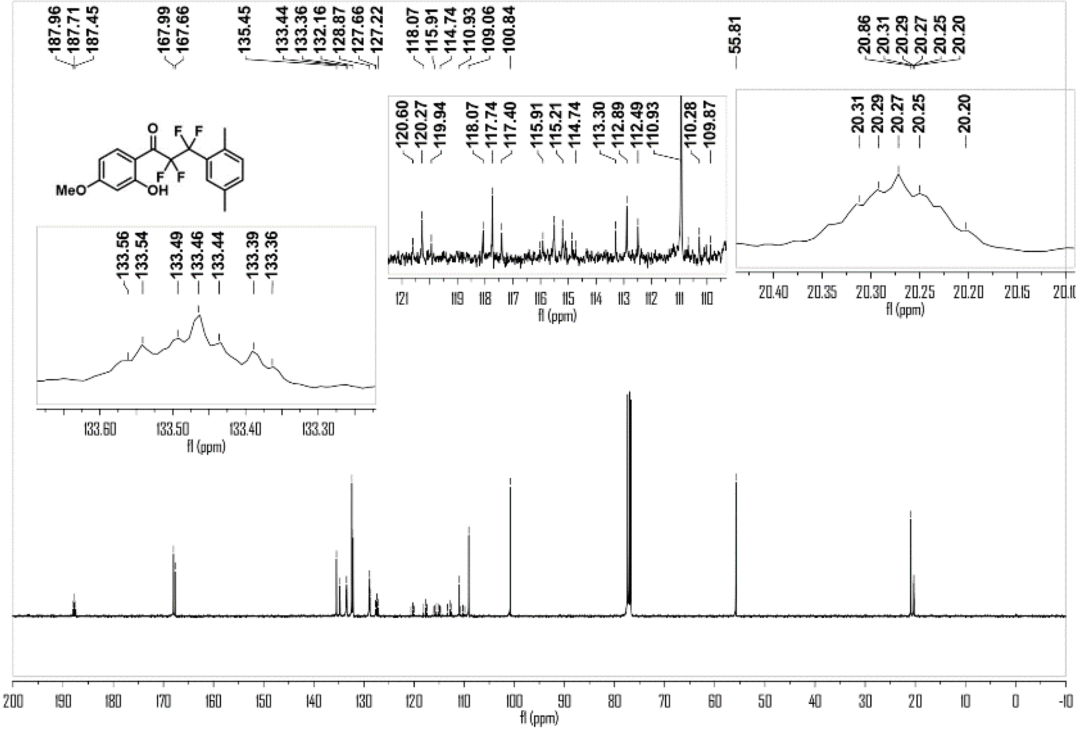

187.96
187.71
187.45
167.99
167.66
135.45
133.44
133.36
132.16
128.87
127.66
127.22
118.07
115.91
114.74
110.93
109.06
100.84
55.81
20.86
20.31
20.29
20.27
20.25
20.20
MeO
OH
O
F
133.56
133.54
133.49
133.46
133.44
133.39
133.36
120.60
120.27
119.94
118.07
117.74
117.40
115.91
115.21
114.74
113.30
112.89
112.49
110.93
110.28
109.87
fl (ppm)

^{1}H, ^{19}F and ^{13}C NMR spectra of compound 1bb

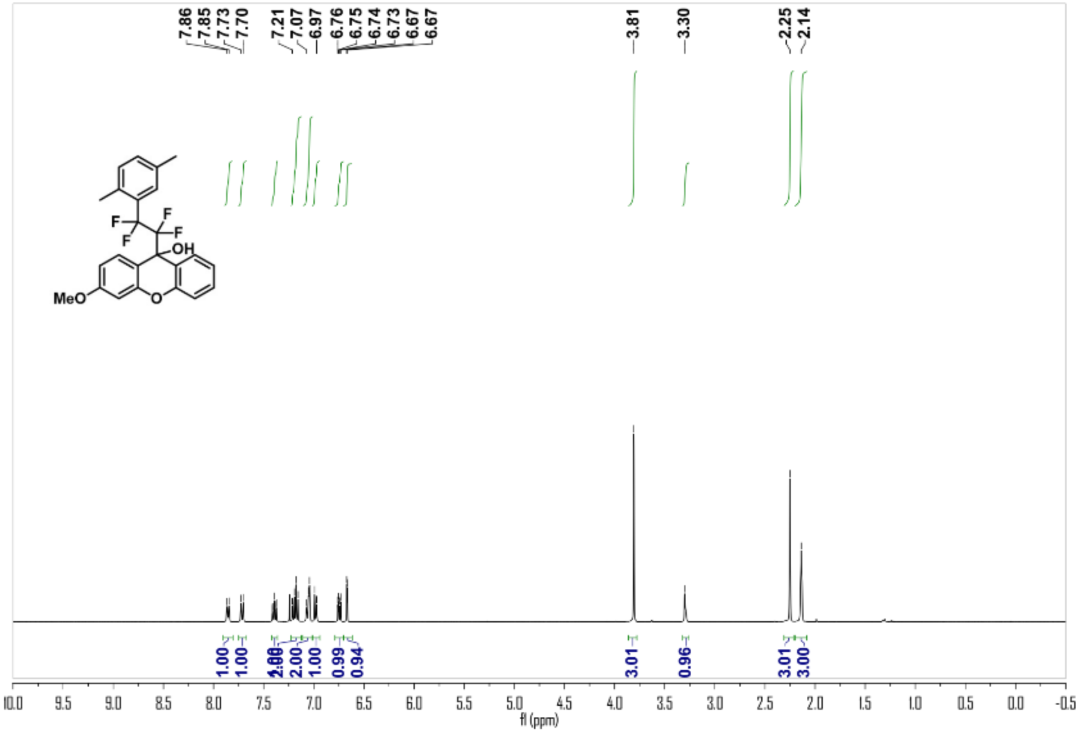

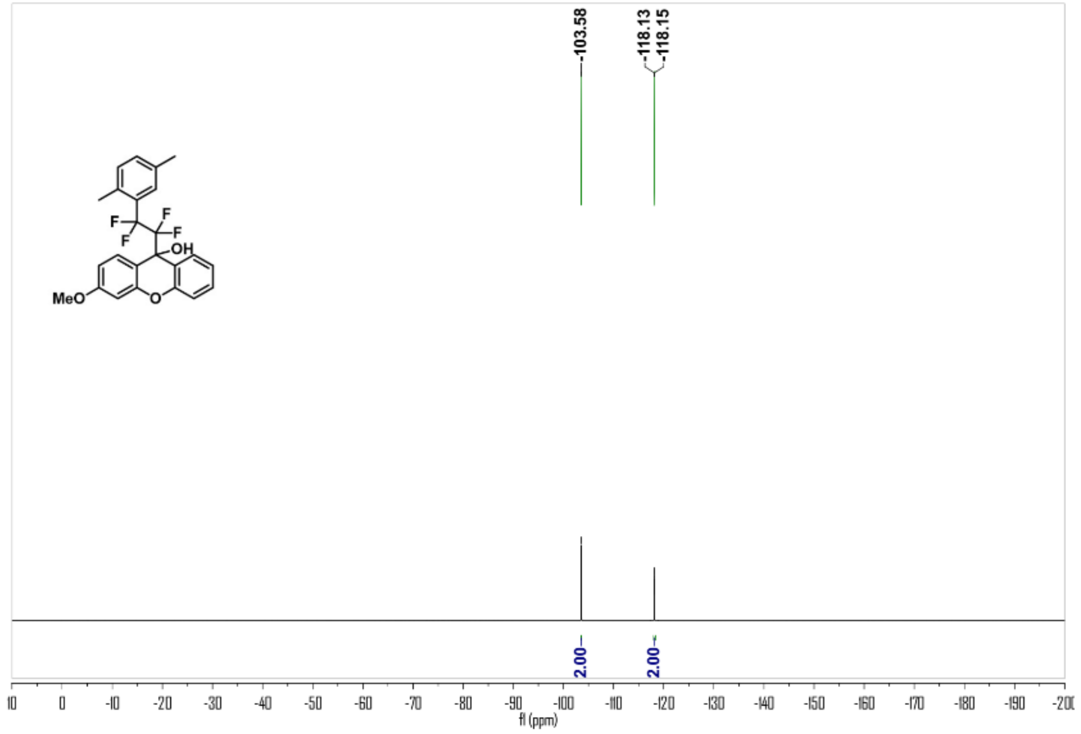

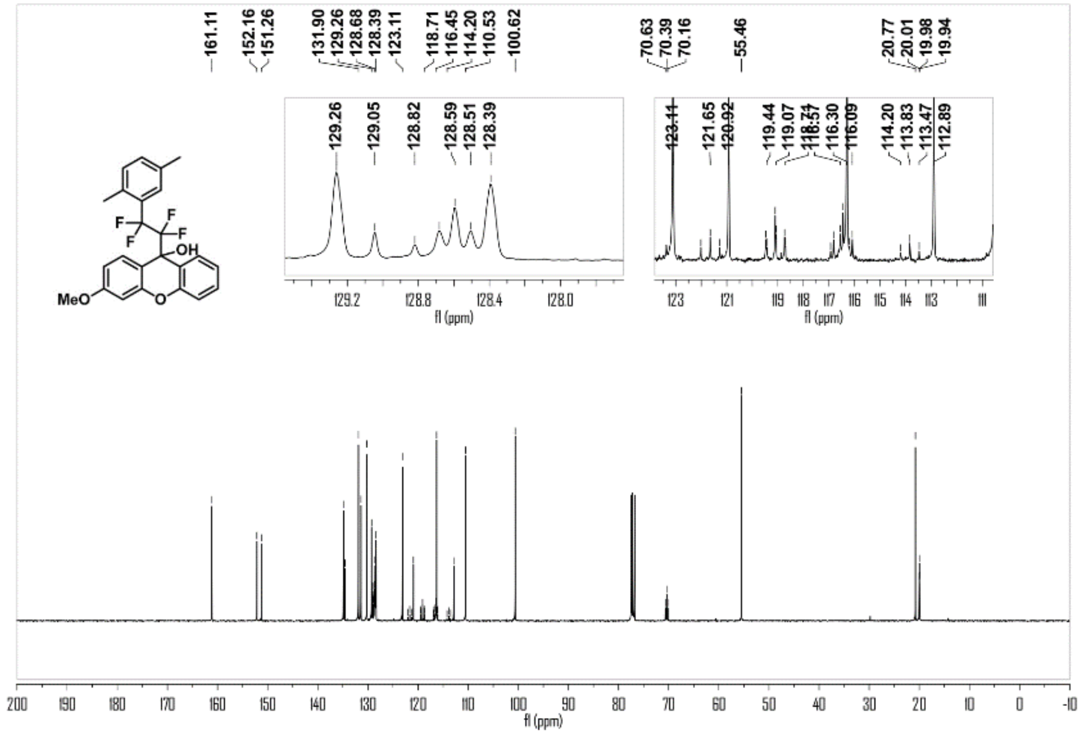

^{1}H, ^{19}F and ^{13}C NMR spectra of compound 1bc

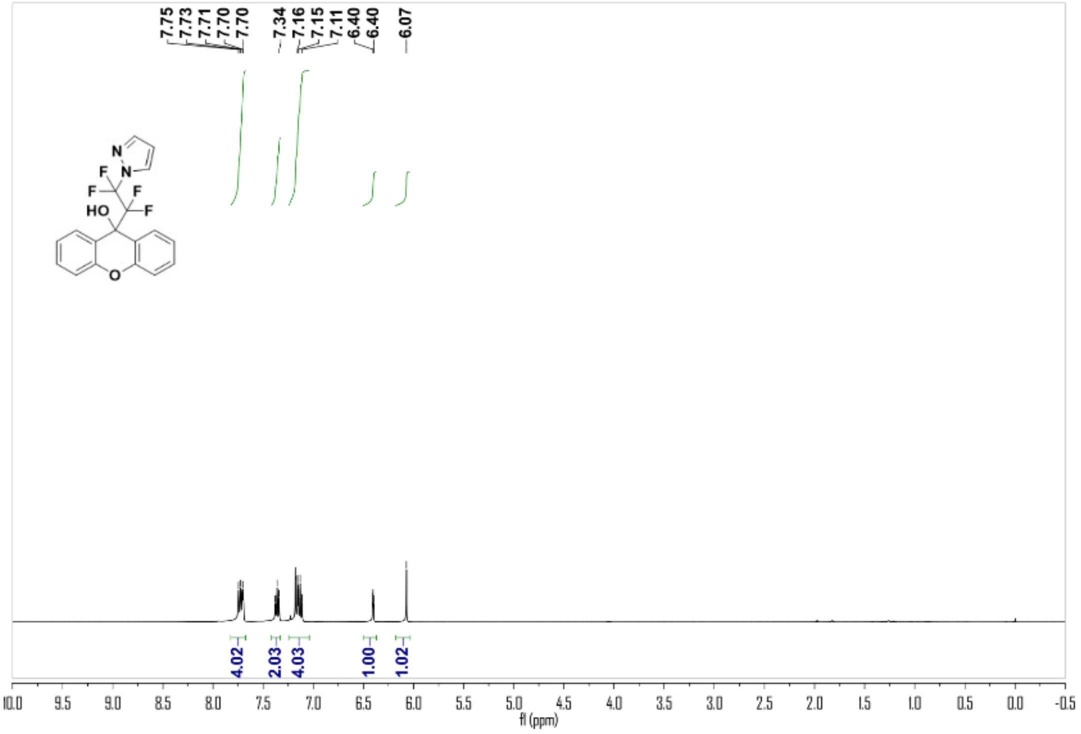

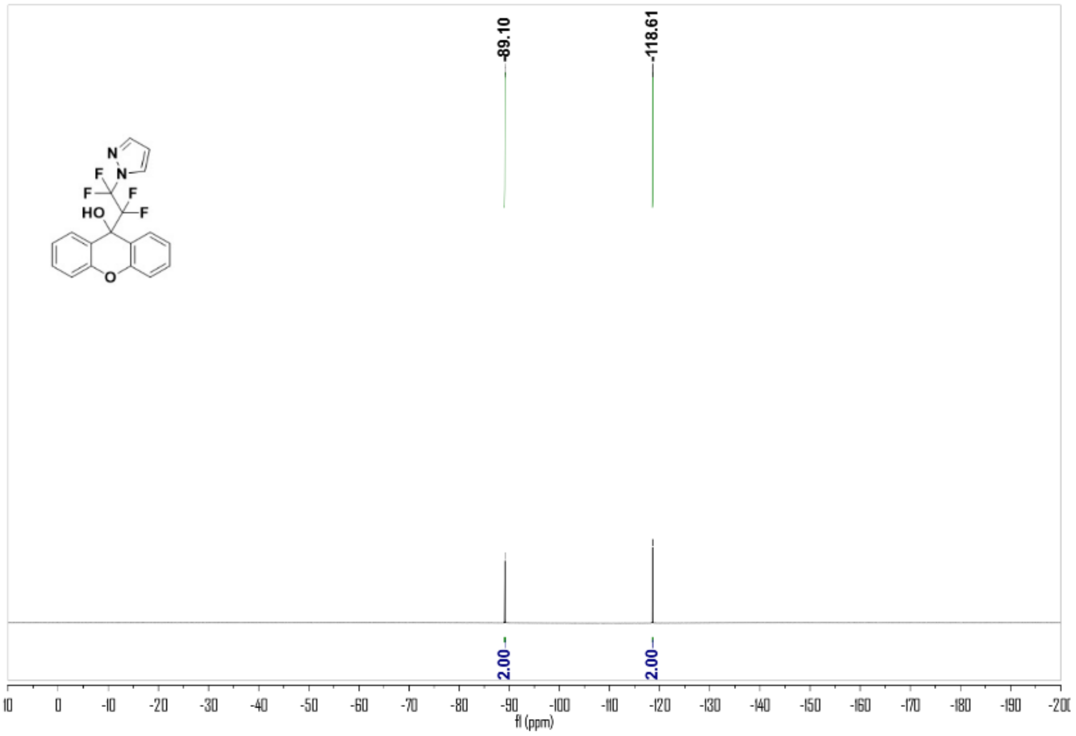
-89.10
-118.61
2.00
2.00
fl (ppm)

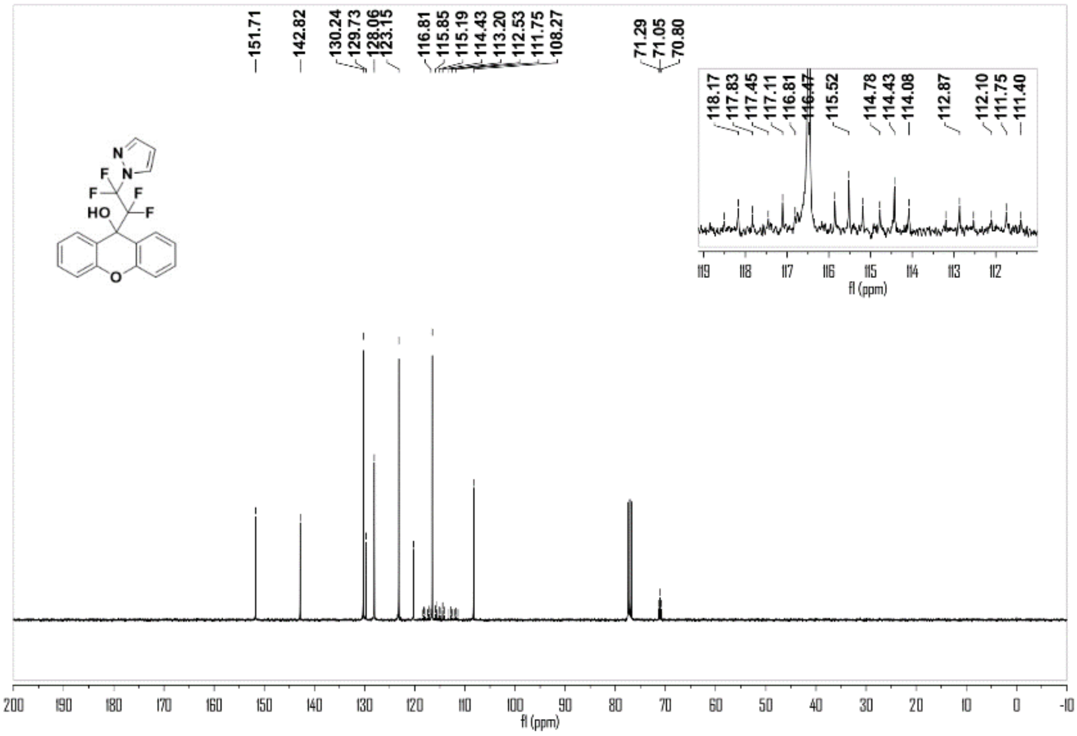
151.71
142.82
130.24
129.73
128.06
123.15
116.81
115.85
115.19
114.43
113.20
112.53
111.75
108.27
71.29
71.05
70.80
118.17
117.83
117.45
117.11
116.81
116.47
115.52
114.78
114.43
114.08
112.87
112.10
111.75
111.40
fl (ppm)

^{1}H, ^{19}F and ^{13}C NMR spectra of compound 1bd-A

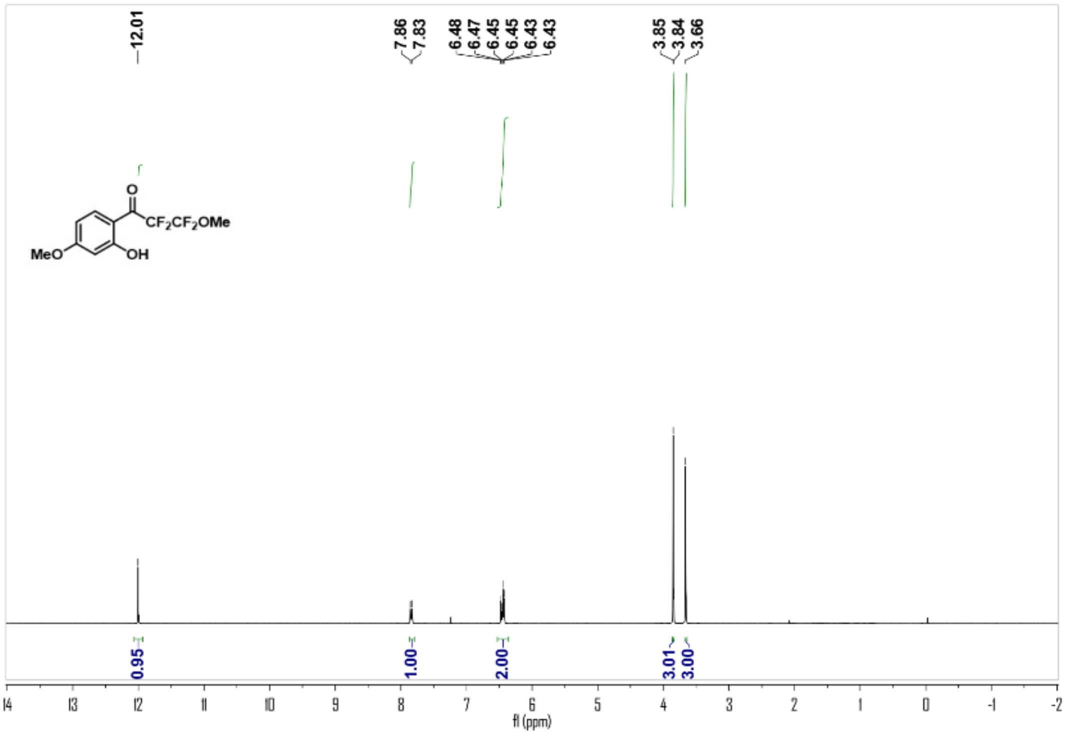

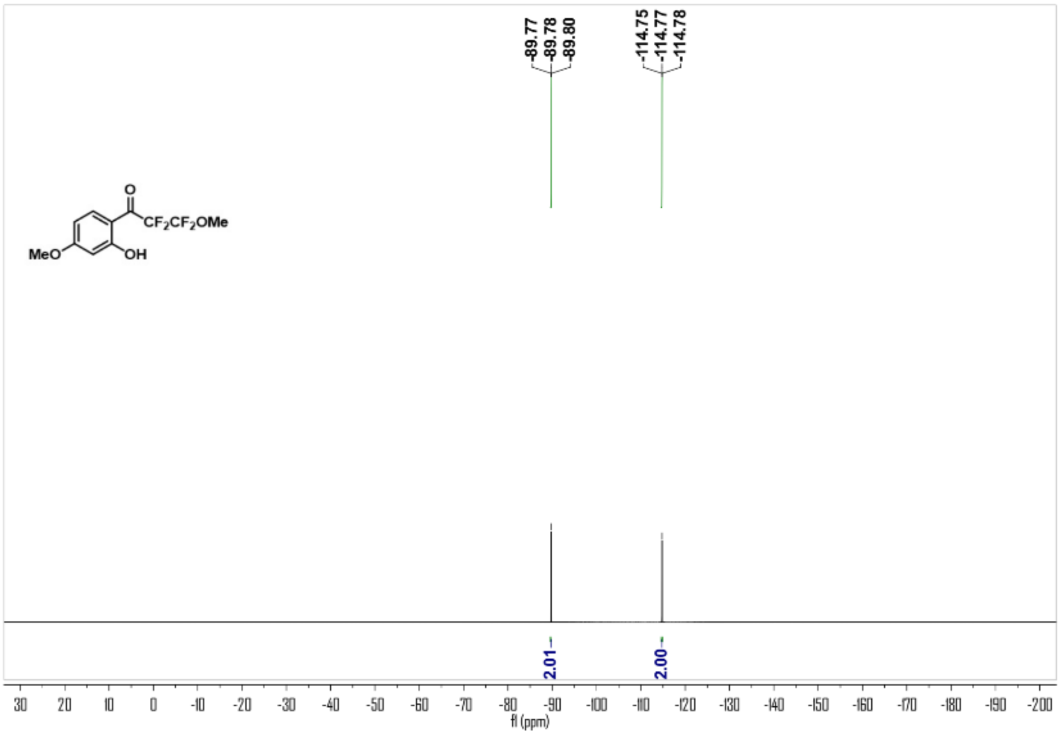

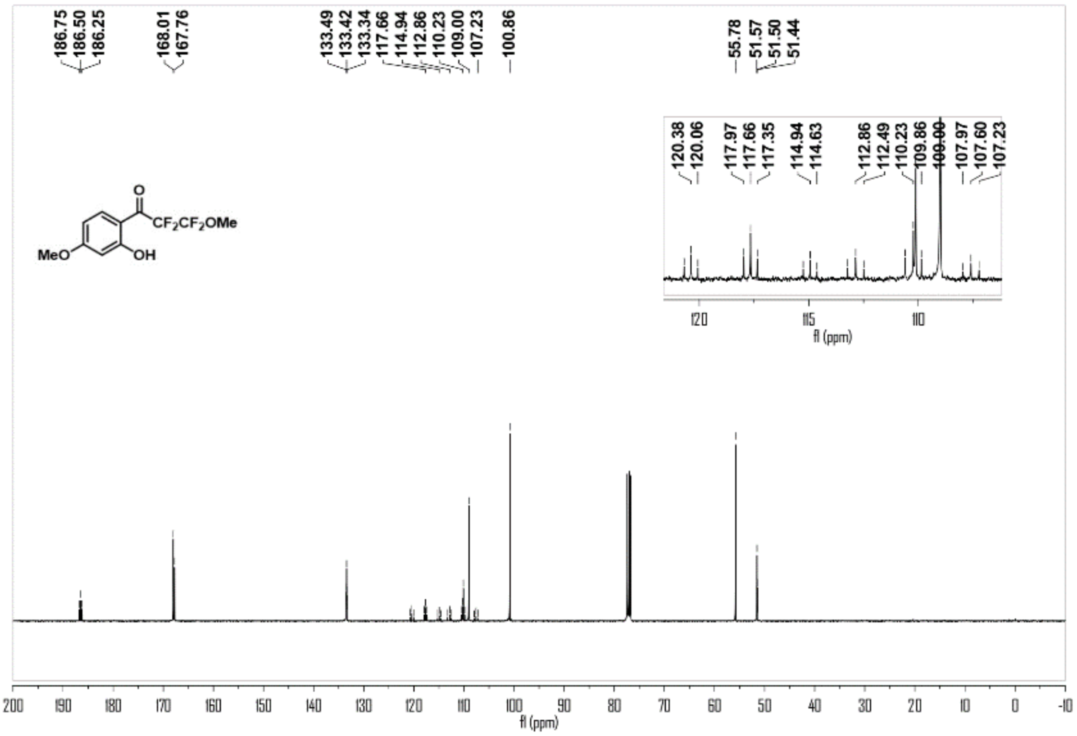

^{1}H, ^{19}F and ^{13}C NMR spectra of compound 1bd

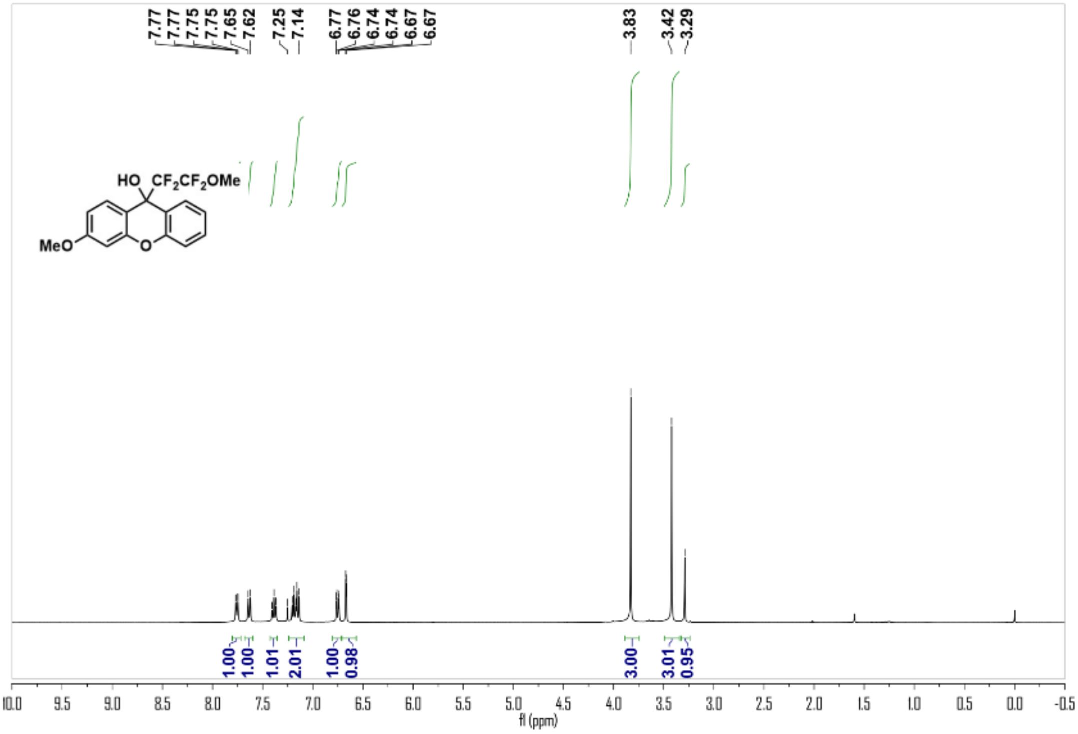

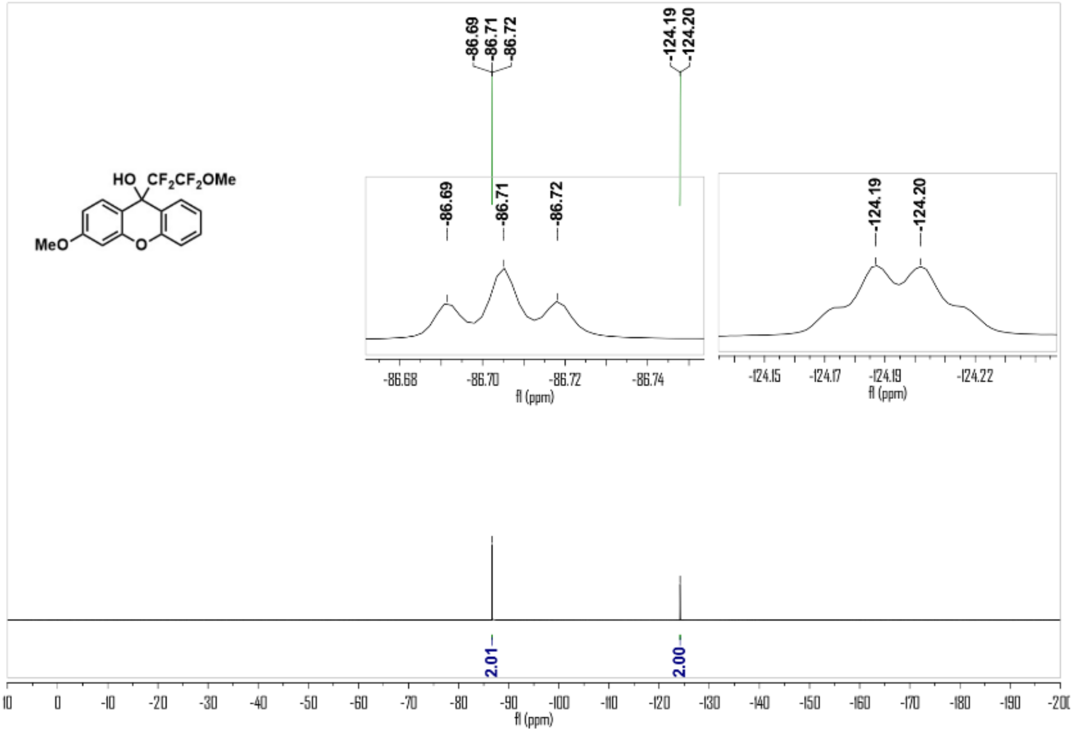
HO CF2CF2OMe
MeO
O
-86.69
-86.71
-86.72
-124.19
-124.20
2.01
2.00
fl (ppm)

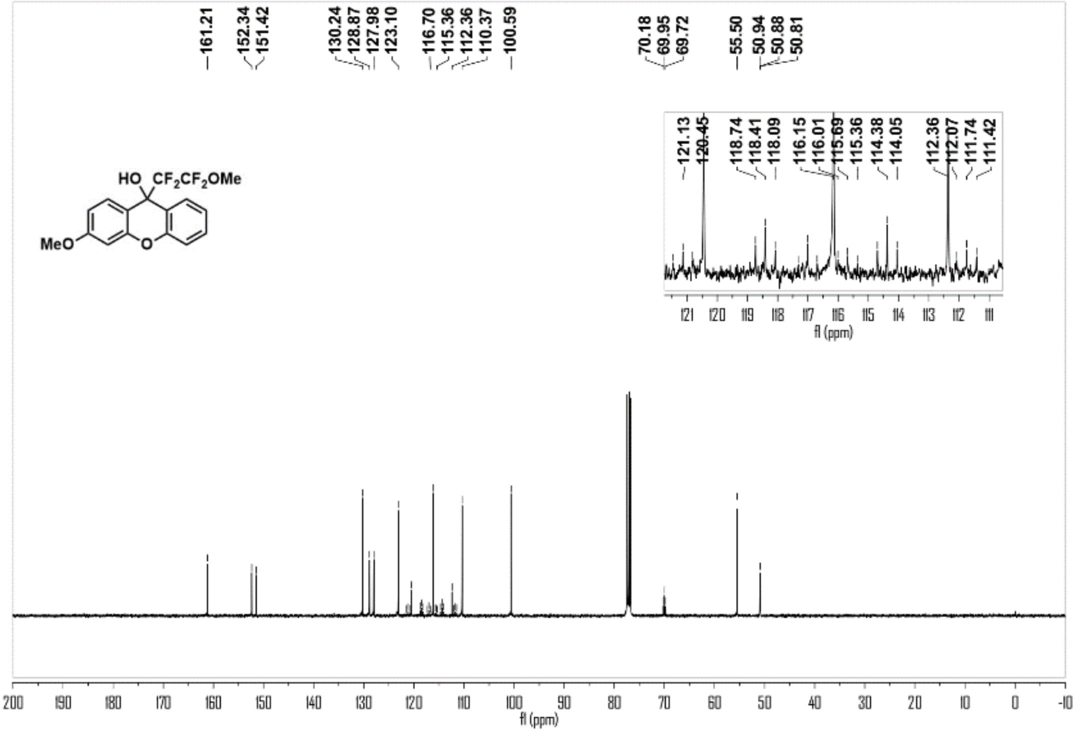
HO CF2CF2OMe
MeO
O
161.21
152.34
151.42
130.24
128.87
127.98
123.10
116.70
115.36
112.36
110.37
100.59
70.18
69.95
69.72
55.50
50.94
50.88
50.81
fl (ppm)

^{1}H, ^{19}F and ^{13}C NMR spectra of compound 1be

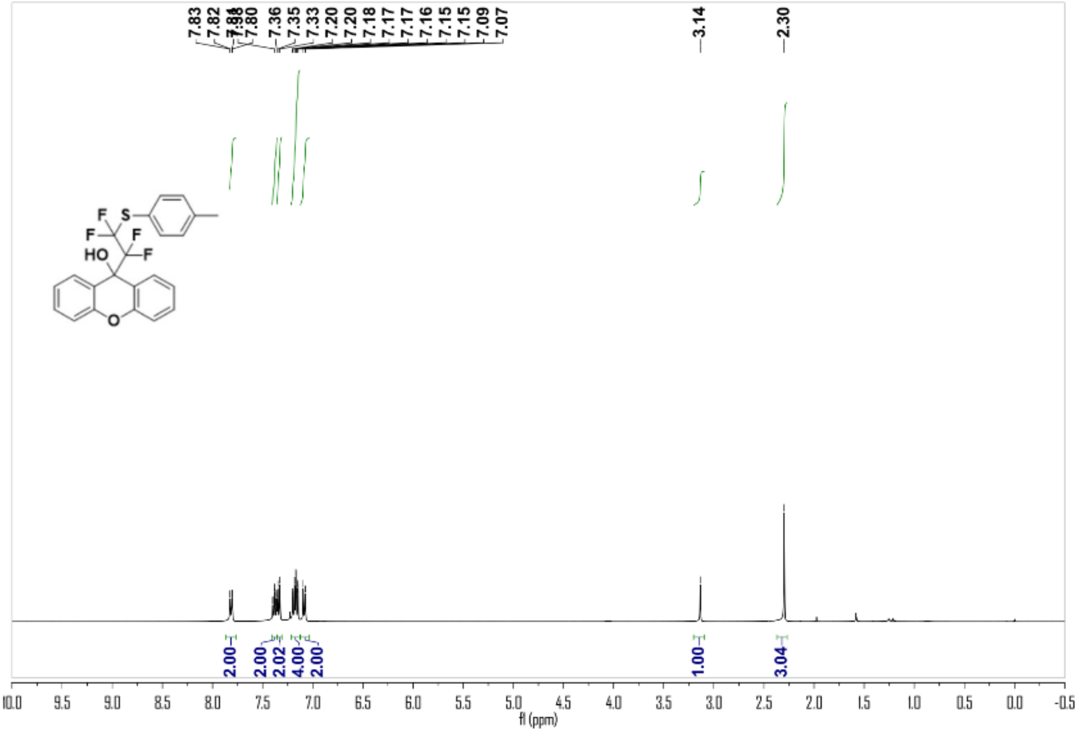

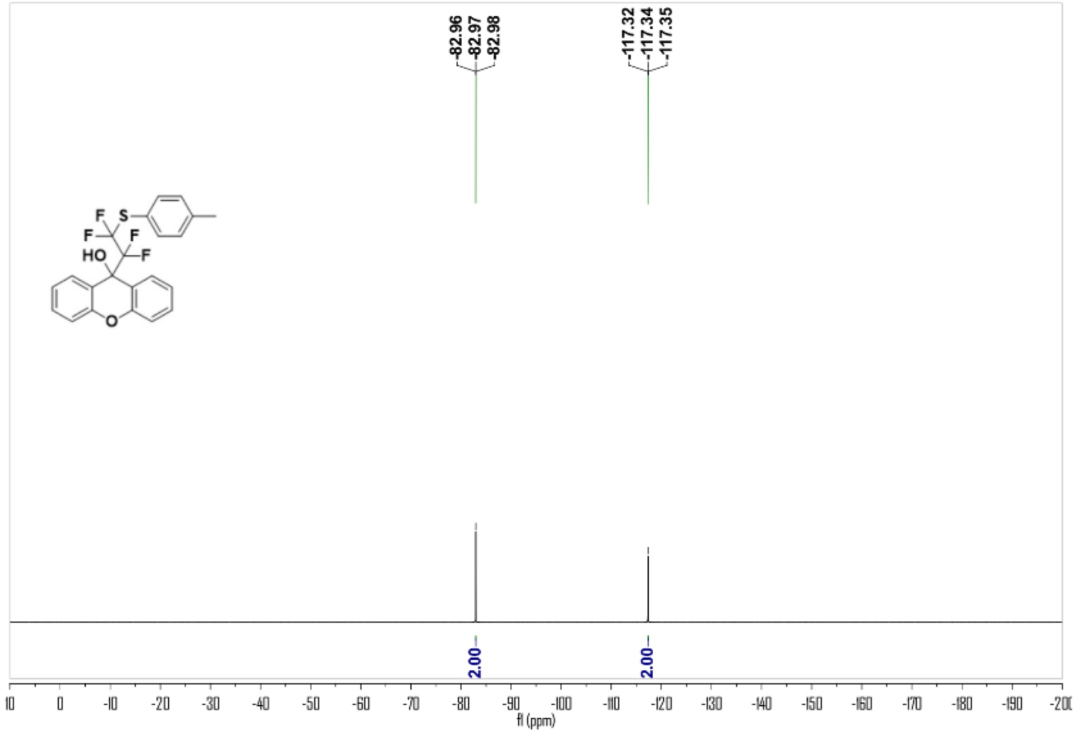

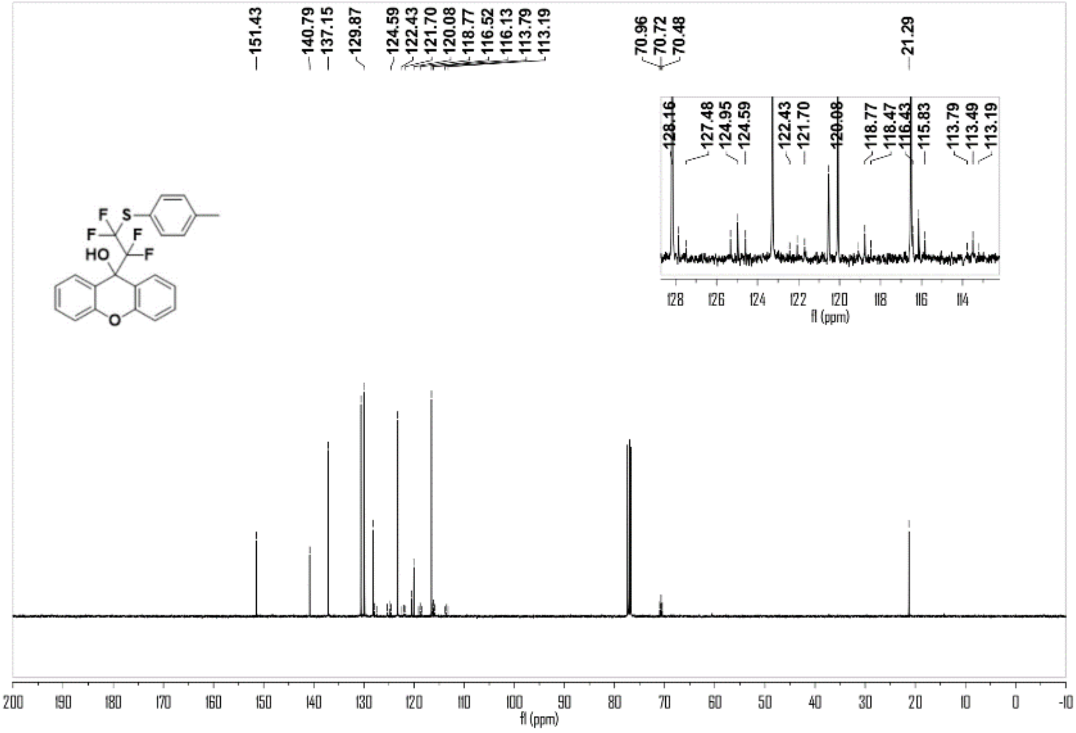

^{1}H, ^{19}F and ^{13}C NMR spectra of compound 1bf-A

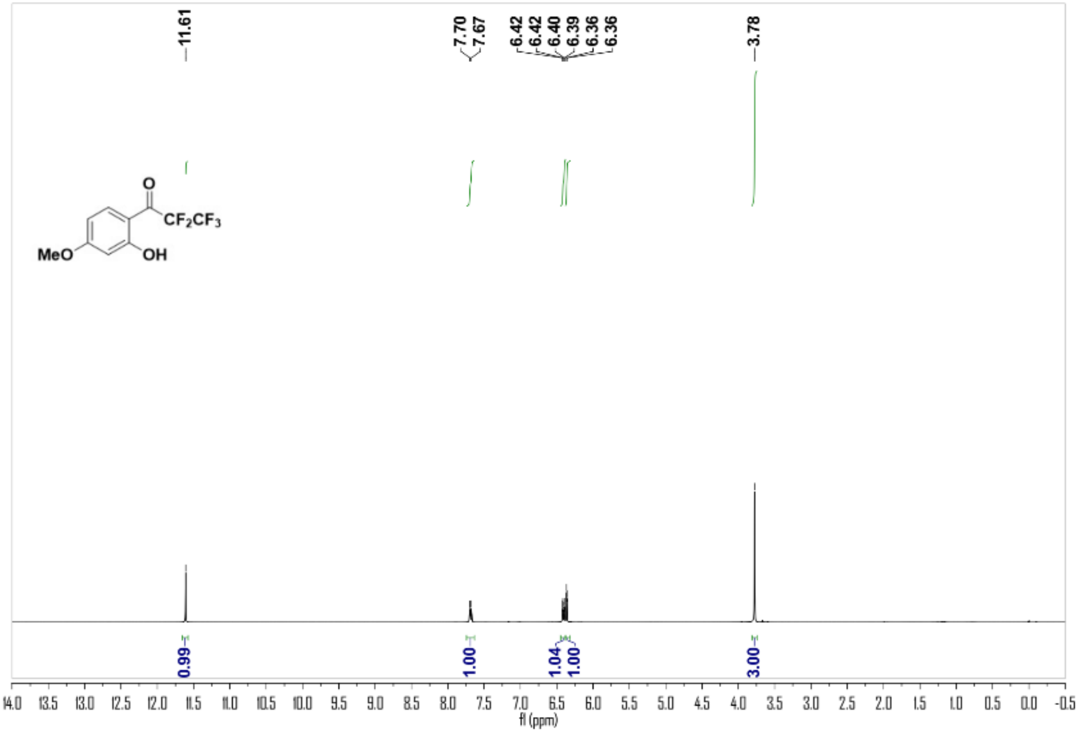

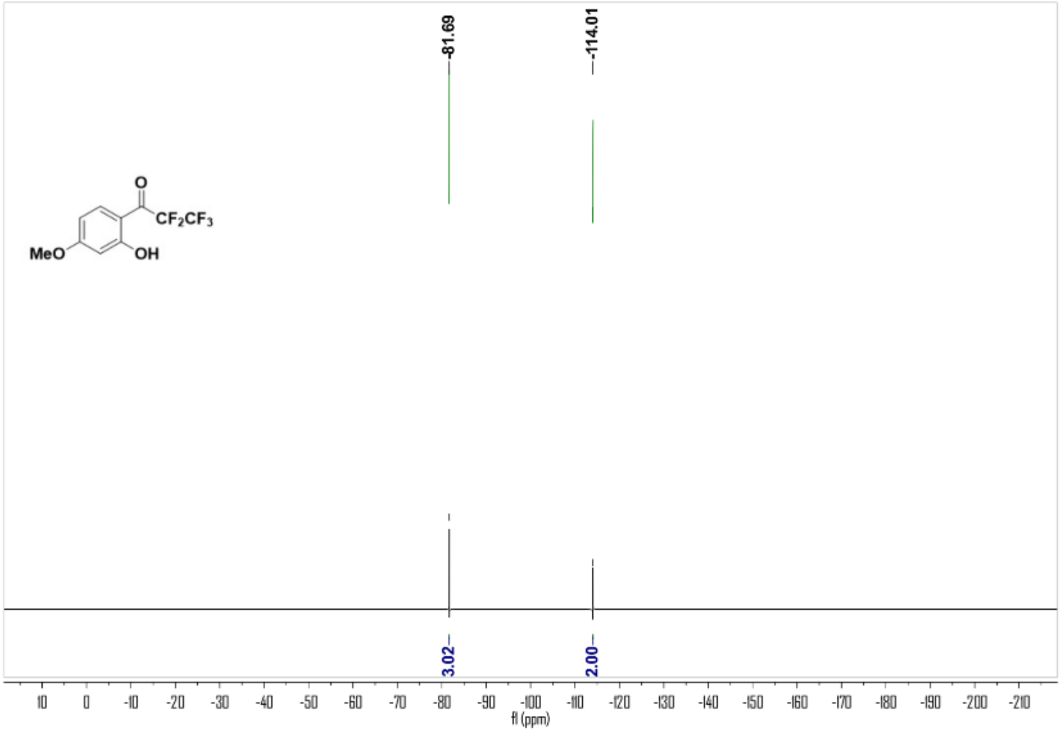
-81.69
-114.01
CF2CF3
O
MeO
OH
3.02
2.00
f1 (ppm)

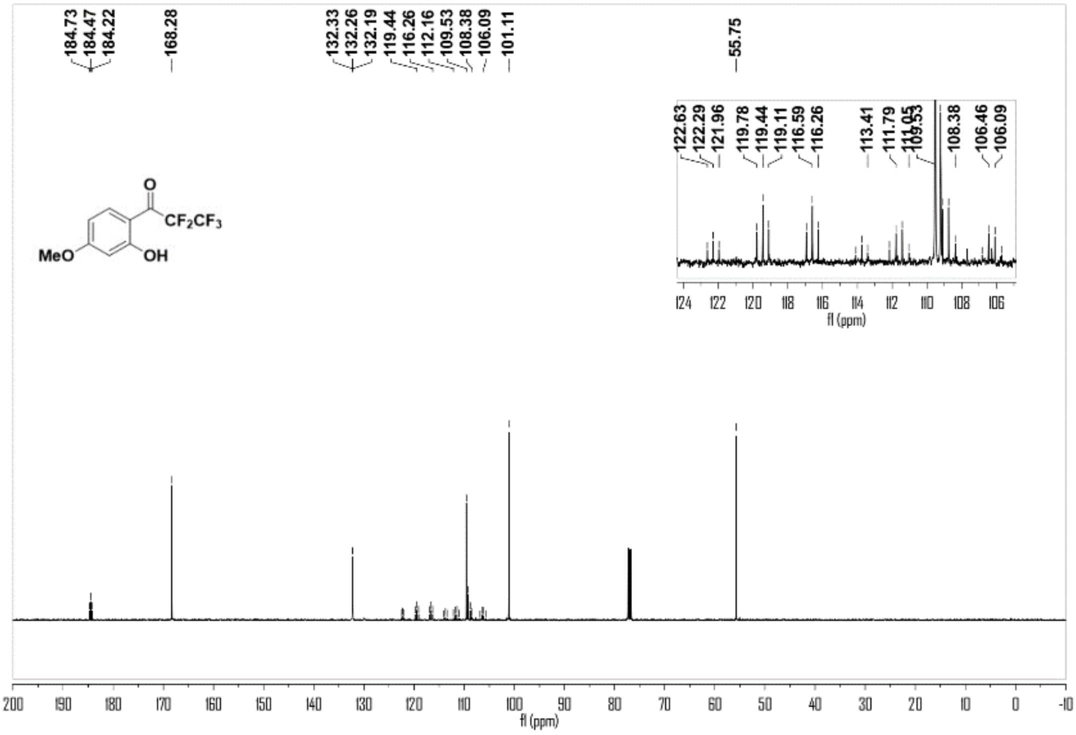
184.73
184.47
184.22
168.28
132.33
132.26
132.19
119.44
116.26
112.16
109.53
108.38
106.09
101.11
55.75
CF2CF3
O
MeO
OH
f1 (ppm)

^{1}H, ^{19}F and ^{13}C NMR spectra of compound 1bf

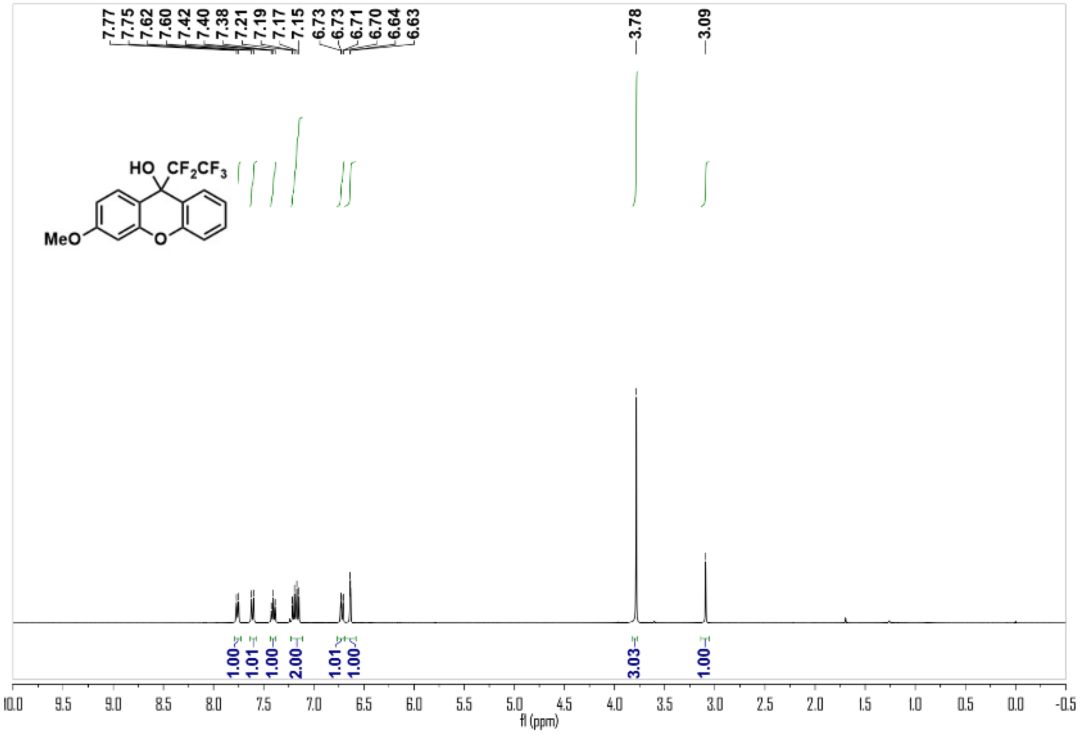

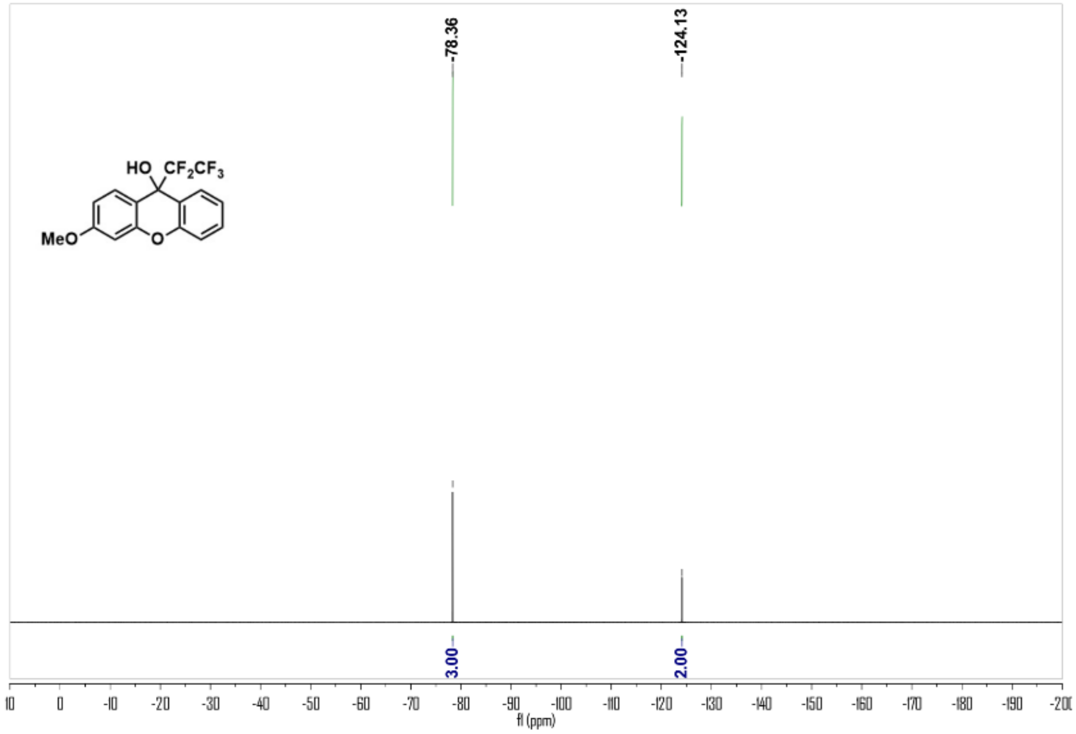

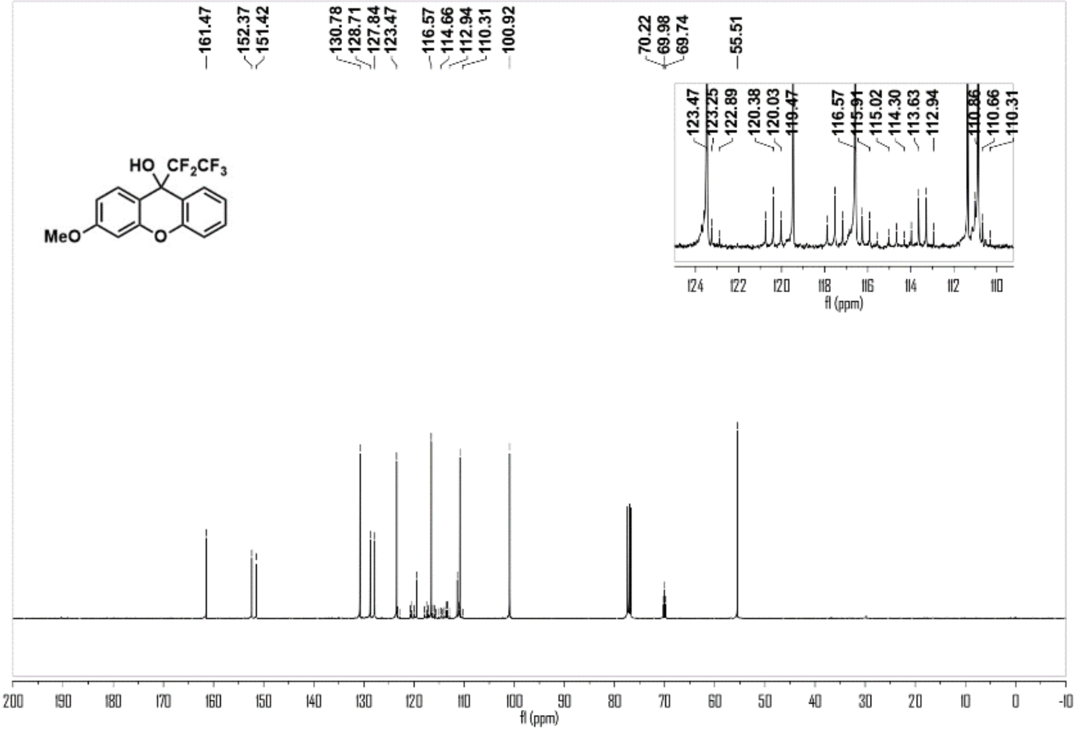

^{1}H and ^{13}C NMR spectra of compound 2h

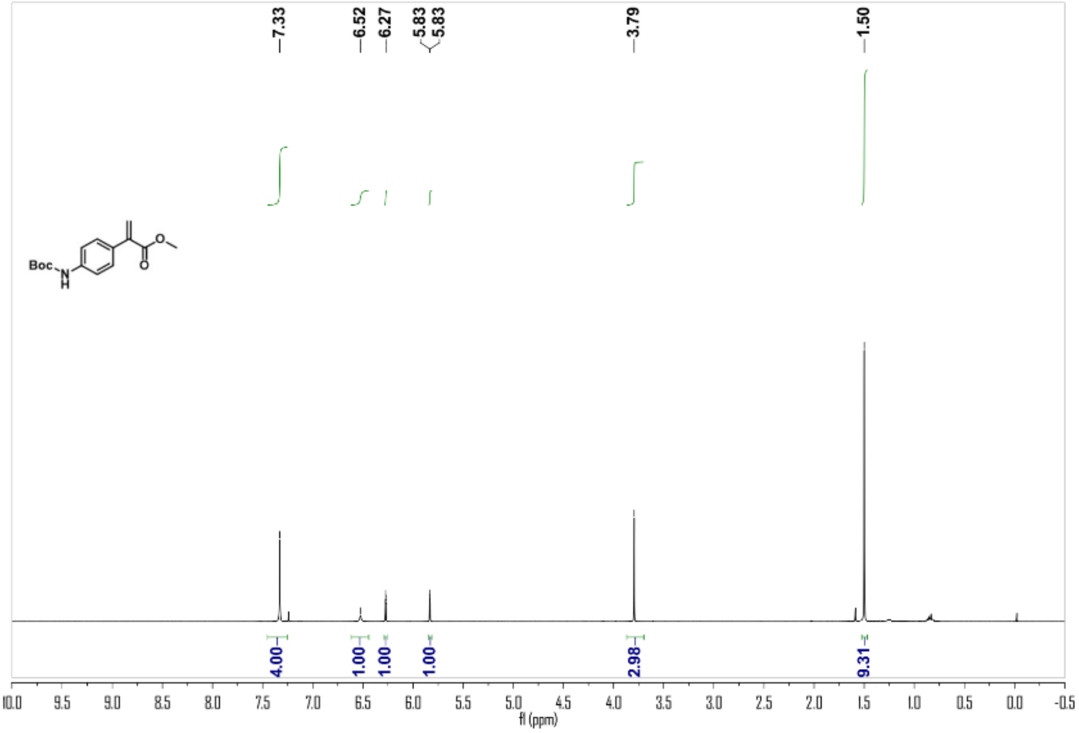

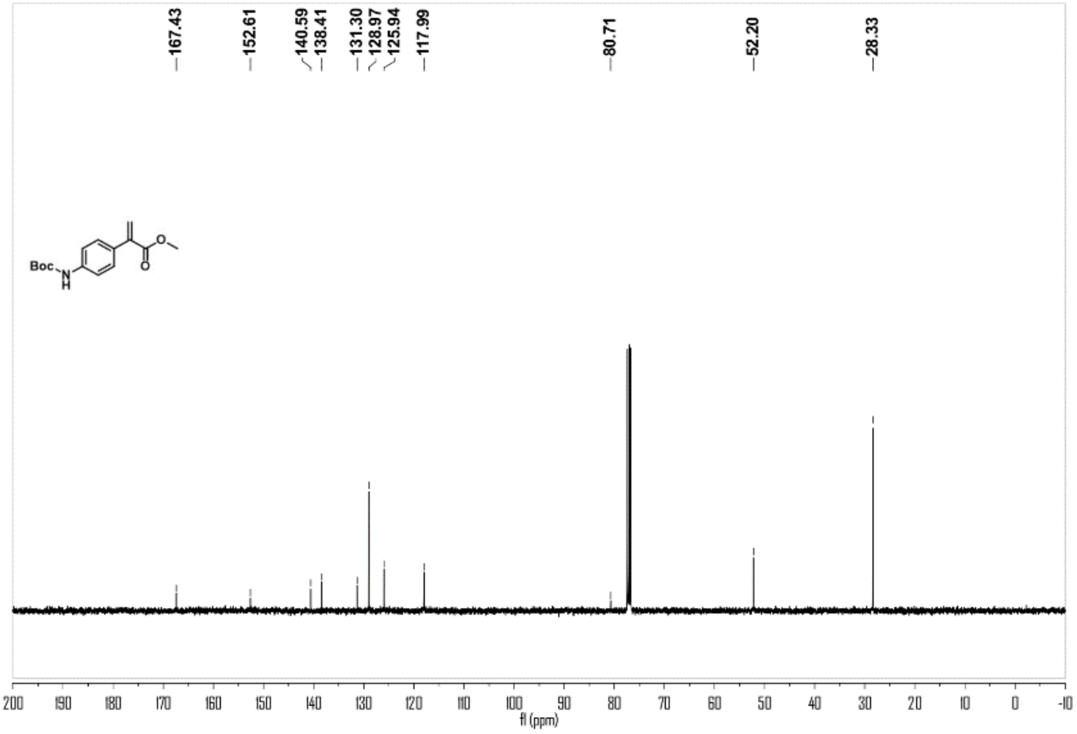

^{1}H and ^{13}C NMR spectra of compound 2j

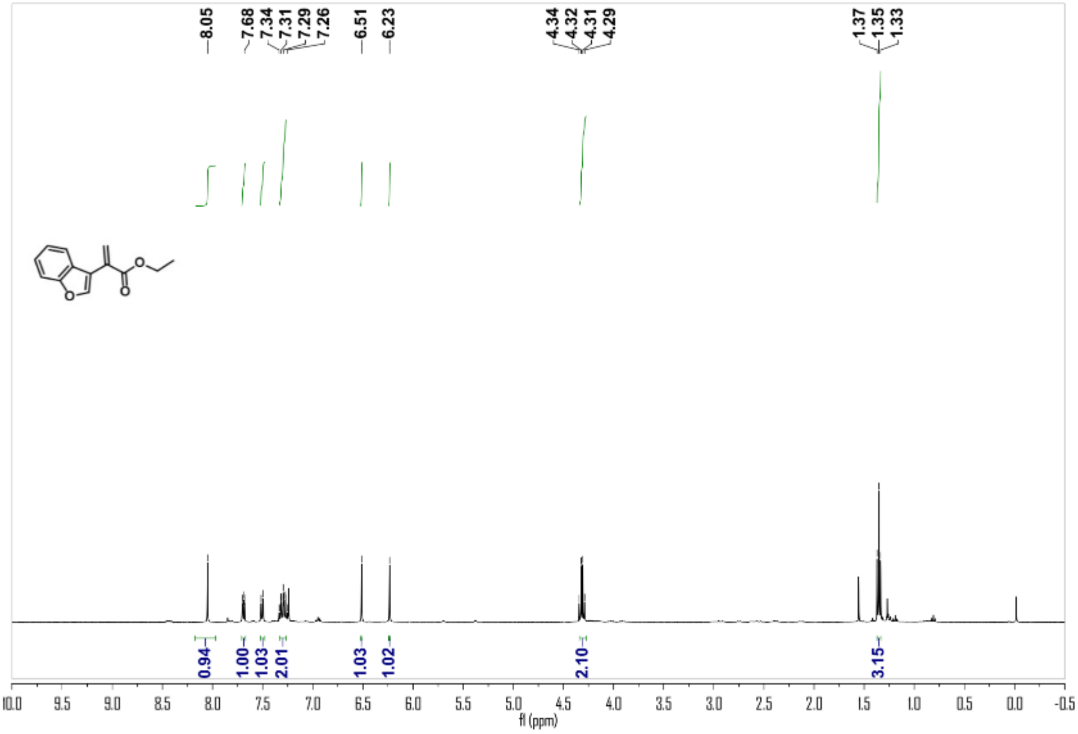

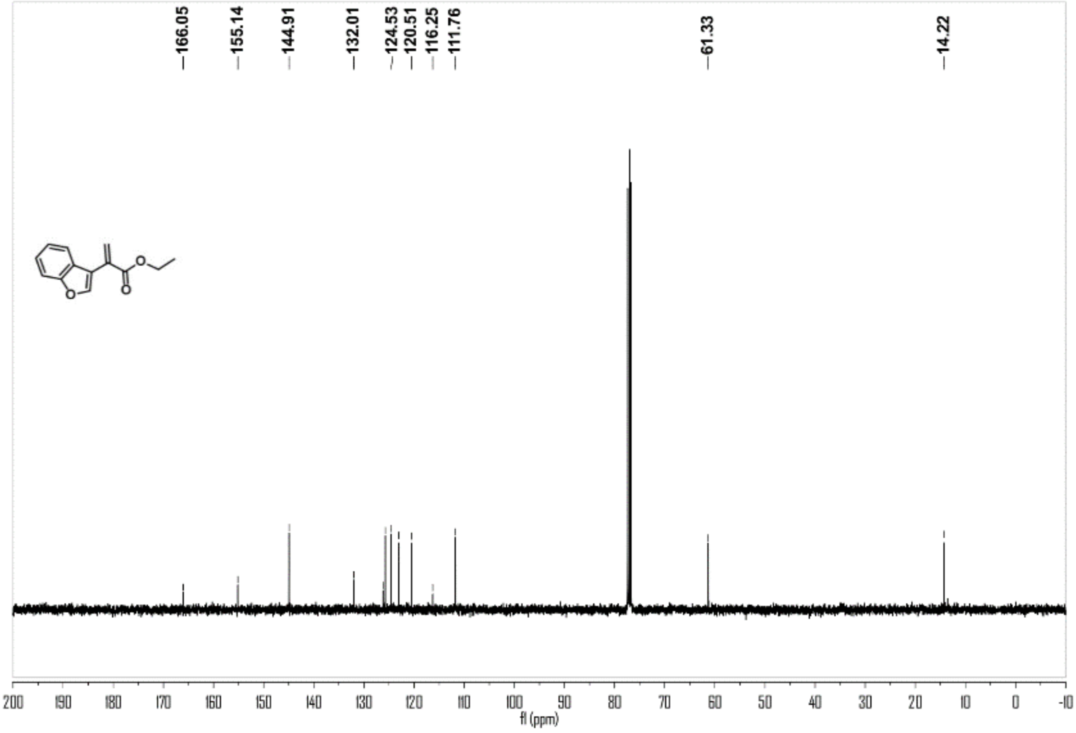

^{1}H and ^{13}C NMR spectra of compound 2k

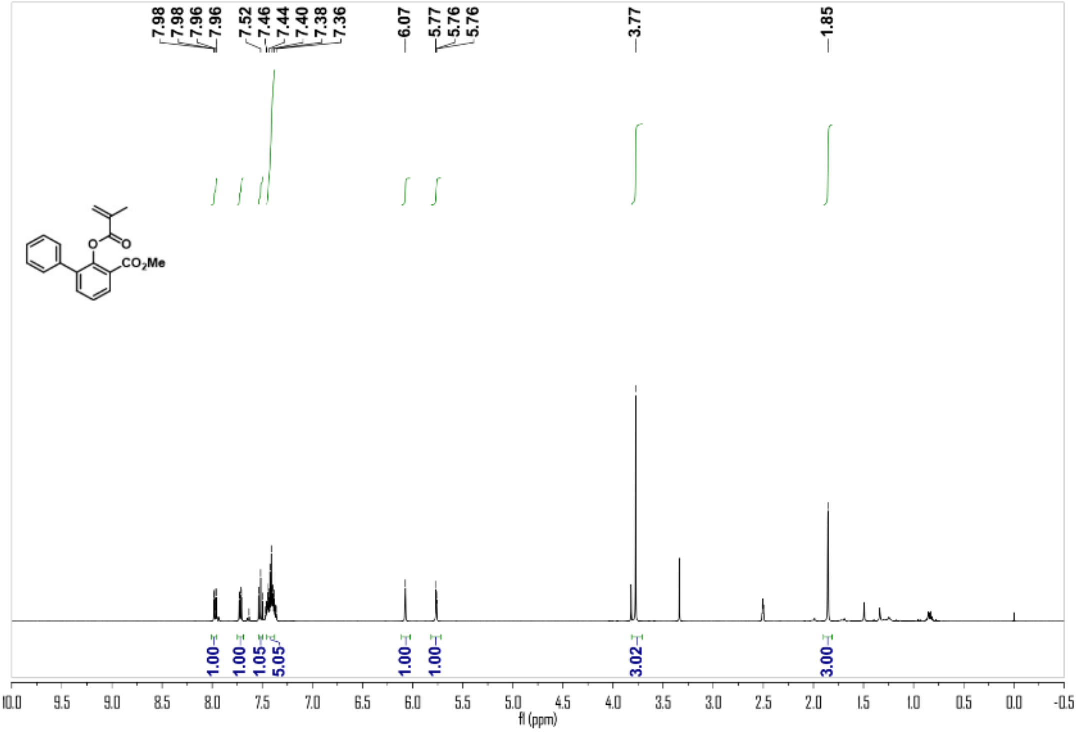

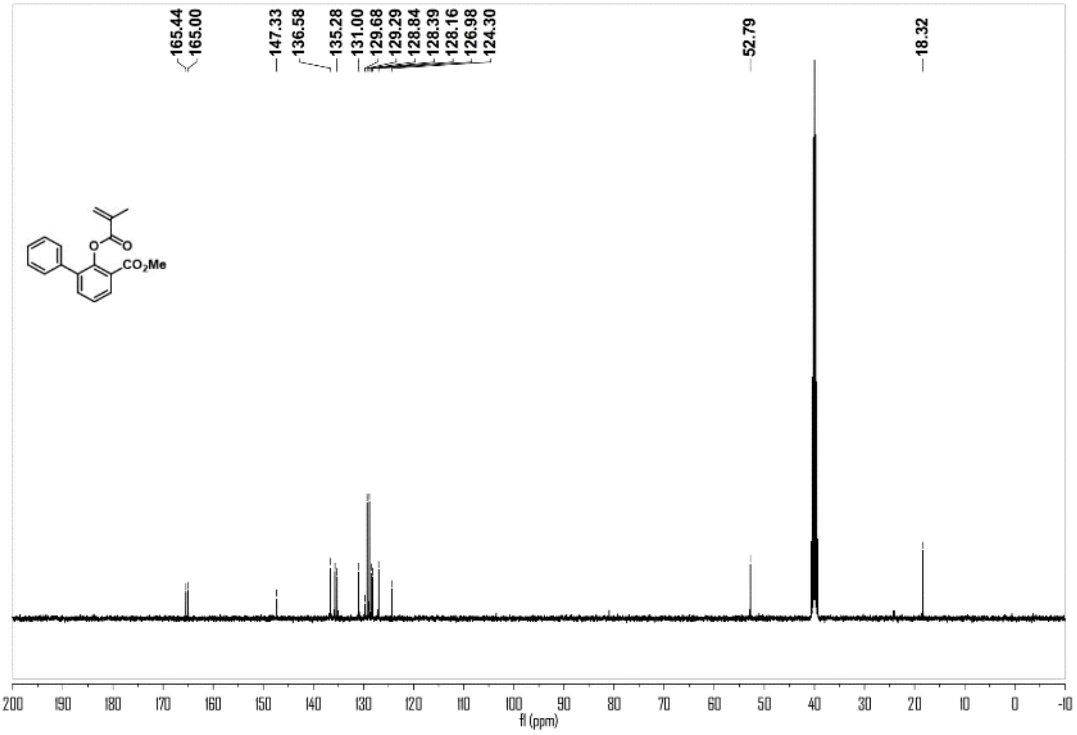

^{1}H and ^{13}C NMR spectra of compound 2m

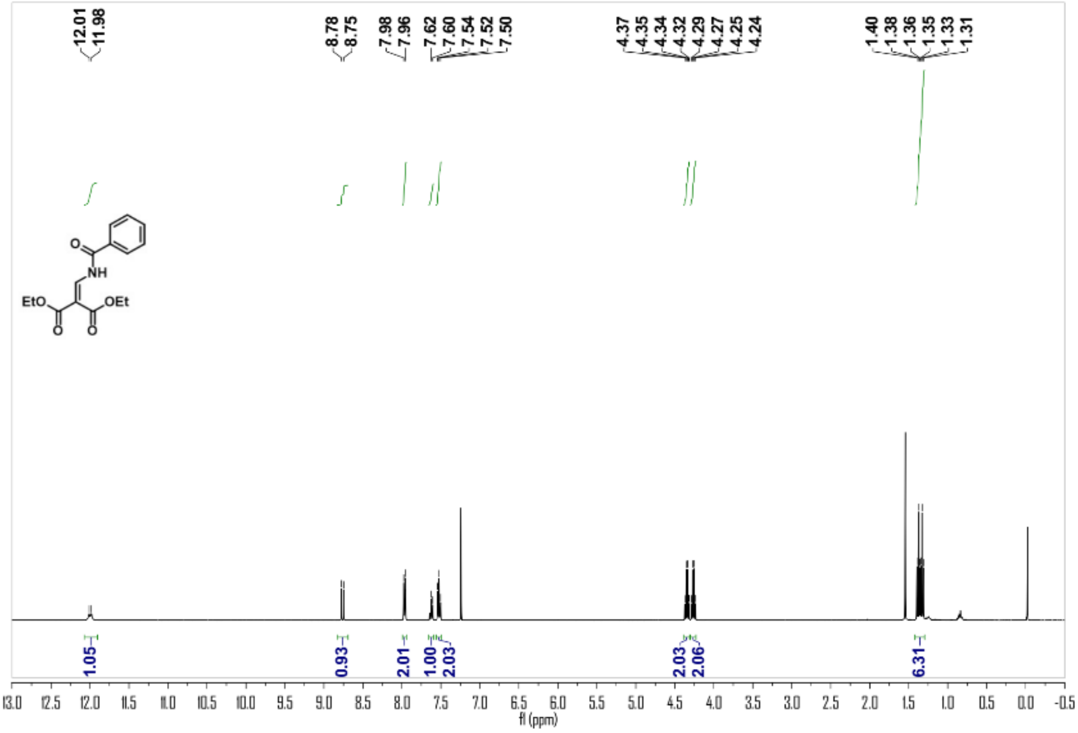

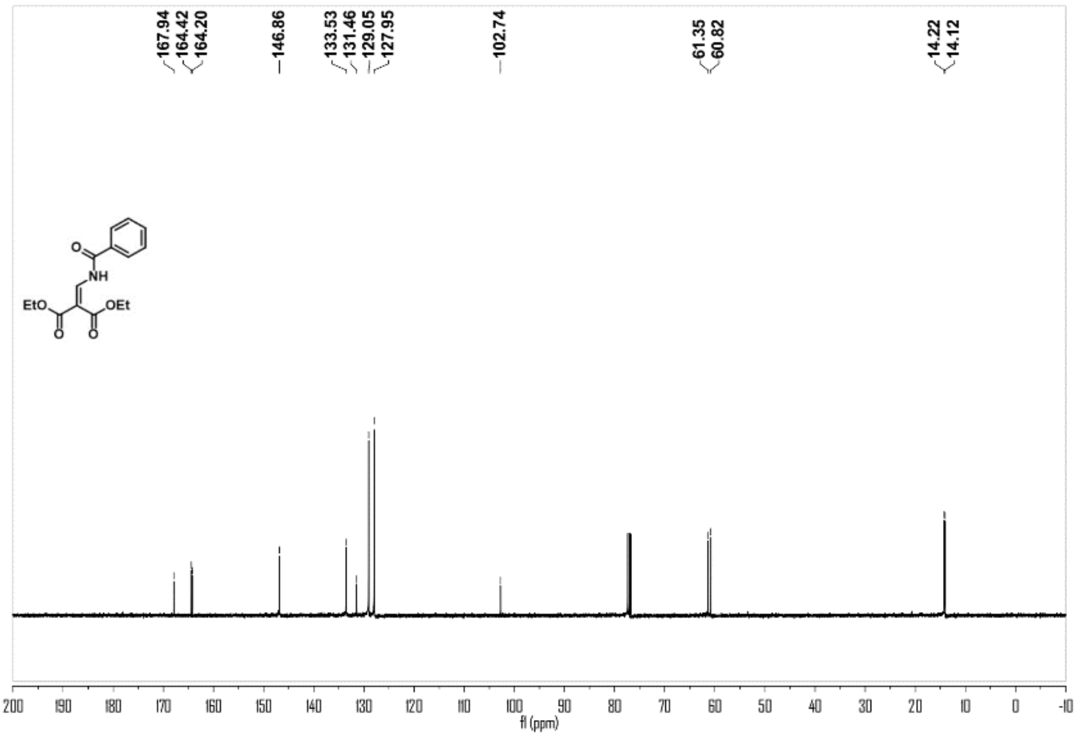

^{1}H and ^{13}C NMR spectra of compound 2p

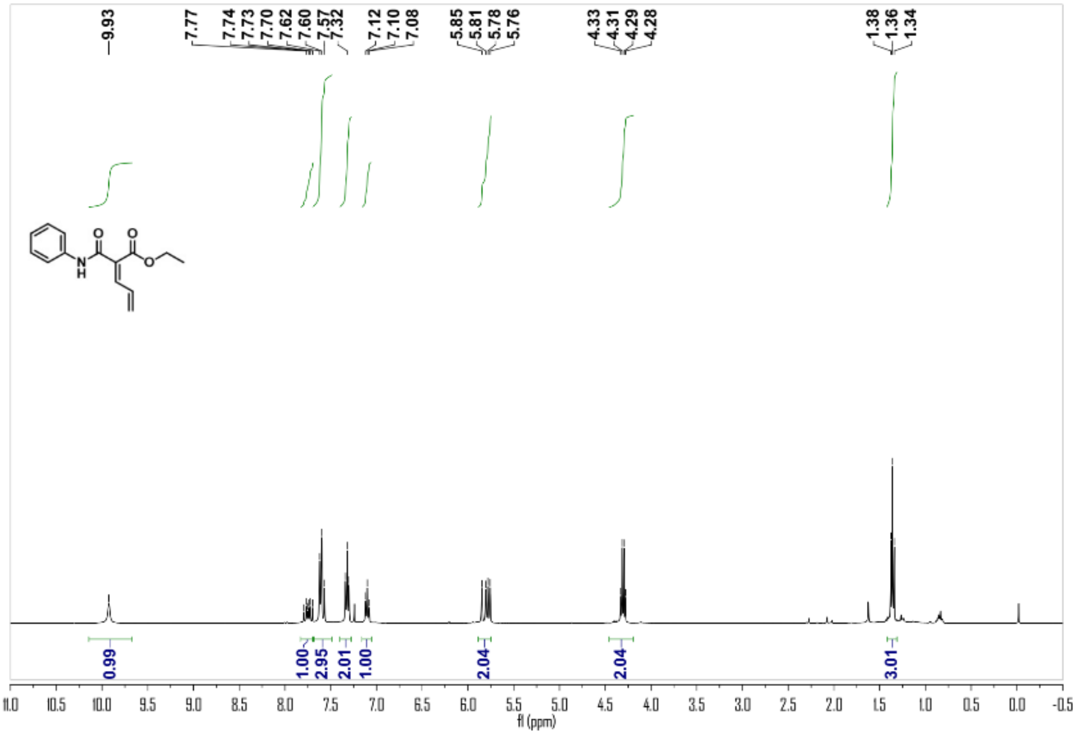

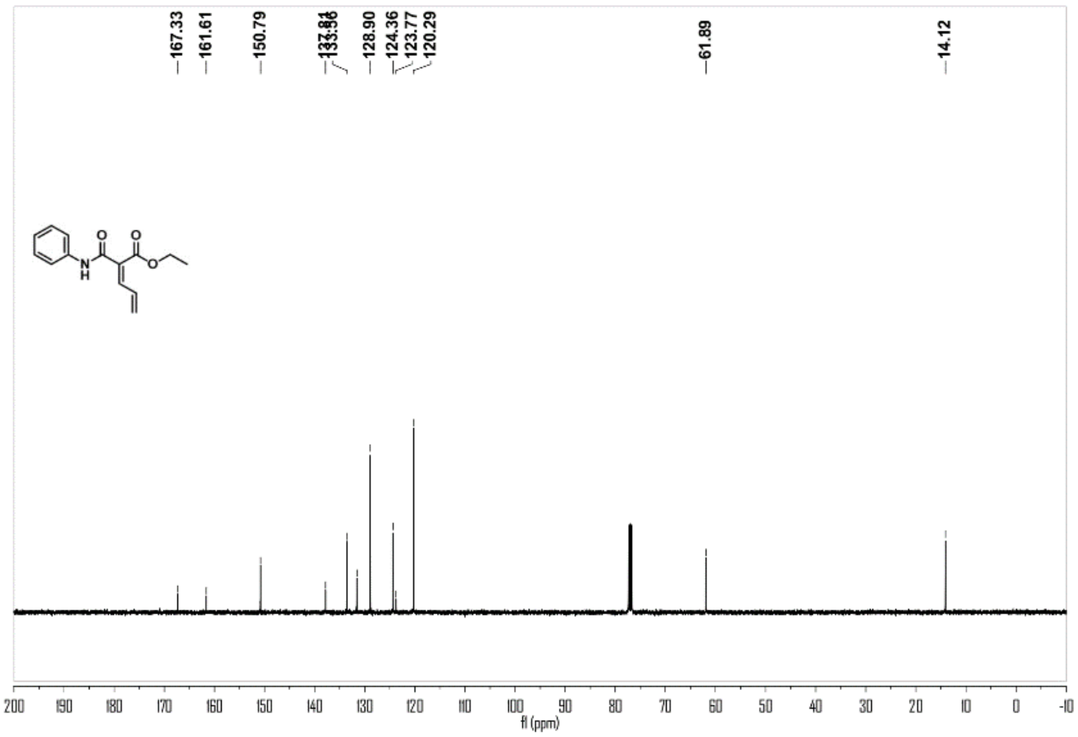

^{1}H and ^{13}C NMR spectra of compound 2u

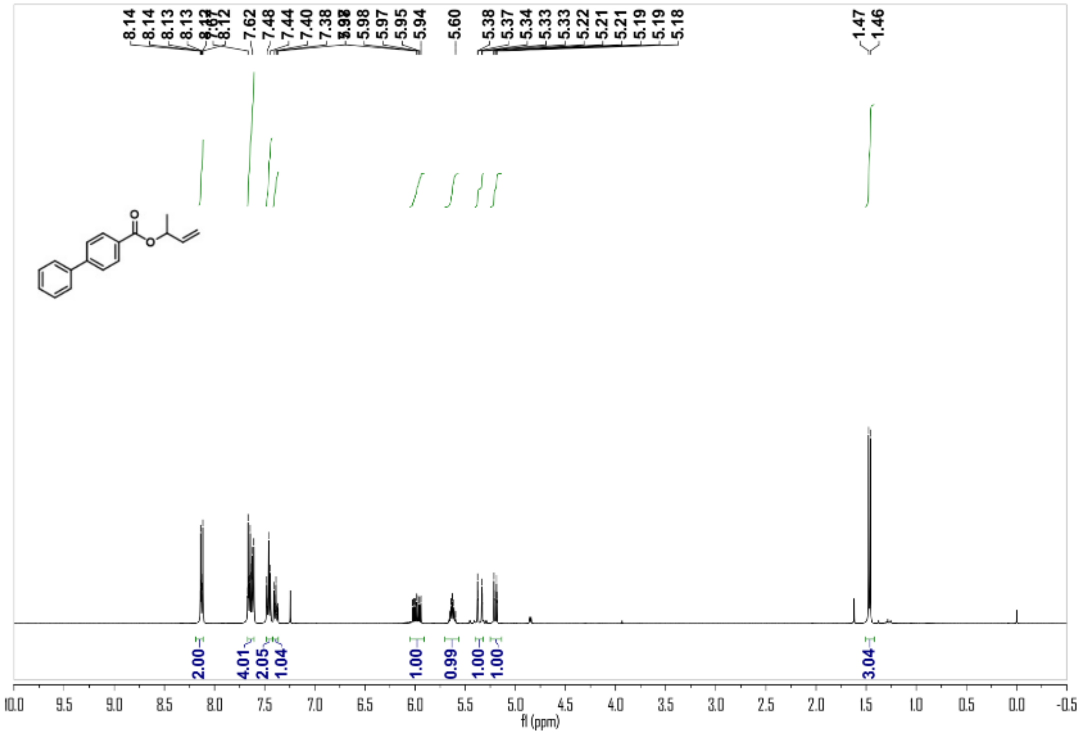

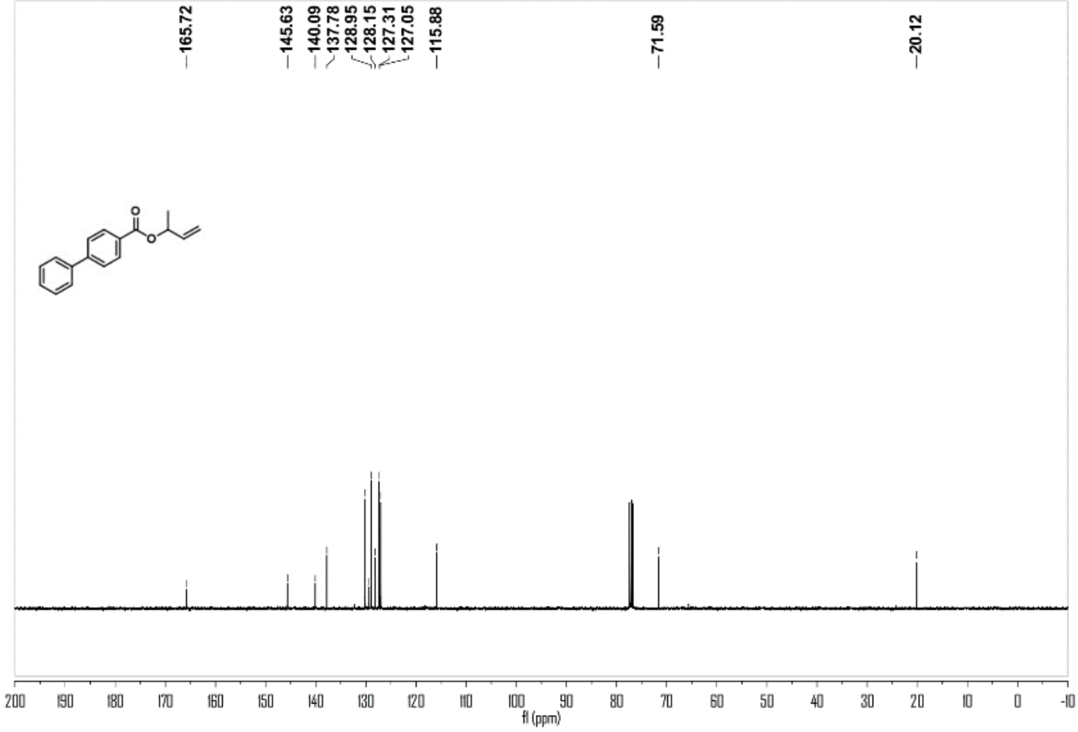

^{1}H and ^{13}C NMR spectra of compound 2aa

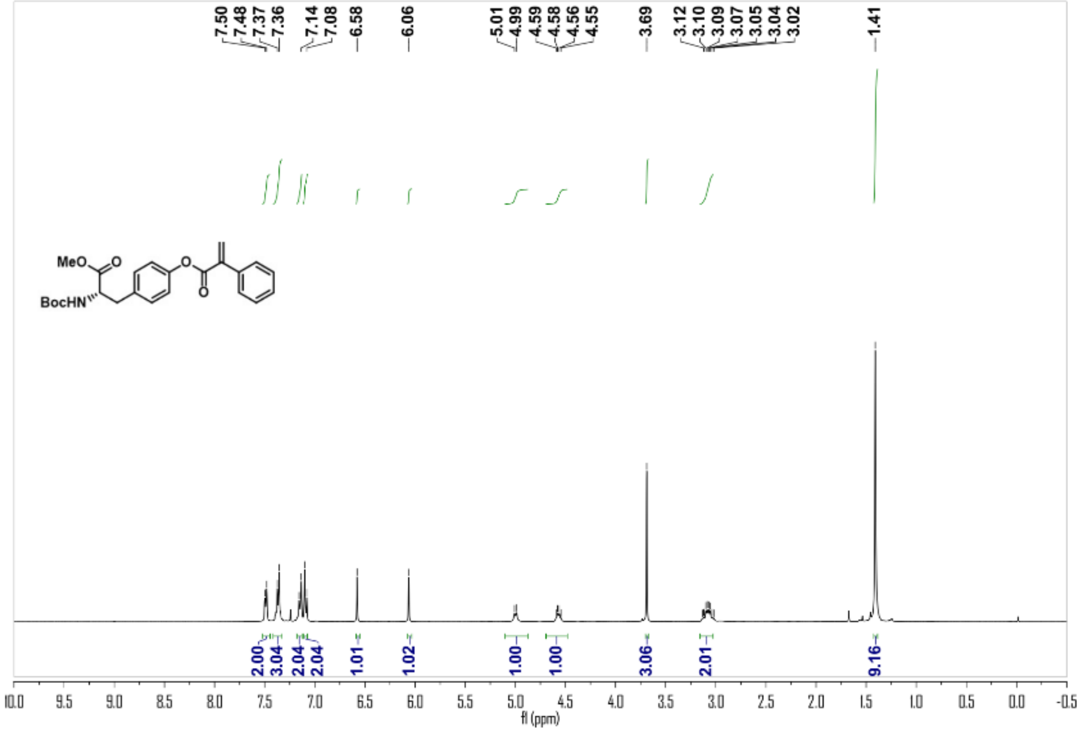

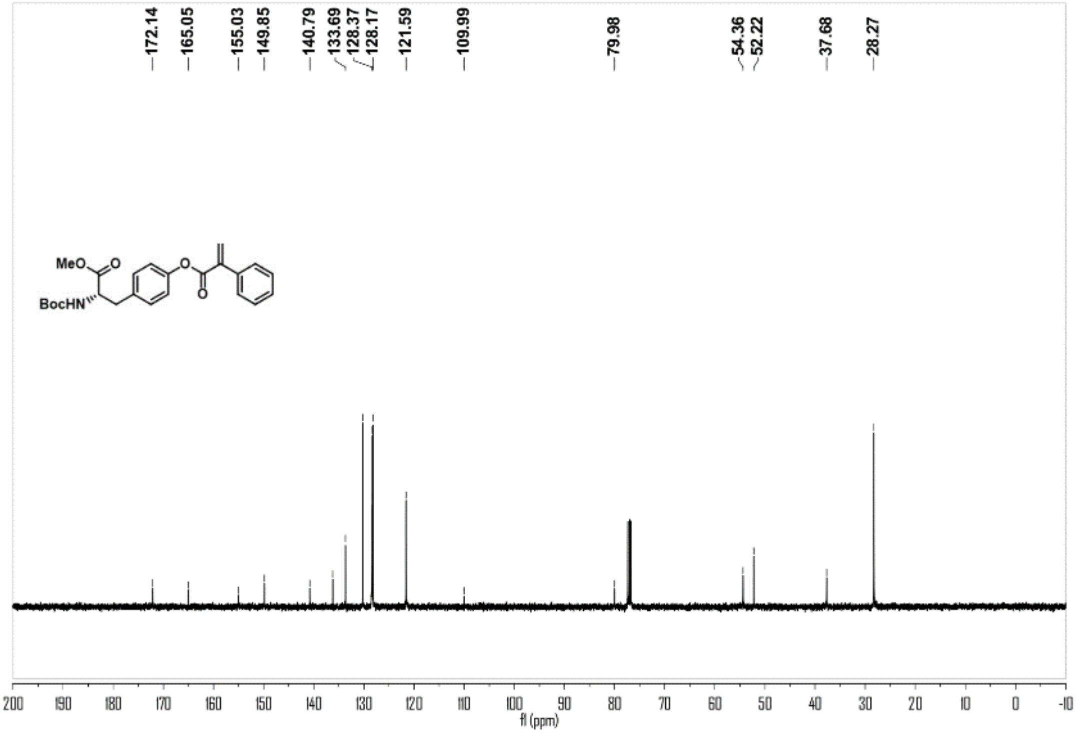

^{1}H and ^{13}C NMR spectra of compound 2ab

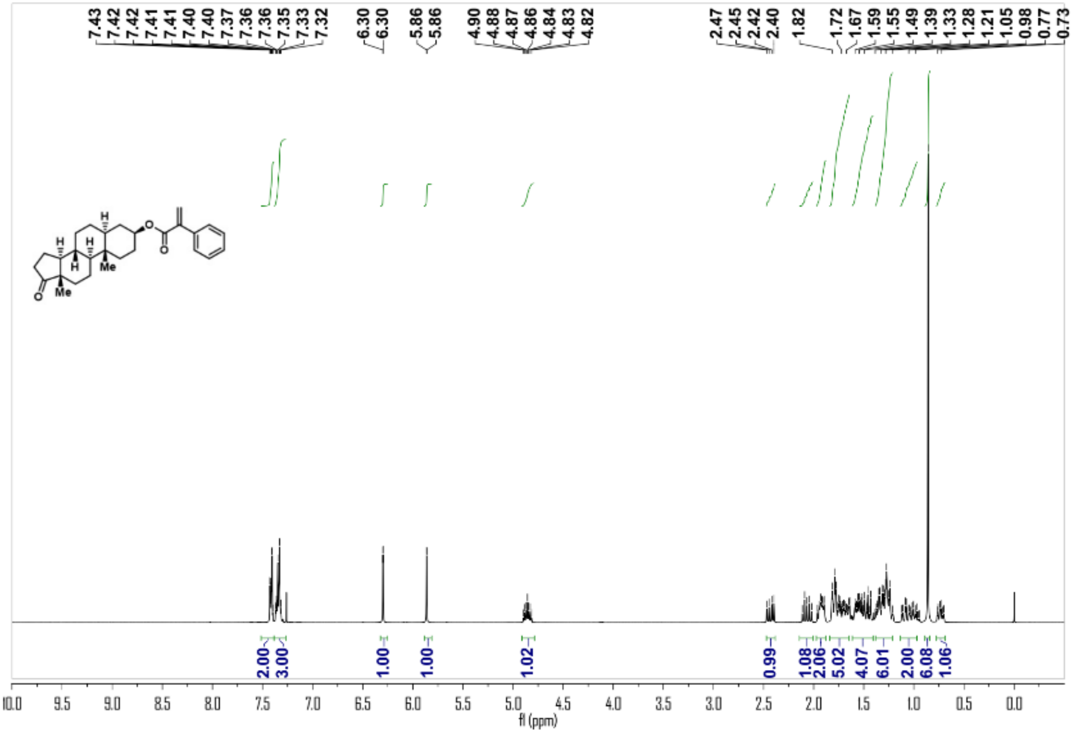

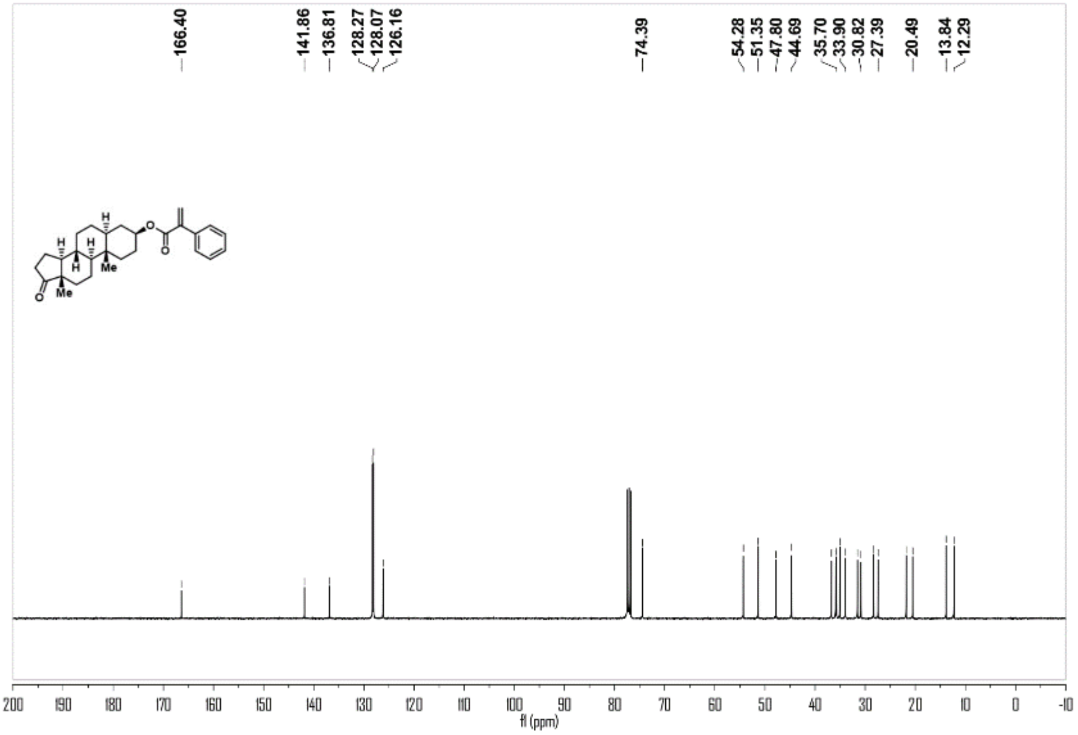

^{1}H and ^{13}C NMR spectra of compound 2ac

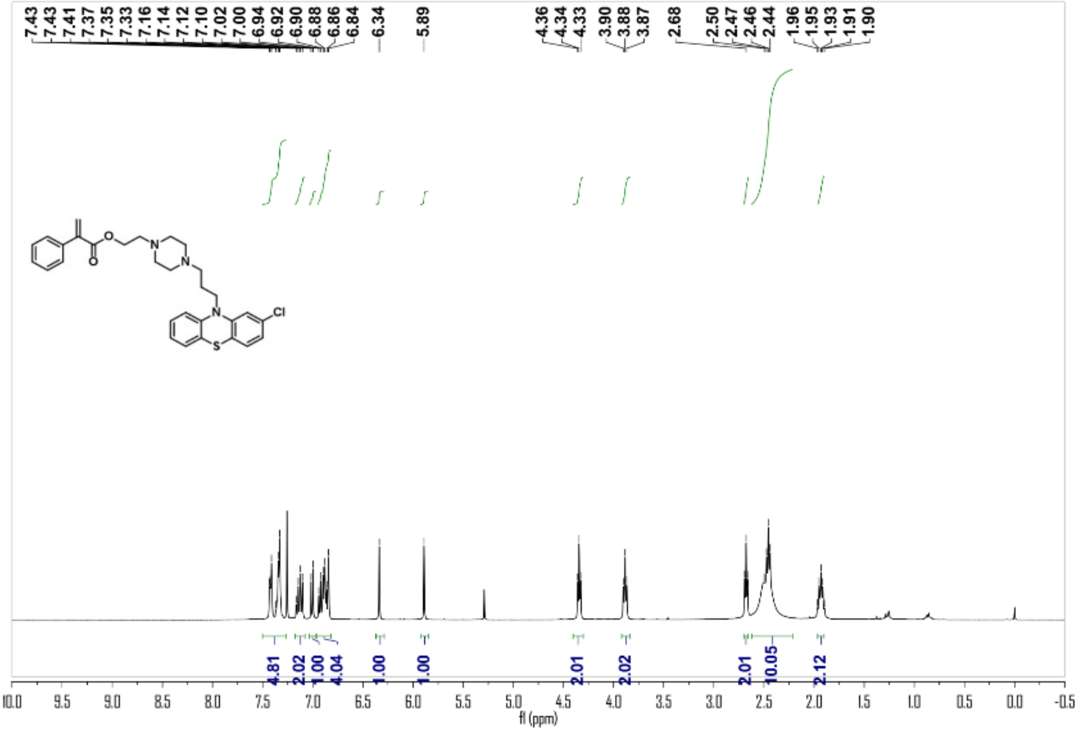

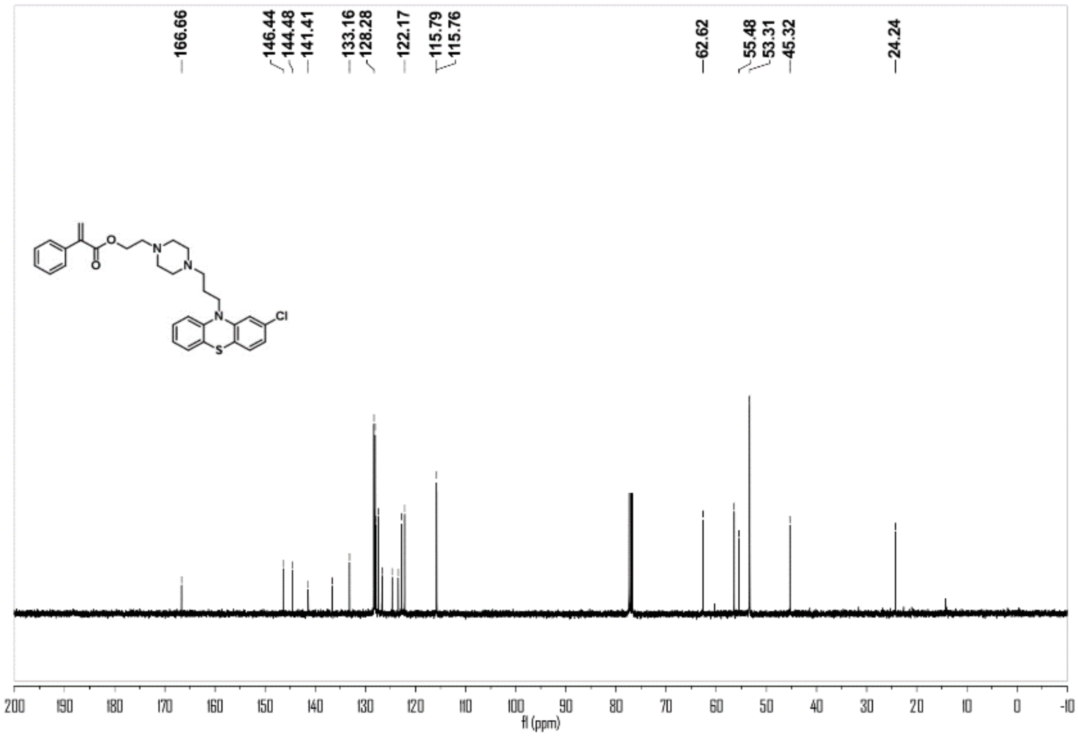

^{1}H and ^{13}C NMR spectra of compound 4j

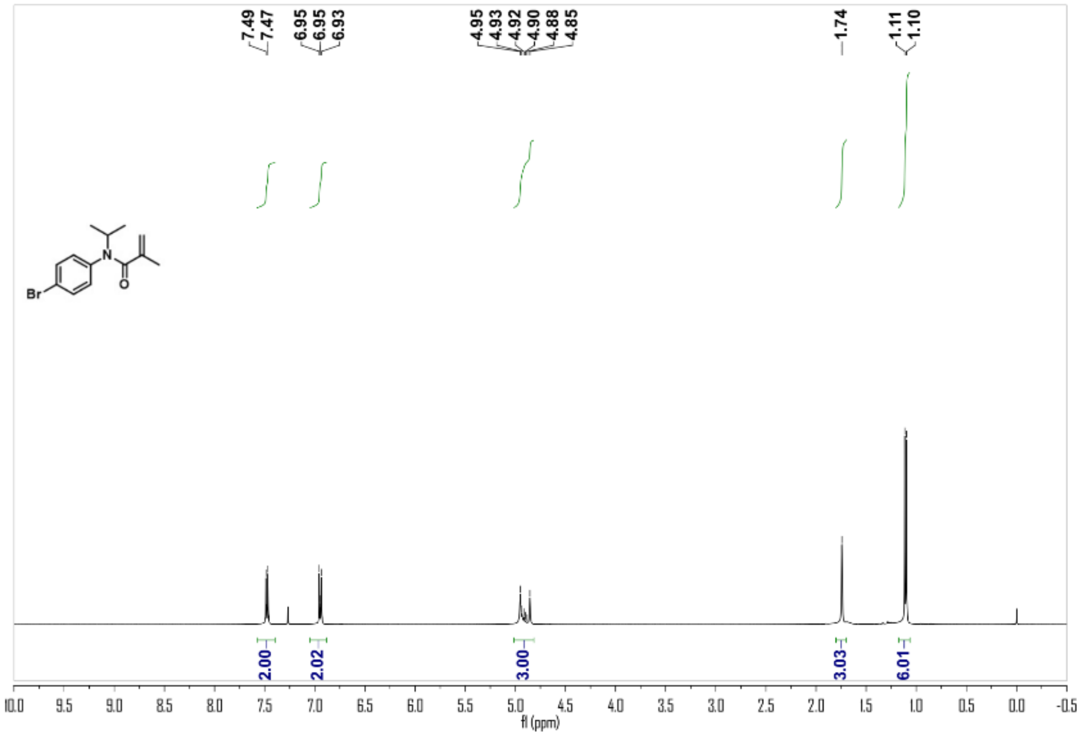

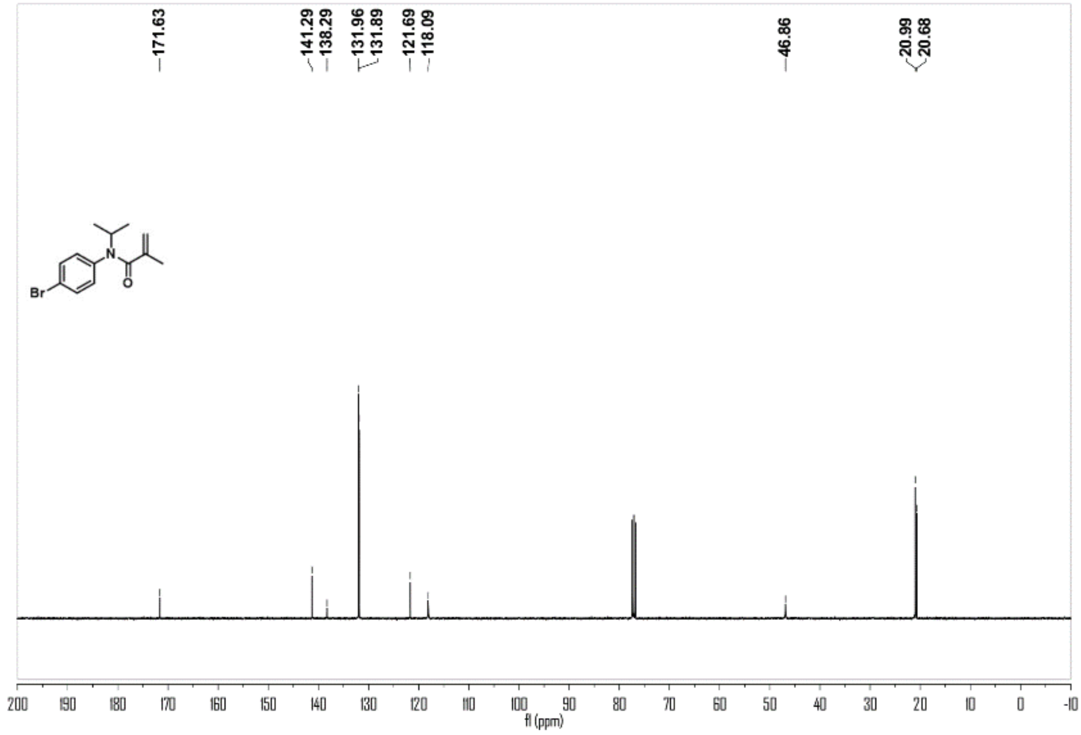

^{1}H and ^{13}C NMR spectra of compound 4n

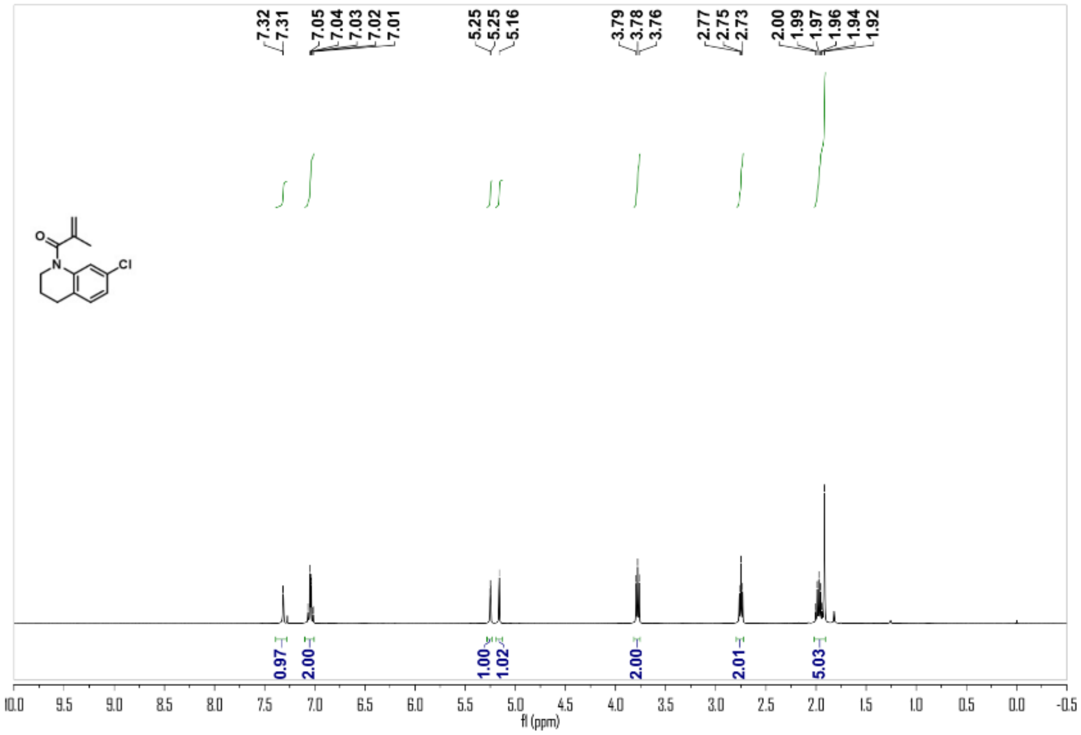

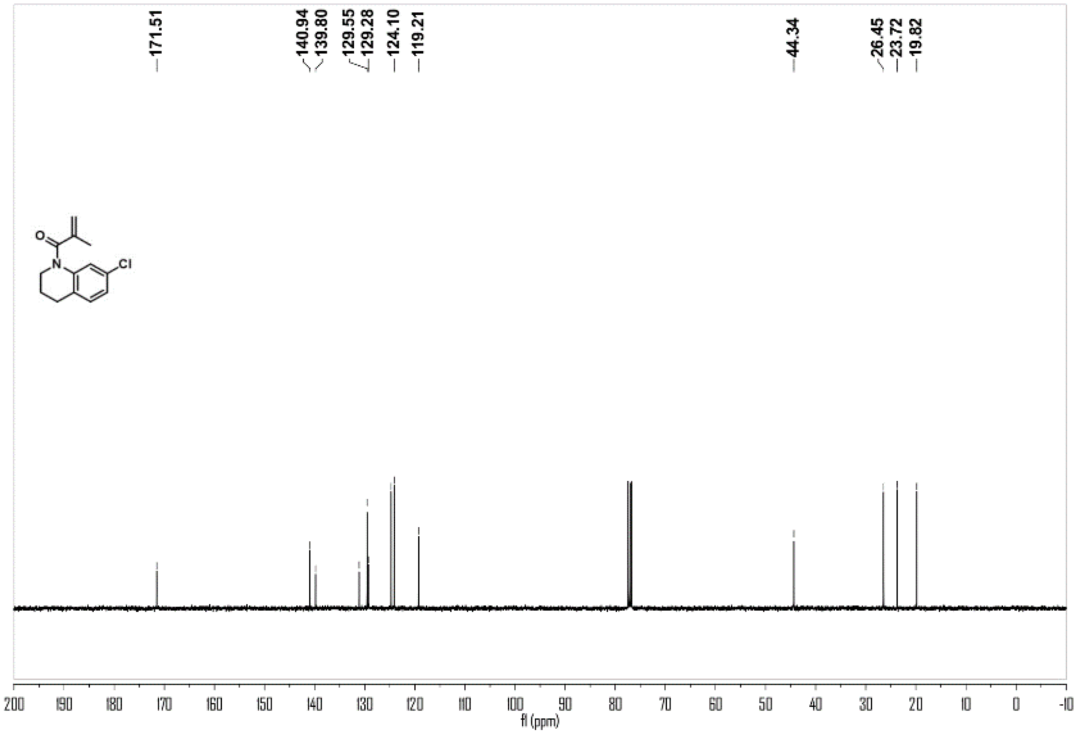

^{1}H and ^{13}C NMR spectra of compound 4x

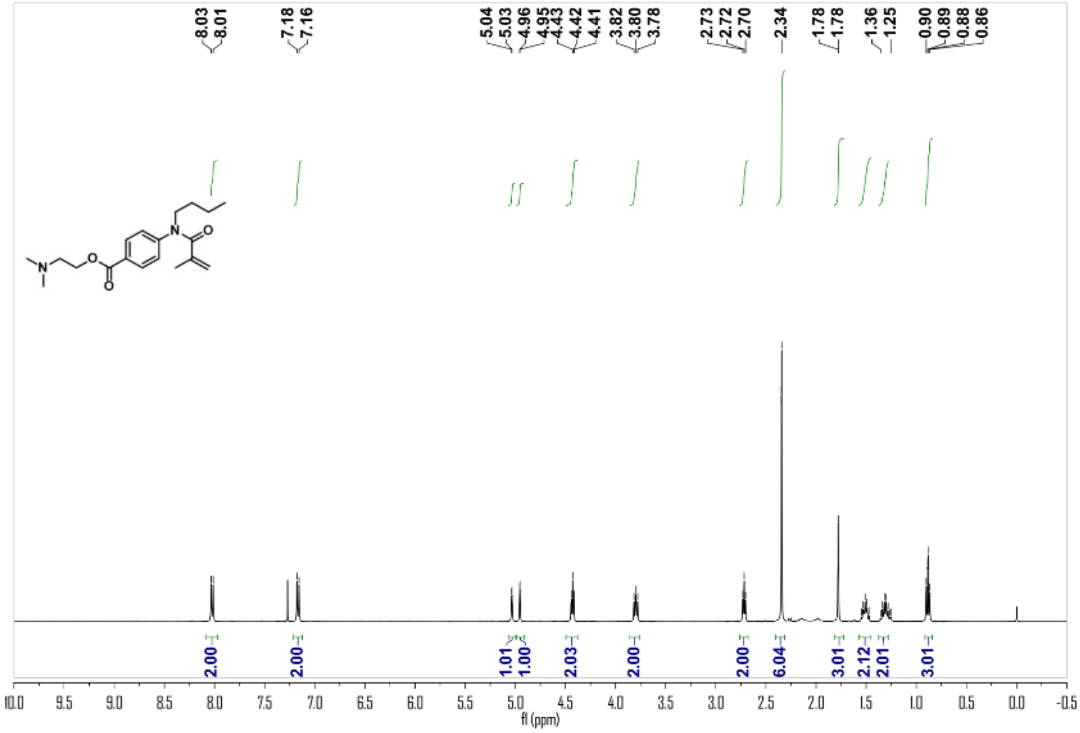

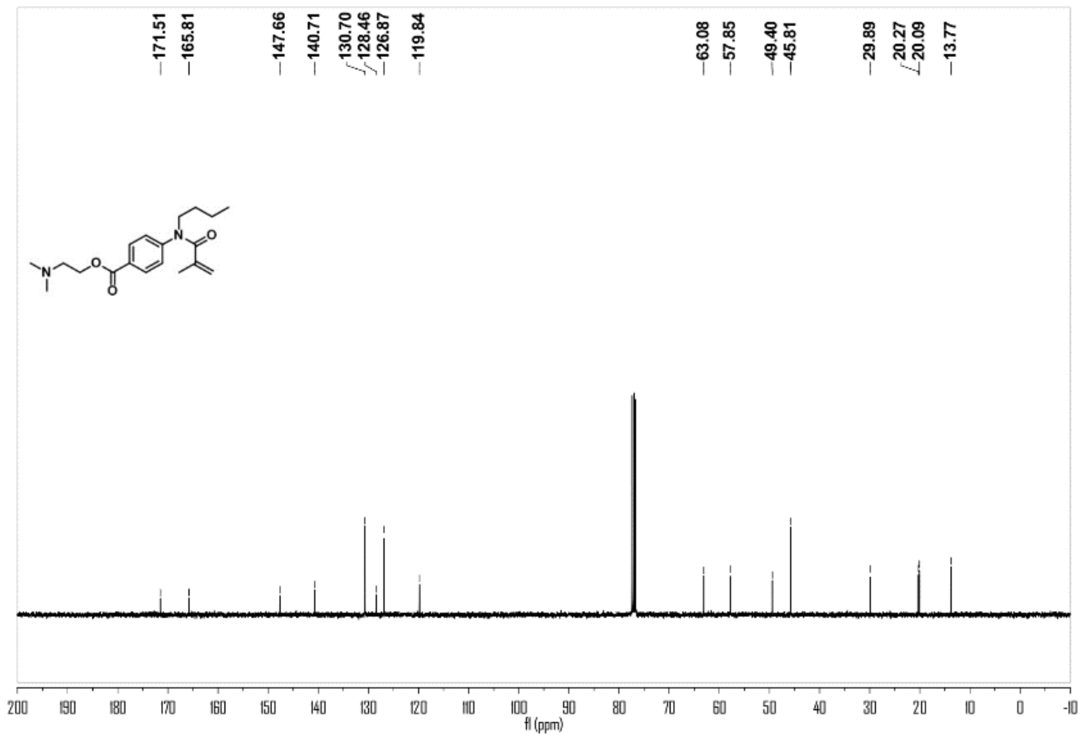

^1H, ^{19}F and ^{13}C NMR spectra of compound PC-3

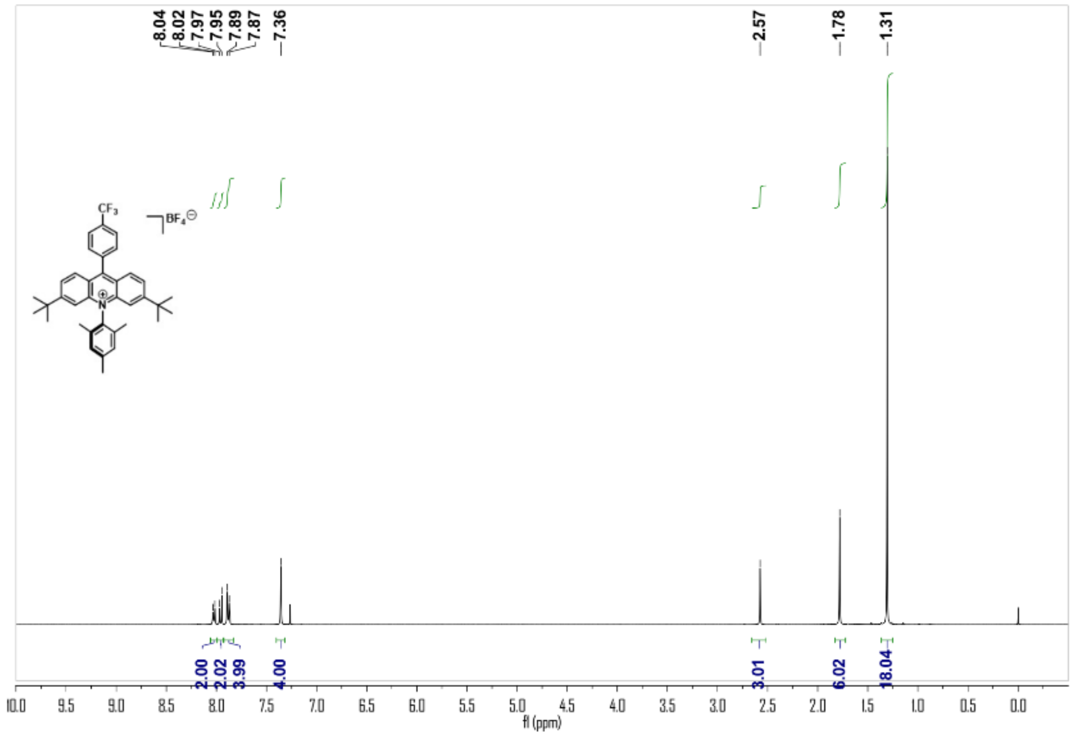

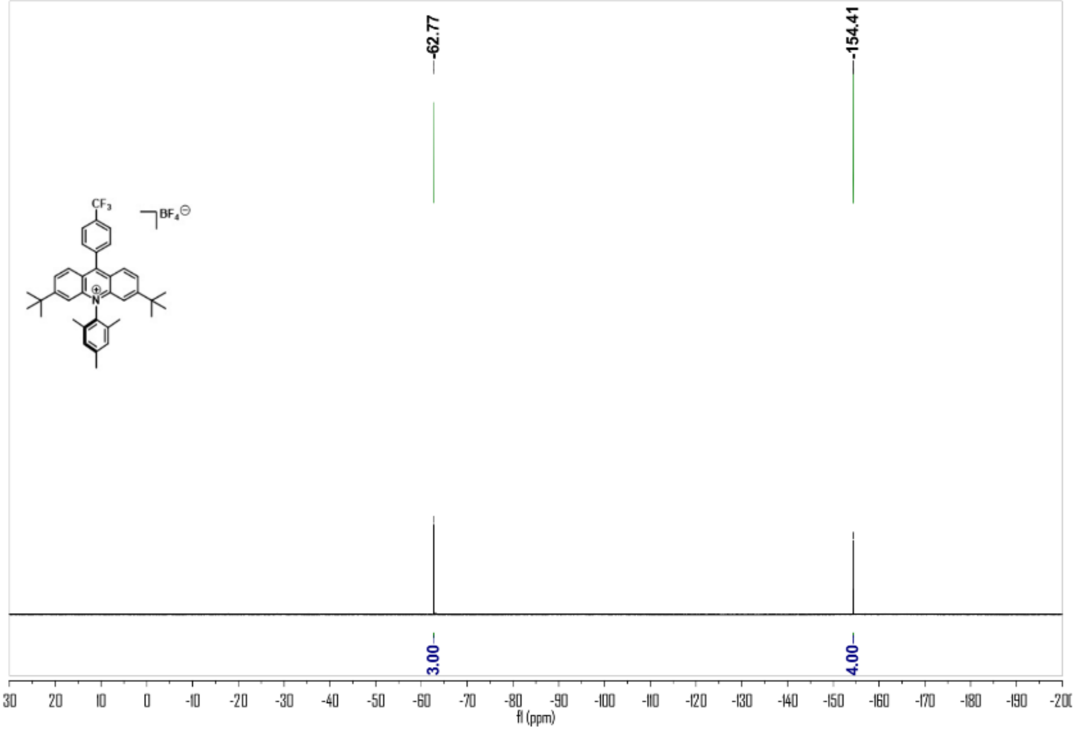
-62.77
-154.41
CF3
BF4
N
3.00
4.00
30 20 10 0 -10 -20 -30 -40 -50 -60 -70 -80 -90 -100 -110 -120 -130 -140 -150 -160 -170 -180 -190 -200
f1 (ppm)

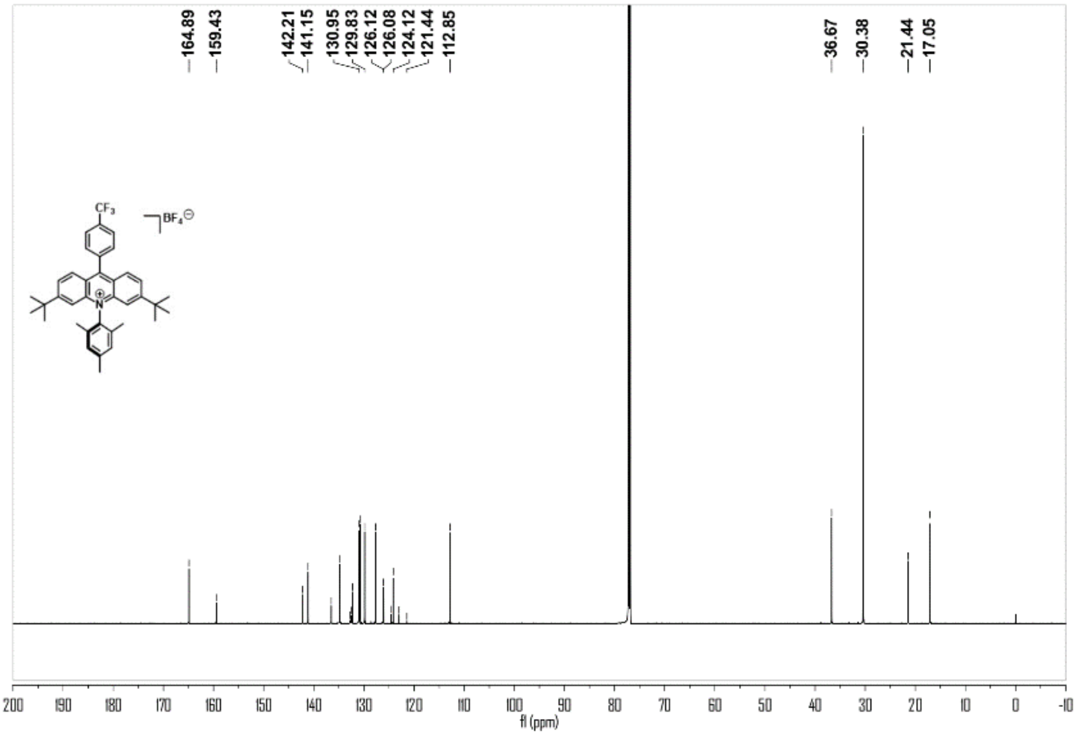
164.89
159.43
142.21
141.15
130.95
129.83
126.12
126.08
124.12
121.44
112.85
36.67
30.38
21.44
17.05
CF3
BF4
N
200 190 180 170 160 150 140 130 120 110 100 90 80 70 60 50 40 30 20 10 0 -10
f1 (ppm)

^{1}H, ^{19}F and ^{13}C NMR spectra of compound PC-4

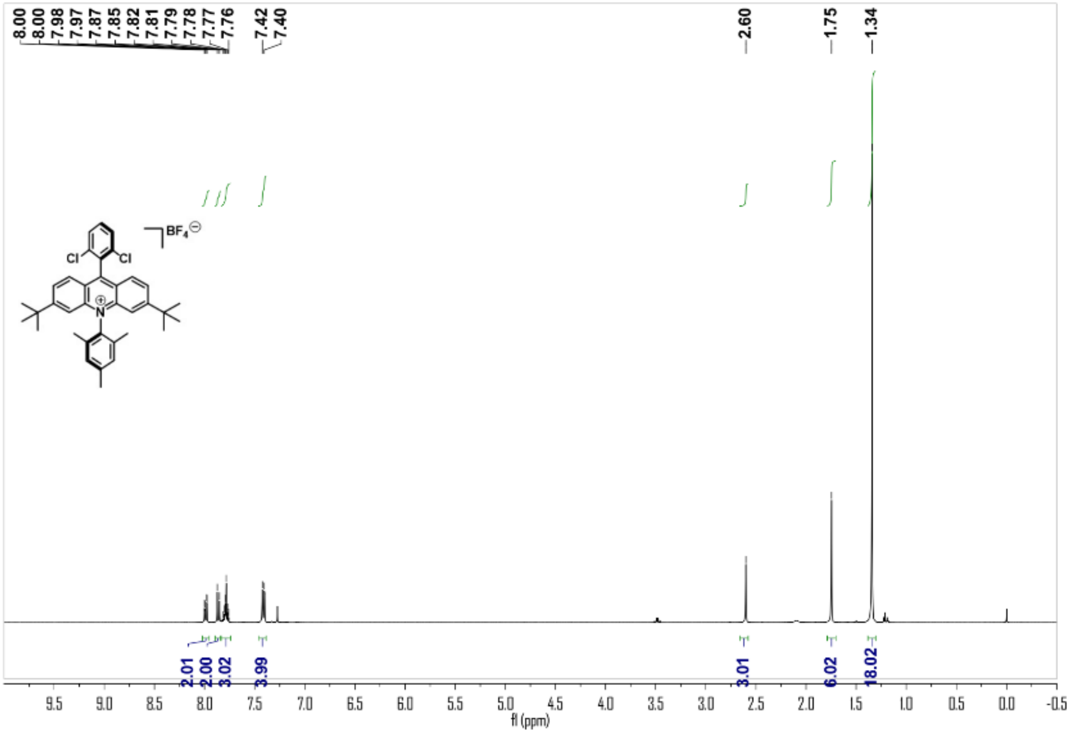

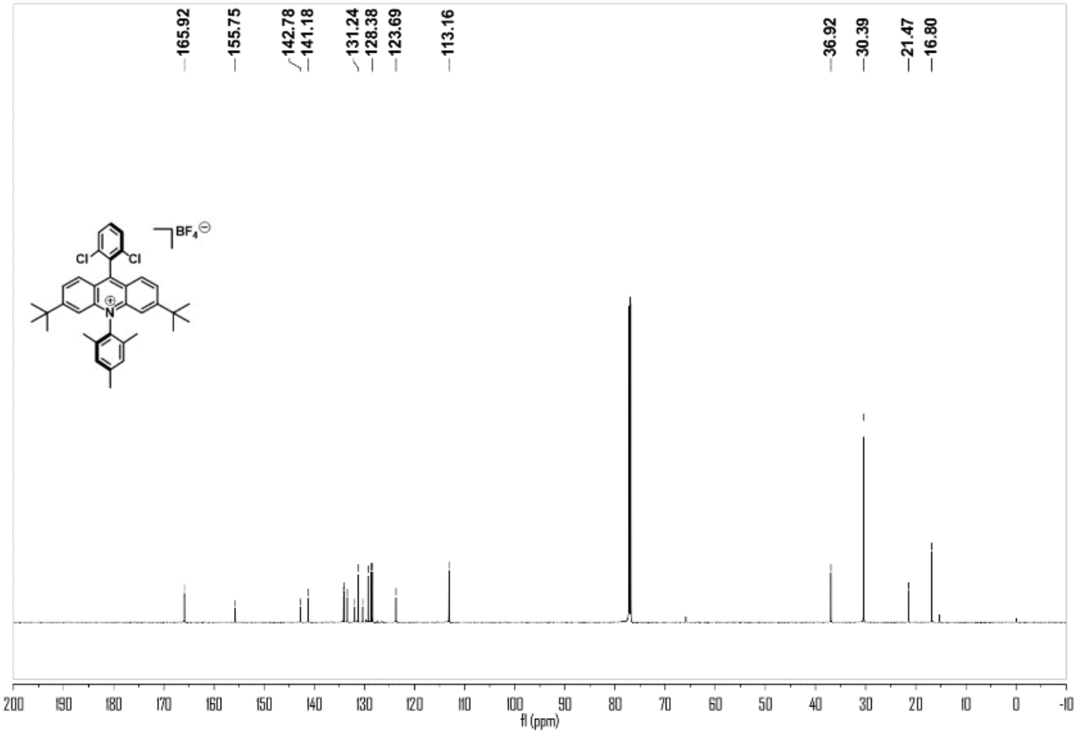

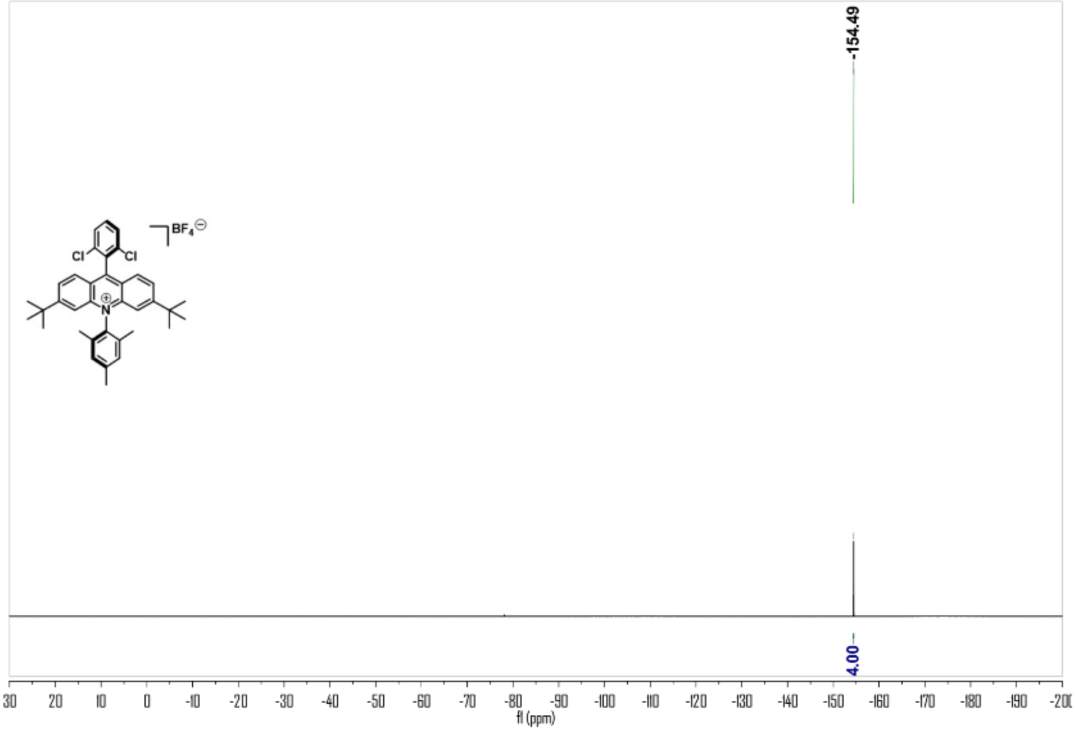

^{1}H, ^{19}F and ^{13}C NMR spectra of compound PC-5

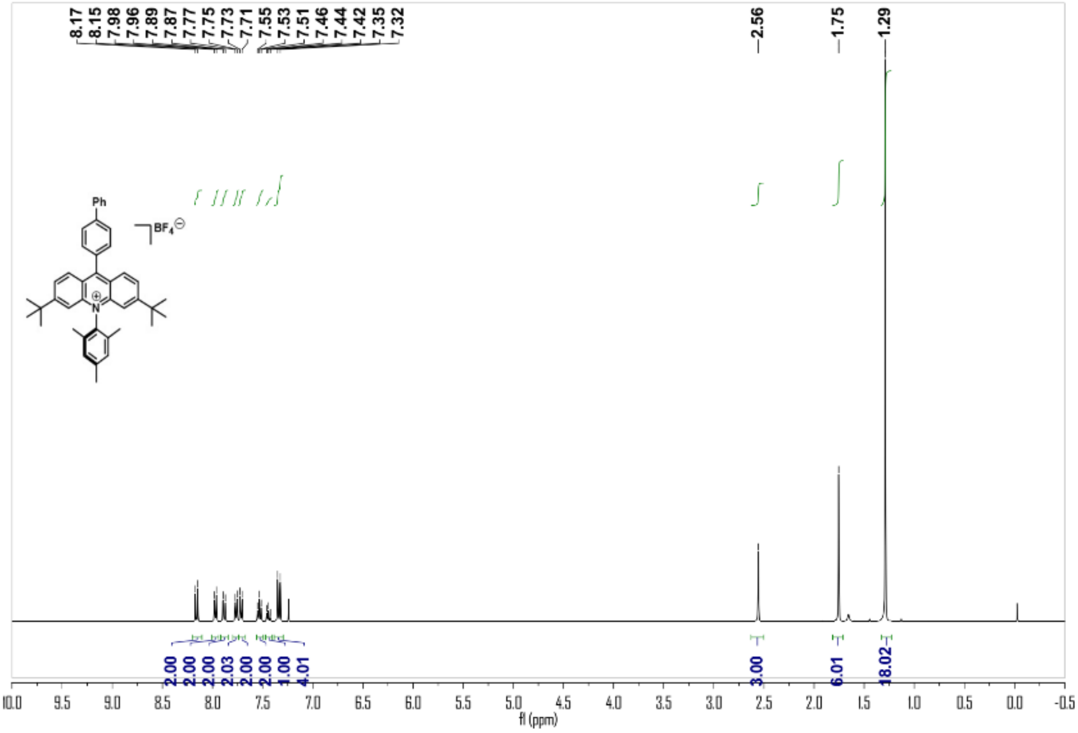

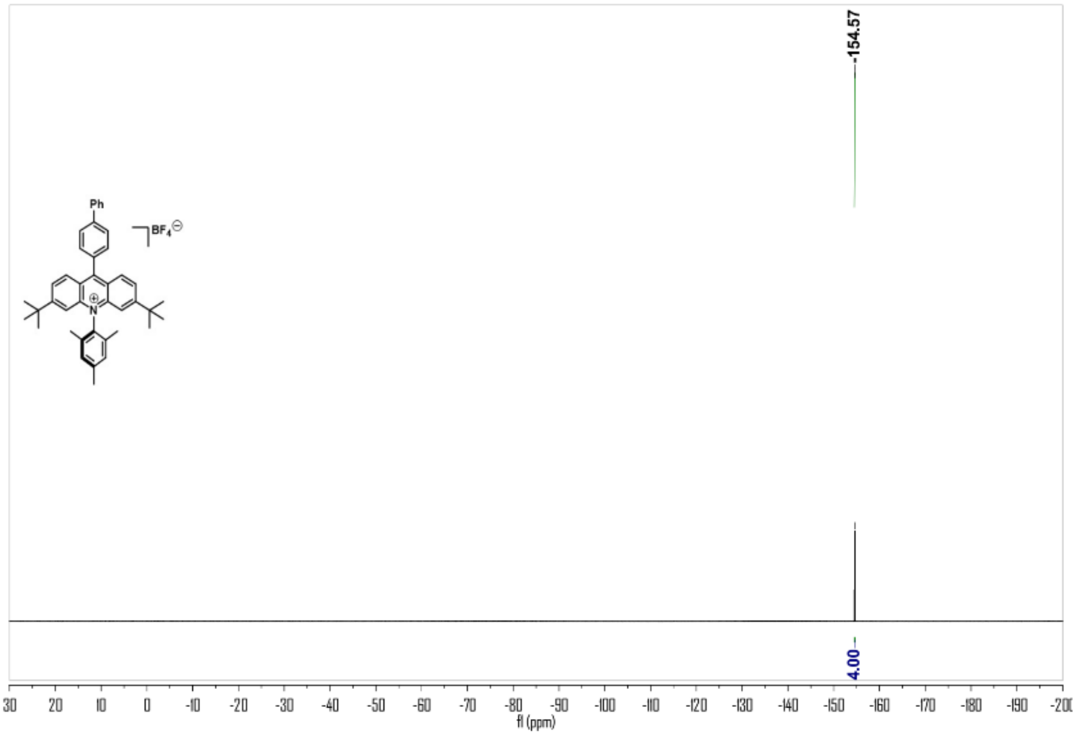

-154.57
Ph
BF4
4.00
fl (ppm)

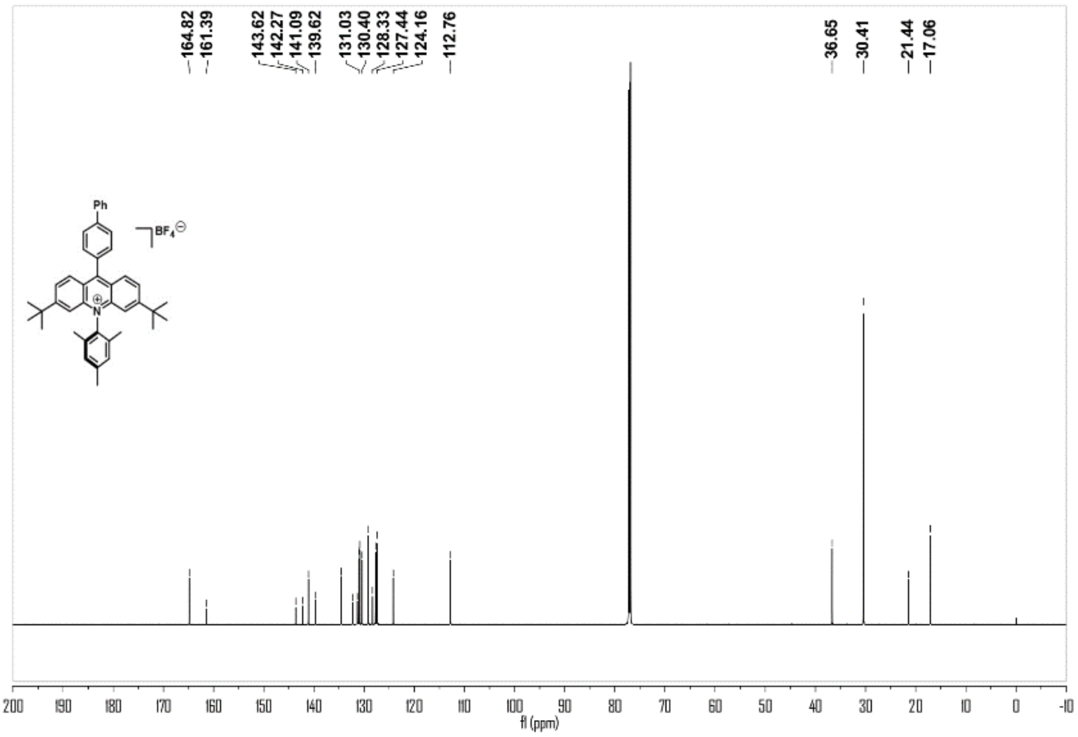

164.82
161.39
143.62
142.27
141.09
139.62
131.03
130.40
128.33
127.44
124.16
112.76
36.65
30.41
21.44
17.06
Ph
BF4
fl (ppm)

^{1}H, ^{19}F and ^{13}C NMR spectra of compound PC-6

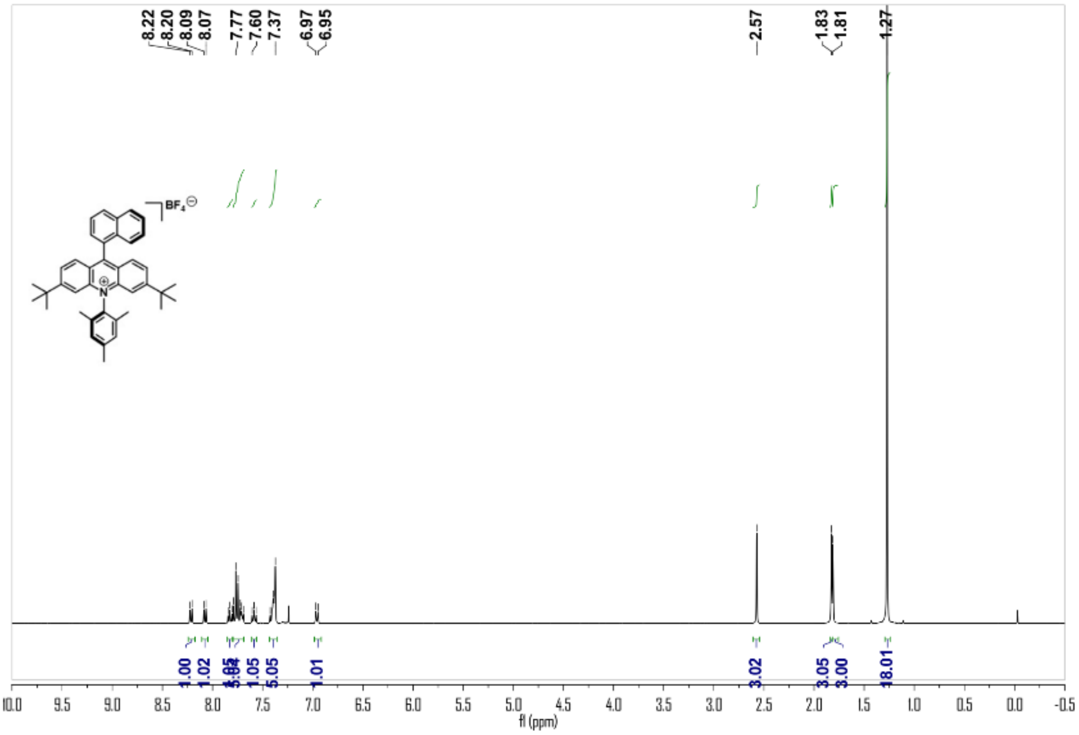

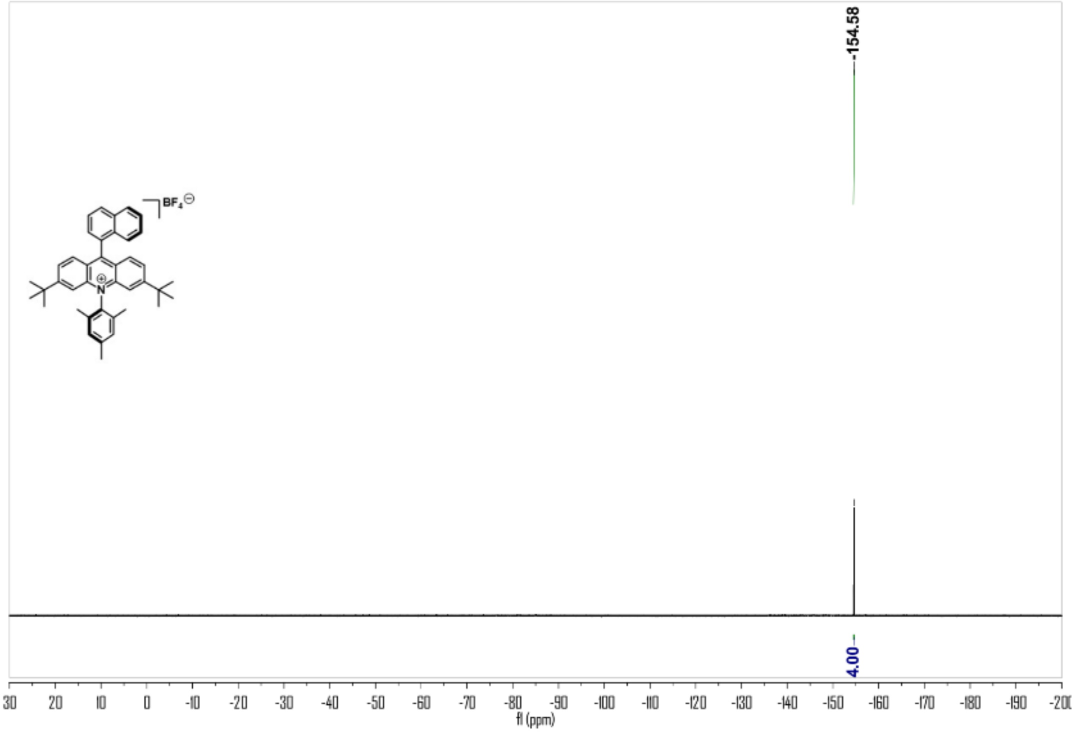

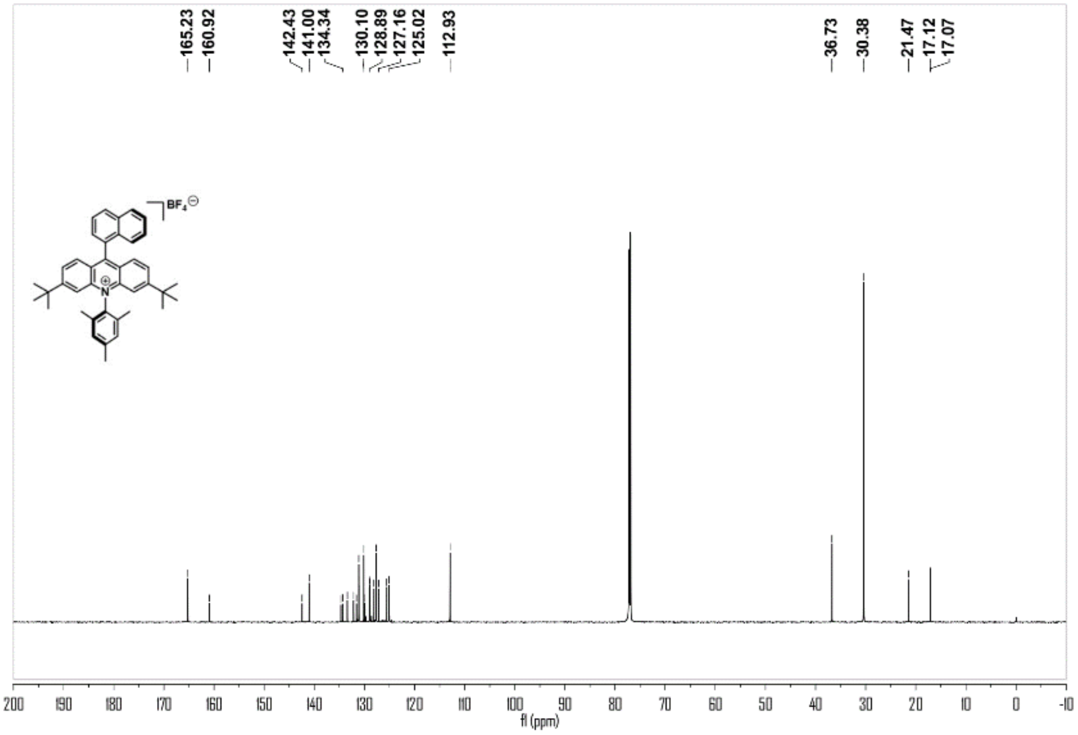

^{1}H, ^{19}F and ^{13}C NMR spectra of compound 3a

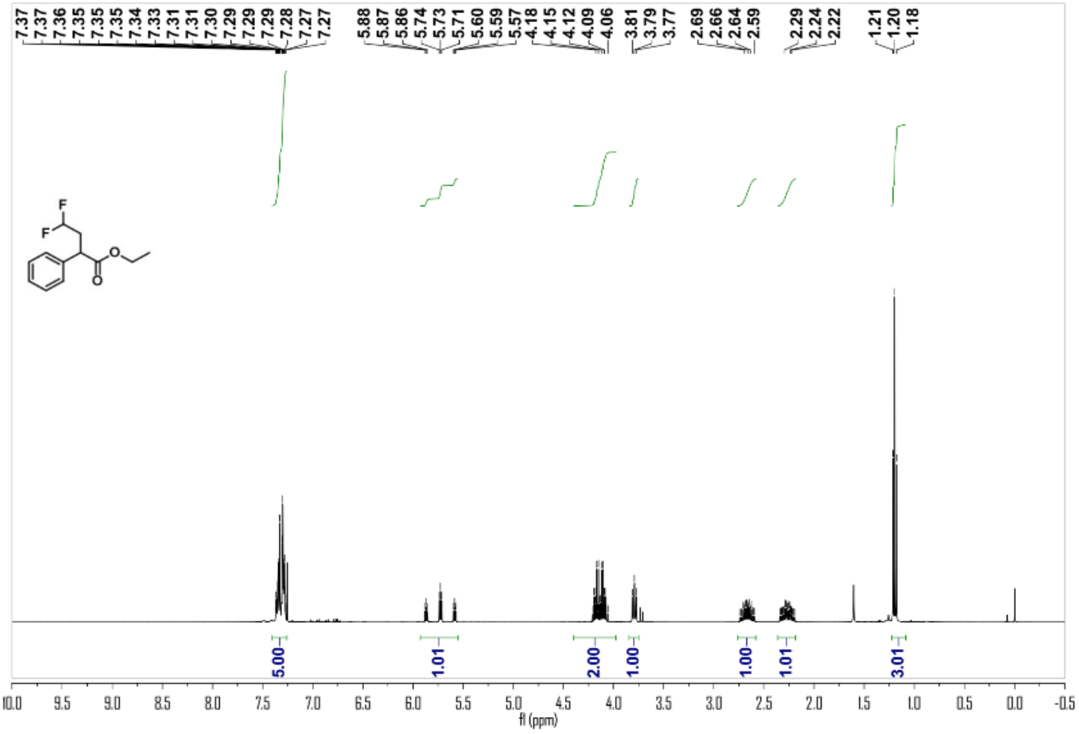

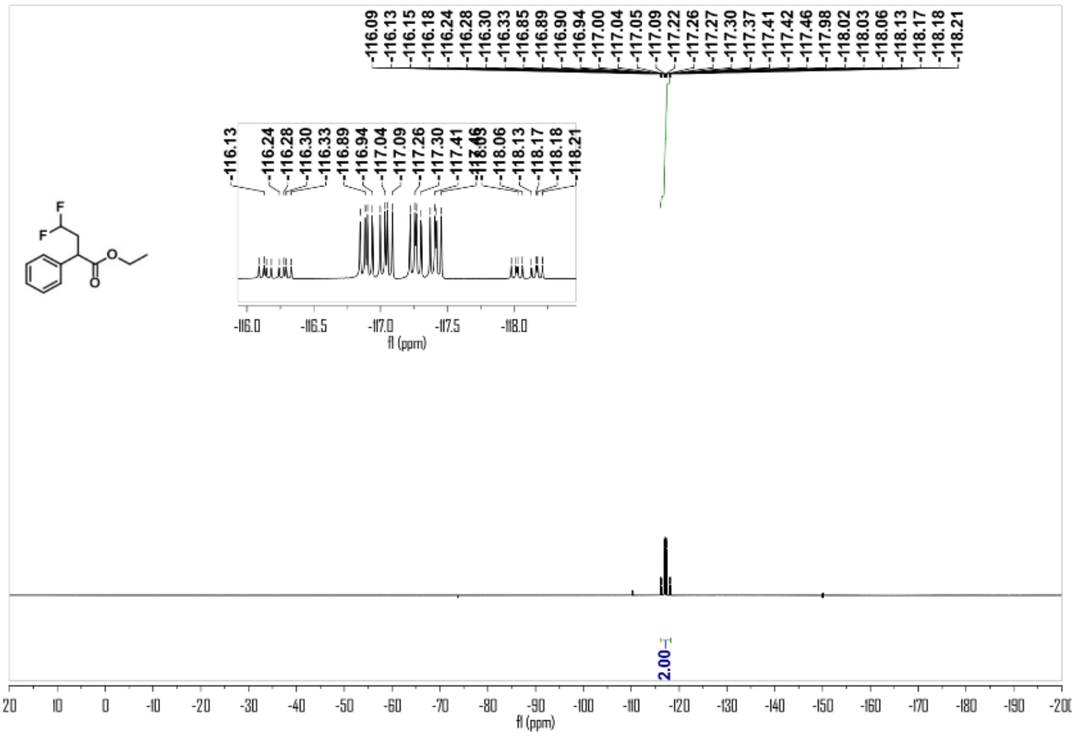
-116.09
-116.13
-116.15
-116.18
-116.24
-116.28
-116.30
-116.33
-116.85
-116.89
-116.90
-116.94
-117.00
-117.04
-117.05
-117.09
-117.22
-117.26
-117.27
-117.30
-117.37
-117.41
-117.42
-117.46
-117.98
-118.02
-118.03
-118.06
-118.13
-118.17
-118.18
-118.21
2.00
f1 (ppm)

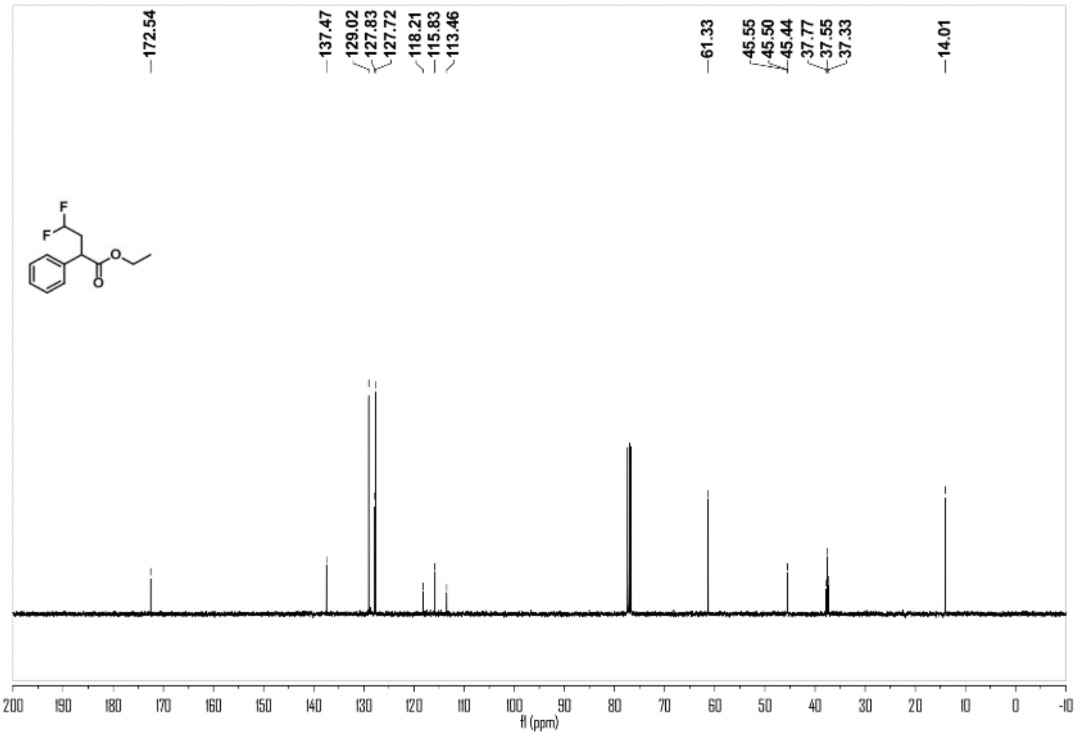
172.54
137.47
129.02
127.83
127.72
118.21
115.83
113.46
61.33
45.55
45.50
45.44
37.77
37.55
37.33
14.01
f1 (ppm)

^{1}H, ^{19}F and ^{13}C NMR spectra of compound 3b

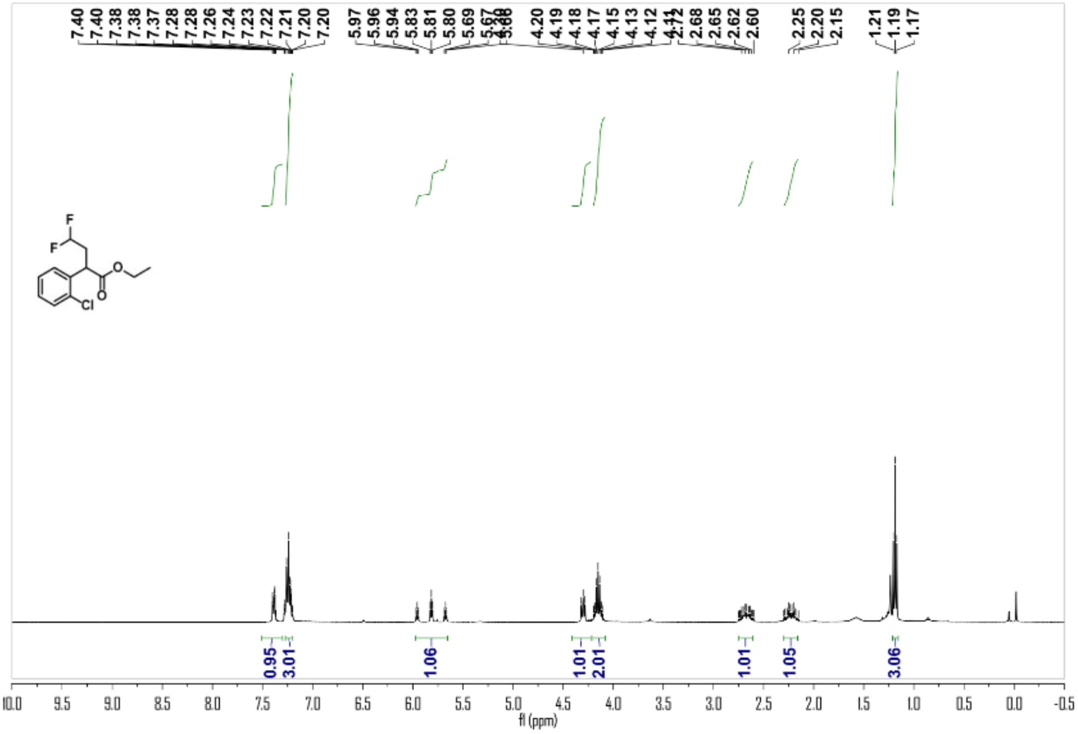

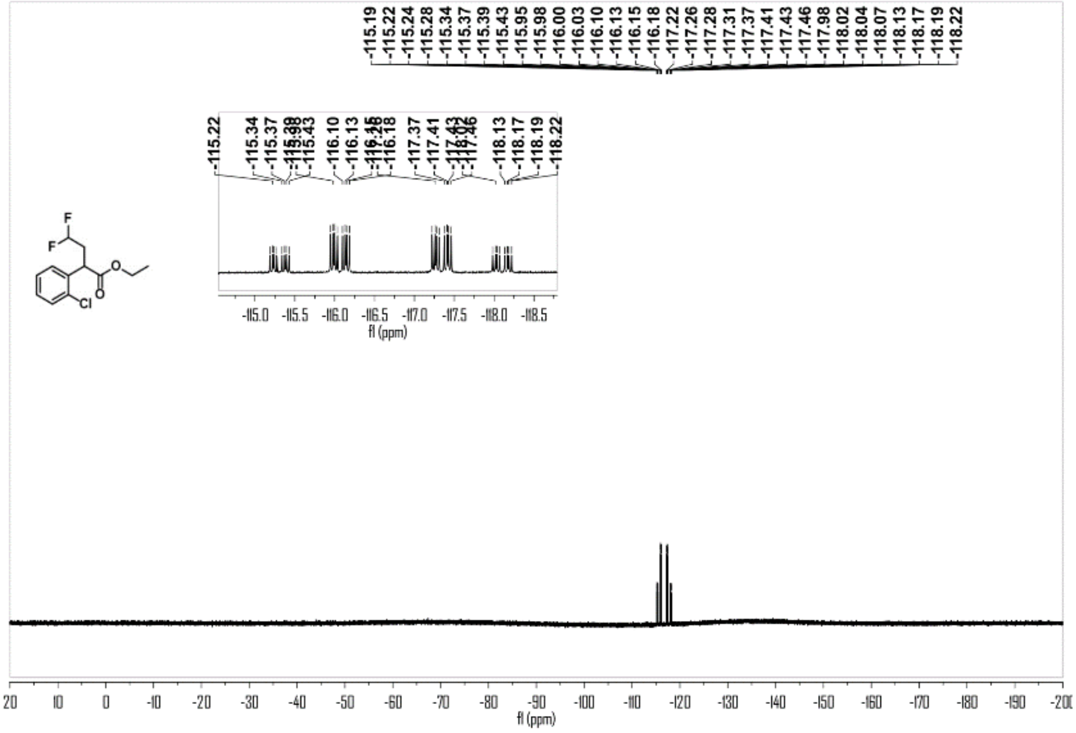

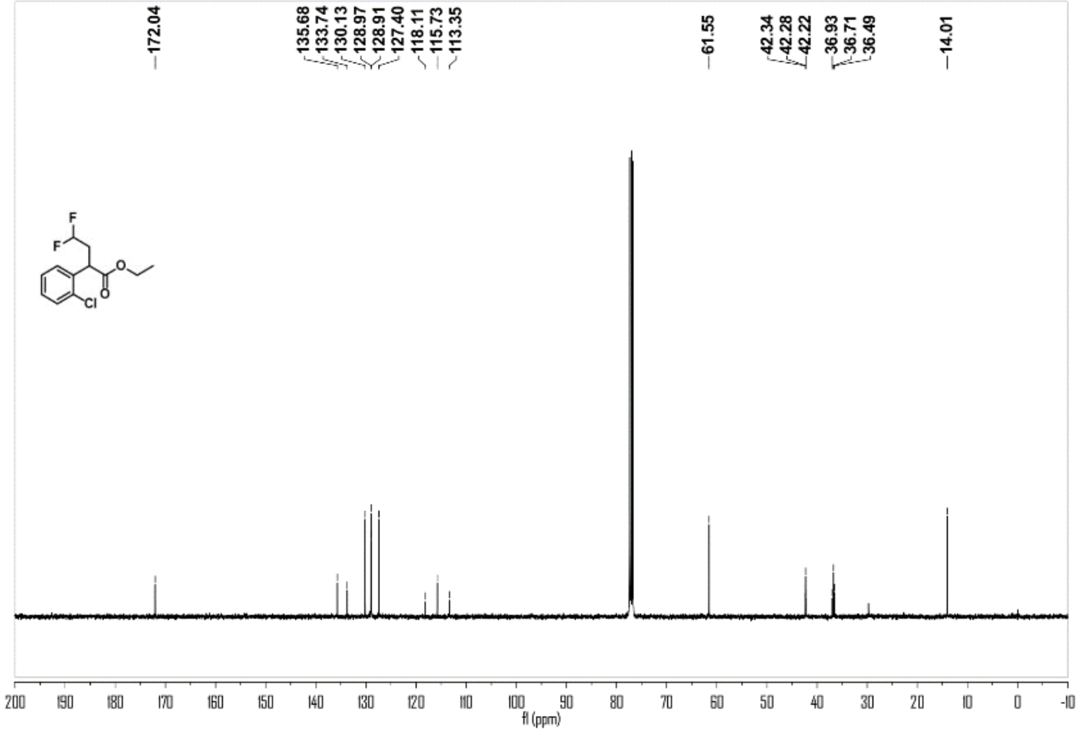

^{1}H, ^{19}F and ^{13}C NMR spectra of compound 3c

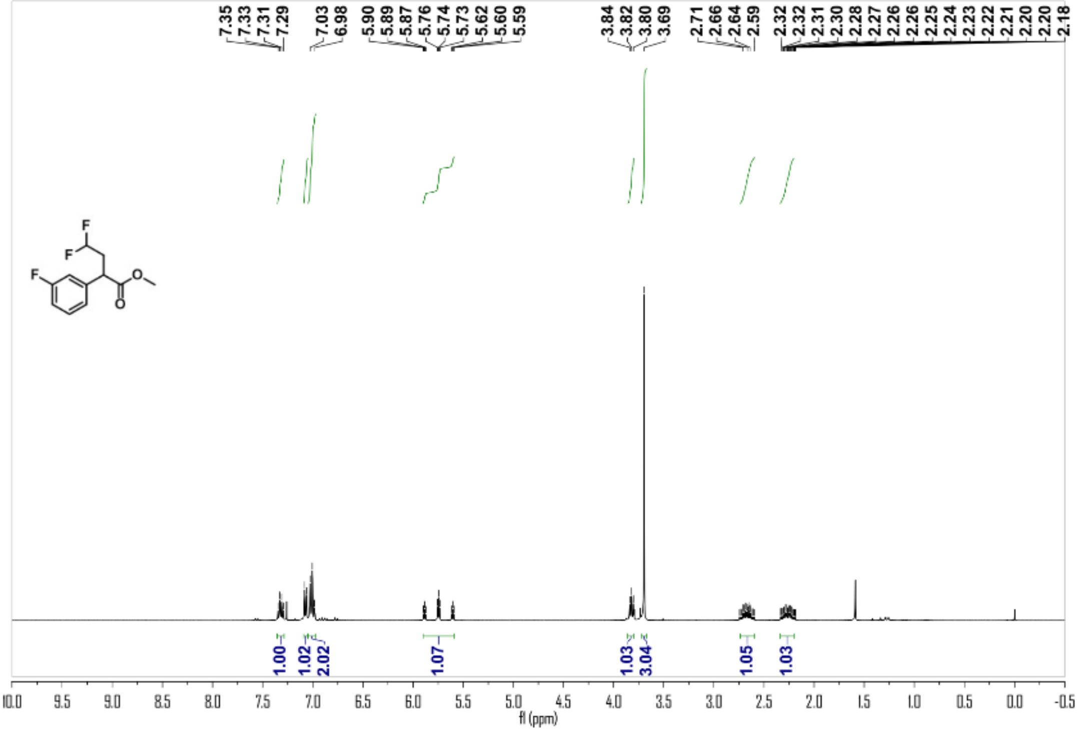

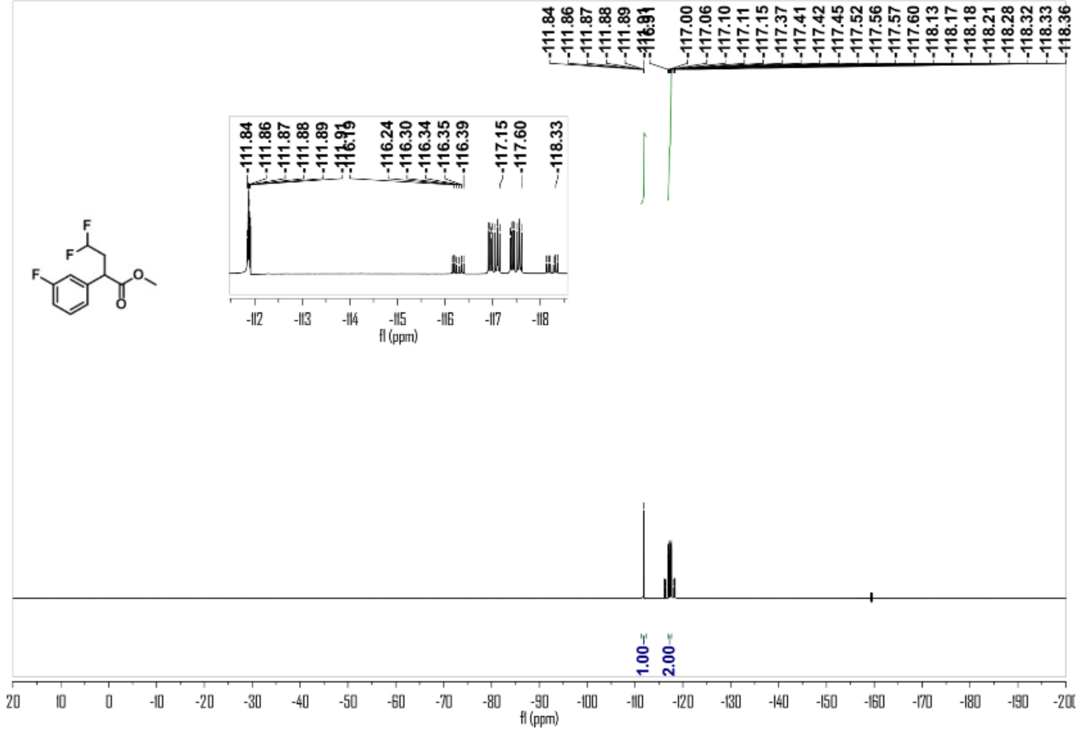

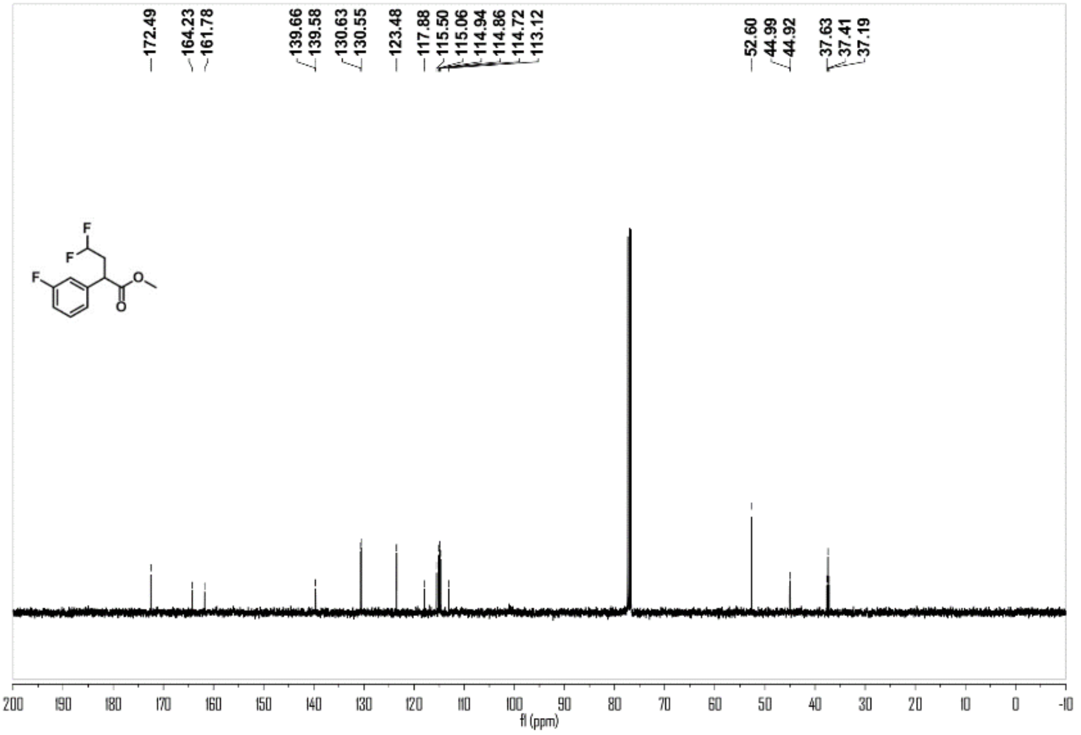

^{1}H, ^{19}F and ^{13}C NMR spectra of compound 3d

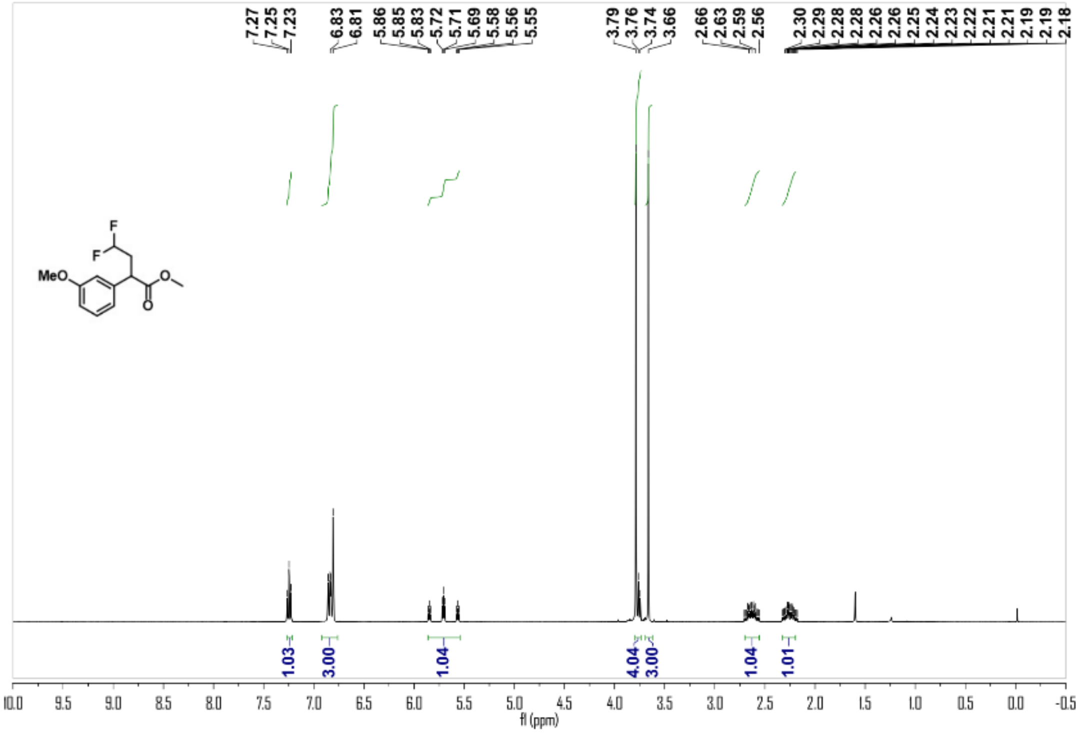

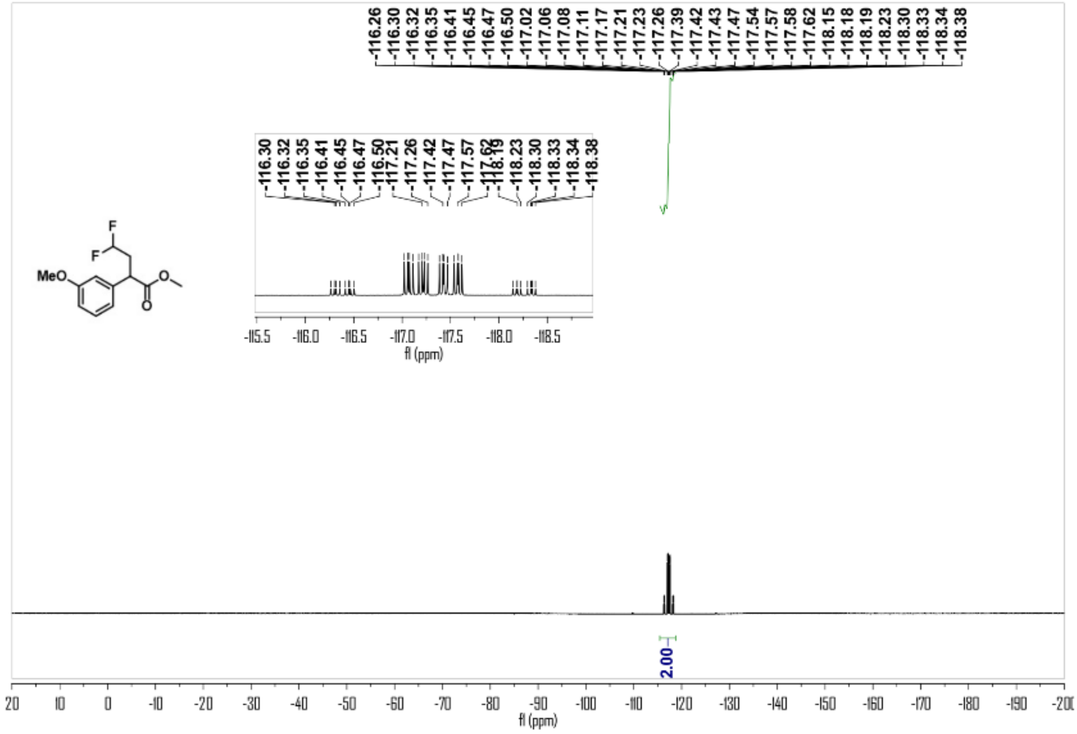

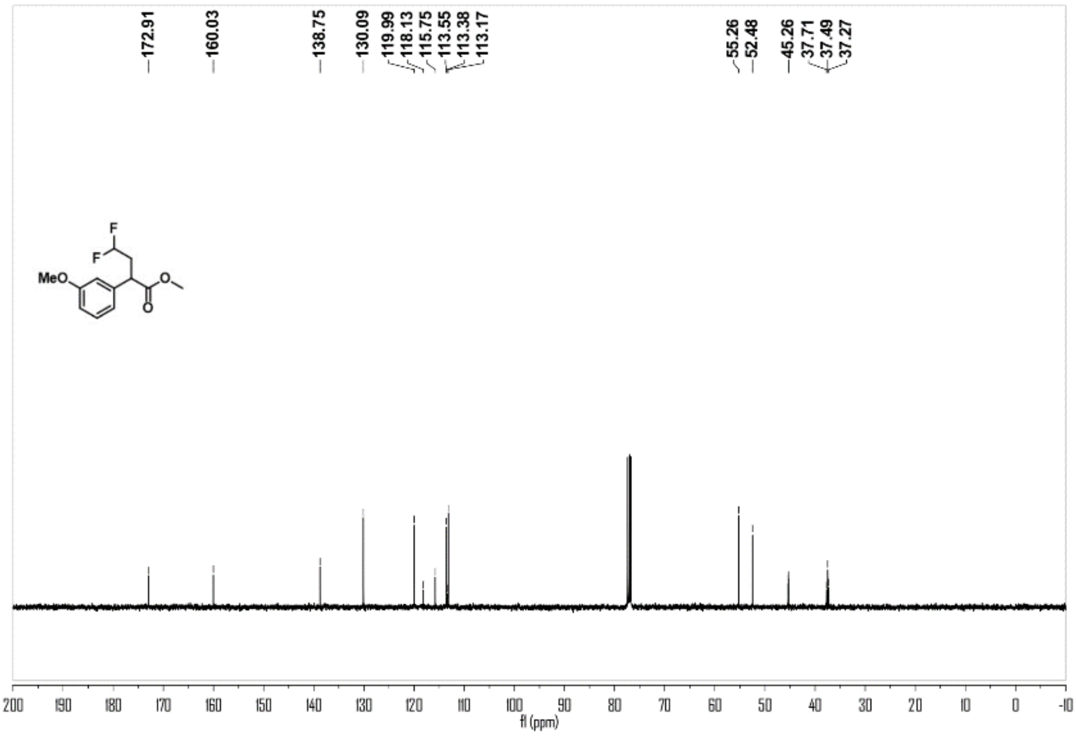

^{1}H, ^{19}F and ^{13}C NMR spectra of compound 3e

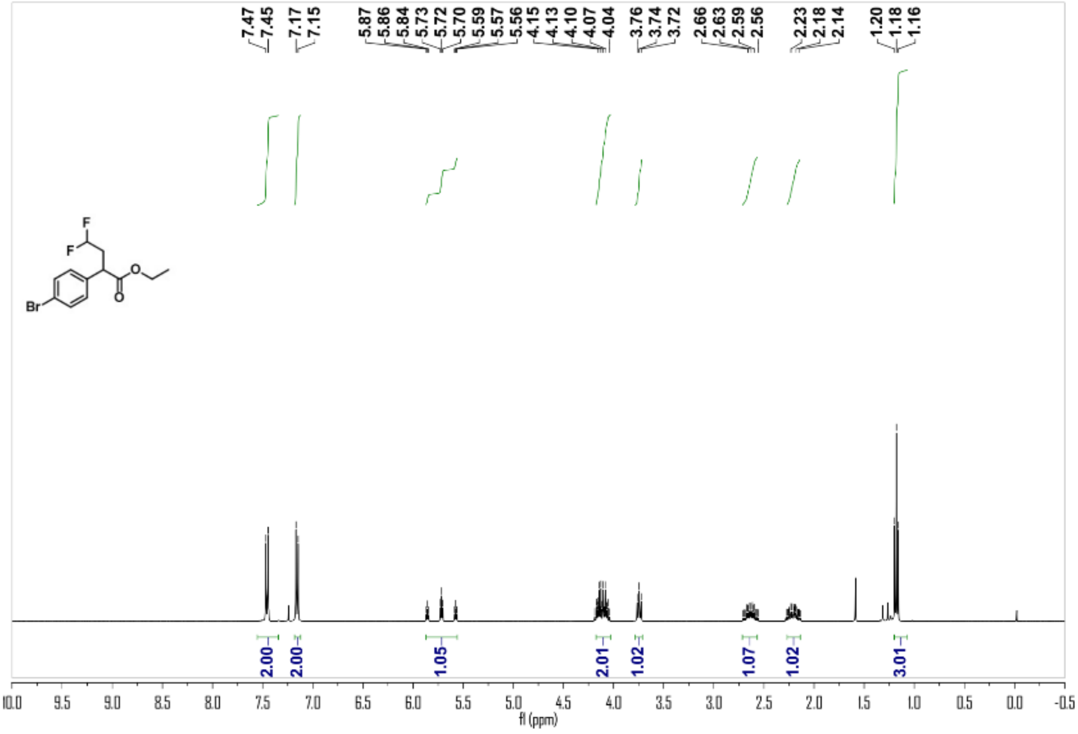

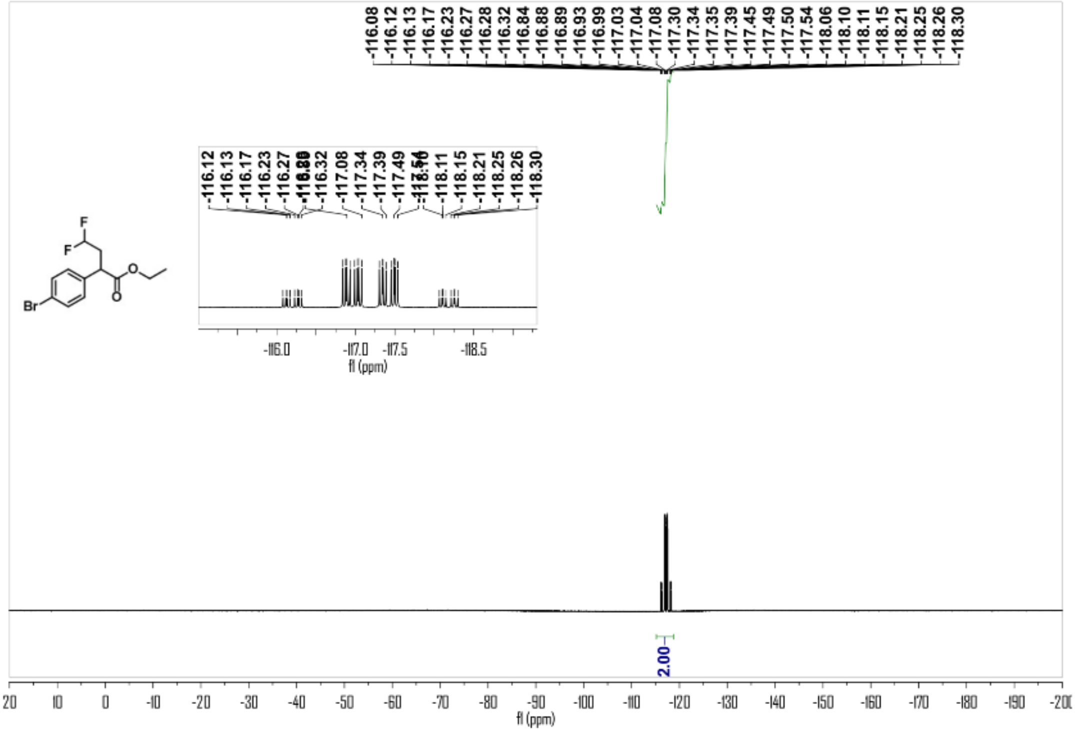
-116.08
-116.12
-116.13
-116.17
-116.23
-116.27
-116.28
-116.32
-116.84
-116.88
-116.89
-116.93
-116.99
-117.03
-117.04
-117.08
-117.30
-117.34
-117.35
-117.39
-117.45
-117.49
-117.50
-117.54
-118.06
-118.10
-118.11
-118.15
-118.21
-118.25
-118.26
-118.30
2.00
f1 (ppm)
Br
F
F
O
O

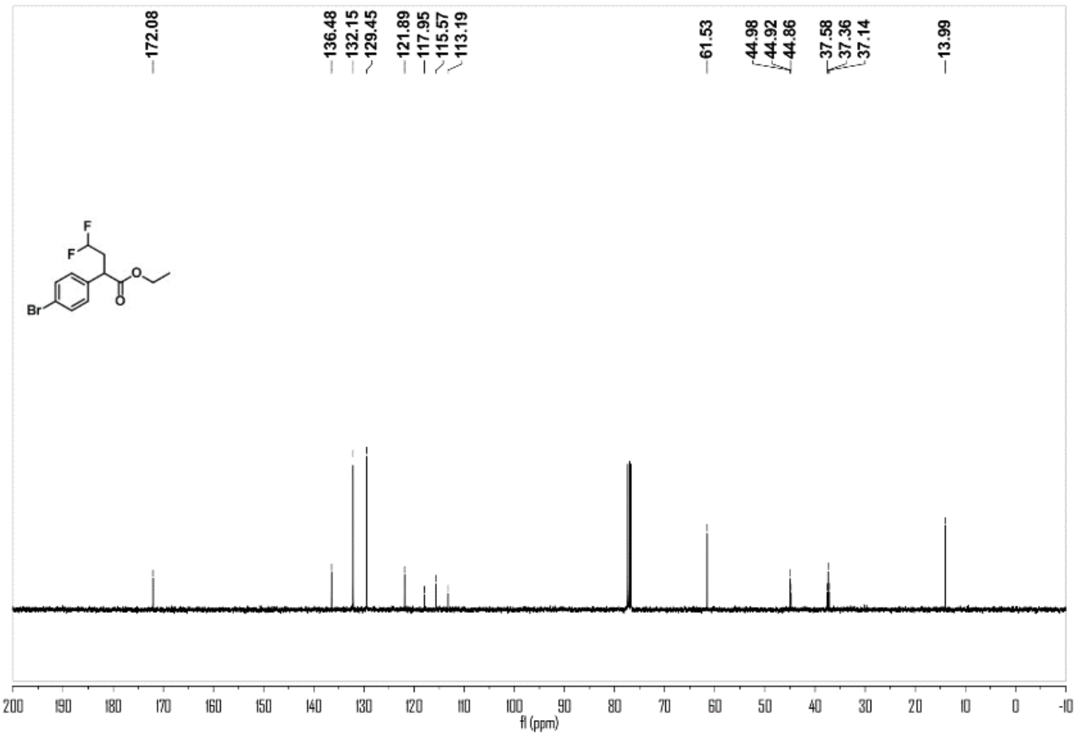
172.08
136.48
132.15
129.45
121.89
117.95
115.57
113.19
61.53
44.98
44.92
44.86
37.58
37.36
37.14
13.99
f1 (ppm)
Br
F
F
O
O

^{1}H, ^{19}F and ^{13}C NMR spectra of compound 3f

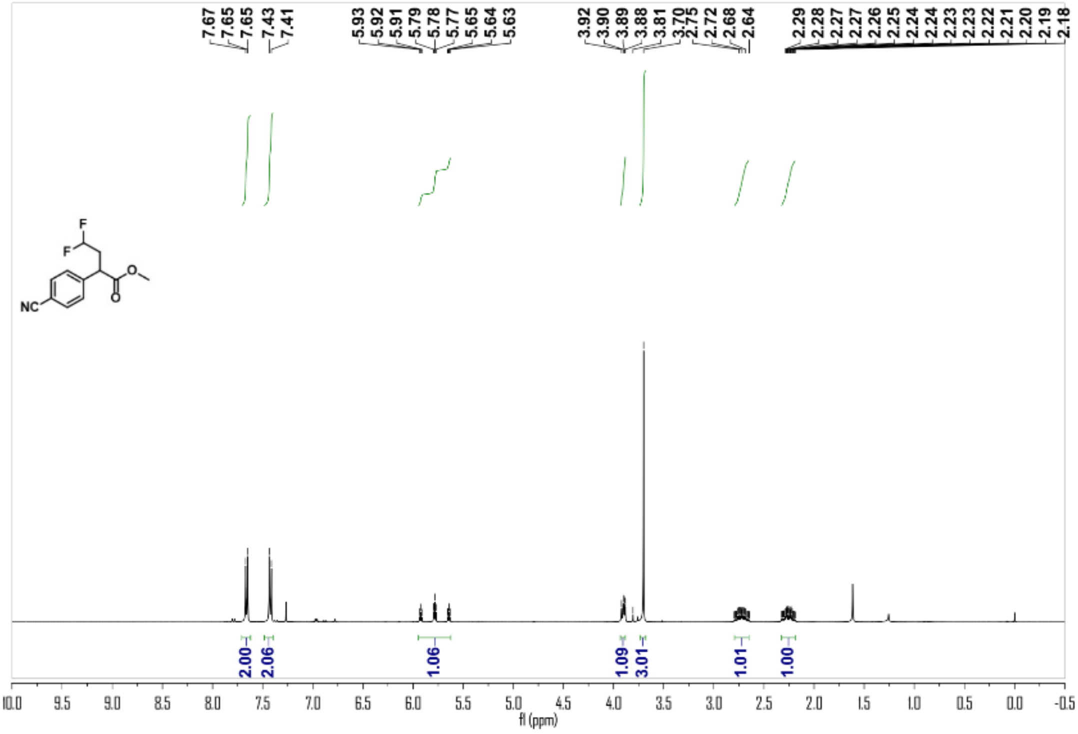

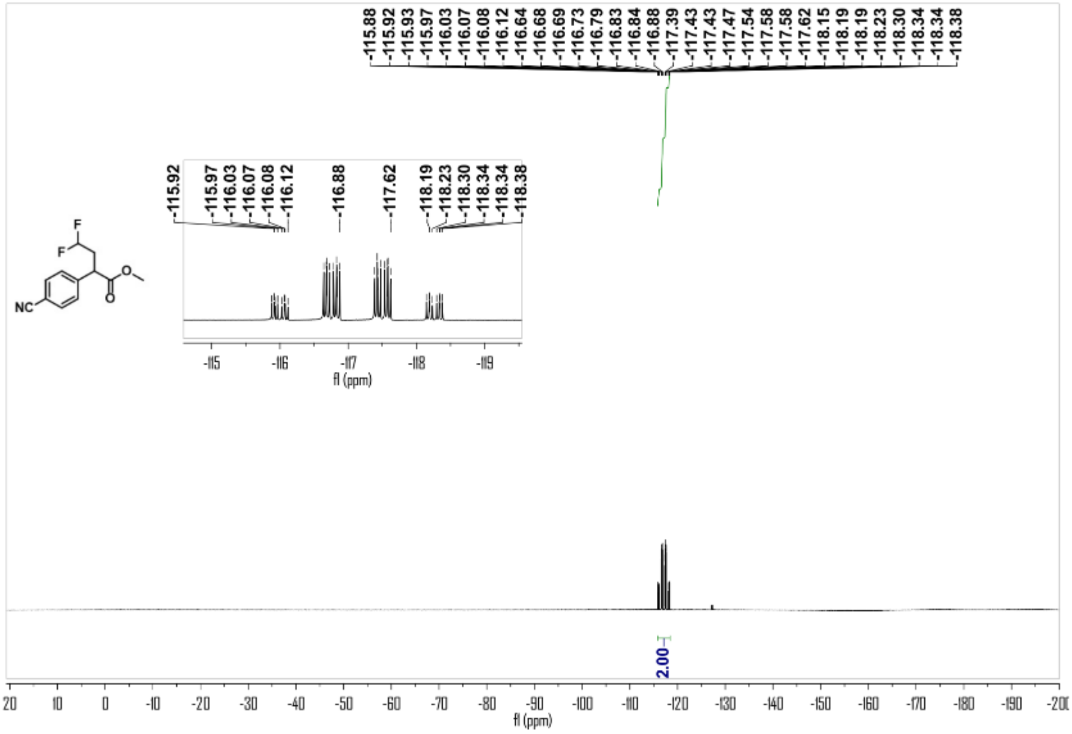

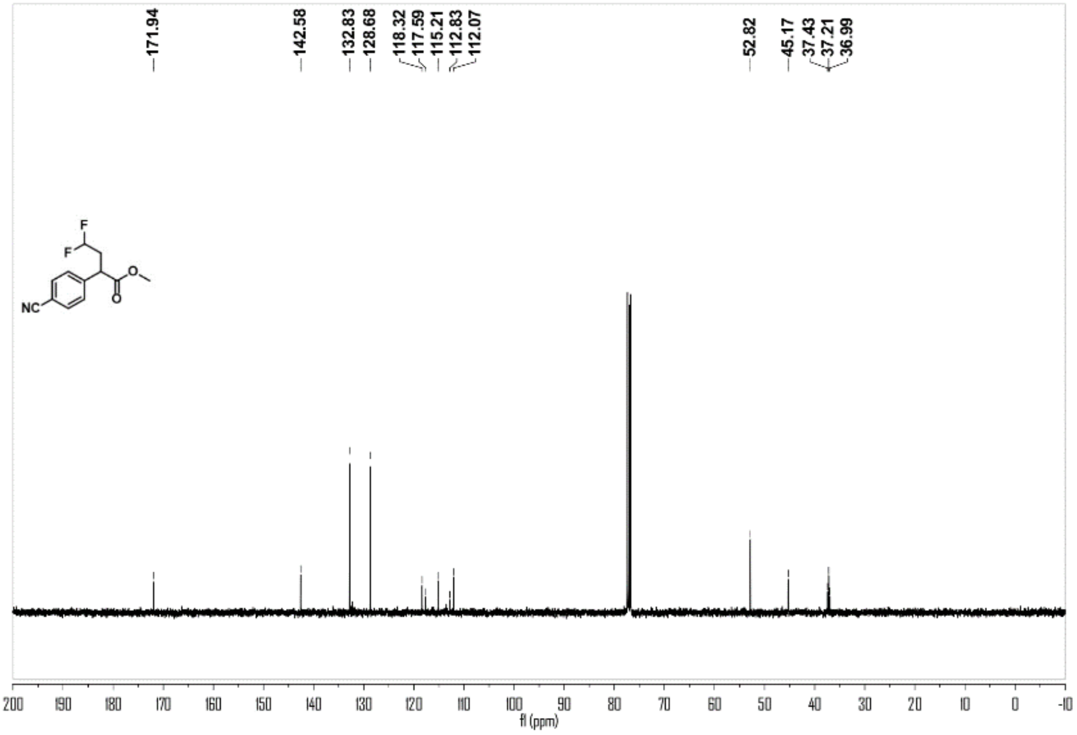

^{1}H, ^{19}F and ^{13}C **NMR spectra of compound 3g**

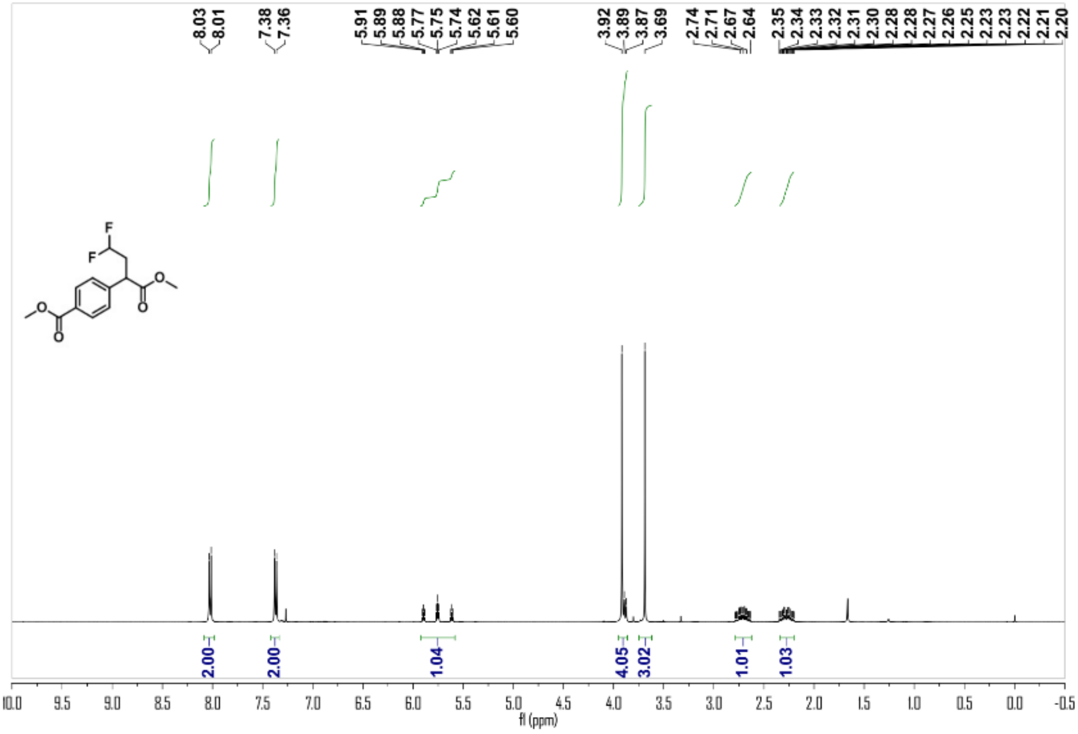

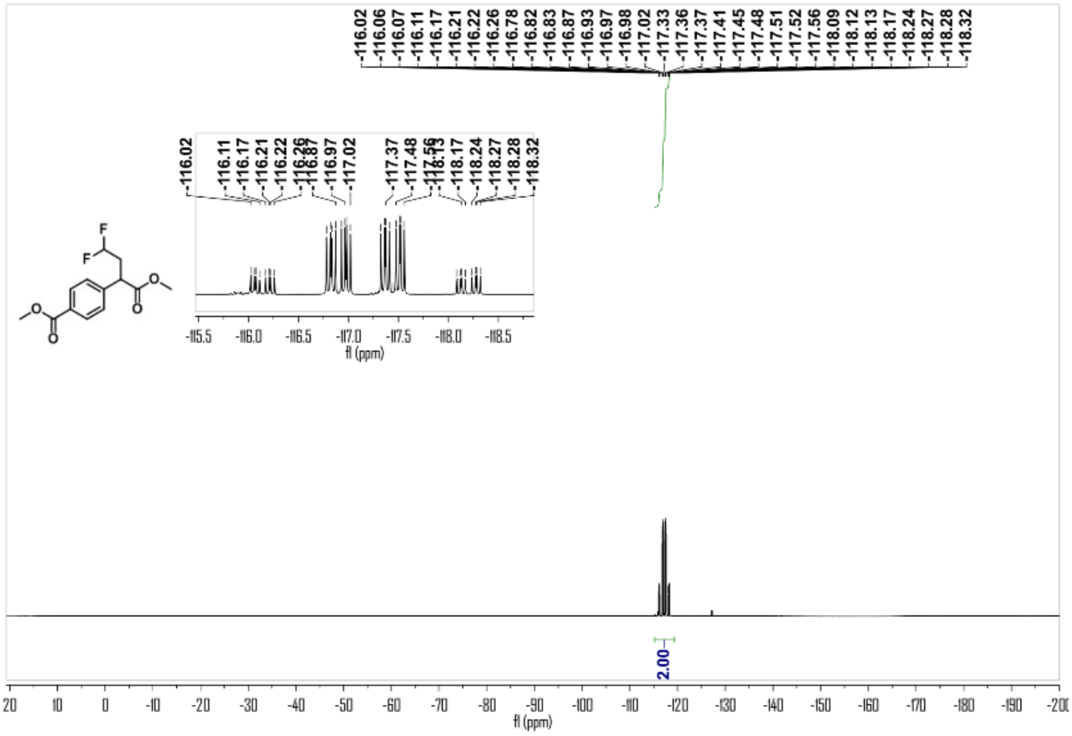
-116.02
-116.06
-116.07
-116.11
-116.17
-116.21
-116.22
-116.26
-116.78
-116.82
-116.83
-116.87
-116.93
-116.97
-116.98
-117.02
-117.33
-117.36
-117.37
-117.41
-117.45
-117.48
-117.51
-117.52
-117.56
-118.09
-118.12
-118.13
-118.17
-118.24
-118.27
-118.28
-118.32
2.00
fl (ppm)

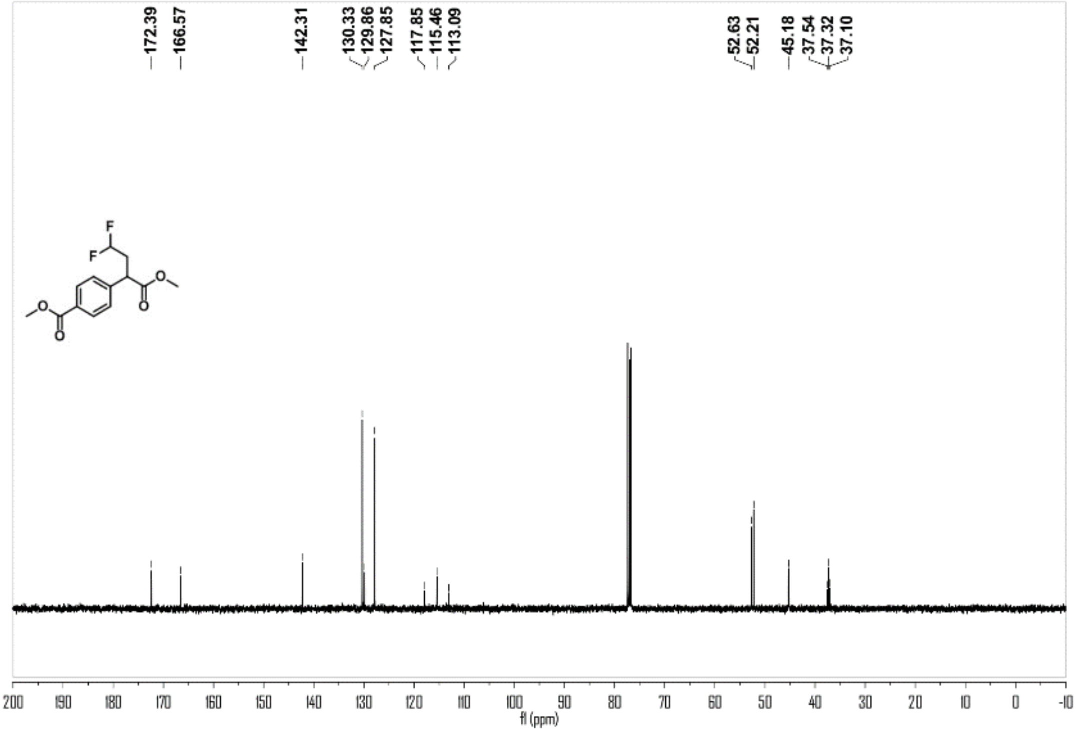
172.39
166.57
142.31
130.33
129.86
127.85
117.85
115.46
113.09
52.63
52.21
45.18
37.54
37.32
37.10
fl (ppm)

^{1}H, ^{19}F and ^{13}C NMR spectra of compound 3h

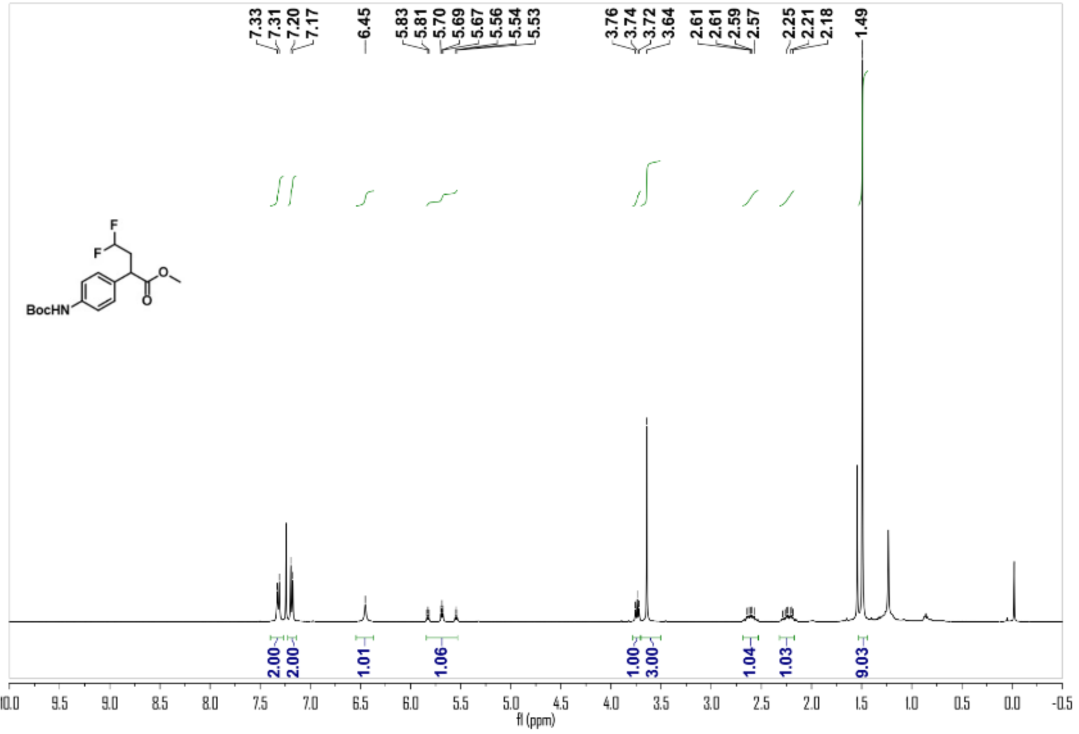

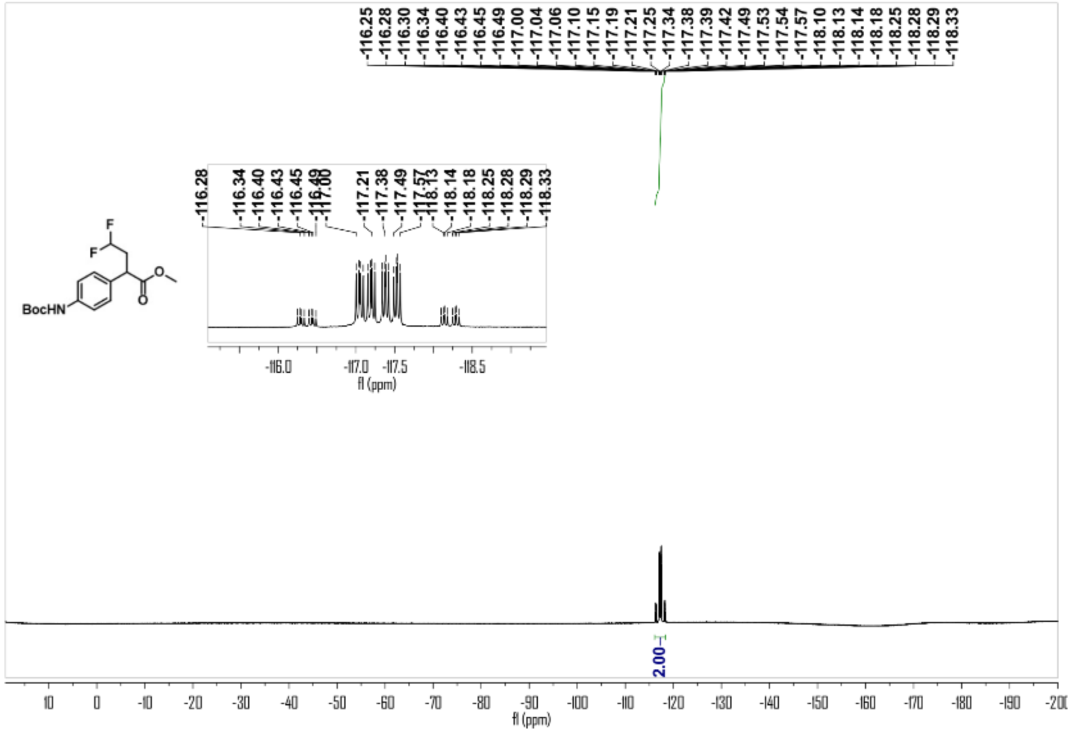

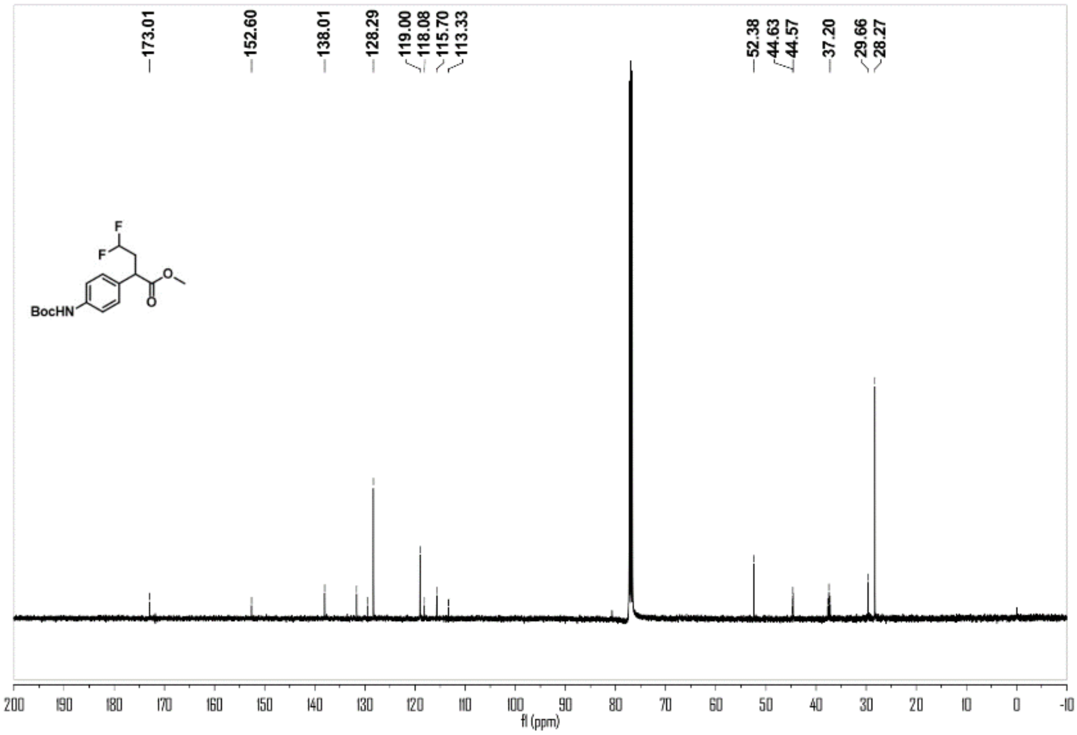

^{1}H, ^{19}F and ^{13}C NMR spectra of compound 3i

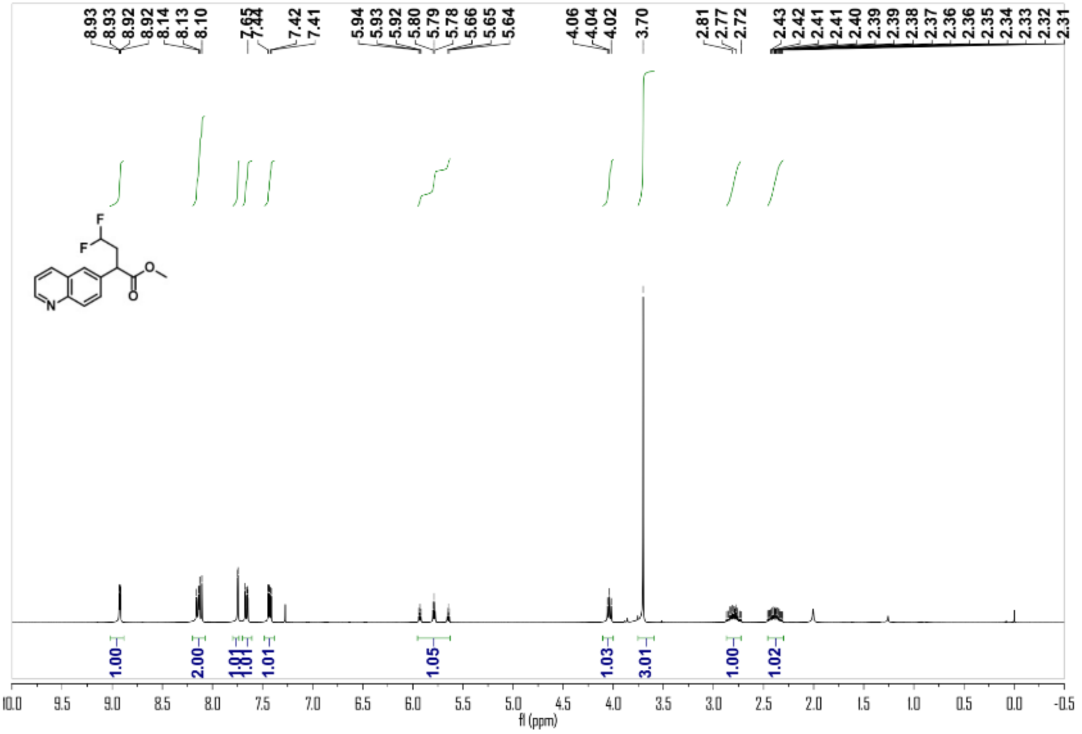

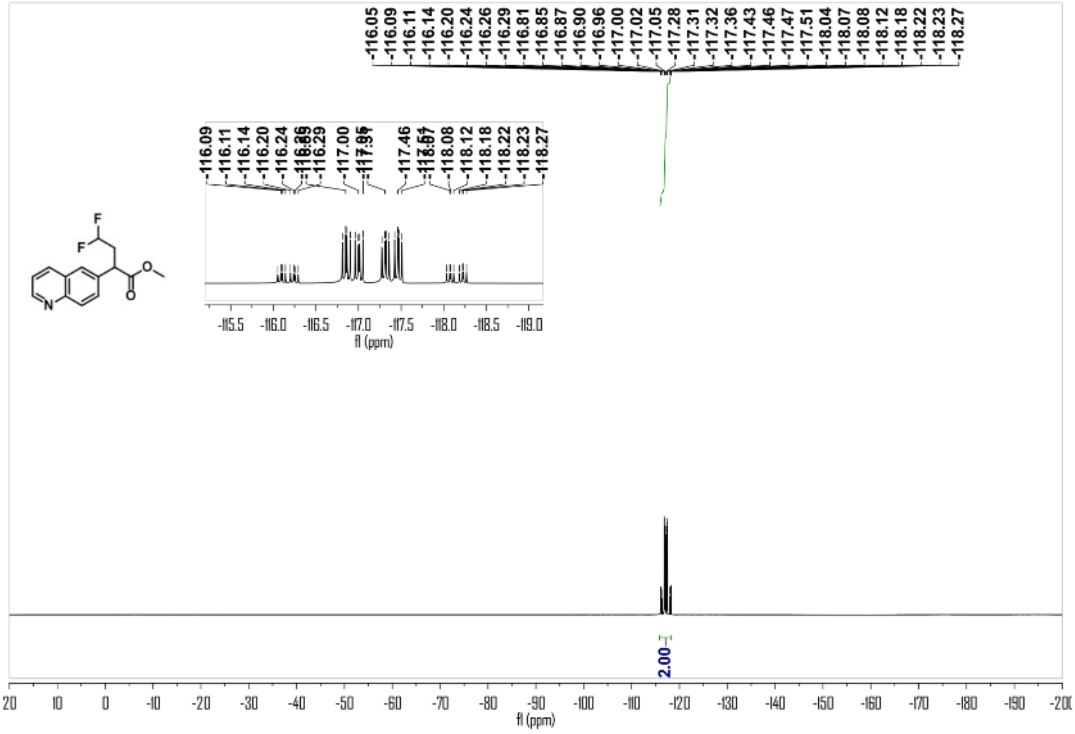

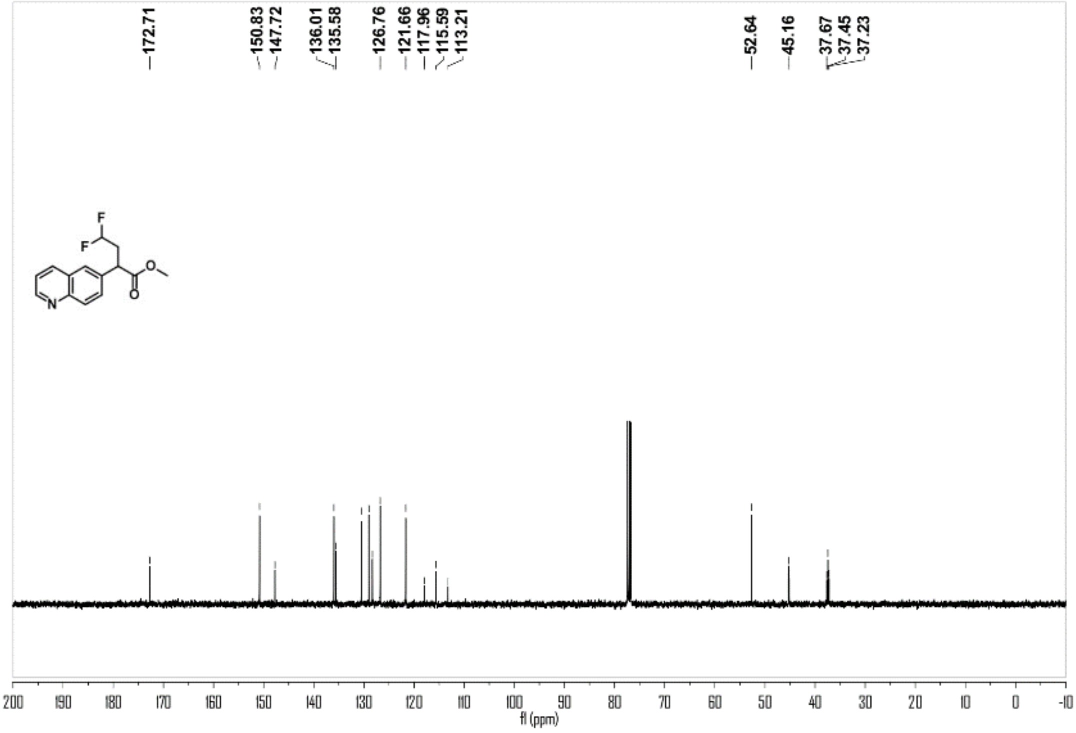

^{1}H, ^{19}F and ^{13}C NMR spectra of compound 3j

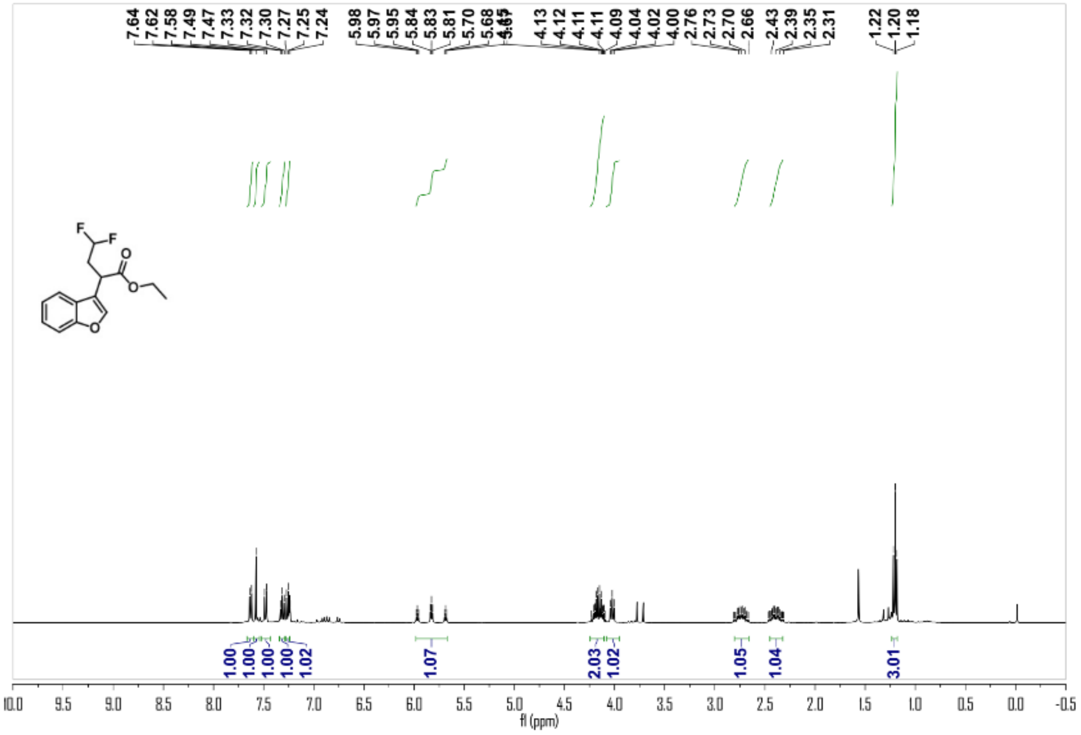

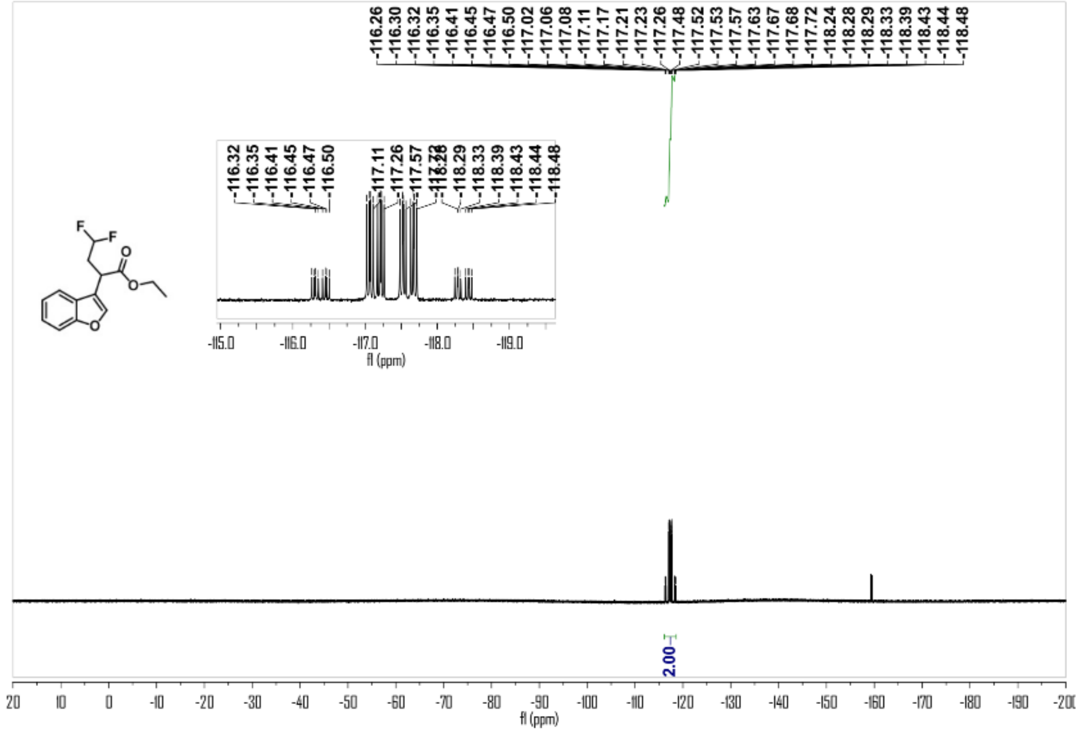

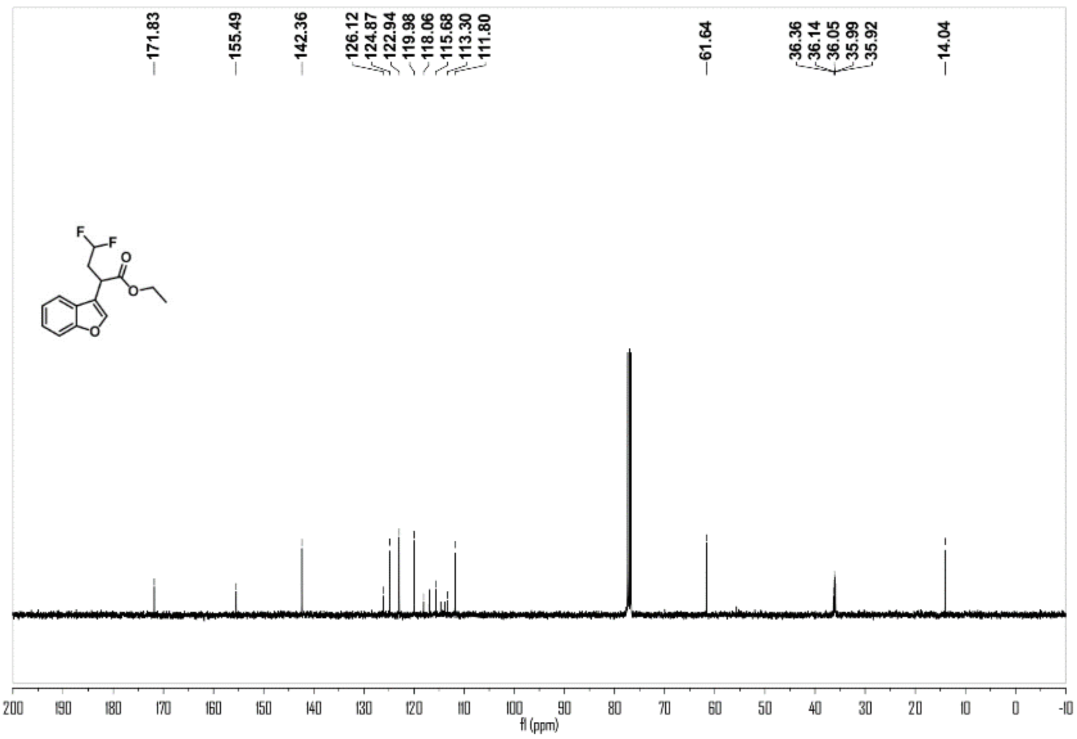

^{1}H, ^{19}F and ^{13}C NMR spectra of compound 3k

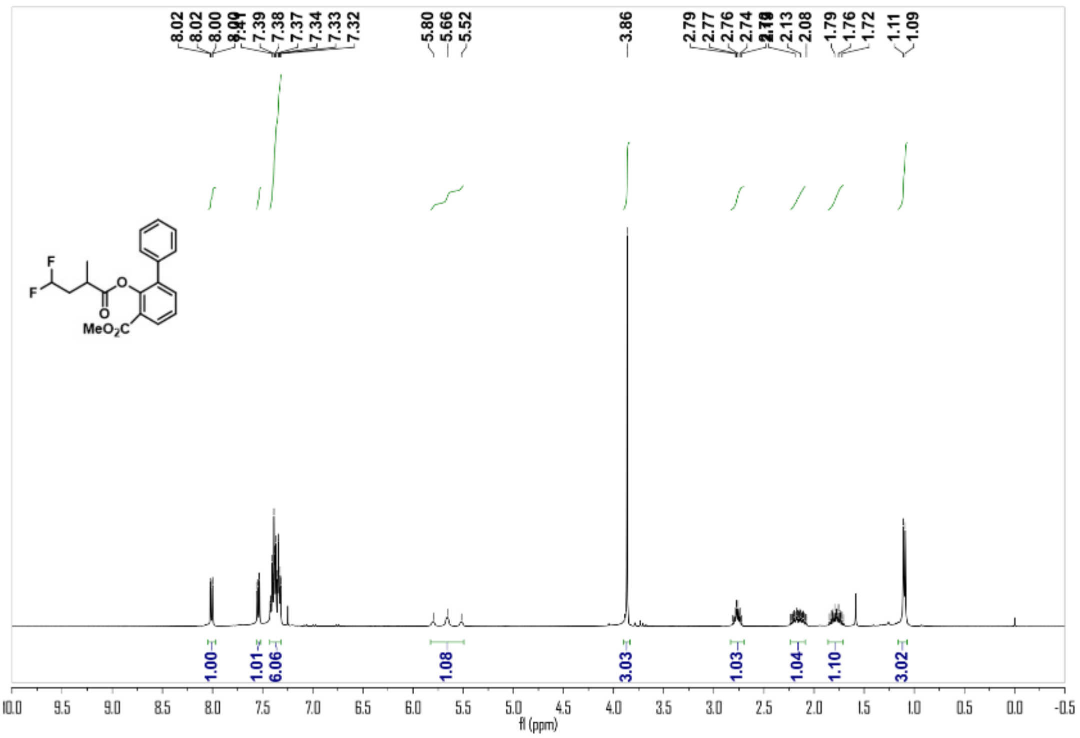

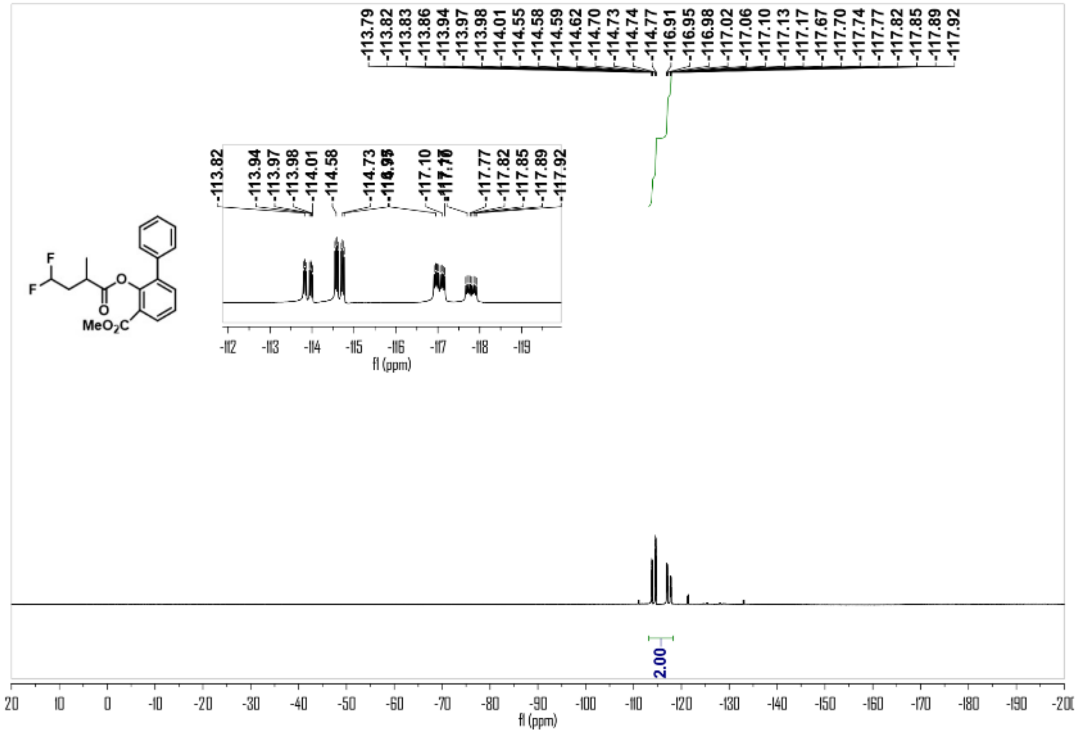

-113.79
-113.82
-113.83
-113.86
-113.94
-113.97
-113.98
-114.01
-114.55
-114.58
-114.59
-114.62
-114.70
-114.73
-114.74
-114.77
-116.91
-116.95
-116.98
-117.02
-117.06
-117.10
-117.13
-117.17
-117.67
-117.70
-117.74
-117.77
-117.82
-117.85
-117.89
-117.92
F
F
O
O
MeO2C
2.00
fl (ppm)

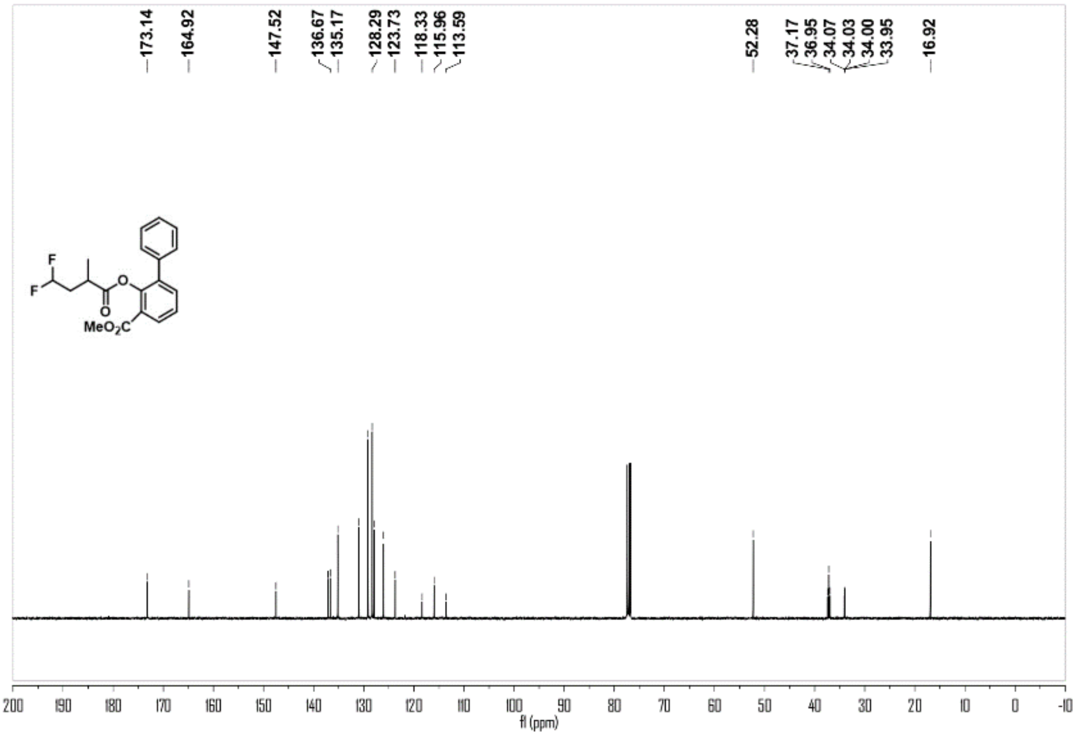

173.14
164.92
147.52
136.67
135.17
128.29
123.73
118.33
115.96
113.59
52.28
37.17
36.95
34.07
34.03
34.00
33.95
16.92
F
F
O
O
MeO2C
fl (ppm)

^{1}H, ^{19}F and ^{13}C NMR spectra of compound 3l

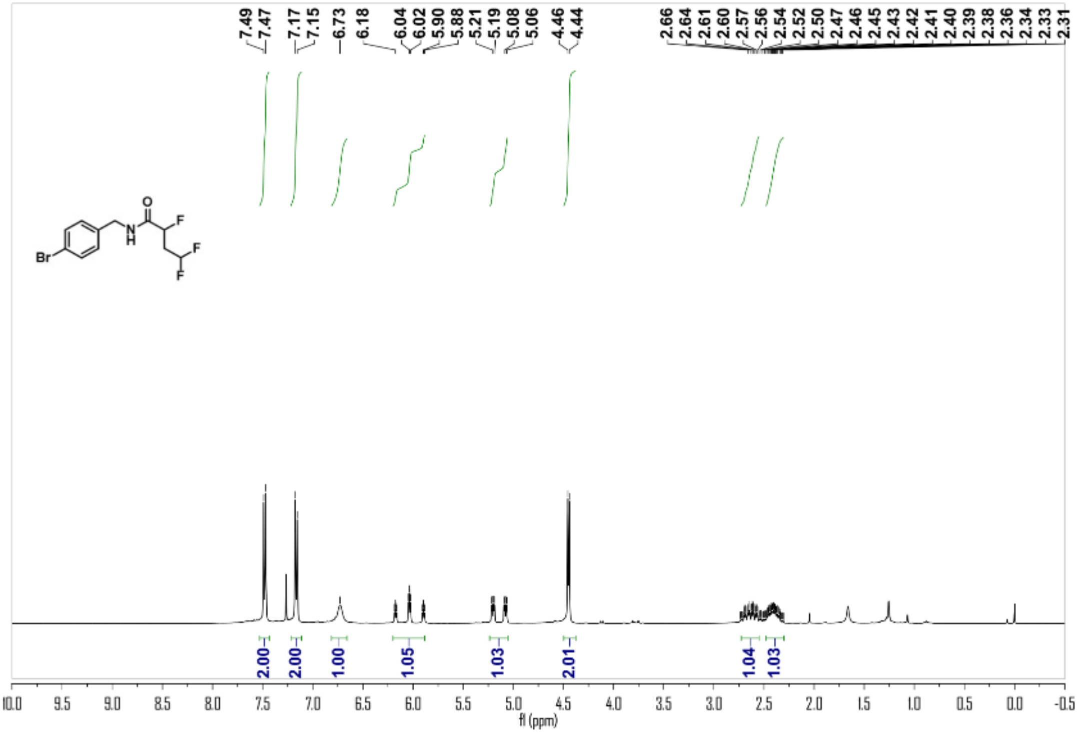

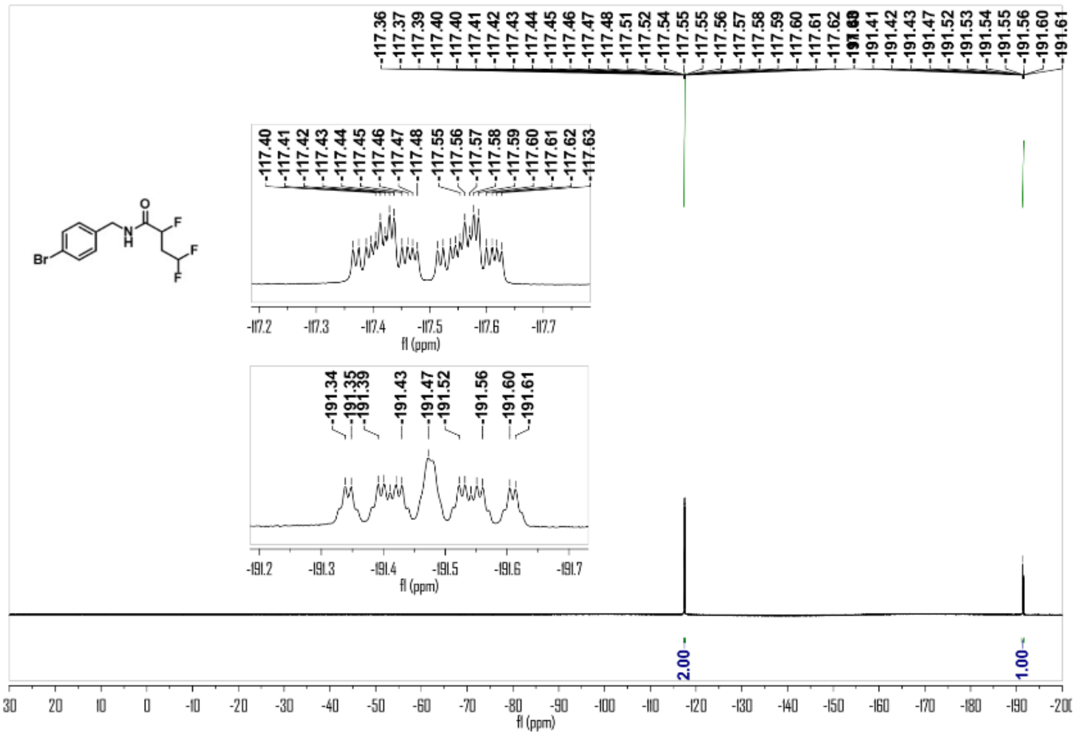

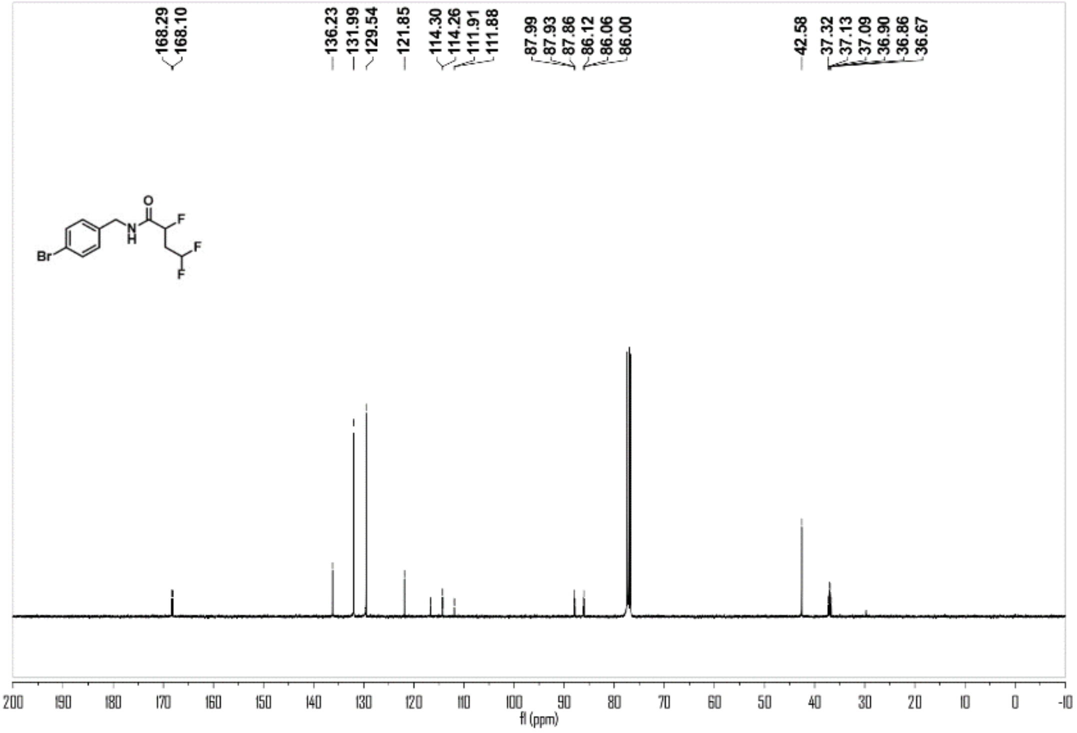

^{1}H, ^{19}F and ^{13}C NMR spectra of compound 3m

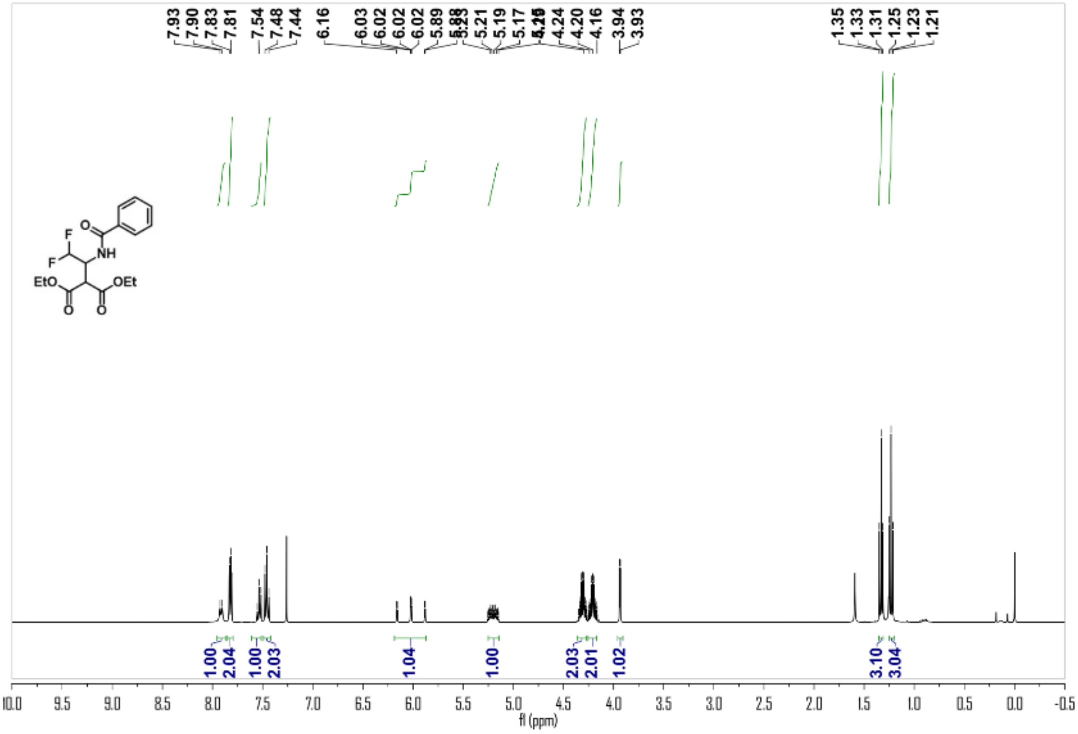

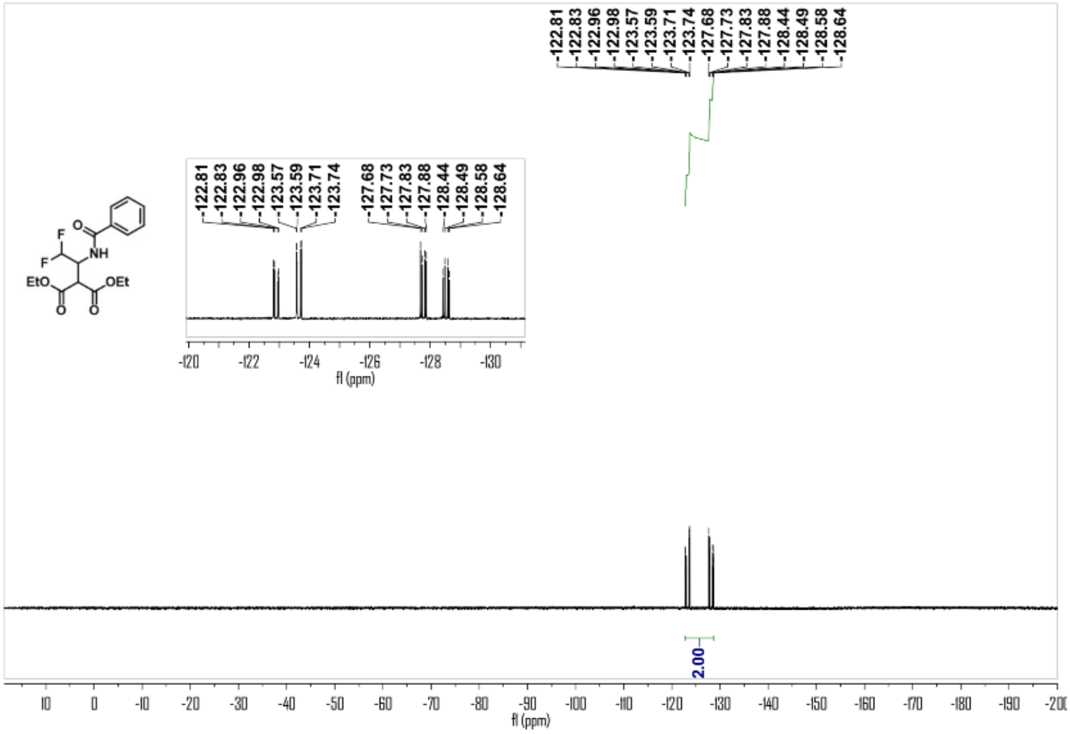
-122.81
-122.83
-122.96
-122.98
-123.57
-123.59
-123.71
-123.74
-127.68
-127.73
-127.83
-127.88
-128.44
-128.49
-128.58
-128.64
2.00
fl (ppm)

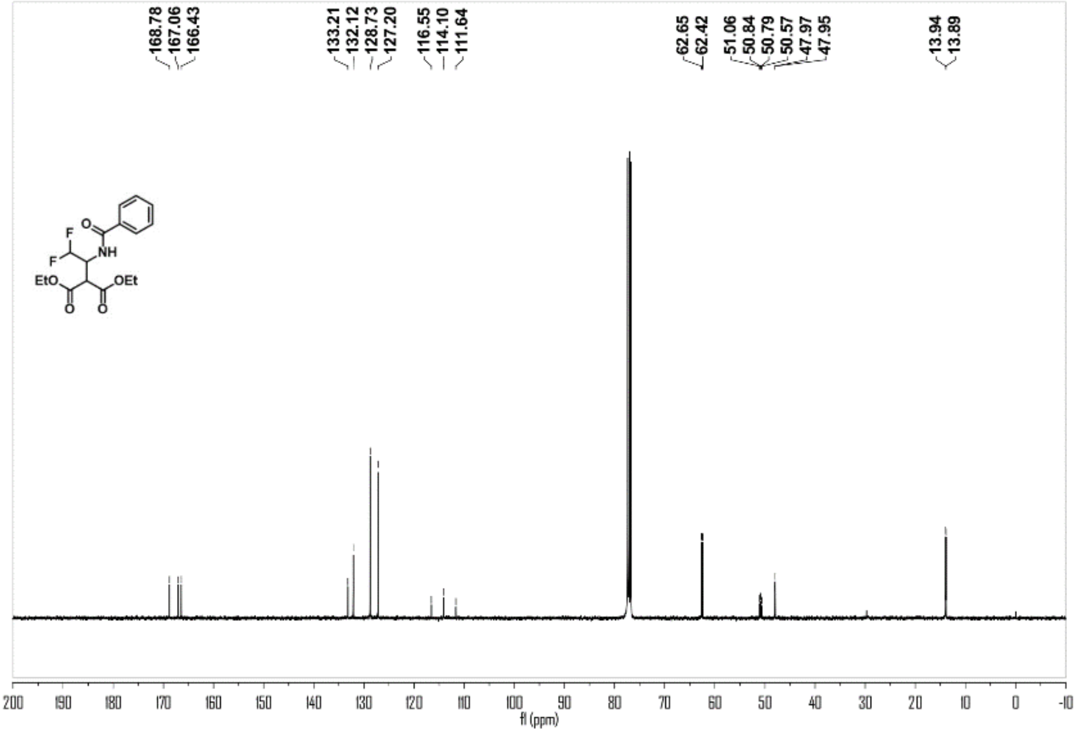
168.78
167.06
166.43
133.21
132.12
128.73
127.20
116.55
114.10
111.64
62.65
62.42
51.06
50.84
50.79
50.57
47.97
47.95
13.94
13.89
fl (ppm)

^{1}H, ^{19}F and ^{13}C NMR spectra of compound 3n

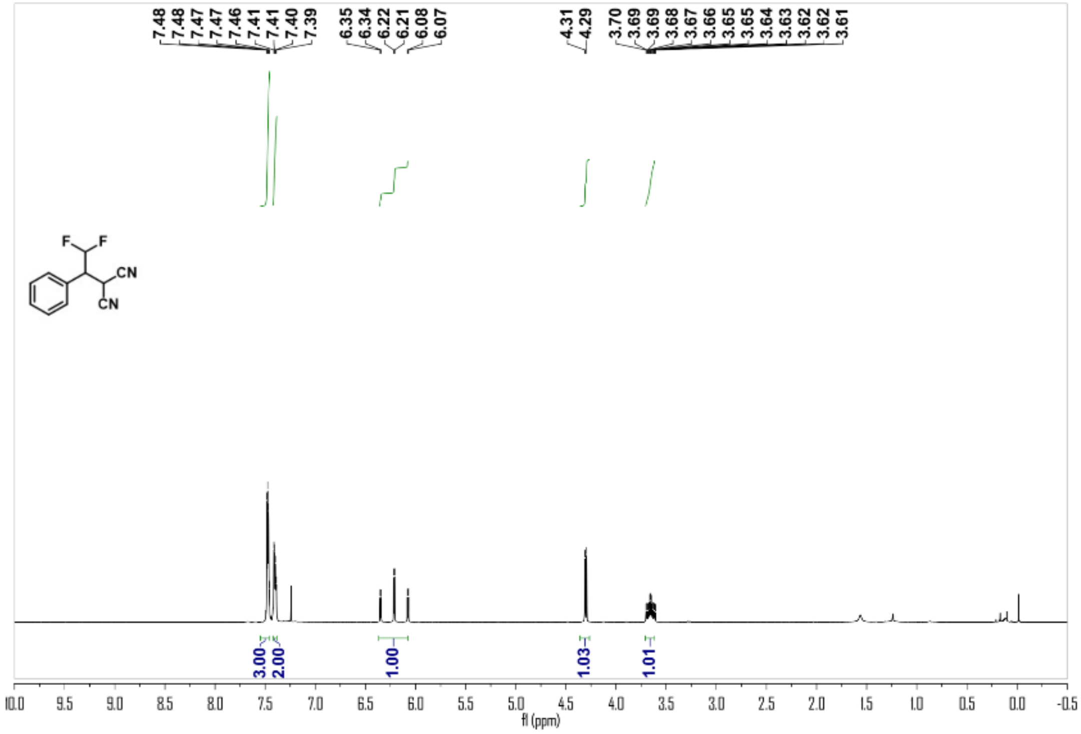

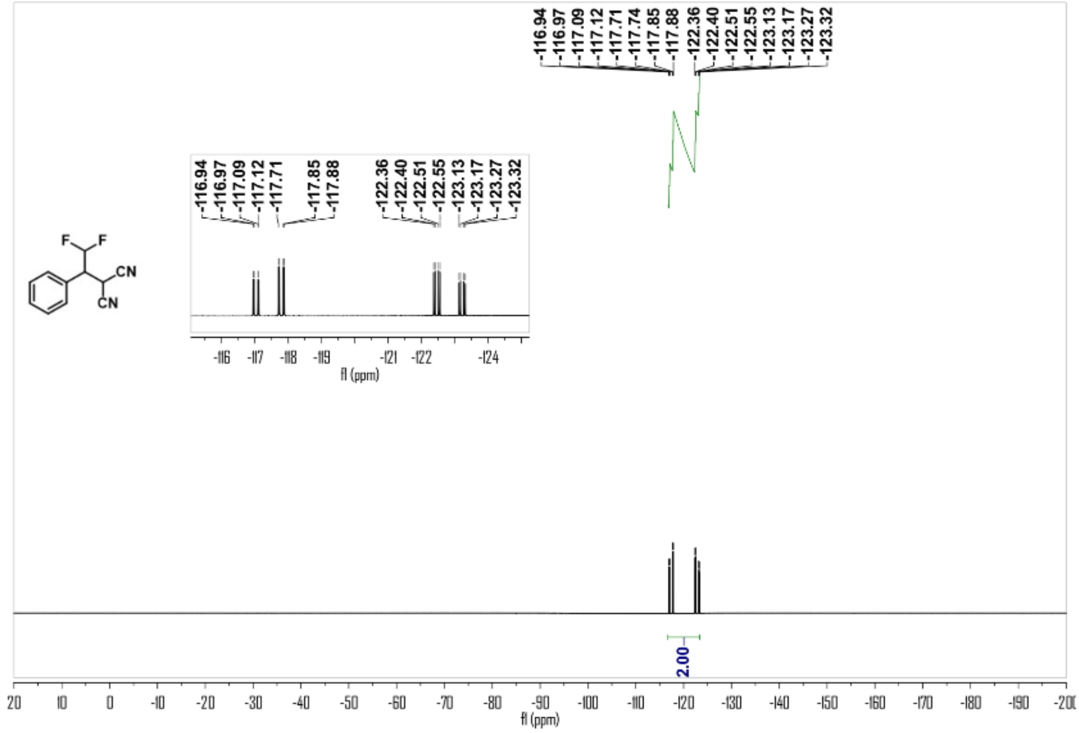

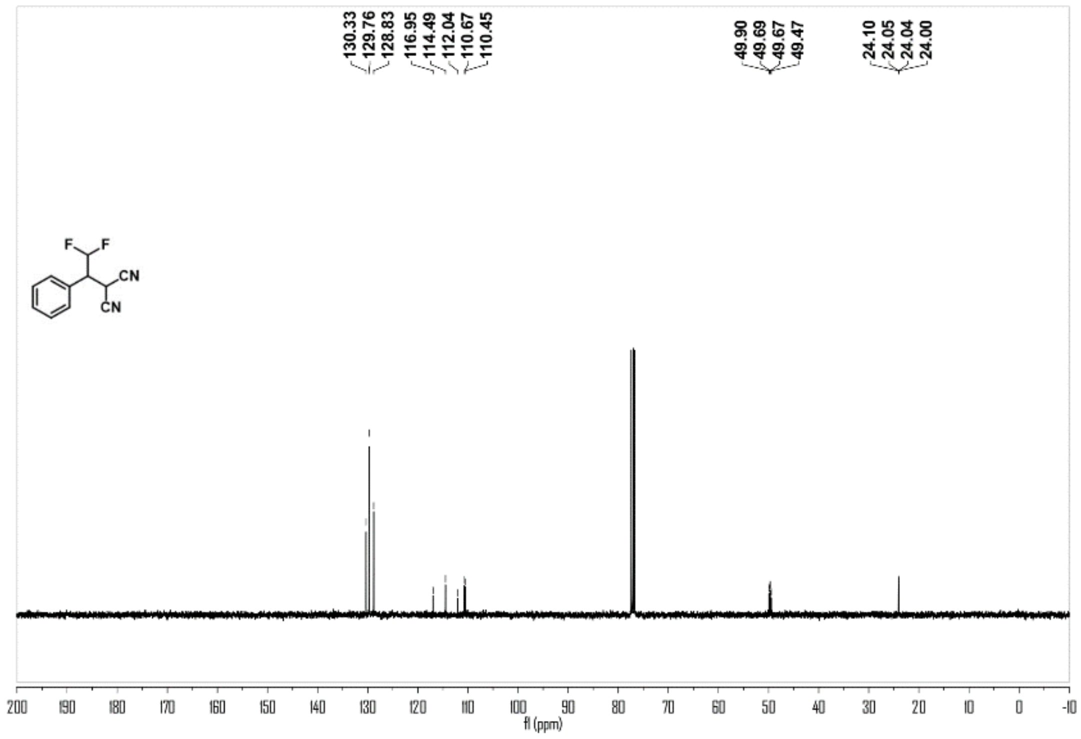

^{1}H, ^{19}F and ^{13}C NMR spectra of compound 3o

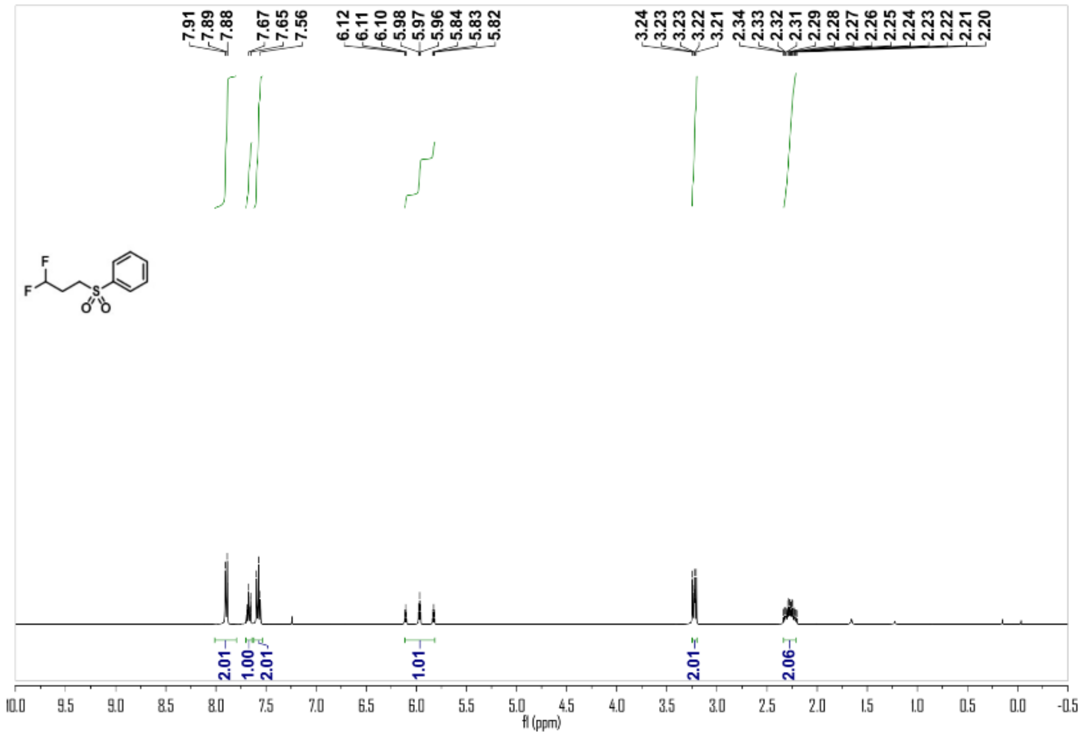

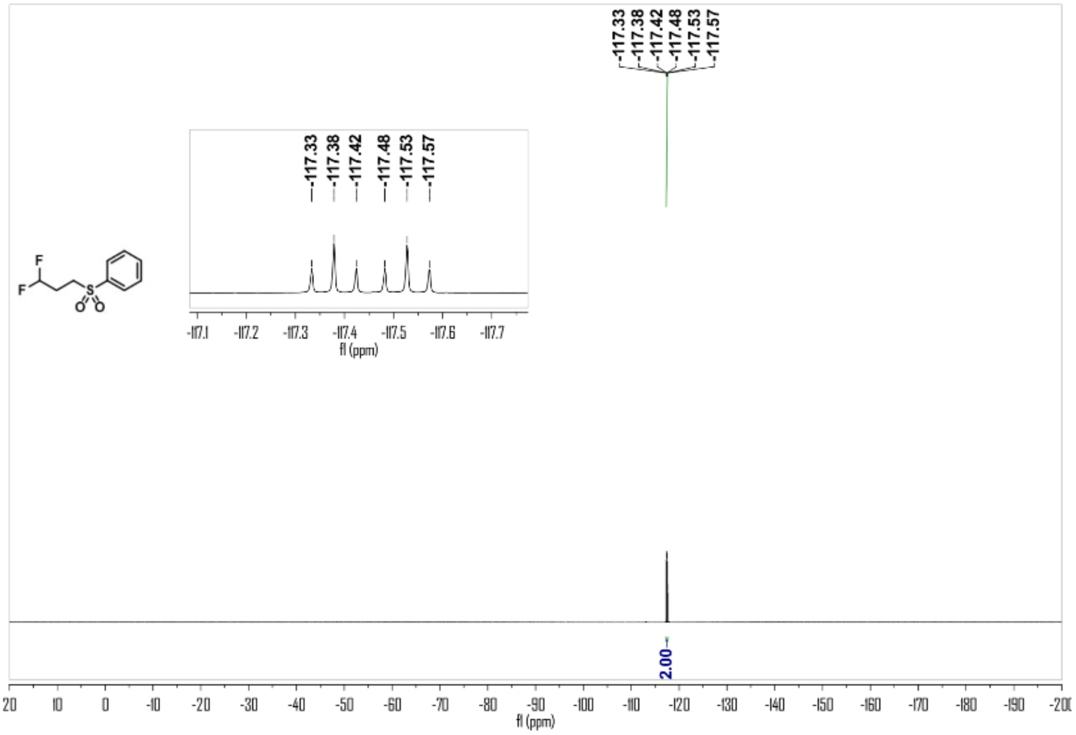
-117.33
-117.38
-117.42
-117.48
-117.53
-117.57
2.00
f1 (ppm)

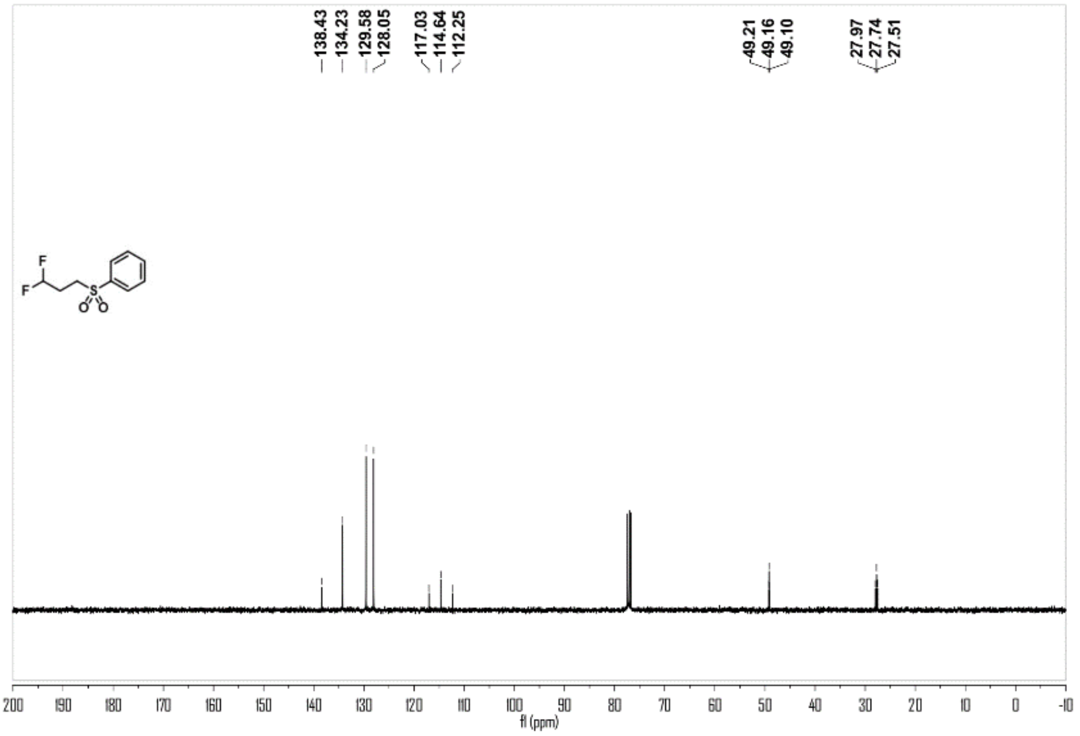
138.43
134.23
129.58
128.05
117.03
114.64
112.25
49.21
49.16
49.10
27.97
27.74
27.51
f1 (ppm)

^{1}H, ^{19}F and ^{13}C NMR spectra of compound 3p

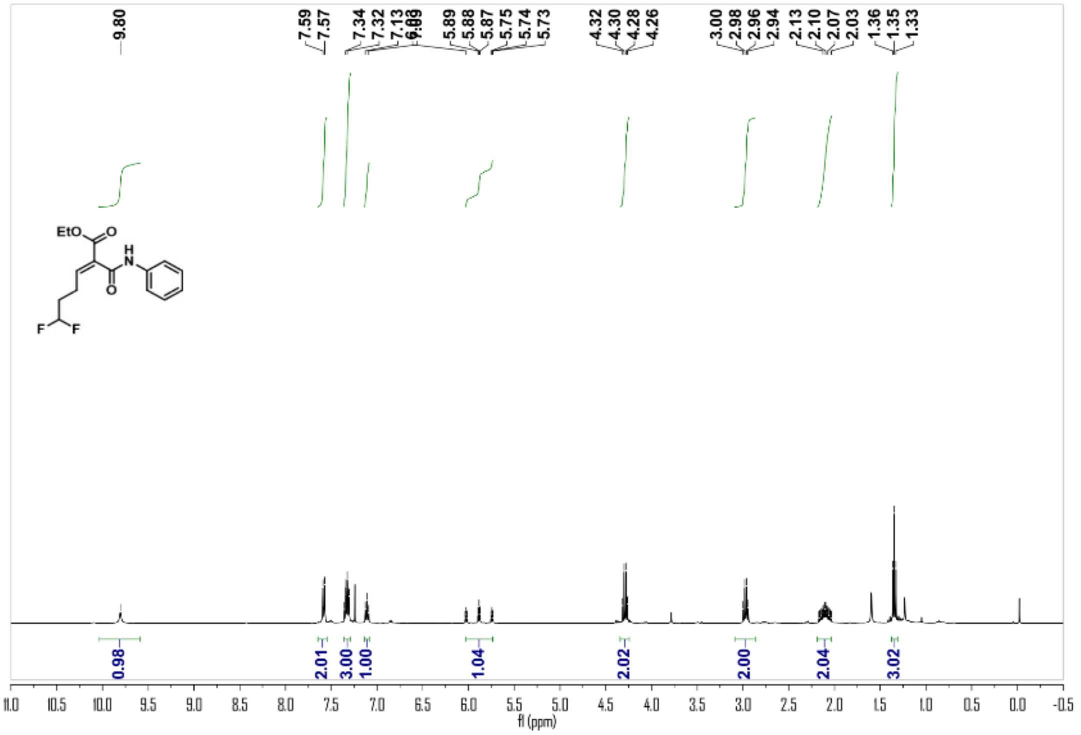

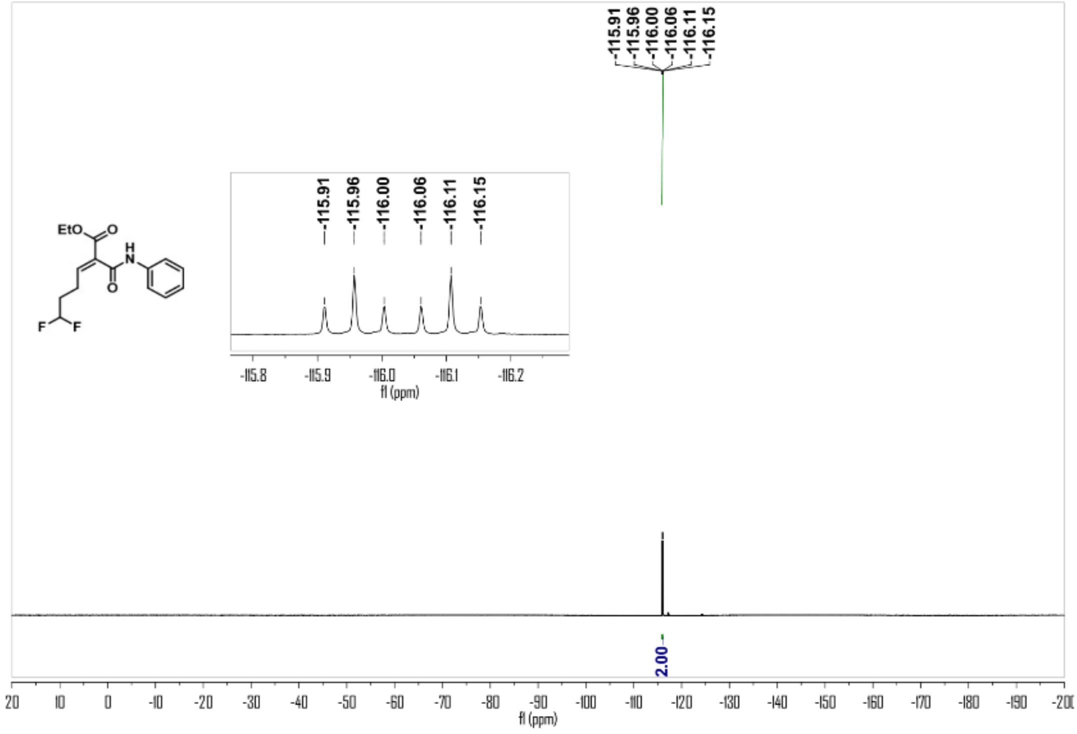

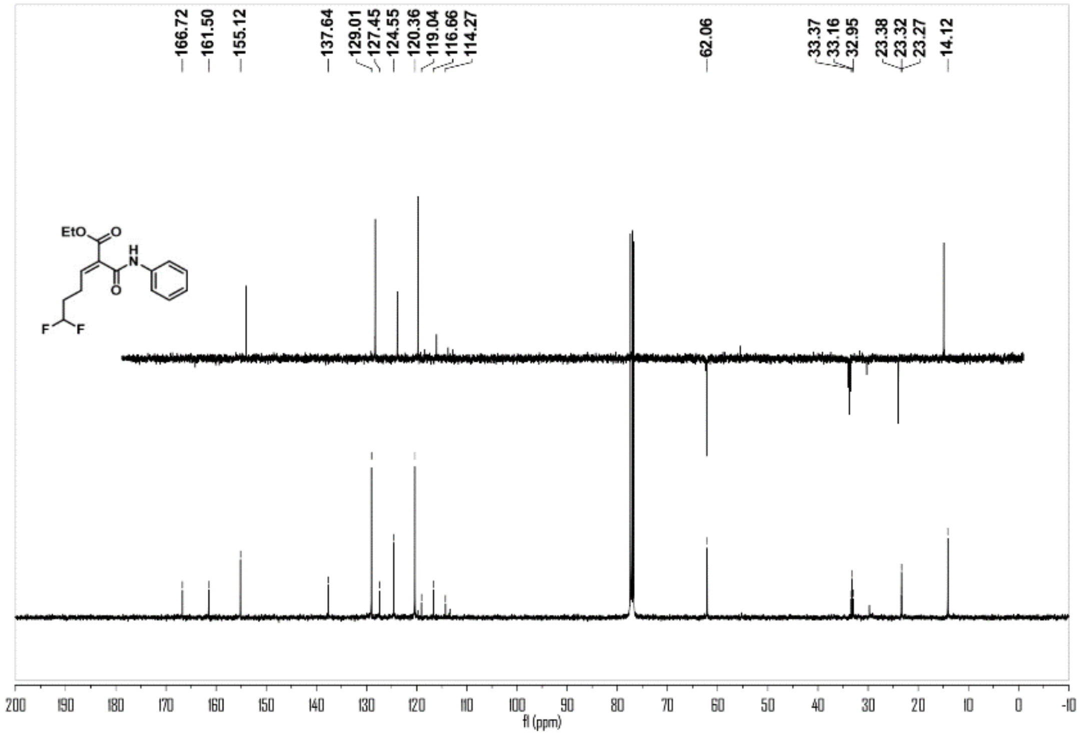

^{1}H, ^{19}F and ^{13}C NMR spectra of compound 3q

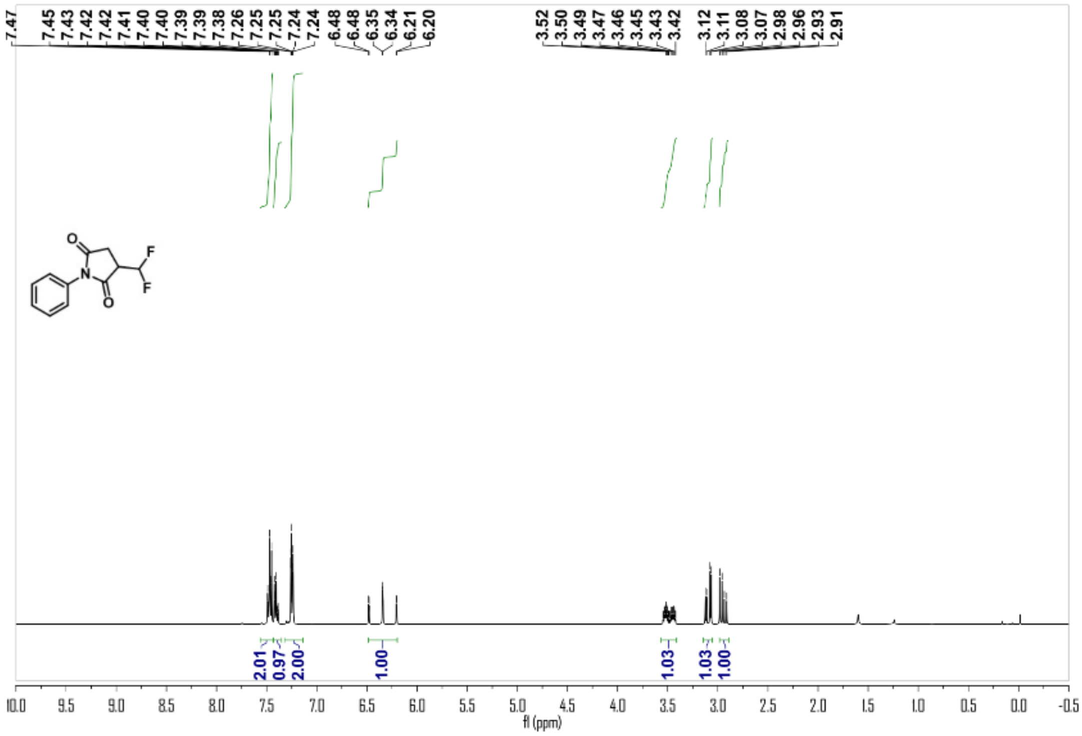

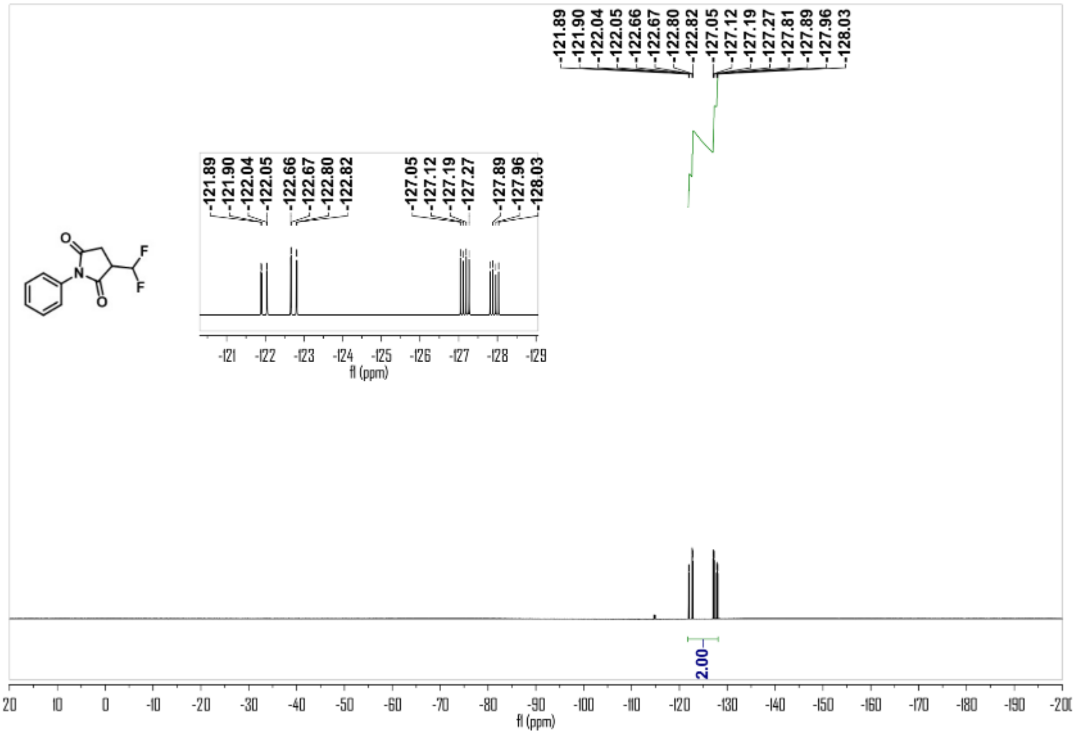
-121.89
-121.90
-122.04
-122.05
-122.66
-122.67
-122.80
-122.82
-127.05
-127.12
-127.19
-127.27
-127.81
-127.89
-127.96
-128.03
2.00
fl (ppm)

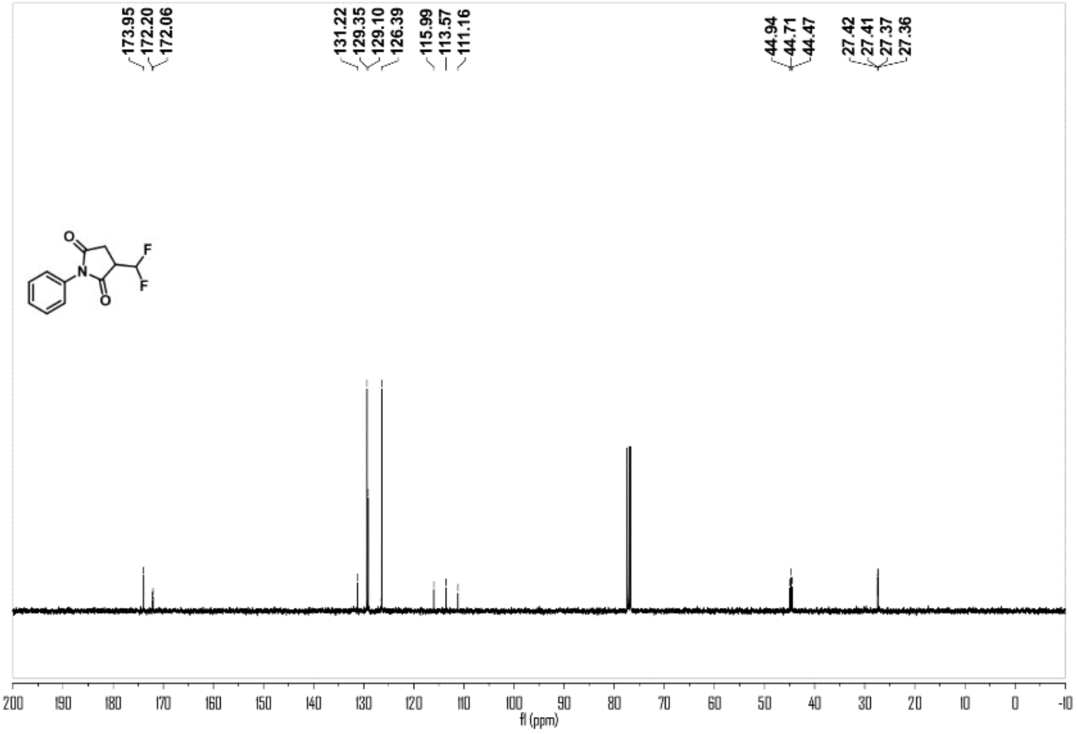
173.95
172.20
172.06
131.22
129.35
129.10
126.39
115.99
113.57
111.16
44.94
44.71
44.47
27.42
27.41
27.37
27.36
fl (ppm)

^{1}H, ^{19}F and ^{13}C NMR spectra of compound 3r

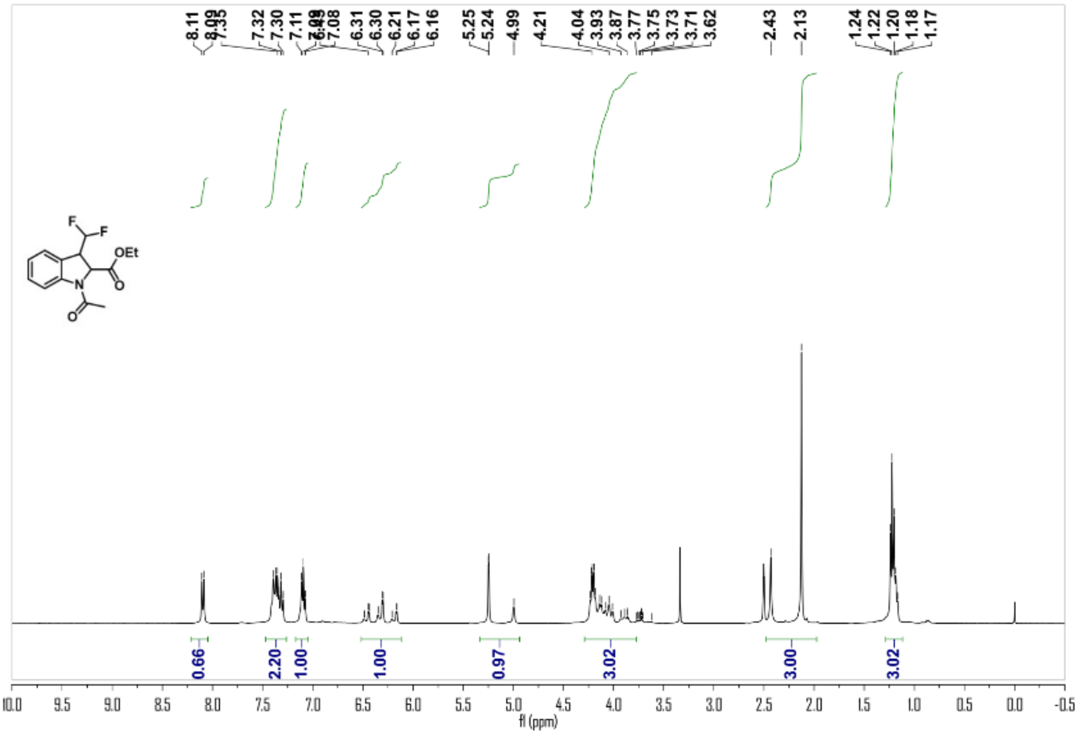

^{1}H NMR spectra of compound 3r at 363 K

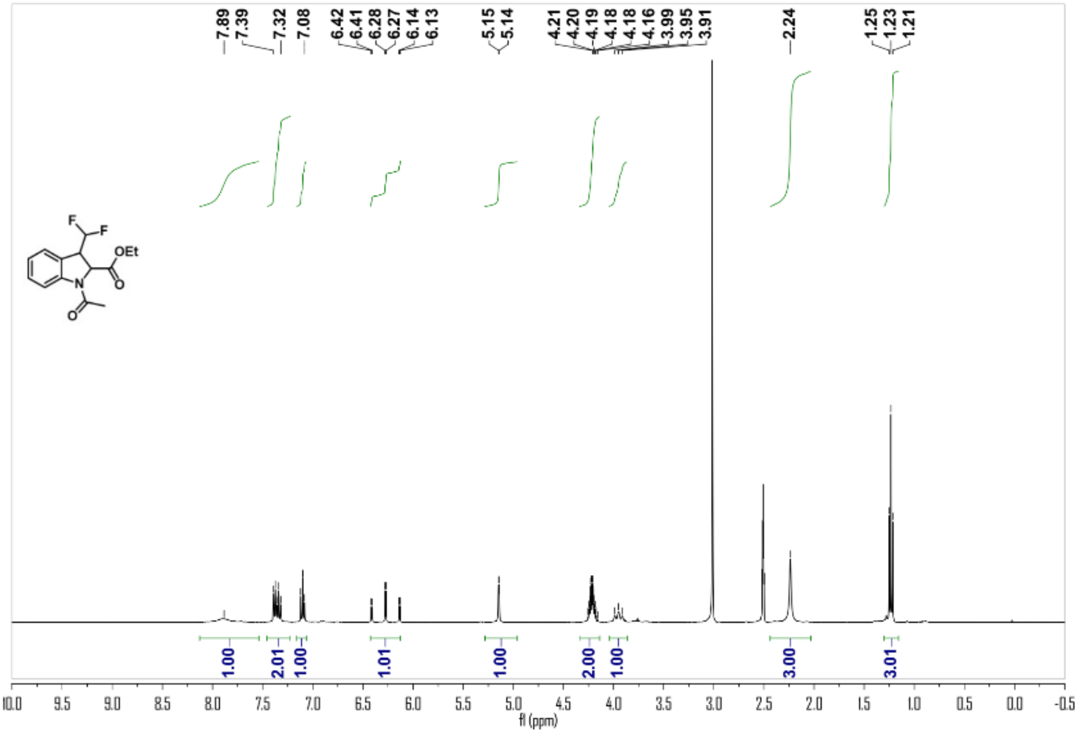

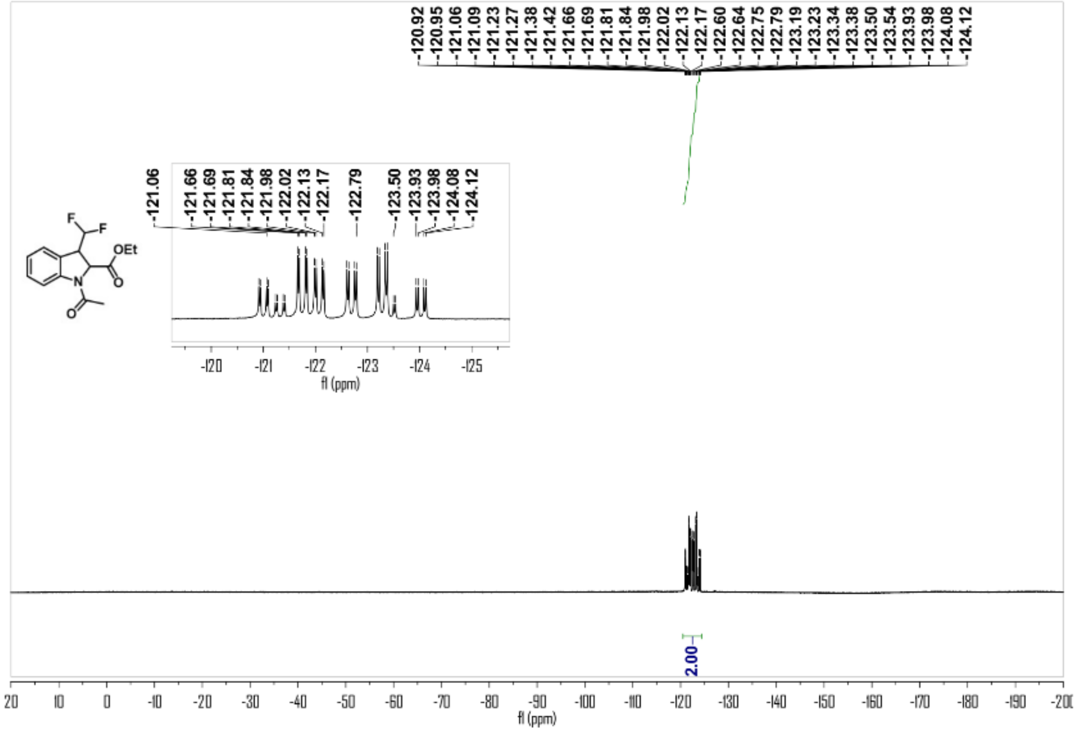
-120.92
-120.95
-121.06
-121.09
-121.23
-121.27
-121.38
-121.42
-121.66
-121.69
-121.81
-121.84
-121.98
-122.02
-122.13
-122.17
-122.60
-122.64
-122.75
-122.79
-123.19
-123.23
-123.34
-123.38
-123.50
-123.54
-123.93
-123.98
-124.08
-124.12
F
F
OEt
N
O
O
2.00
fl (ppm)

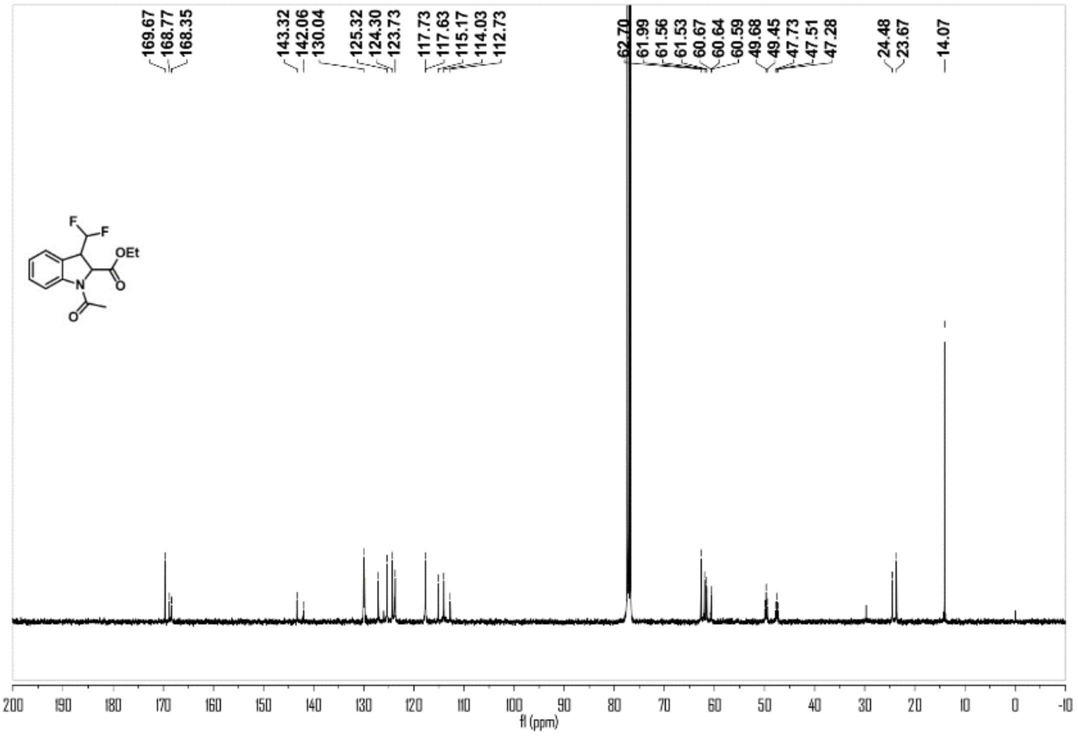
169.67
168.77
168.35
143.32
142.06
130.04
125.32
124.30
123.73
117.73
117.63
115.17
114.03
112.73
62.70
61.99
61.56
61.53
60.67
60.64
60.59
49.68
49.45
47.73
47.51
47.28
24.48
23.67
14.07
F
F
OEt
N
O
O
fl (ppm)

^{1}H, ^{19}F and ^{13}C NMR spectra of compound 3s

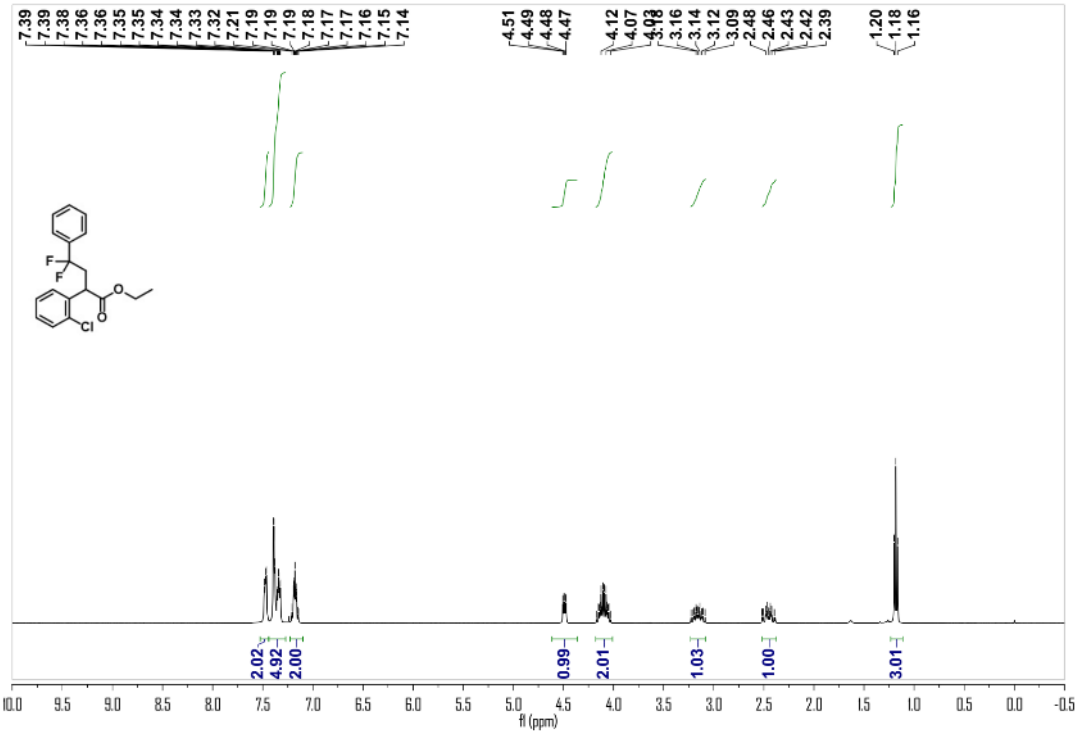

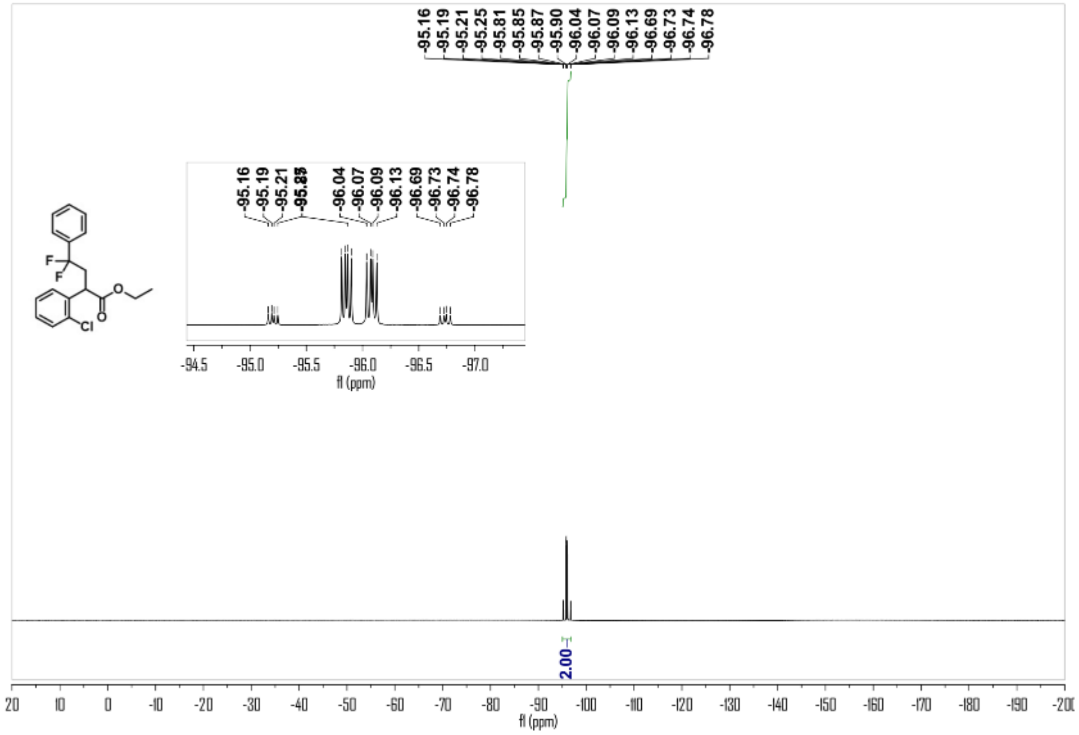

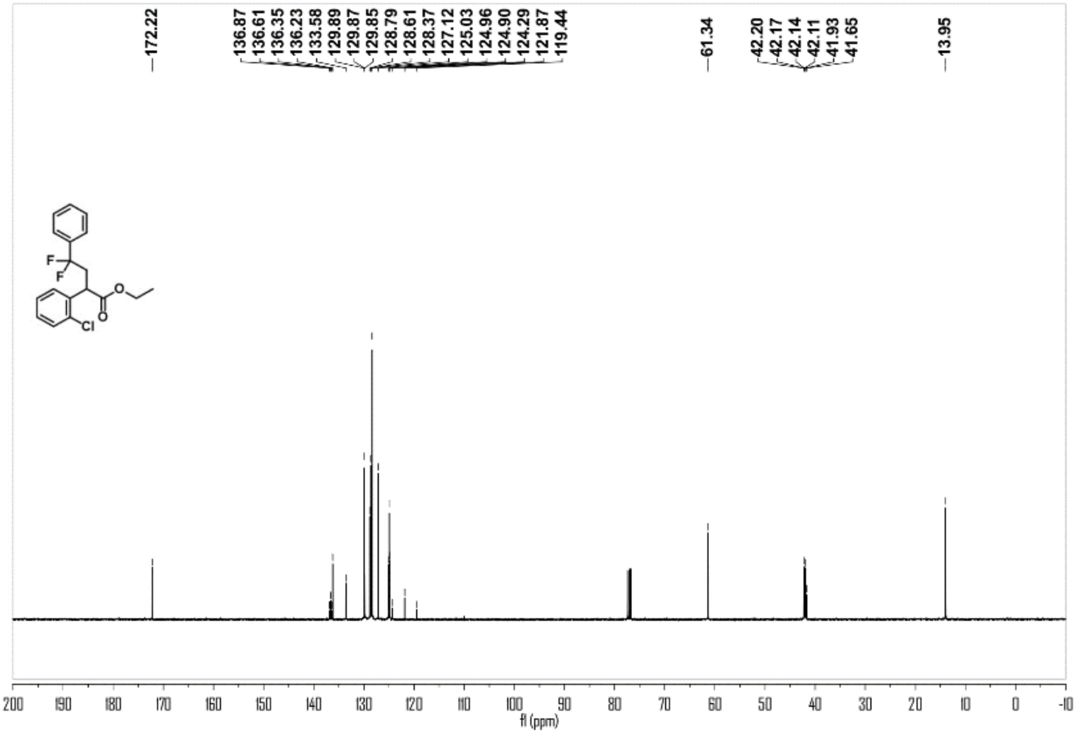

^{1}H, ^{19}F and ^{13}C NMR spectra of compound 3t

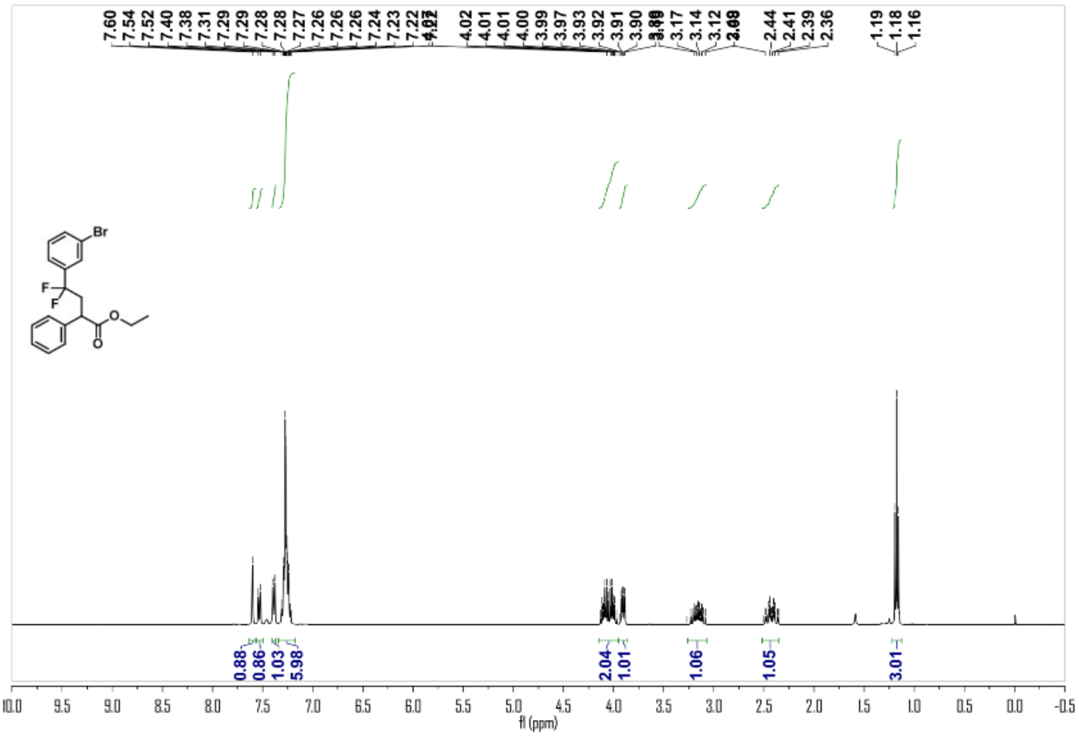

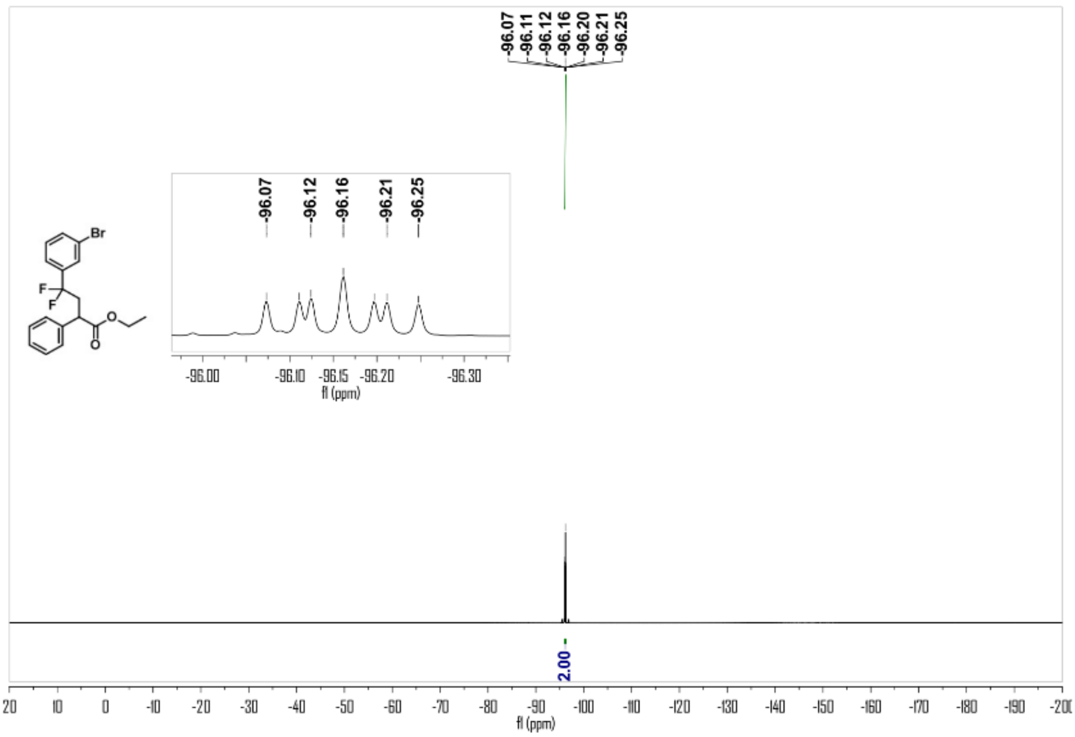
-96.07
-96.11
-96.12
-96.16
-96.20
-96.21
-96.25
Br
F
F
O
O
-96.07
-96.12
-96.16
-96.21
-96.25
-96.00
-96.10
-96.15
-96.20
-96.30
f1 (ppm)
2.00
20 10 0 -10 -20 -30 -40 -50 -60 -70 -80 -90 -100 -110 -120 -130 -140 -150 -160 -170 -180 -190 -200
f1 (ppm)

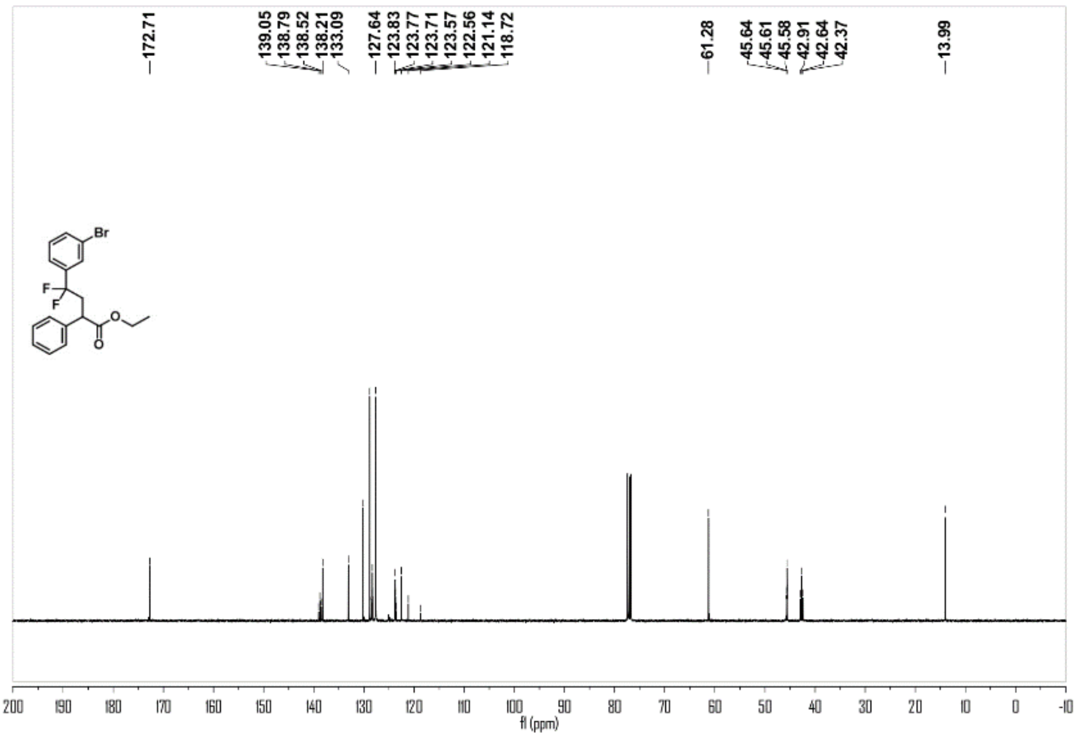
172.71
139.05
138.79
138.52
138.21
133.09
127.64
123.83
123.77
123.71
123.57
122.56
121.14
118.72
61.28
45.64
45.61
45.58
42.91
42.64
42.37
13.99
Br
F
F
O
O
200 190 180 170 160 150 140 130 120 110 100 90 80 70 60 50 40 30 20 10 0 -10
f1 (ppm)

^{1}H, ^{19}F and ^{13}C NMR spectra of compound 3u

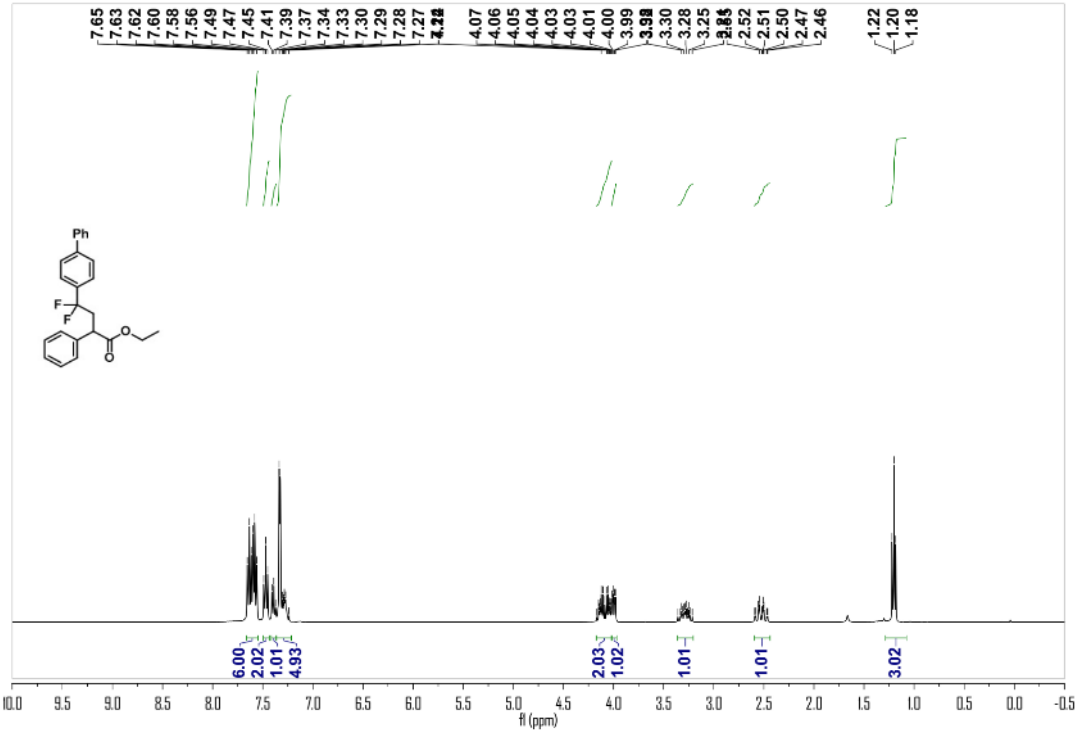

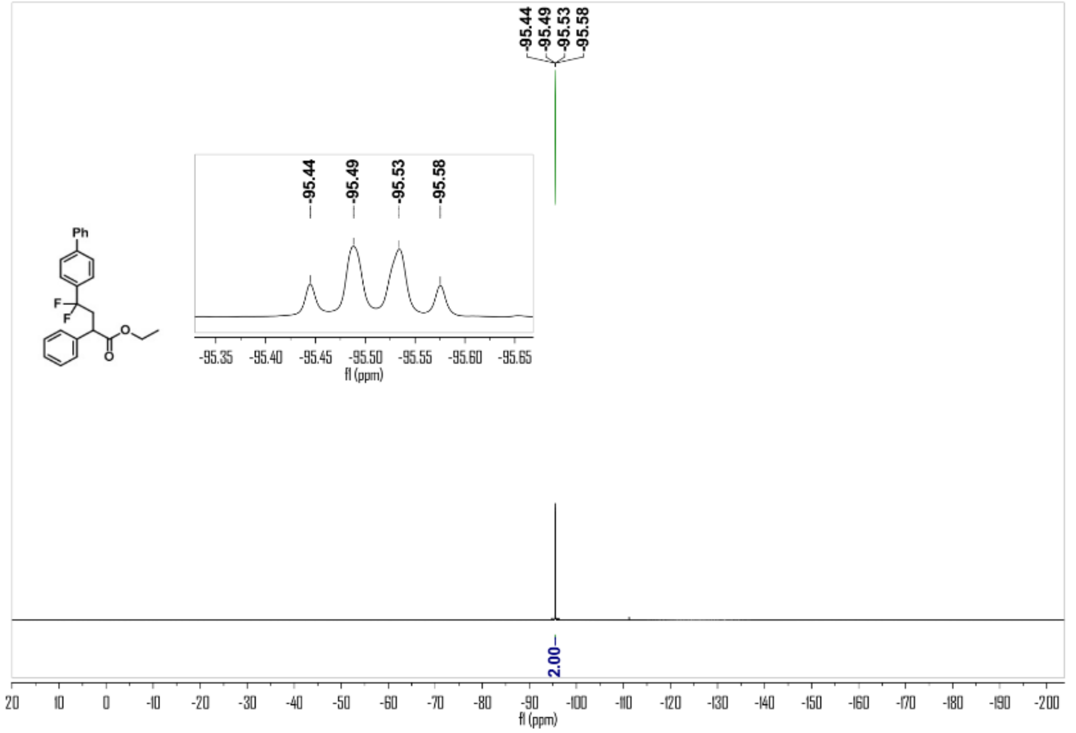

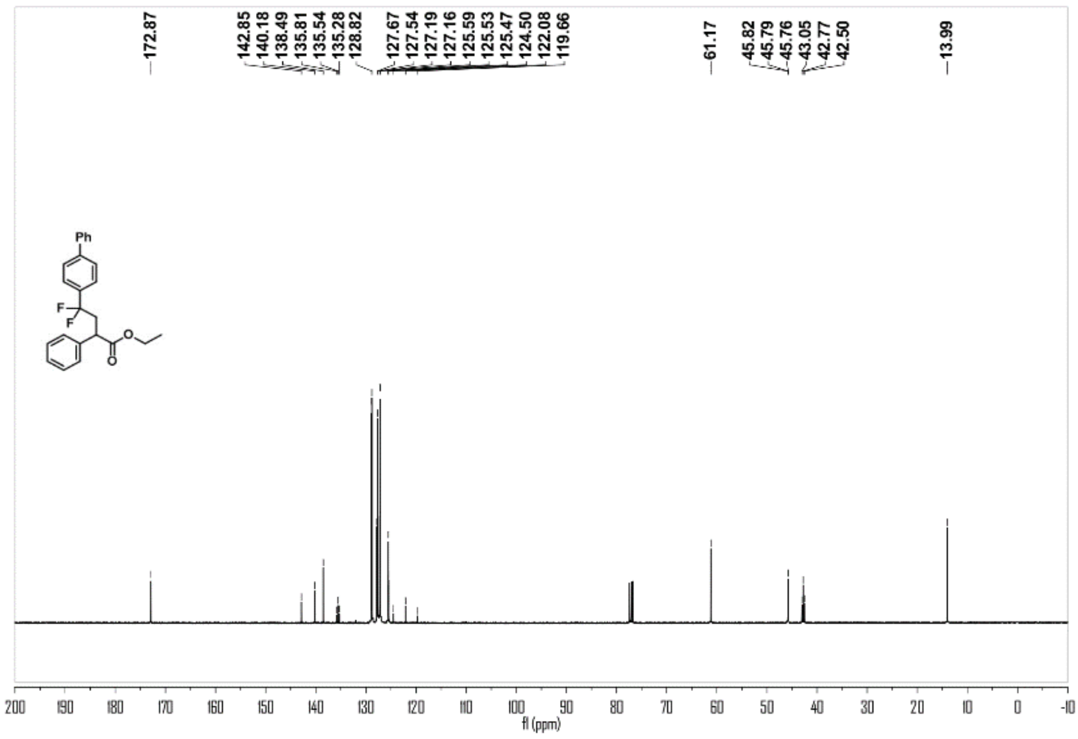

^{1}H, ^{19}F and ^{13}C NMR spectra of compound 3v

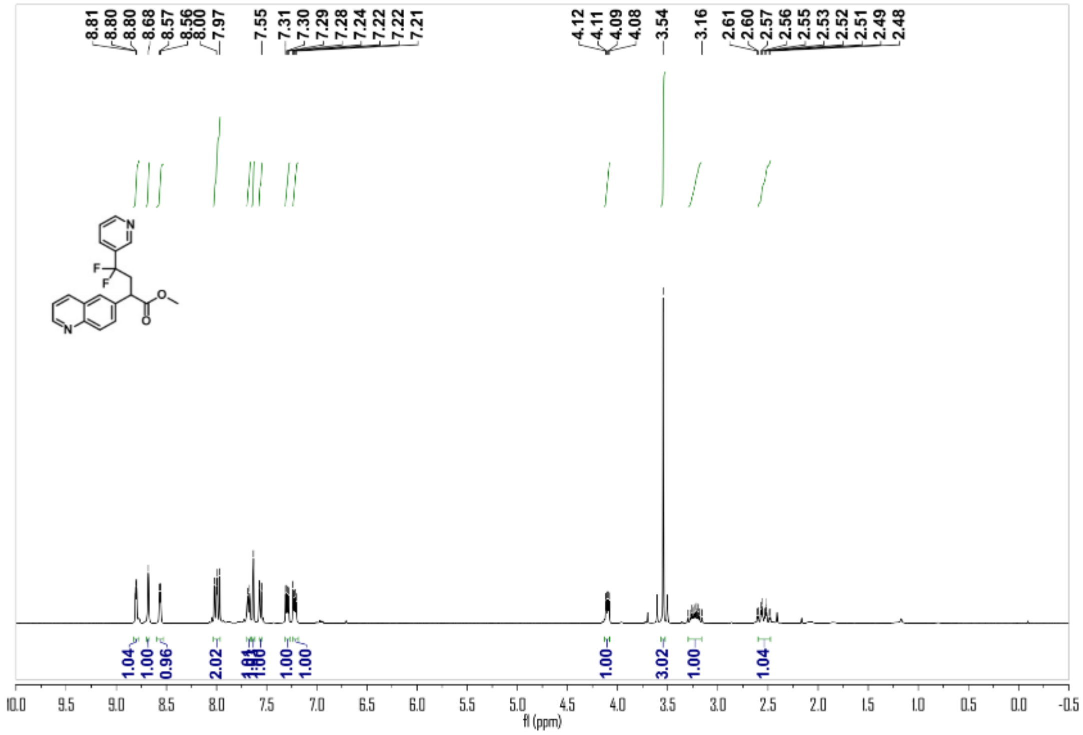

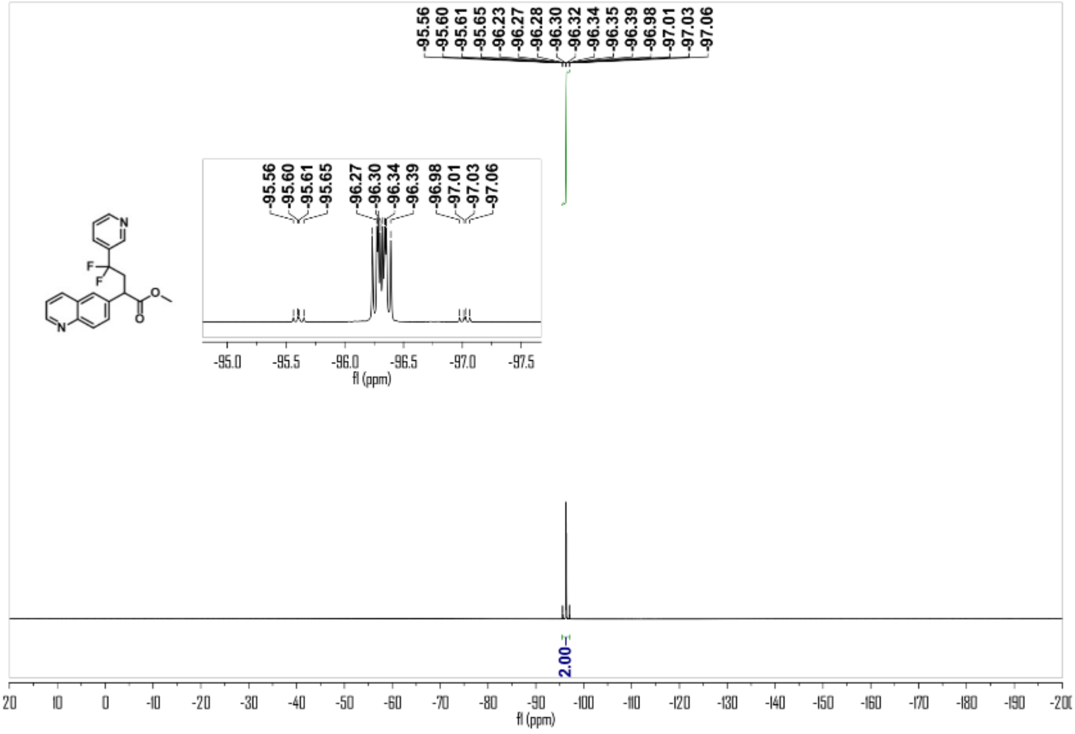

-95.56
-95.60
-95.61
-95.65
-96.23
-96.27
-96.28
-96.30
-96.32
-96.34
-96.35
-96.39
-96.98
-97.01
-97.03
-97.06
2.00
f1 (ppm)

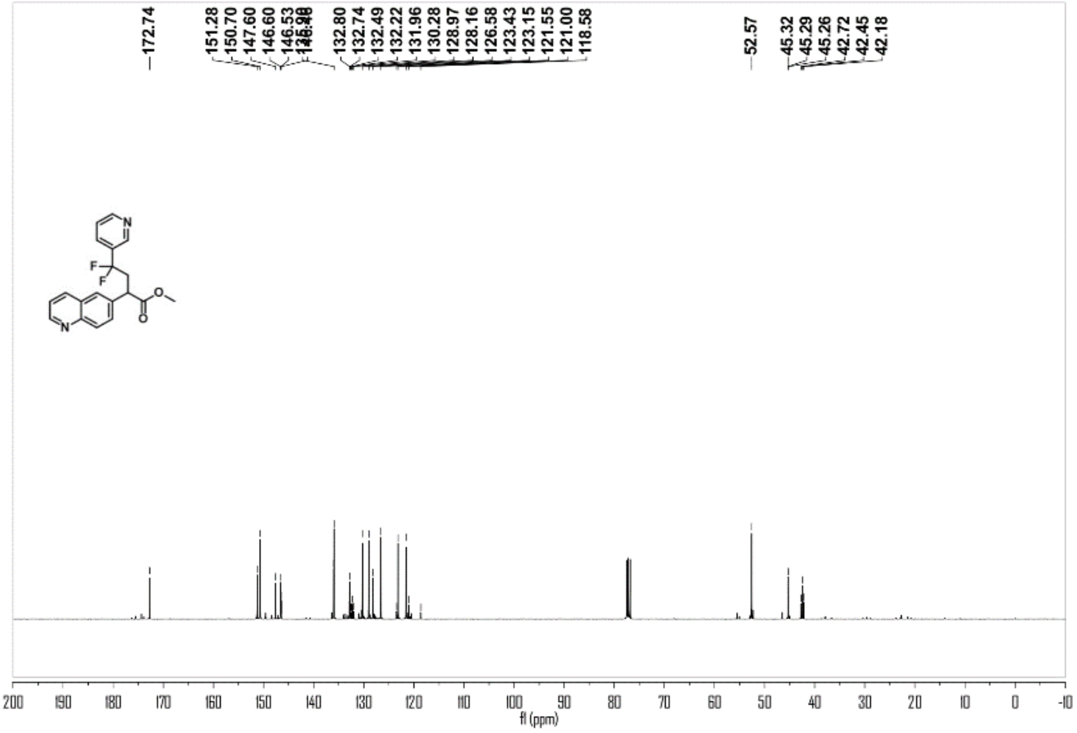

172.74
151.28
150.70
147.60
146.60
146.53
132.80
132.74
132.49
132.22
131.96
130.28
128.97
128.16
126.58
123.43
123.15
121.55
121.00
118.58
52.57
45.32
45.29
45.26
42.72
42.45
42.18
f1 (ppm)

^{1}H, ^{19}F and ^{13}C NMR spectra of compound 3w

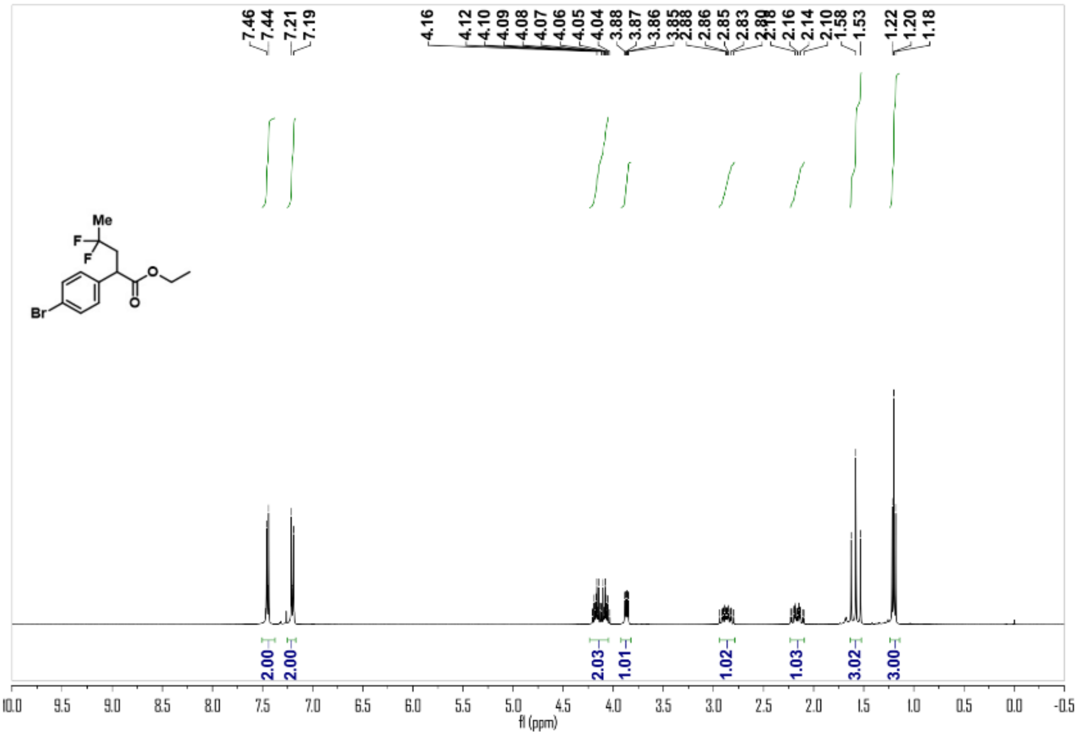

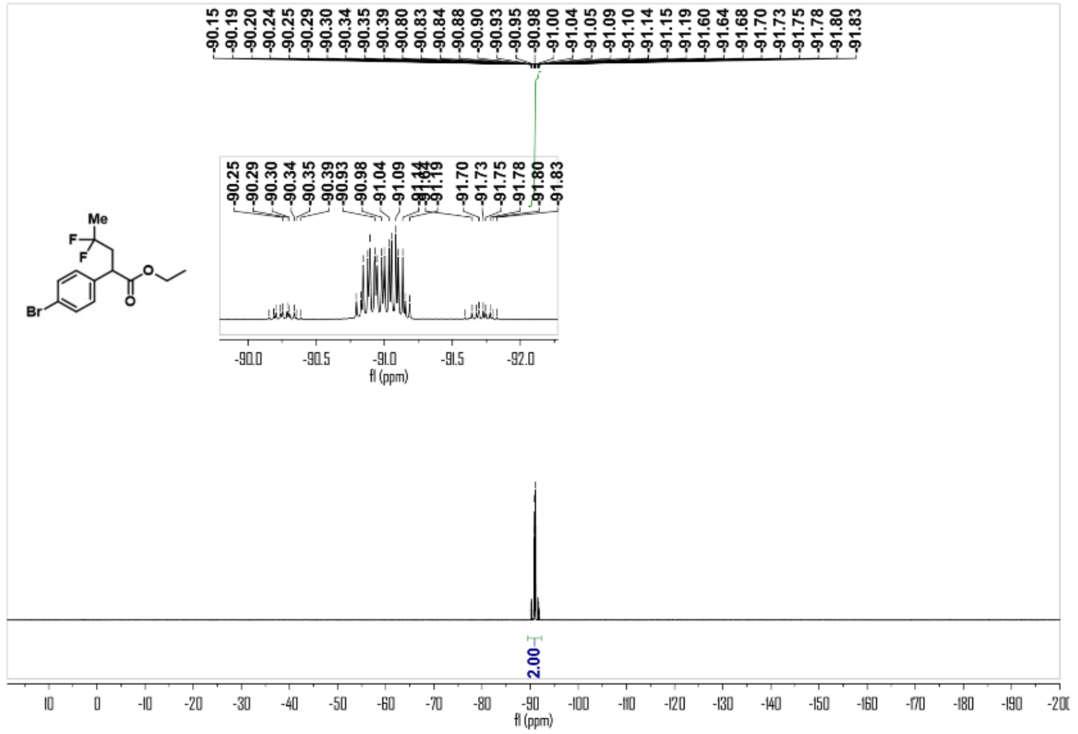

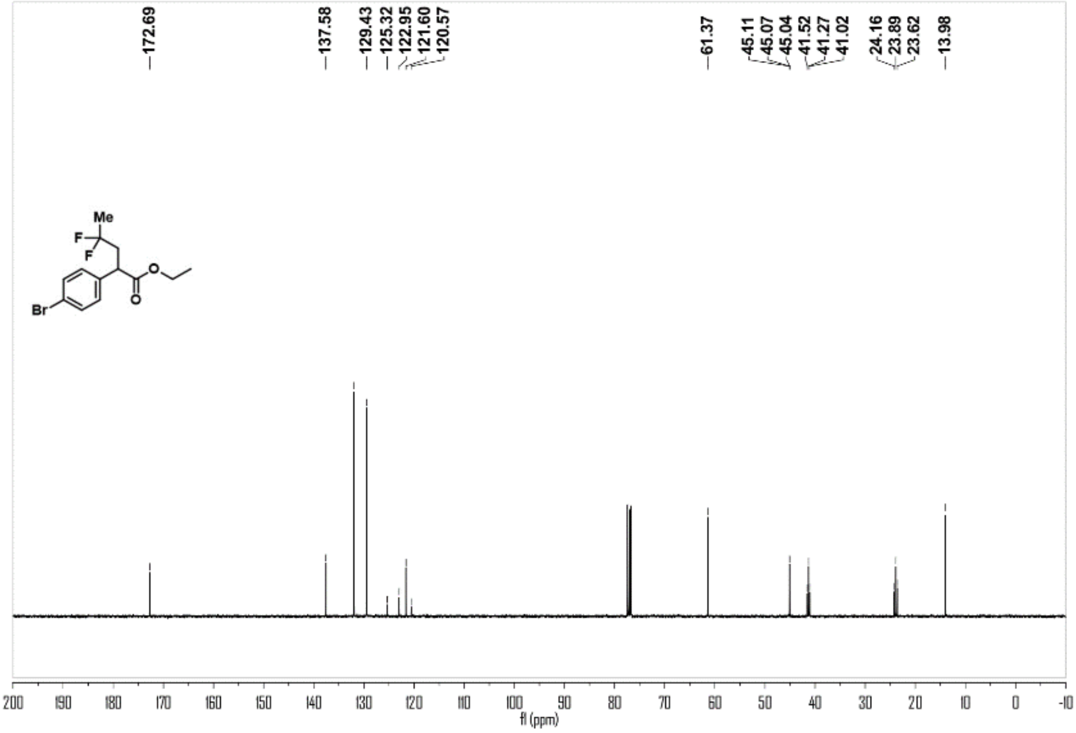

^{1}H, ^{19}F and ^{13}C NMR spectra of compound 3x

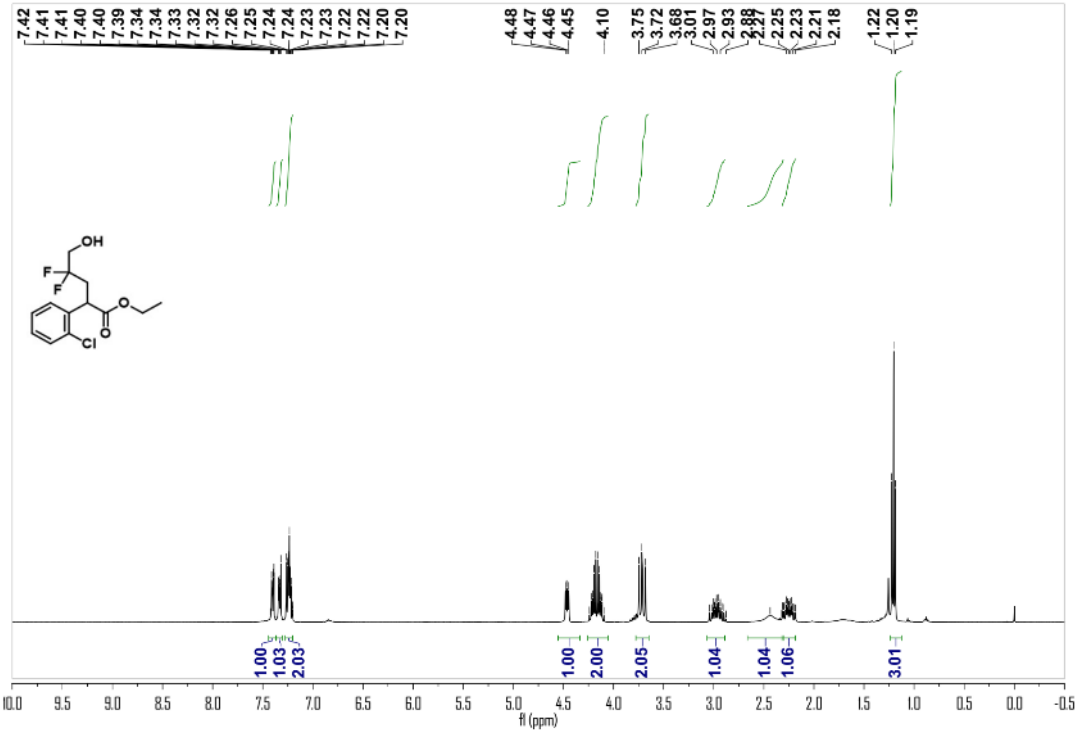

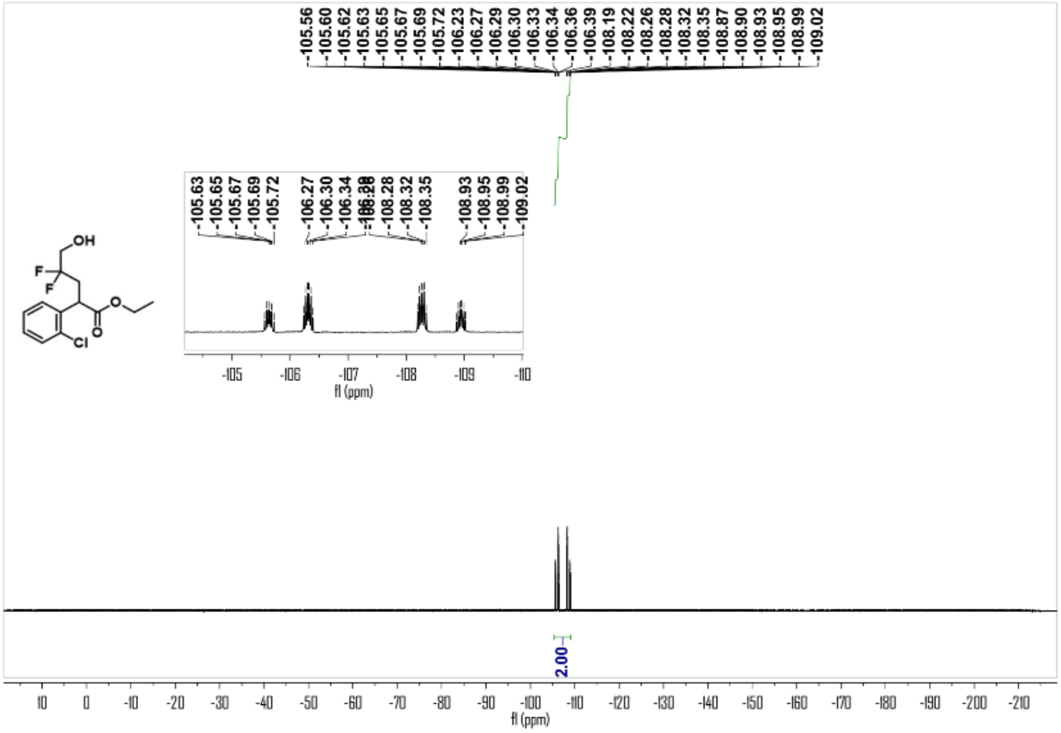
-105.56
-105.60
-105.62
-105.63
-105.65
-105.67
-105.69
-105.72
-106.23
-106.27
-106.29
-106.30
-106.33
-106.34
-106.36
-106.39
-108.19
-108.22
-108.26
-108.28
-108.32
-108.35
-108.87
-108.90
-108.93
-108.95
-108.99
-109.02
OH
F
F
Cl
O
O
2.00
f1 (ppm)

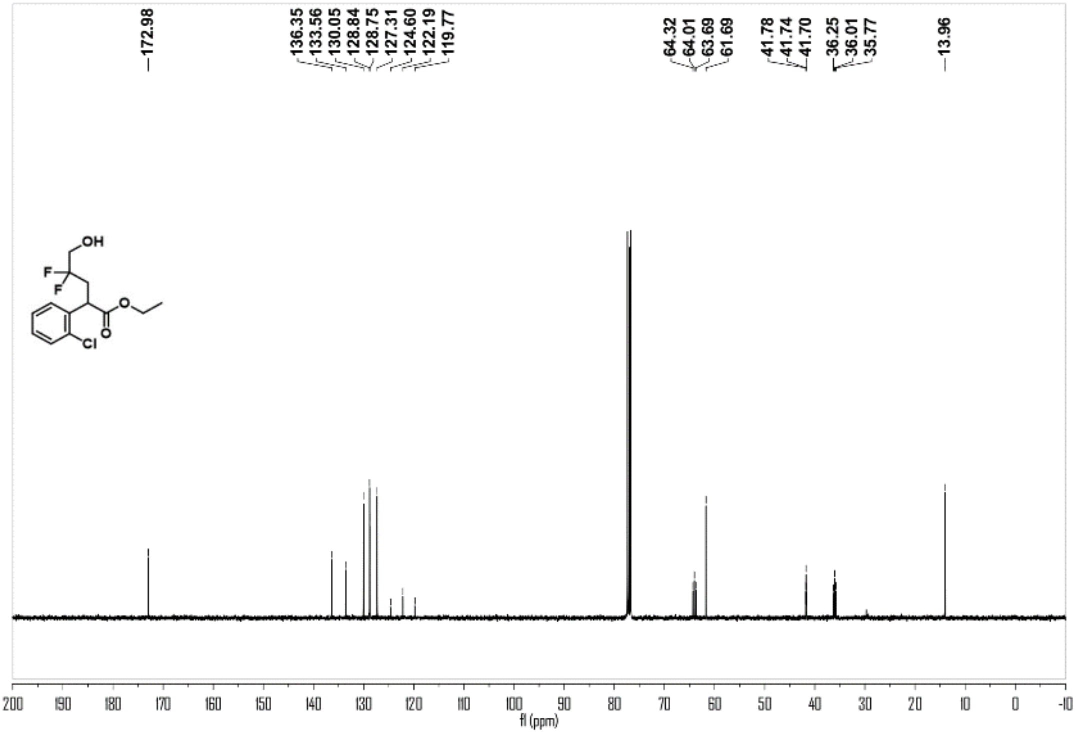
172.98
136.35
133.56
130.05
128.84
128.75
127.31
124.60
122.19
119.77
64.32
64.01
63.69
61.69
41.78
41.74
41.70
36.25
36.01
35.77
13.96
OH
F
F
Cl
O
O
f1 (ppm)

^{1}H, ^{19}F and ^{13}C NMR spectra of compound 3y

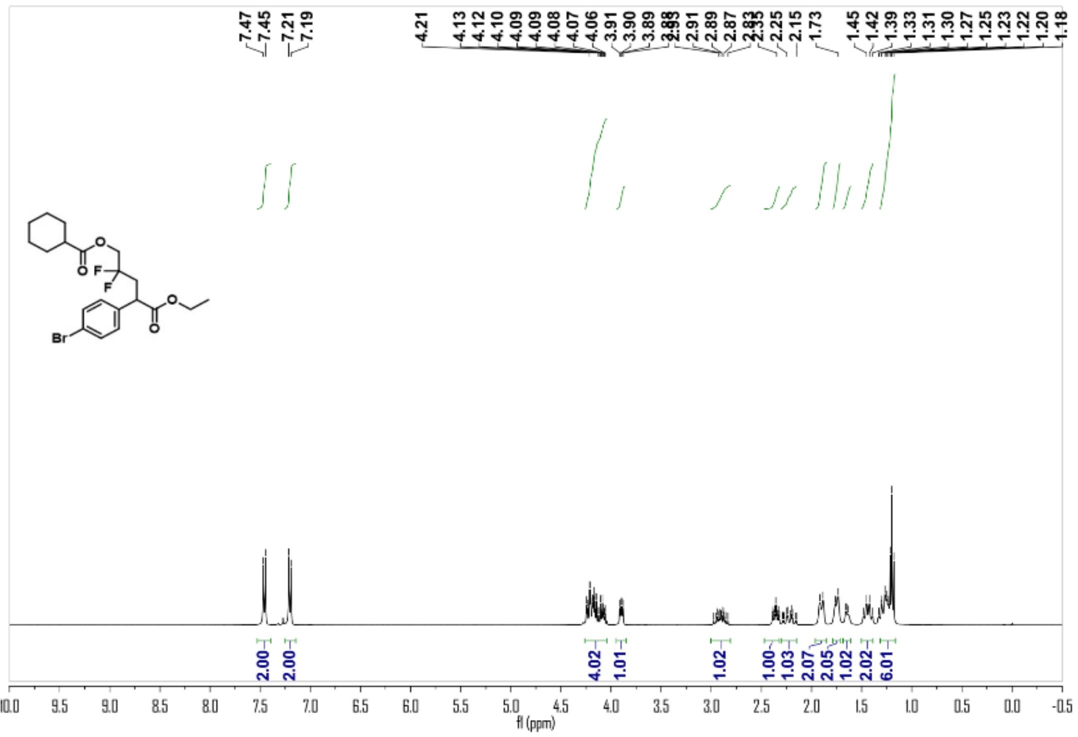

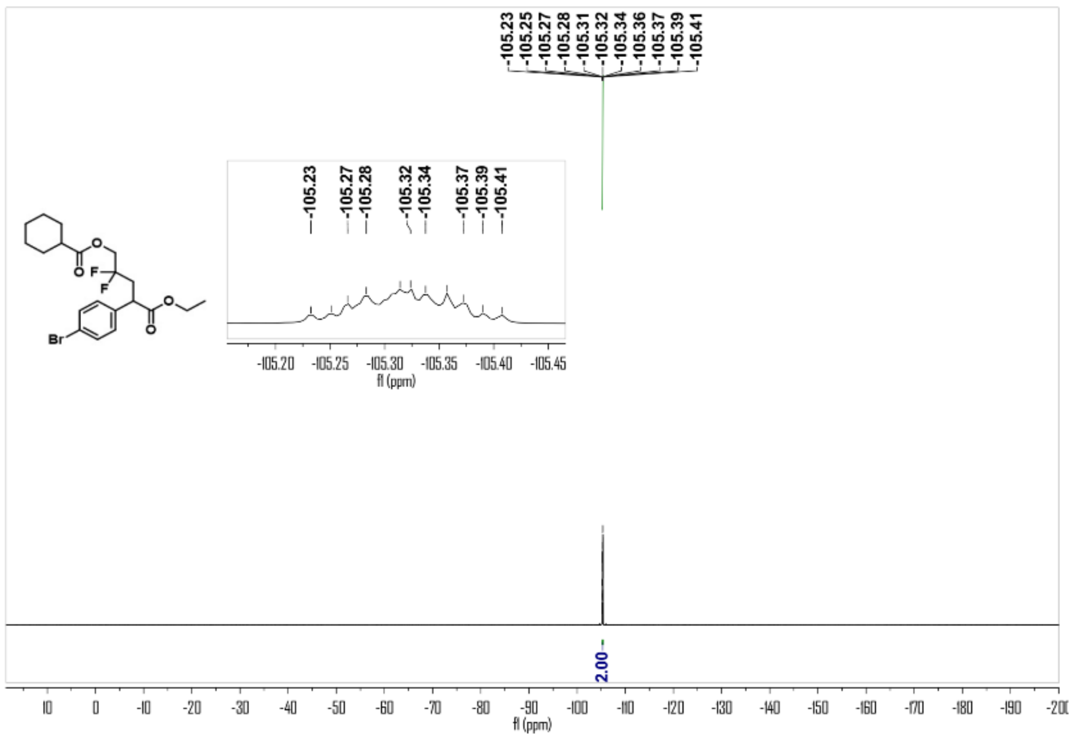

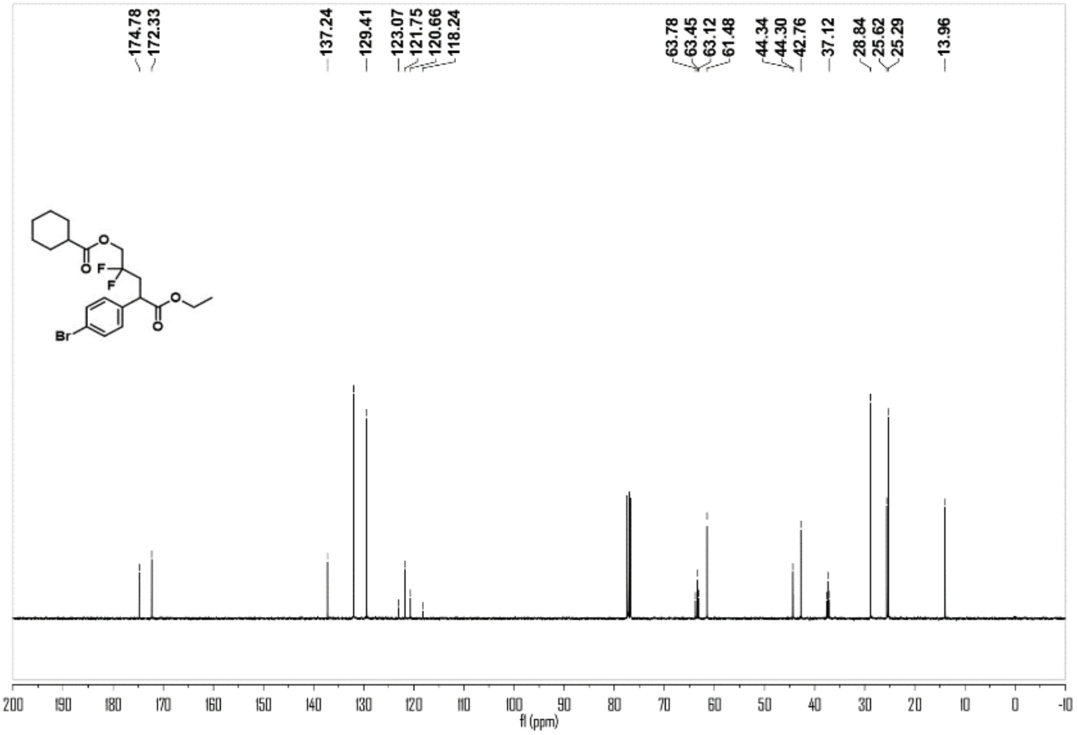

^{1}H, ^{19}F and ^{13}C NMR spectra of compound 3z

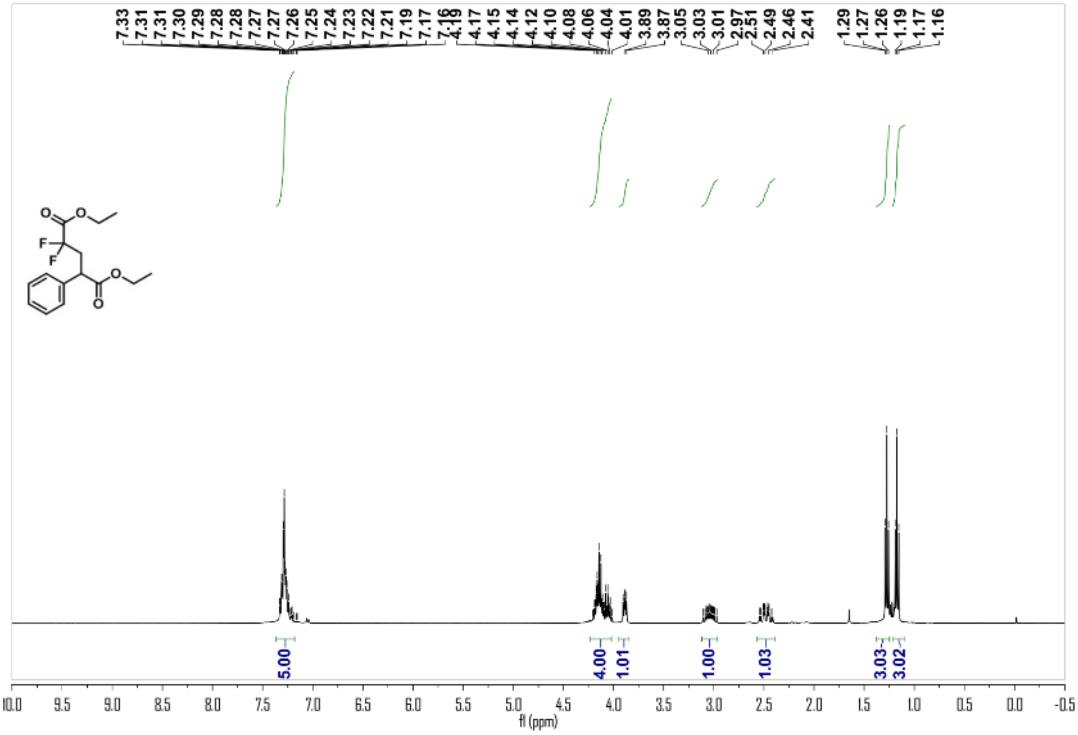

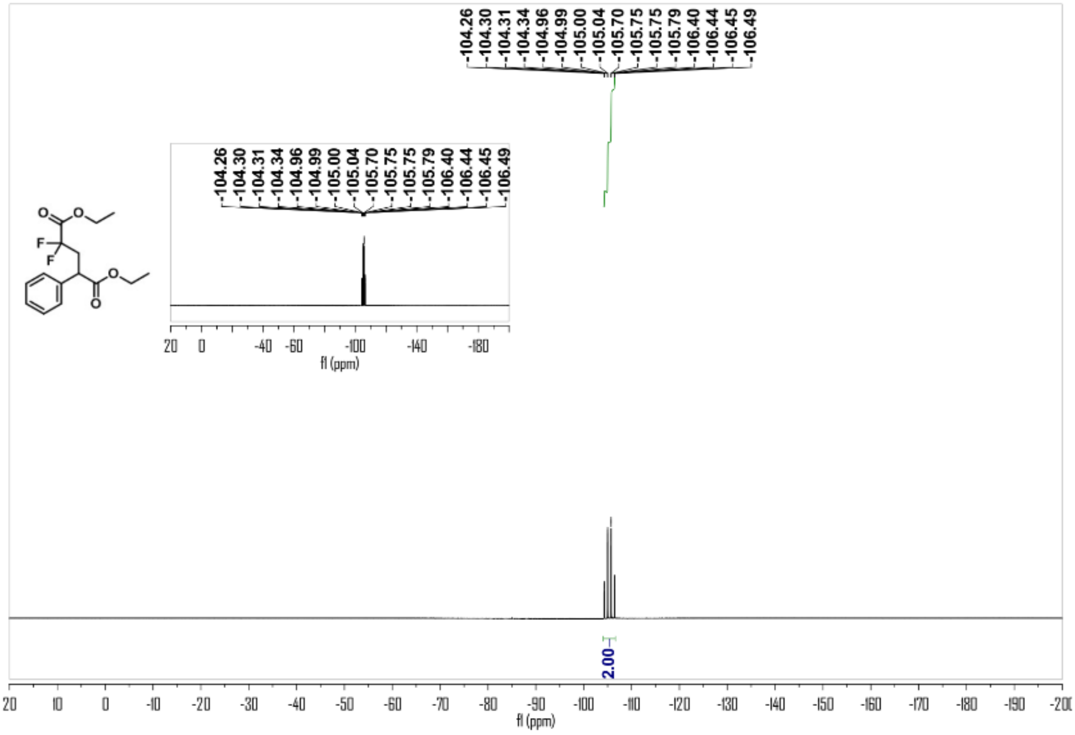
-104.26
-104.30
-104.31
-104.34
-104.96
-104.99
-105.00
-105.04
-105.70
-105.75
-105.75
-105.79
-106.40
-106.44
-106.45
-106.49
2.00
fl (ppm)

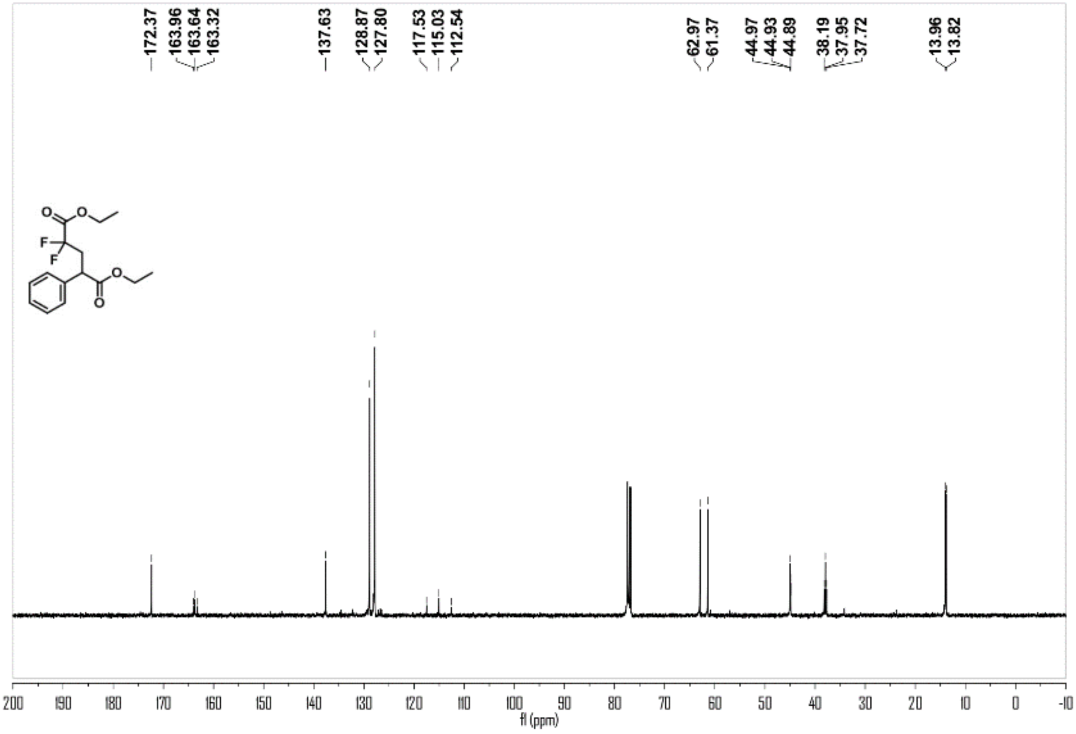
172.37
163.96
163.64
163.32
137.63
128.87
127.80
117.53
115.03
112.54
62.97
61.37
44.97
44.93
44.89
38.19
37.95
37.72
13.96
13.82
fl (ppm)

^{1}H, ^{19}F and ^{13}C NMR spectra of compound 3aa

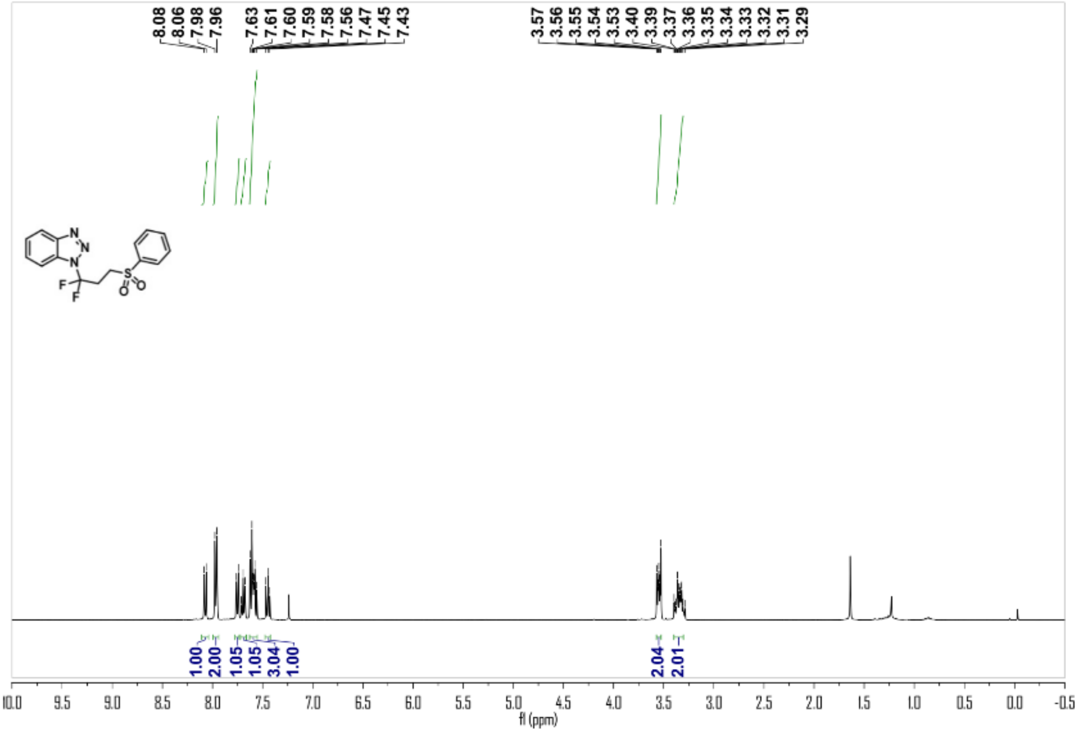

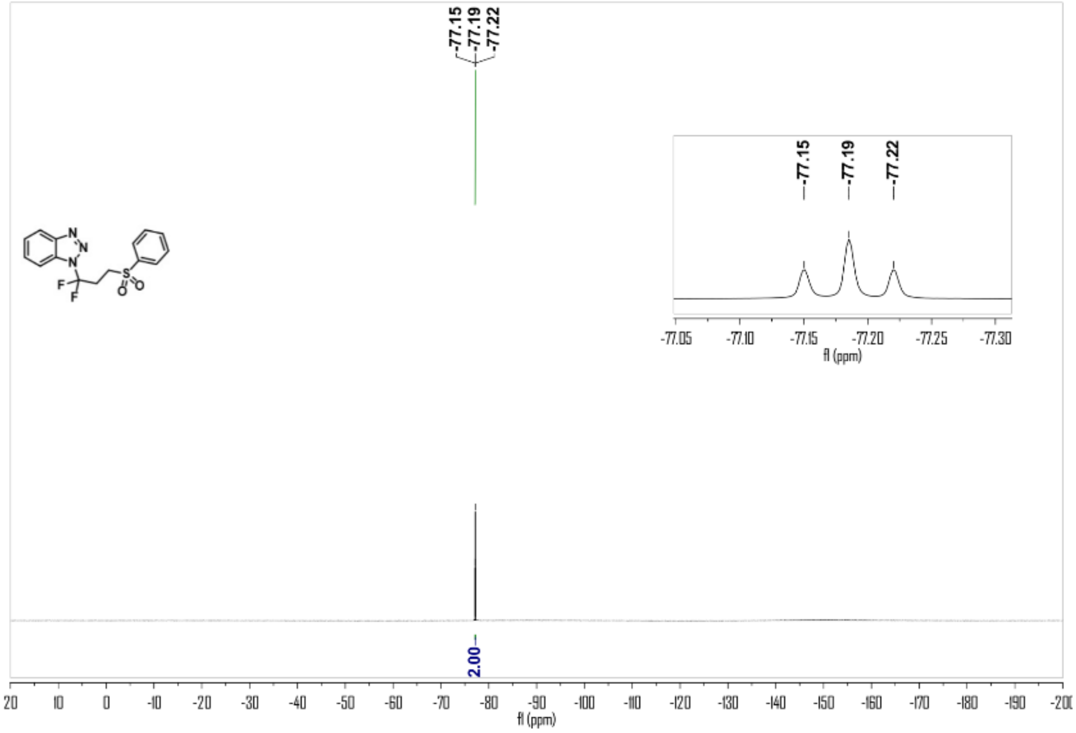

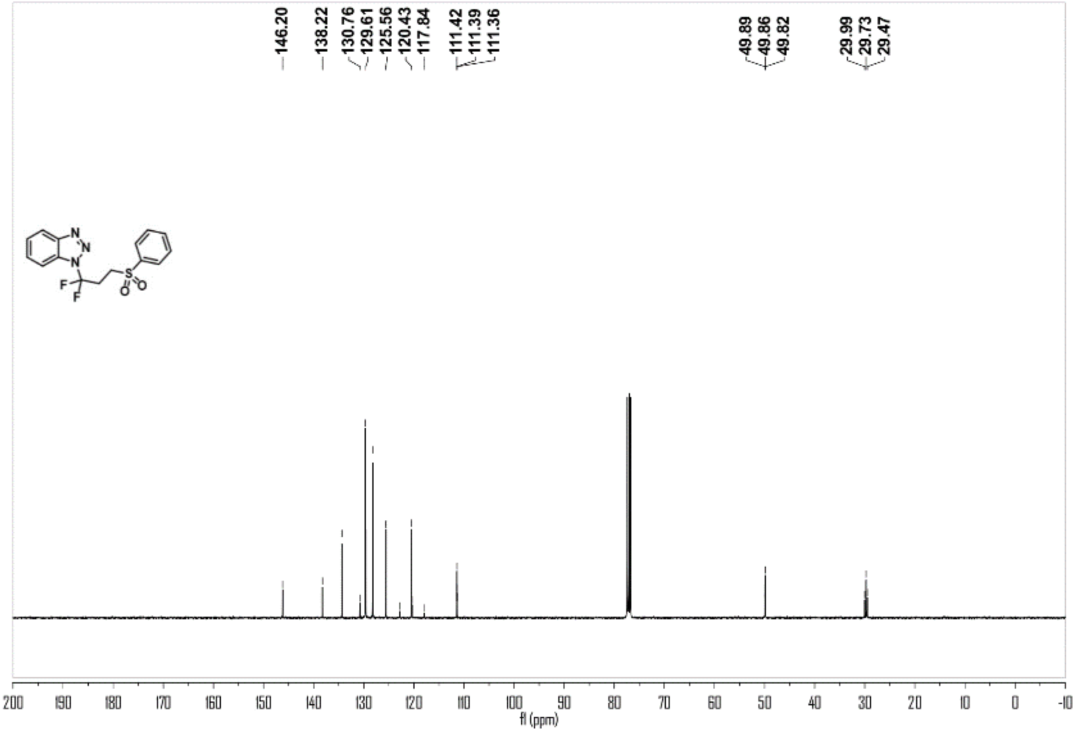

^{1}H, ^{19}F and ^{13}C NMR spectra of compound 3ab

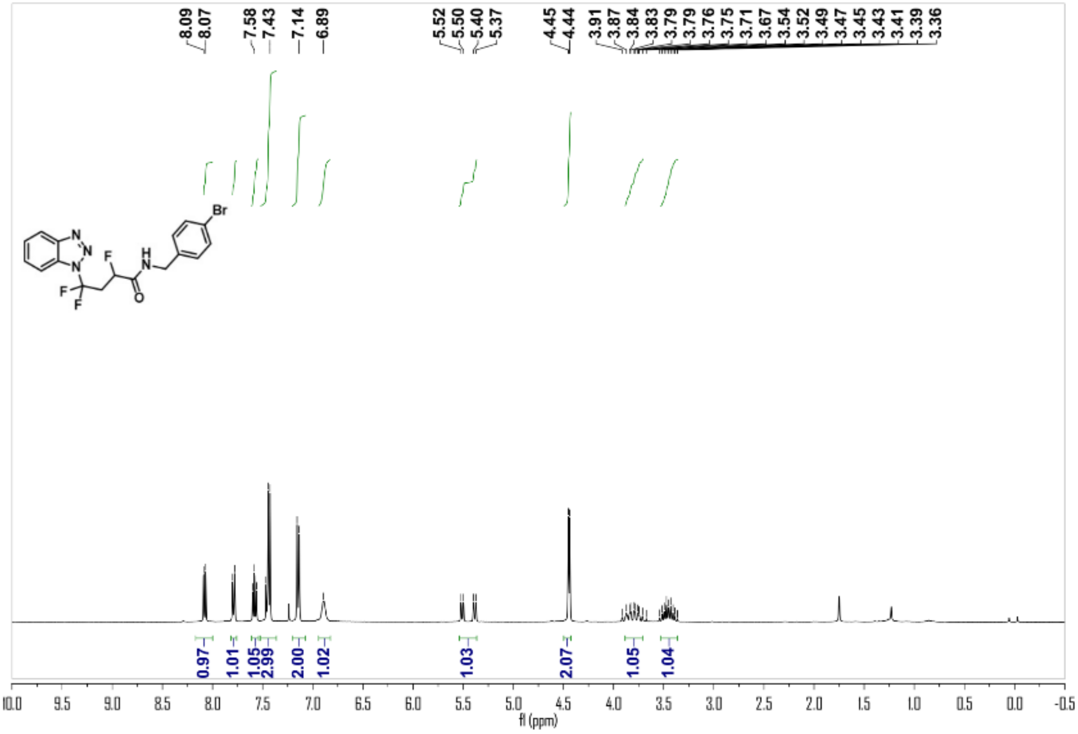

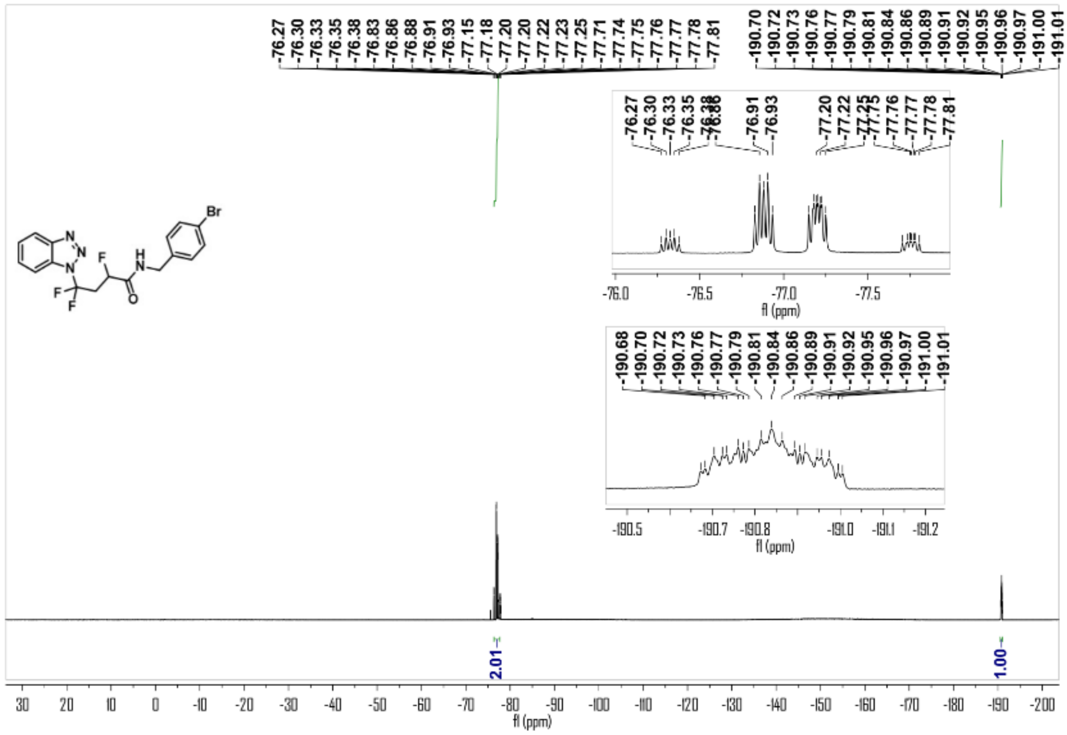

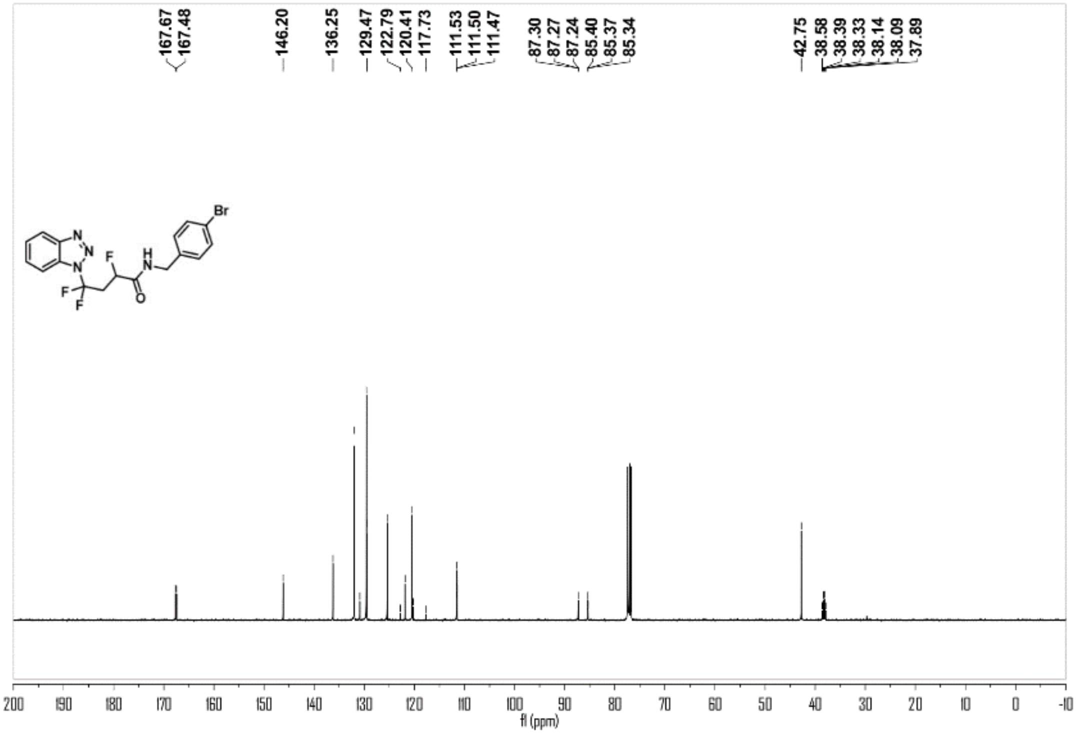

^{1}H, ^{19}F and ^{13}C NMR spectra of compound 3ac

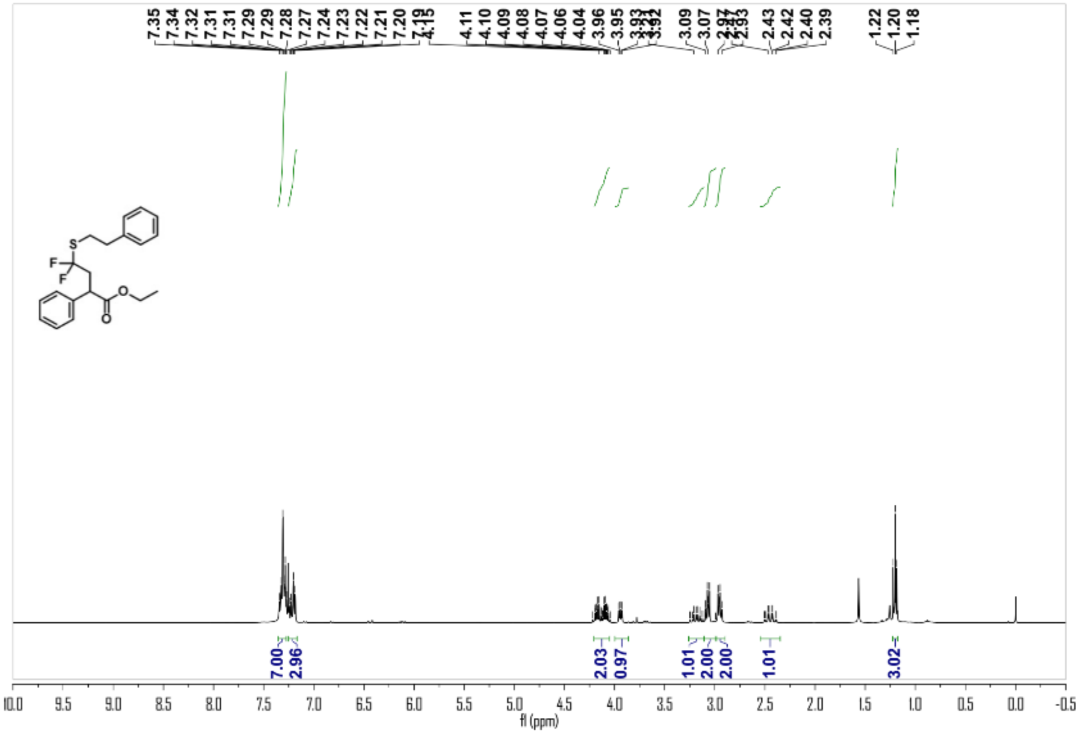

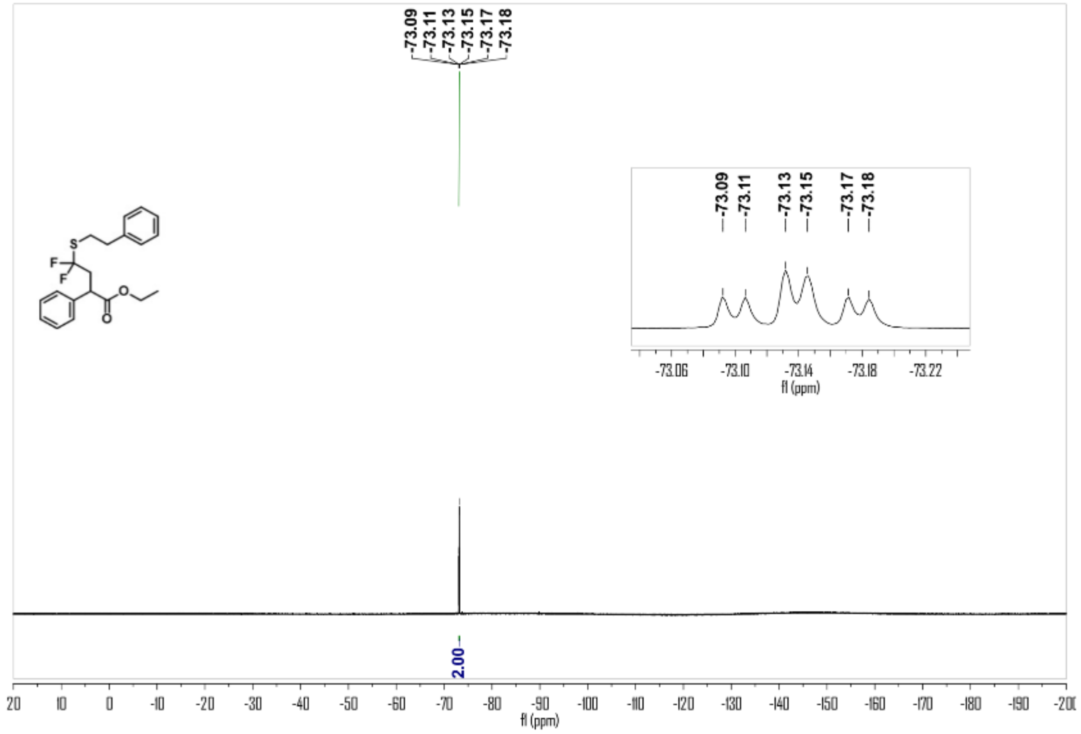

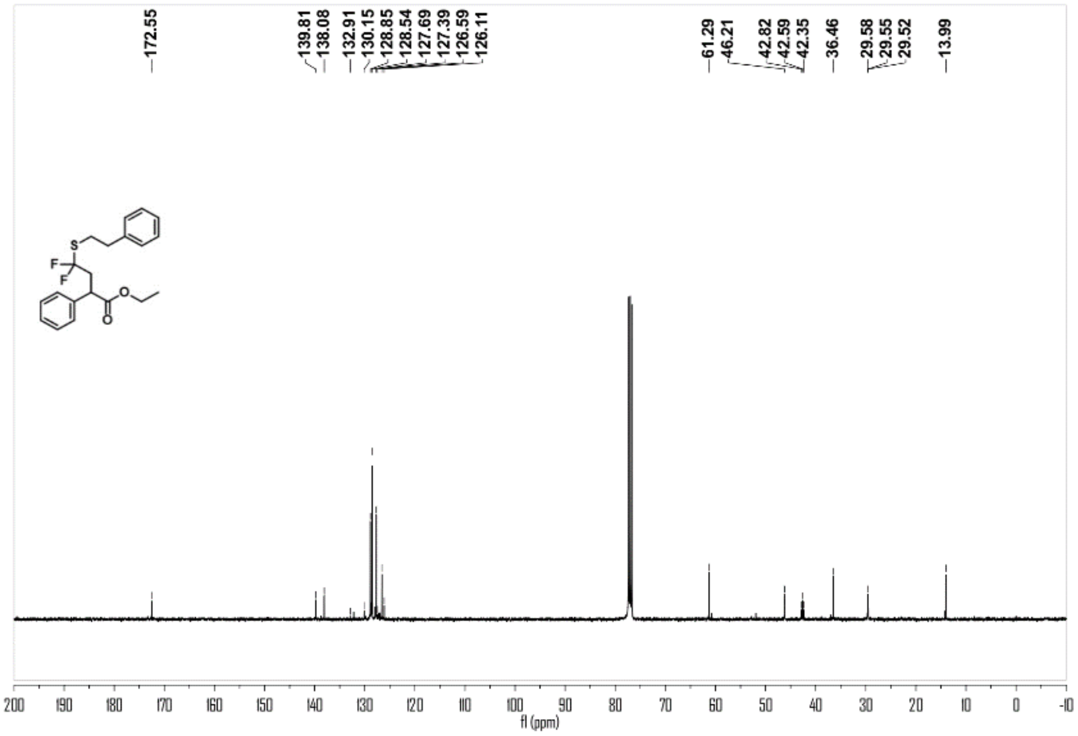

¹H, ¹⁹F and ¹³C NMR spectra of compound 3ad

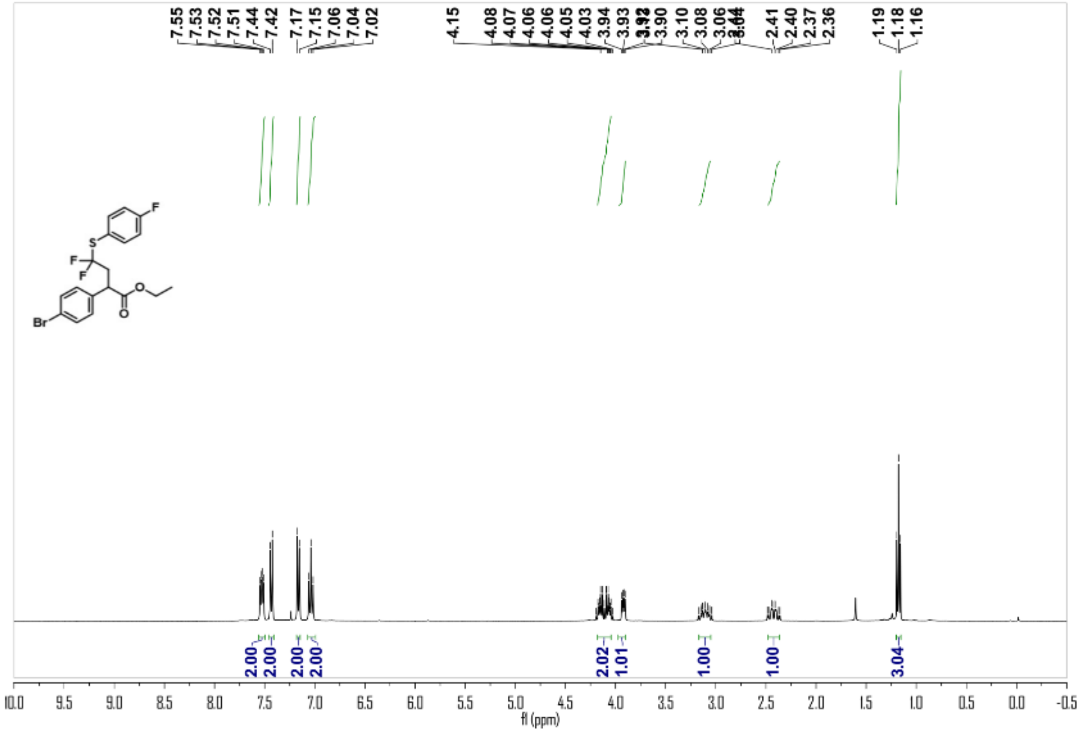

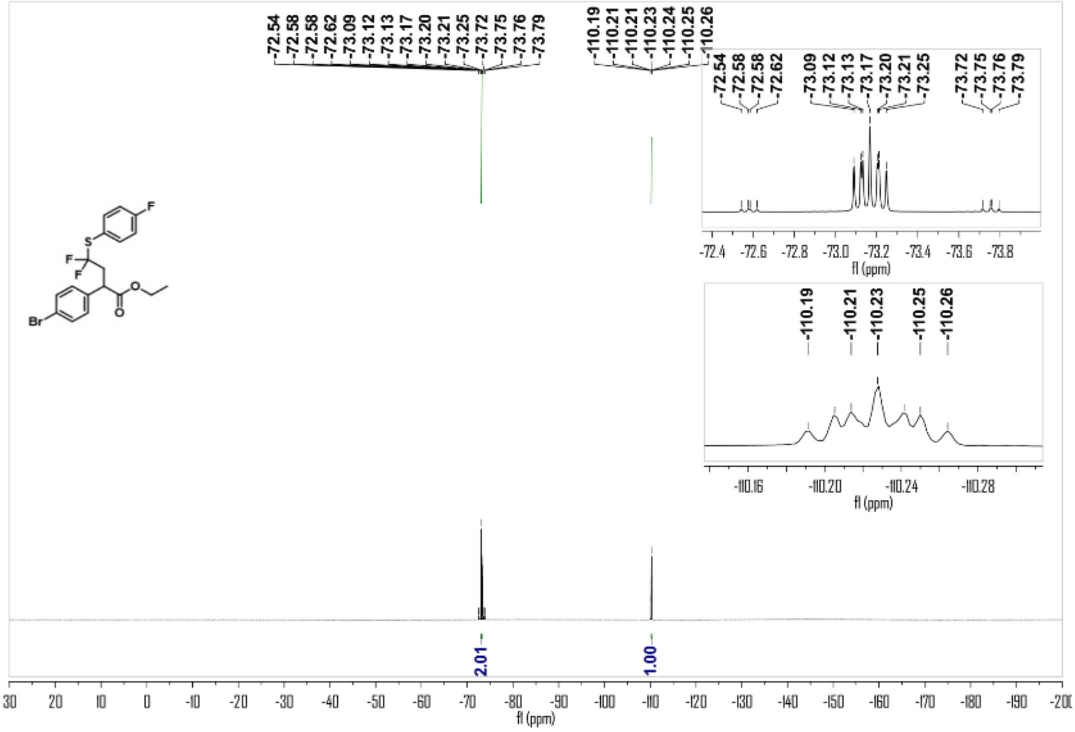
-72.54
-72.58
-72.58
-72.62
-73.09
-73.12
-73.13
-73.17
-73.20
-73.21
-73.25
-73.72
-73.75
-73.76
-73.79
-110.19
-110.21
-110.21
-110.23
-110.24
-110.25
-110.26
2.01
1.00
fl (ppm)

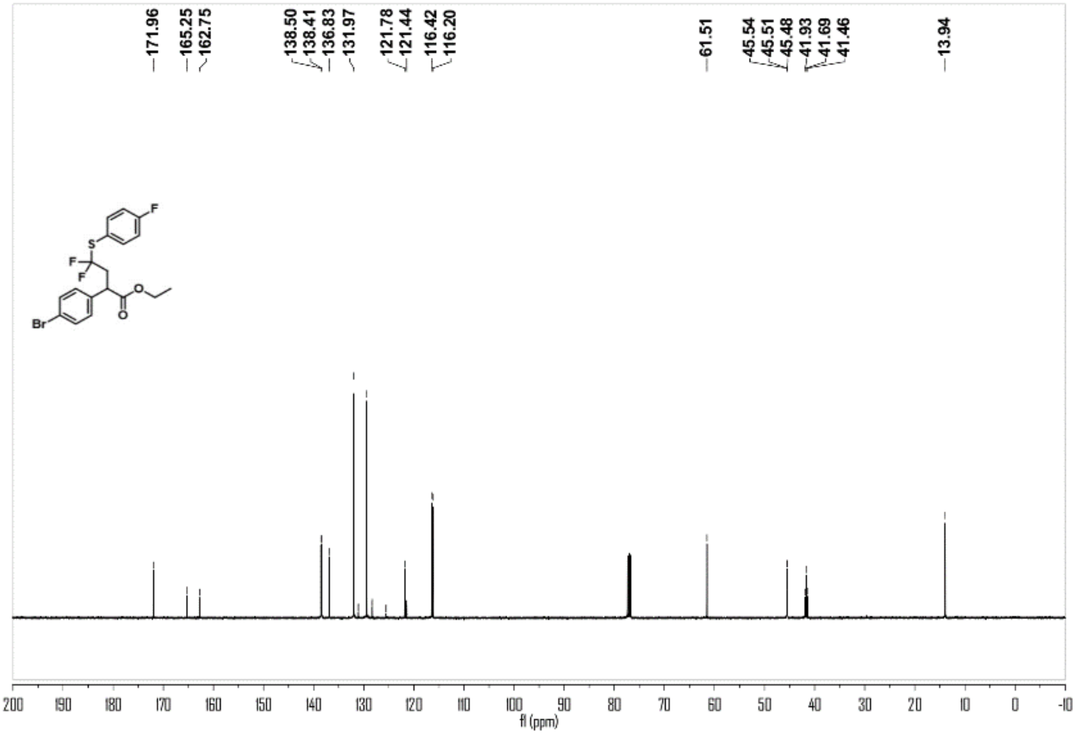
171.96
165.25
162.75
138.50
138.41
136.83
131.97
121.78
121.44
116.42
116.20
61.51
45.54
45.51
45.48
41.93
41.69
41.46
13.94
fl (ppm)

^{1}H, ^{19}F and ^{13}C NMR spectra of compound 3ae

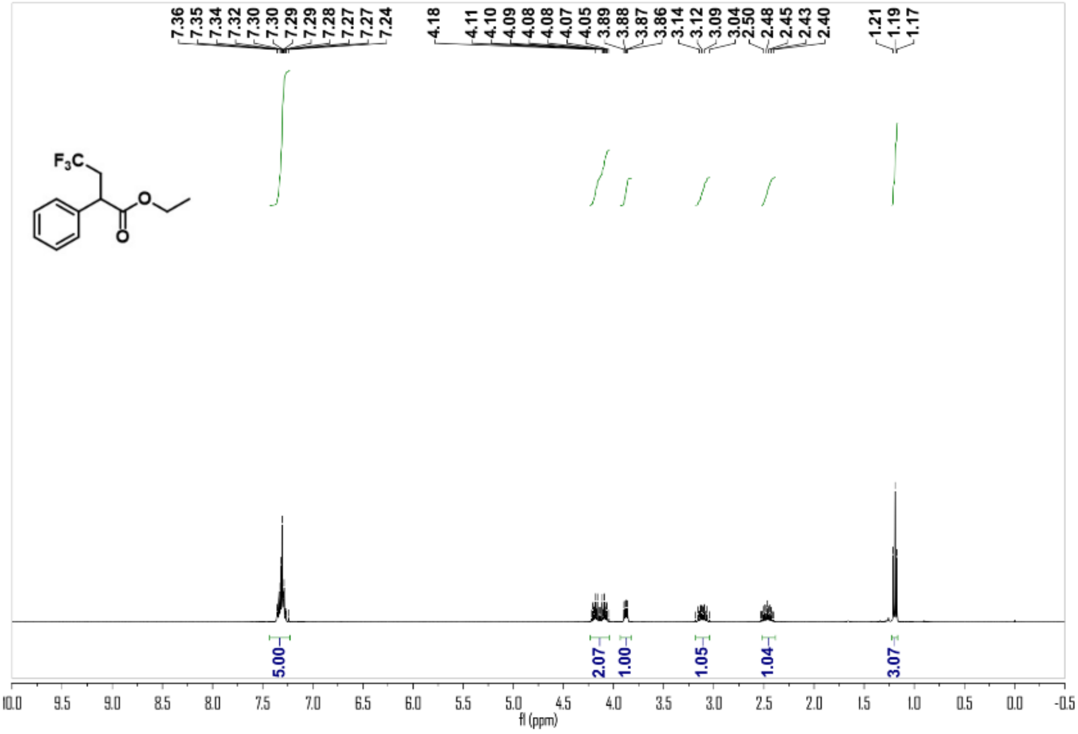

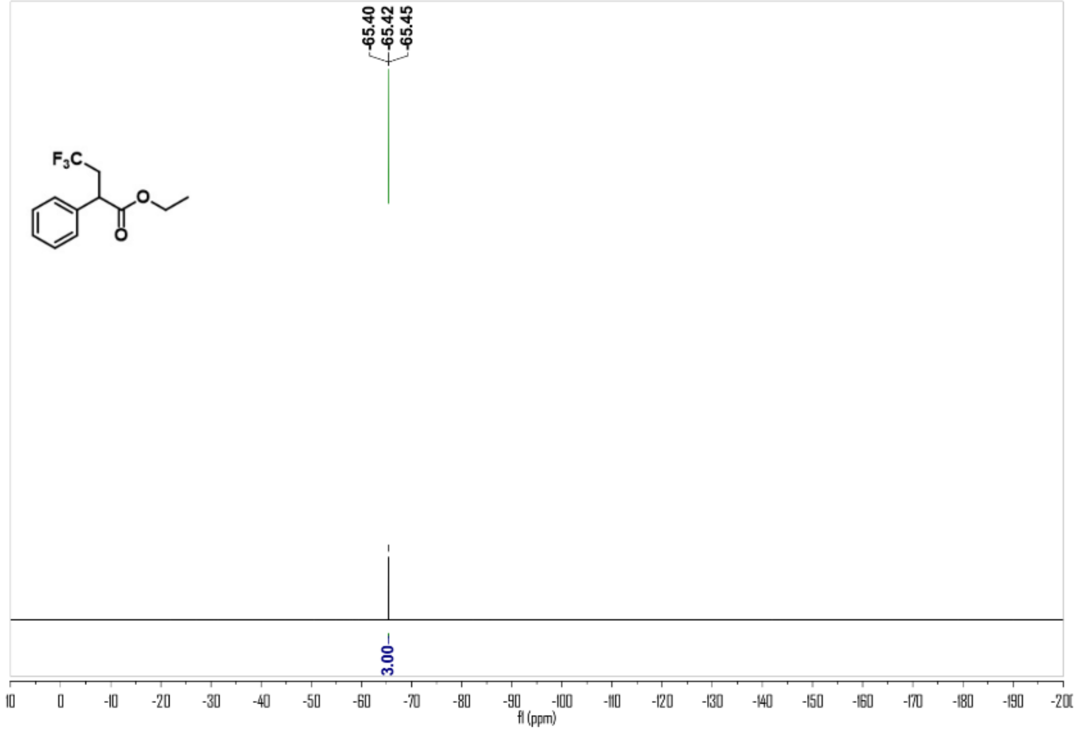

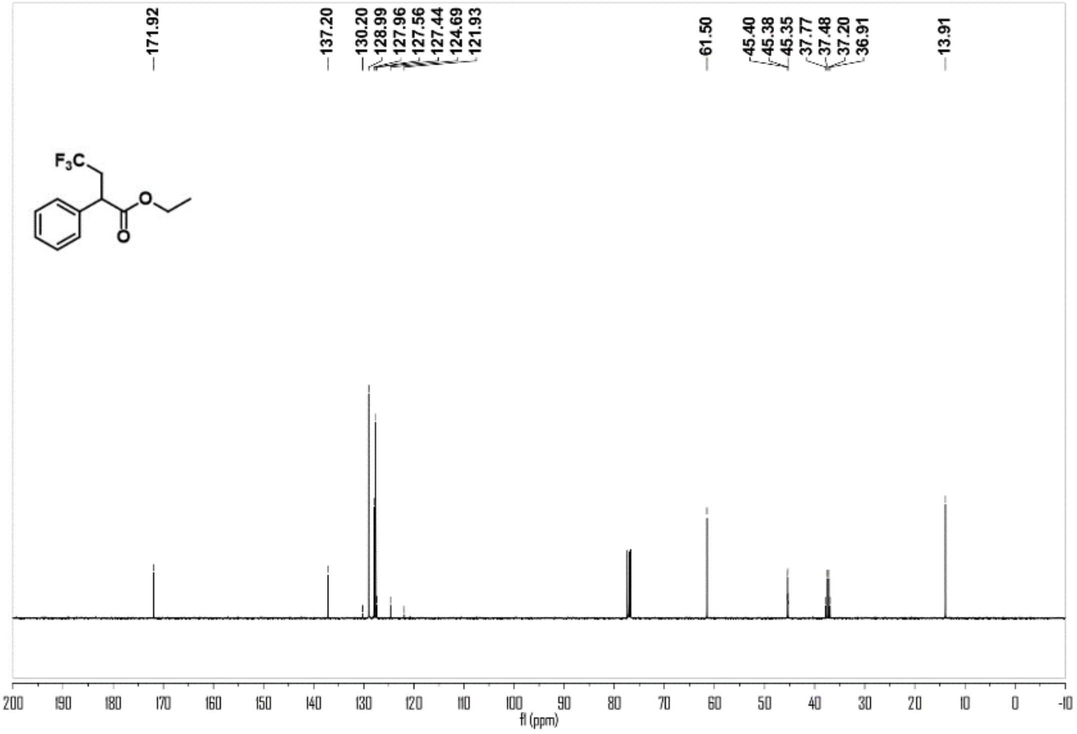

^{1}H, ^{19}F and ^{13}C NMR spectra of compound 3af

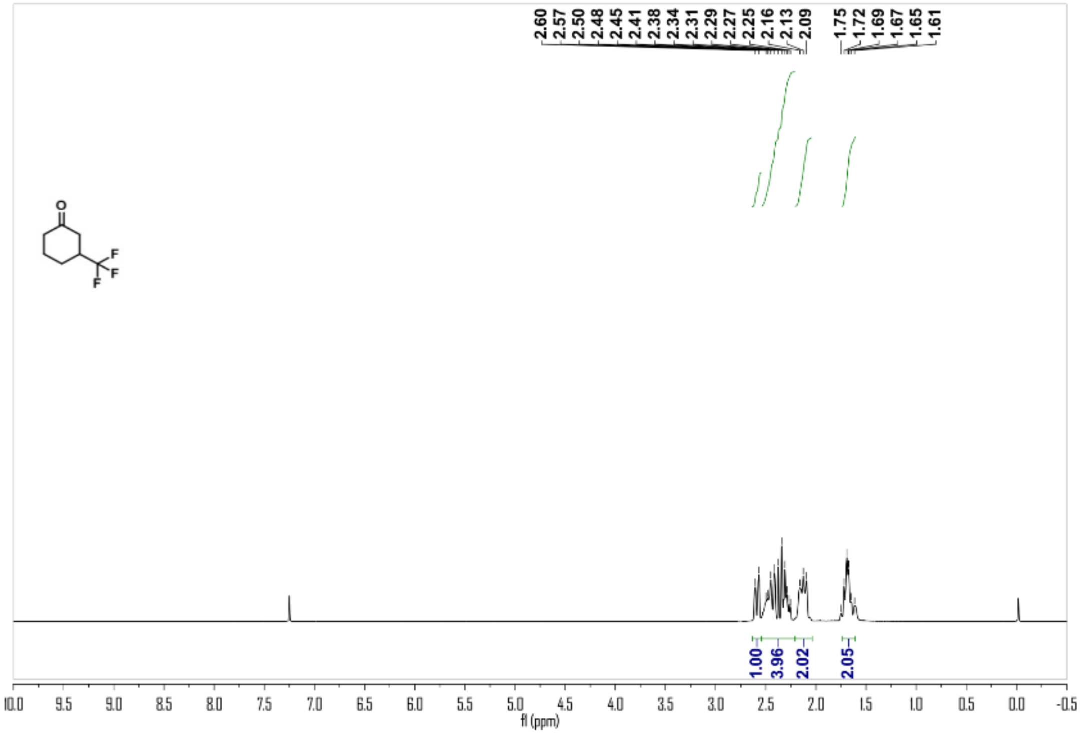

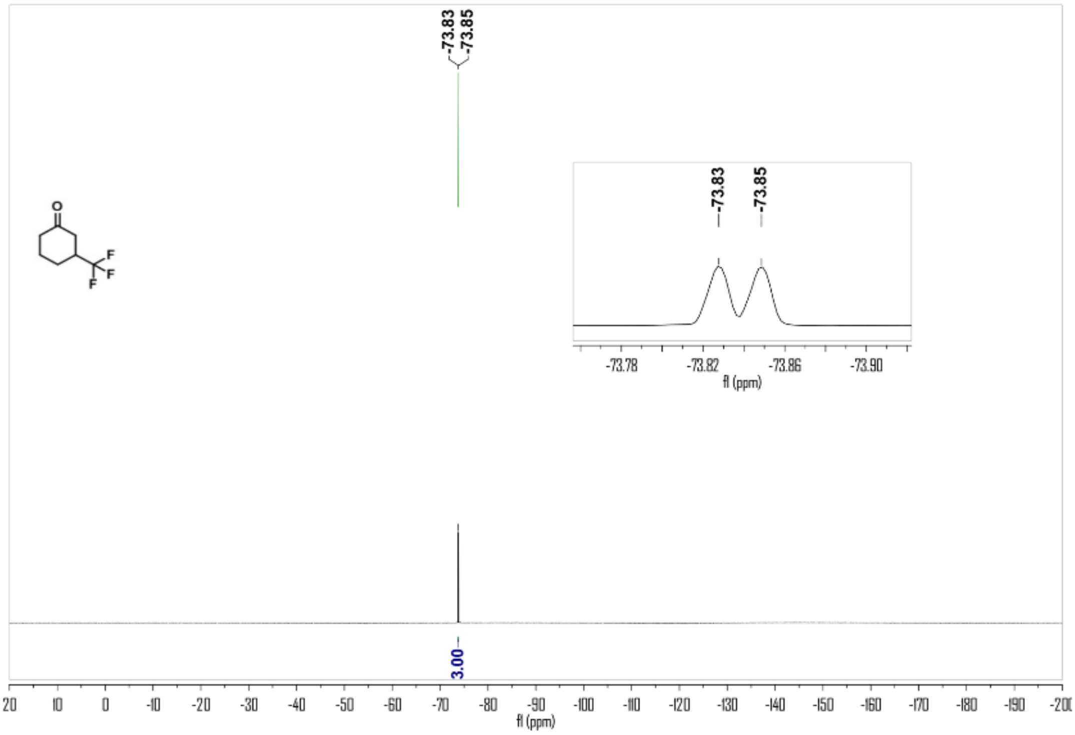
-73.83
-73.85
F
F
F
3.00
-73.78
-73.82
-73.86
-73.90
f1 (ppm)
20
10
0
-10
-20
-30
-40
-50
-60
-70
-80
-90
-100
-110
-120
-130
-140
-150
-160
-170
-180
-190
f1 (ppm)

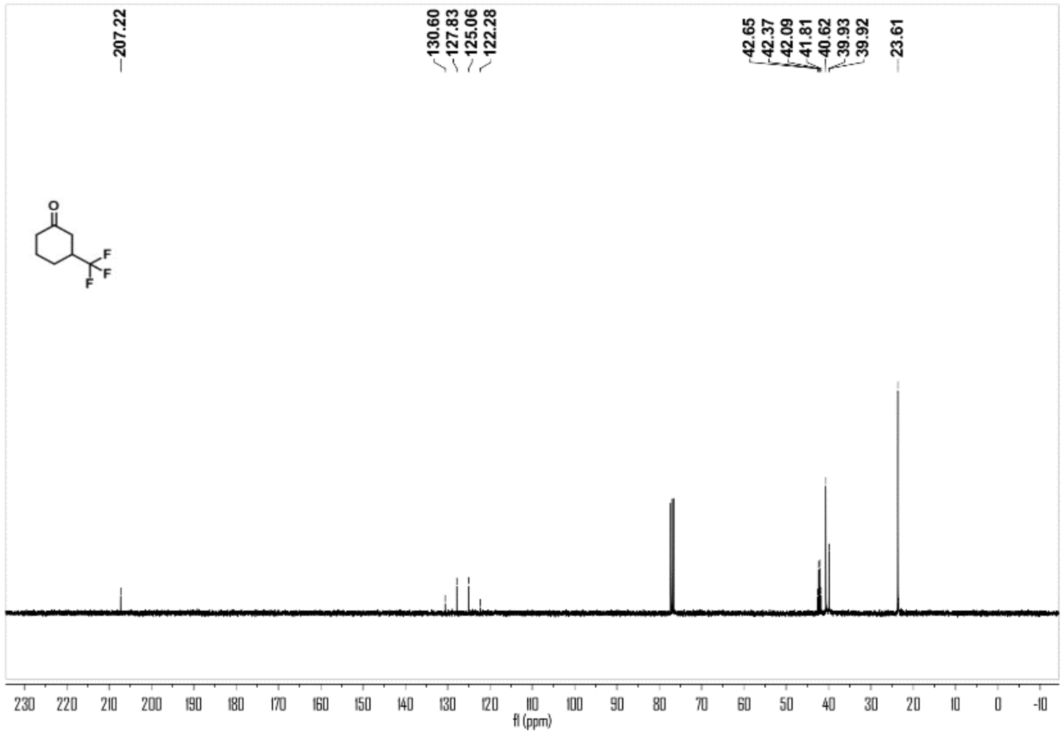
207.22
130.60
127.83
125.06
122.28
42.65
42.37
42.09
41.81
40.62
39.93
39.92
23.61
O
F
F
F
230
220
210
200
190
180
170
160
150
140
130
120
110
100
90
80
70
60
50
40
30
20
10
0
-10
f1 (ppm)

^{1}H, ^{19}F and ^{13}C NMR spectra of compound 3ag

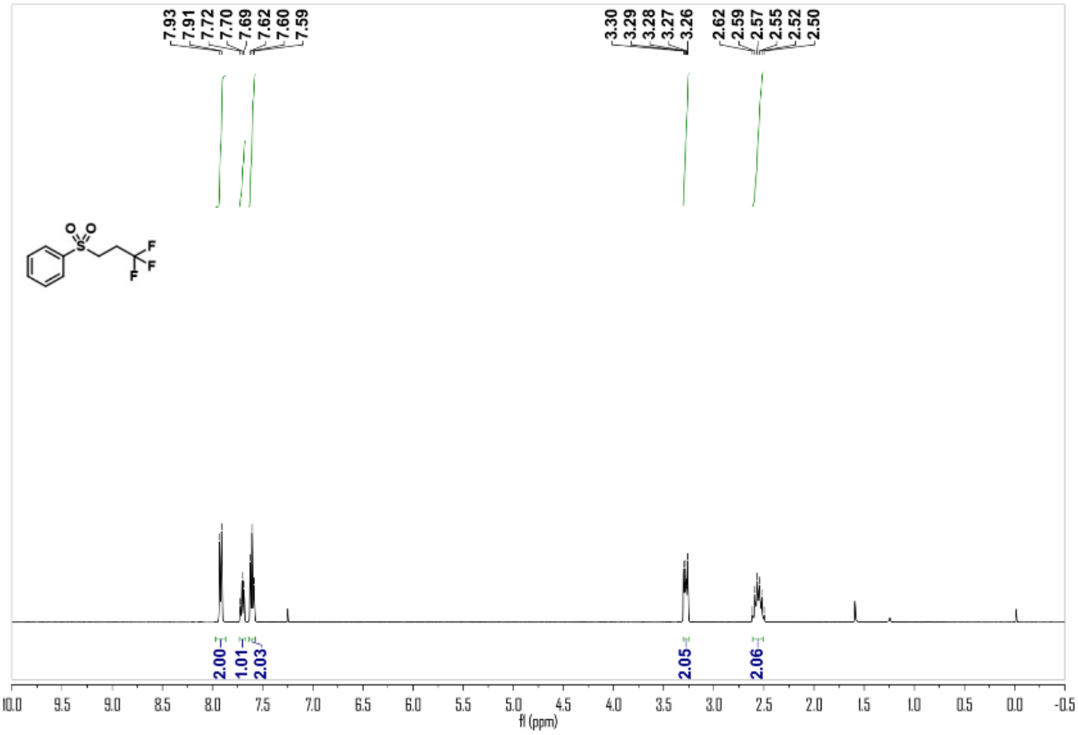

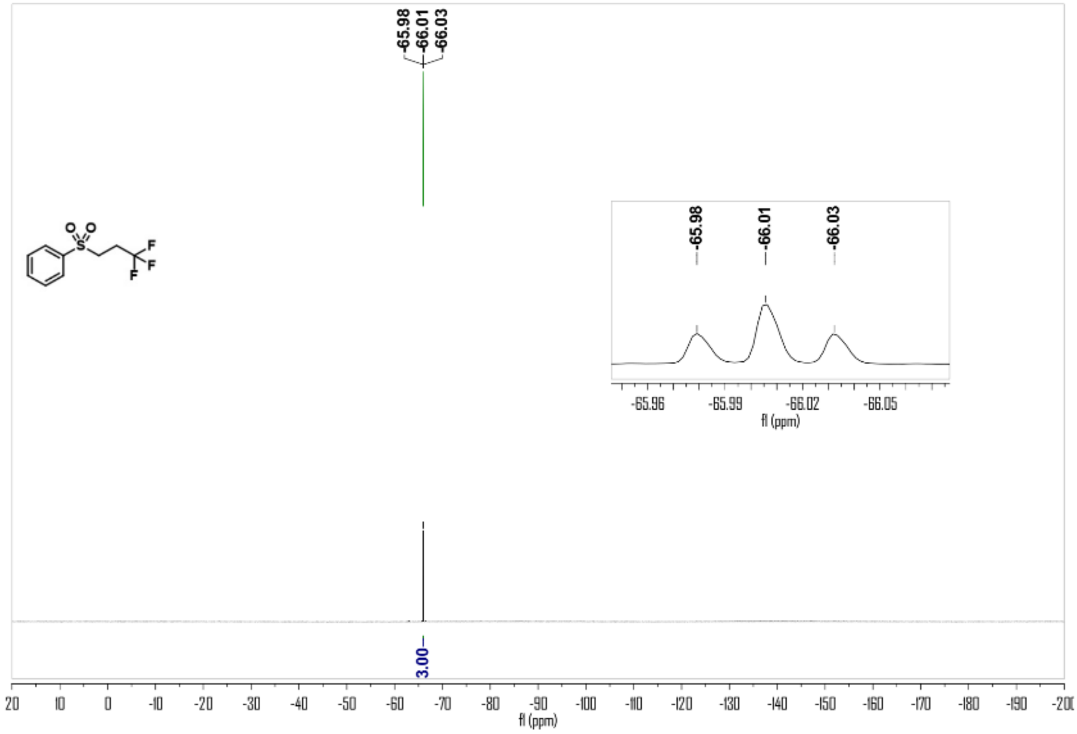

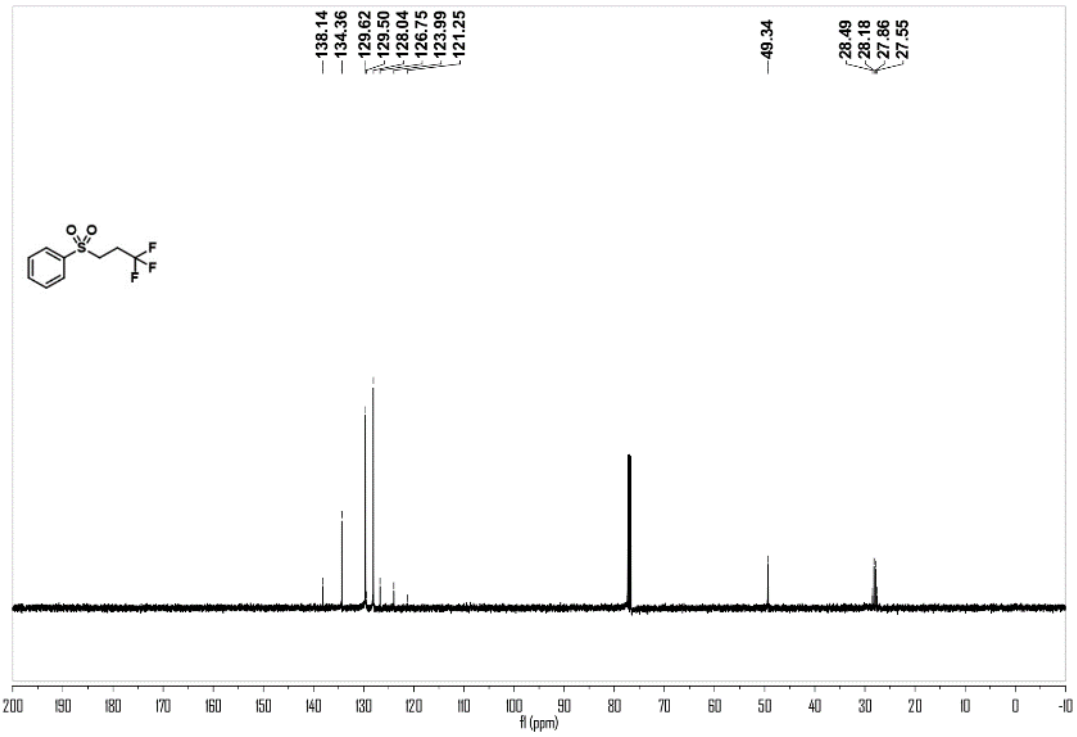

^{1}H, ^{19}F and ^{13}C NMR spectra of compound 3ah

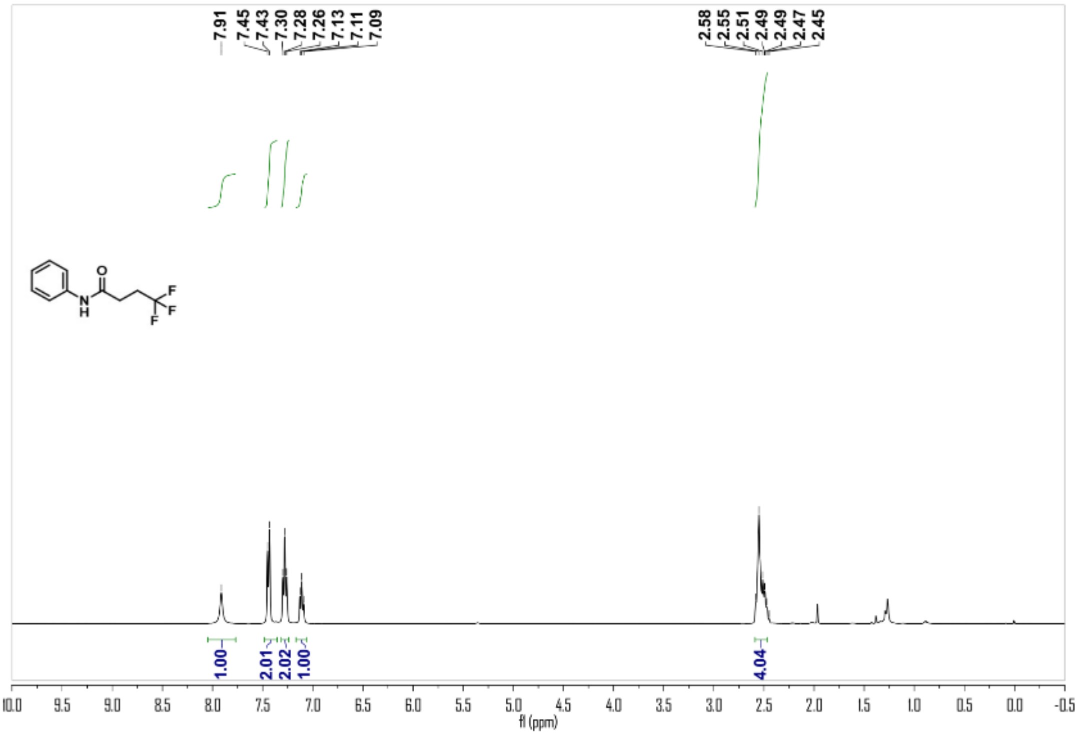

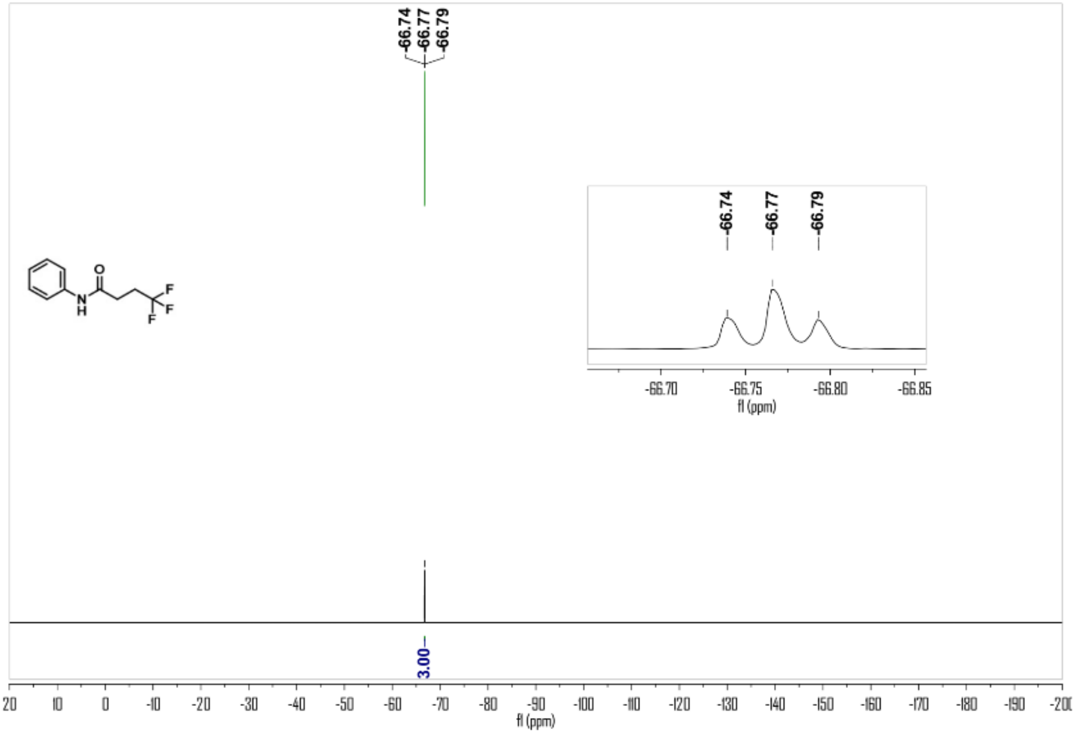
-66.74
-66.77
-66.79
3.00
20
10
0
-10
-20
-30
-40
-50
-60
-70
-80
-90
-100
-110
-120
-130
-140
-150
-160
-170
-180
-190
-200
f1 (ppm)
-66.70
-66.75
-66.80
-66.85

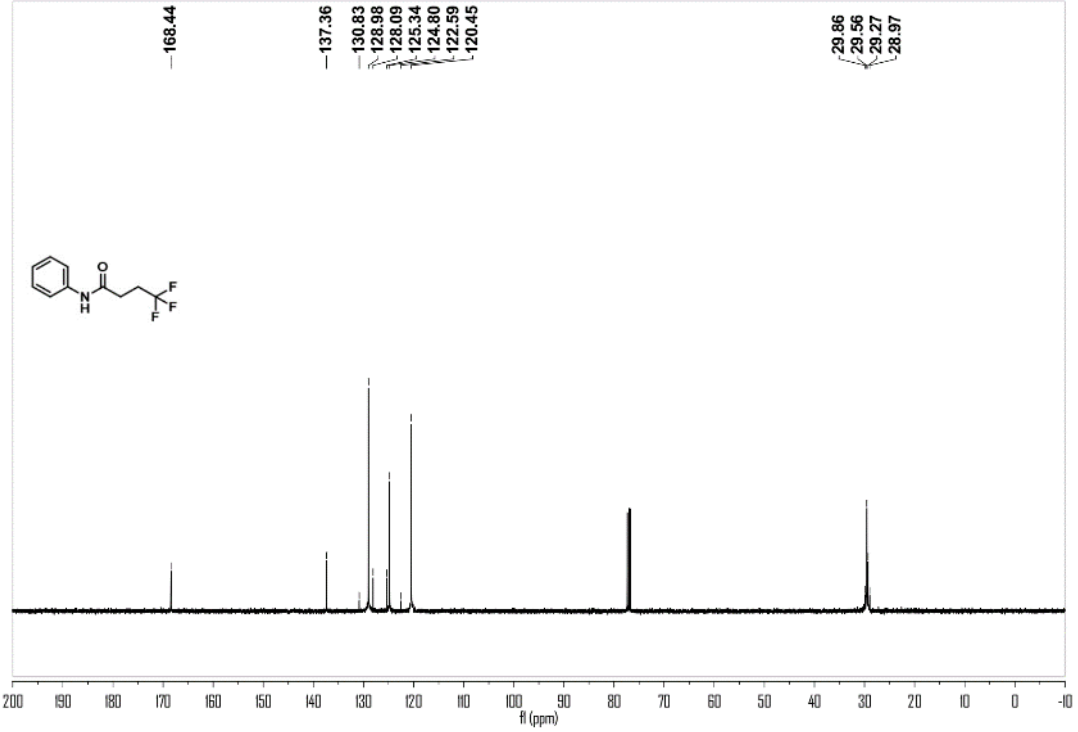
168.44
137.36
130.83
128.98
128.09
125.34
124.80
122.59
120.45
29.86
29.56
29.27
28.97
200
190
180
170
160
150
140
130
120
110
100
90
80
70
60
50
40
30
20
10
0
-10
f1 (ppm)

^{1}H, ^{19}F and ^{13}C NMR spectra of compound 3ai

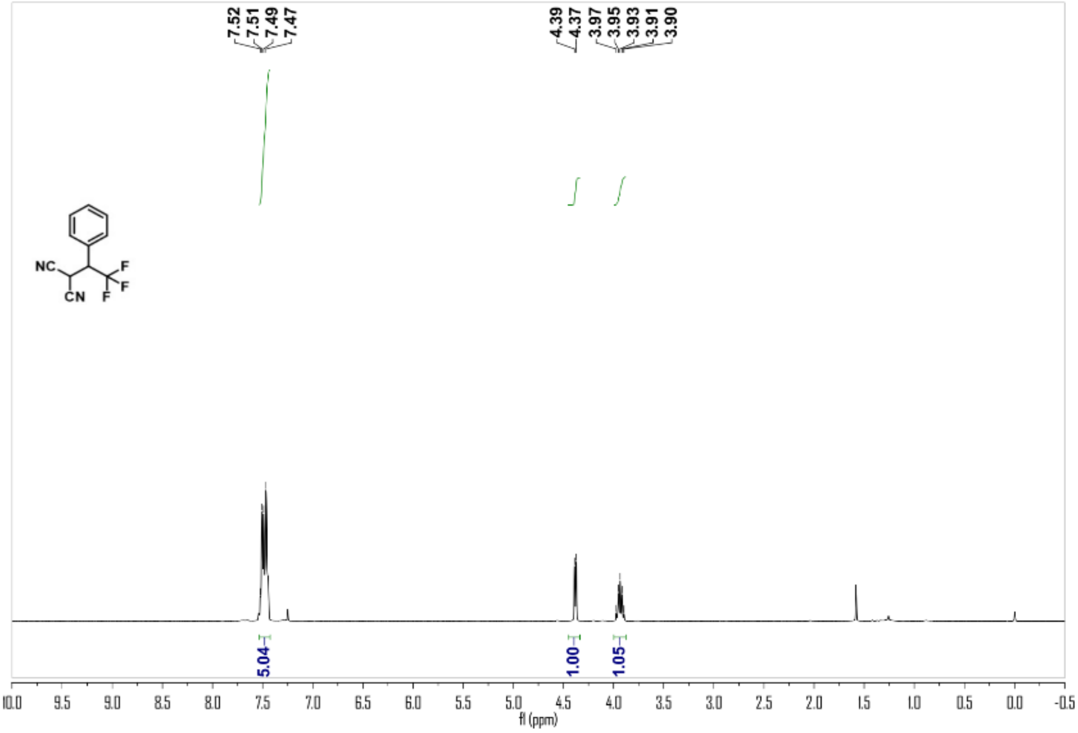

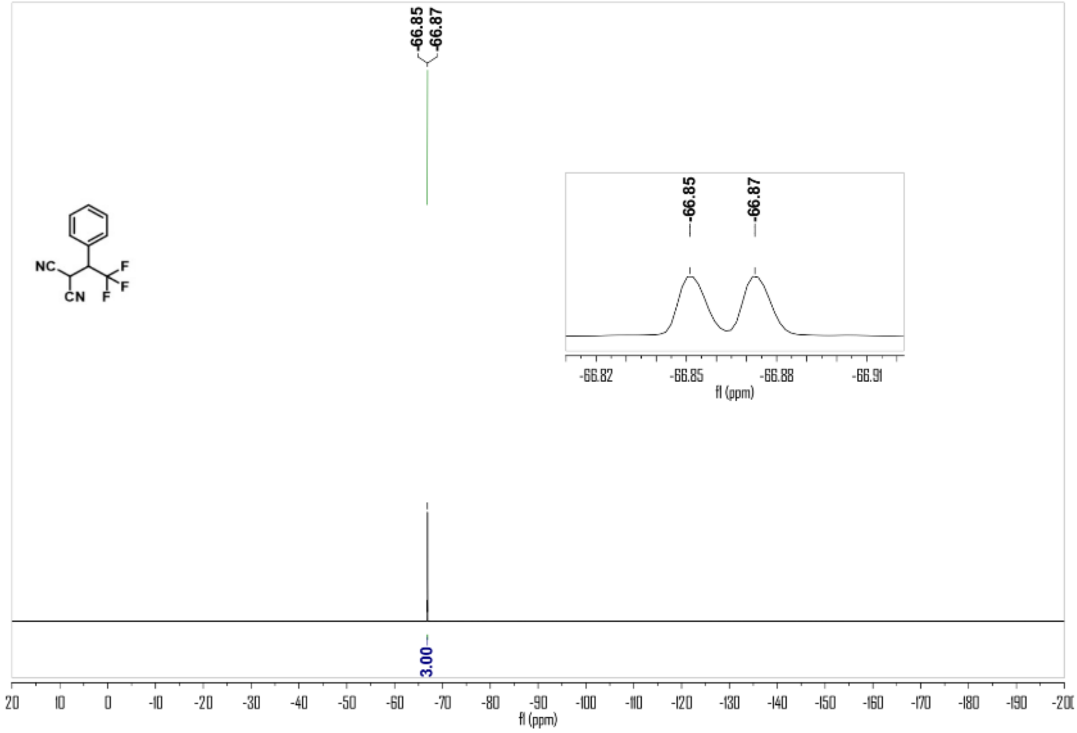

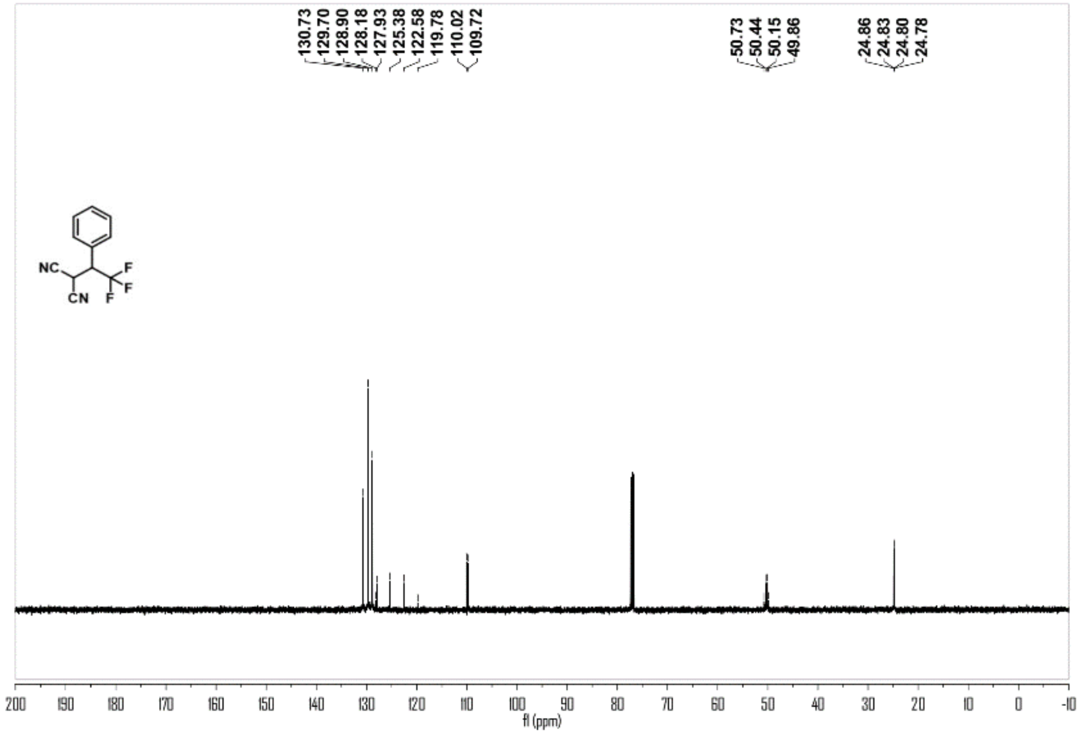

^{1}H, ^{19}F and ^{13}C NMR spectra of compound 3aj

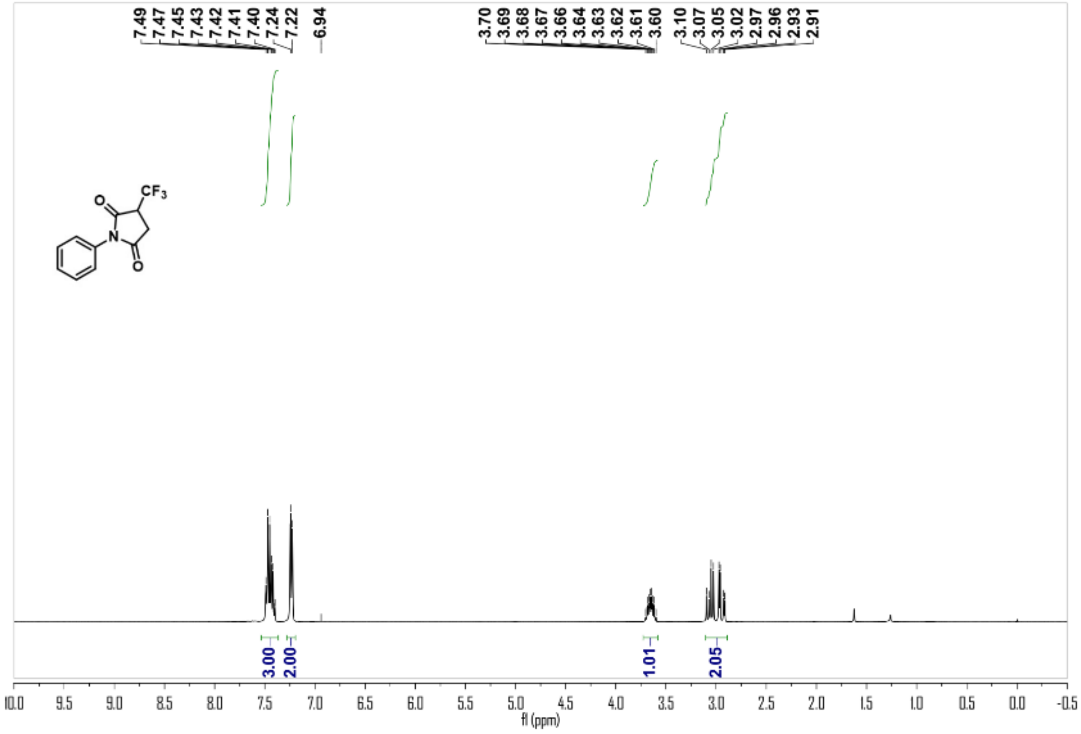

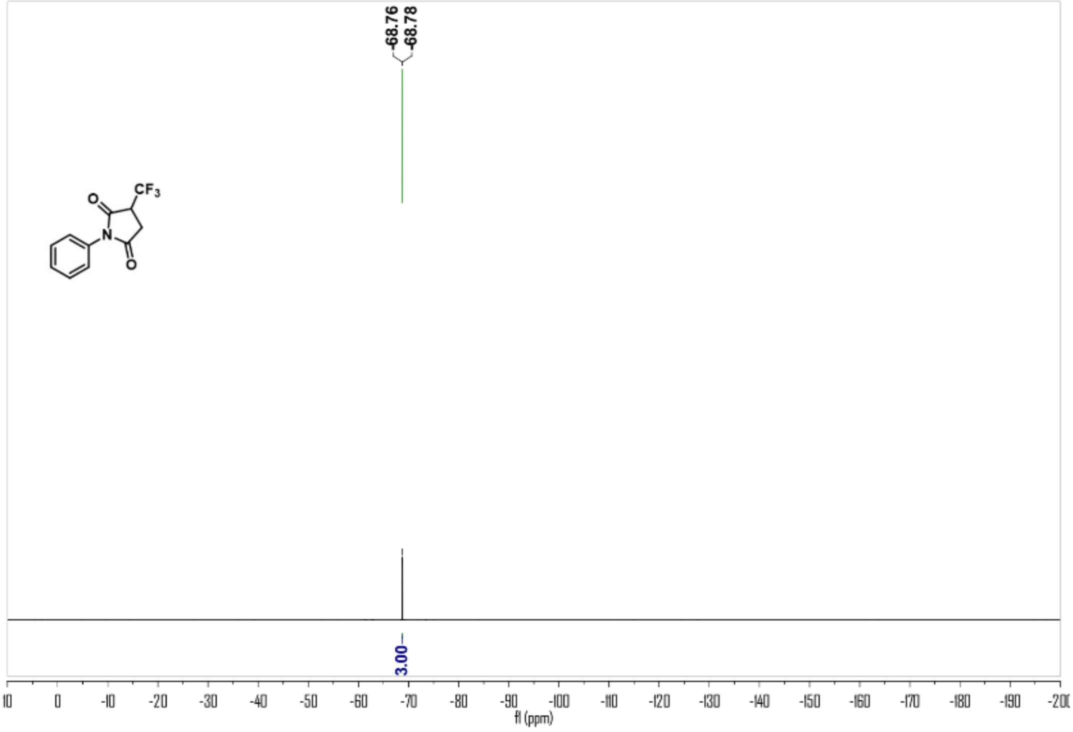
-68.76
-68.78
CF3
O
N
O
3.00
10
0
-10
-20
-30
-40
-50
-60
-70
-80
-90
-100
-110
-120
-130
-140
-150
-160
-170
-180
-190
-200
f1 (ppm)

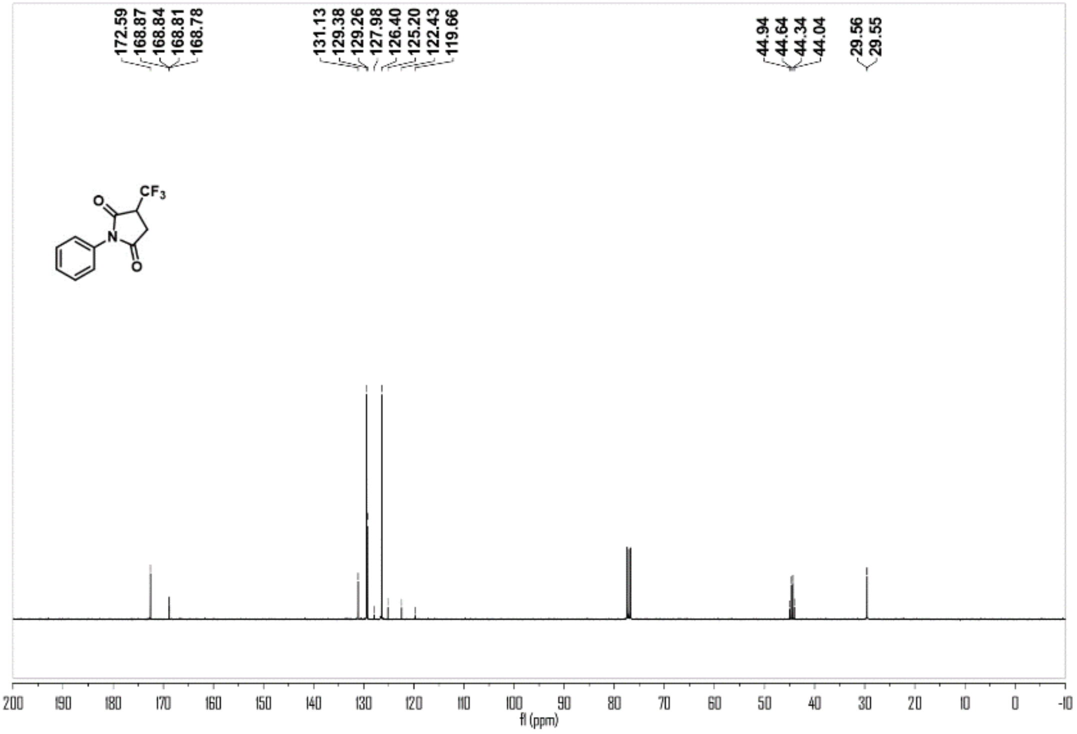
172.59
168.87
168.84
168.81
168.78
131.13
129.38
129.26
127.98
126.40
125.20
122.43
119.66
44.94
44.64
44.34
44.04
29.56
29.55
CF3
O
N
O
200
190
180
170
160
150
140
130
120
110
100
90
80
70
60
50
40
30
20
10
0
-10
f1 (ppm)

^{1}H, ^{19}F and ^{13}C NMR spectra of compound 3ak

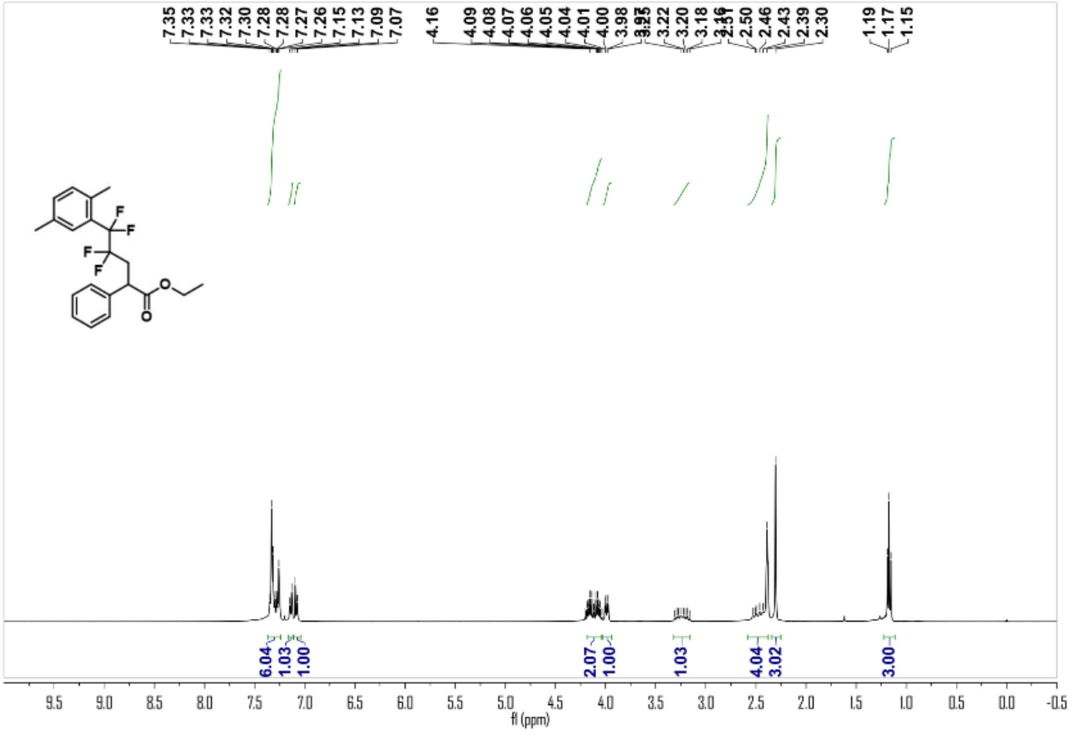

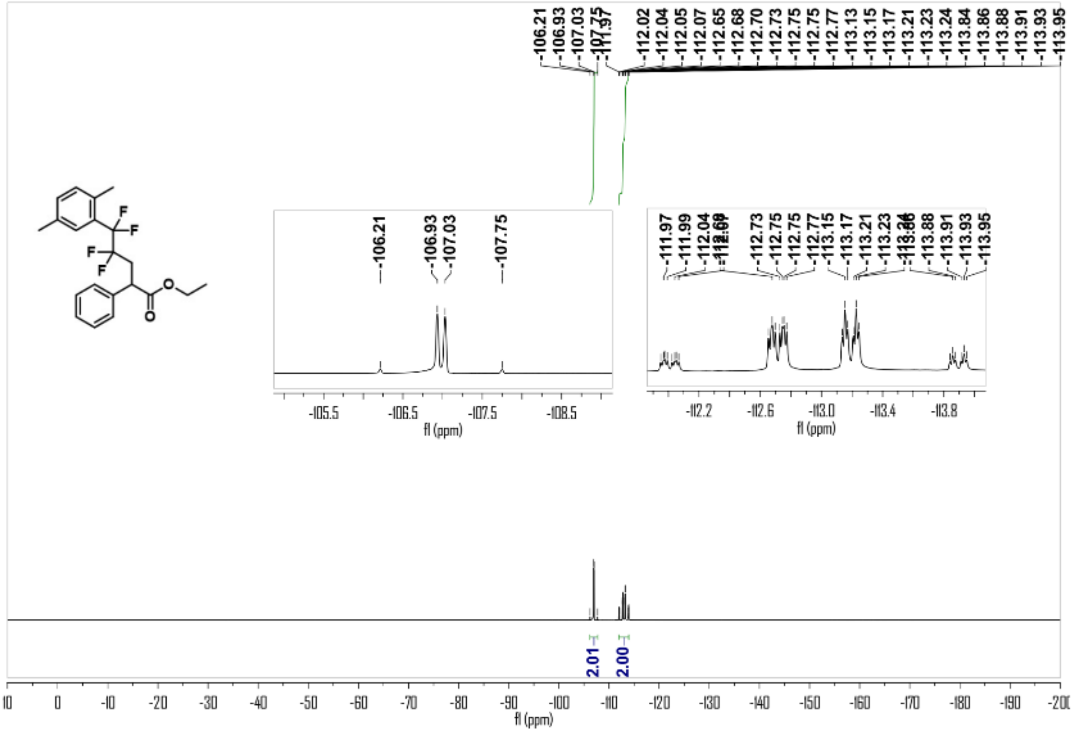

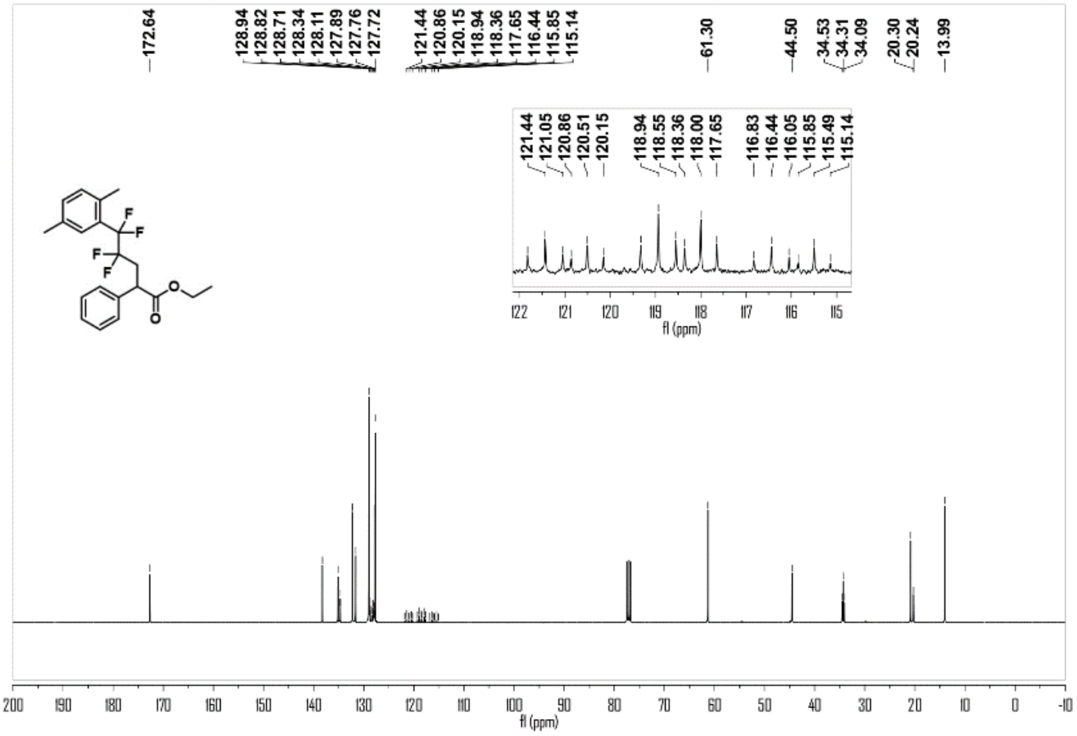

^{1}H, ^{19}F and ^{13}C NMR spectra of compound 3al

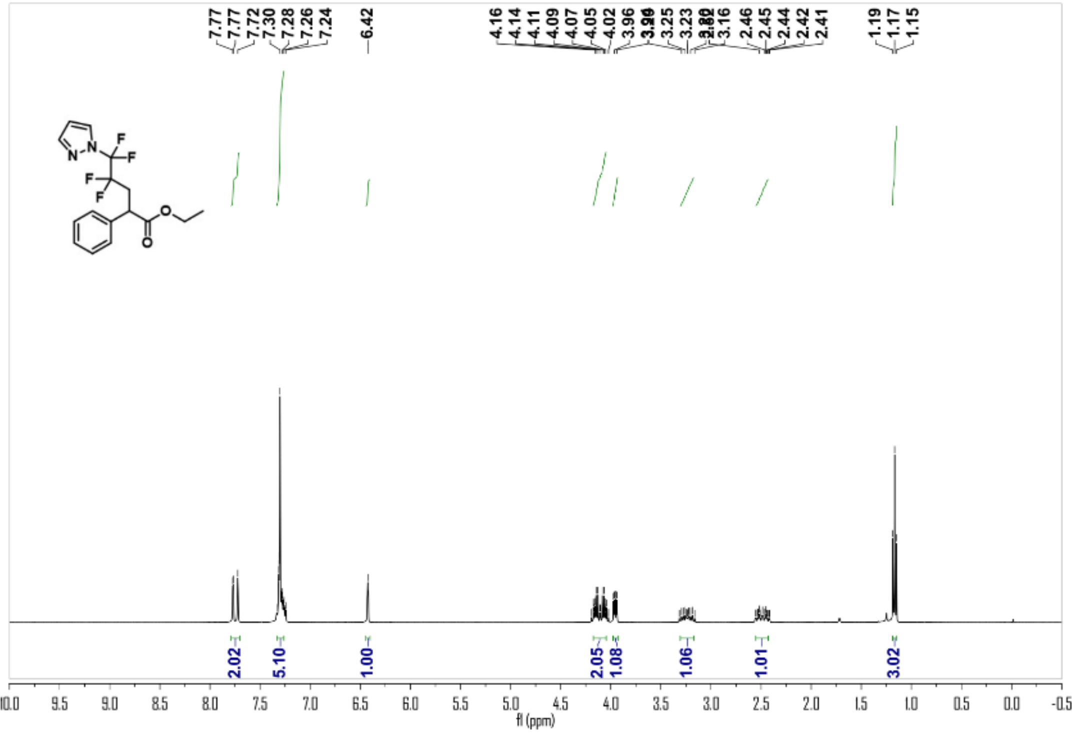

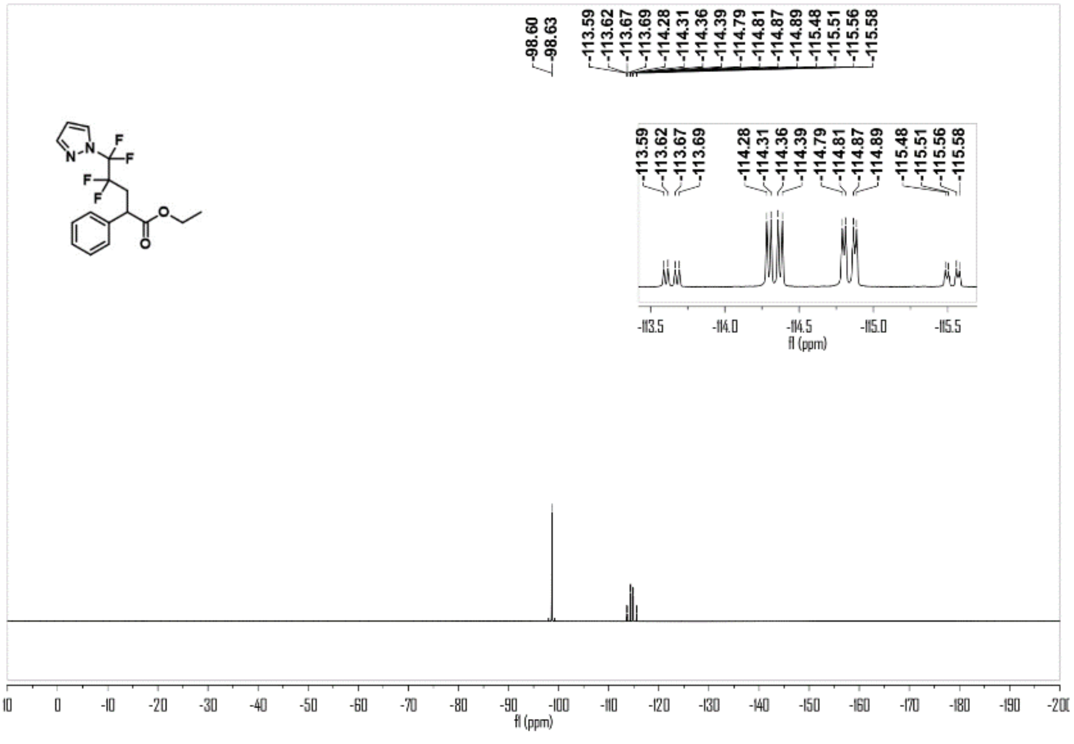

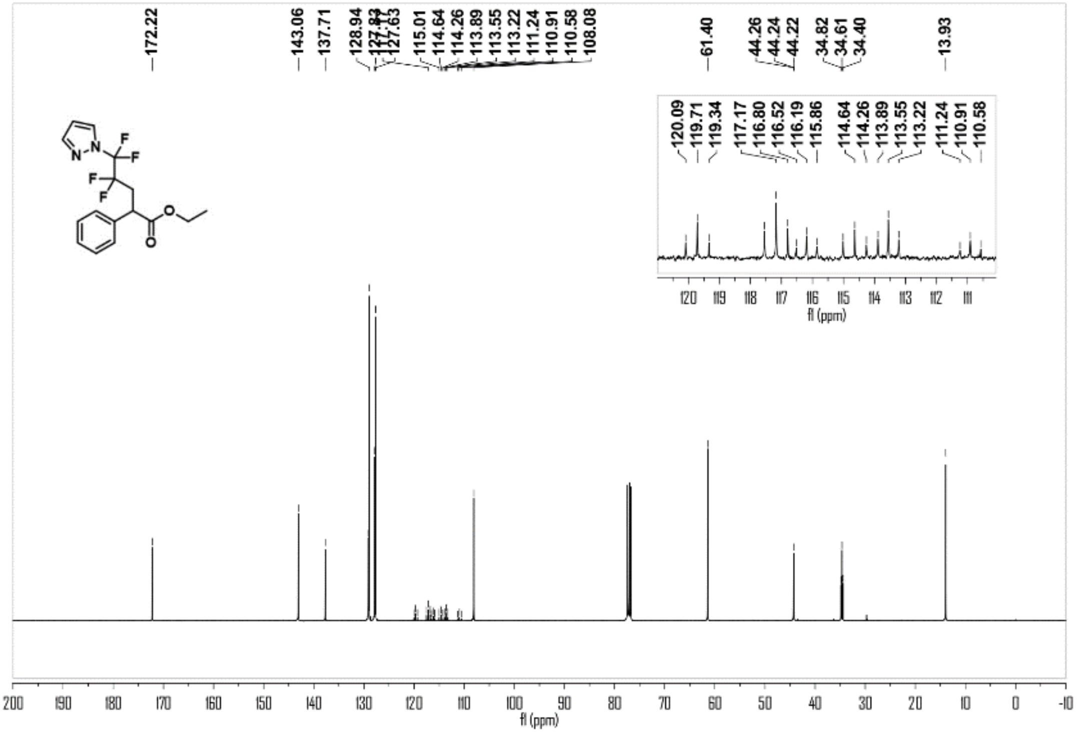

^{1}H, ^{19}F and ^{13}C NMR spectra of compound 3am

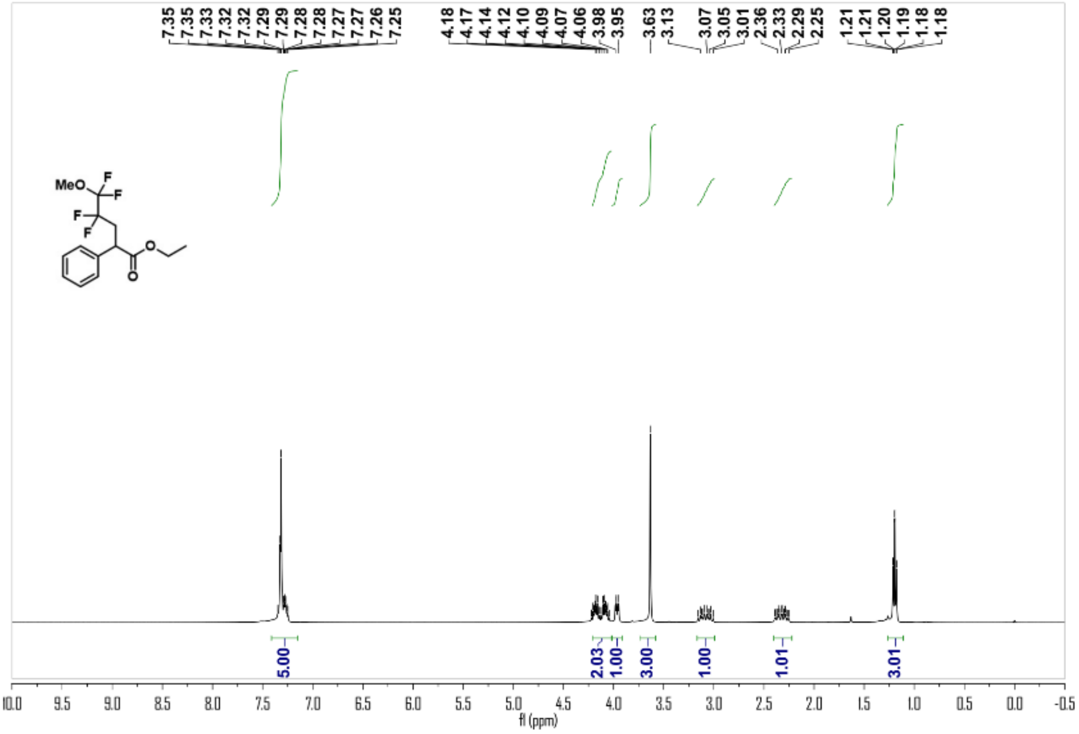

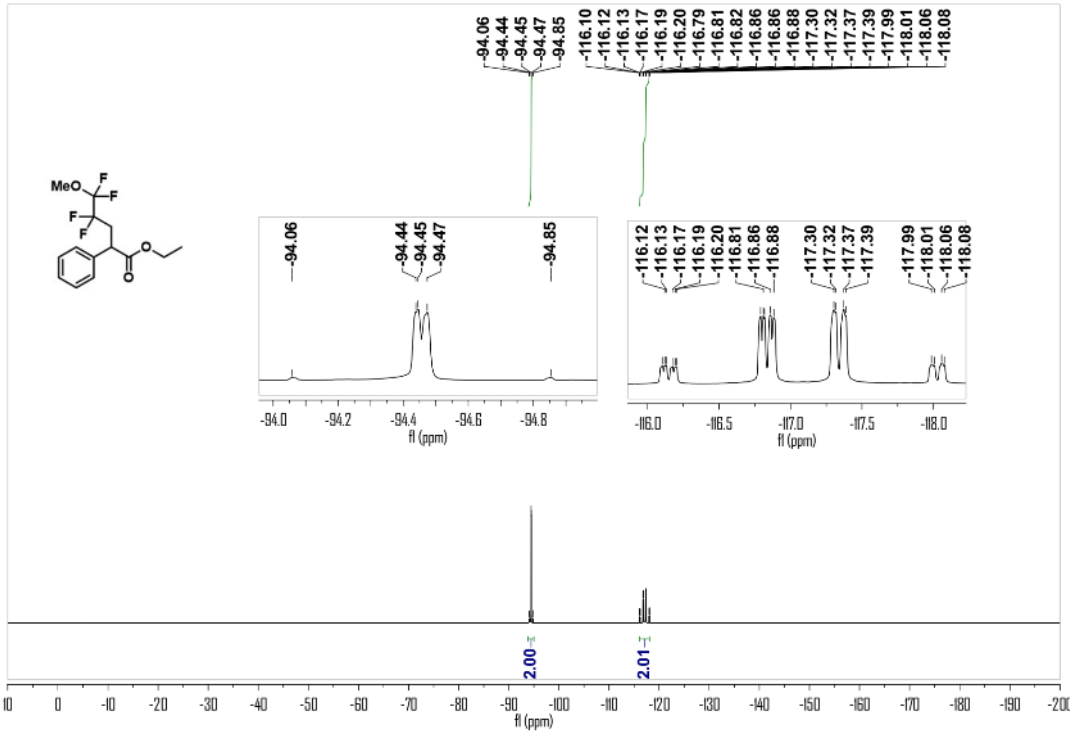

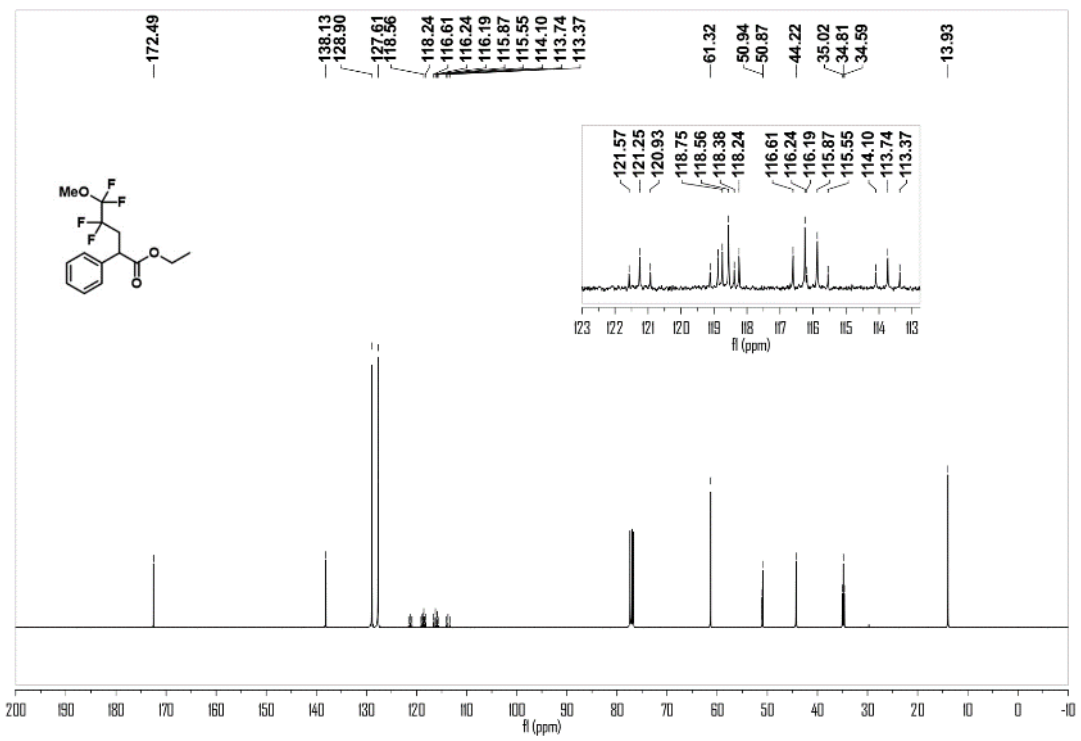

^{1}H, ^{19}F and ^{13}C NMR spectra of compound 3an

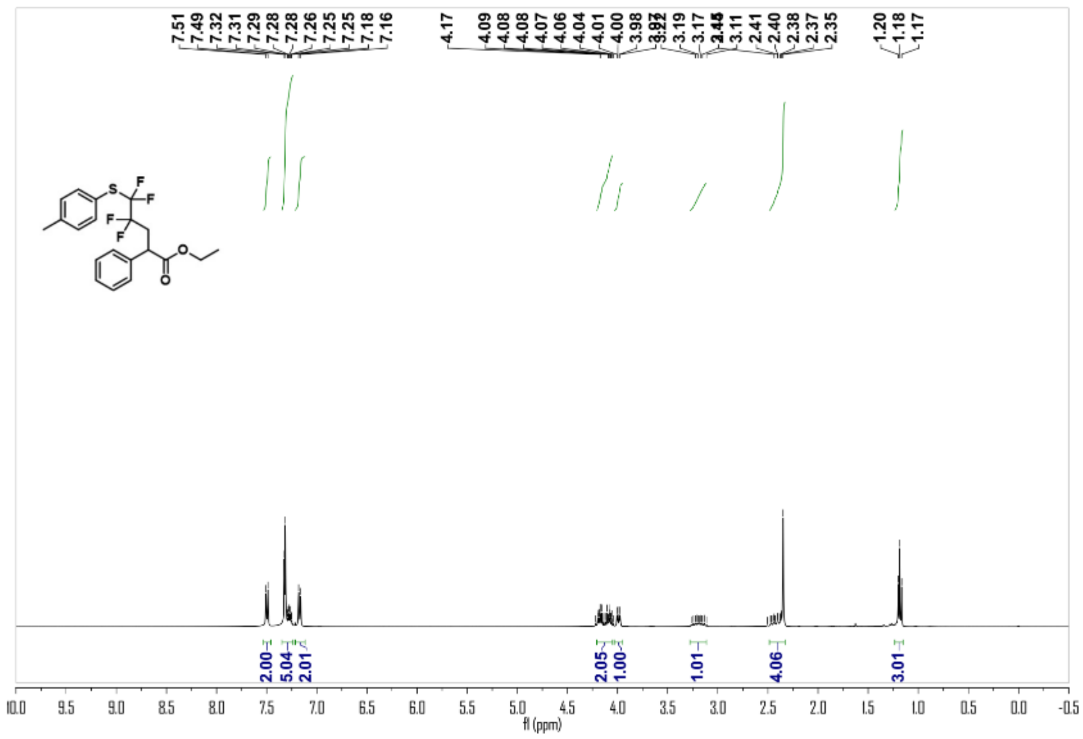

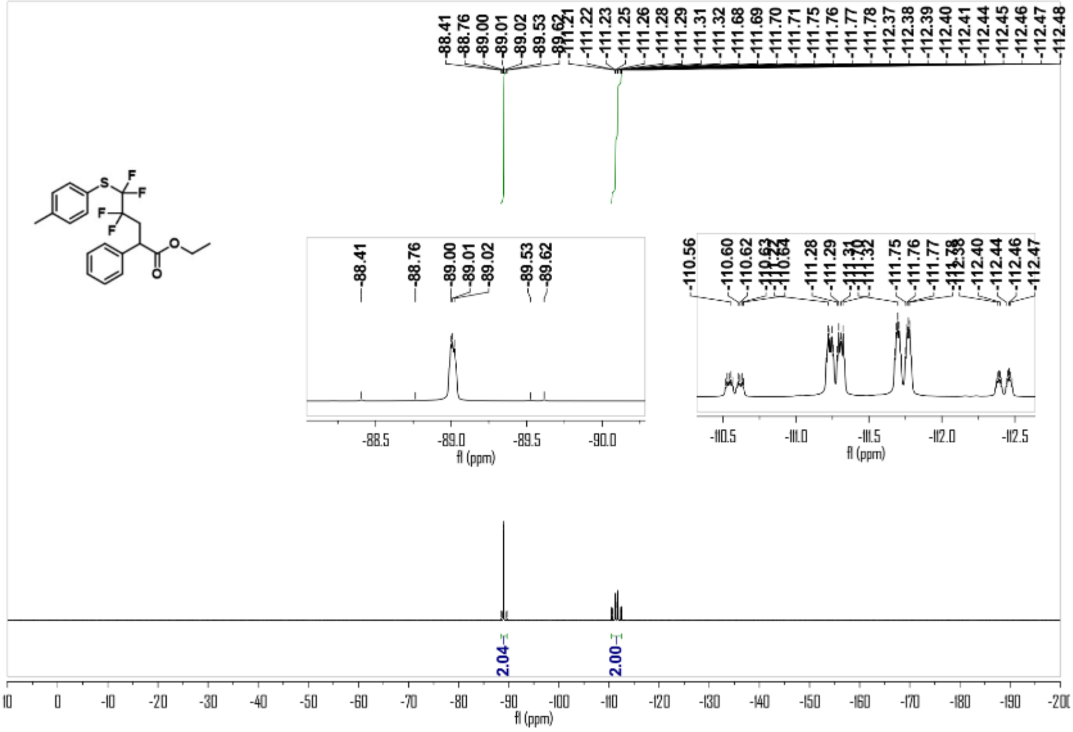

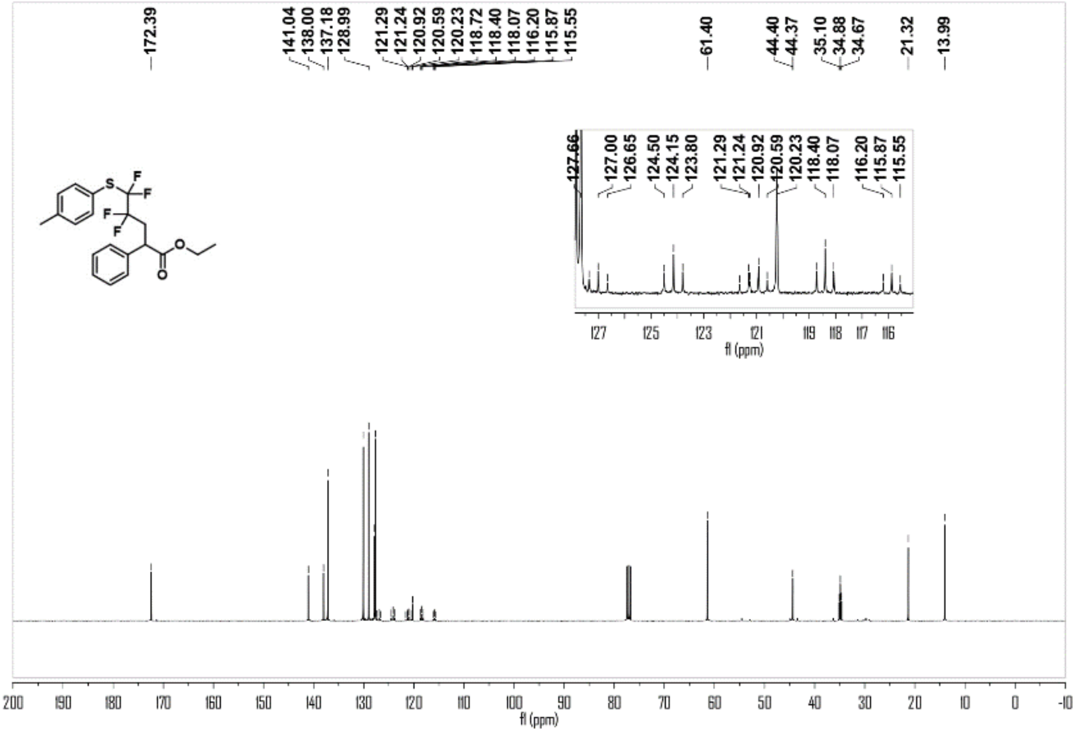

^{1}H, ^{19}F and ^{13}C NMR spectra of compound 3ao

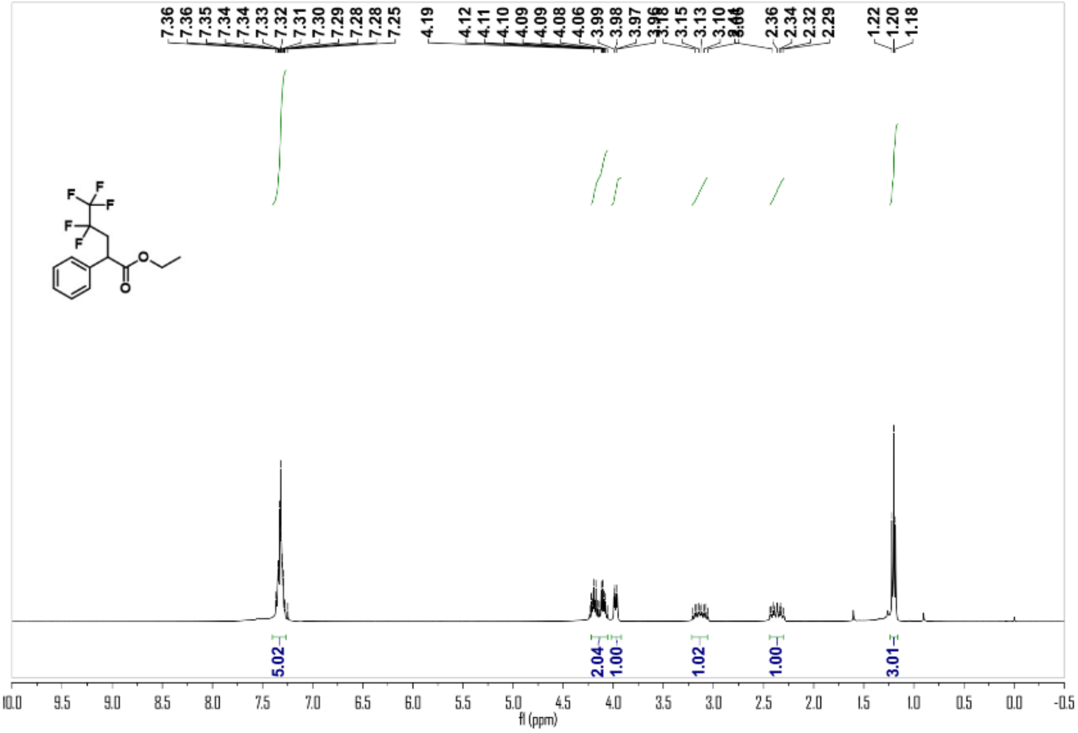

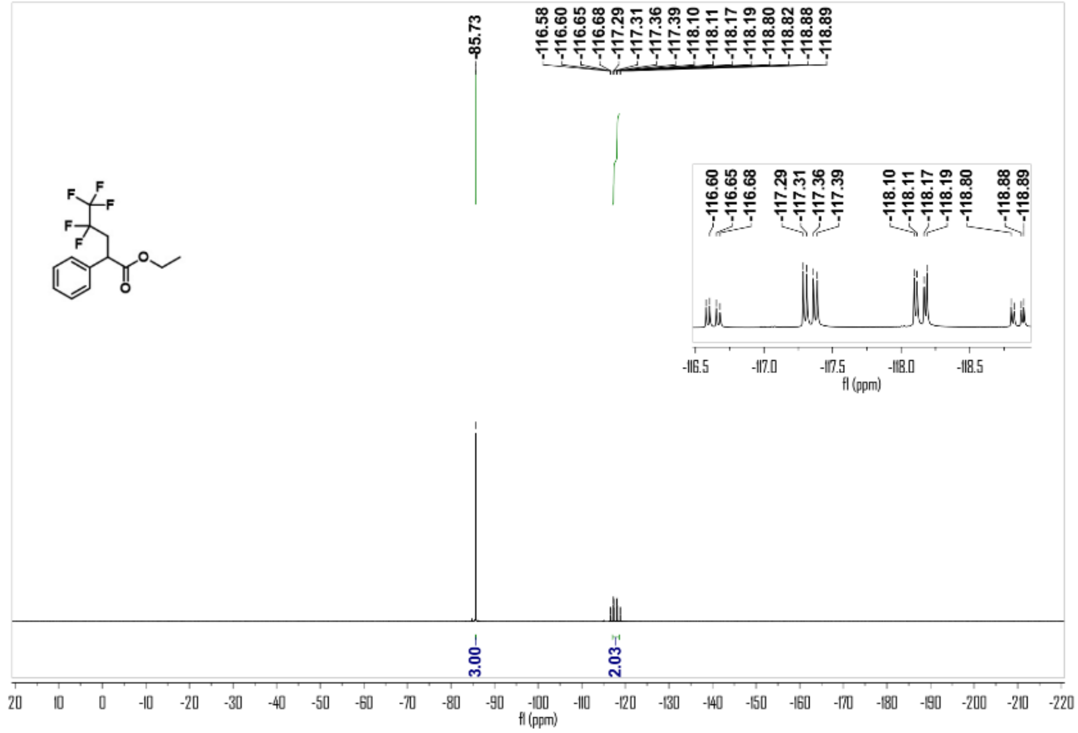

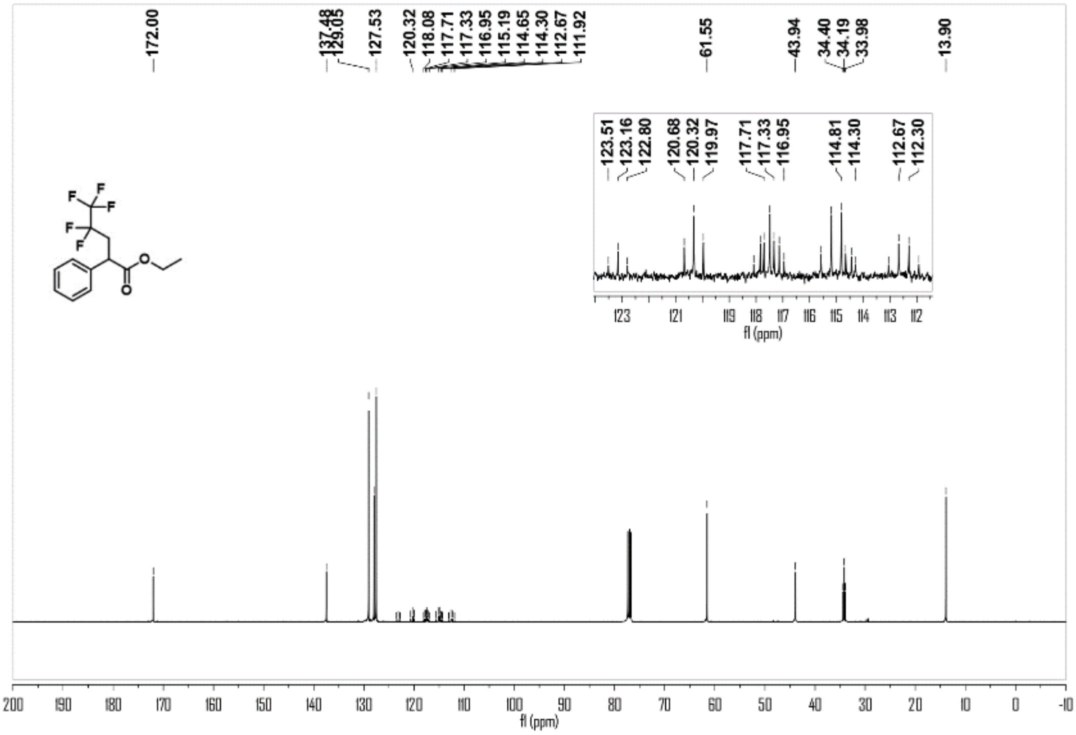

^{1}H, ^{19}F and ^{13}C NMR spectra of compound 3ap

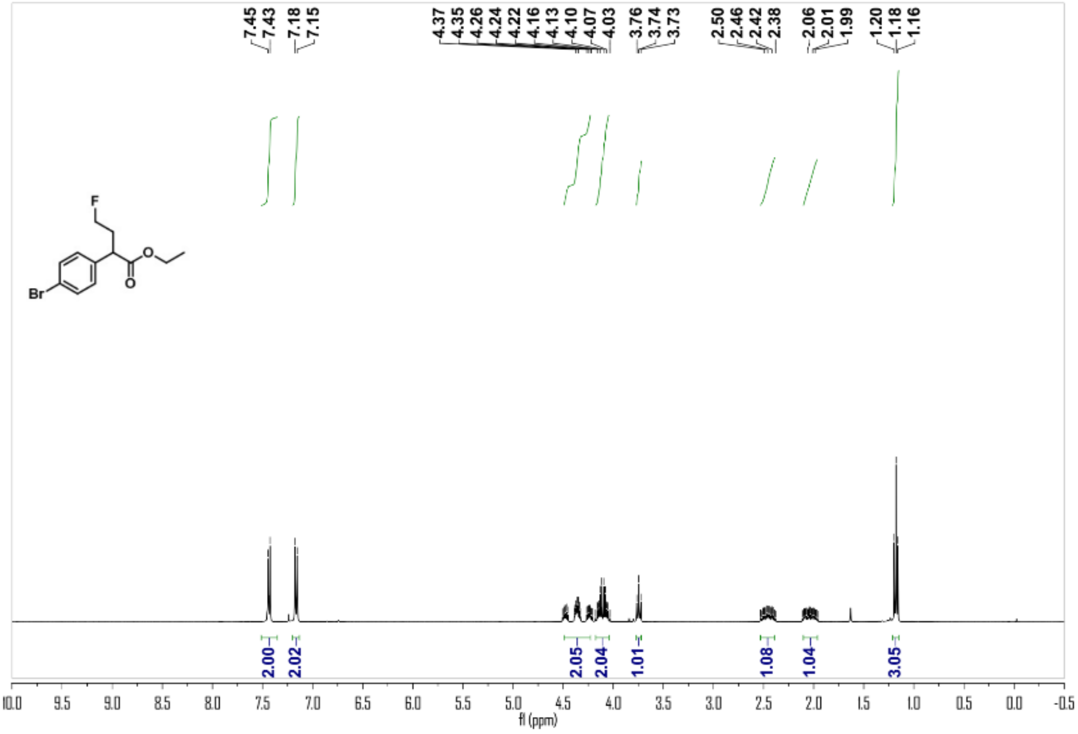

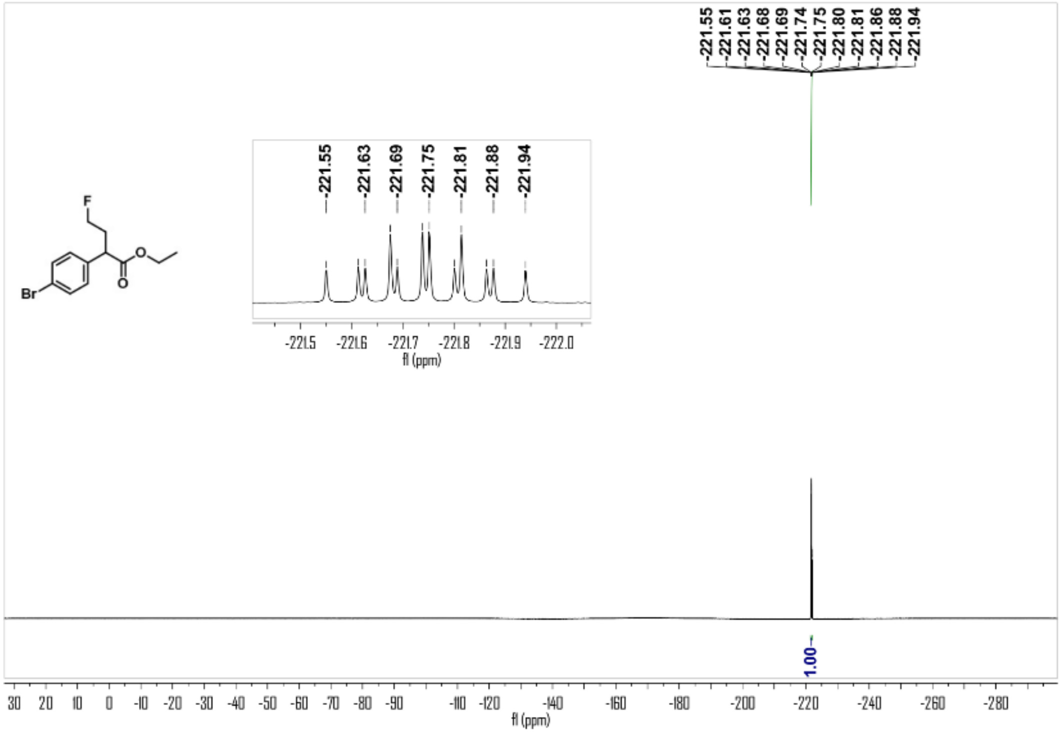
-221.55
-221.61
-221.63
-221.68
-221.69
-221.74
-221.75
-221.80
-221.81
-221.86
-221.88
-221.94
F
Br
O
O
-221.55
-221.63
-221.69
-221.75
-221.81
-221.88
-221.94
-221.5 -221.6 -221.7 -221.8 -221.9 -222.0
fl (ppm)
1.00
30 20 10 0 -10 -20 -30 -40 -50 -60 -70 -80 -90 -110 -120 -140 -160 -180 -200 -220 -240 -260 -280
fl (ppm)

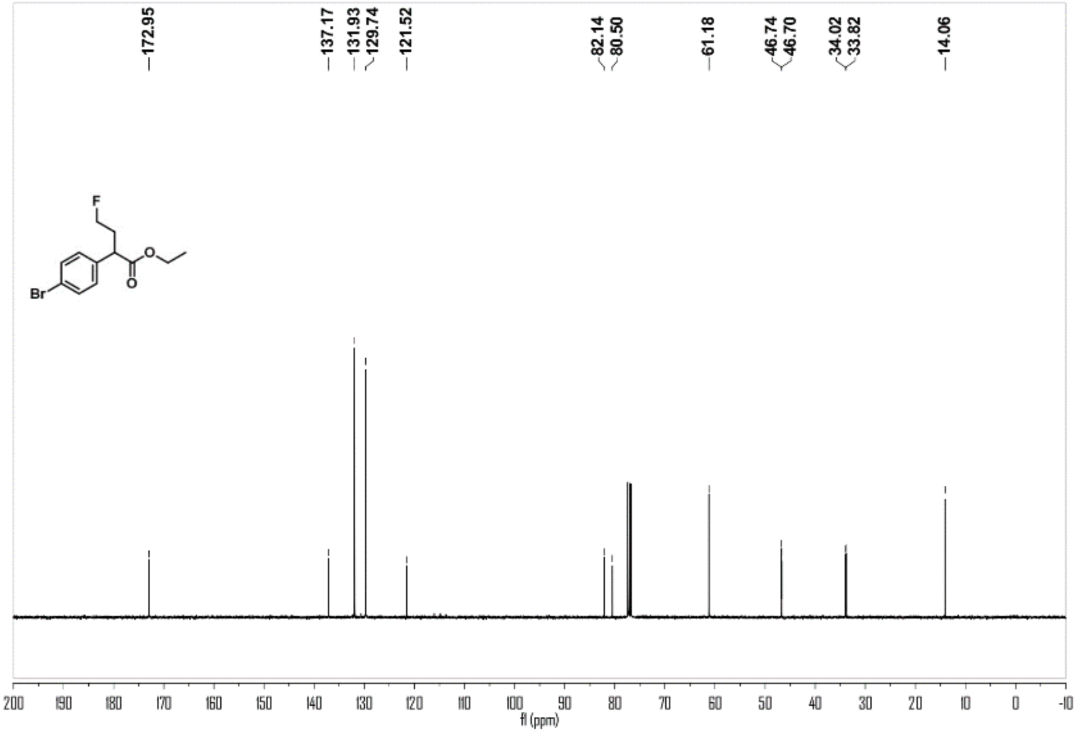
172.95
137.17
131.93
129.74
121.52
82.14
80.50
61.18
46.74
46.70
34.02
33.82
14.06
F
Br
O
O
200 190 180 170 160 150 140 130 120 110 100 90 80 70 60 50 40 30 20 10 0 -10
fl (ppm)

^{1}H, ^{19}F and ^{13}C NMR spectra of compound 3aq

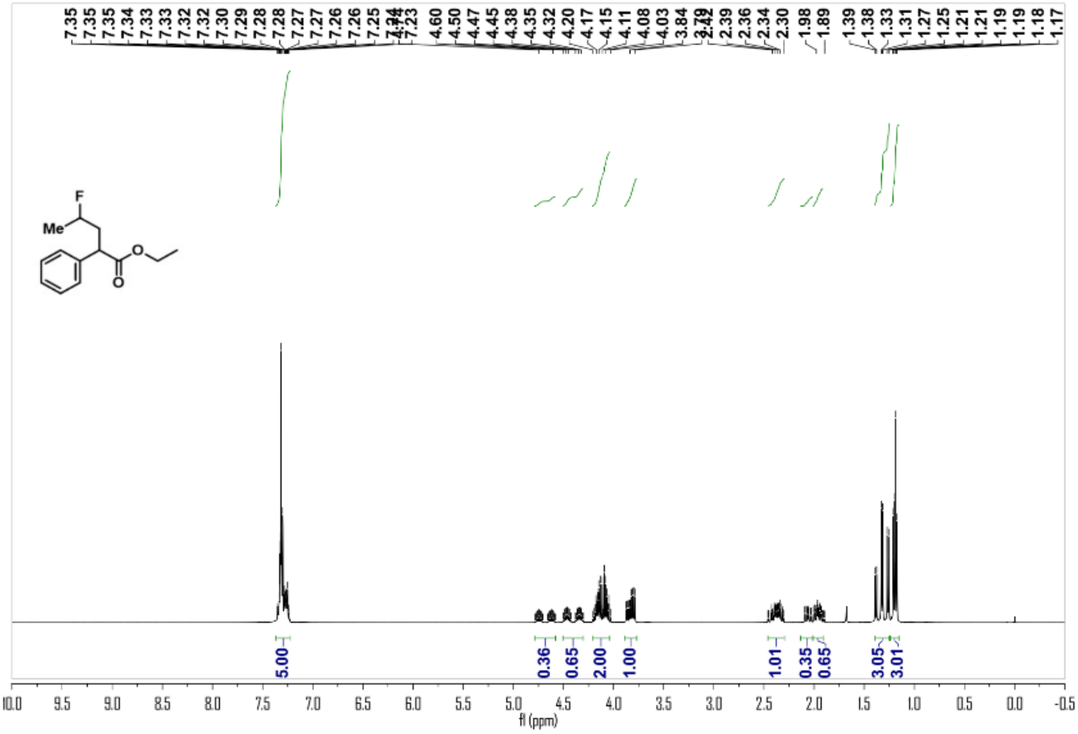

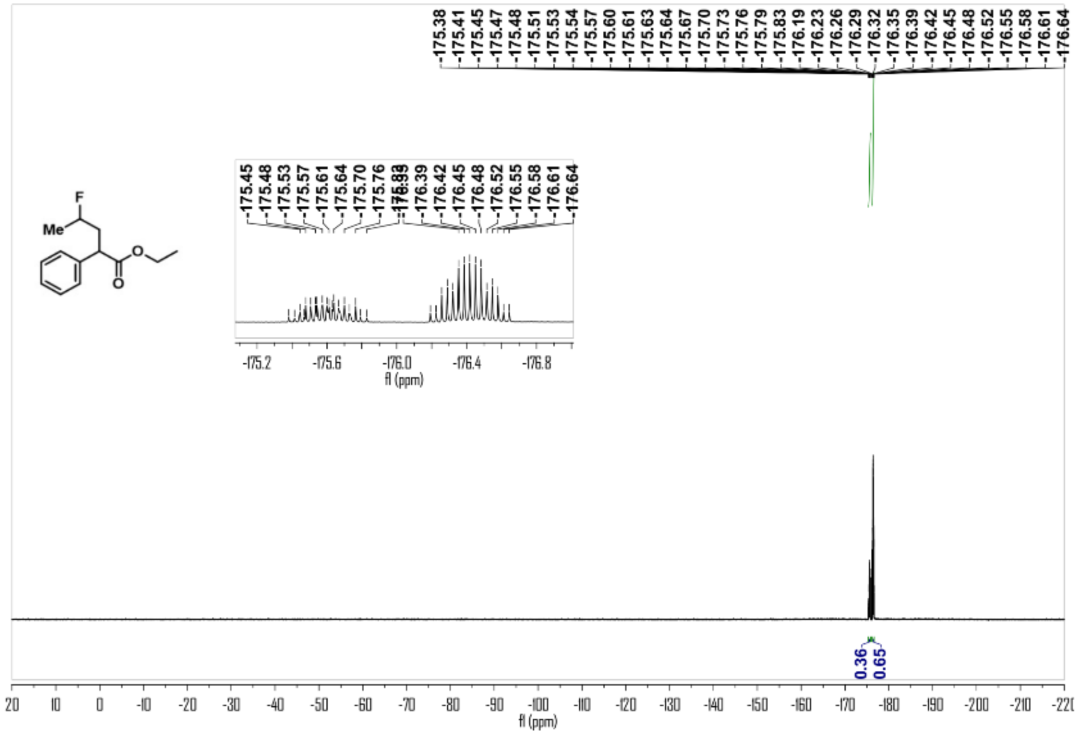

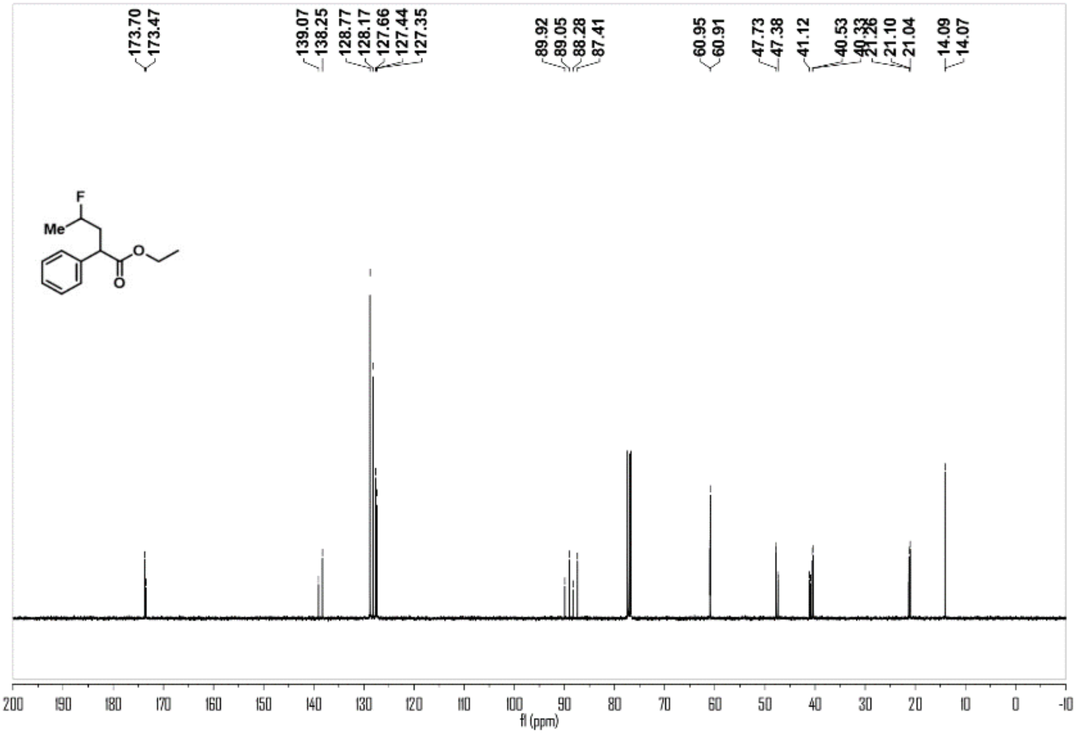

^{1}H, ^{19}F and ^{13}C NMR spectra of compound 3ar

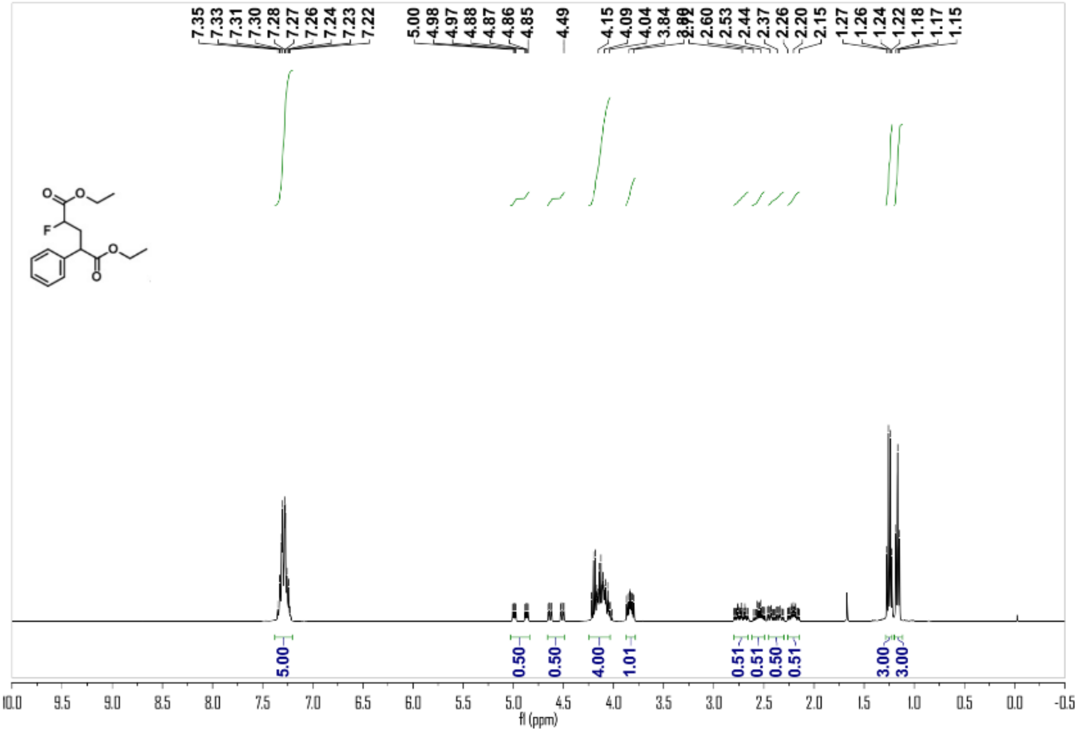

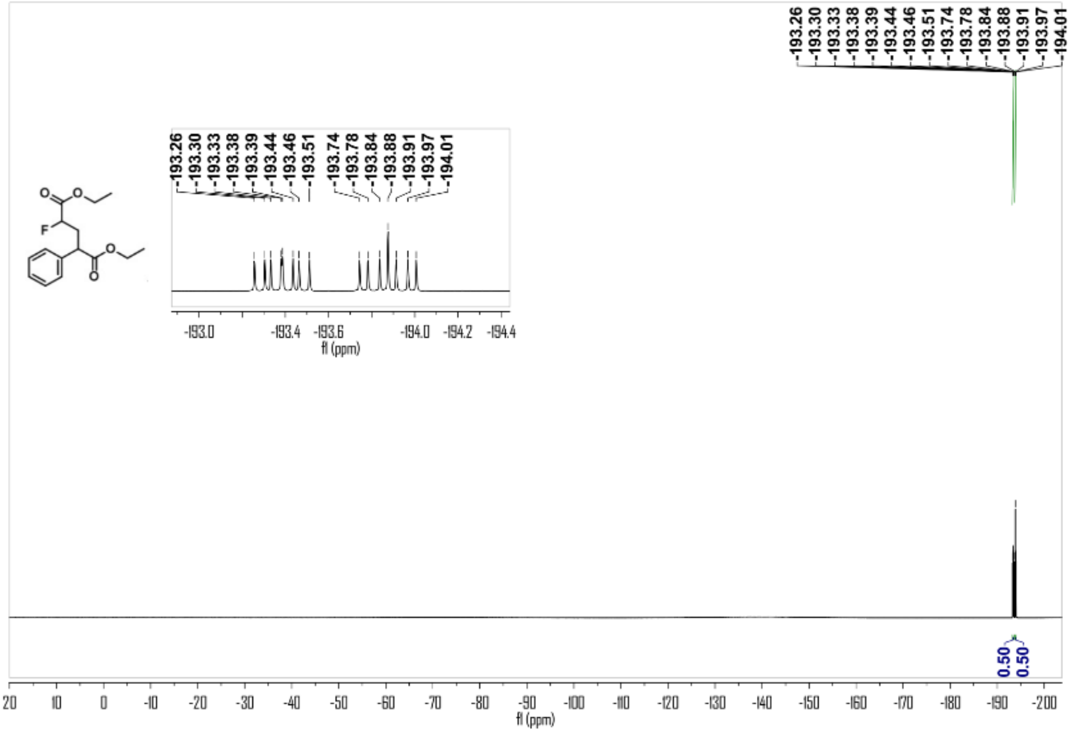
-193.26
-193.30
-193.33
-193.38
-193.39
-193.44
-193.46
-193.51
-193.74
-193.78
-193.84
-193.88
-193.91
-193.97
-194.01
F
0.50
0.50
f1 (ppm)

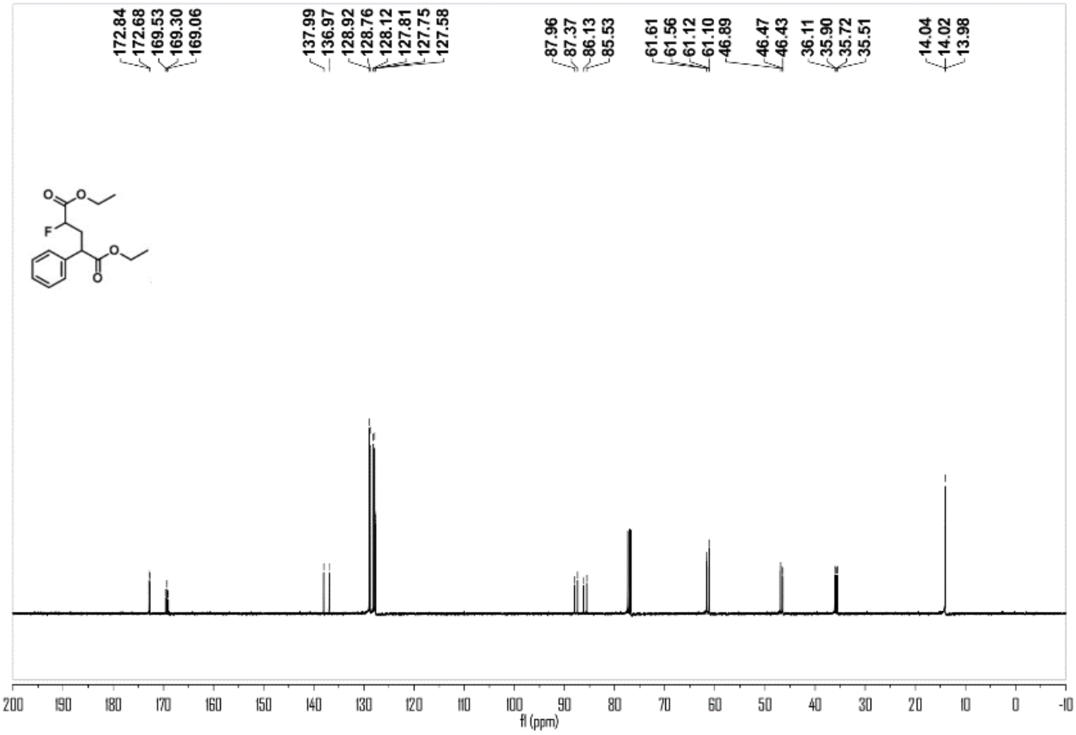
172.84
172.68
169.53
169.30
169.06
137.99
136.97
128.92
128.76
128.12
127.81
127.75
127.58
87.96
87.37
86.13
85.53
61.61
61.56
61.12
61.10
46.89
46.47
46.43
36.11
35.90
35.72
35.51
14.04
14.02
13.98
F
f1 (ppm)

^{1}H, ^{19}F and ^{13}C NMR spectra of compound 3as

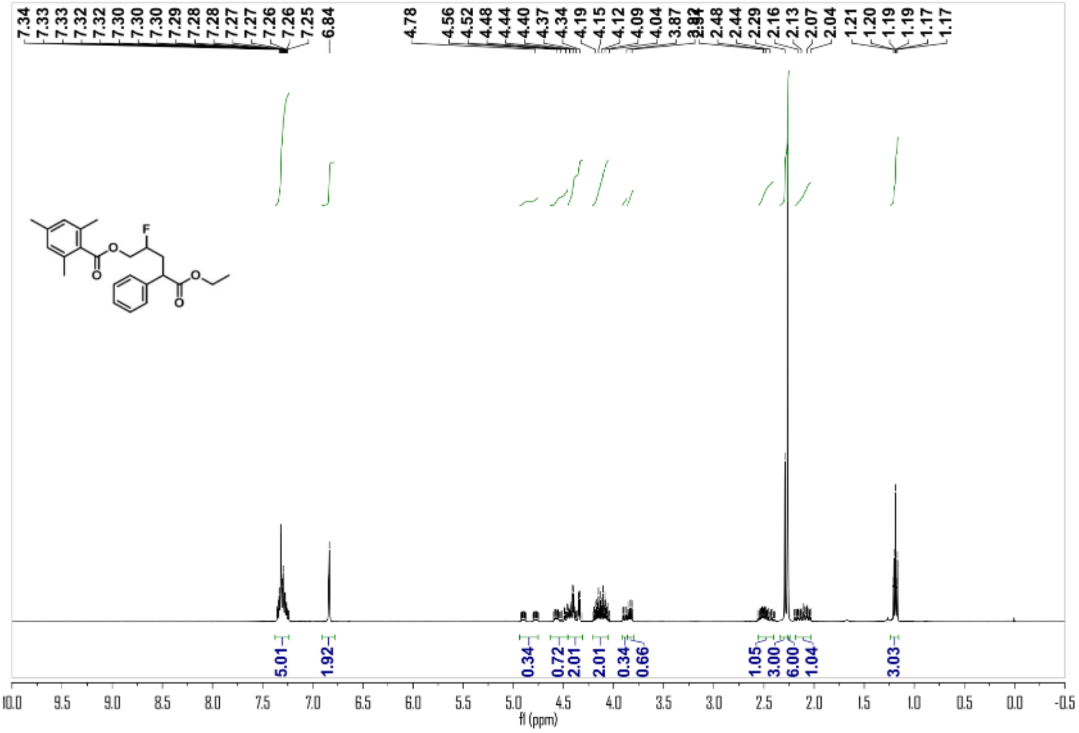

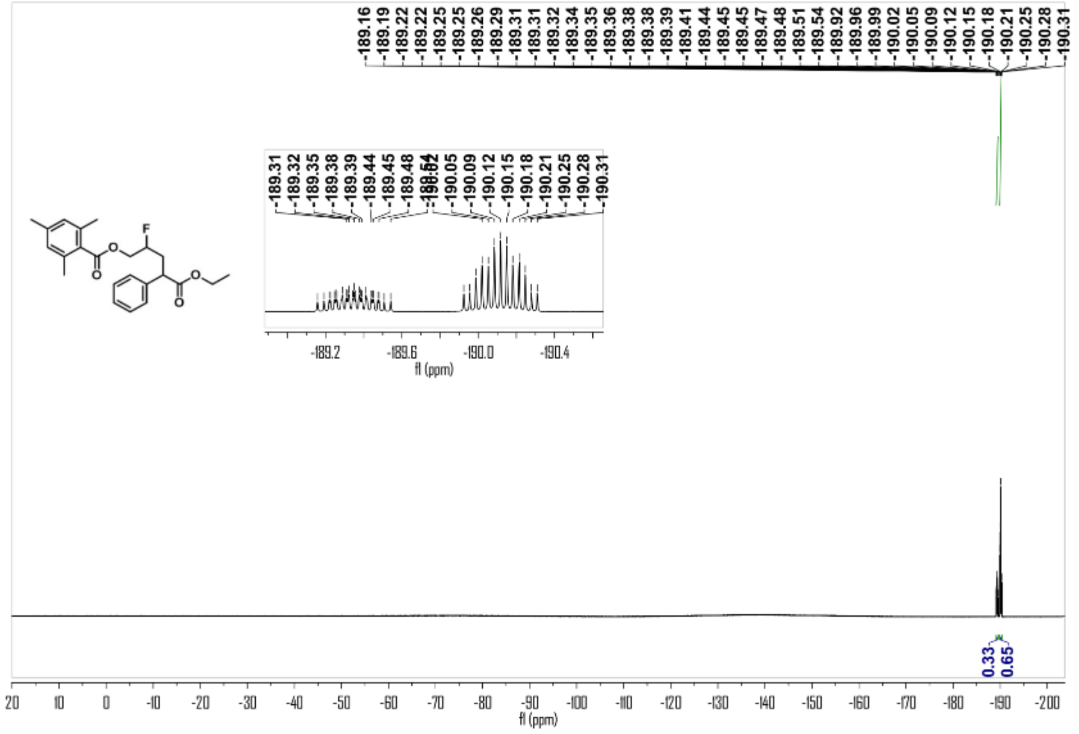

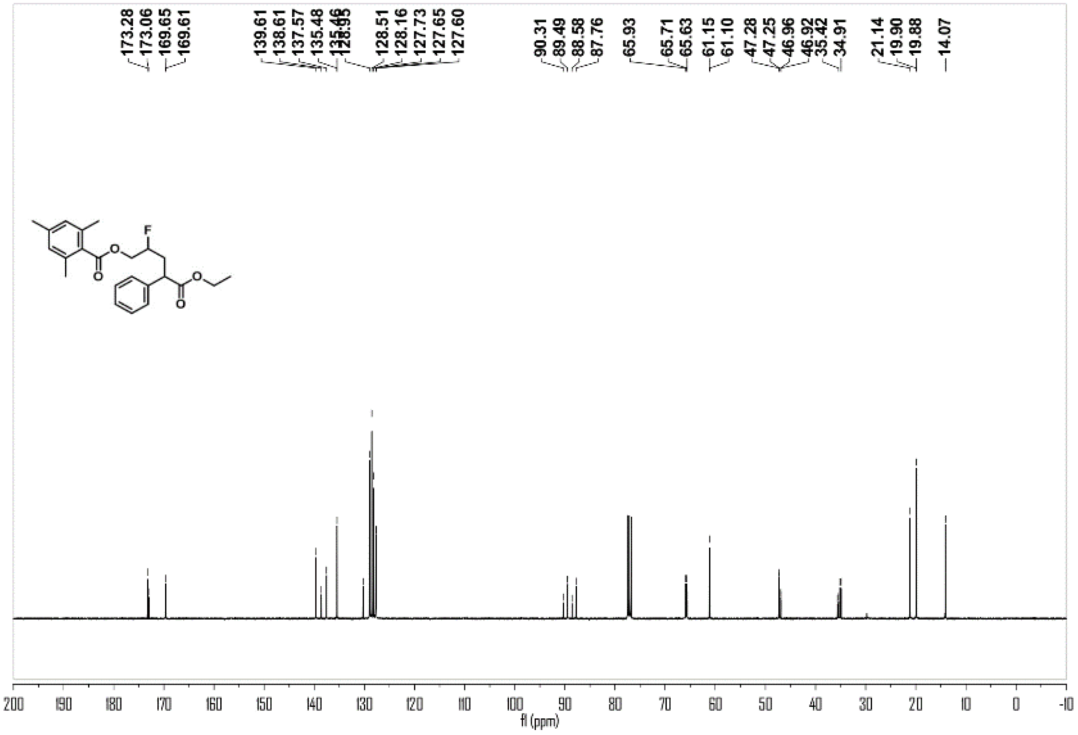

^{1}H, ^{19}F and ^{13}C NMR spectra of compound 3at

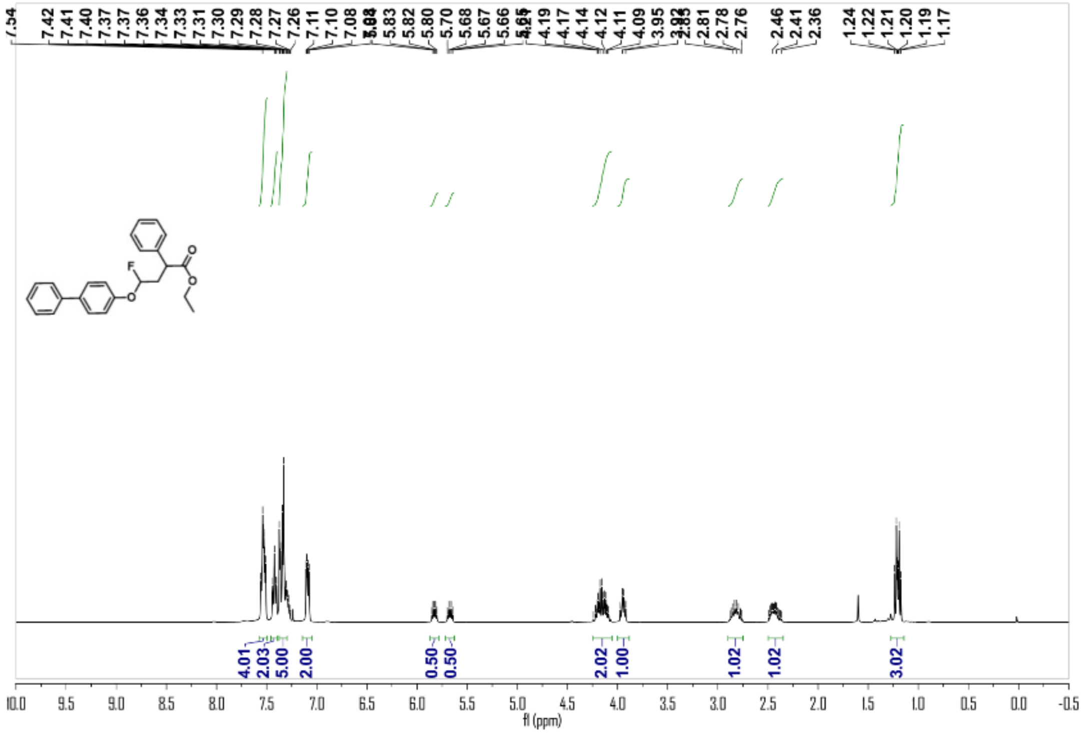

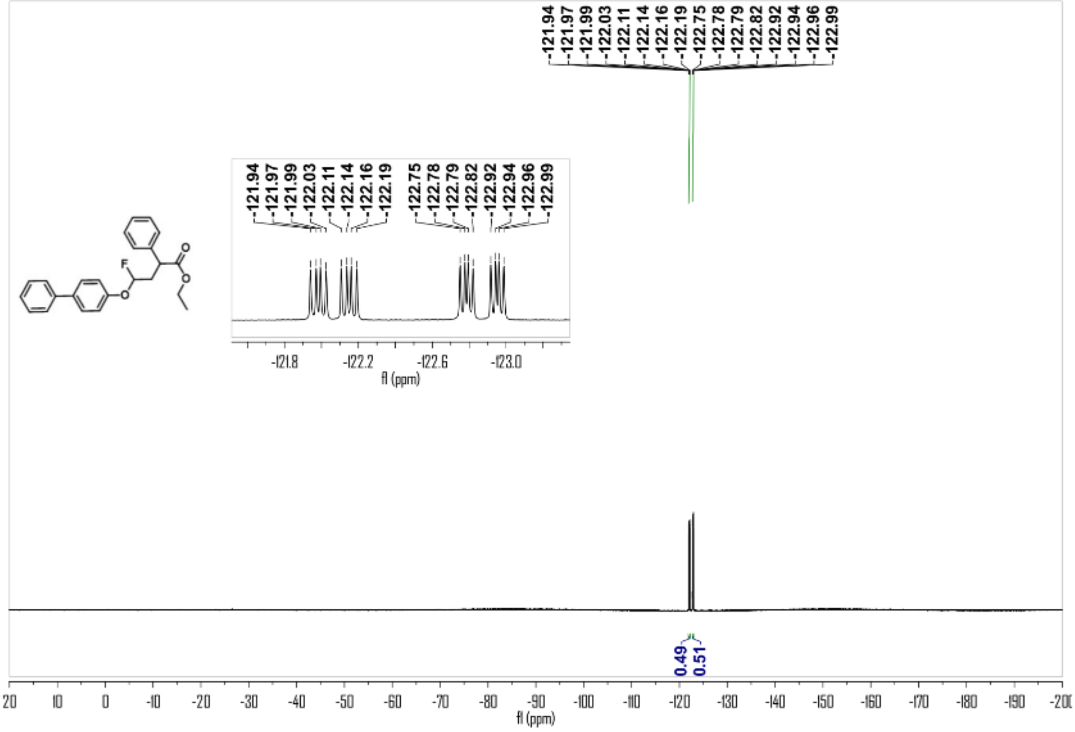
-121.94
-121.97
-121.99
-122.03
-122.11
-122.14
-122.16
-122.19
-122.75
-122.78
-122.79
-122.82
-122.92
-122.94
-122.96
-122.99
0.49
0.51
f1 (ppm)

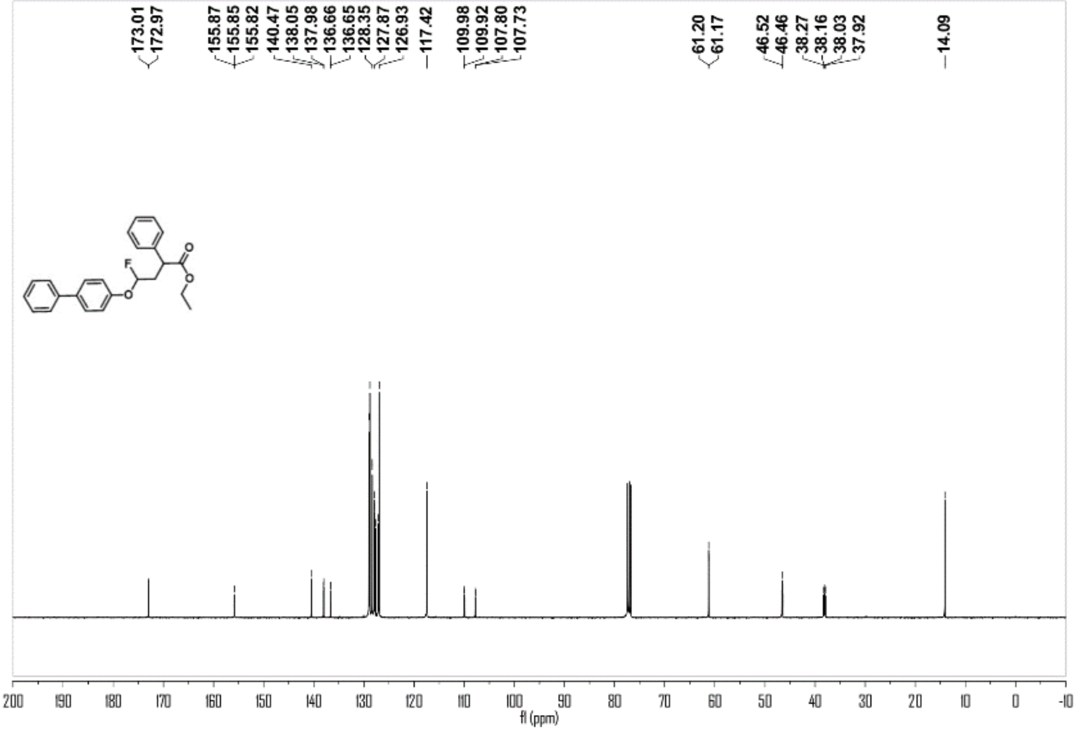
173.01
172.97
155.87
155.85
155.82
140.47
138.05
137.98
136.66
136.65
128.35
127.87
126.93
117.42
109.98
109.92
107.80
107.73
61.20
61.17
46.52
46.46
38.27
38.16
38.03
37.92
14.09
f1 (ppm)

^{1}H, ^{19}F and ^{13}C NMR spectra of compound 3au

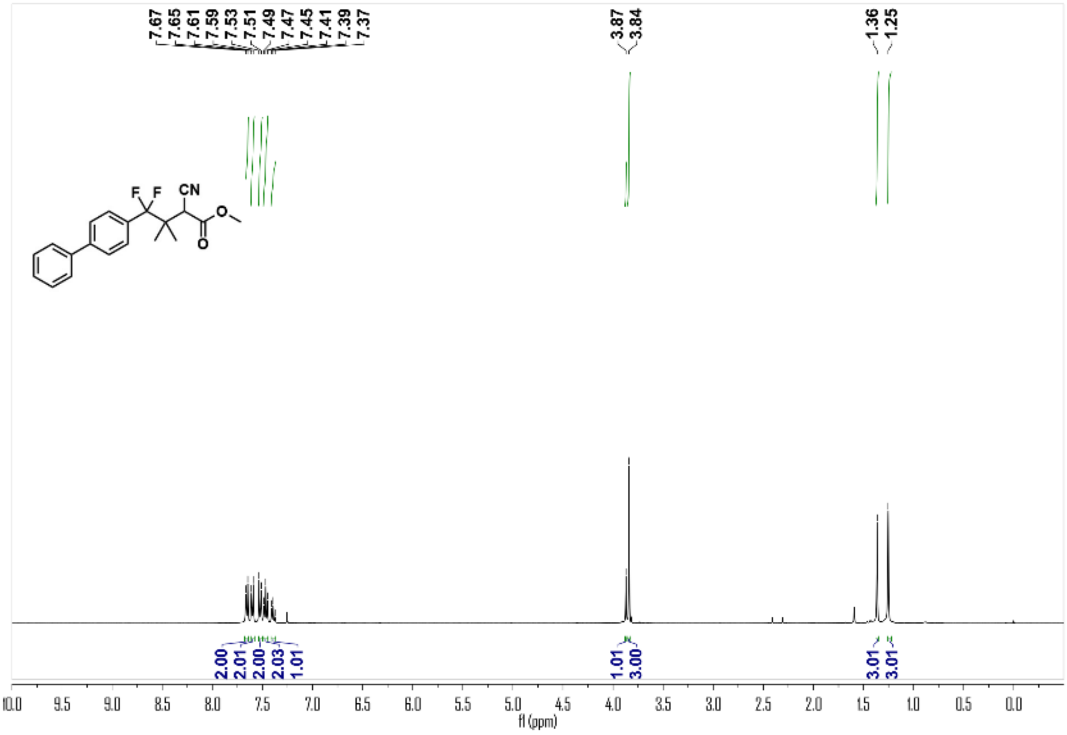

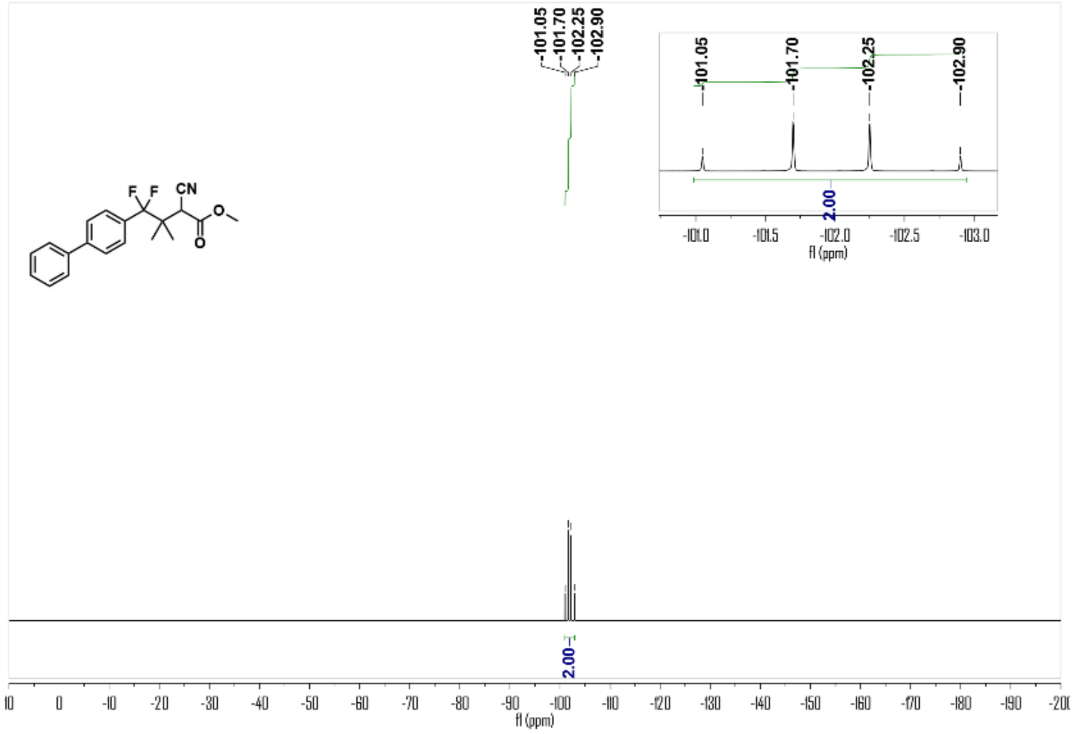

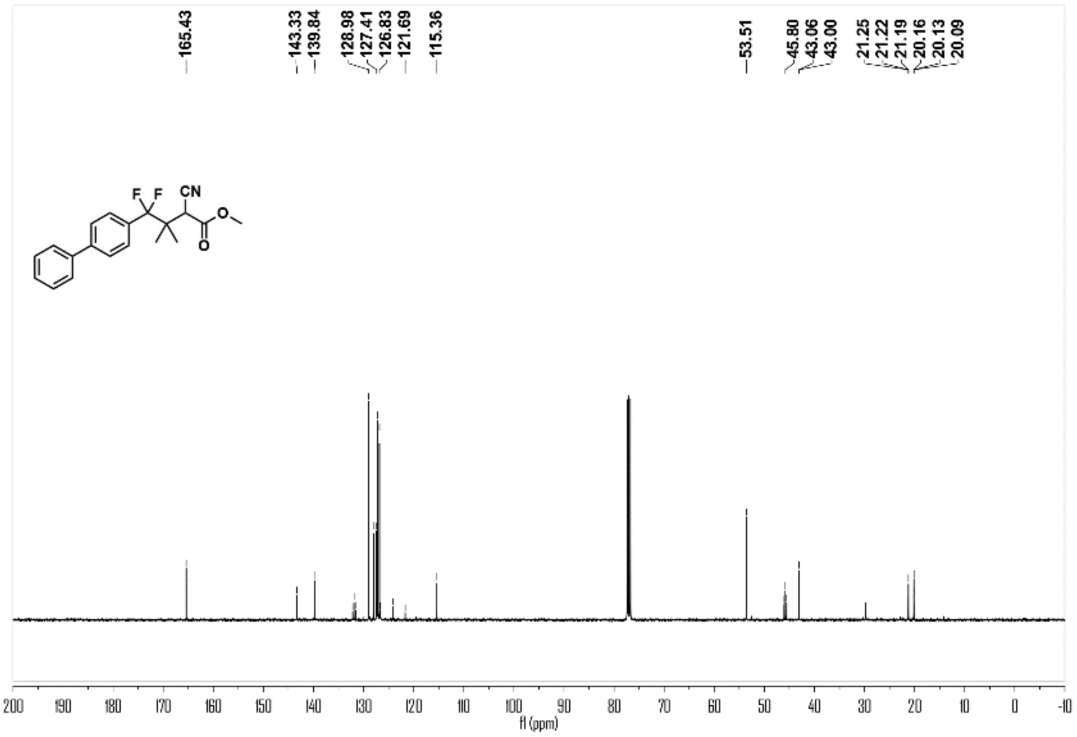

^{1}H, ^{19}F and ^{13}C NMR spectra of compound 3av

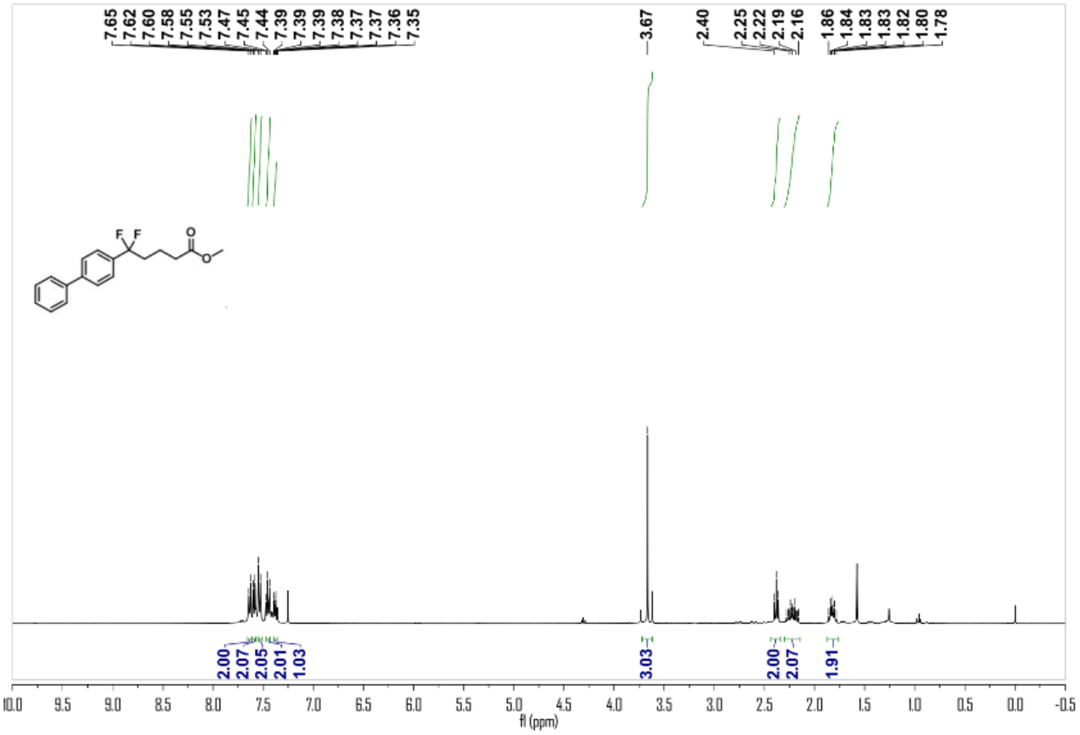

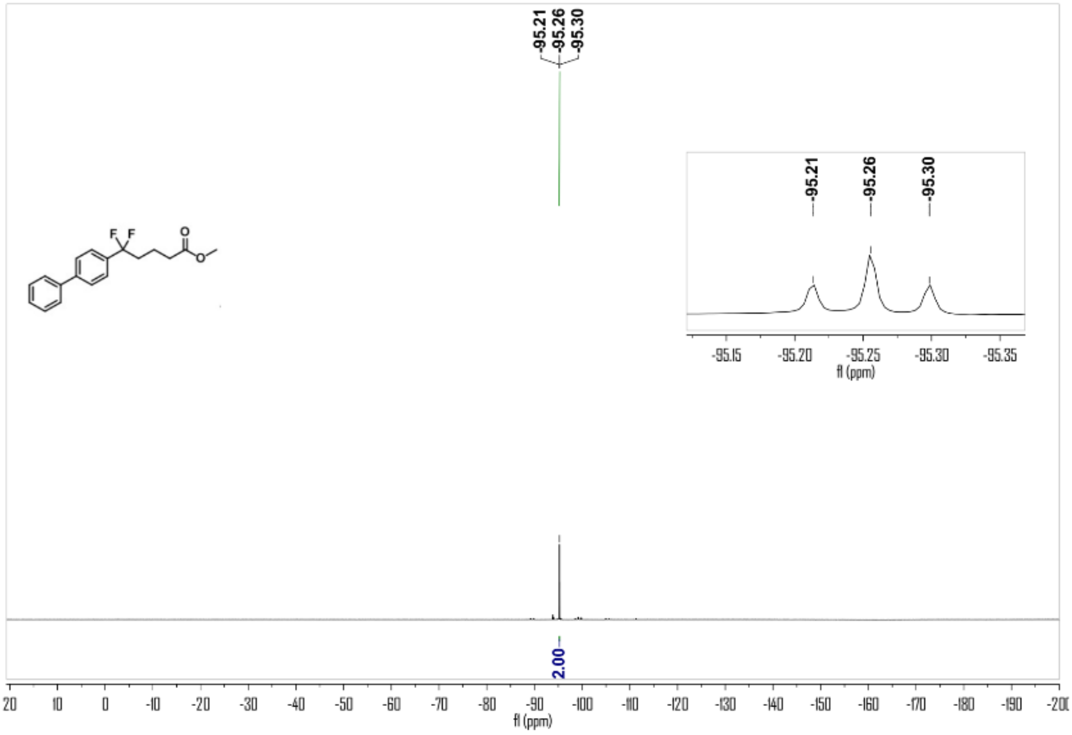
-95.21
-95.26
-95.30
2.00
fl (ppm)

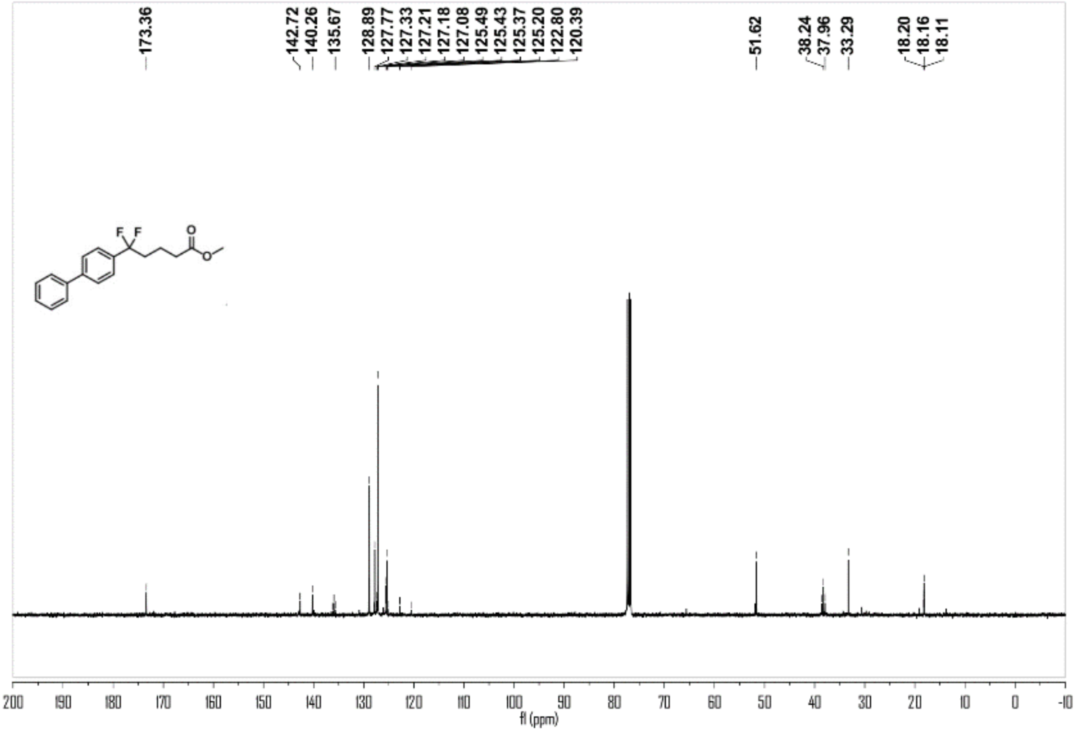
173.36
142.72
140.26
135.67
128.89
127.77
127.33
127.21
127.18
127.08
125.49
125.43
125.37
125.20
122.80
120.39
51.62
38.24
37.96
33.29
18.20
18.16
18.11
fl (ppm)

^{1}H, ^{19}F and ^{13}C NMR spectra of compound 3aw

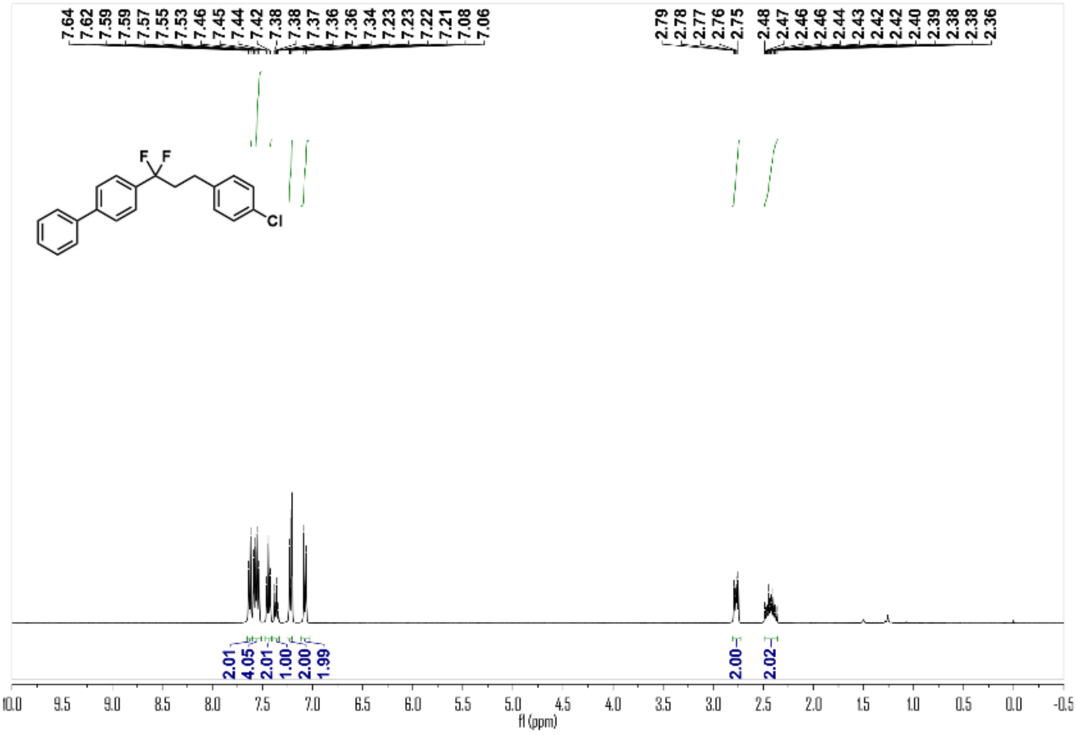

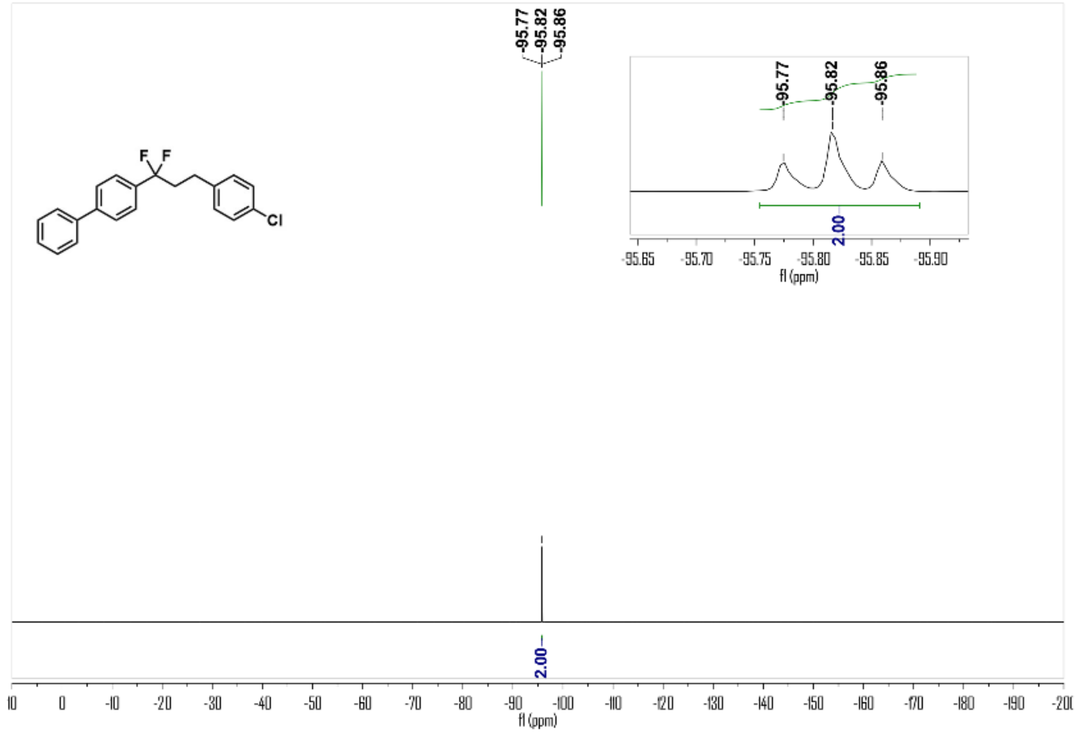

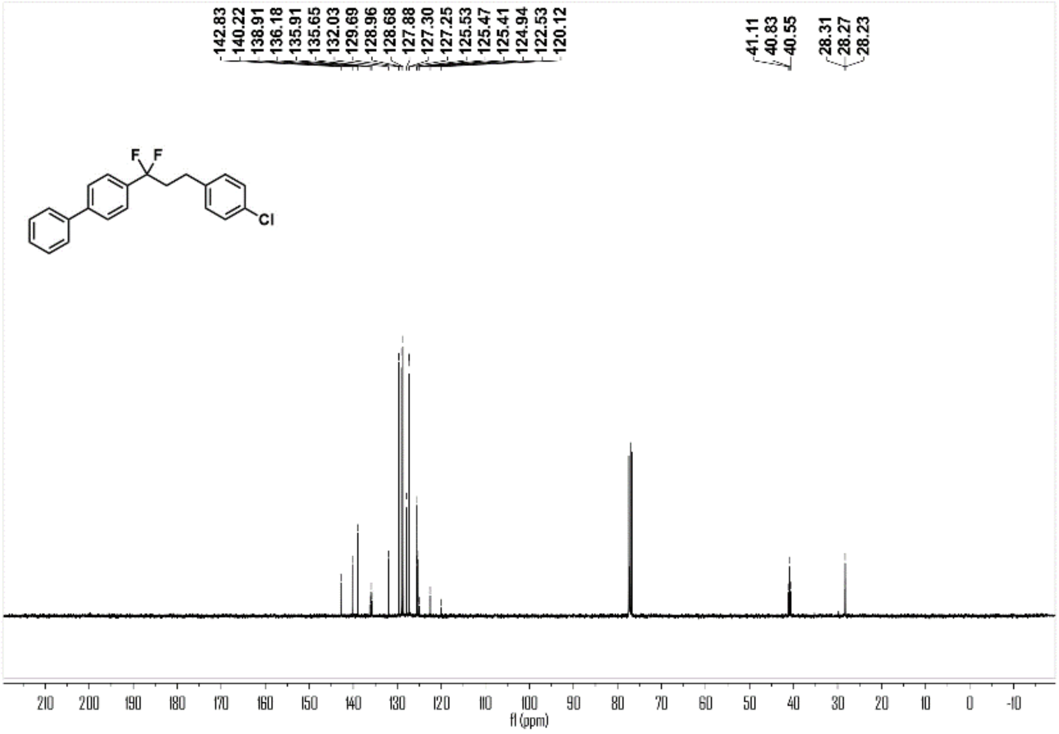

^{1}H, ^{19}F and ^{13}C NMR spectra of compound 3ax

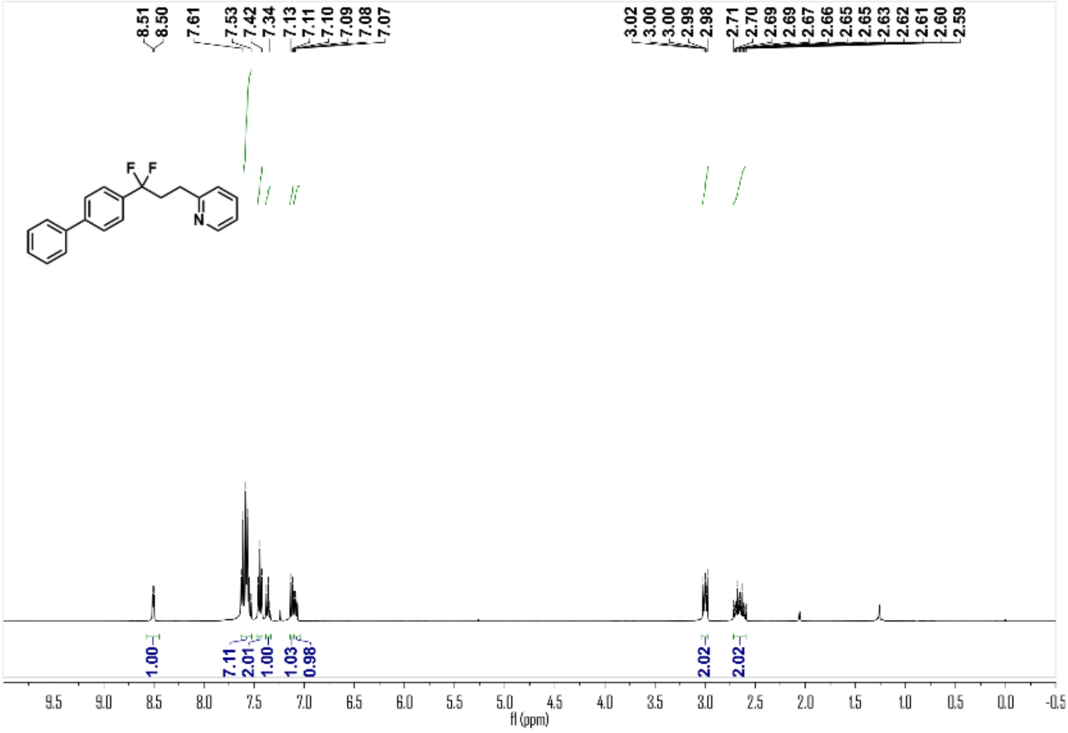

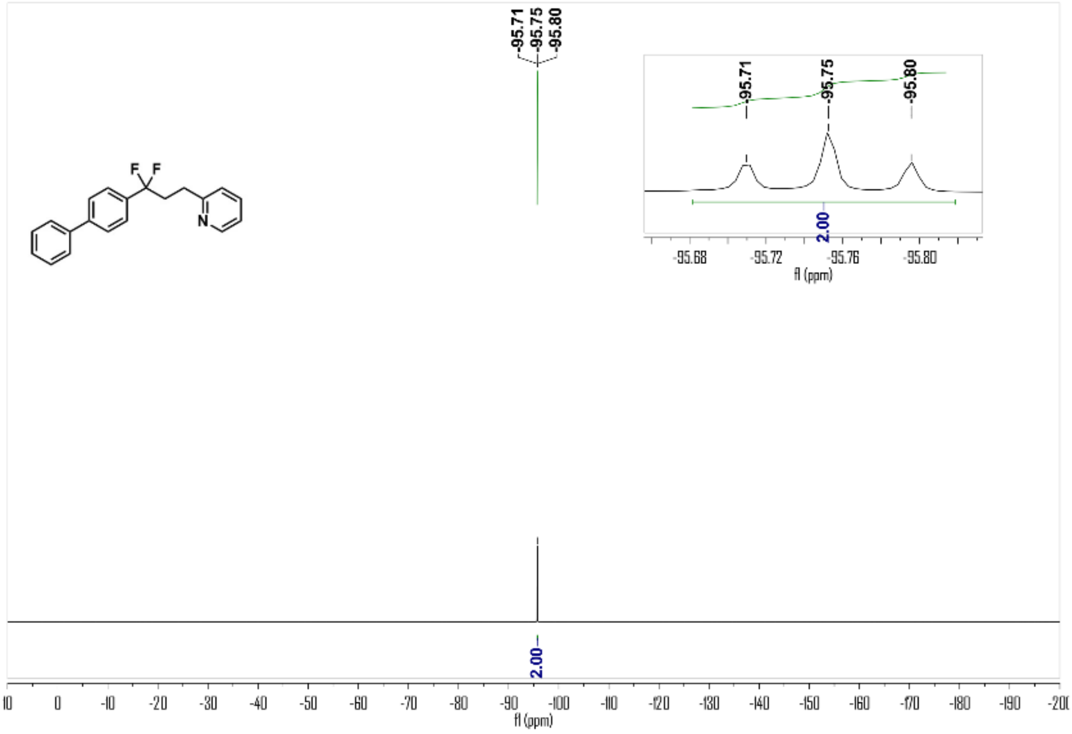

-95.71
-95.75
-95.80
2.00
fl (ppm)

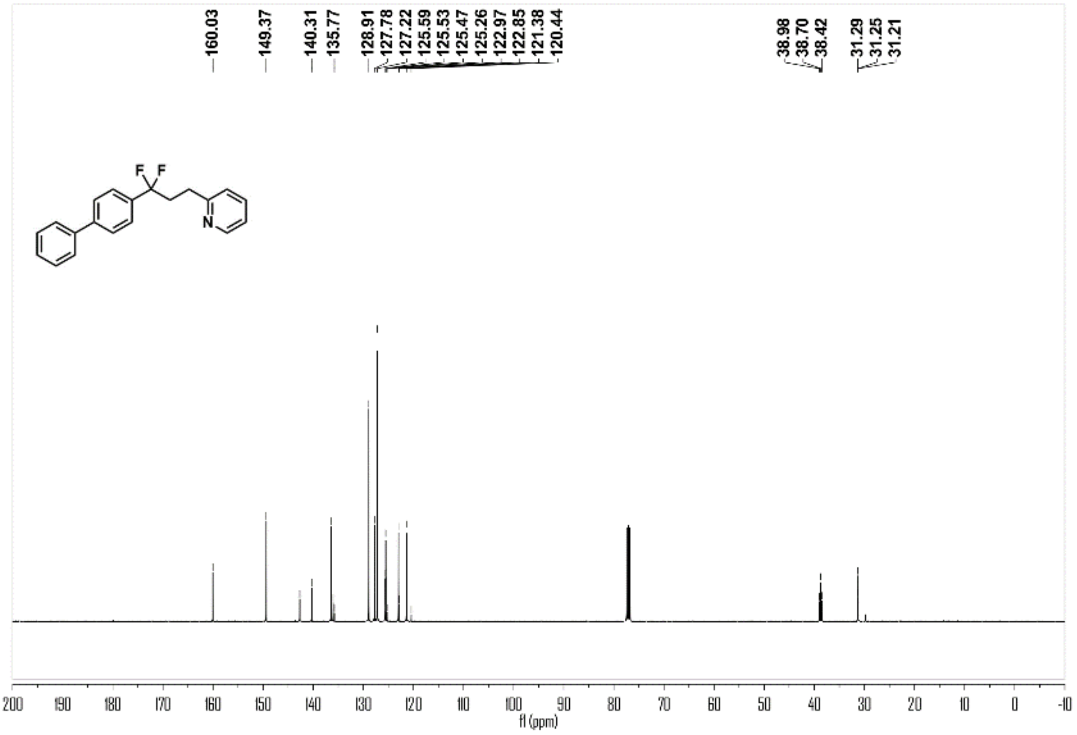

160.03
149.37
140.31
135.77
128.91
127.78
127.22
125.59
125.53
125.47
125.26
122.97
122.85
121.38
120.44
38.98
38.70
38.42
31.29
31.25
31.21
fl (ppm)

^{1}H, ^{19}F and ^{13}C NMR spectra of compound 3ay

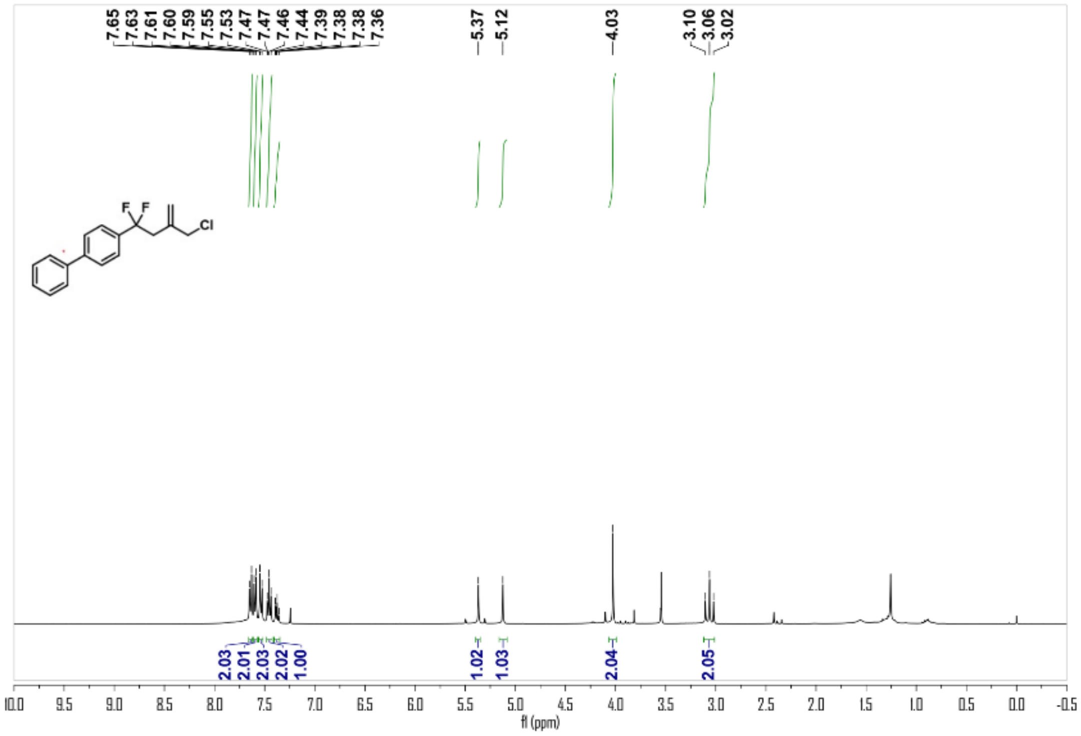

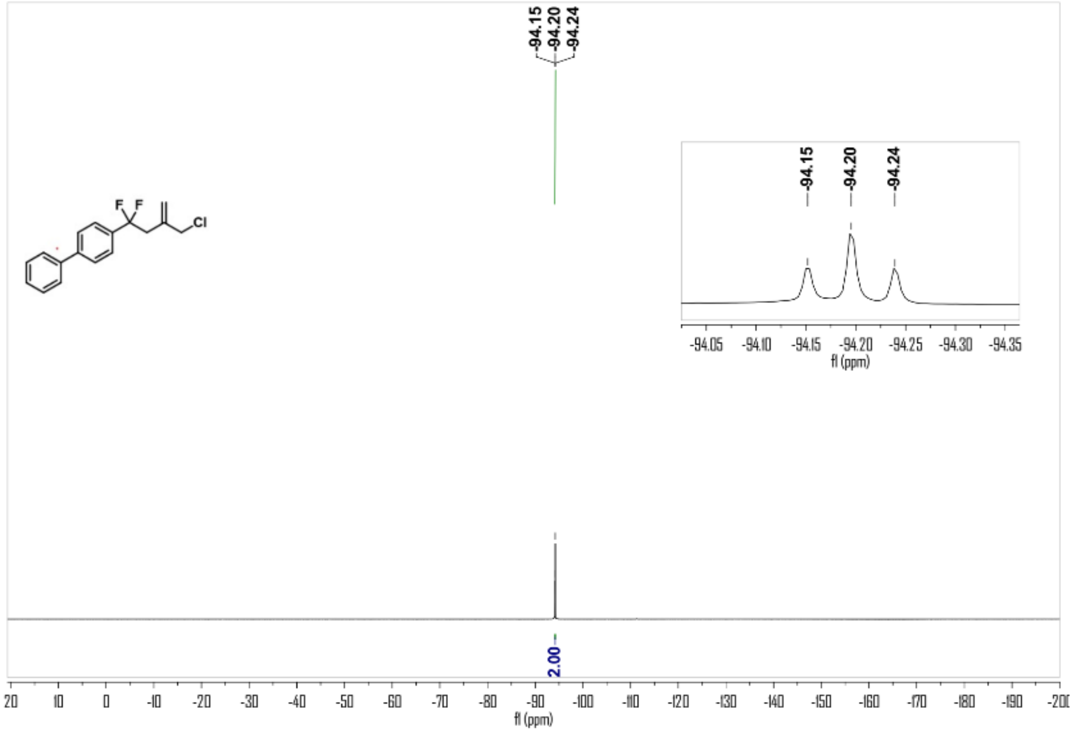

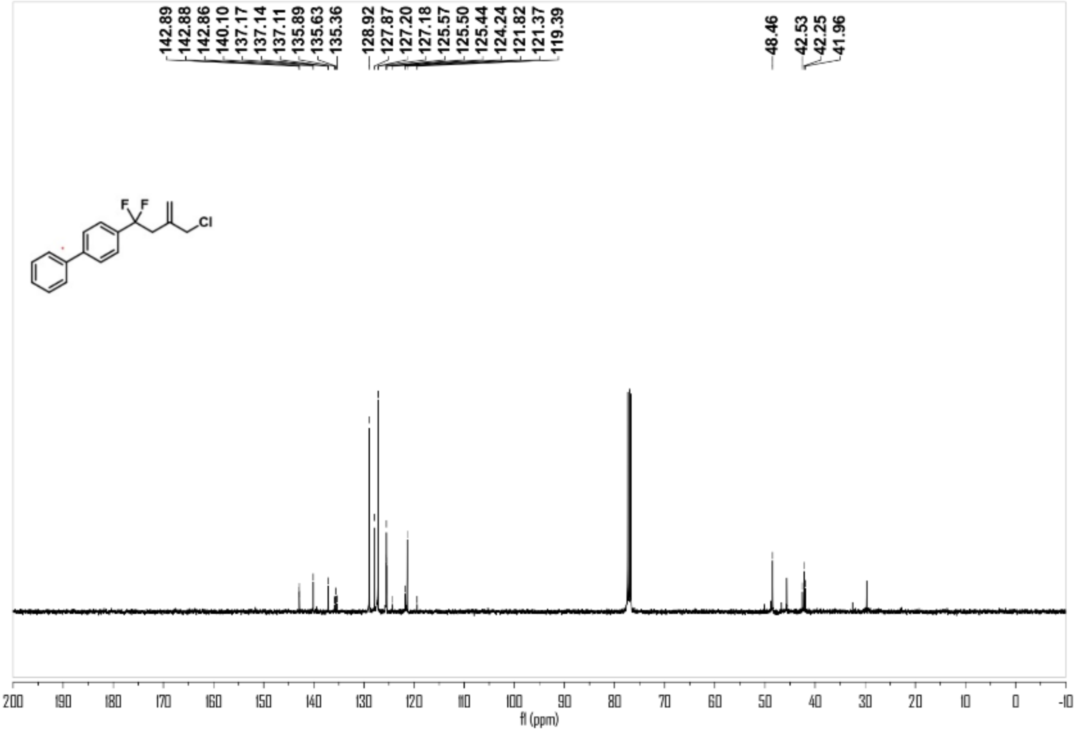

^{1}H, ^{19}F and ^{13}C NMR spectra of compound 3ay'

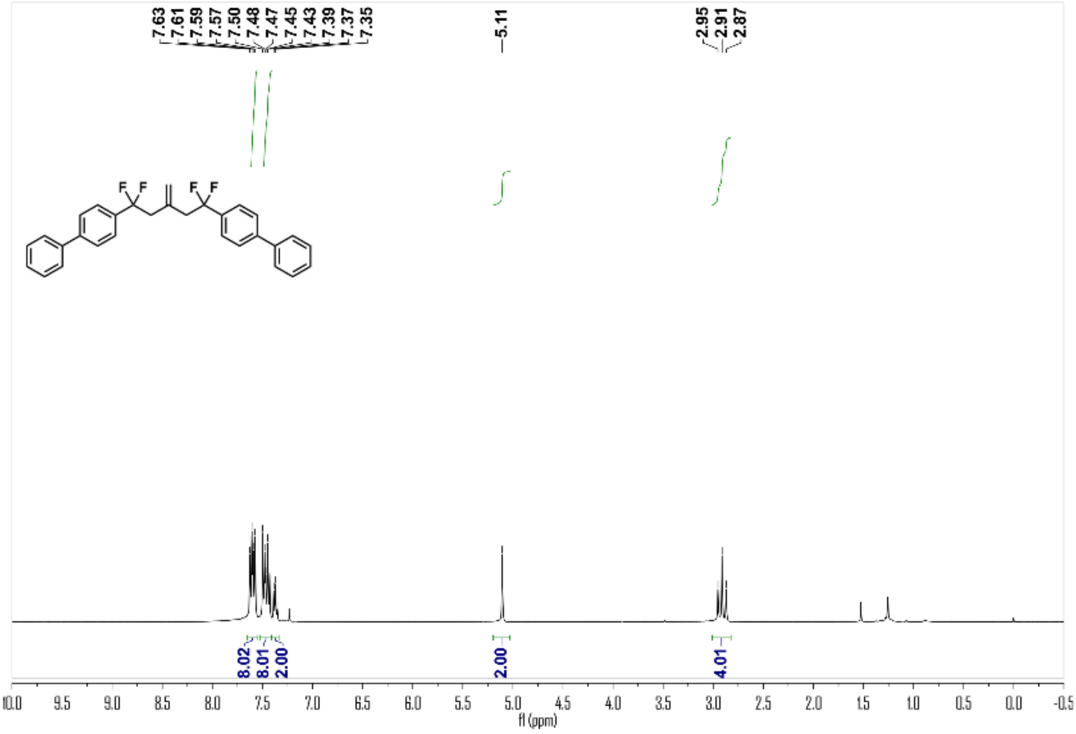

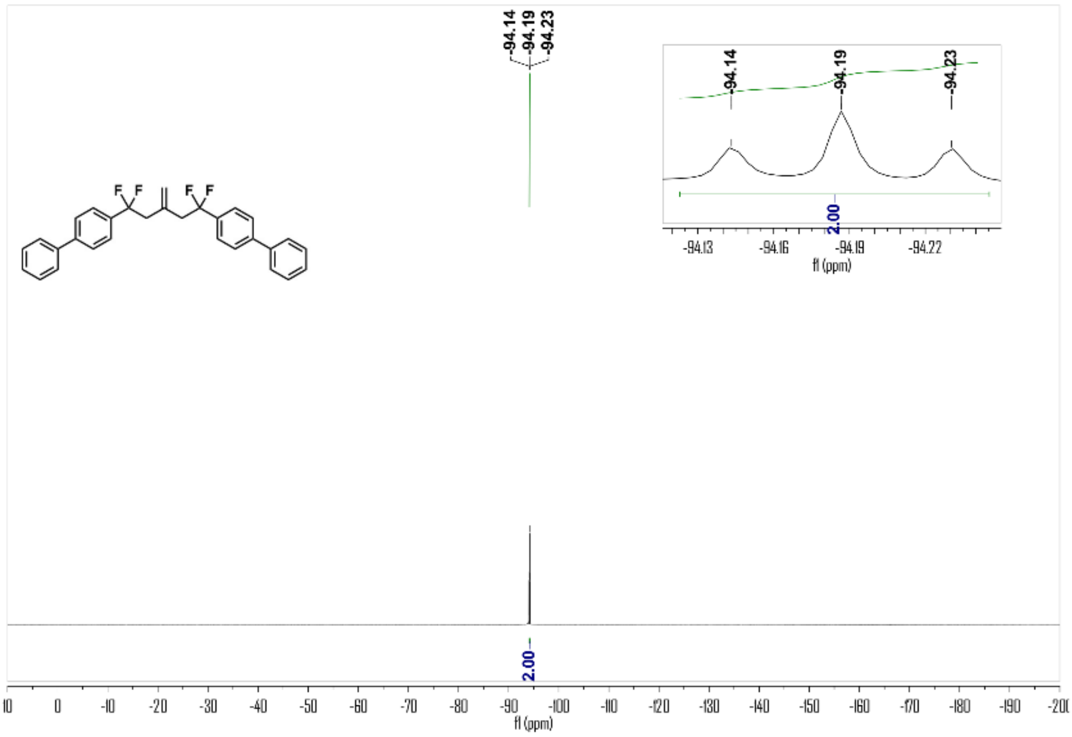
-94.14
-94.19
-94.23
2.00
-94.13
-94.16
-94.19
-94.22
f1 (ppm)
F F
F F
2.00
f1 (ppm)

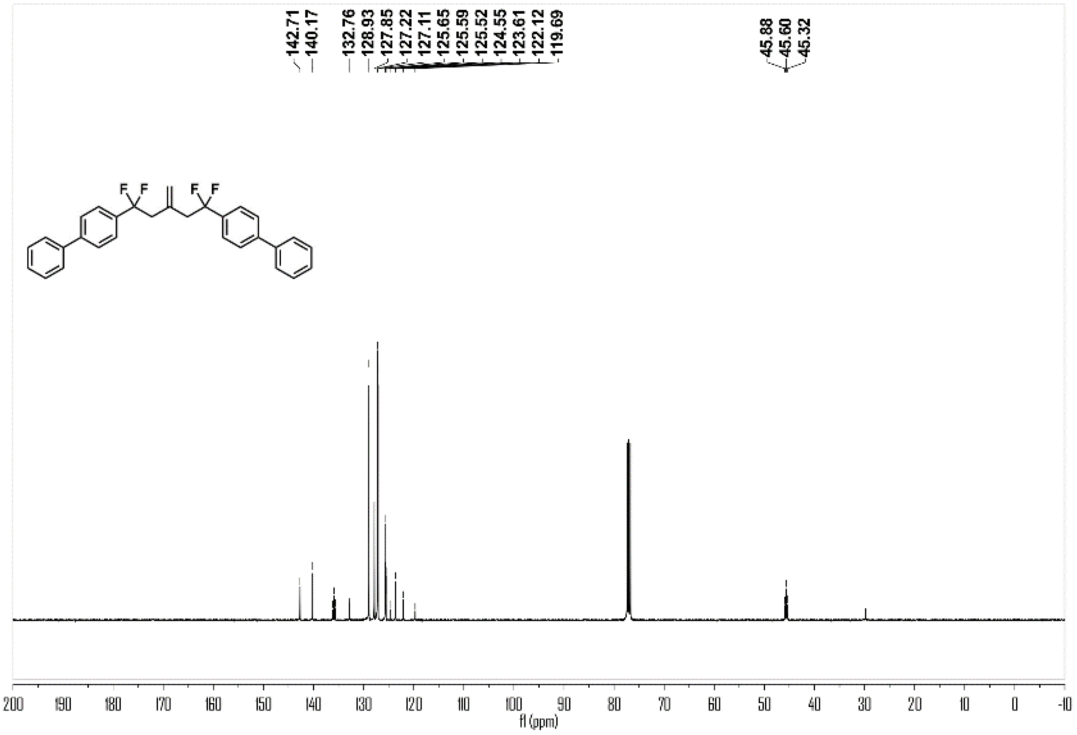
142.71
140.17
132.76
128.93
127.85
127.22
127.11
125.65
125.59
125.52
124.55
123.61
122.12
119.69
45.88
45.60
45.32
F F
F F
f1 (ppm)

^{1}H, ^{19}F and ^{13}C NMR spectra of compound 3az

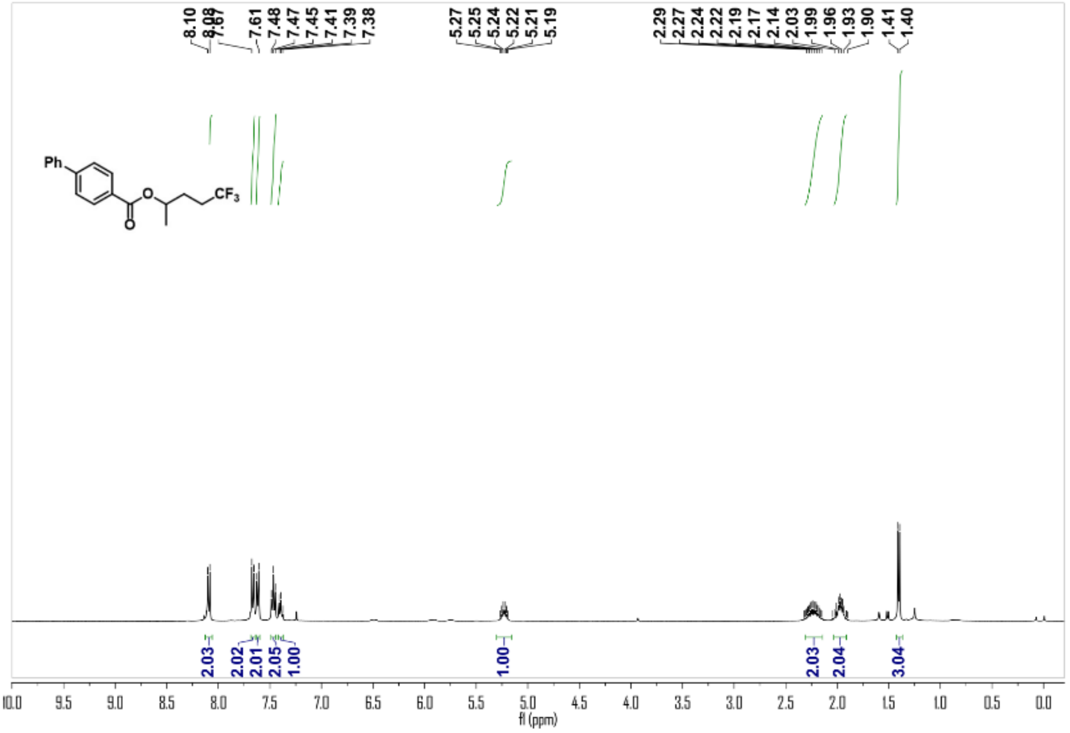

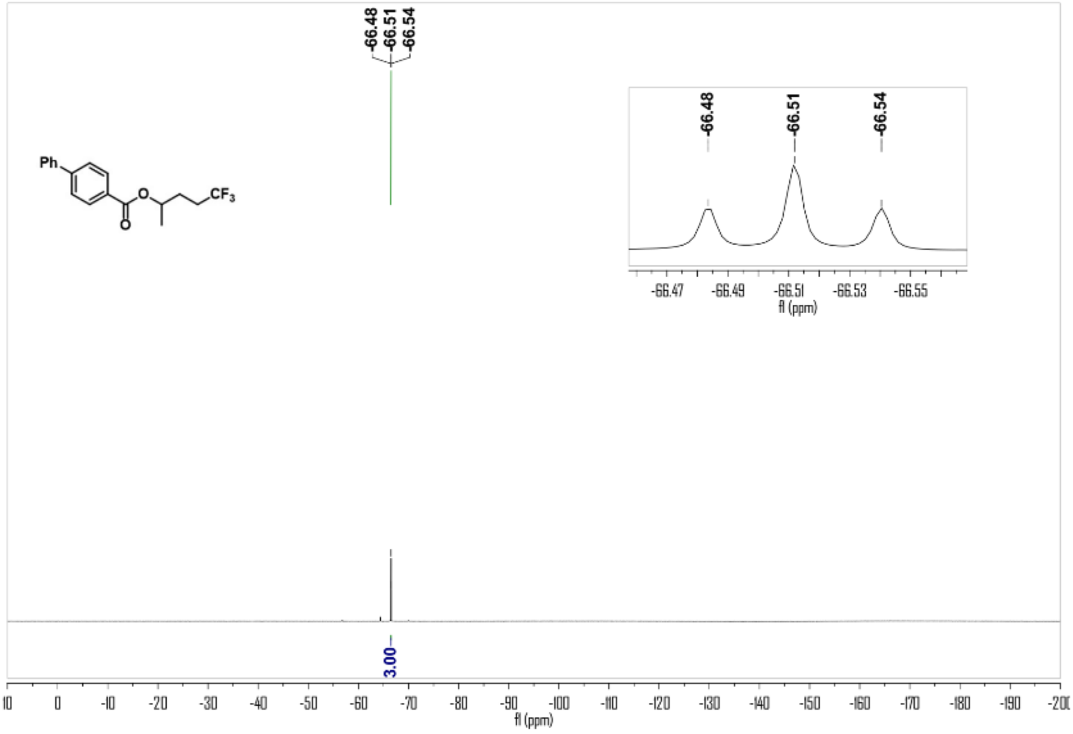

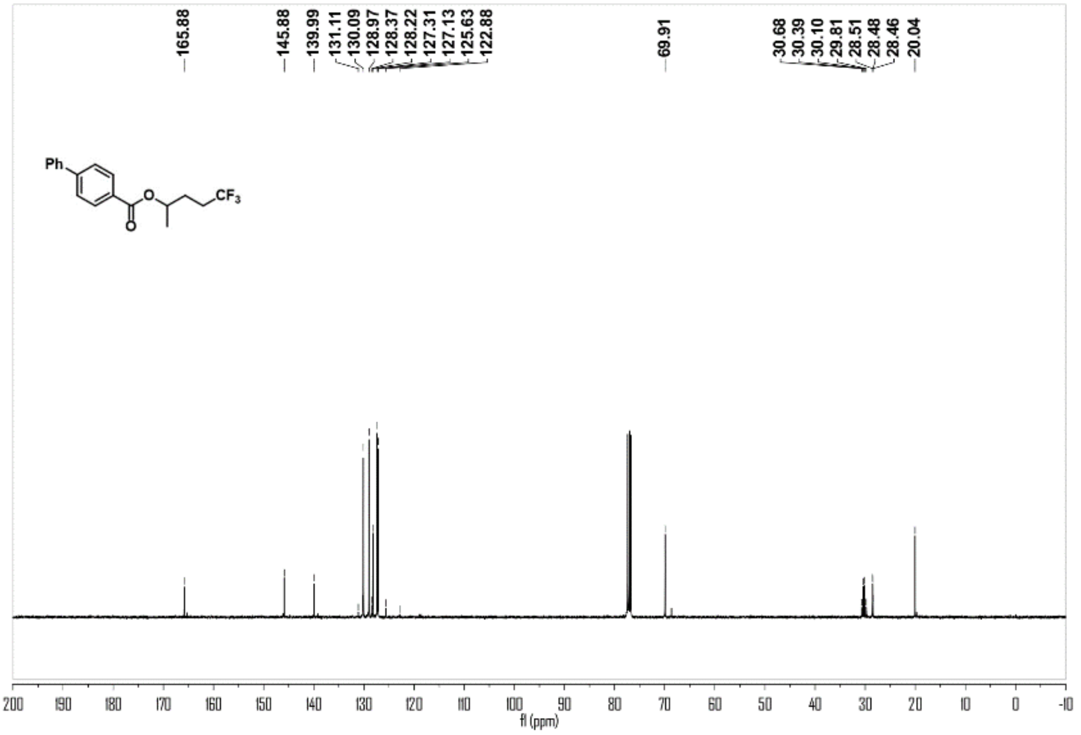

^{1}H, ^{19}F and ^{13}C NMR spectra of compound 3ba

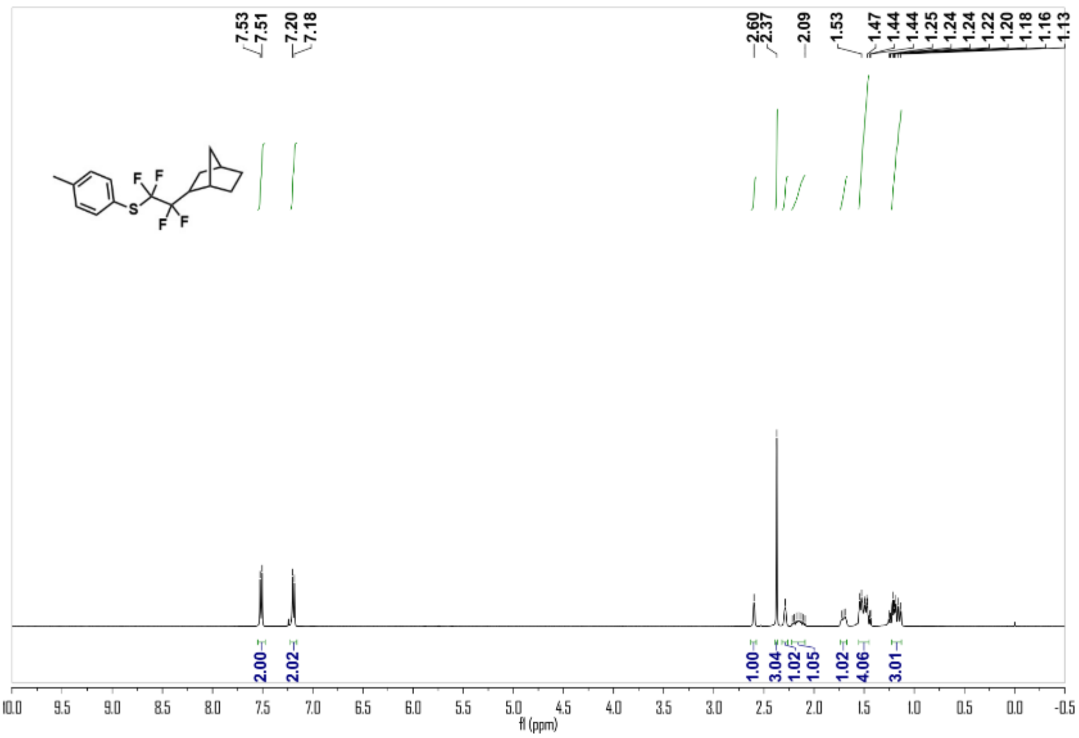

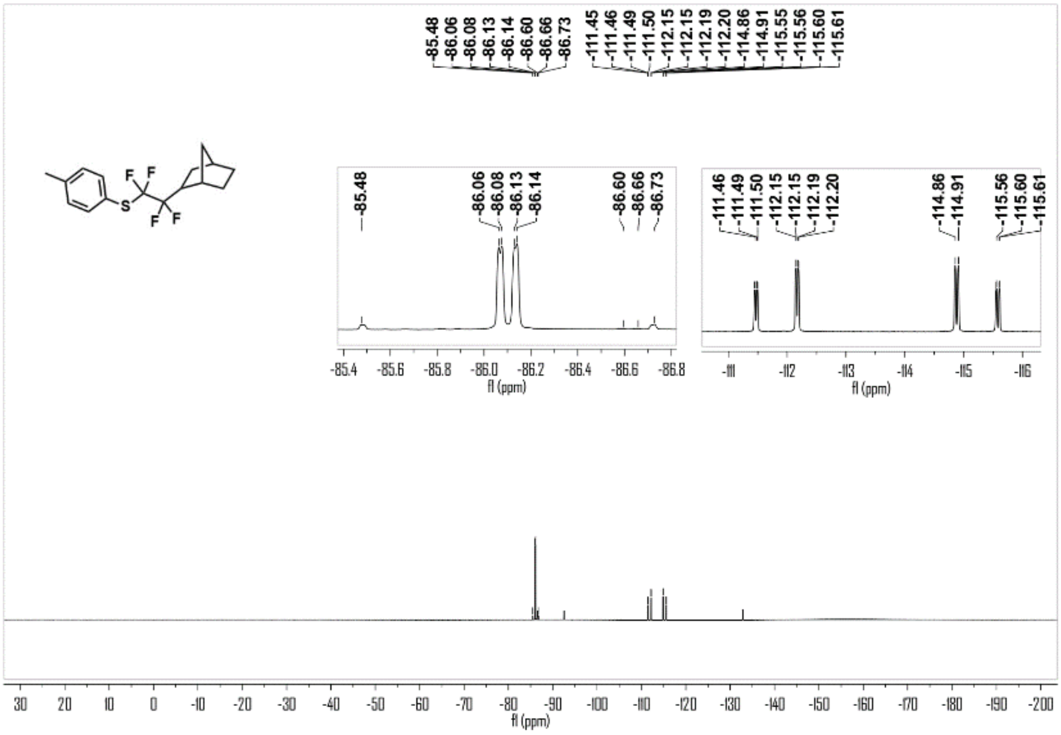
-85.48
-86.06
-86.08
-86.13
-86.14
-86.60
-86.66
-86.73
-111.45
-111.46
-111.49
-111.50
-112.15
-112.15
-112.19
-112.20
-114.86
-114.91
-115.55
-115.56
-115.60
-115.61
F F
S
F F
f1 (ppm)

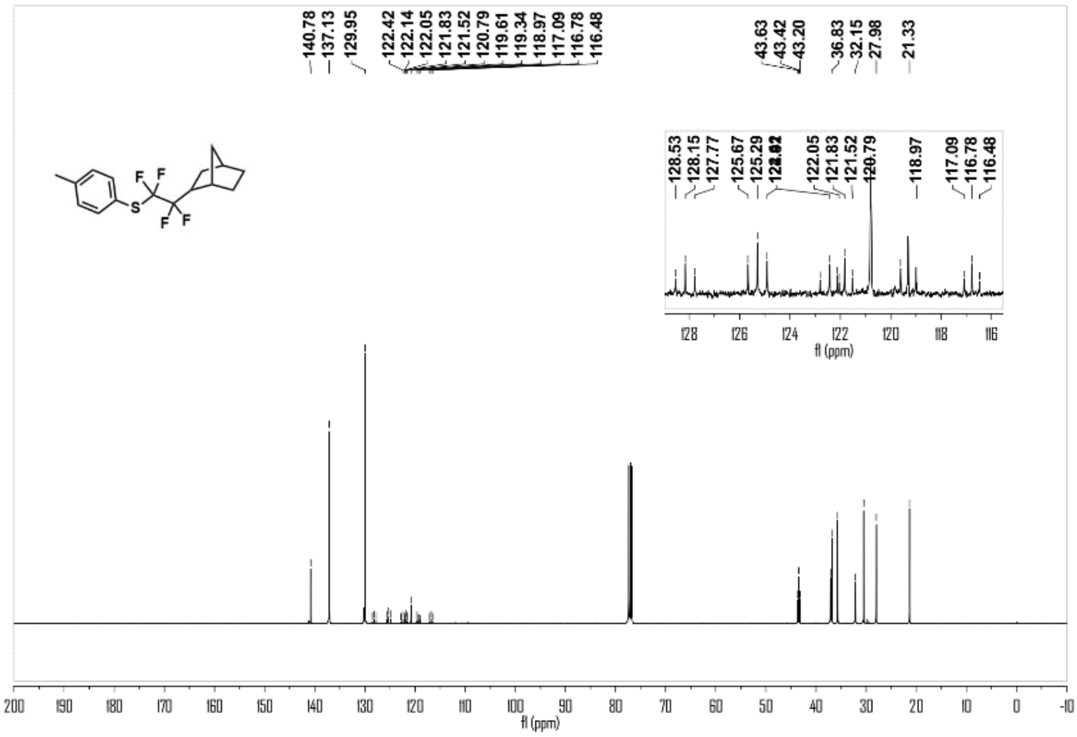
140.78
137.13
129.95
122.42
122.14
122.05
121.83
121.52
120.79
119.61
119.34
118.97
117.09
116.78
116.48
43.63
43.42
43.20
36.83
32.15
27.98
21.33
128.53
128.15
127.77
125.67
125.29
122.05
121.83
121.52
120.79
118.97
117.09
116.78
116.48
F F
S
F F
f1 (ppm)

^{1}H, ^{19}F and ^{13}C NMR spectra of compound 3bb

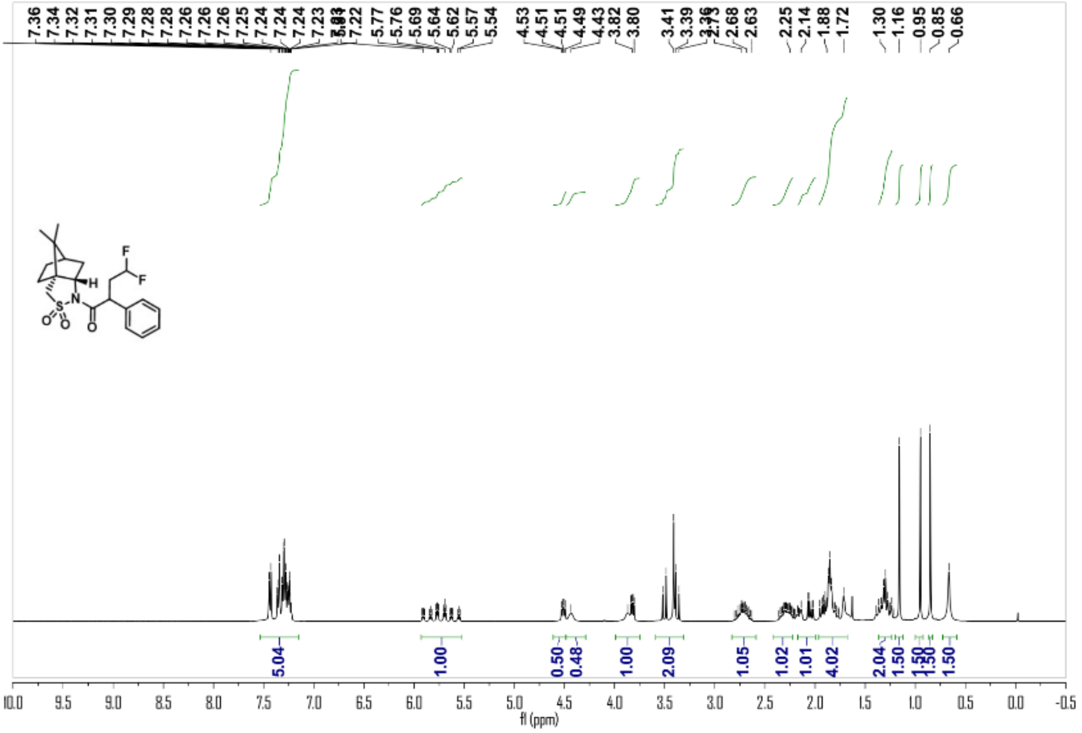

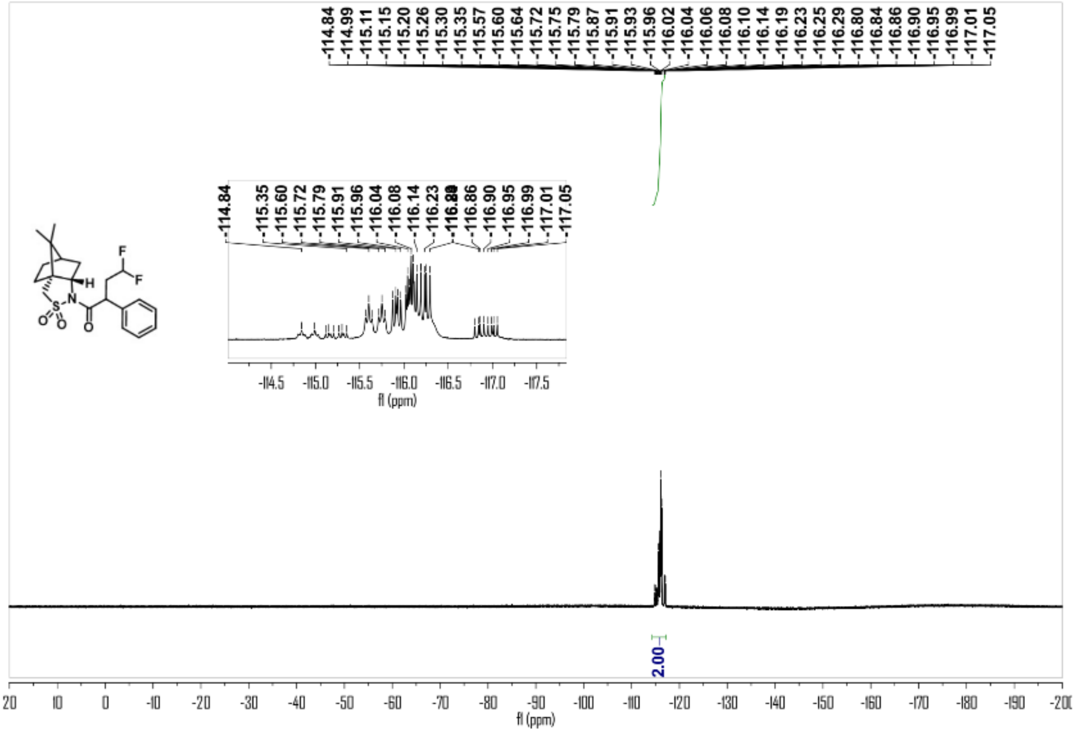

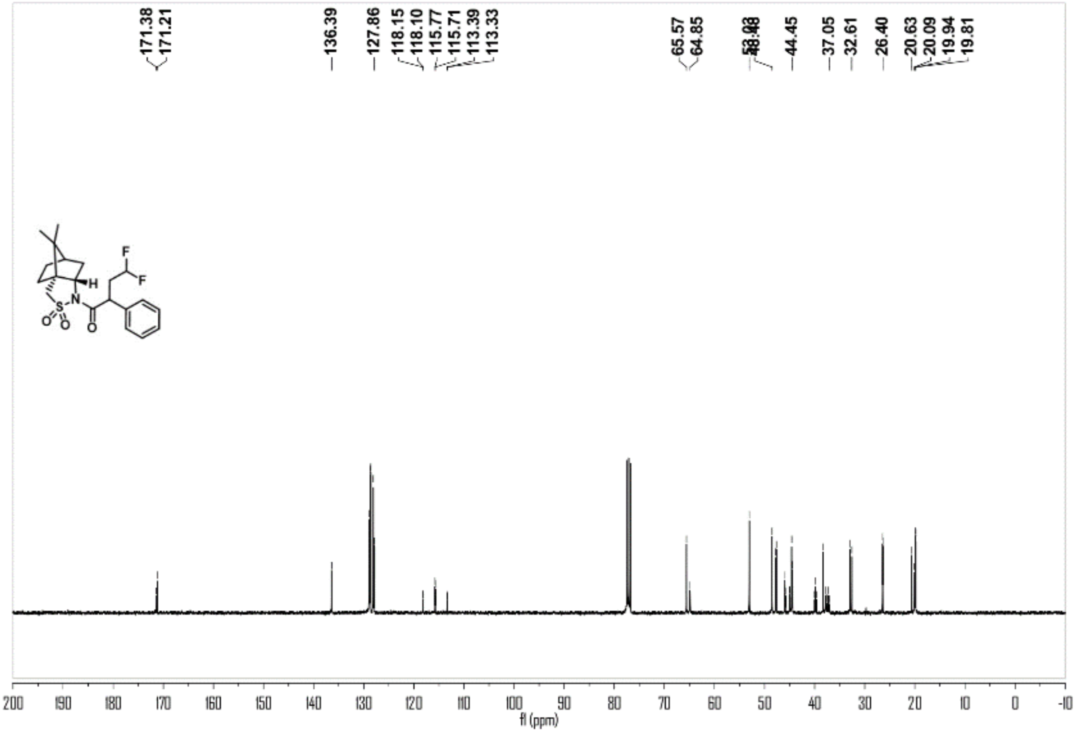

^{1}H, ^{19}F and ^{13}C NMR spectra of compound 3bc

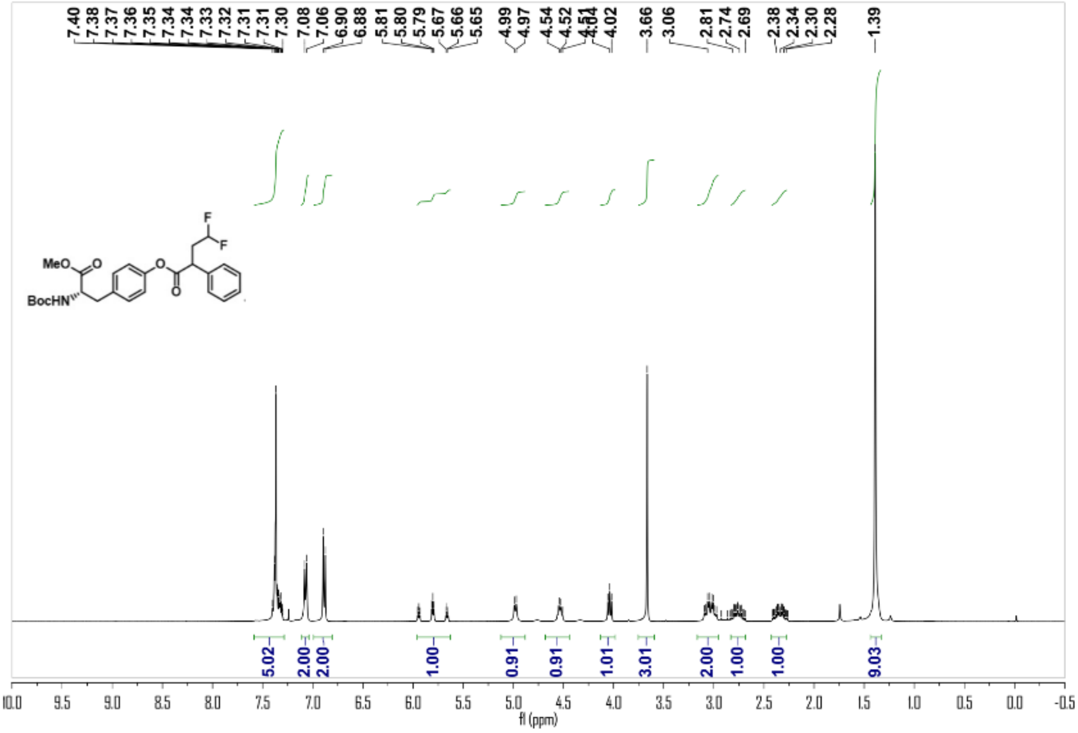

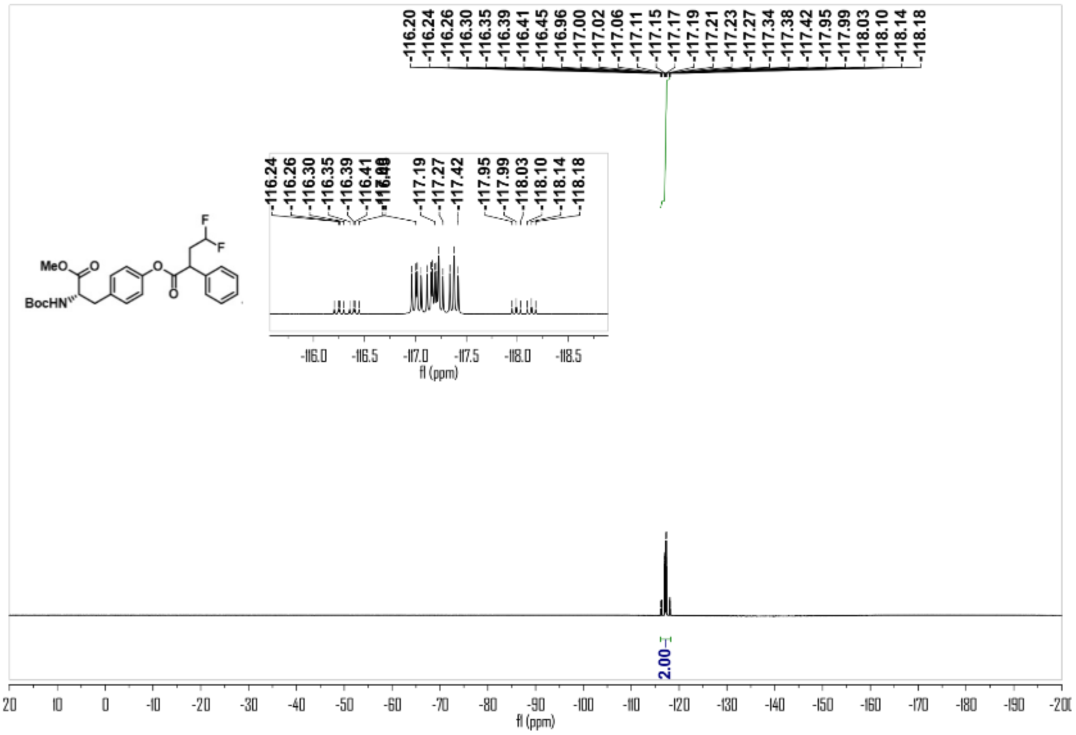

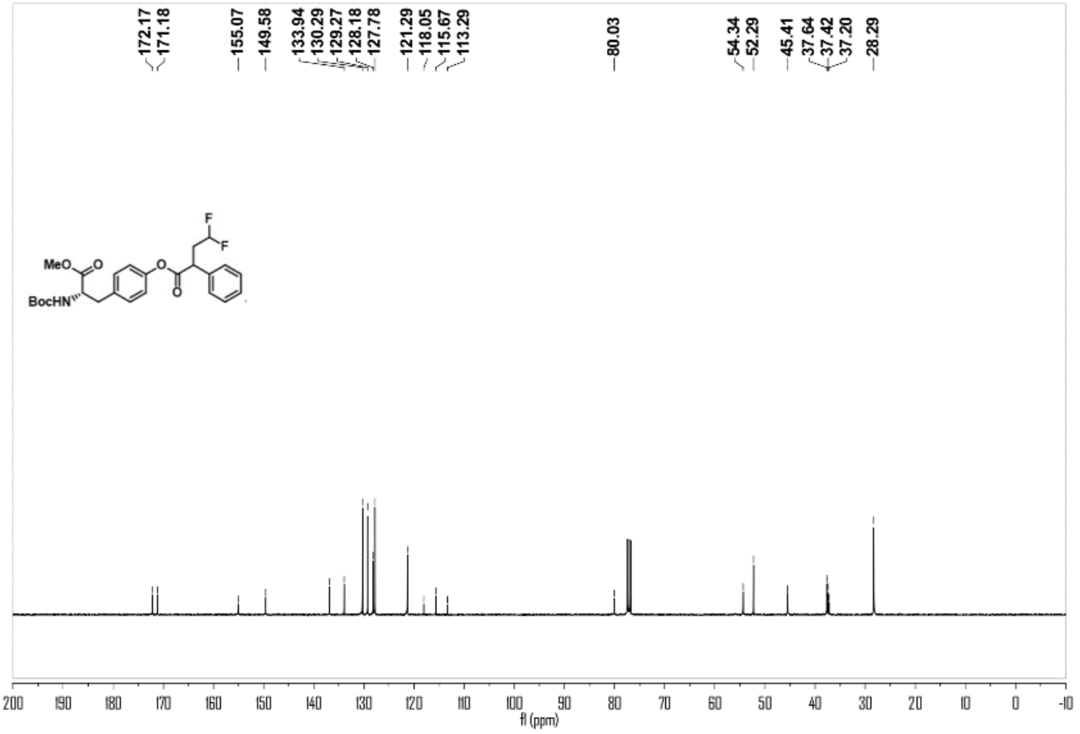

^{1}H, ^{19}F and ^{13}C NMR spectra of compound 3bd

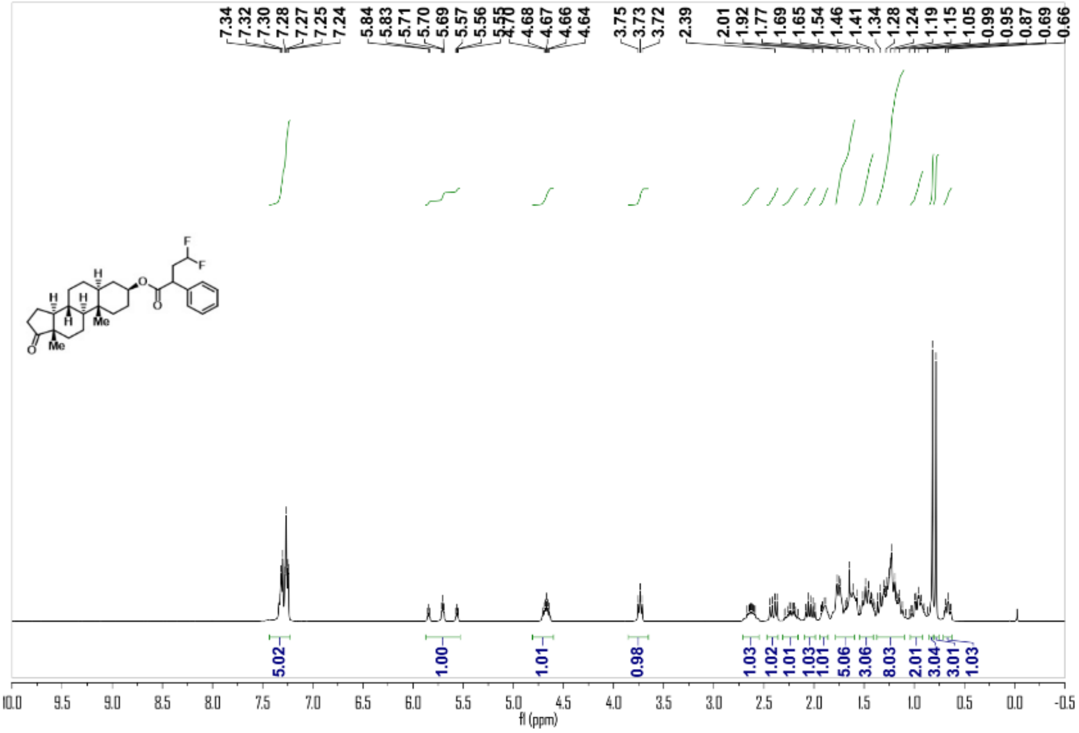

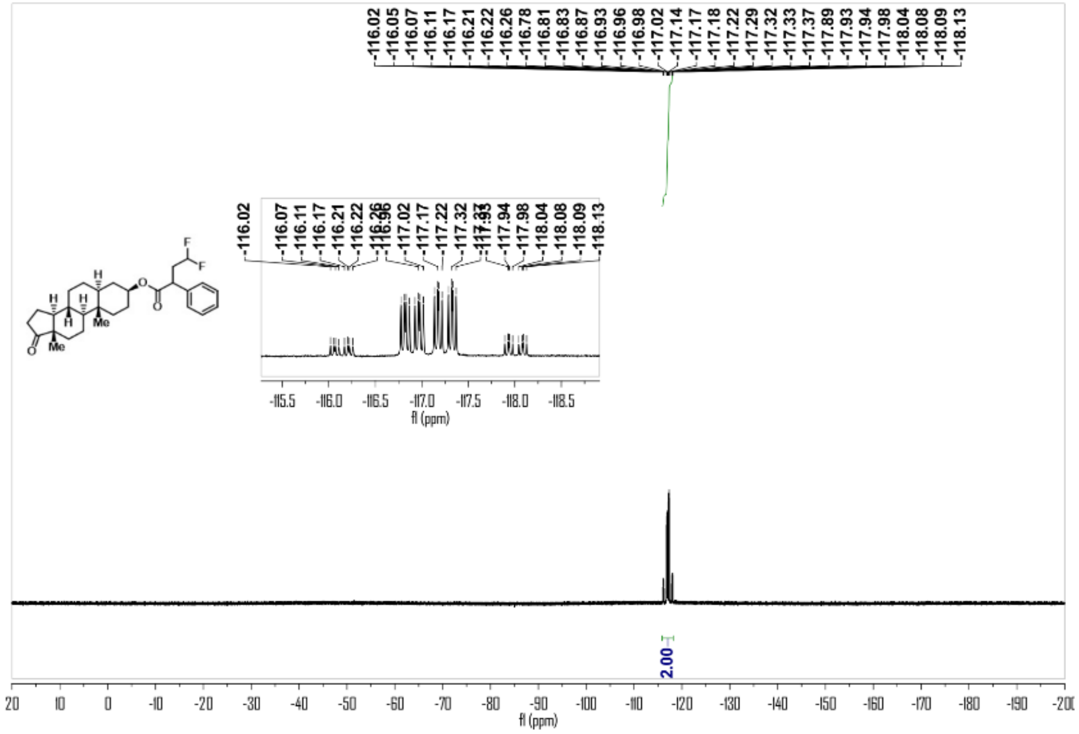

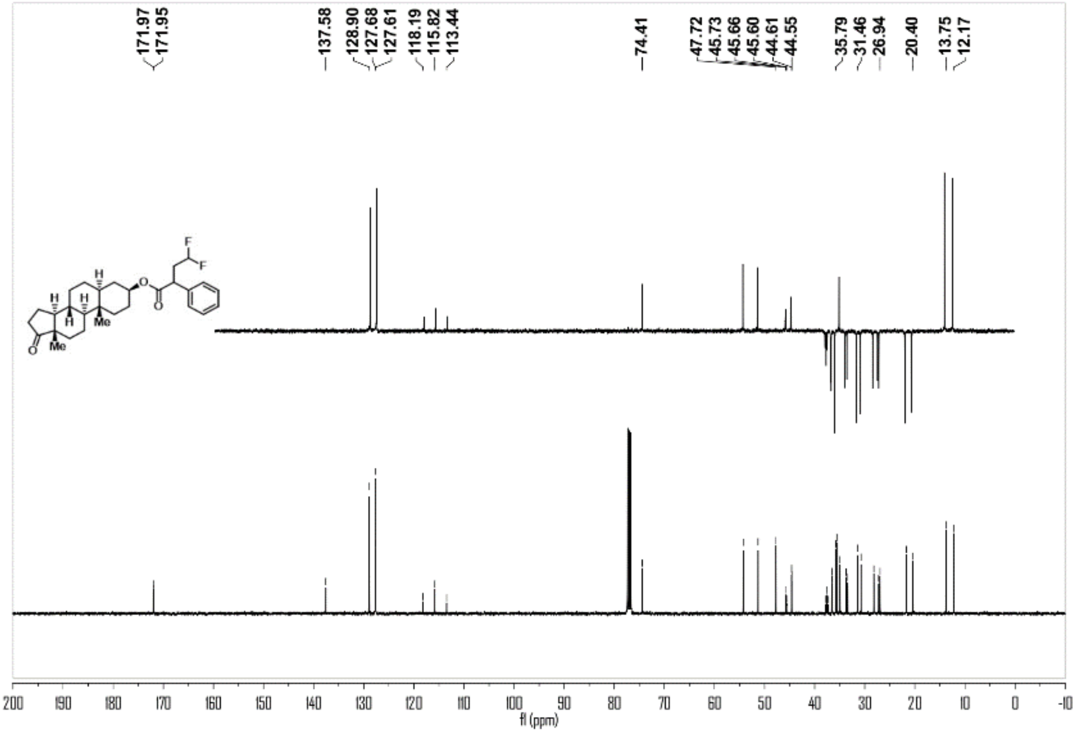

^{1}H, ^{19}F and ^{13}C NMR spectra of compound 3be

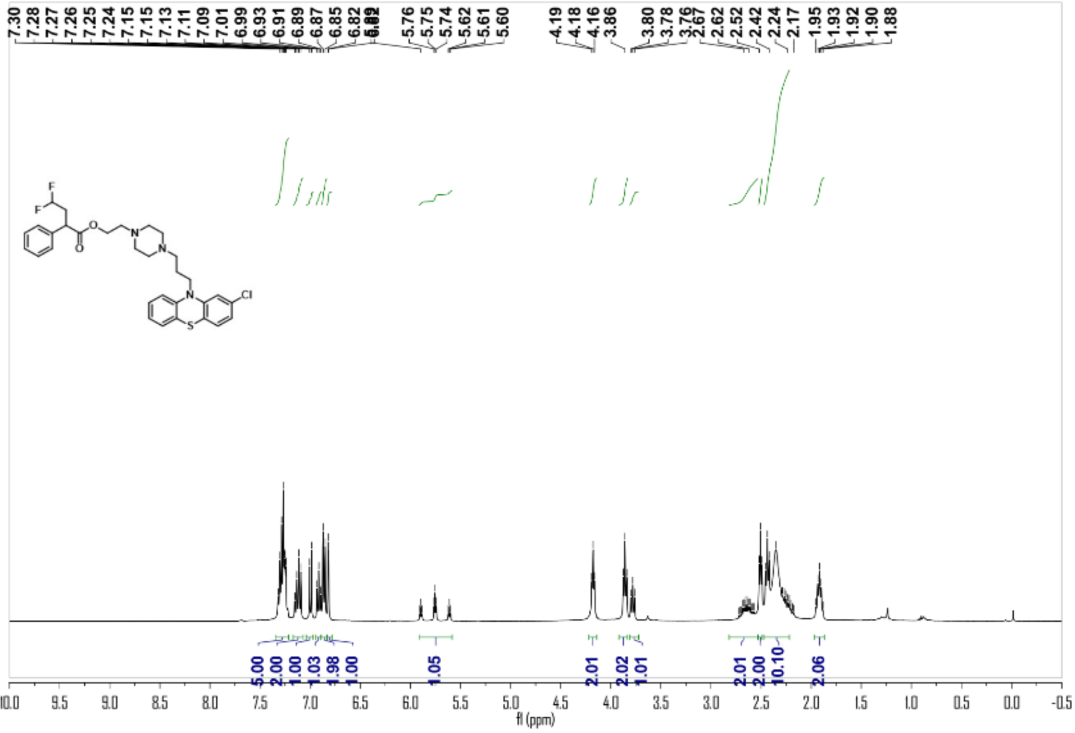

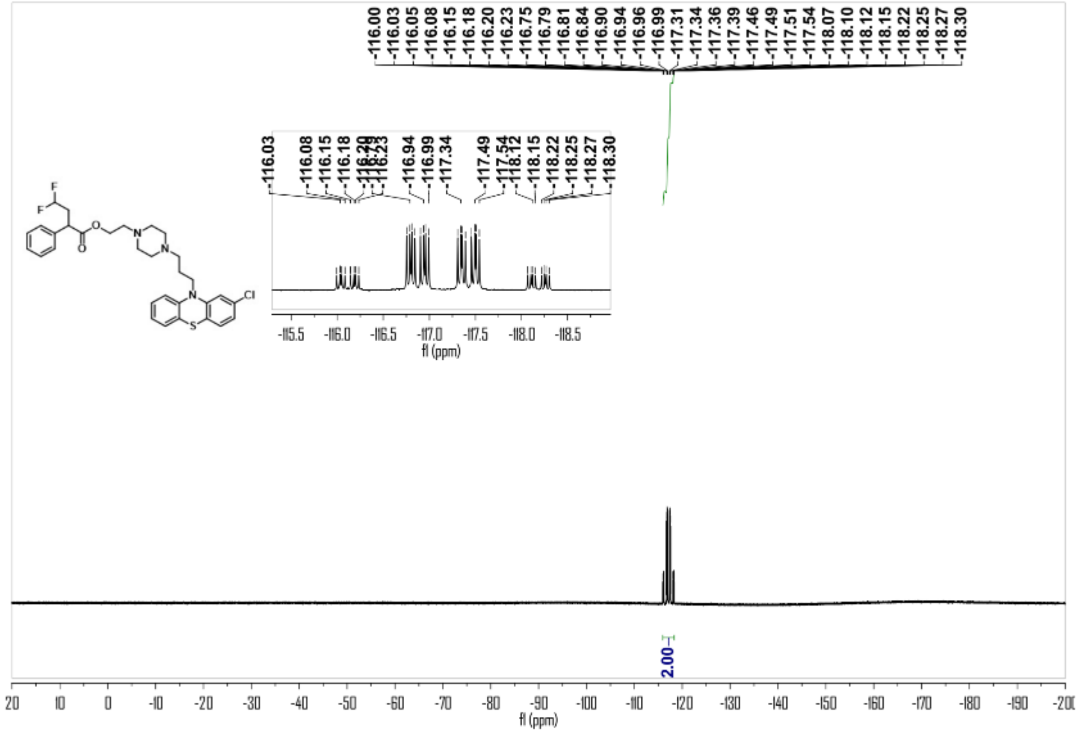

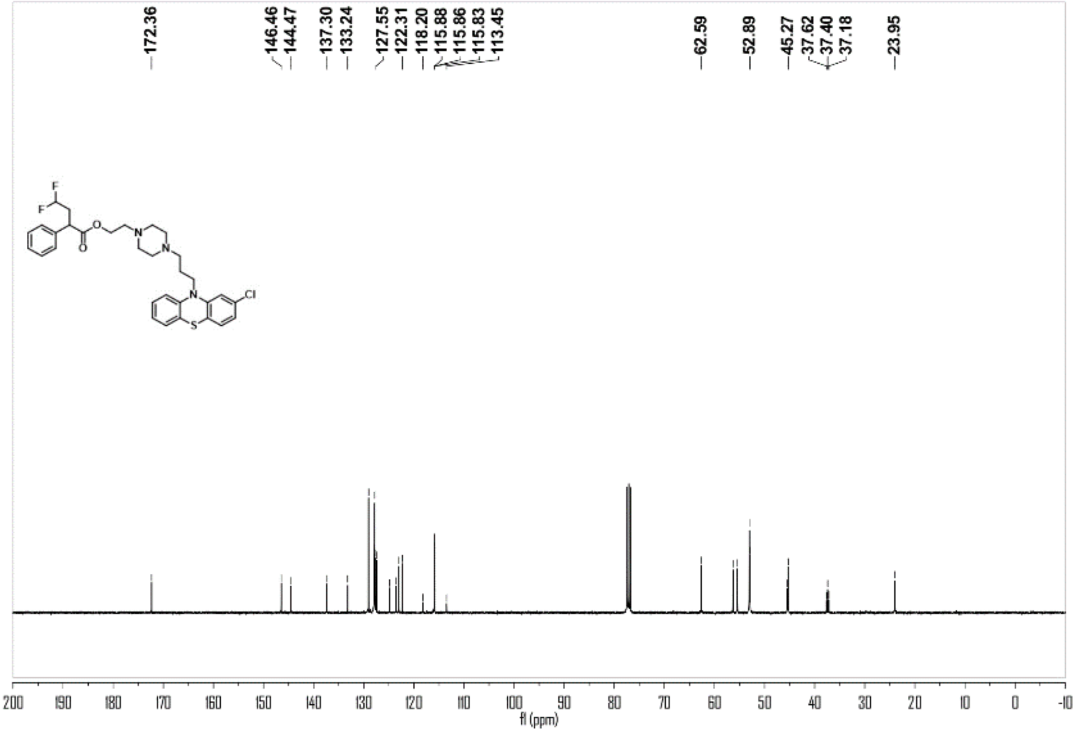

^{1}H, ^{19}F and ^{13}C NMR spectra of compound 3bf

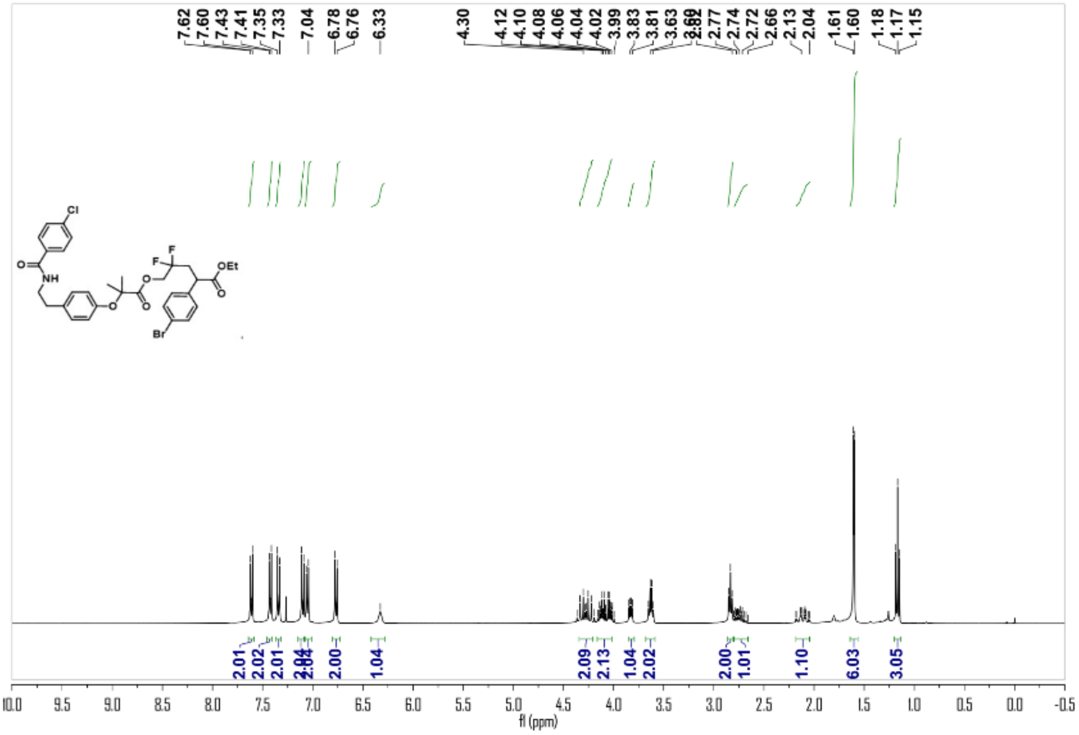

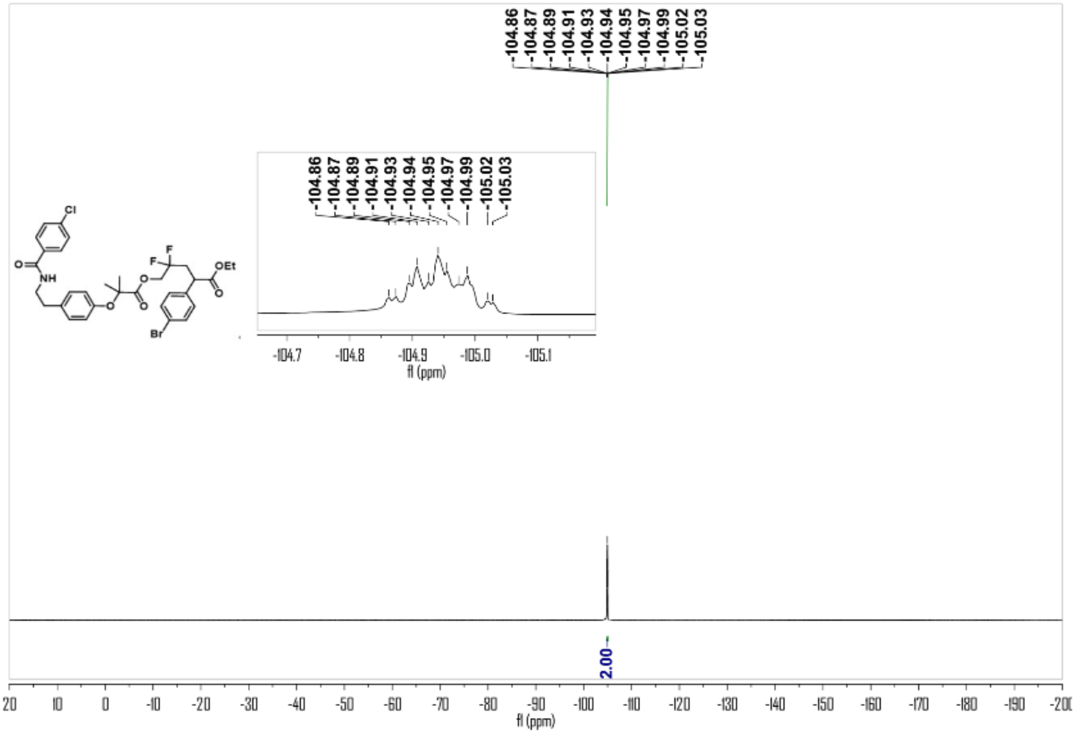

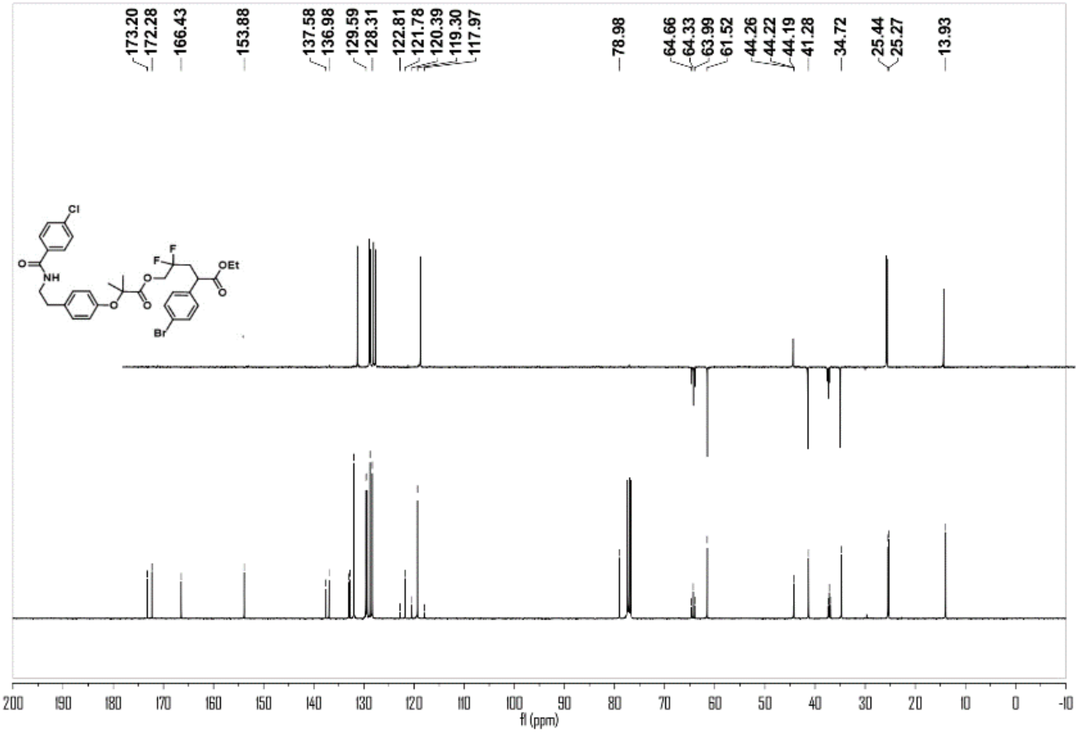

^{1}H, ^{19}F and ^{13}C NMR spectra of compound 3bg

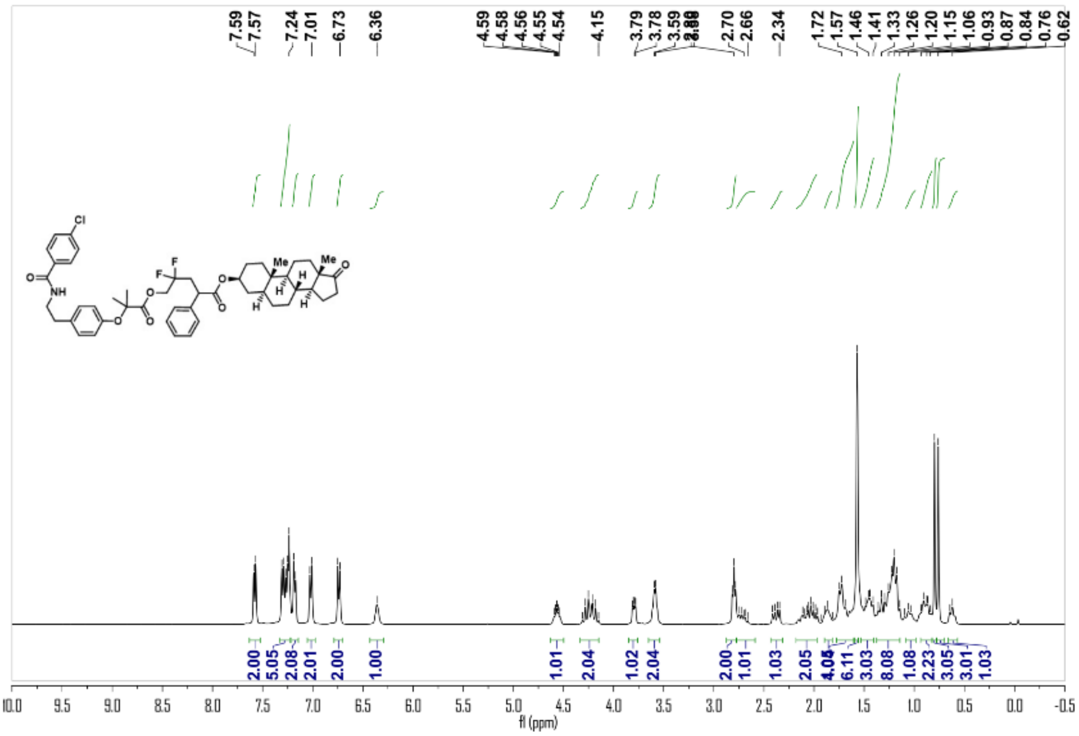

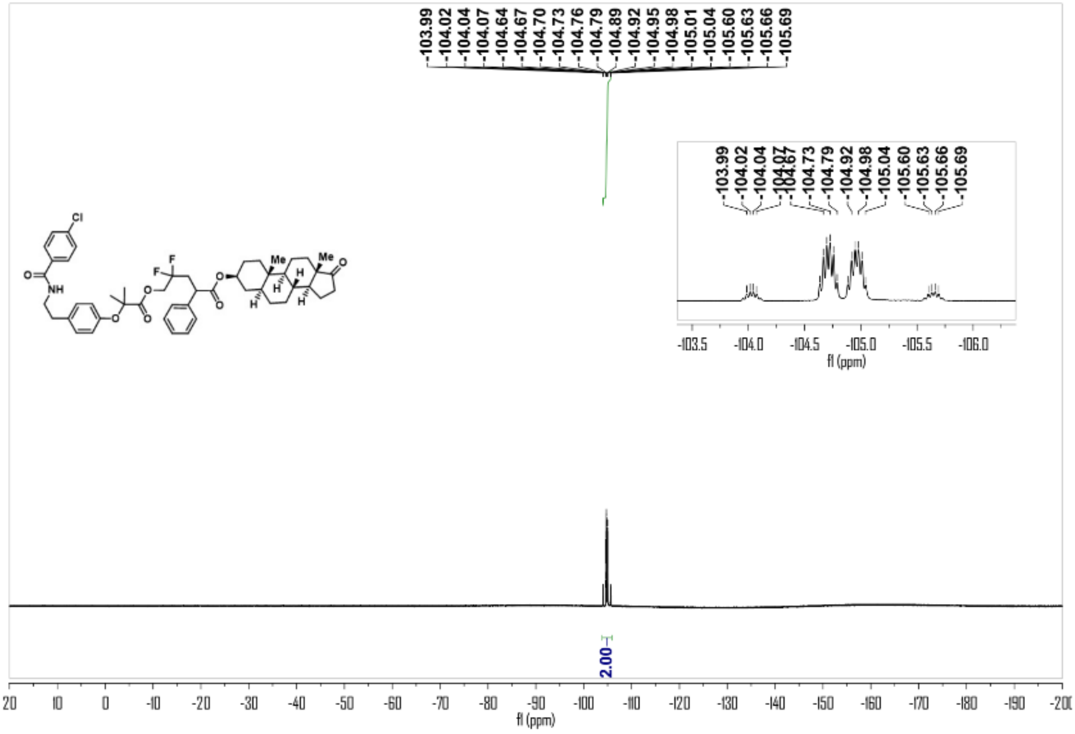

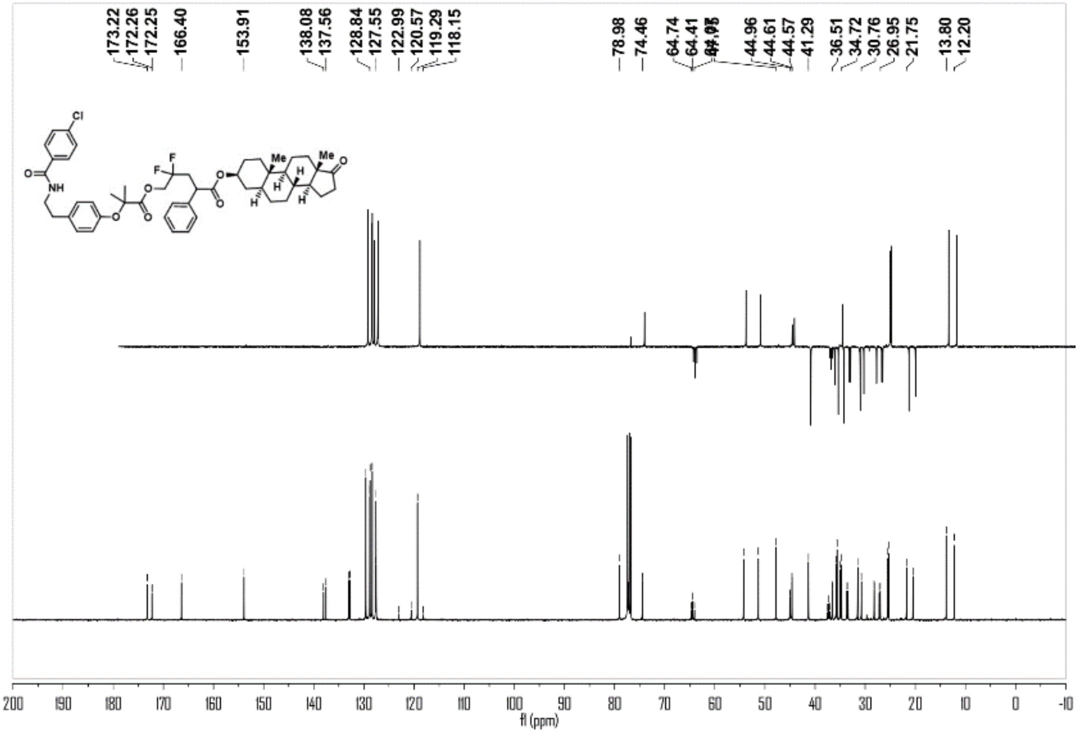

^{1}H, ^{19}F and ^{13}C NMR spectra of compound 3bh-1

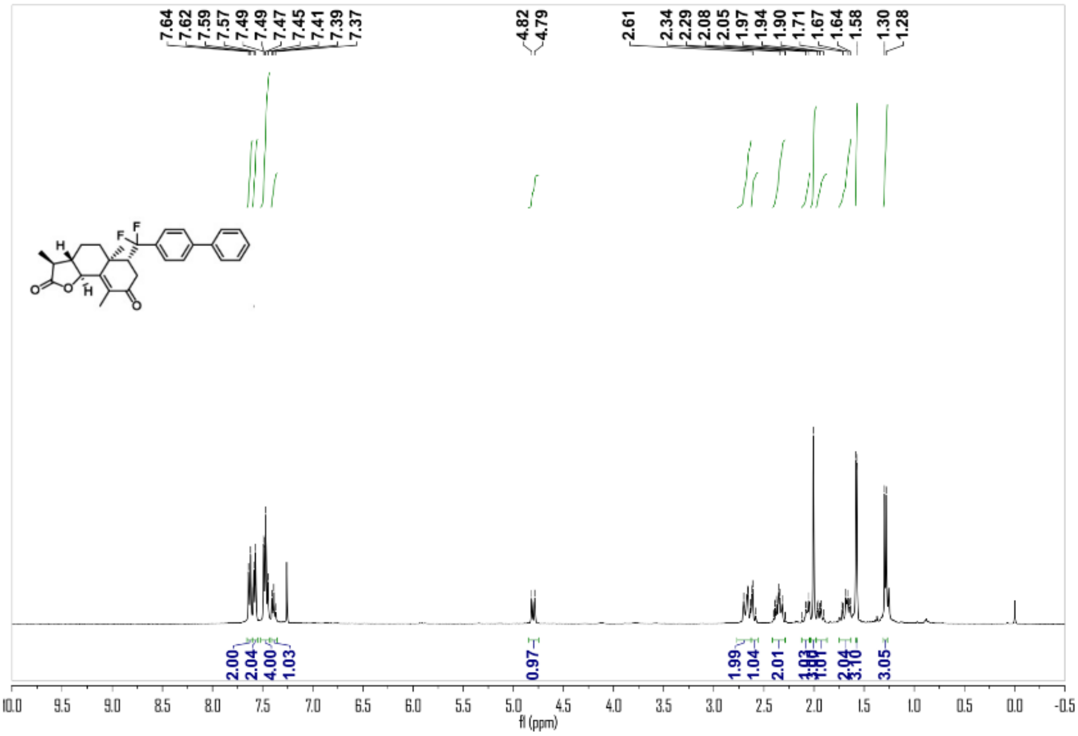

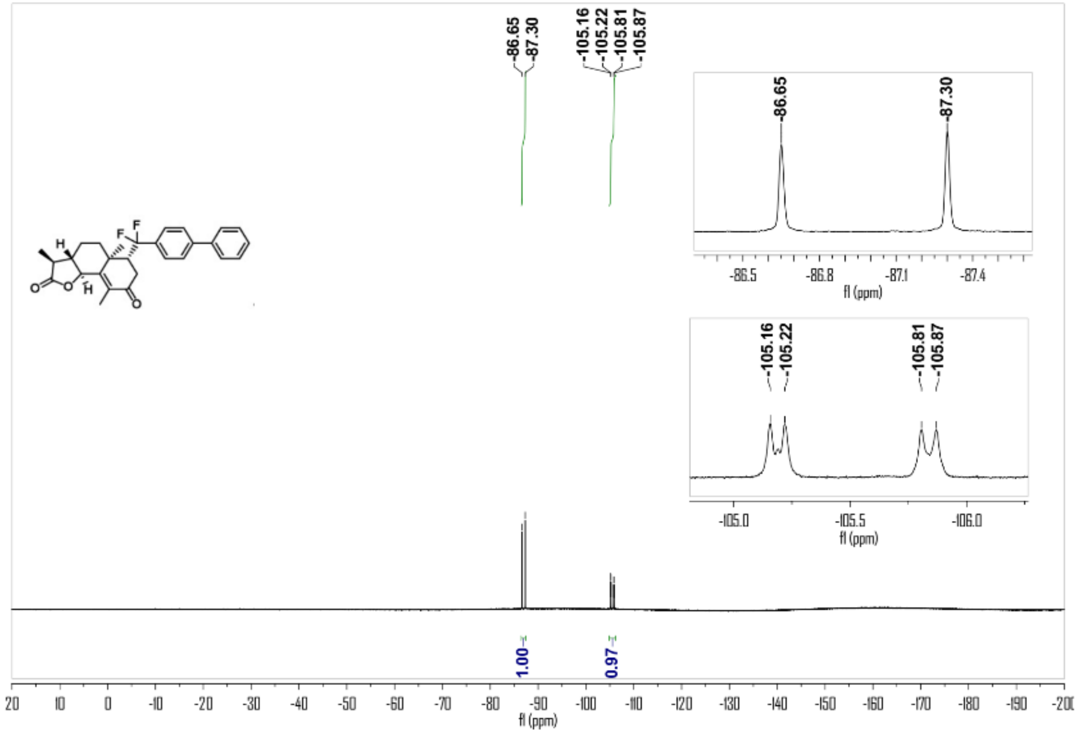

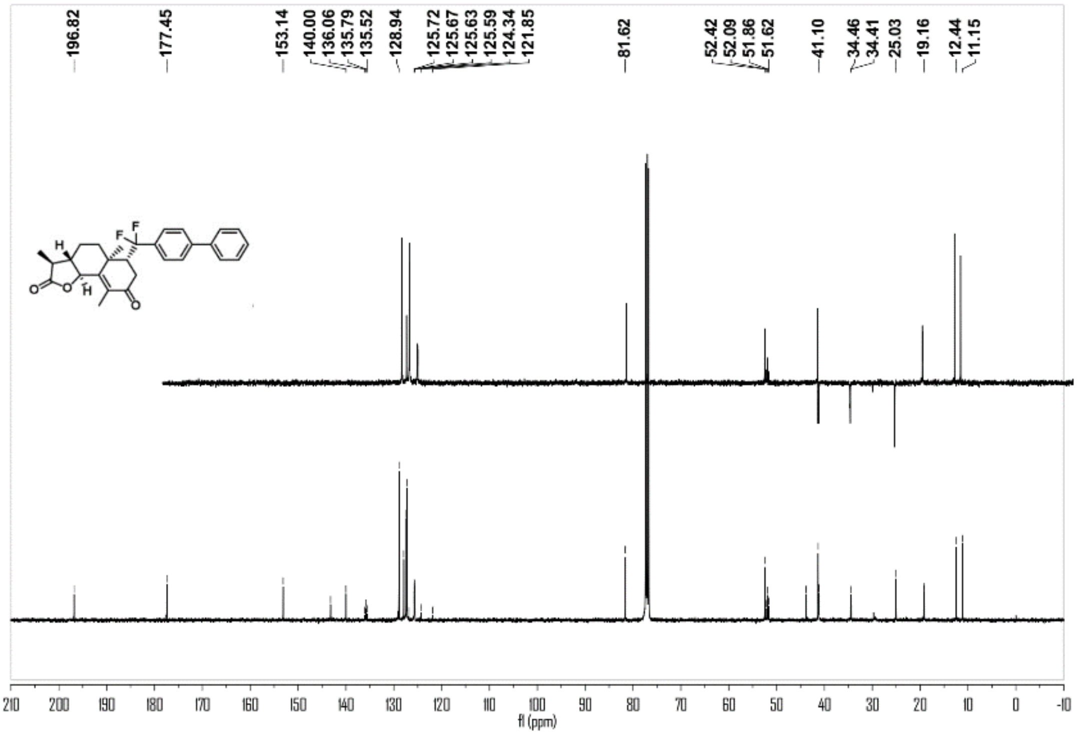

^{1}H, ^{19}F and ^{13}C NMR spectra of compound 3bh-2

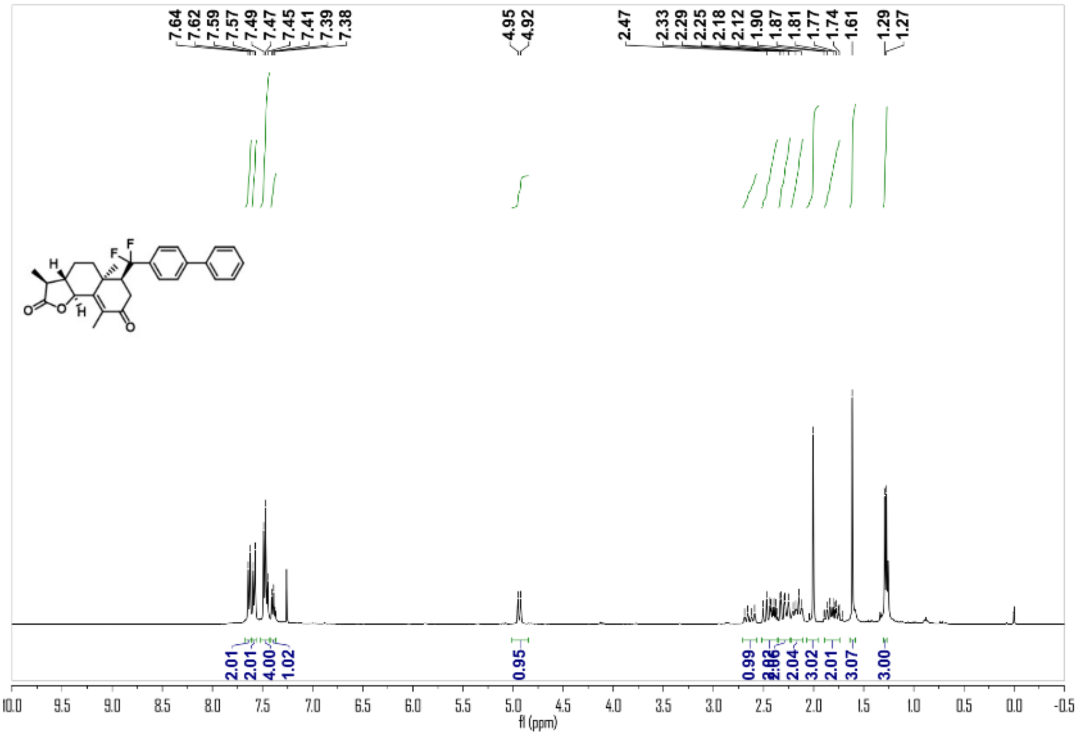

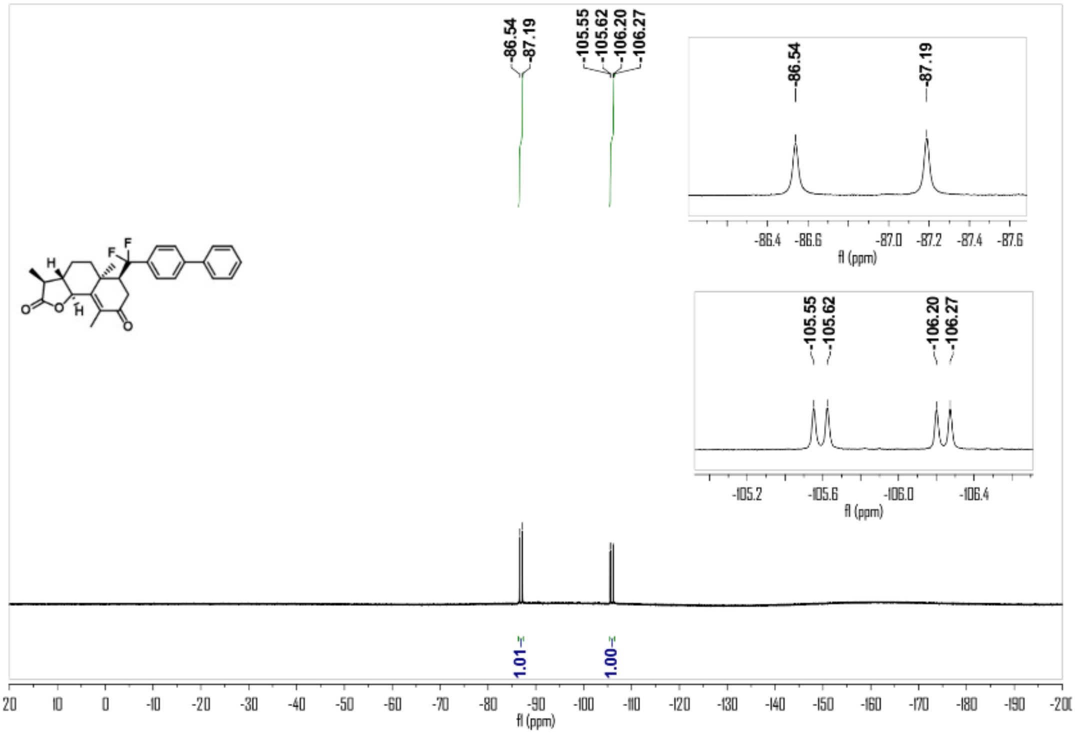
-86.54
-87.19
-105.55
-105.62
-106.20
-106.27
1.01
1.00
fl (ppm)

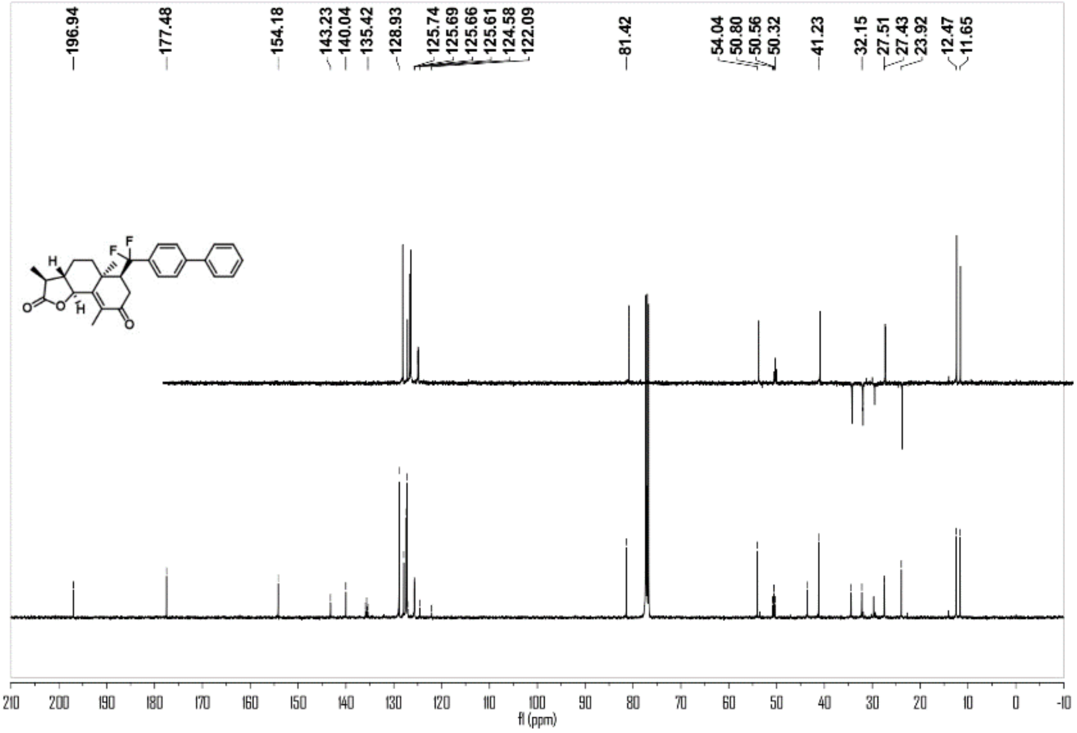
196.94
177.48
154.18
143.23
140.04
135.42
128.93
125.74
125.69
125.66
125.61
124.58
122.09
81.42
54.04
50.80
50.56
50.32
41.23
32.15
27.51
27.43
23.92
12.47
11.65
fl (ppm)

^{1}H, ^{19}F and ^{13}C NMR spectra of compound 3bi

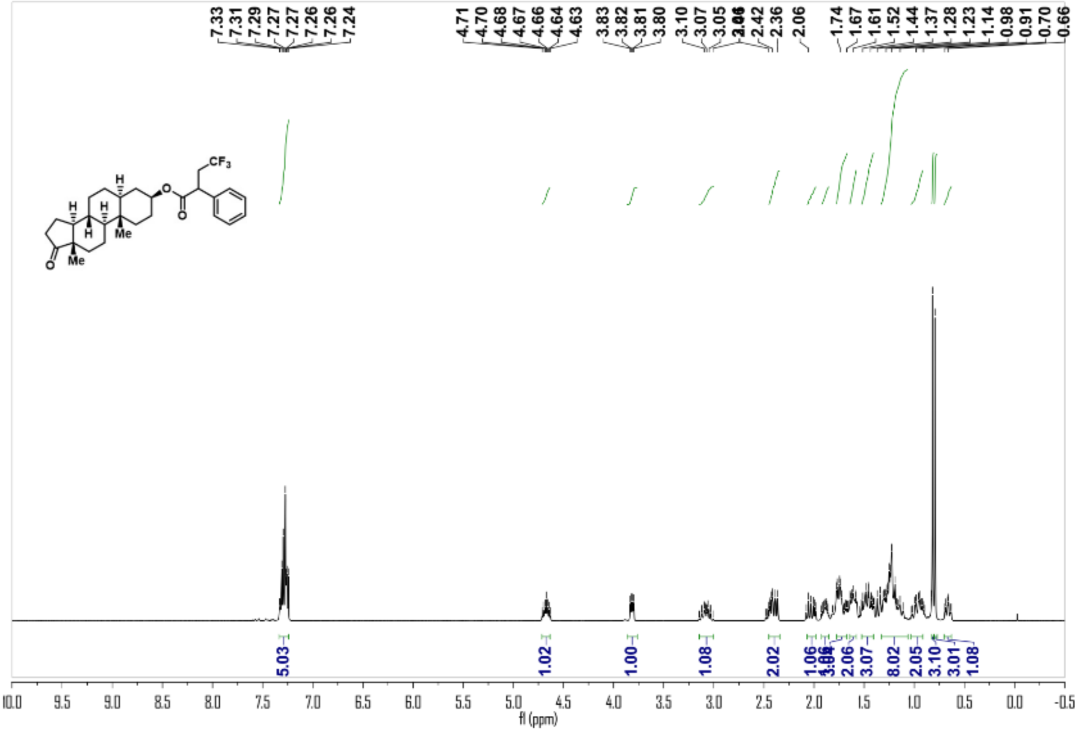

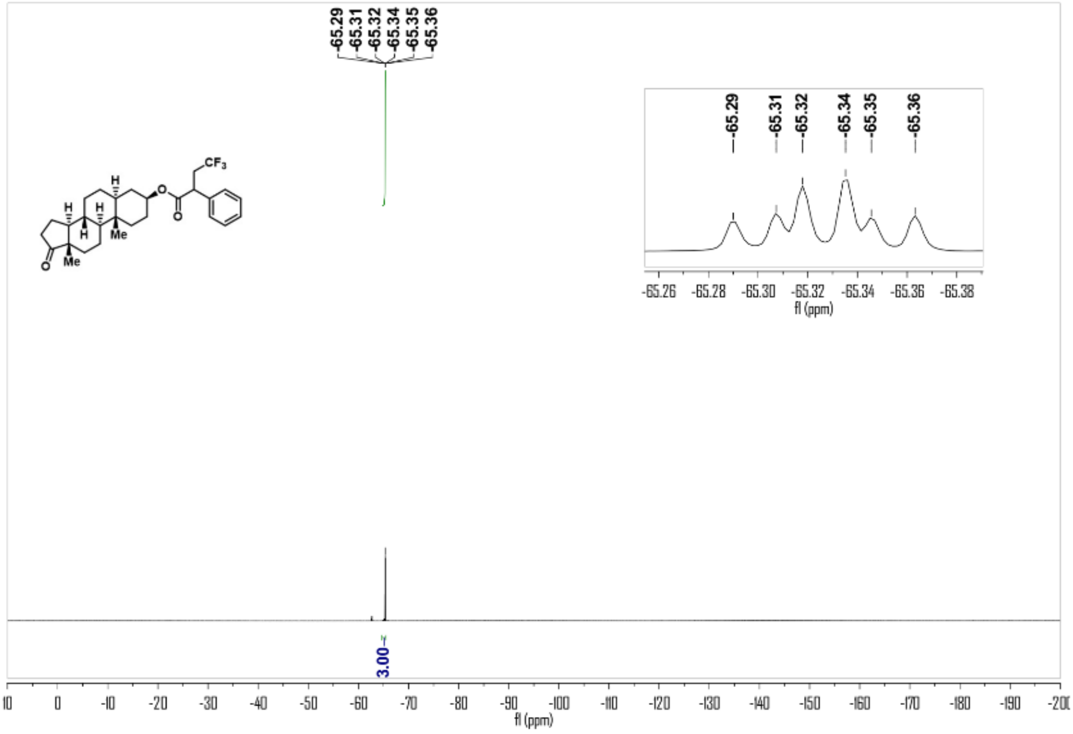

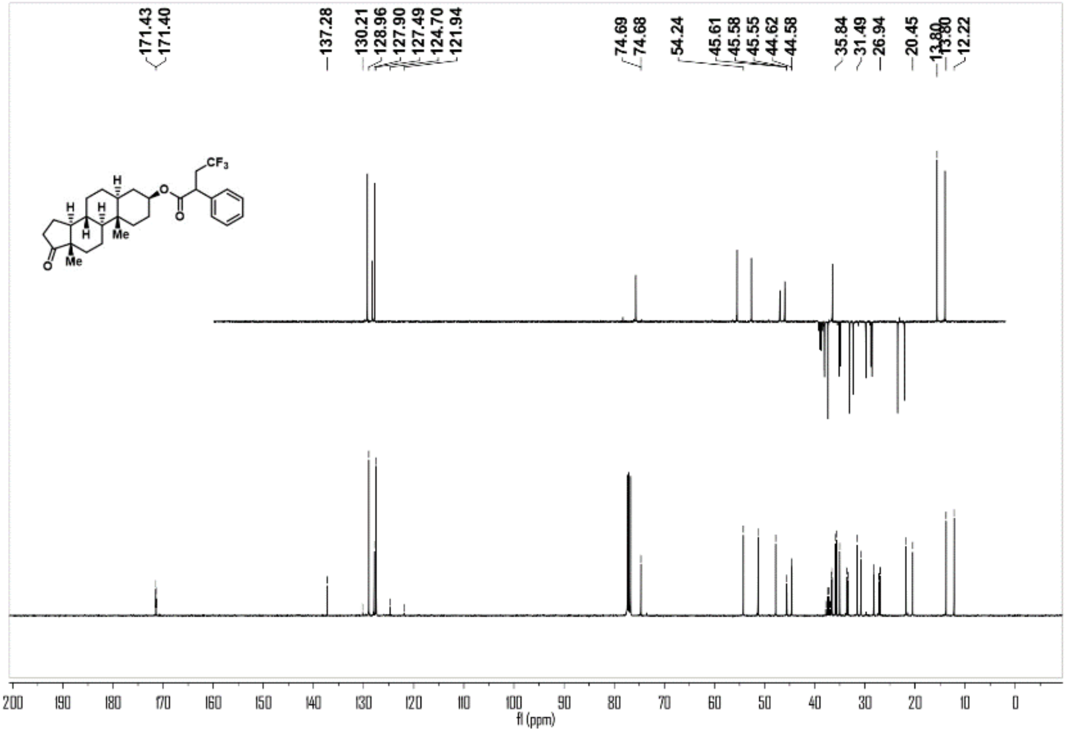

^{1}H, ^{19}F and ^{13}C NMR spectra of compound 3bj

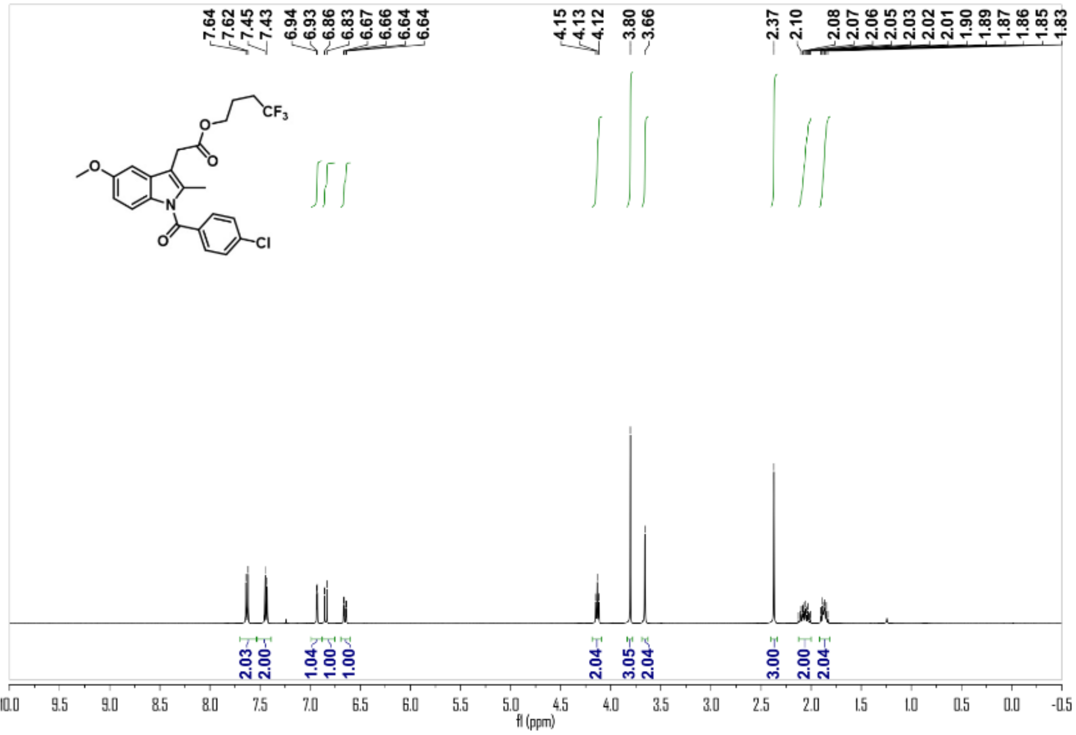

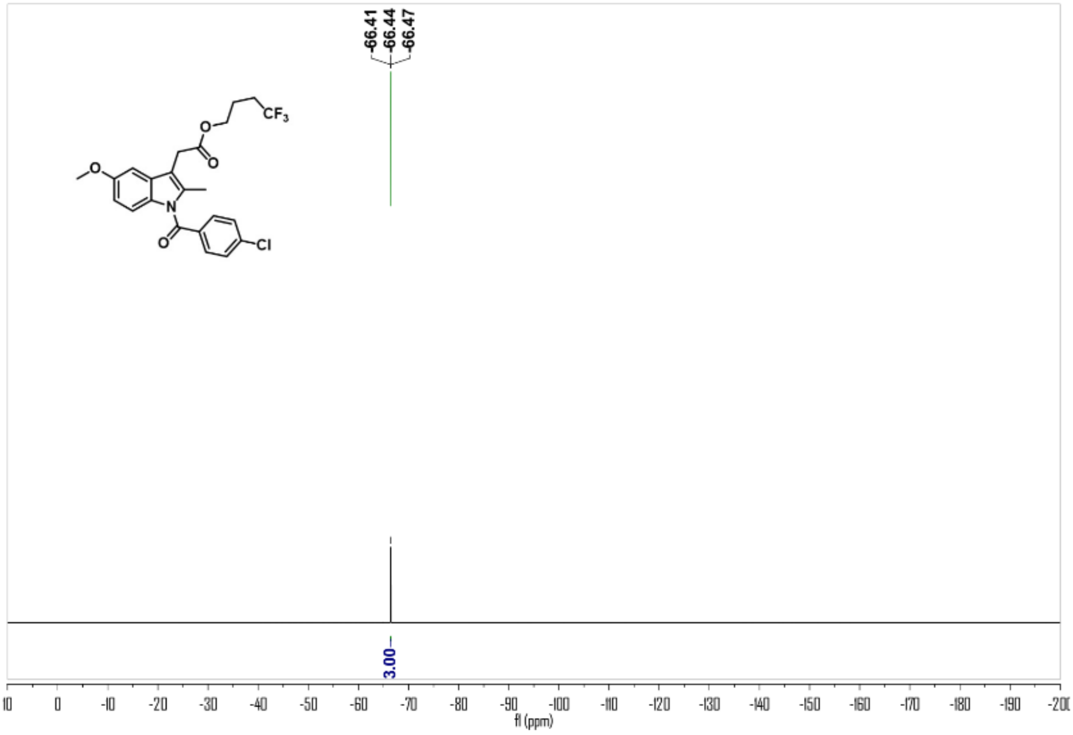
-66.41
-66.44
-66.47
CF3
O
O
O
N
O
Cl
3.00
fl (ppm)

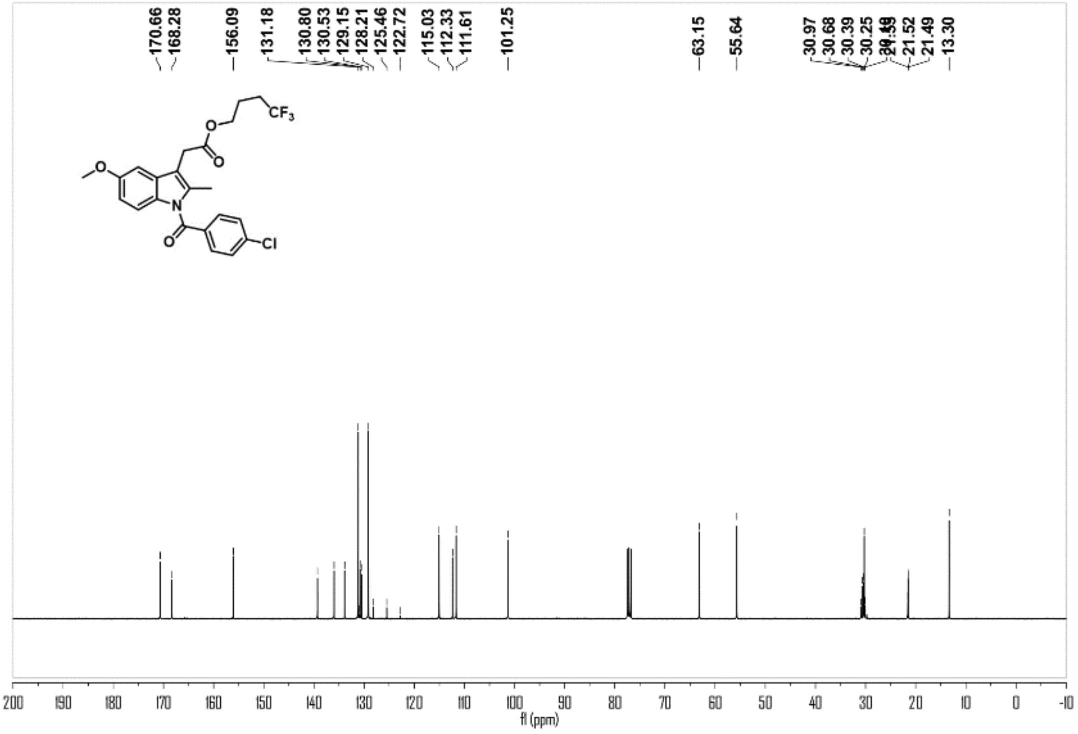
170.66
168.28
156.09
131.18
130.80
130.53
129.15
128.21
125.46
122.72
115.03
112.33
111.61
101.25
63.15
55.64
30.97
30.68
30.39
30.25
21.52
21.49
13.30
CF3
O
O
O
N
O
Cl
fl (ppm)

^{1}H, ^{19}F and ^{13}C NMR spectra of compound 3bk

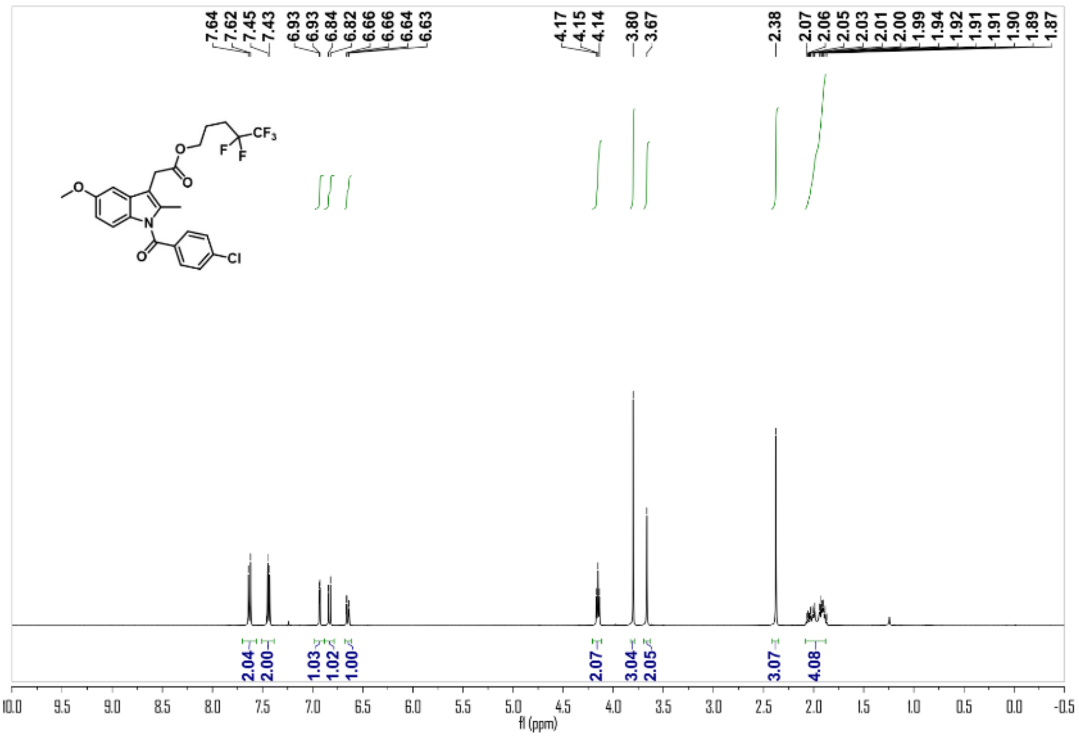

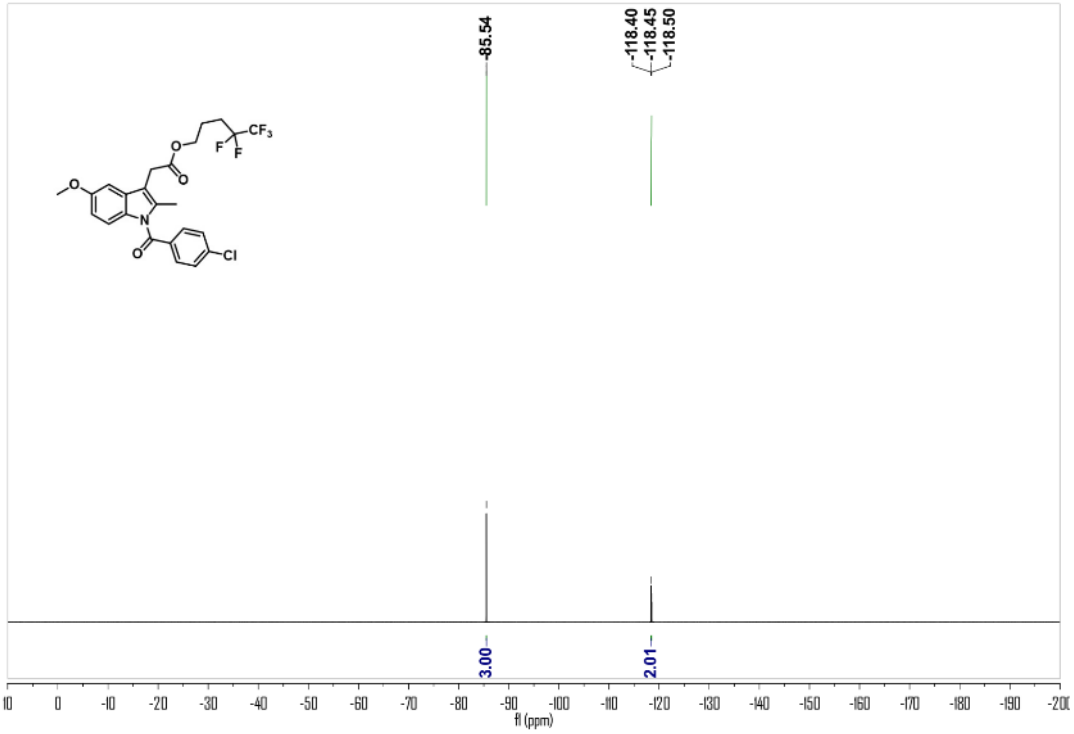

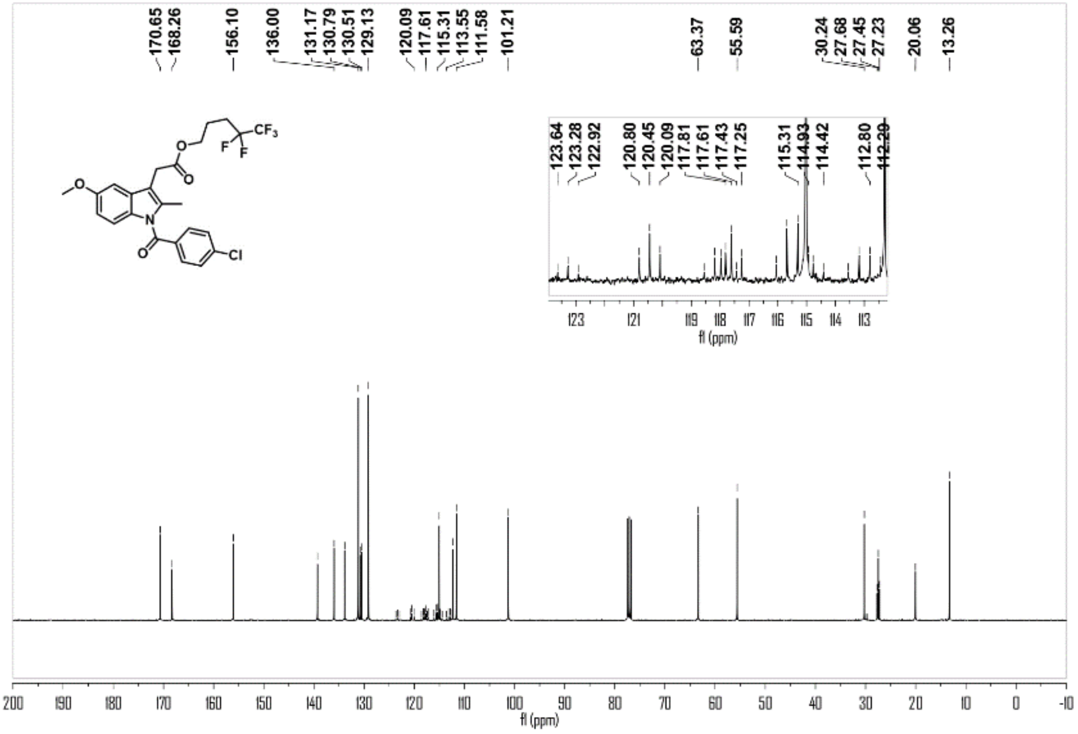

^{1}H, ^{19}F and ^{13}C NMR spectra of compound 3bl

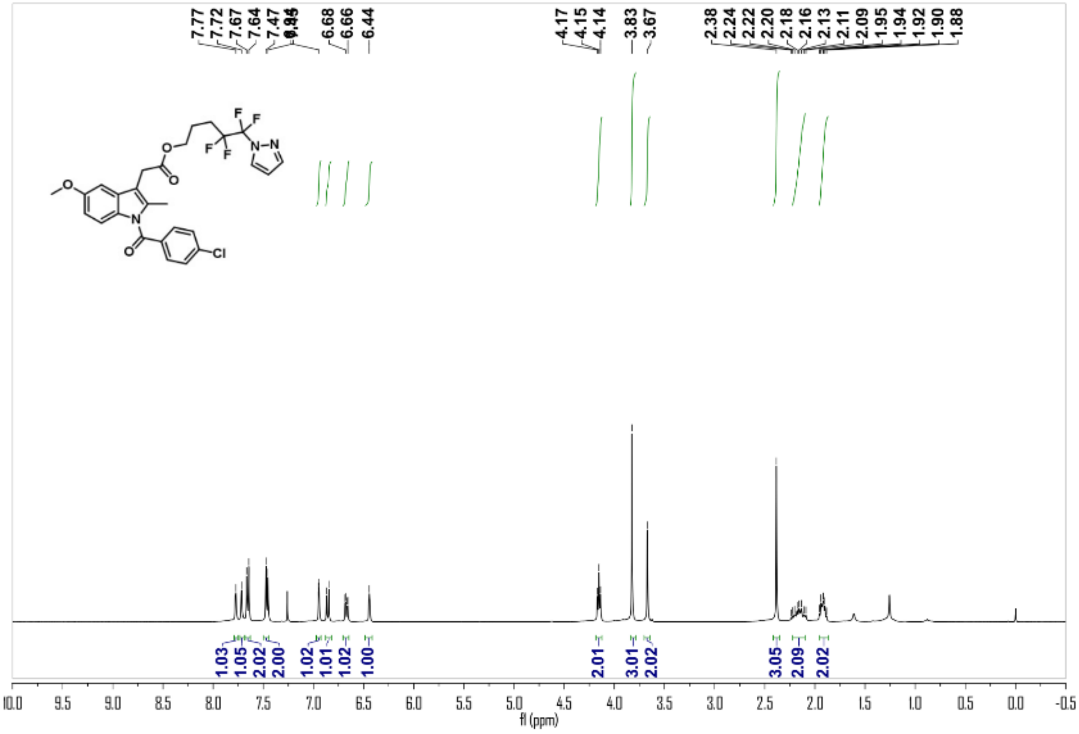

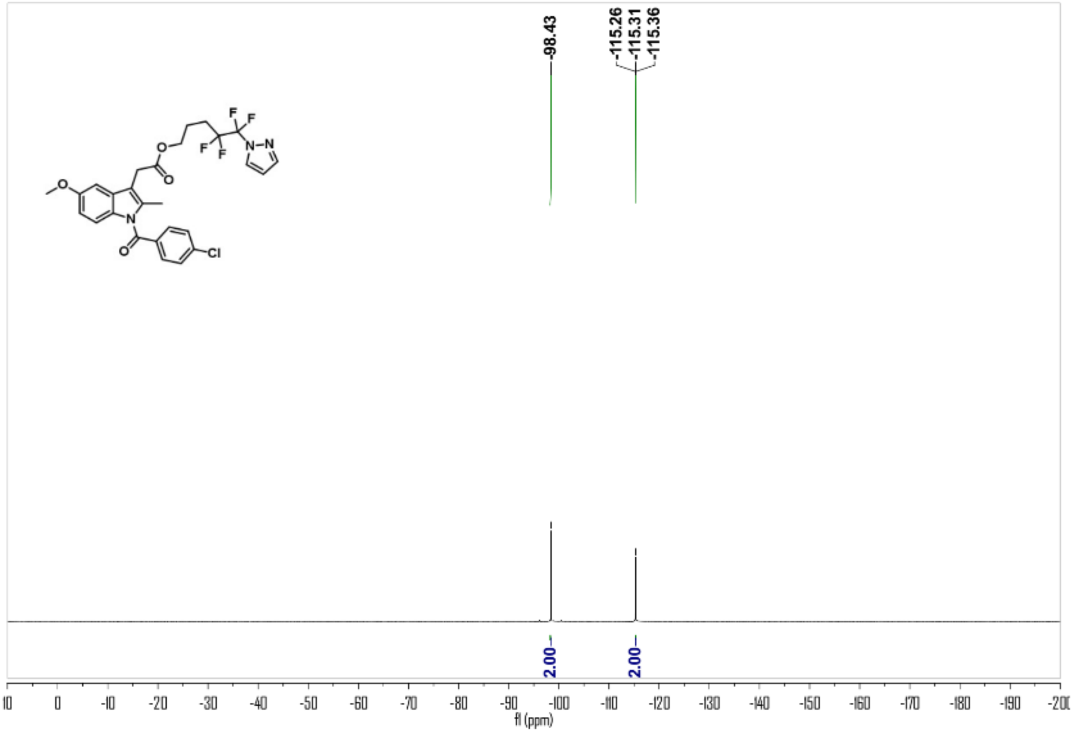

-98.43
-115.26
-115.31
-115.36
2.00
2.00
fl (ppm)

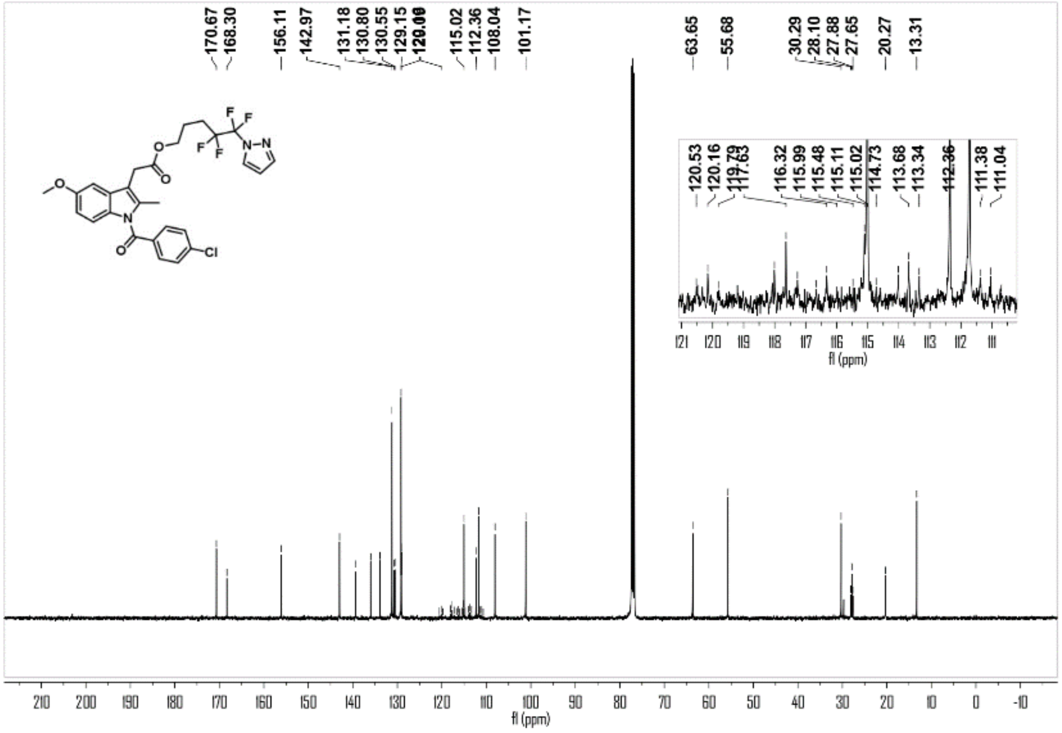

170.67
168.30
156.11
142.97
131.18
130.80
130.55
129.15
115.02
112.36
108.04
101.17
63.65
55.68
30.29
28.10
27.88
27.65
20.27
13.31
120.53
120.16
116.32
115.99
115.48
115.11
115.02
114.73
113.68
113.34
112.36
111.38
111.04
fl (ppm)

^{1}H, ^{19}F and ^{13}C NMR spectra of compound 5a

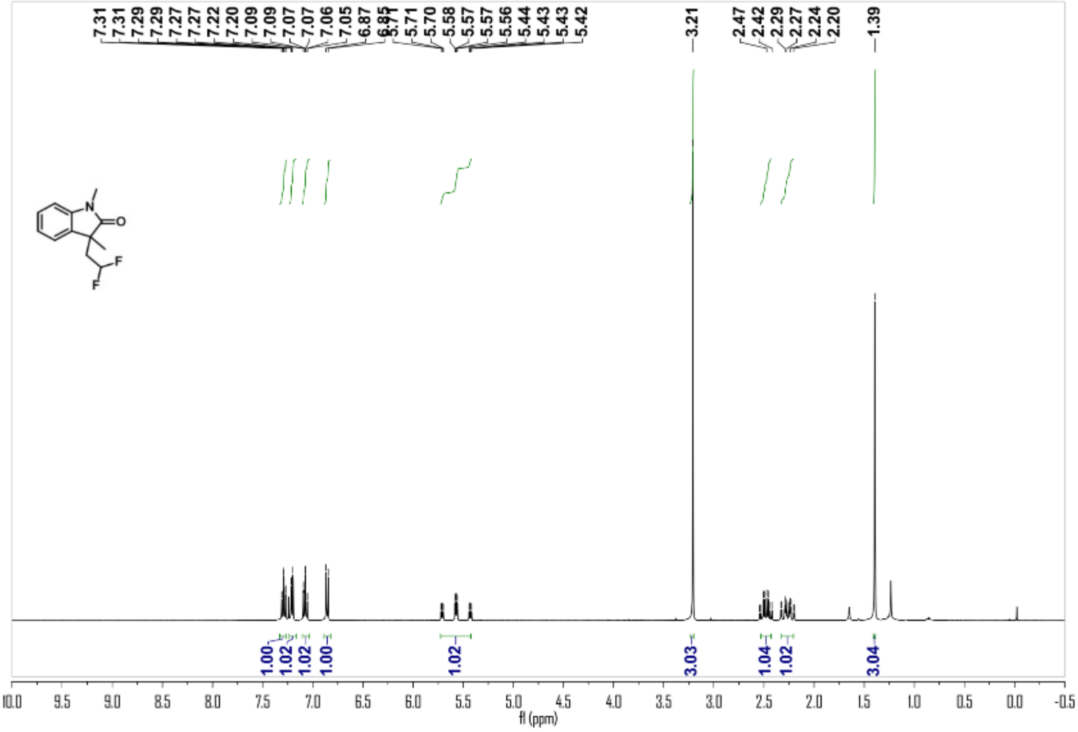

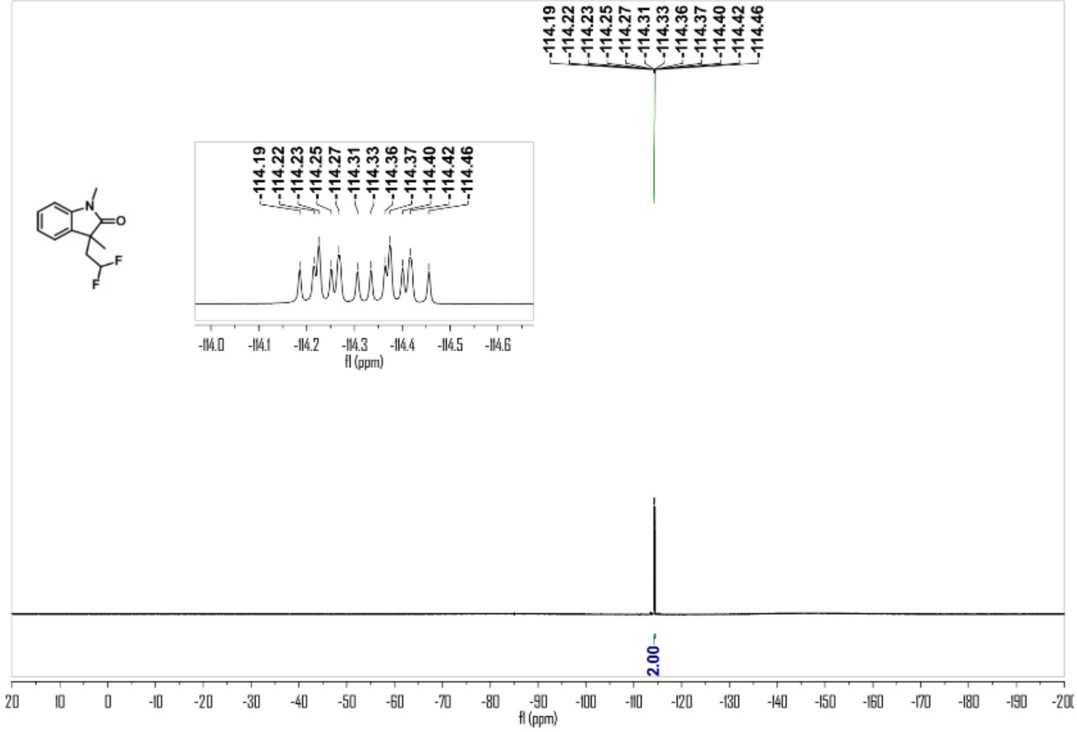

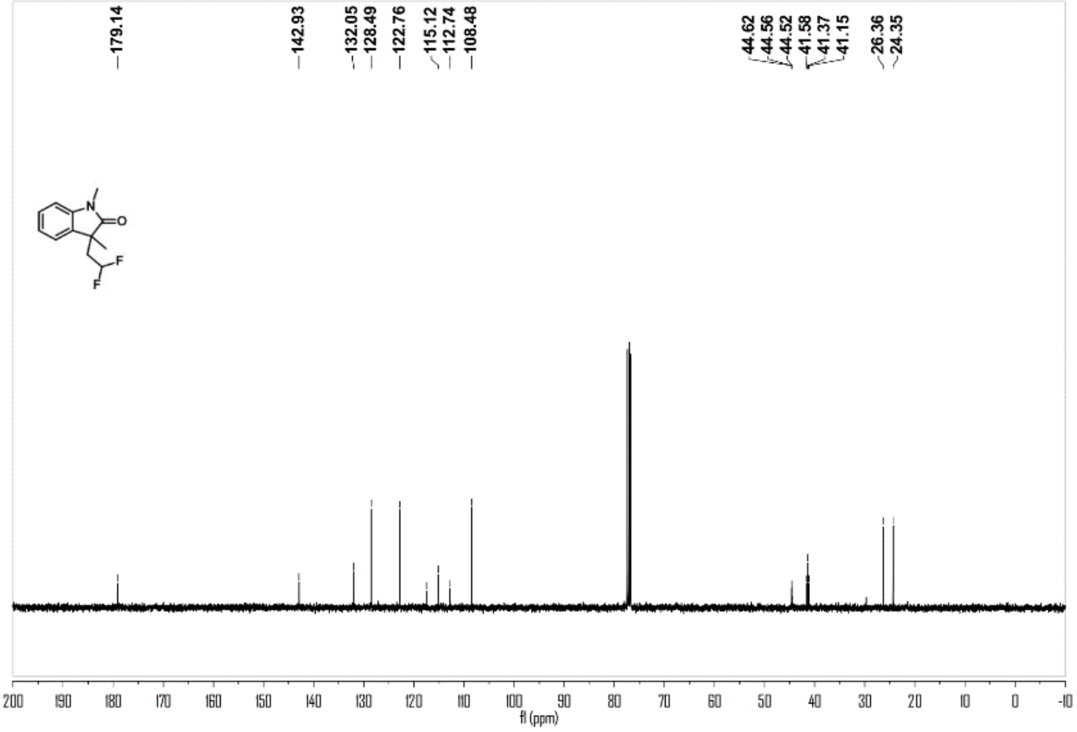

^1H, ^{19}F and ^{13}C NMR spectra of compound 5b

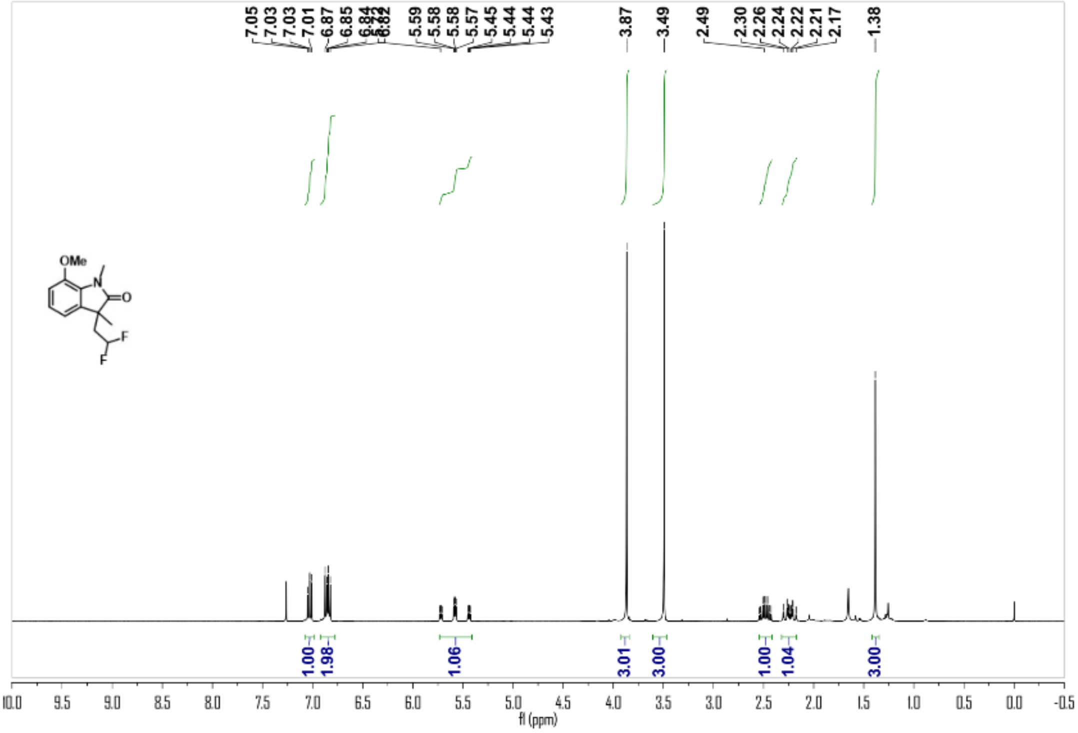

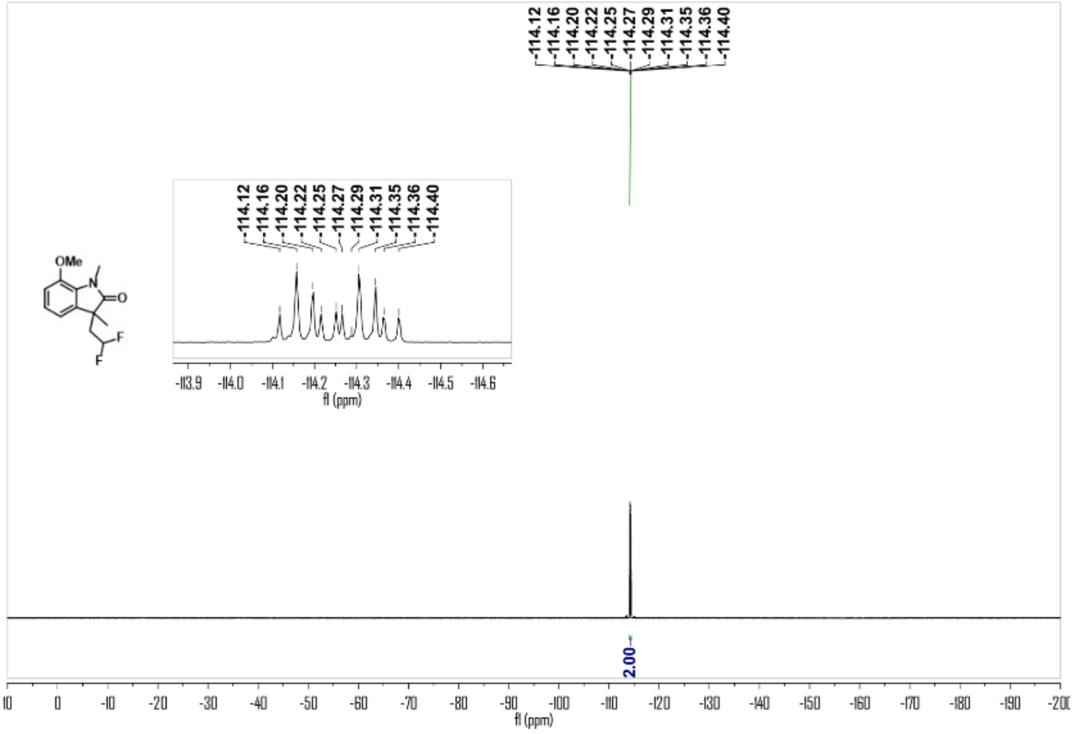
-114.12
-114.16
-114.20
-114.22
-114.25
-114.27
-114.29
-114.31
-114.35
-114.36
-114.40
OMe
N
O
F
F
-113.9 -114.0 -114.1 -114.2 -114.3 -114.4 -114.5 -114.6
f1 (ppm)
2.00
10 0 -10 -20 -30 -40 -50 -60 -70 -80 -90 -100 -110 -120 -130 -140 -150 -160 -170 -180 -190 -200
f1 (ppm)

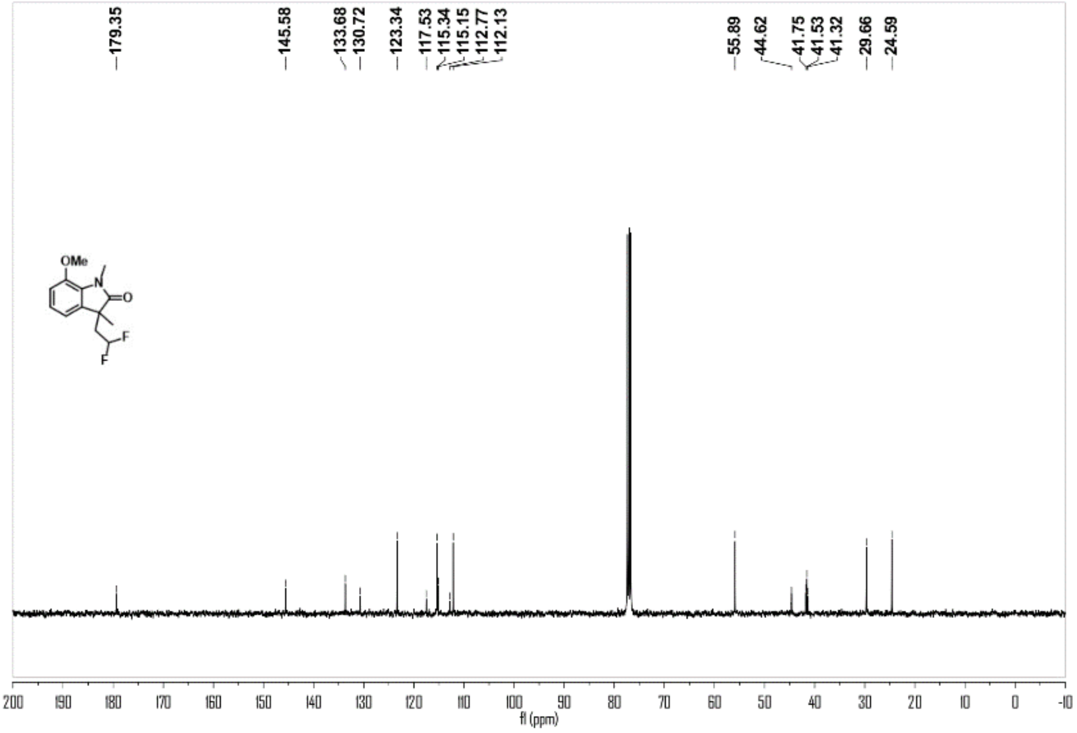
179.35
145.58
133.68
130.72
123.34
117.53
115.34
115.15
112.77
112.13
55.89
44.62
41.75
41.53
41.32
29.66
24.59
OMe
N
O
F
F
200 190 180 170 160 150 140 130 120 110 100 90 80 70 60 50 40 30 20 10 0 -10
f1 (ppm)

^{1}H, ^{19}F and ^{13}C NMR spectra of compound 5c

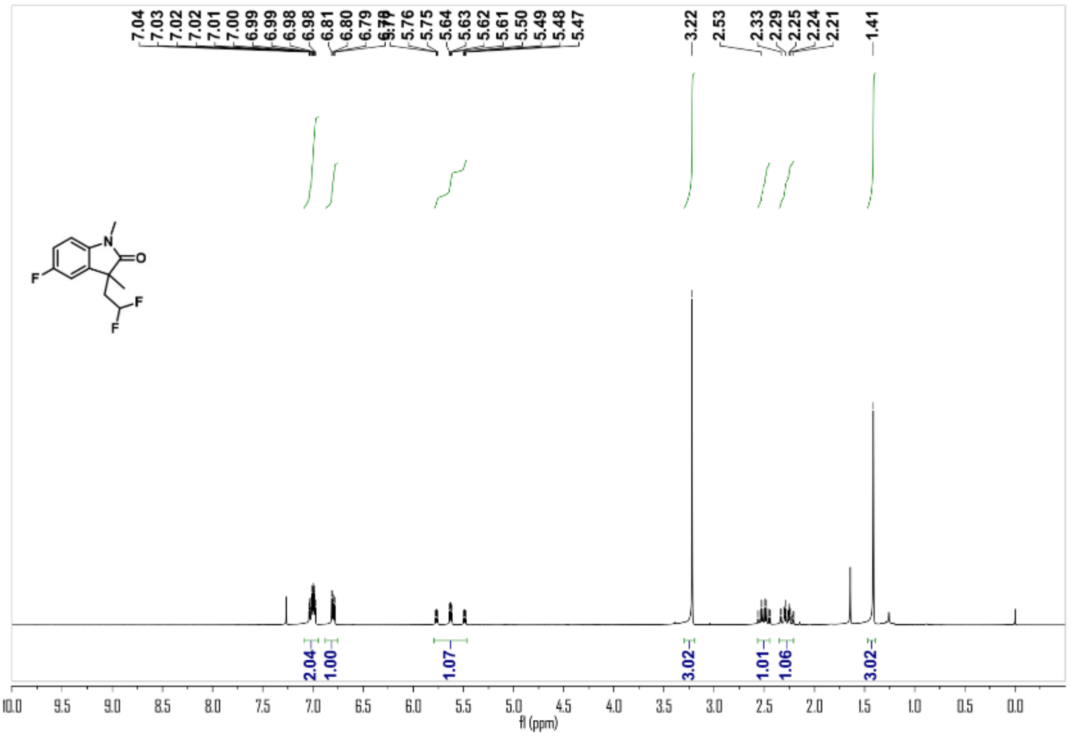

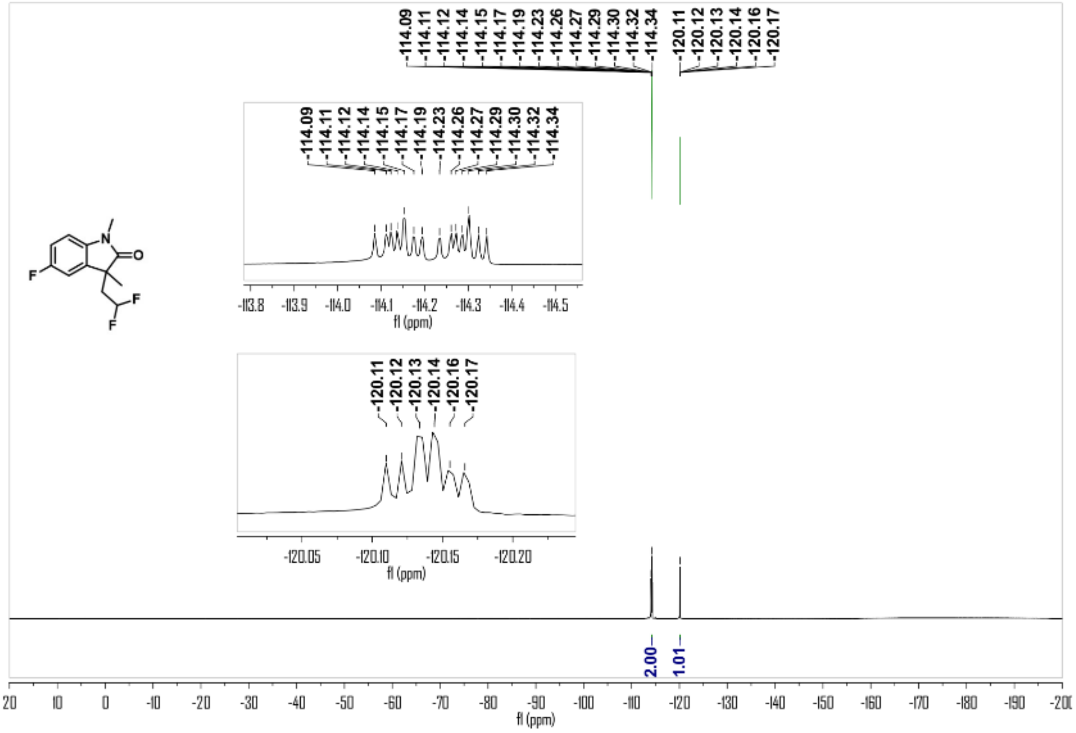

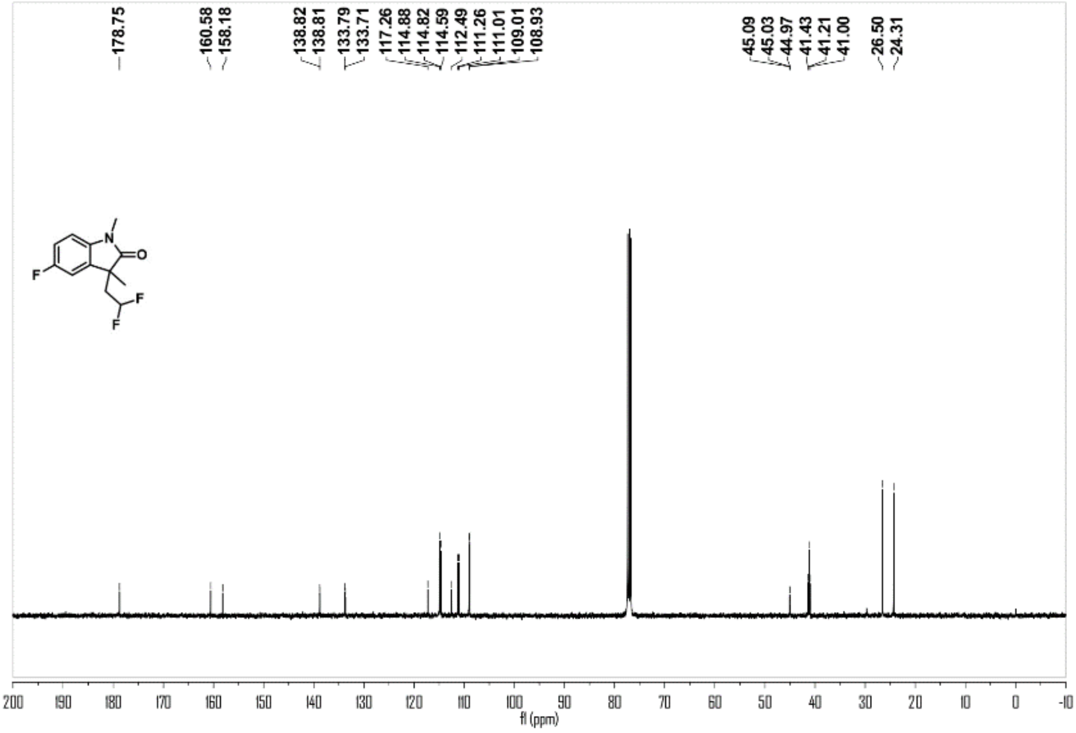

^{1}H, ^{19}F and ^{13}C NMR spectra of compound 5d

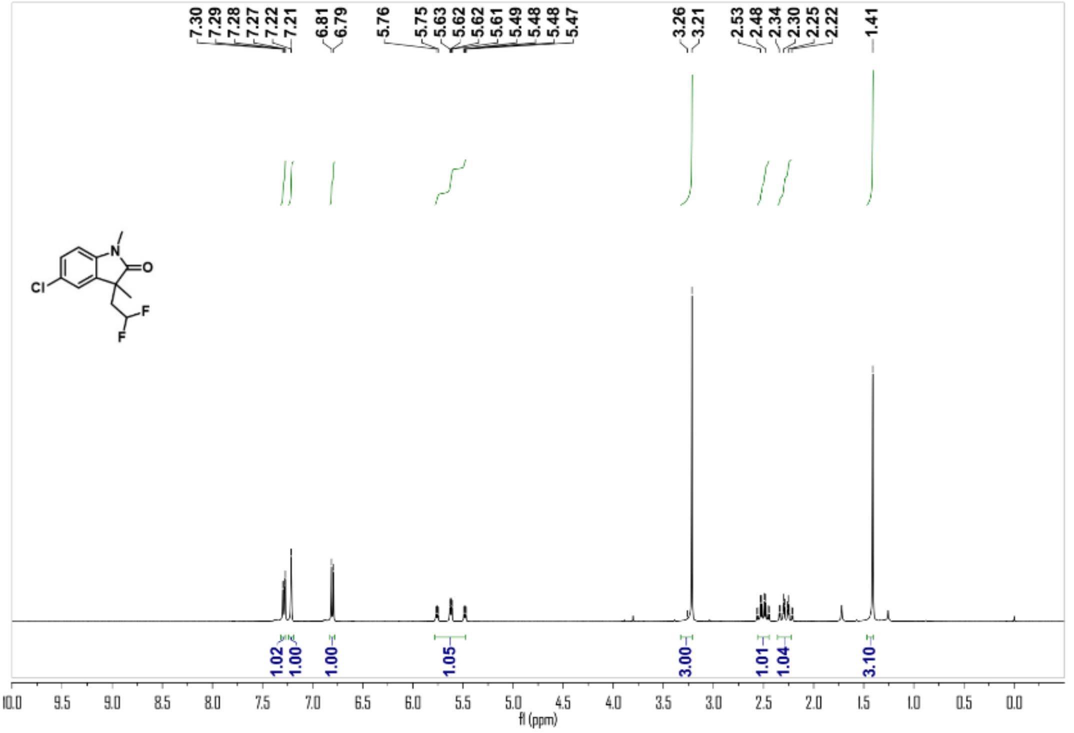

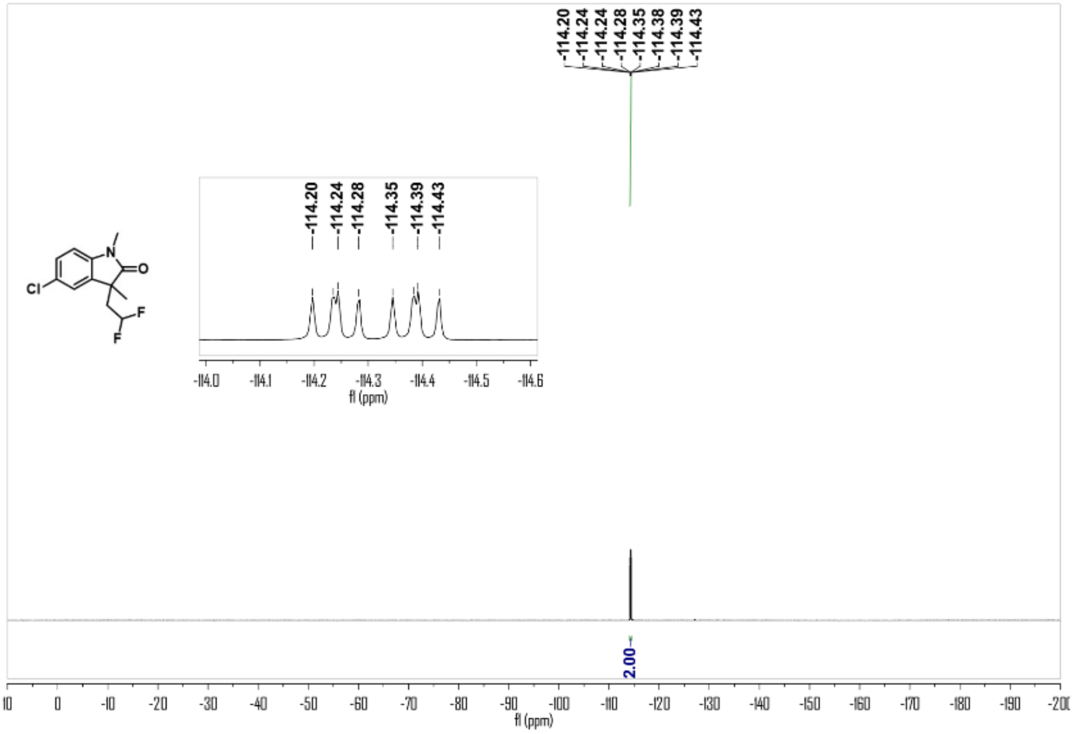

-114.20
-114.24
-114.24
-114.28
-114.35
-114.38
-114.39
-114.43
Cl
N
O
F
F
2.00
f1 (ppm)

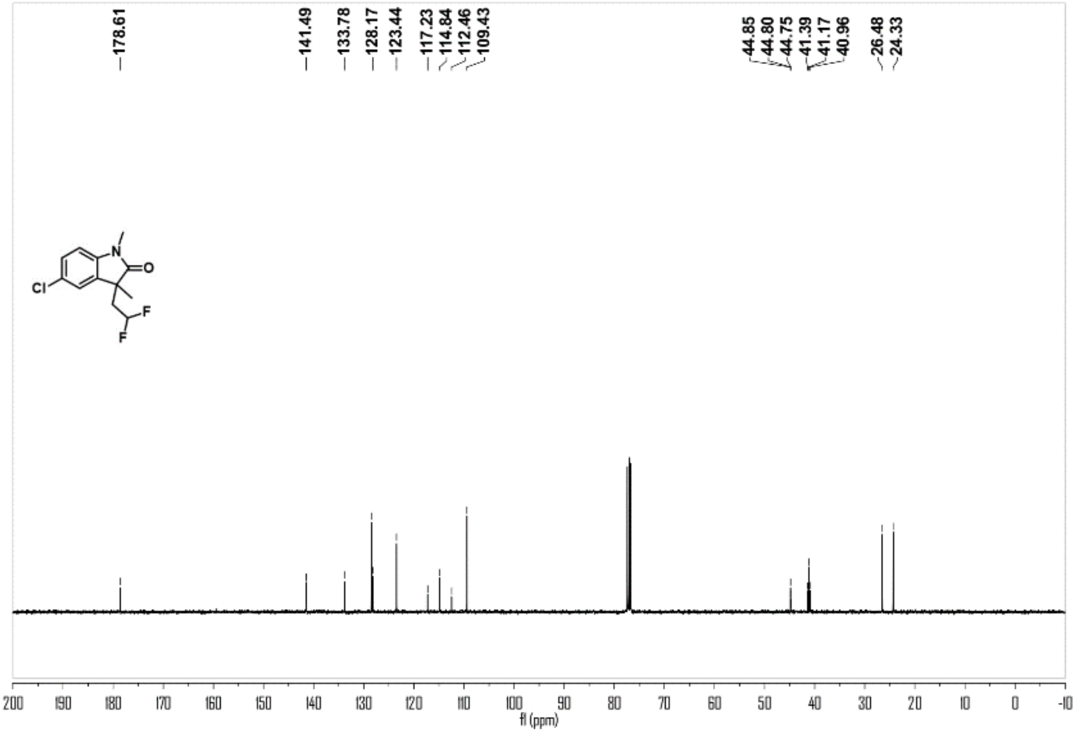

178.61
141.49
133.78
128.17
123.44
117.23
114.84
112.46
109.43
44.85
44.80
44.75
41.39
41.17
40.96
26.48
24.33
Cl
N
O
F
F
f1 (ppm)

^{1}H, ^{19}F and ^{13}C NMR spectra of compound 5e

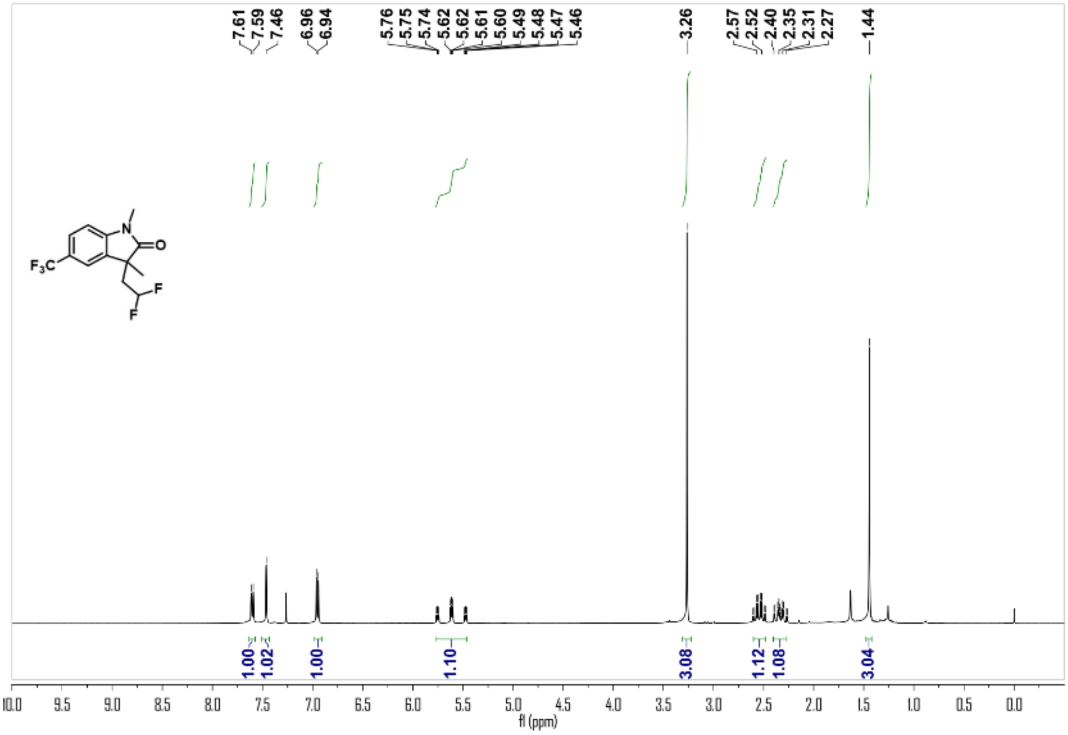

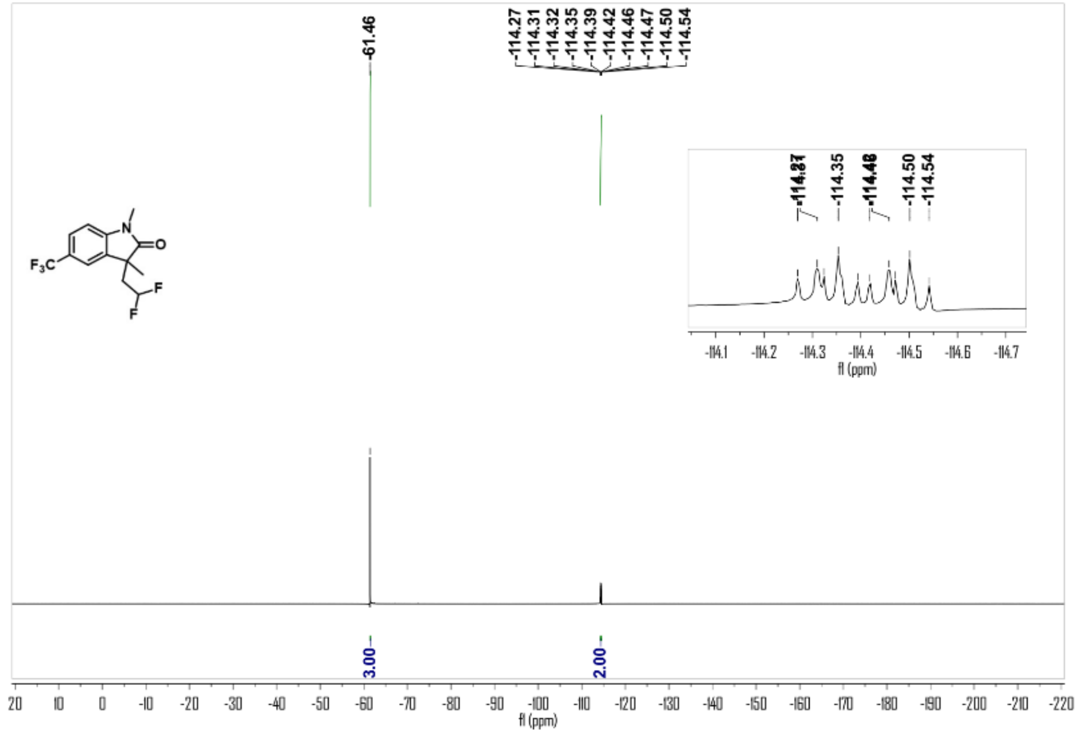

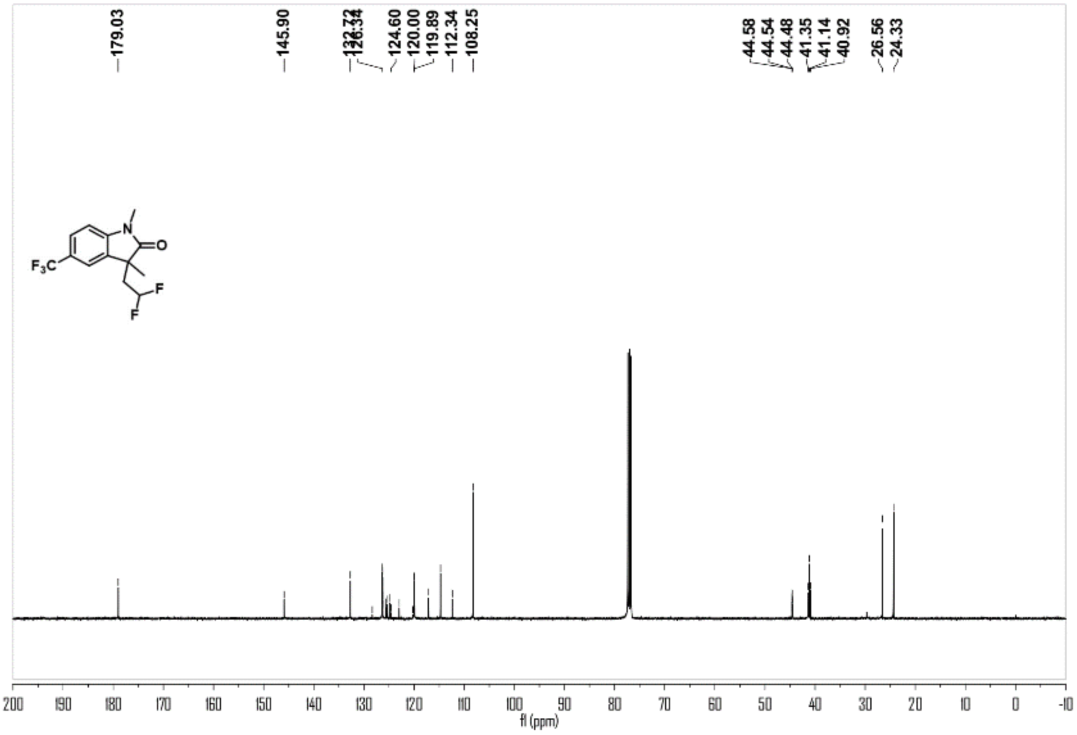

^{1}H, ^{19}F and ^{13}C NMR spectra of compound 5f

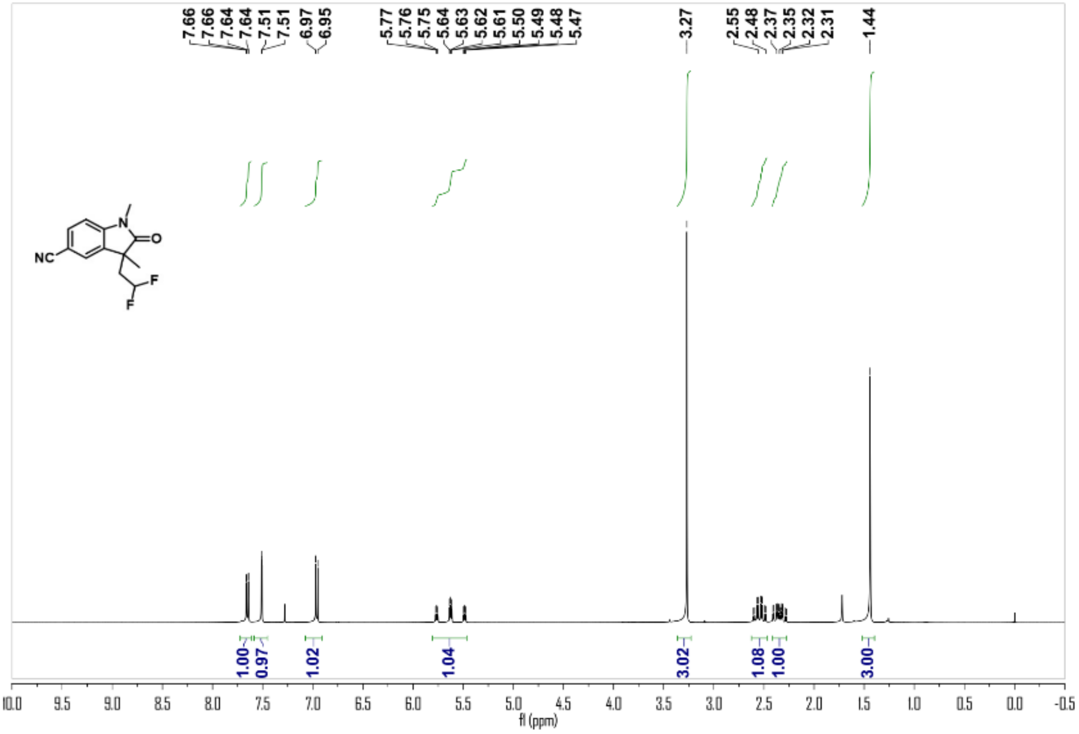

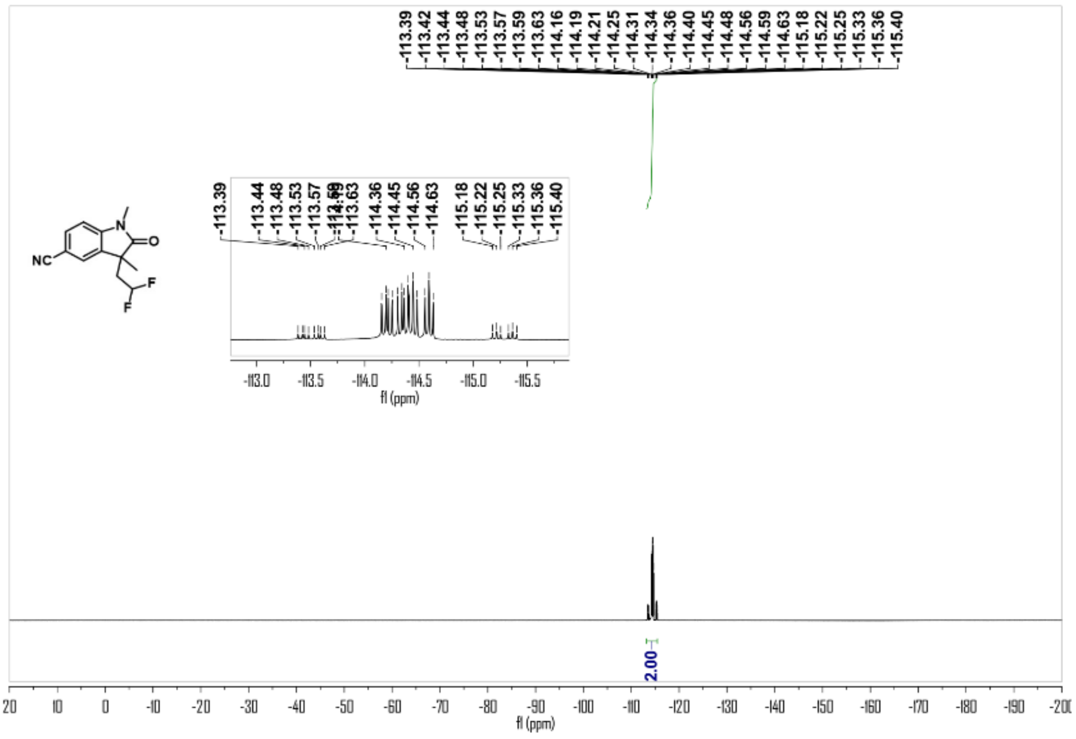

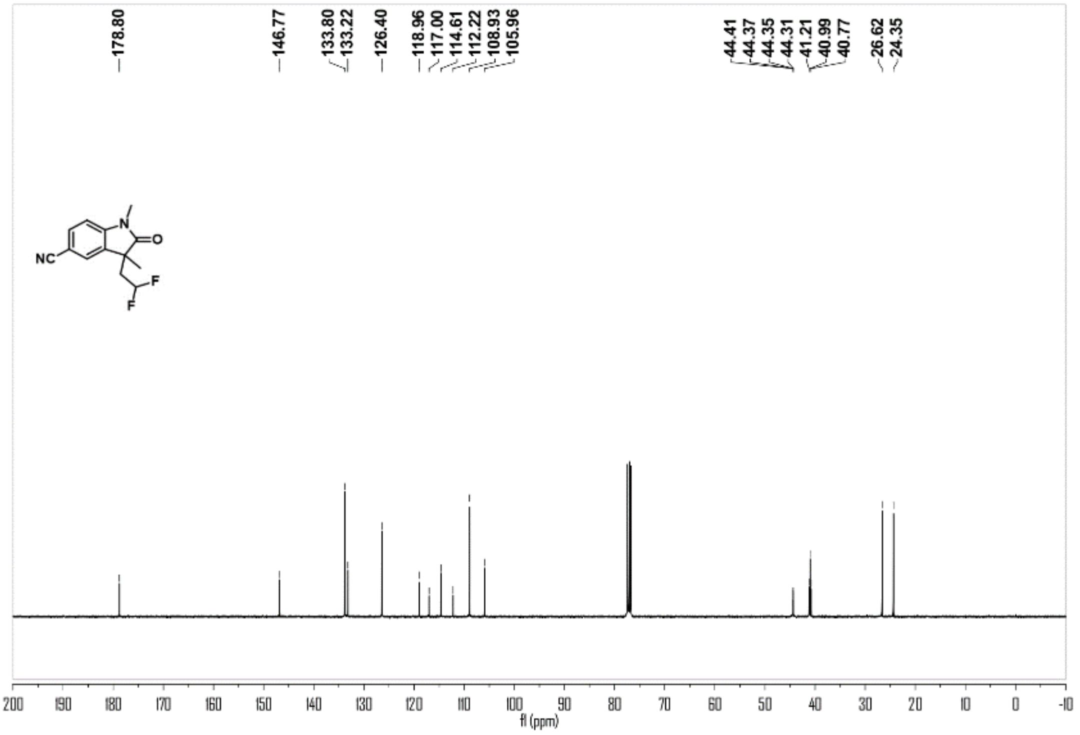

^{1}H, ^{19}F and ^{13}C NMR spectra of compound 5g

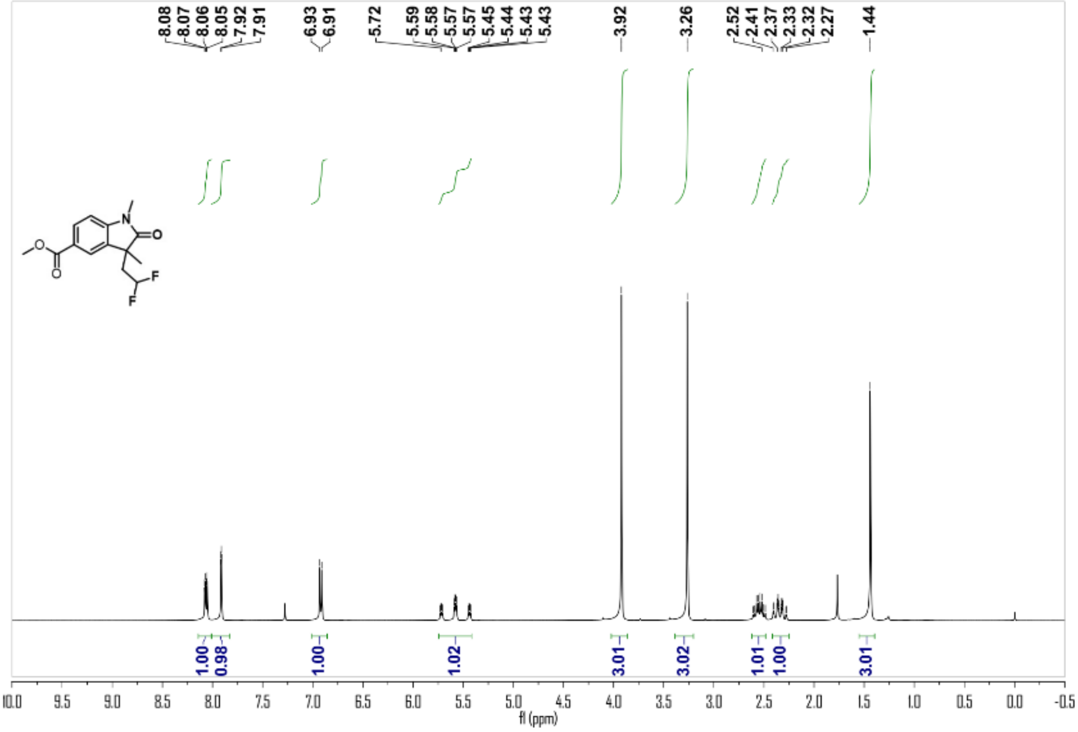

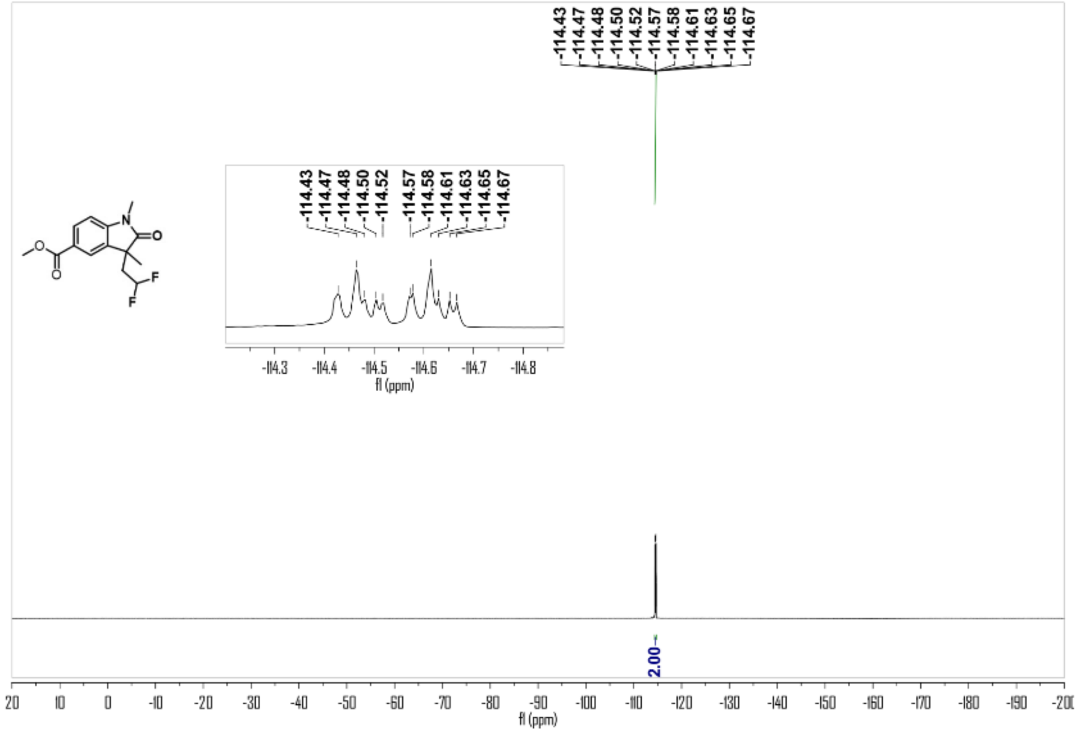

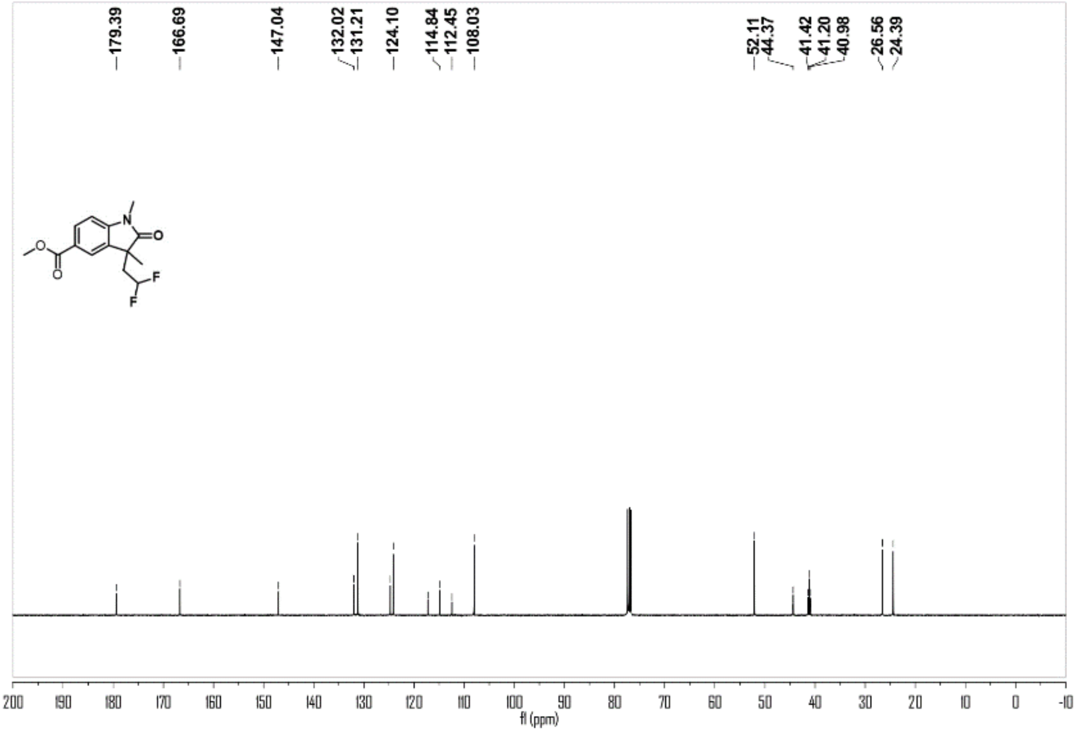

^{1}H, ^{19}F and ^{13}C NMR spectra of compound 5h

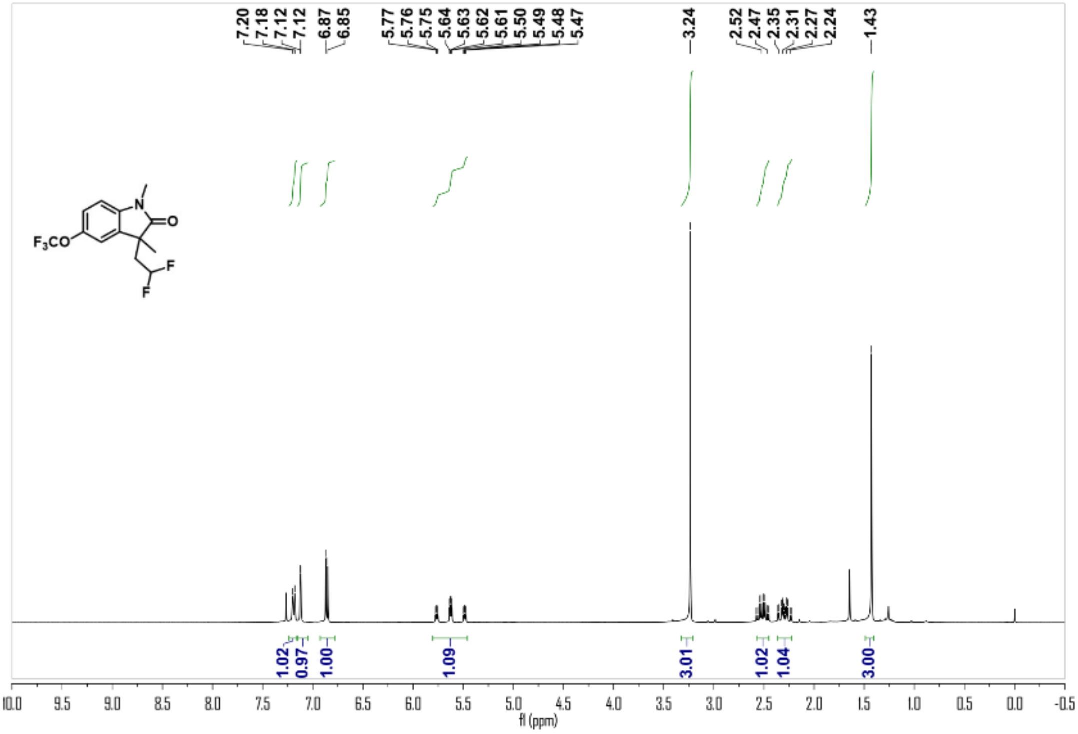

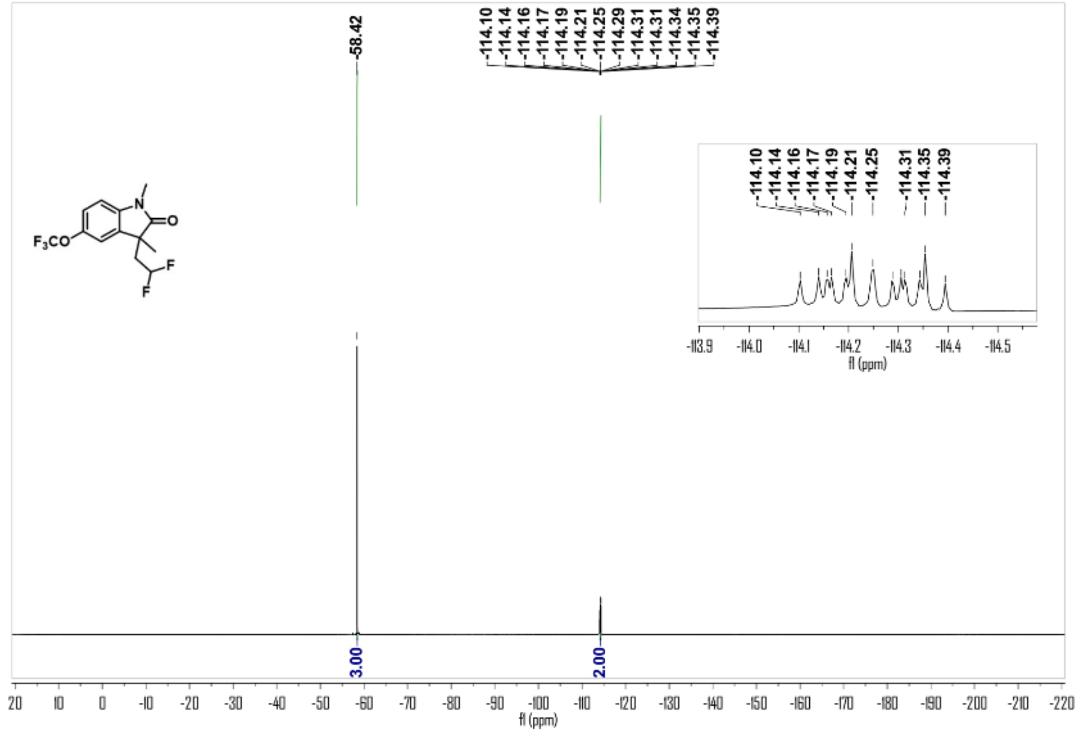
-58.42
-114.10
-114.14
-114.16
-114.17
-114.19
-114.21
-114.25
-114.29
-114.31
-114.31
-114.34
-114.35
-114.39
F3CO
N
O
F
F
3.00
2.00
f1 (ppm)

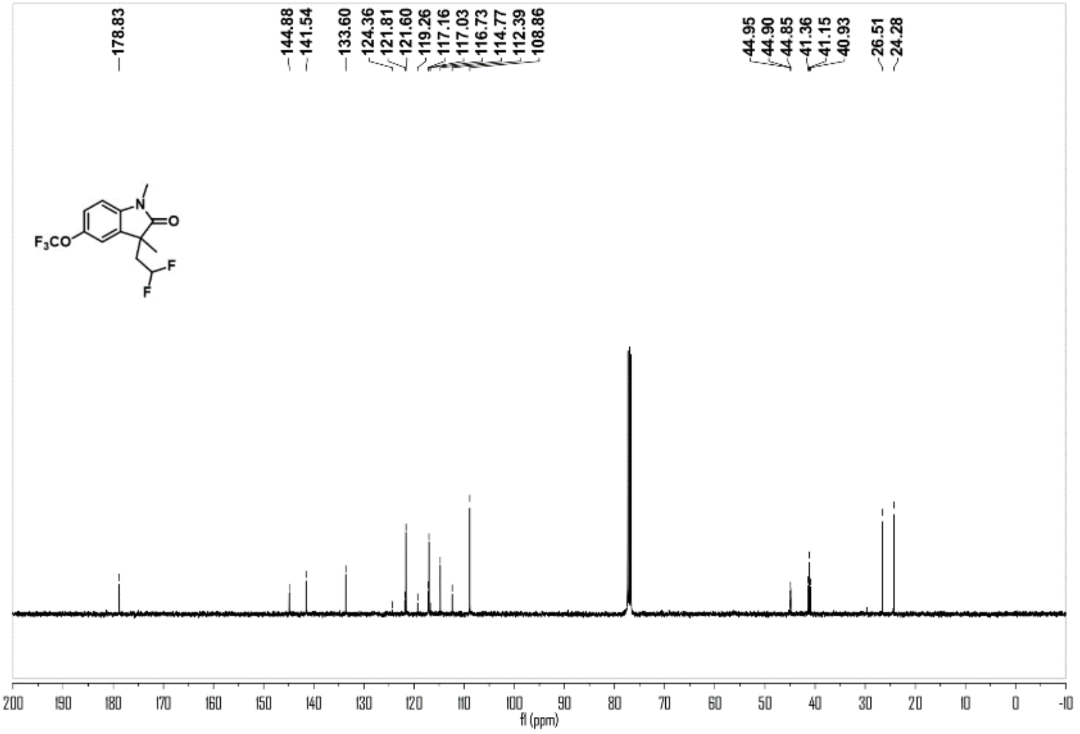
178.83
144.88
141.54
133.60
124.36
121.81
121.60
119.26
117.16
117.03
116.73
114.77
112.39
108.86
44.95
44.90
44.85
41.36
41.15
40.93
26.51
24.28
F3CO
N
O
F
F
f1 (ppm)

^{1}H, ^{19}F and ^{13}C NMR spectra of compound 5i

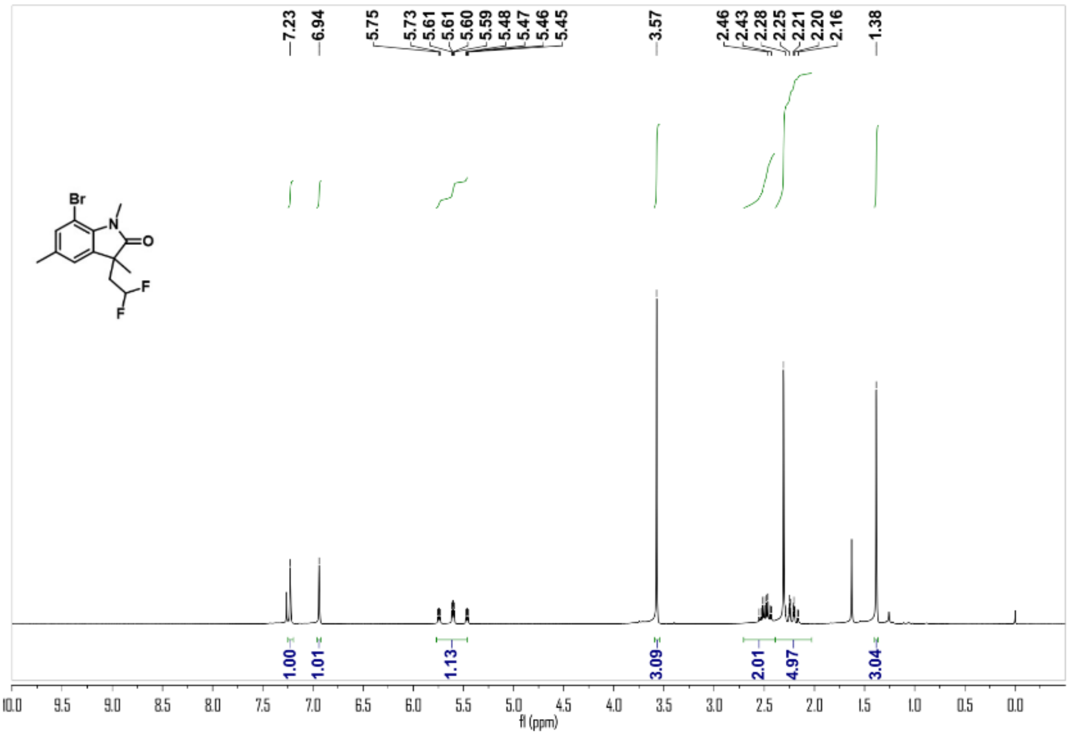

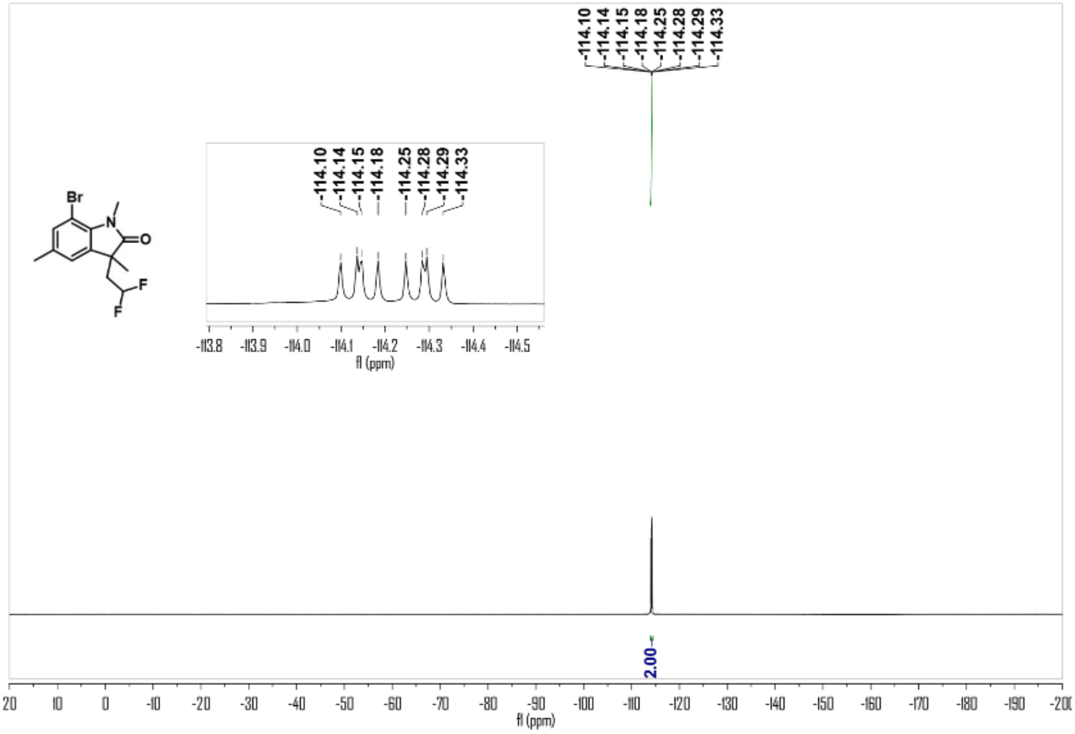

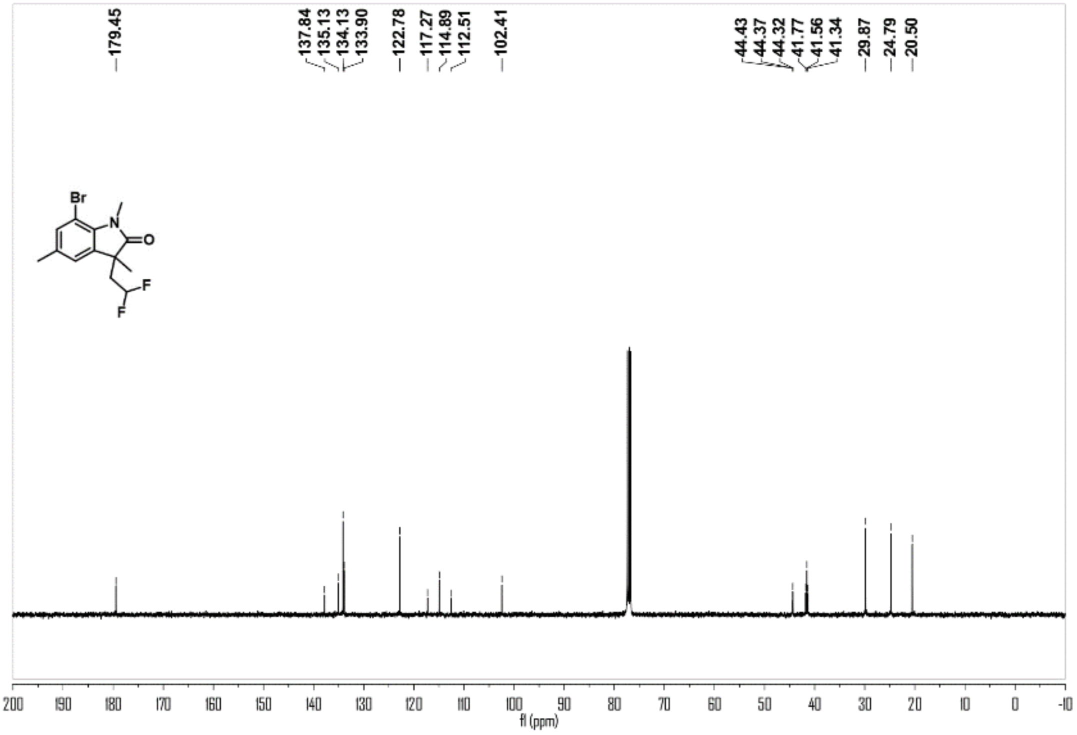

^{1}H, ^{19}F and ^{13}C NMR spectra of compound 5j

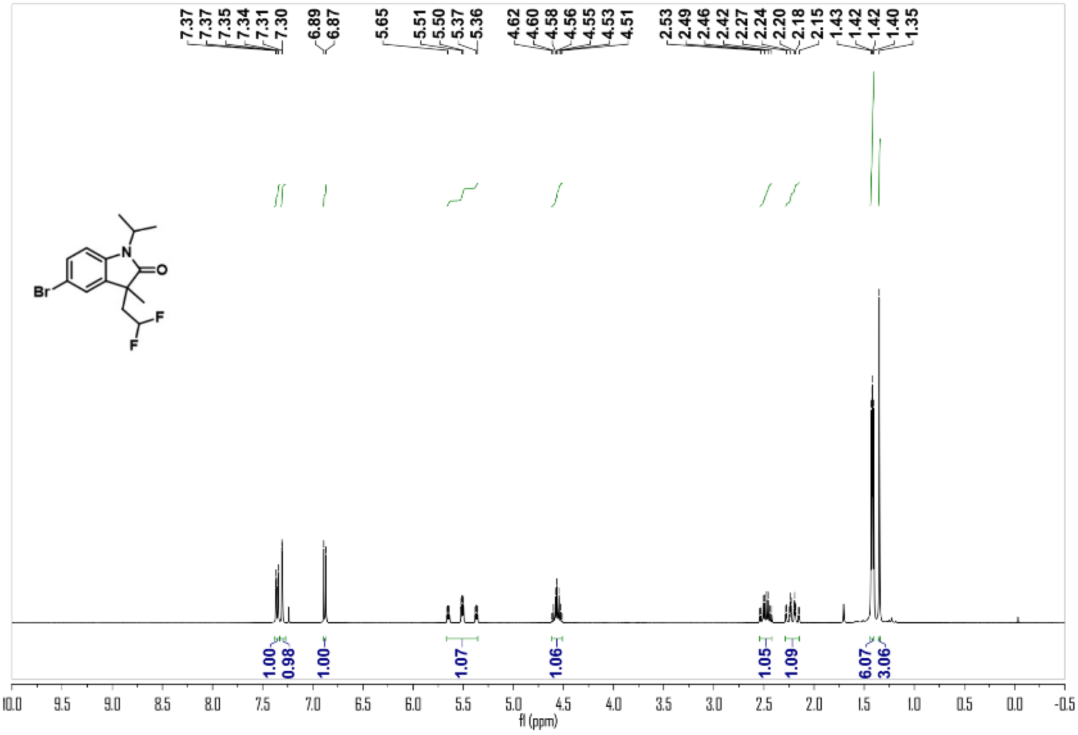

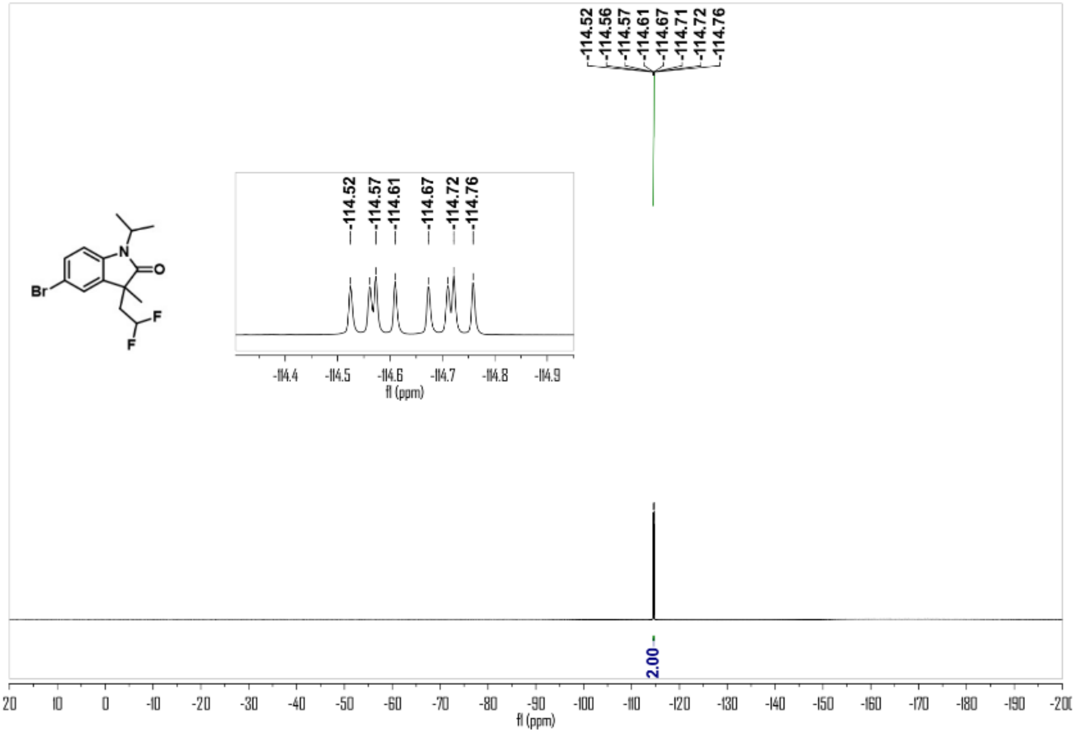
-114.52
-114.56
-114.57
-114.61
-114.67
-114.71
-114.72
-114.76
Br
N
O
F
F
2.00
fl (ppm)

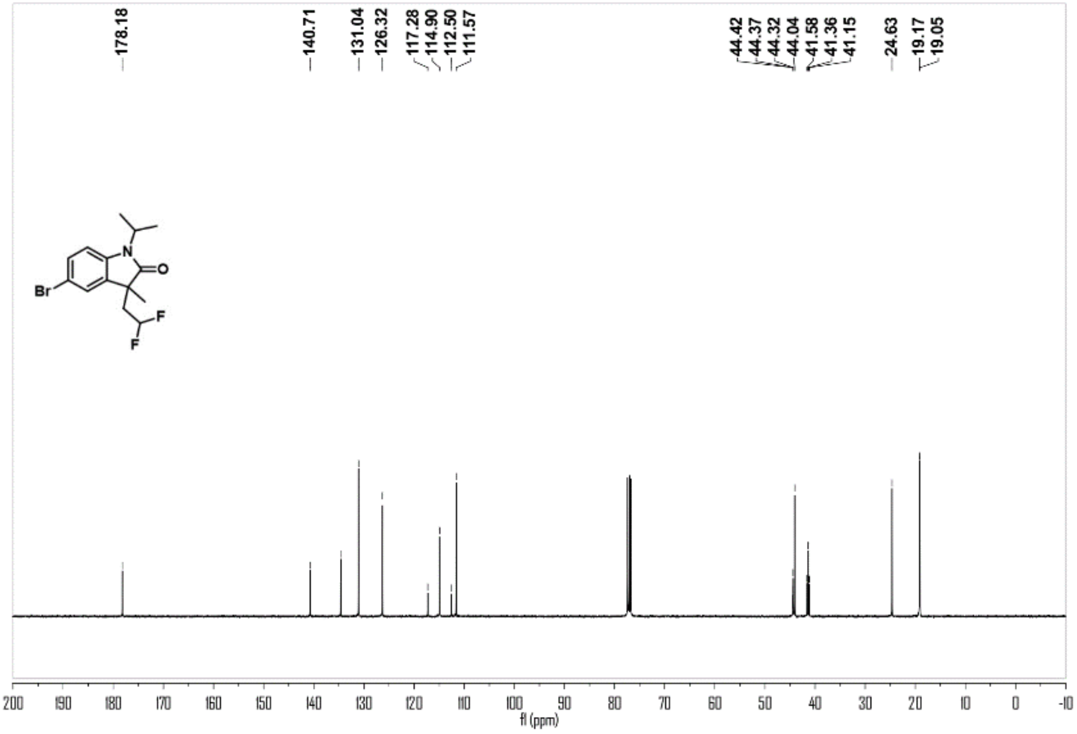
178.18
140.71
131.04
126.32
117.28
114.90
112.50
111.57
44.42
44.37
44.32
44.04
41.58
41.36
41.15
24.63
19.17
19.05
Br
N
O
F
F
fl (ppm)

^{1}H, ^{19}F and ^{13}C NMR spectra of compound 5k

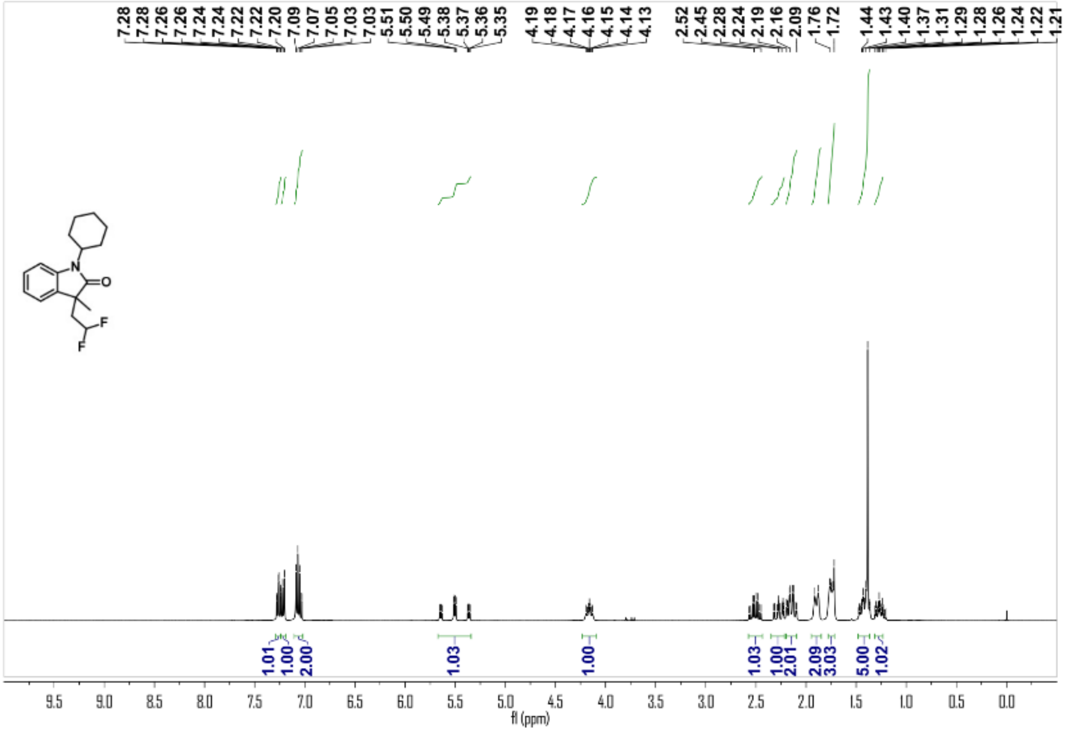

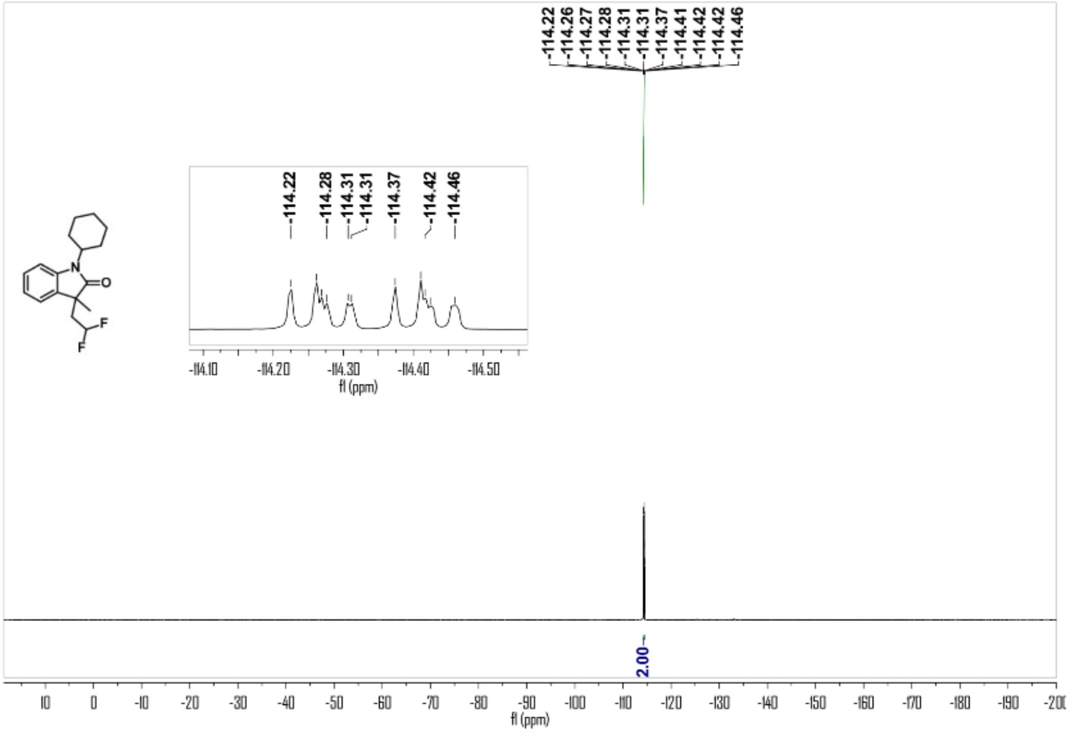

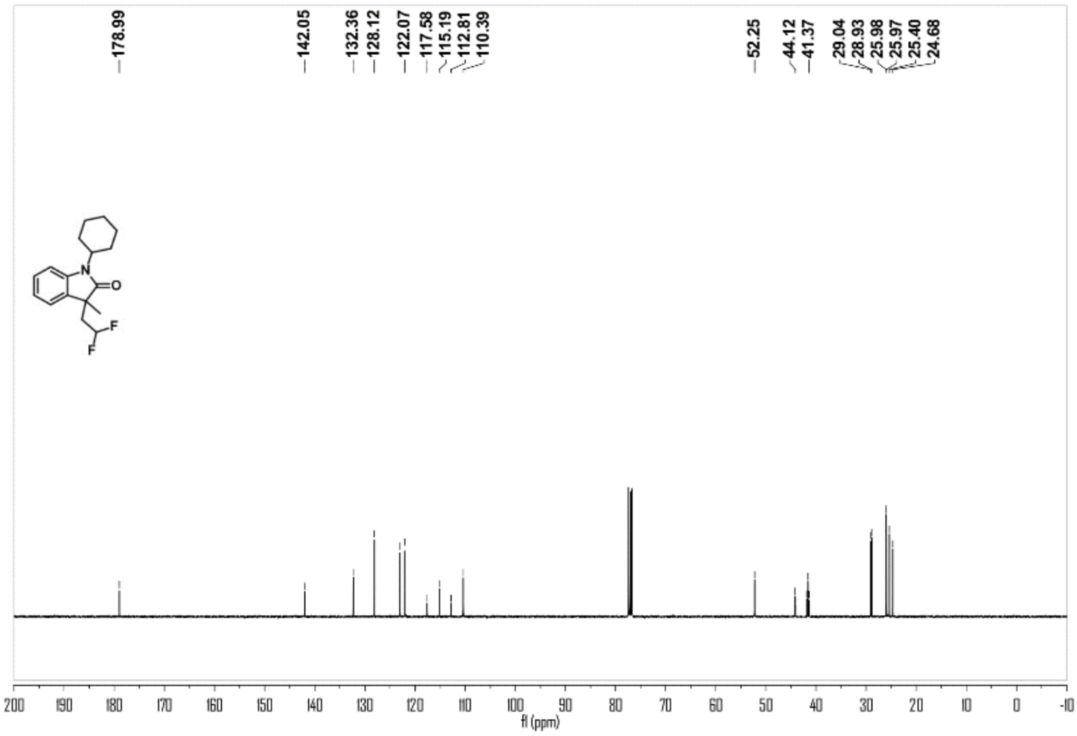

^{1}H, ^{19}F and ^{13}C NMR spectra of compound 5l

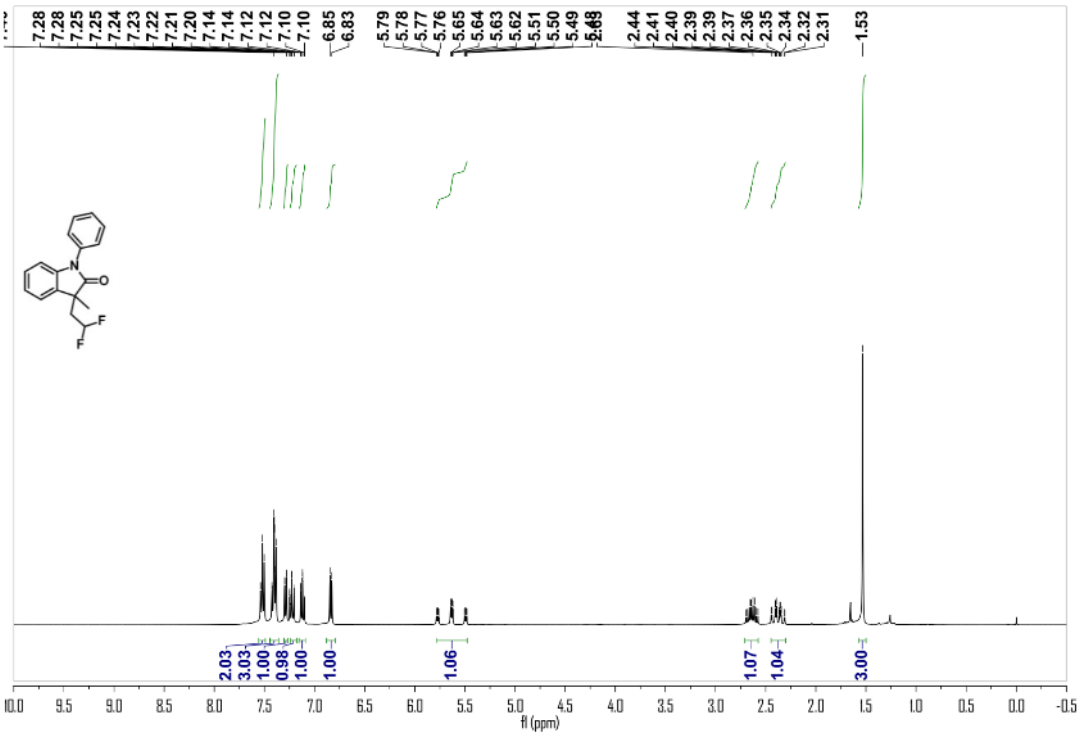

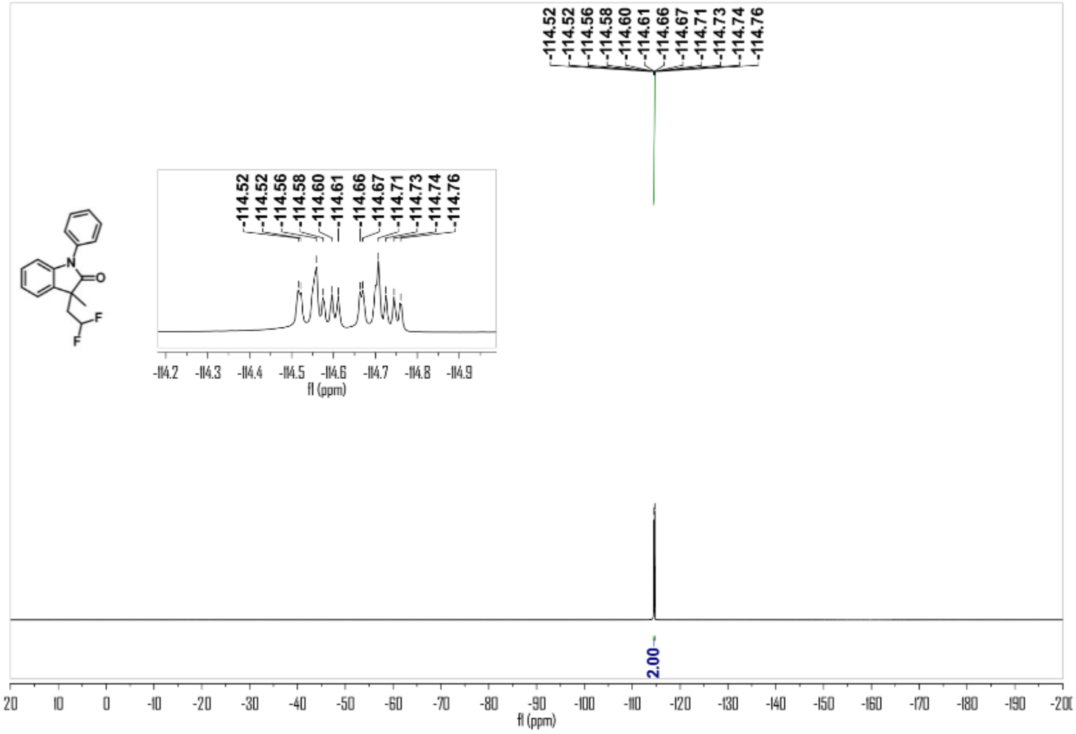
-114.52
-114.52
-114.56
-114.58
-114.60
-114.61
-114.66
-114.67
-114.71
-114.73
-114.74
-114.76
2.00
f1 (ppm)

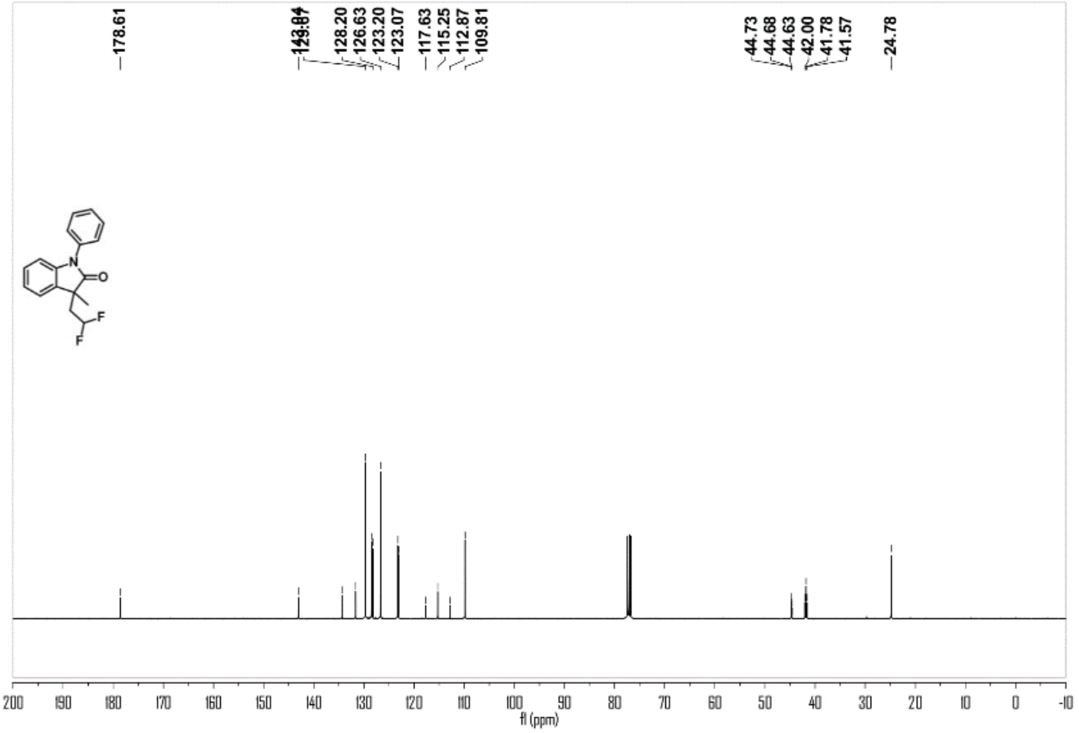
178.61
128.20
126.63
123.20
123.07
117.63
115.25
112.87
109.81
44.73
44.68
44.63
42.00
41.78
41.57
24.78
f1 (ppm)

^{1}H, ^{19}F and ^{13}C NMR spectra of compound 5m

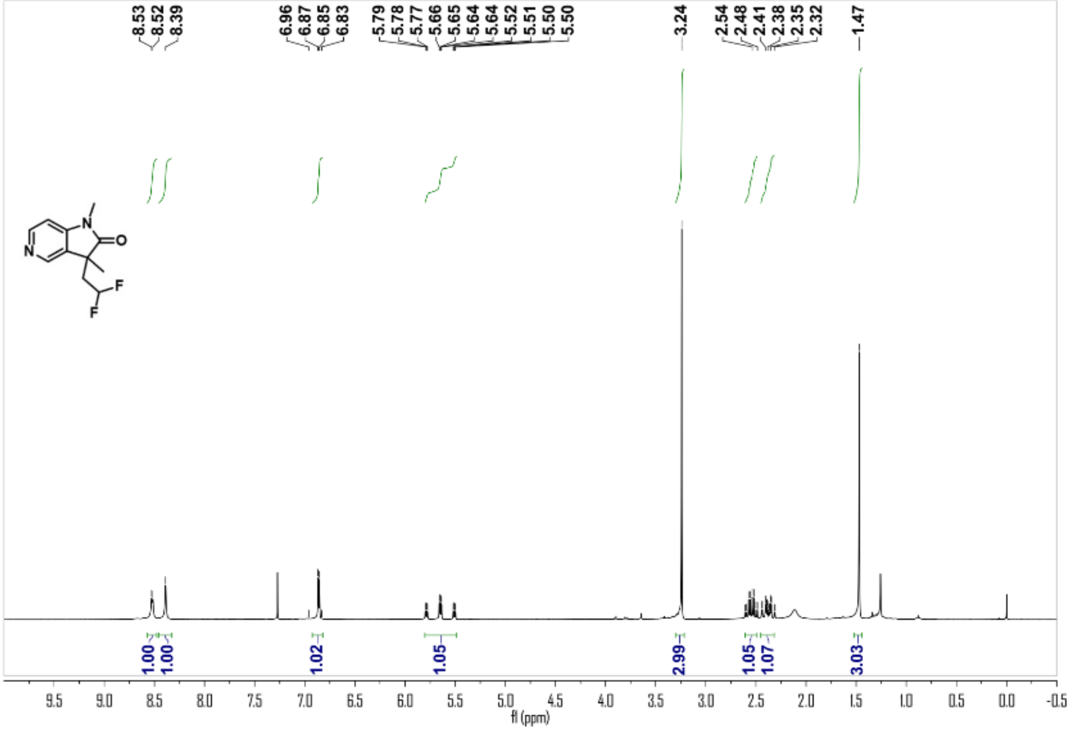

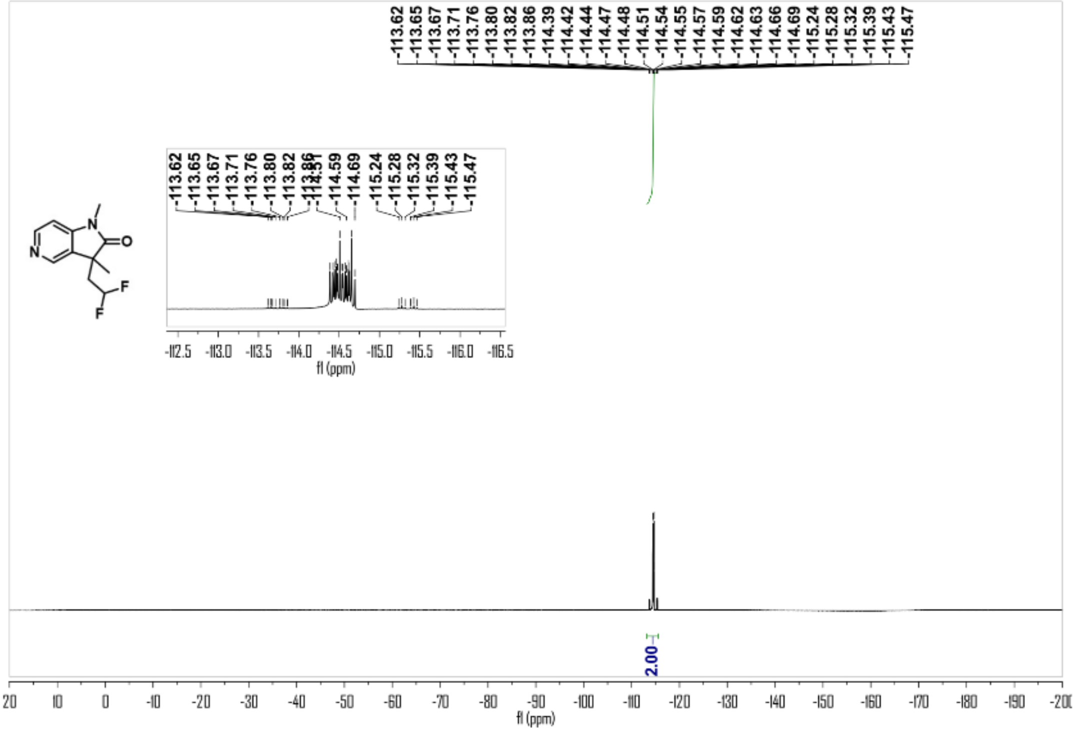

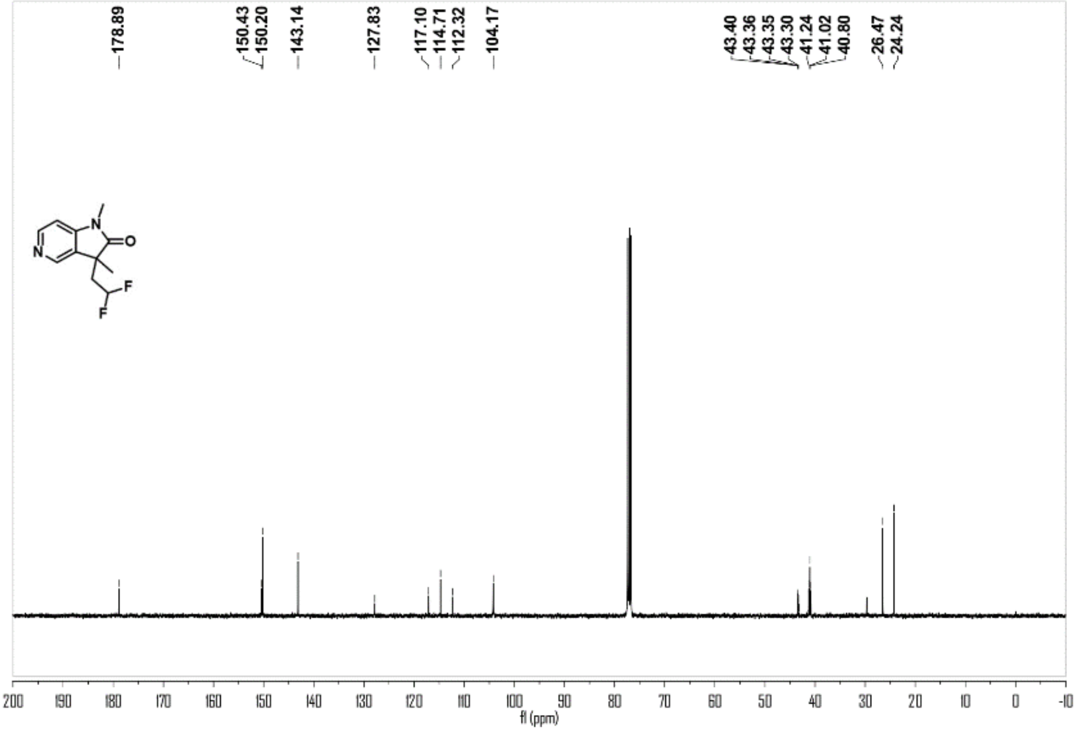

^{1}H, ^{19}F and ^{13}C NMR spectra of compound 5n

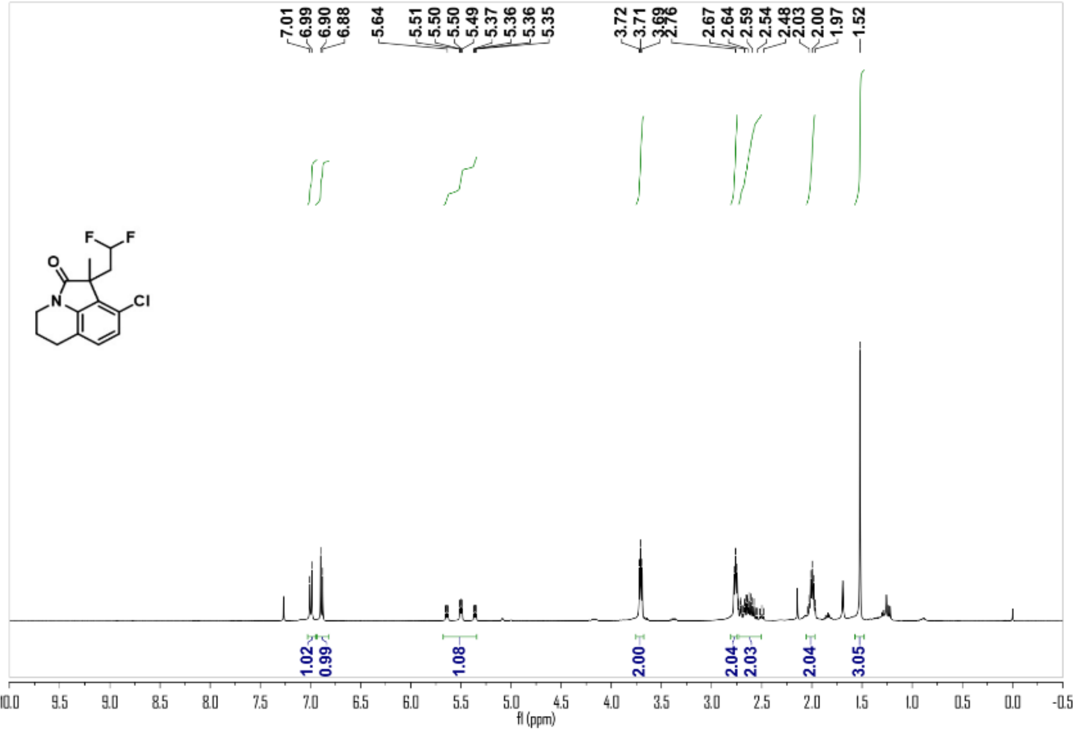

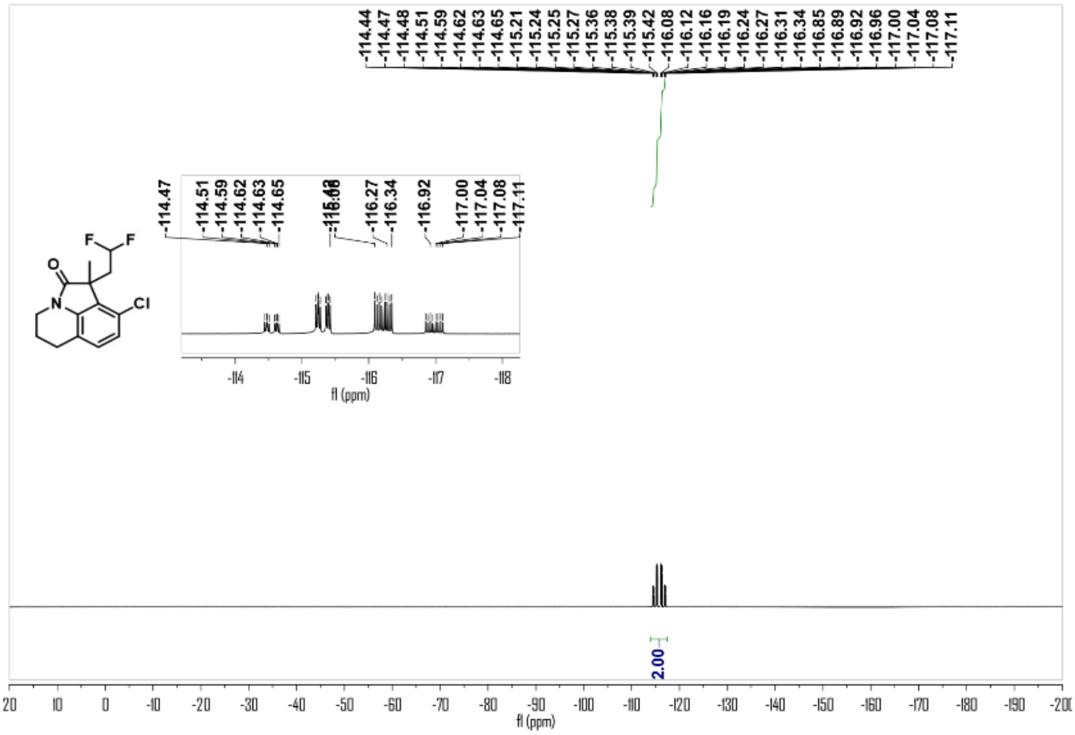

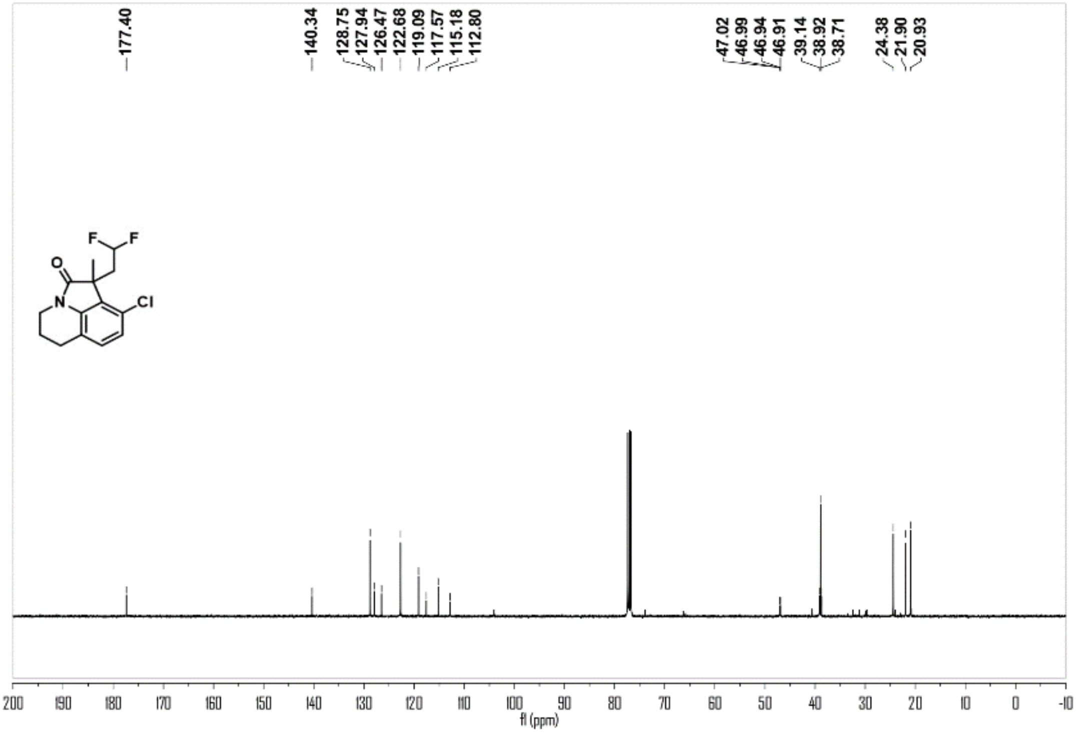

^{1}H, ^{19}F and ^{13}C NMR spectra of compound 5o

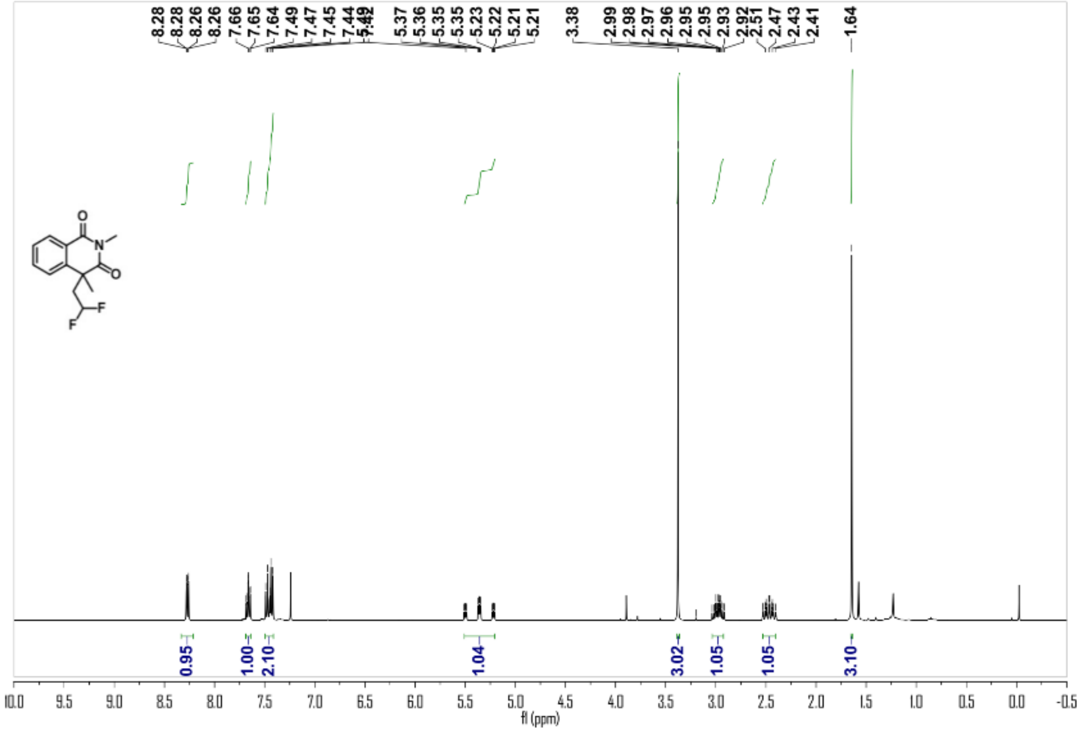

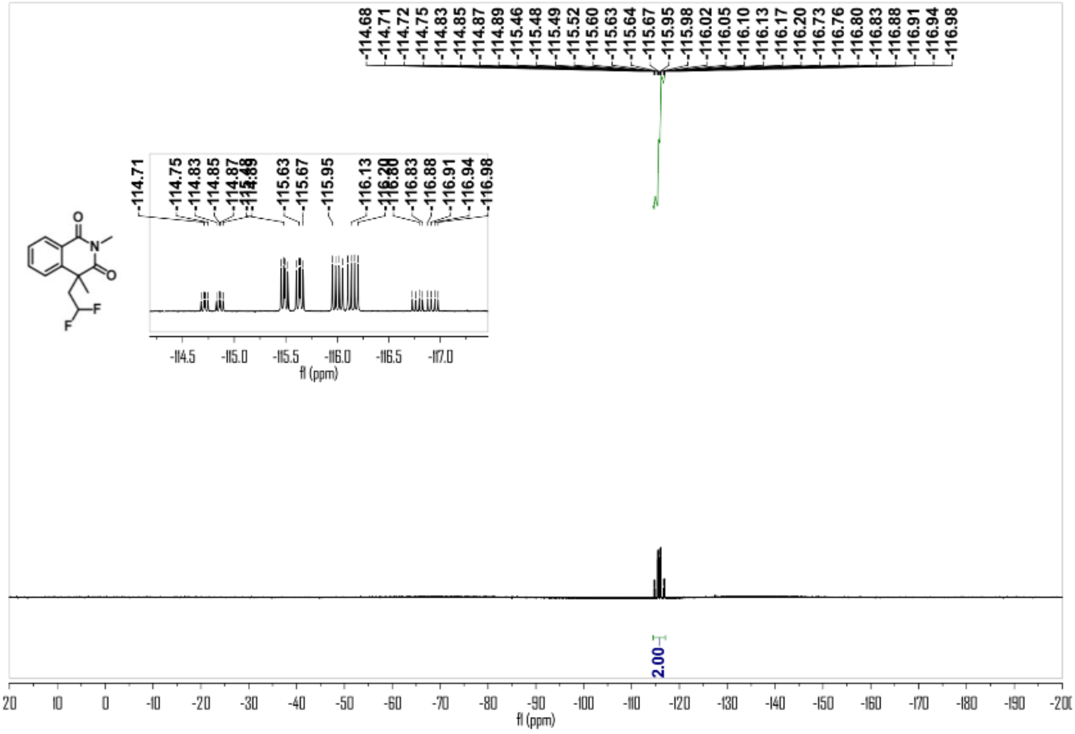

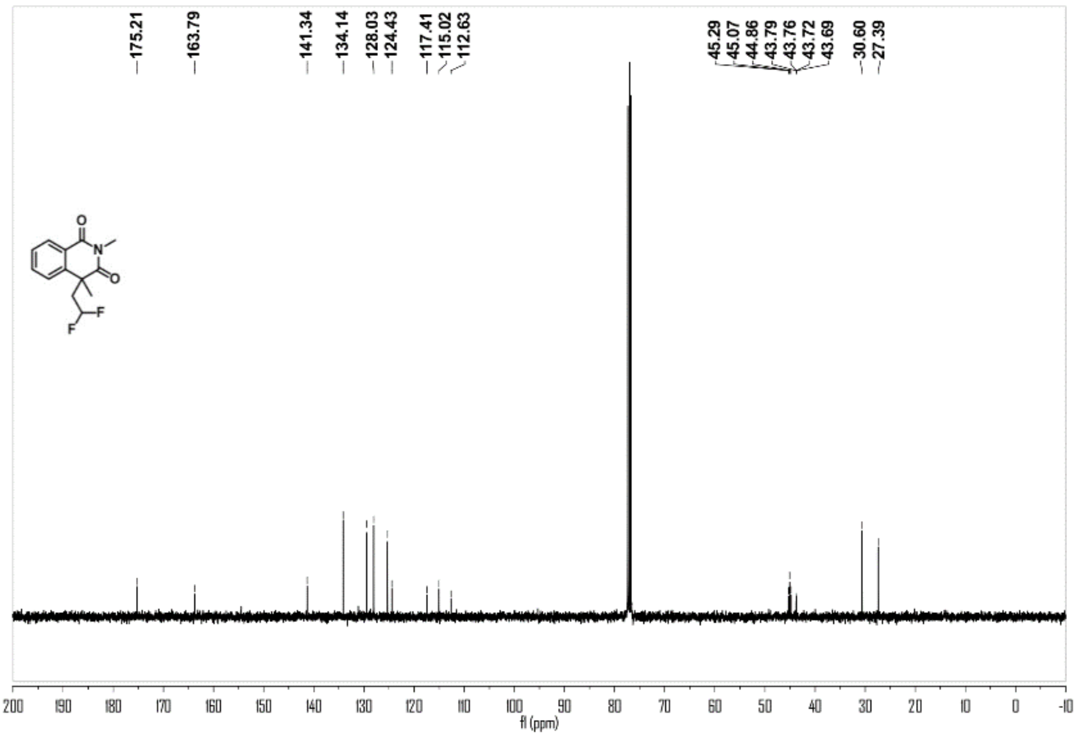

^{1}H, ^{19}F and ^{13}C NMR spectra of compound 5p

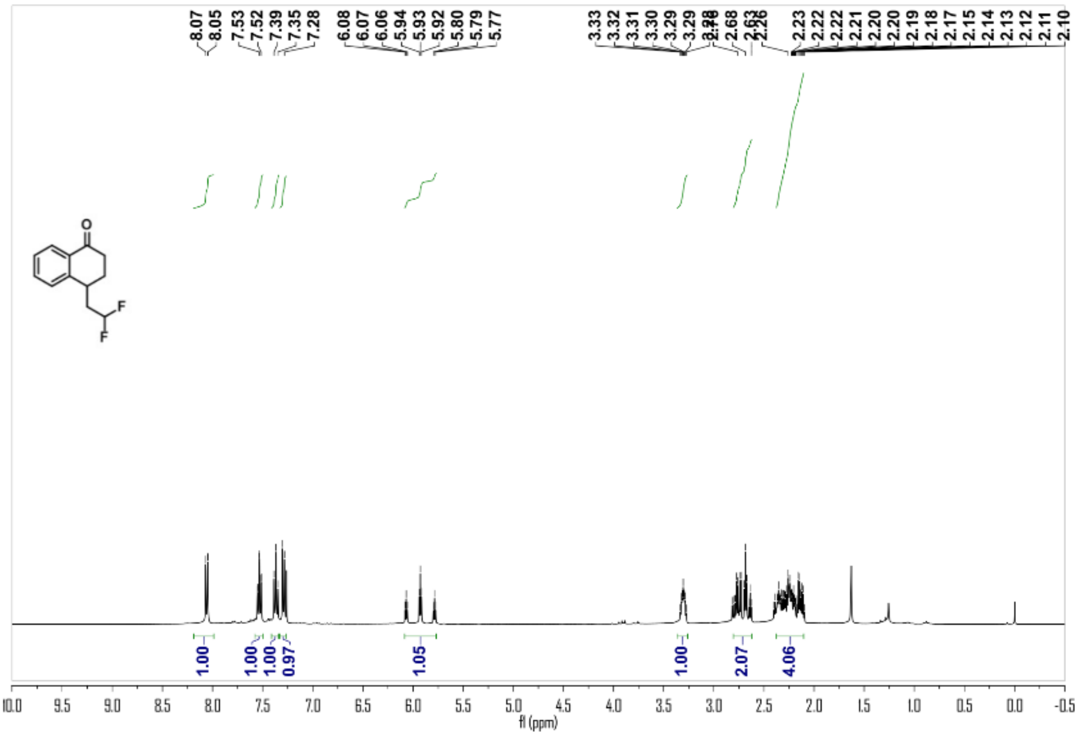

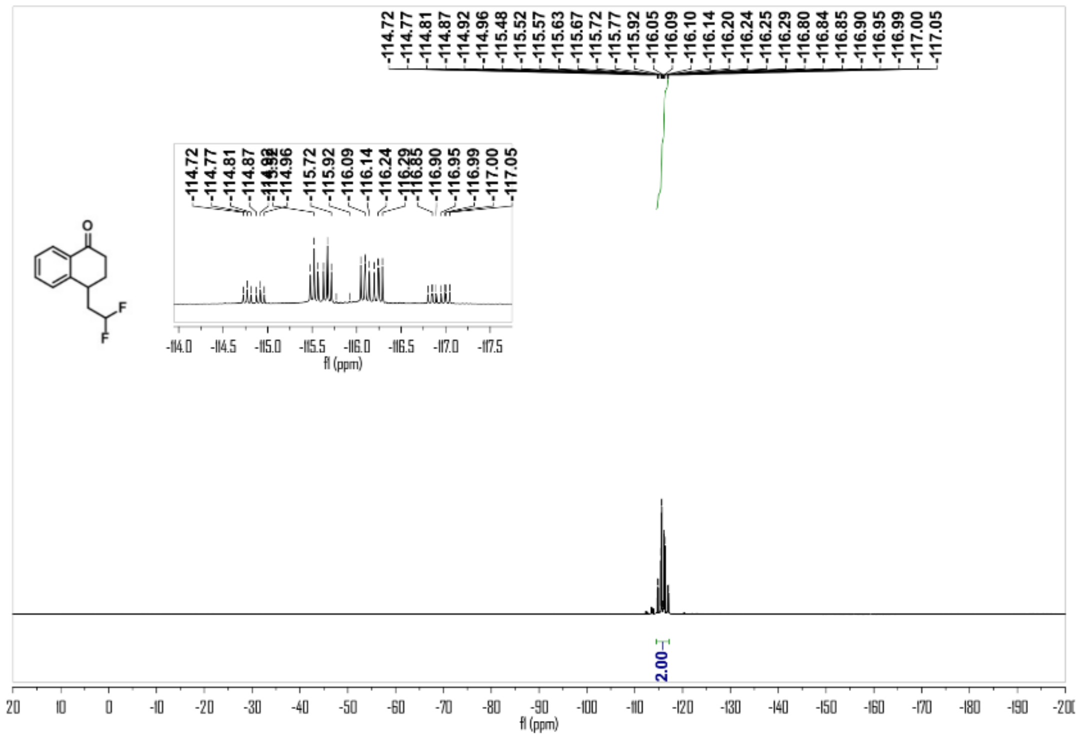

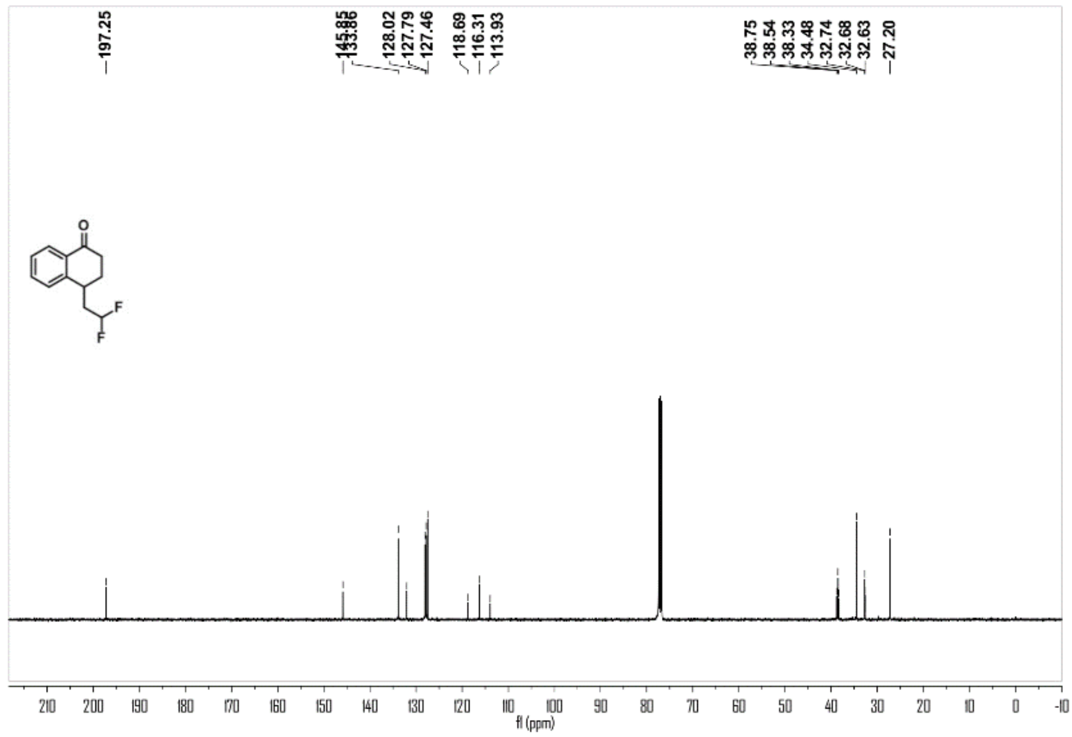

^{1}H, ^{19}F and ^{13}C NMR spectra of compound 5q

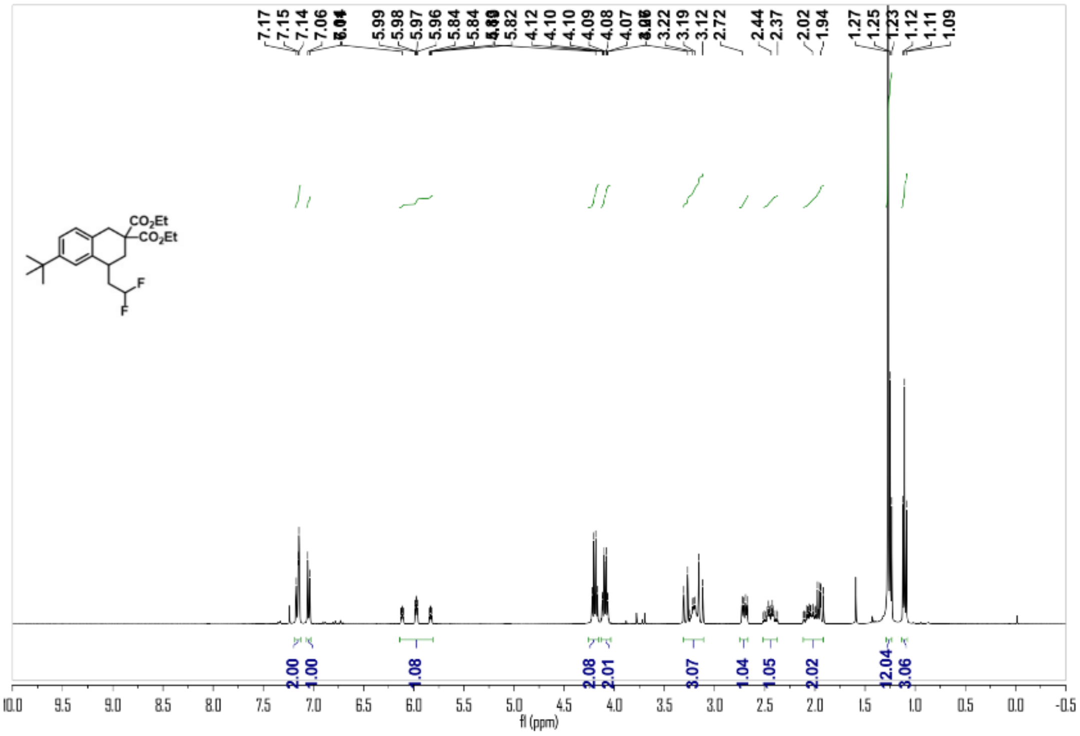

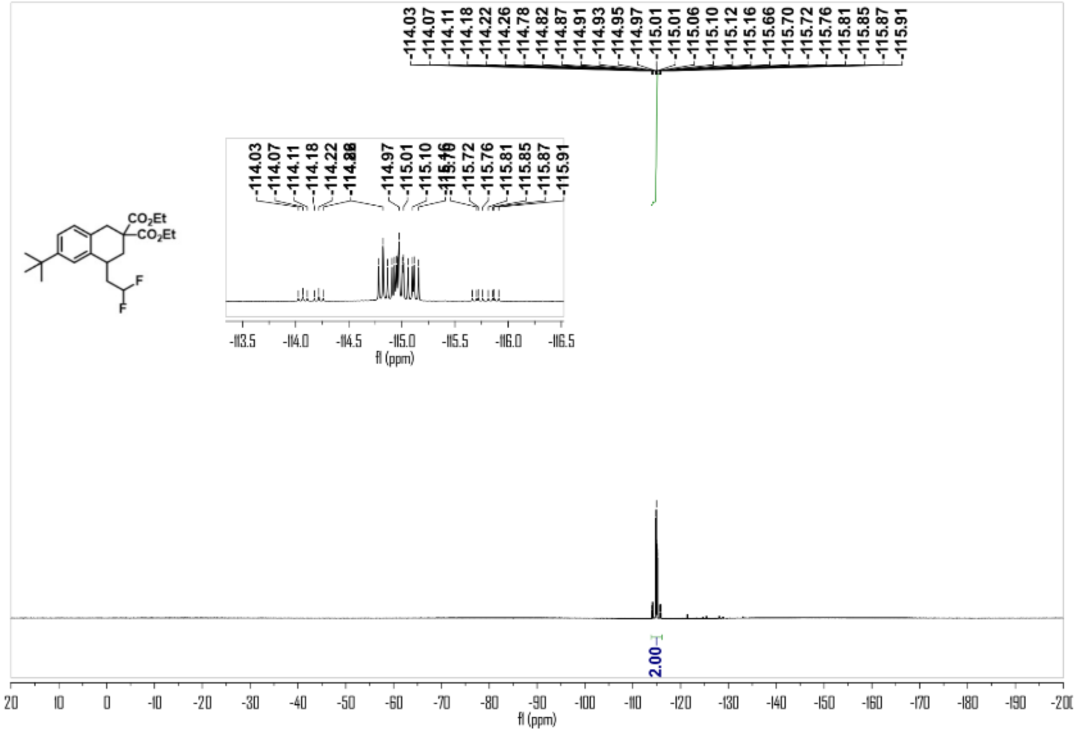

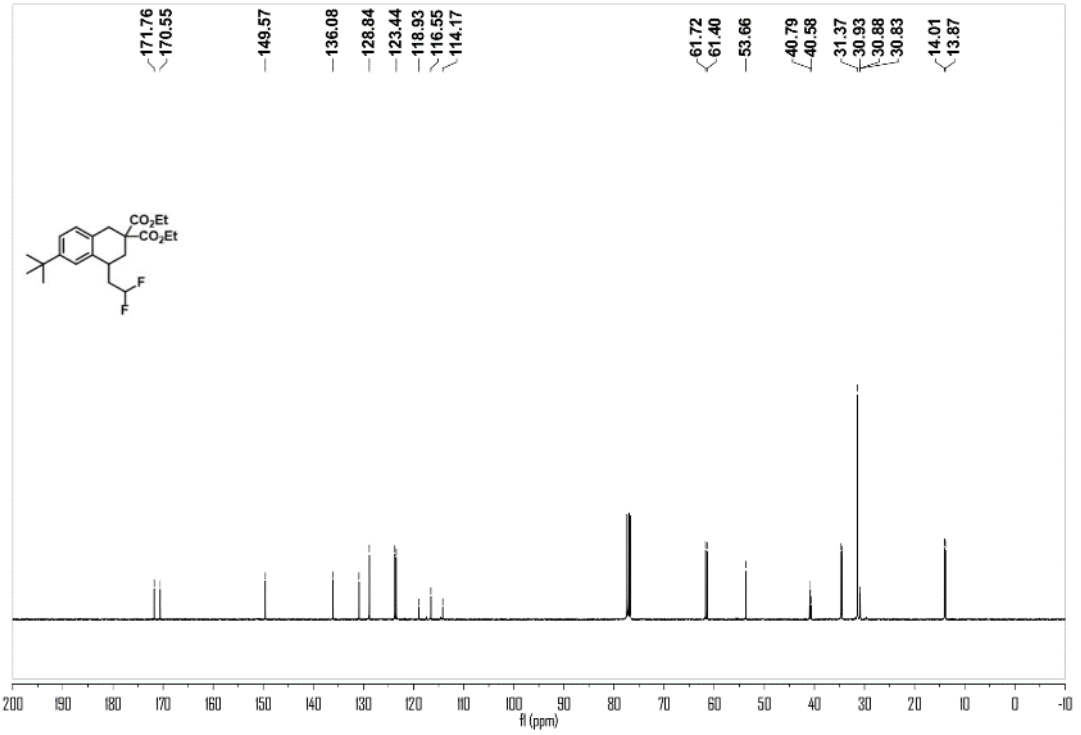

^{1}H, ^{19}F and ^{13}C NMR spectra of compound 5r

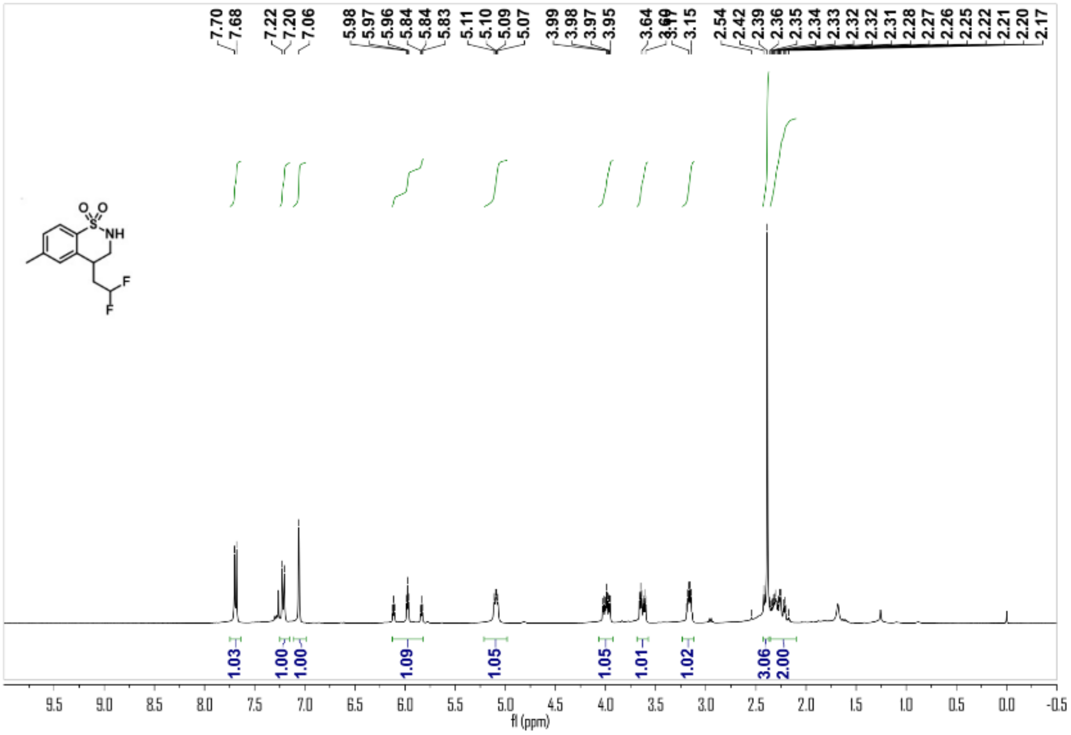

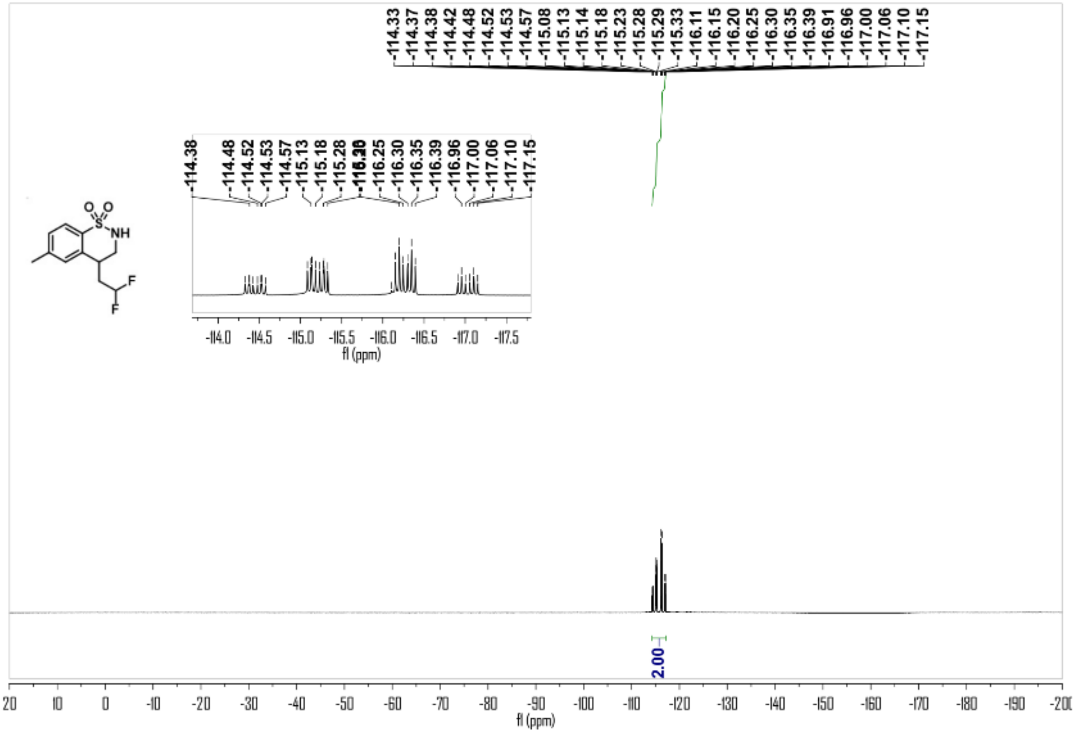
-114.33
-114.37
-114.38
-114.42
-114.48
-114.52
-114.53
-114.57
-115.08
-115.13
-115.14
-115.18
-115.23
-115.28
-115.29
-115.33
-116.11
-116.15
-116.20
-116.25
-116.30
-116.35
-116.39
-116.91
-116.96
-117.00
-117.06
-117.10
-117.15
NH
F
F
2.00
f1 (ppm)

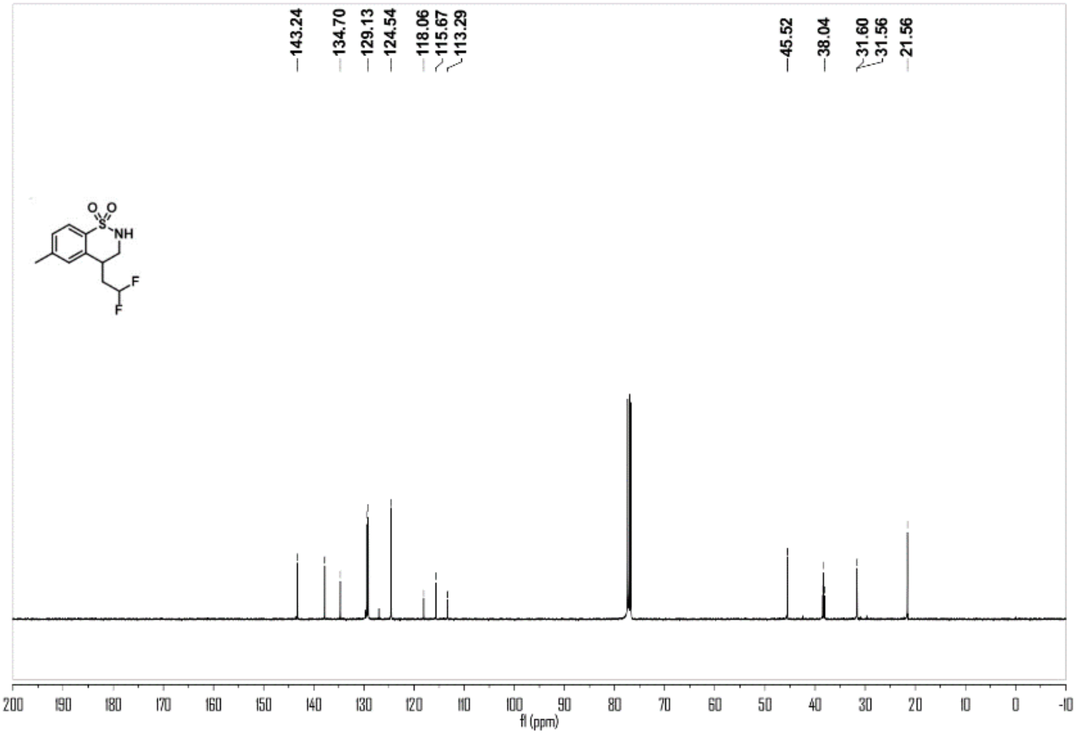
143.24
134.70
129.13
124.54
118.06
115.67
113.29
45.52
38.04
31.60
31.56
21.56
NH
F
F
f1 (ppm)

^{1}H, ^{19}F and ^{13}C NMR spectra of compound 5s

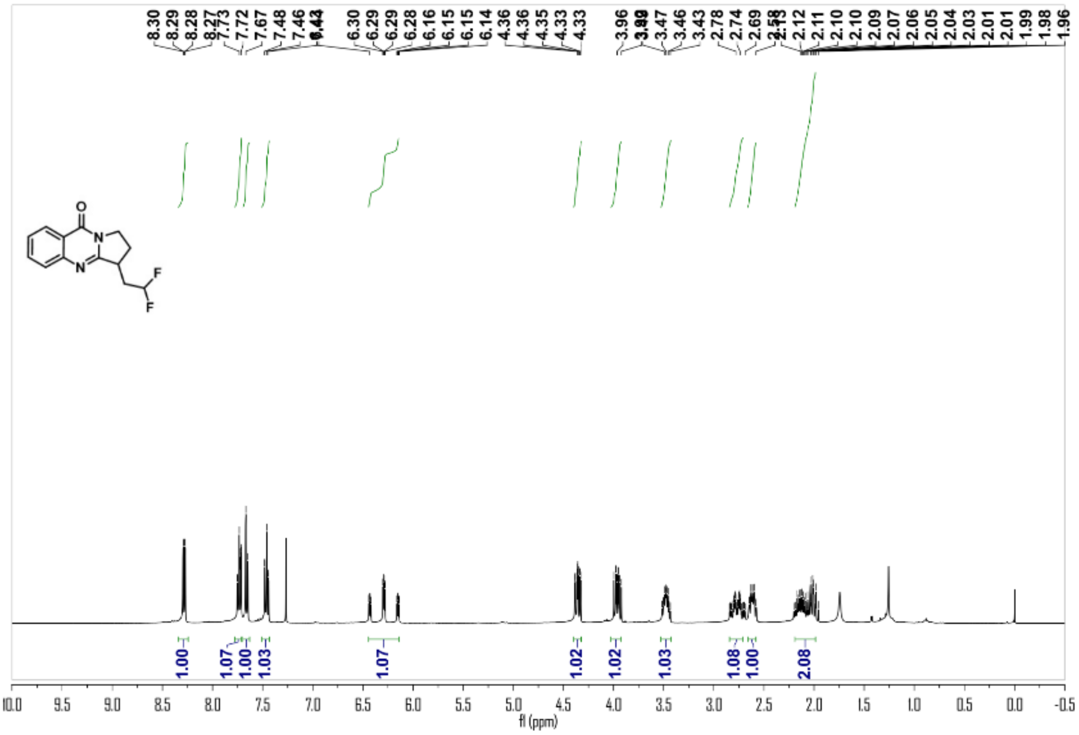

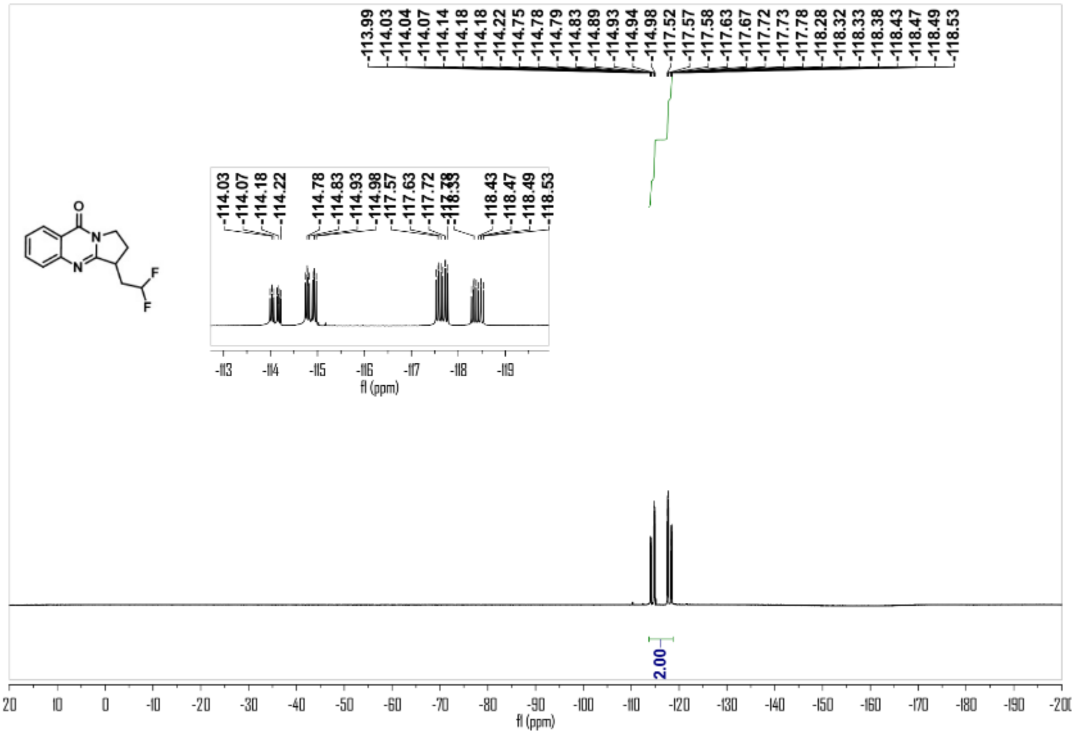

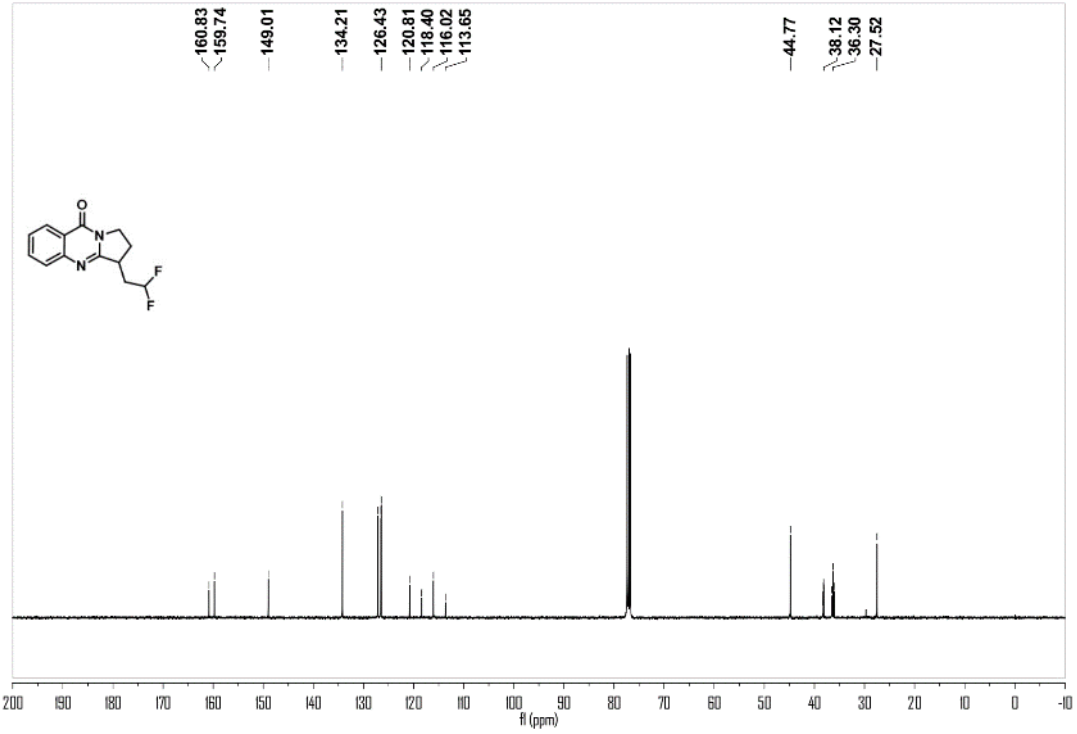

^{1}H, ^{19}F and ^{13}C NMR spectra of compound 5t

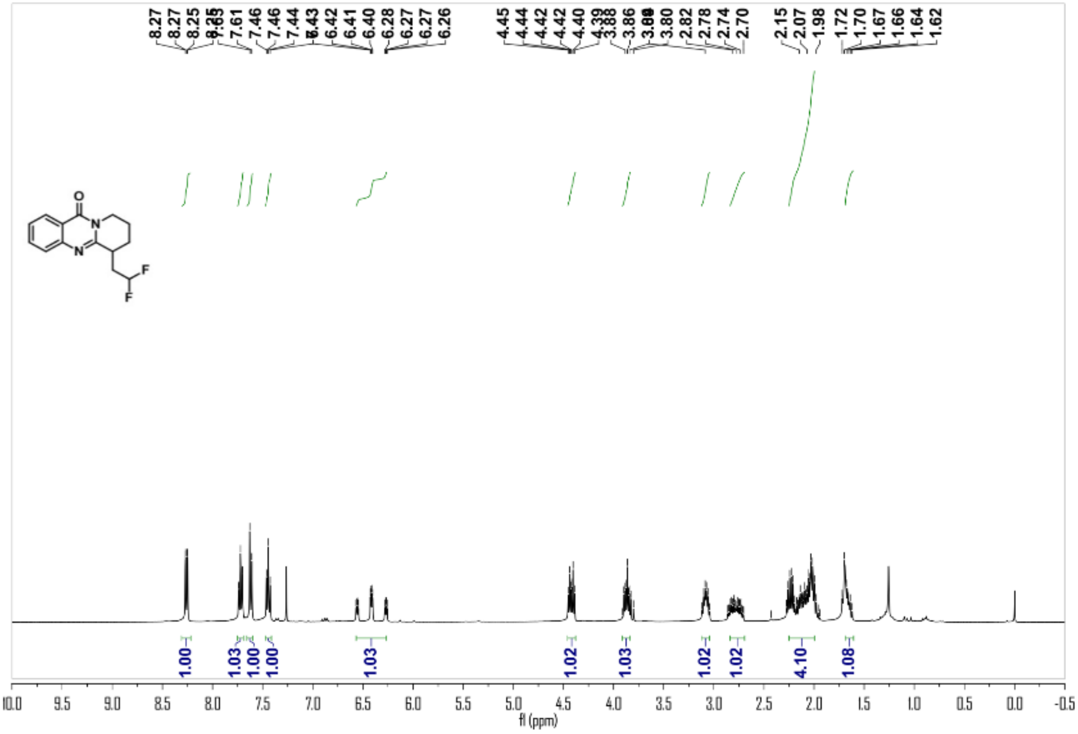

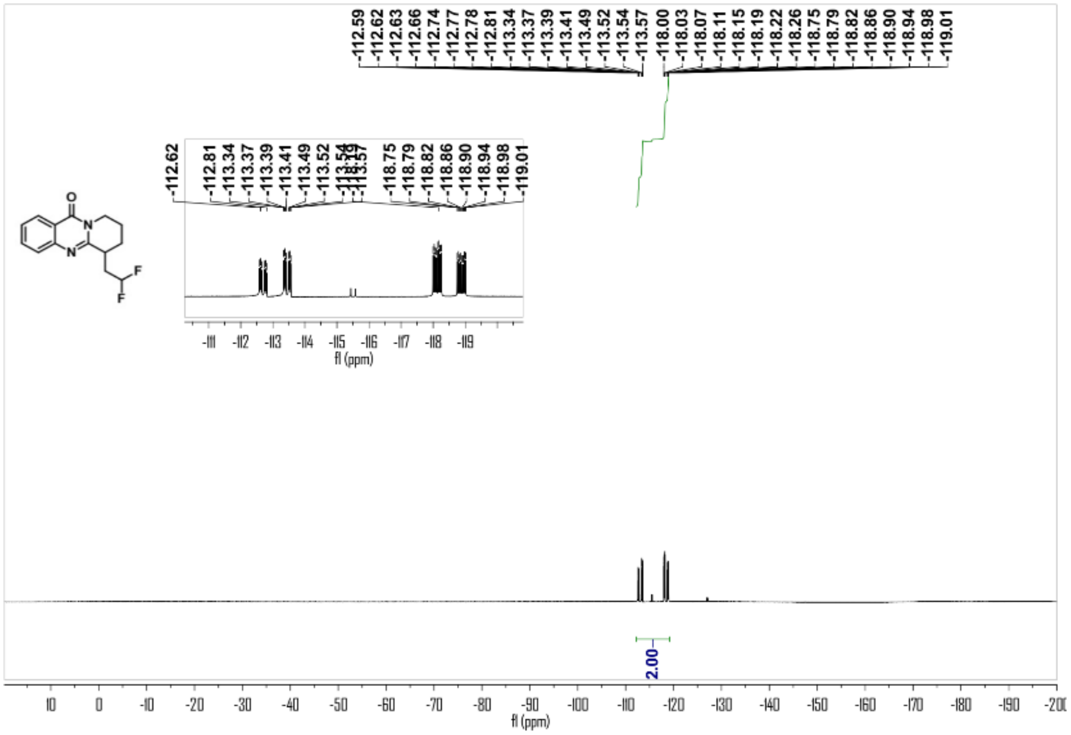

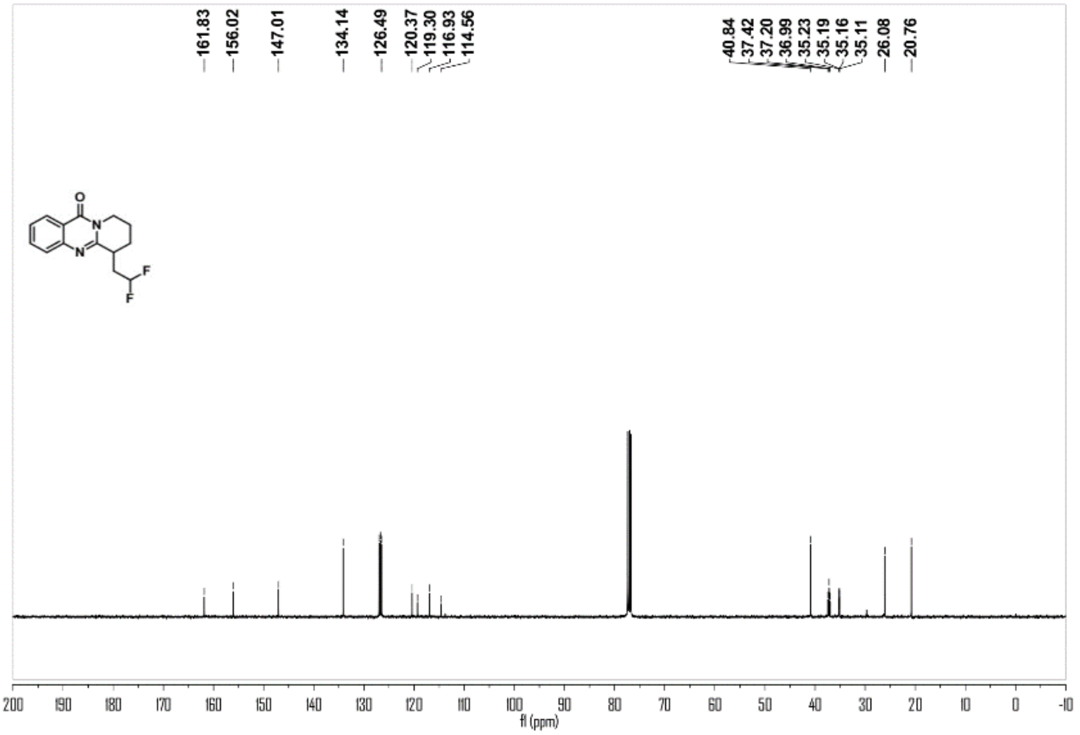

^{1}H, ^{19}F and ^{13}C NMR spectra of compound 5u

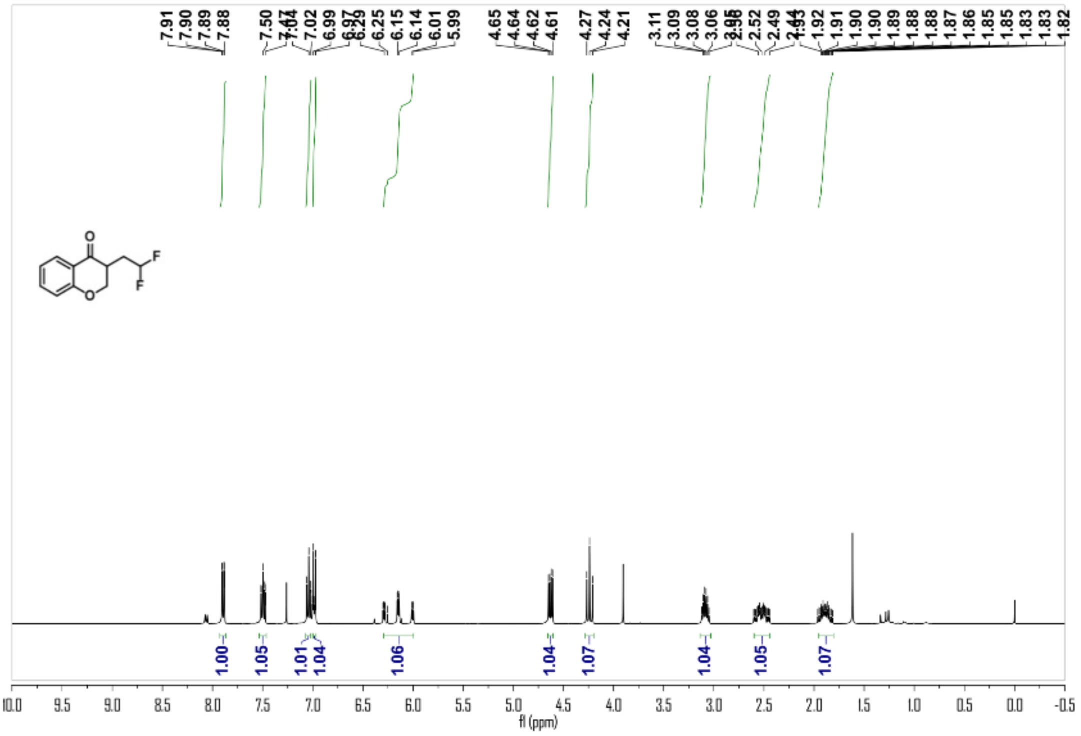

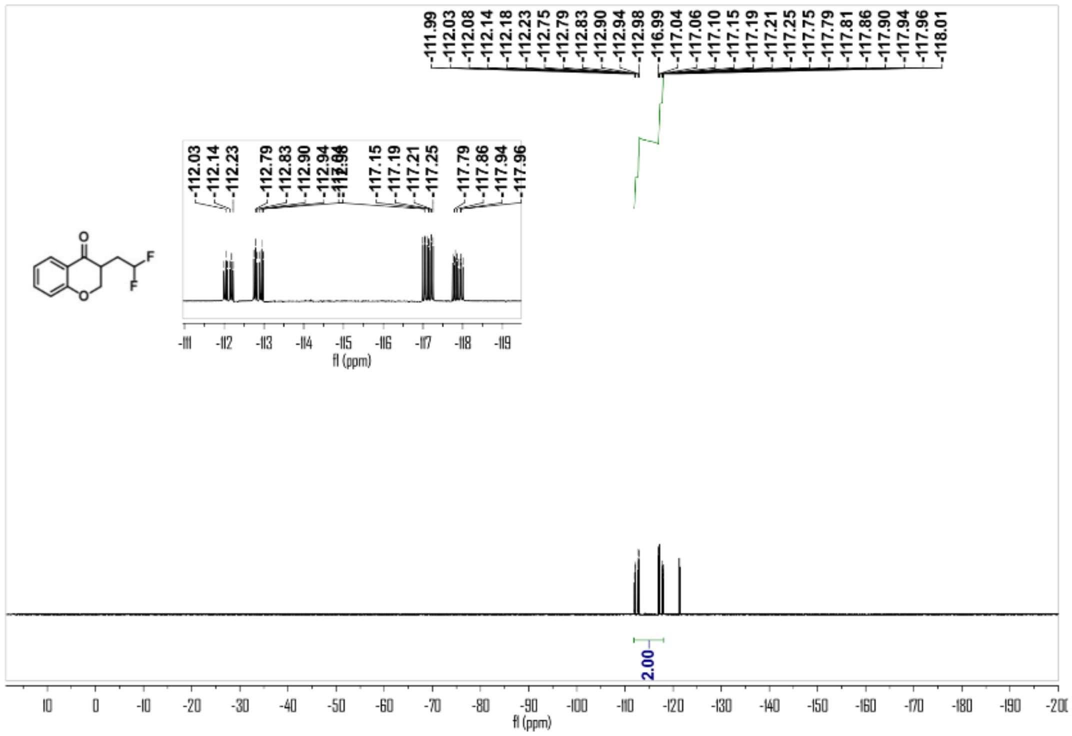

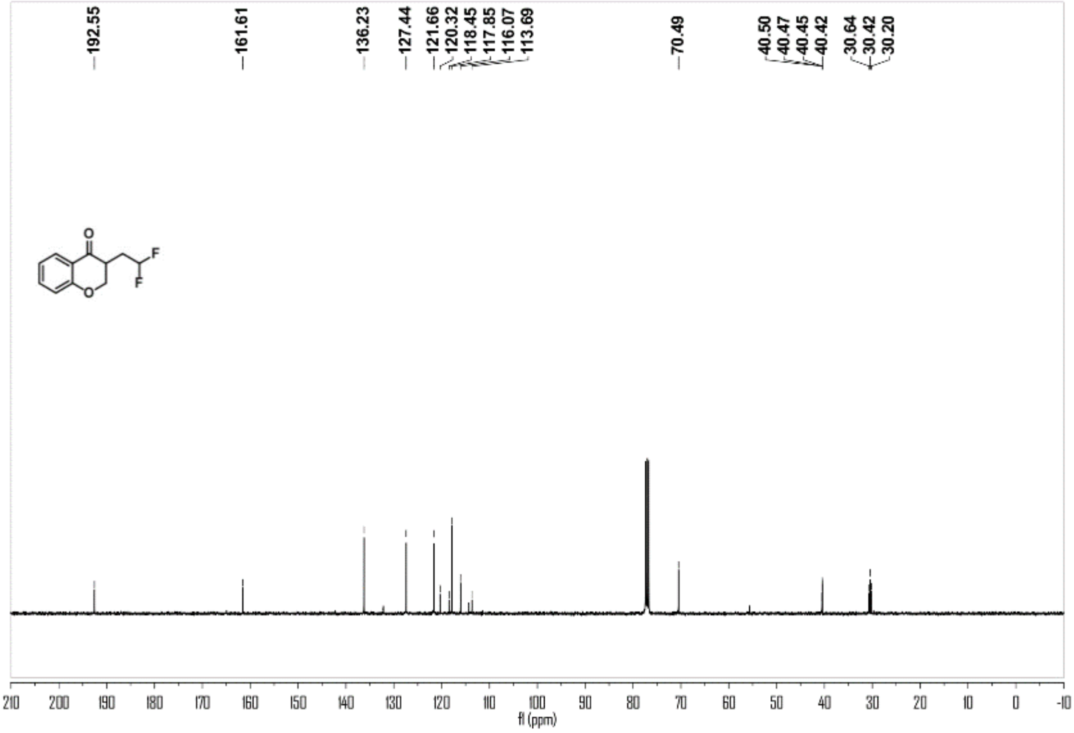

^{1}H, ^{19}F and ^{13}C NMR spectra of compound 5v

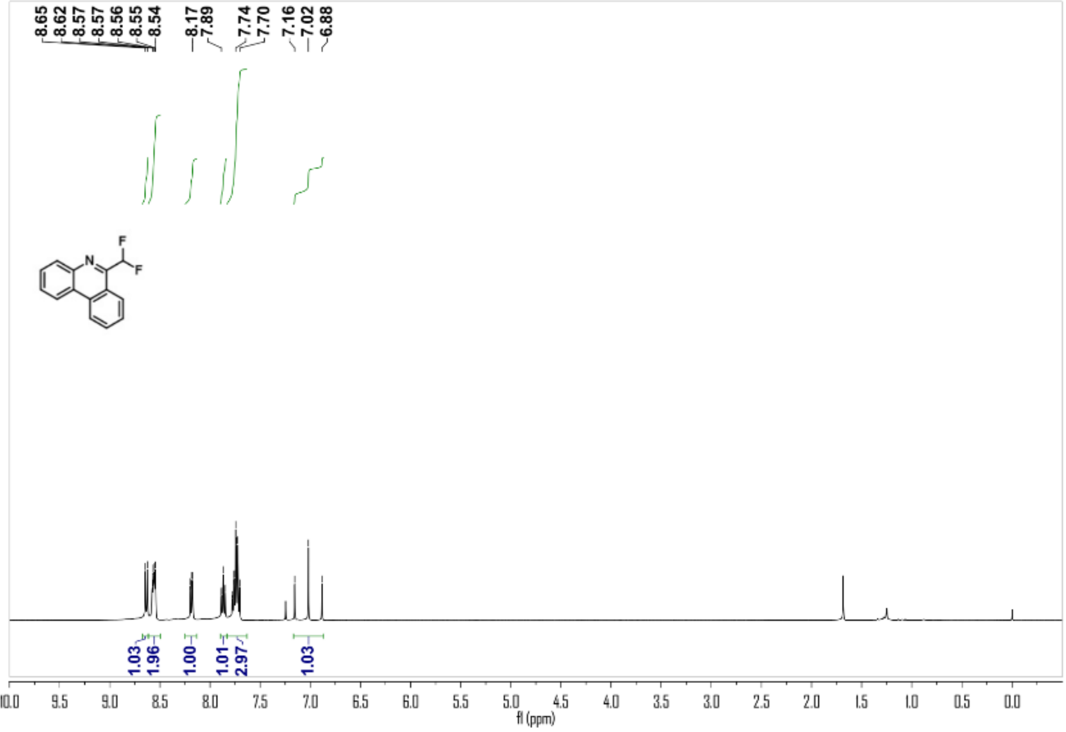

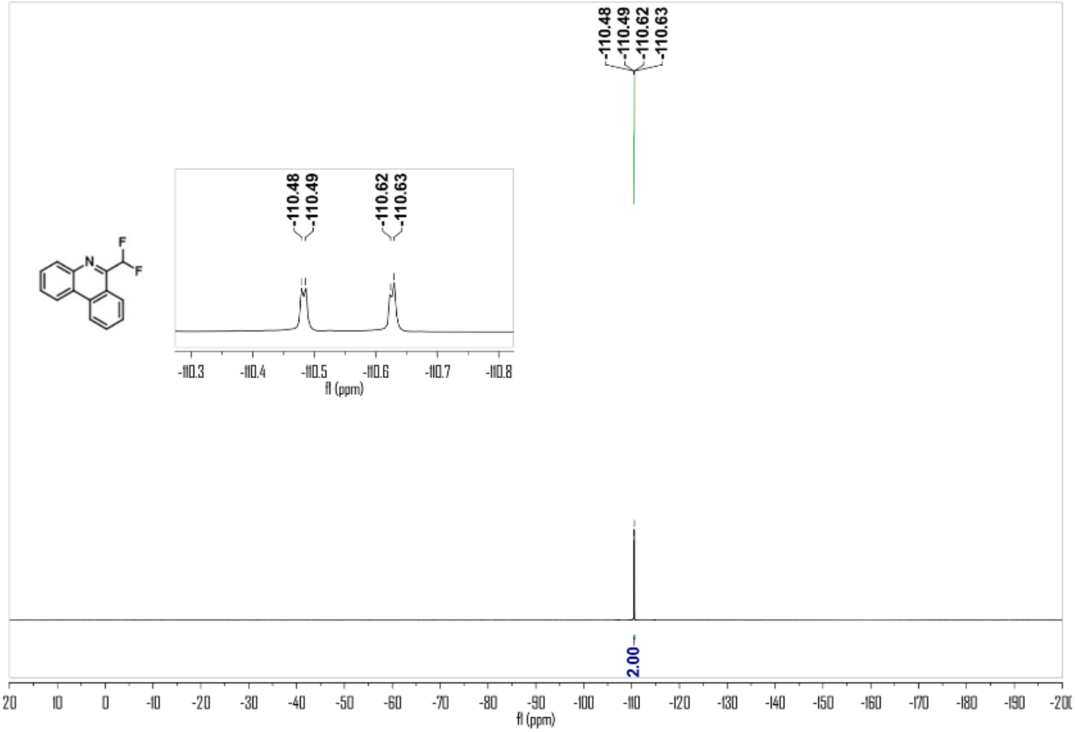
-110.48
-110.49
-110.62
-110.63
2.00
20 10 0 -10 -20 -30 -40 -50 -60 -70 -80 -90 -100 -110 -120 -130 -140 -150 -160 -170 -180 -190 -200
f1 (ppm)
-110.3 -110.4 -110.5 -110.6 -110.7 -110.8
N
F
F

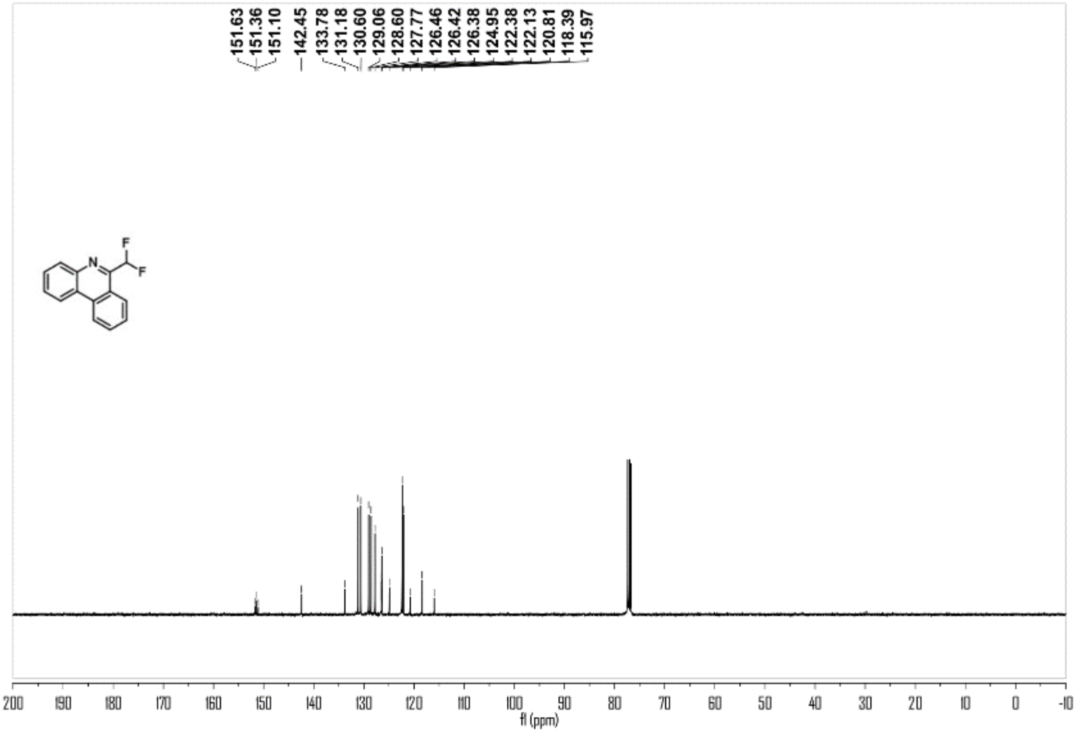
151.63
151.36
151.10
142.45
133.78
131.18
130.60
129.06
128.60
127.77
126.46
126.42
126.38
124.95
122.38
122.13
120.81
118.39
115.97
200 190 180 170 160 150 140 130 120 110 100 90 80 70 60 50 40 30 20 10 0 -10
f1 (ppm)
N
F
F

^{1}H, ^{19}F and ^{13}C NMR spectra of compound 5w

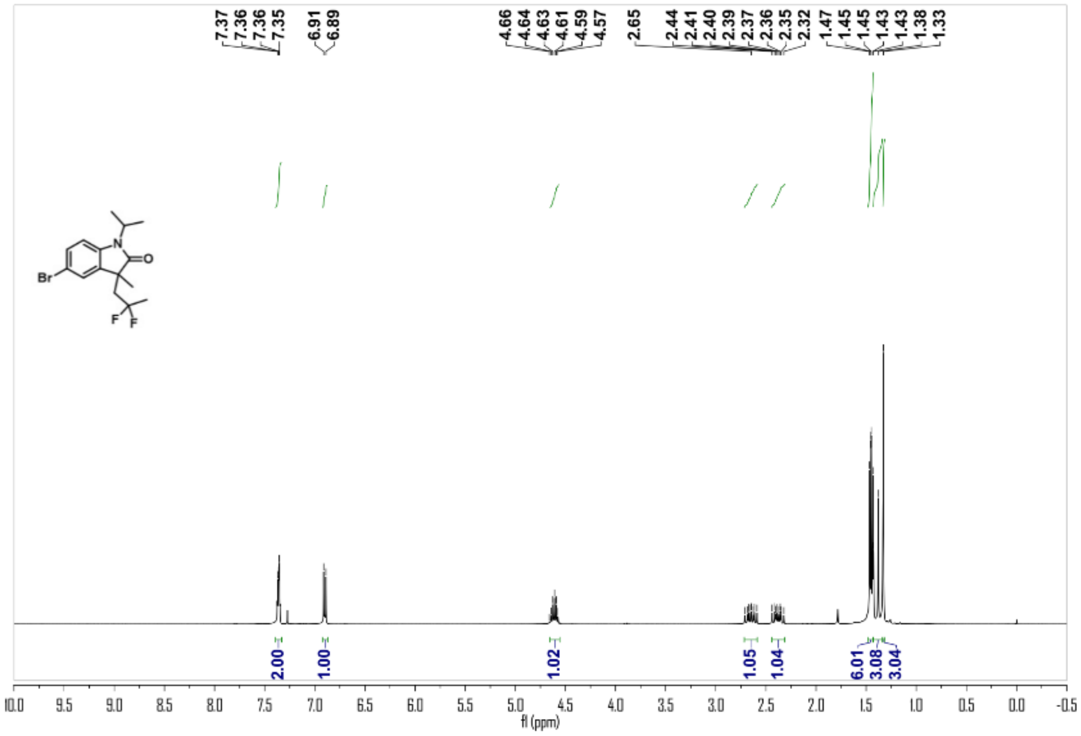

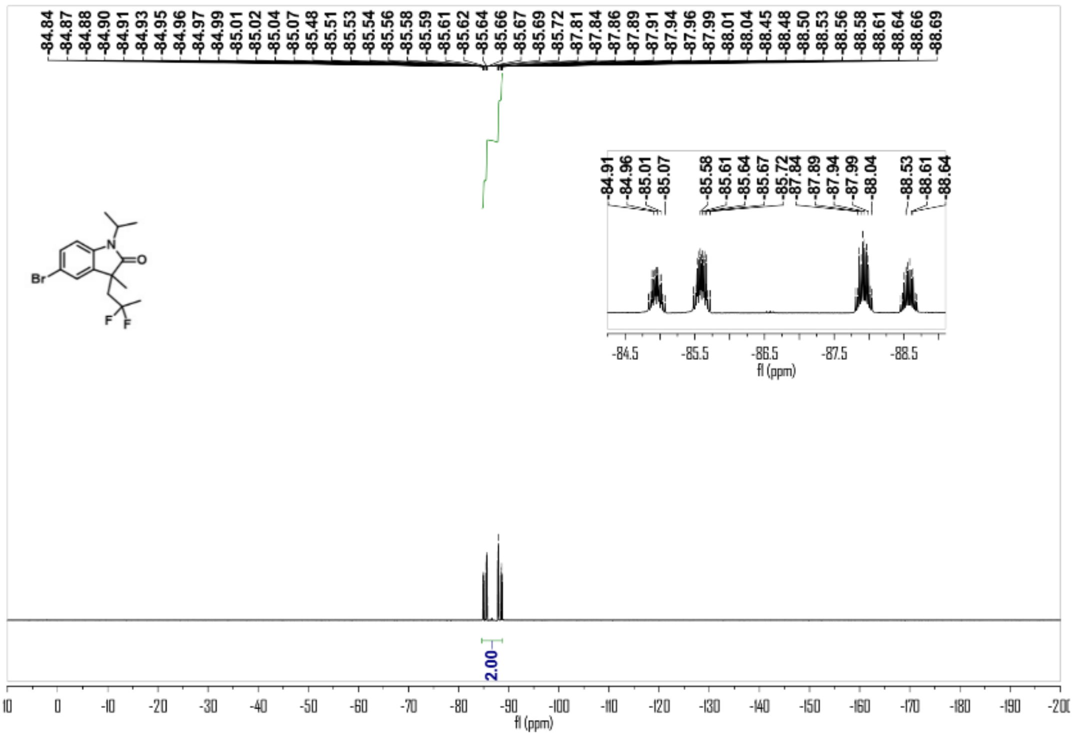

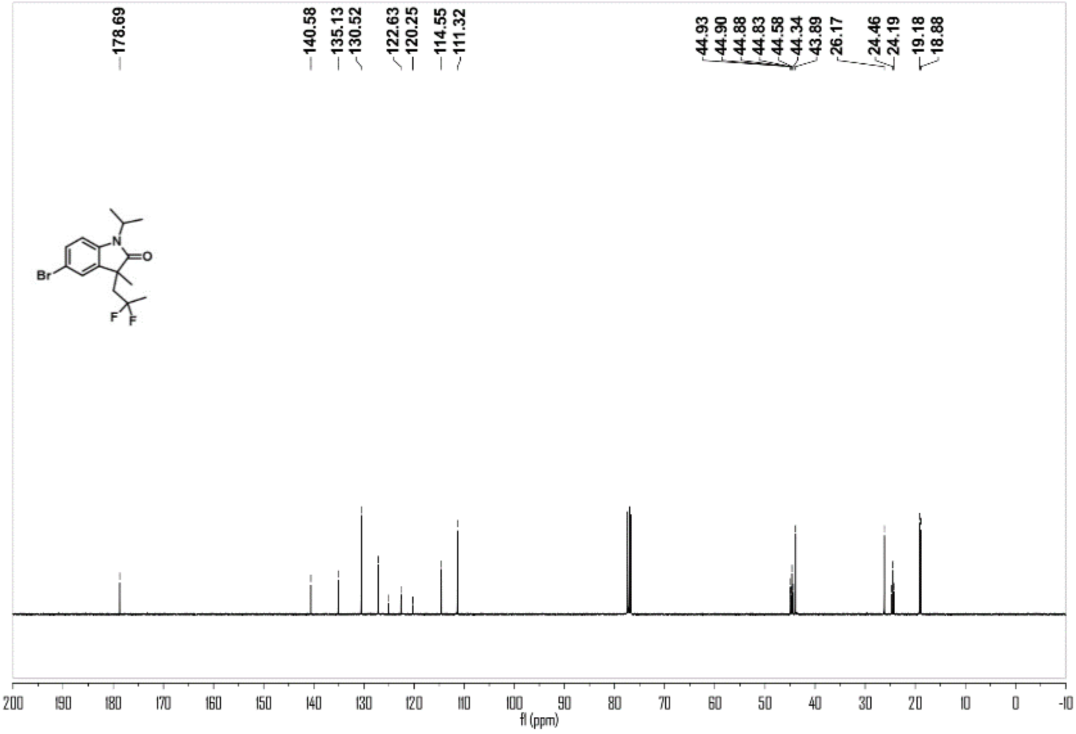

^{1}H, ^{19}F and ^{13}C NMR spectra of compound 5x

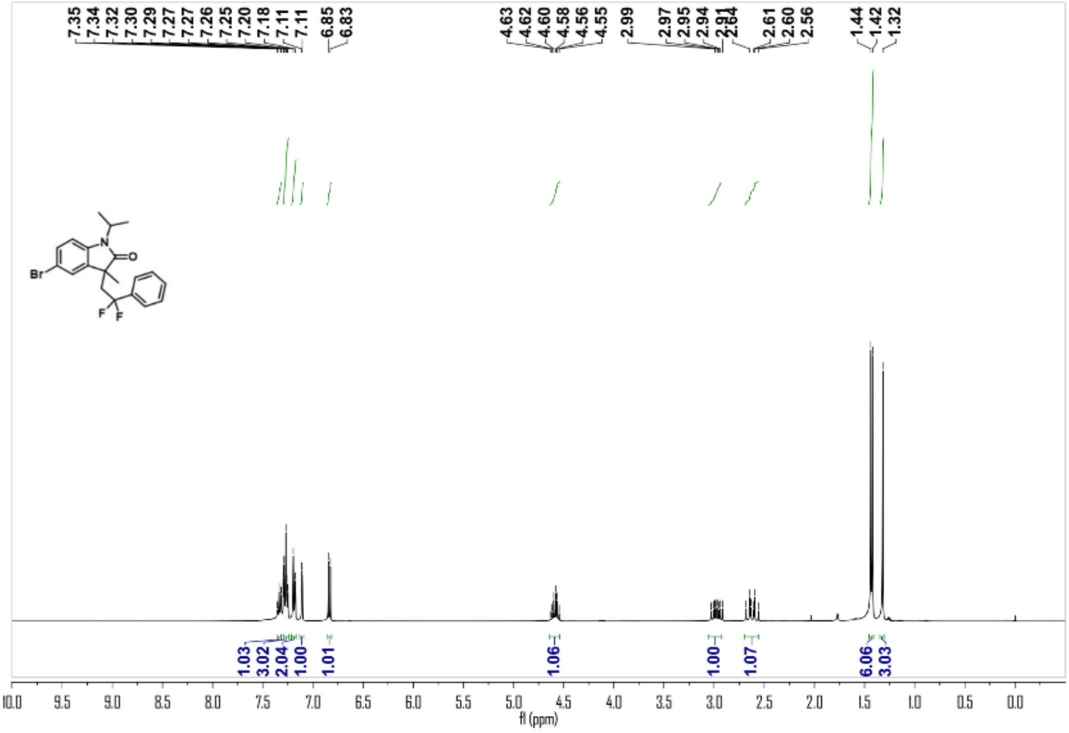

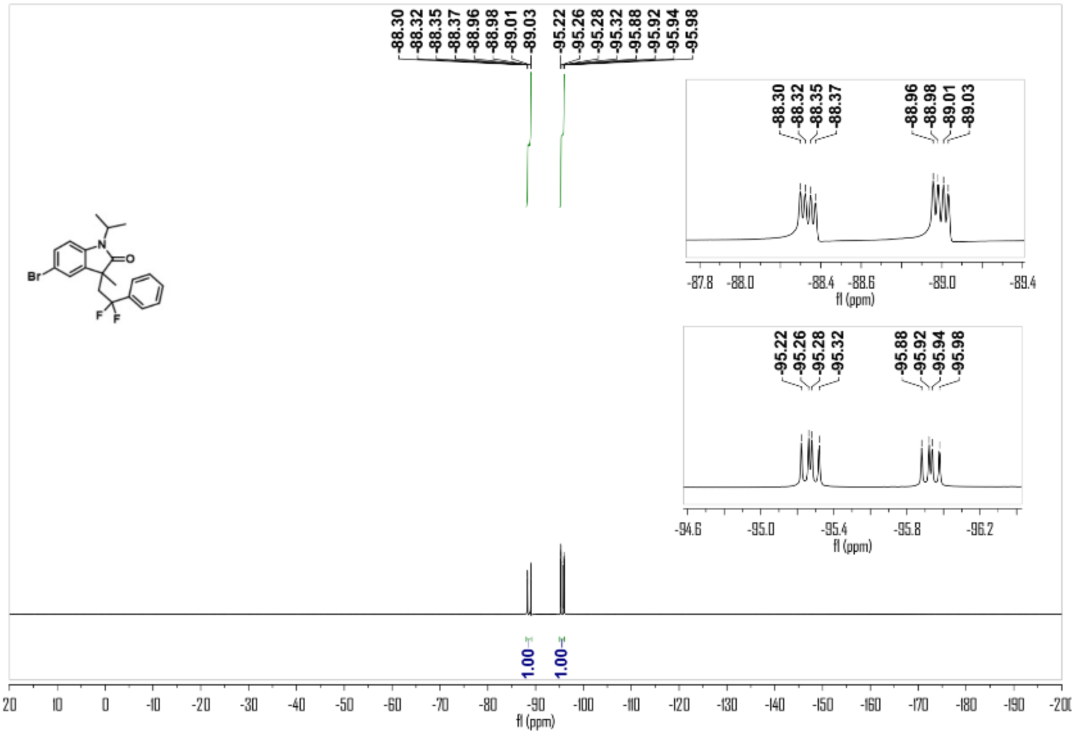
-88.30
-88.32
-88.35
-88.37
-88.96
-88.98
-89.01
-89.03
-95.22
-95.26
-95.28
-95.32
-95.88
-95.92
-95.94
-95.98
Br
N
O
F
F
1.00
1.00
f1 (ppm)

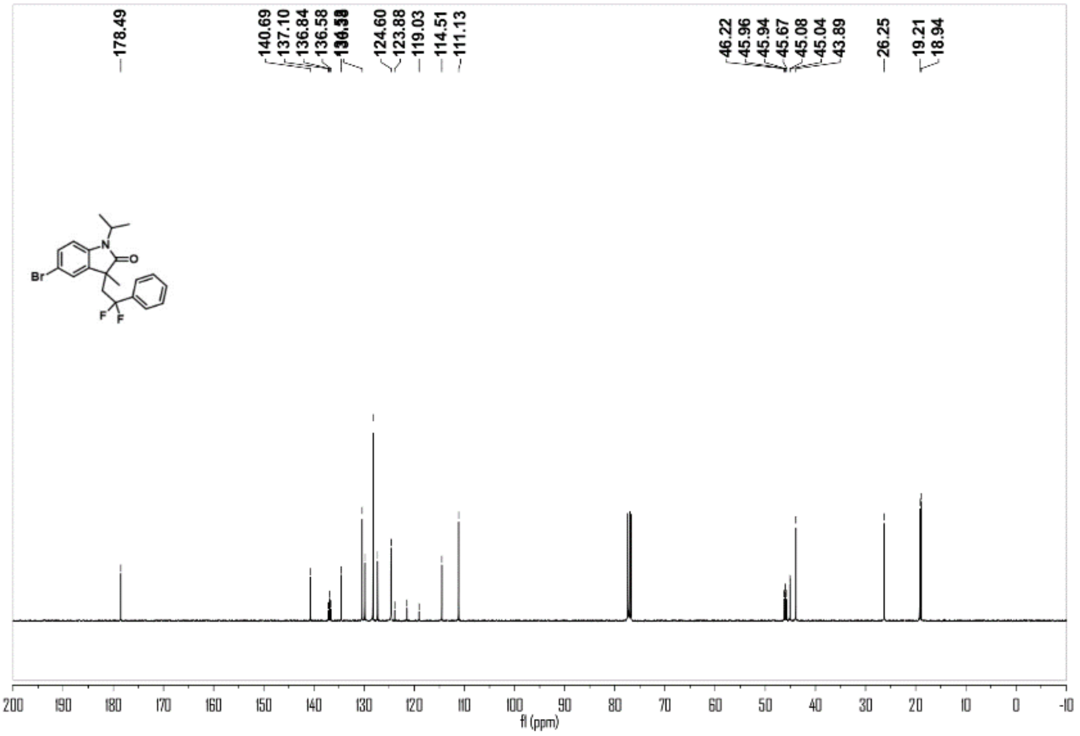
178.49
140.69
137.10
136.84
136.58
124.60
123.88
119.03
114.51
111.13
46.22
45.96
45.94
45.67
45.08
45.04
43.89
26.25
19.21
18.94
Br
N
O
F
F
f1 (ppm)

^{1}H, ^{19}F and ^{13}C NMR spectra of compound 5y

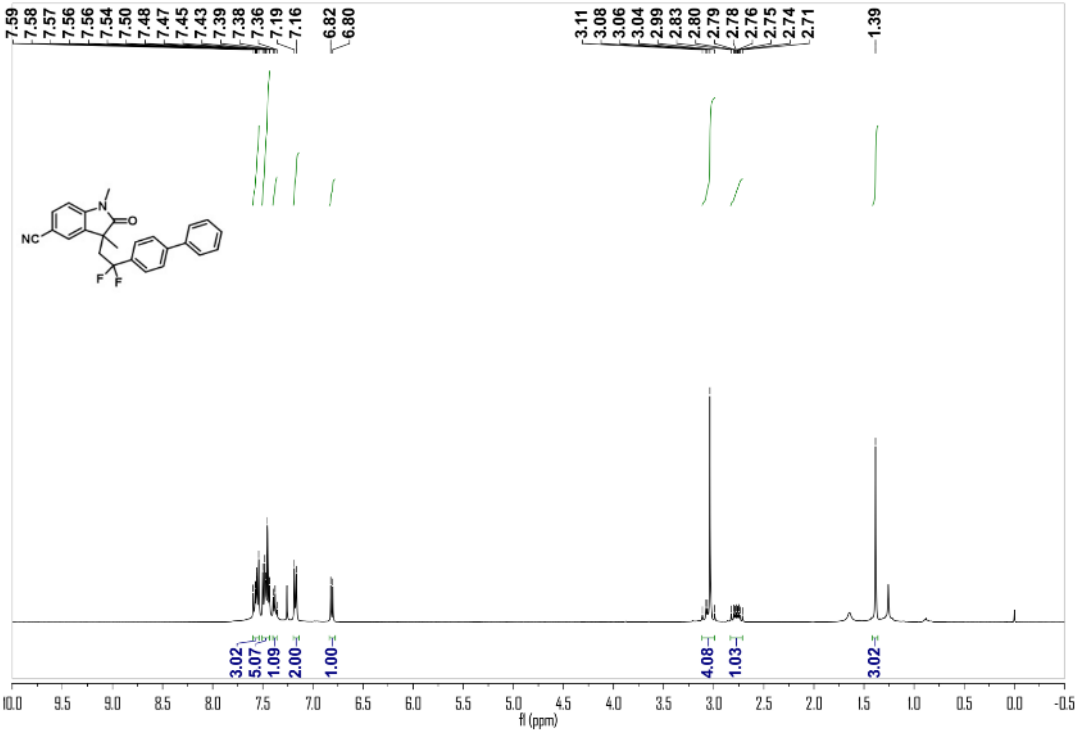

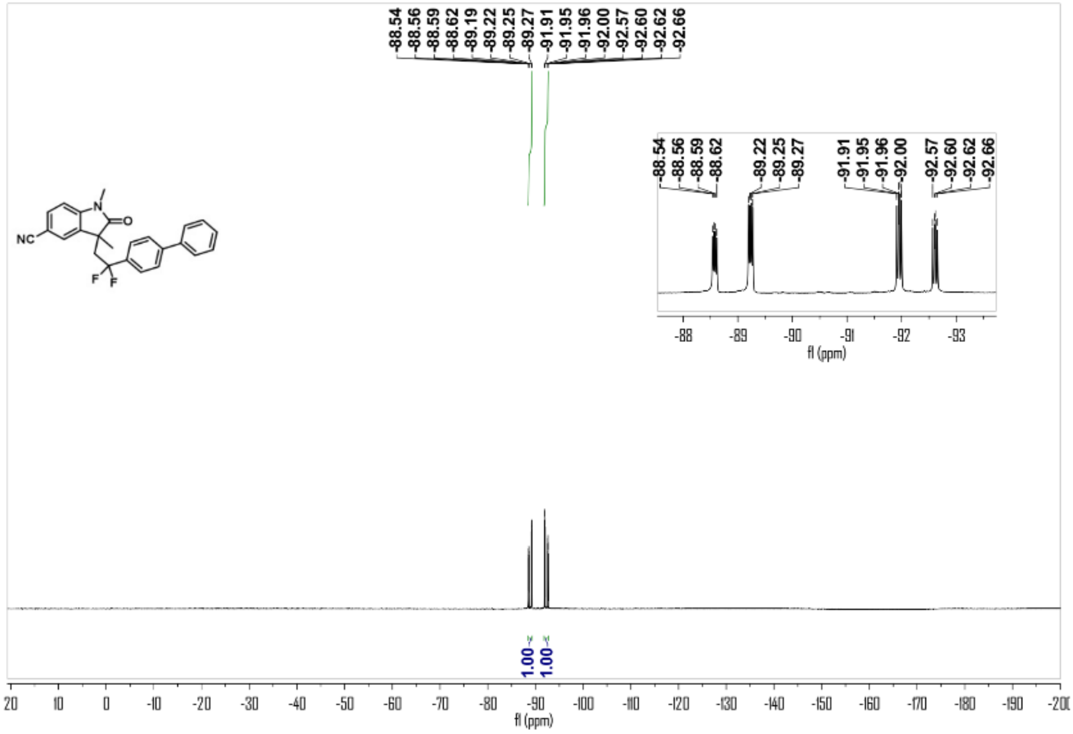

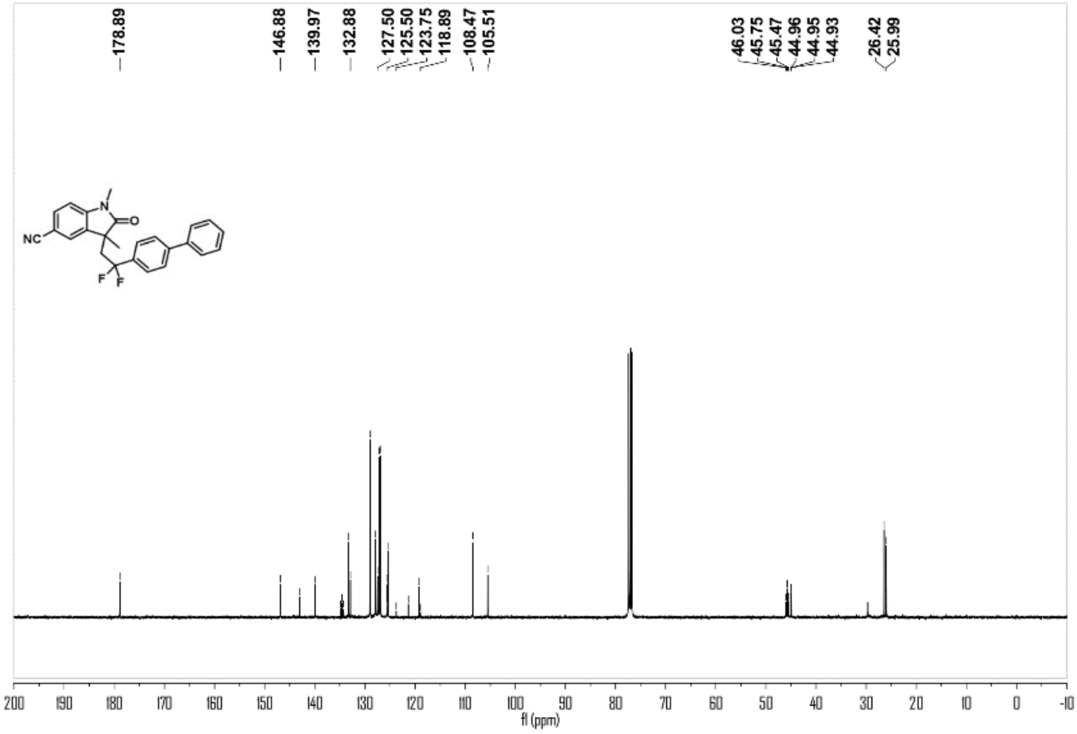

^{1}H, ^{19}F and ^{13}C NMR spectra of compound 5z

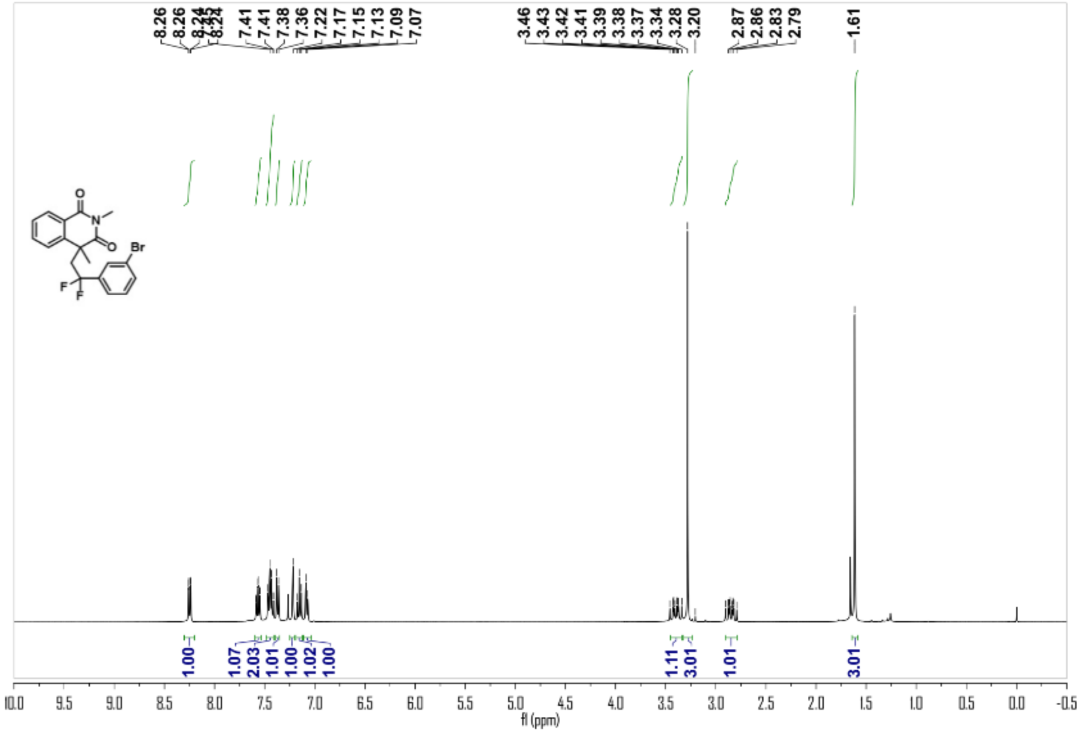

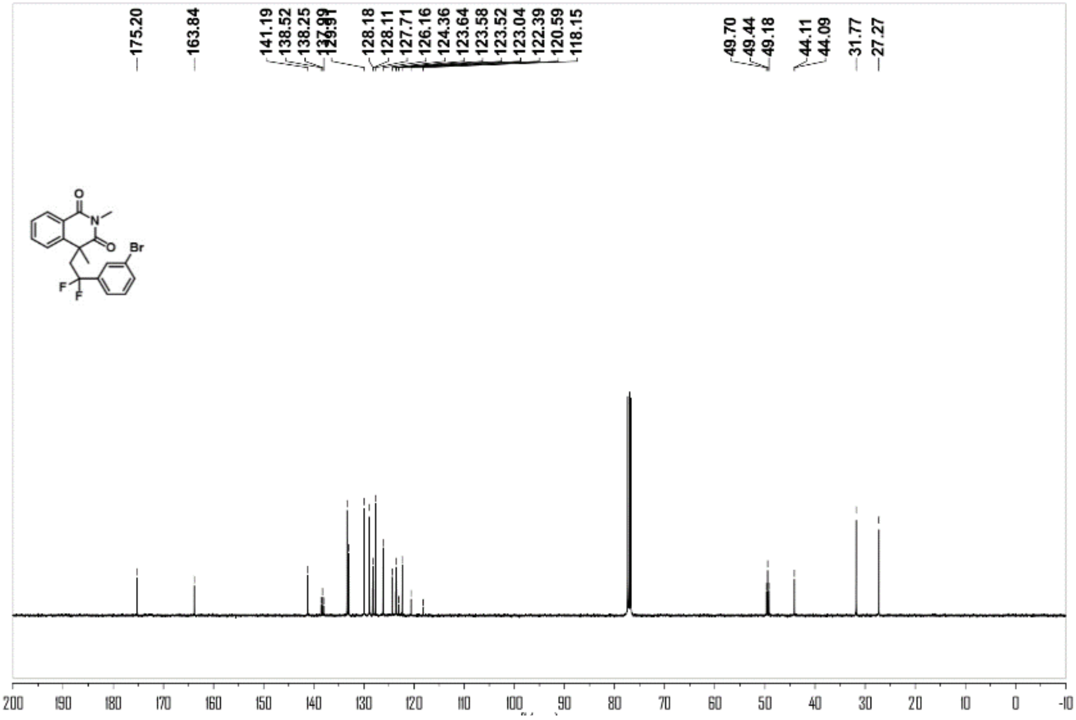
175.20
163.84
141.19
138.52
138.25
137.99
129.91
128.18
128.11
127.71
126.16
124.36
123.64
123.58
123.52
123.04
122.39
120.59
118.15
49.70
49.44
49.18
44.11
44.09
31.77
27.27
Br
F
F
N
O
O
200 190 180 170 160 150 140 130 120 110 100 90 80 70 60 50 40 30 20 10 0 -10

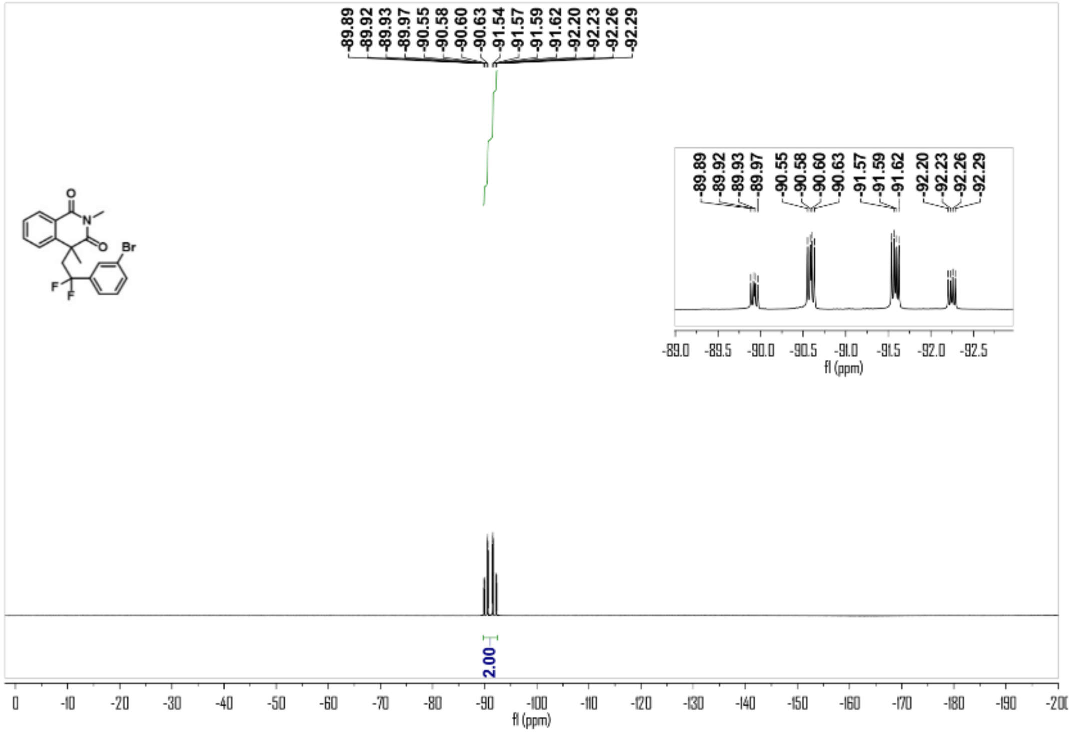
-89.89
-89.92
-89.93
-89.97
-90.55
-90.58
-90.60
-90.63
-91.54
-91.57
-91.59
-91.62
-92.20
-92.23
-92.26
-92.29
Br
F
F
N
O
O
-89.0 -89.5 -90.0 -90.5 -91.0 -91.5 -92.0 -92.5
f1 (ppm)
2.00
0 -10 -20 -30 -40 -50 -60 -70 -80 -90 -100 -110 -120 -130 -140 -150 -160 -170 -180 -190 -200
f1 (ppm)

^{1}H, ^{19}F and ^{13}C NMR spectra of compound 5aa

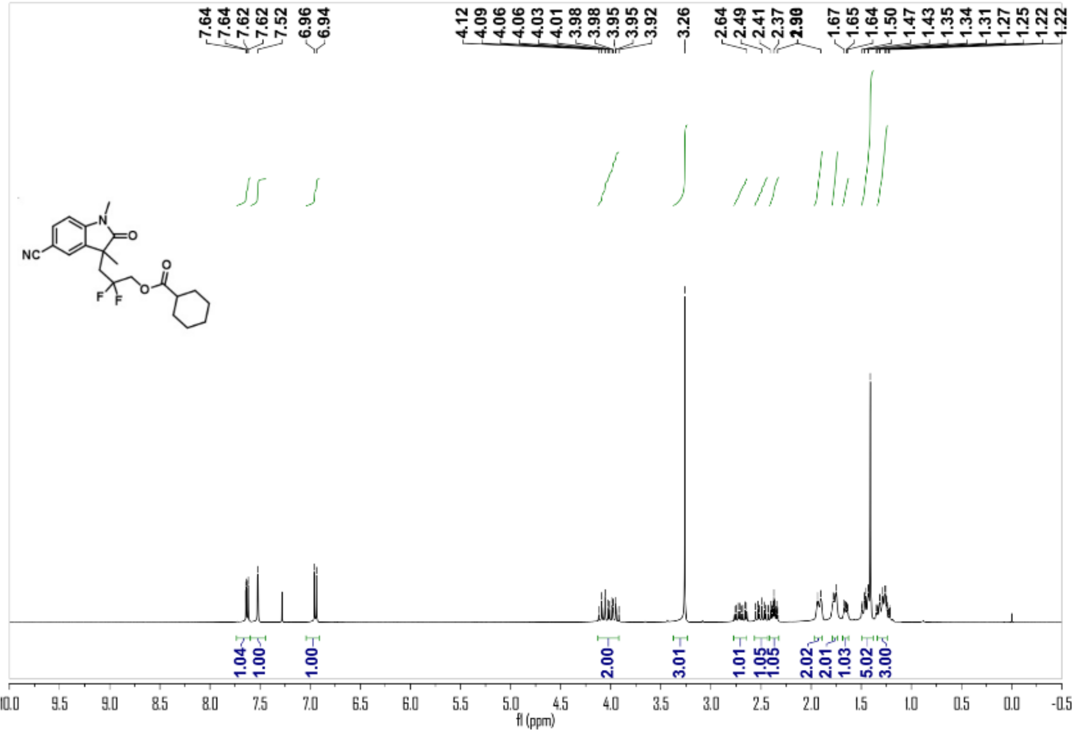

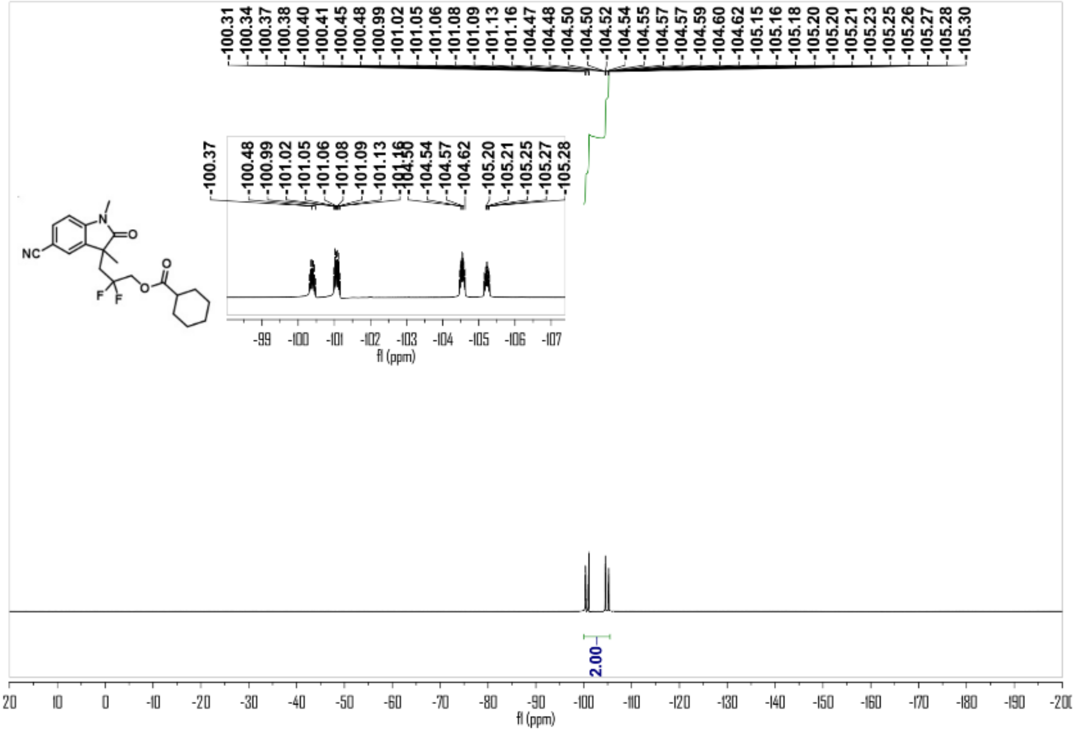

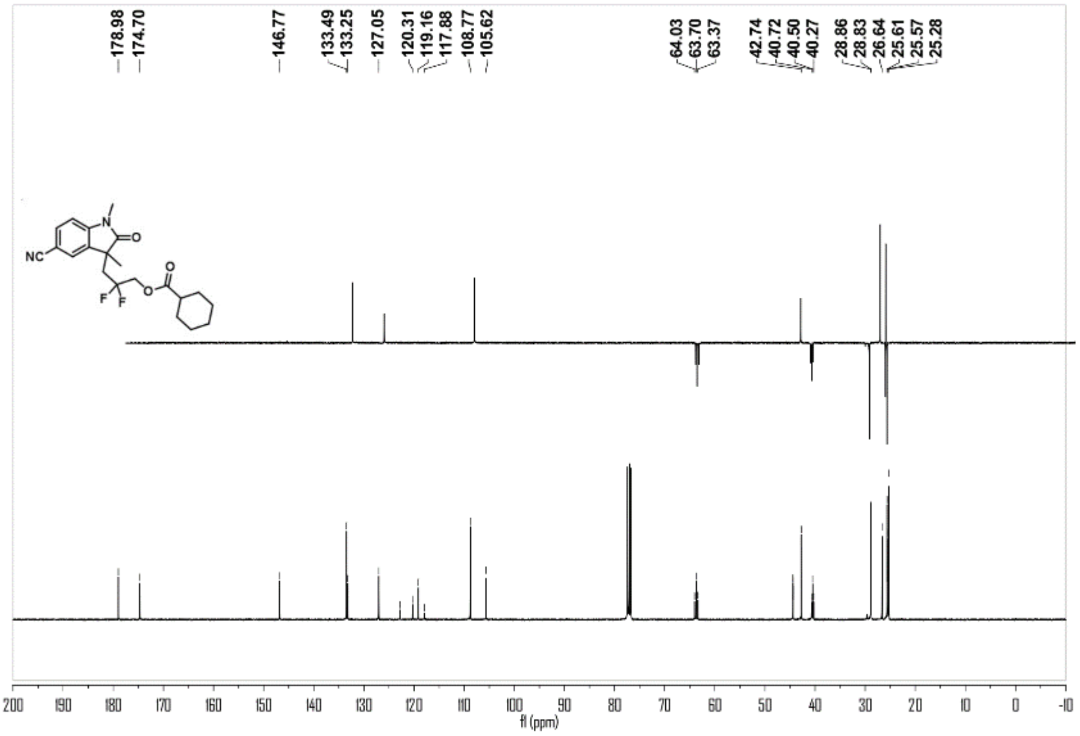

^{1}H, ^{19}F and ^{13}C NMR spectra of compound 5ab

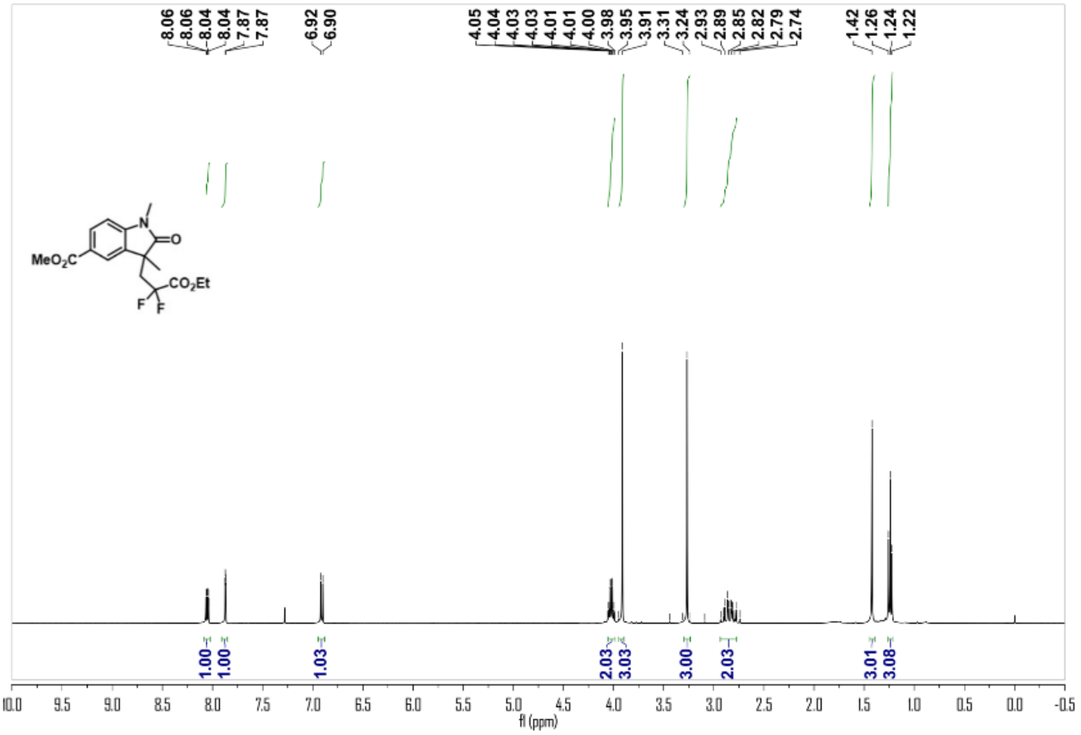

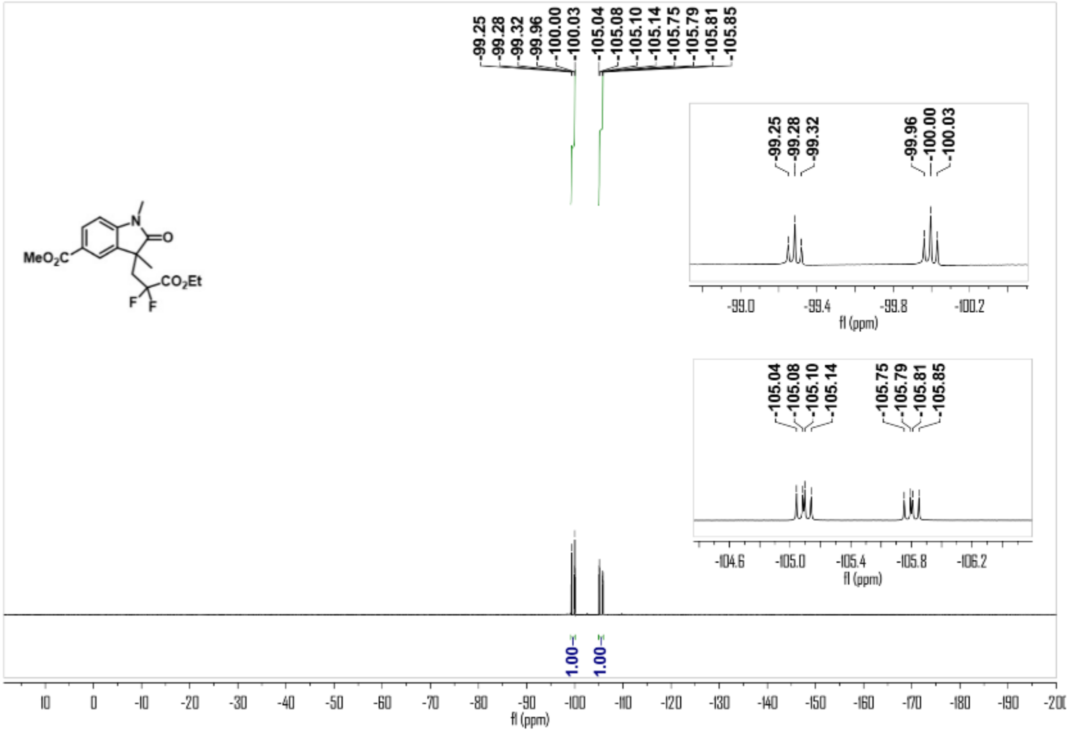

MeO2C
N
O
CO2Et
F
F
-99.25
-99.28
-99.32
-99.96
-100.00
-100.03
-105.04
-105.08
-105.10
-105.14
-105.75
-105.79
-105.81
-105.85
1.00
1.00
fl (ppm)

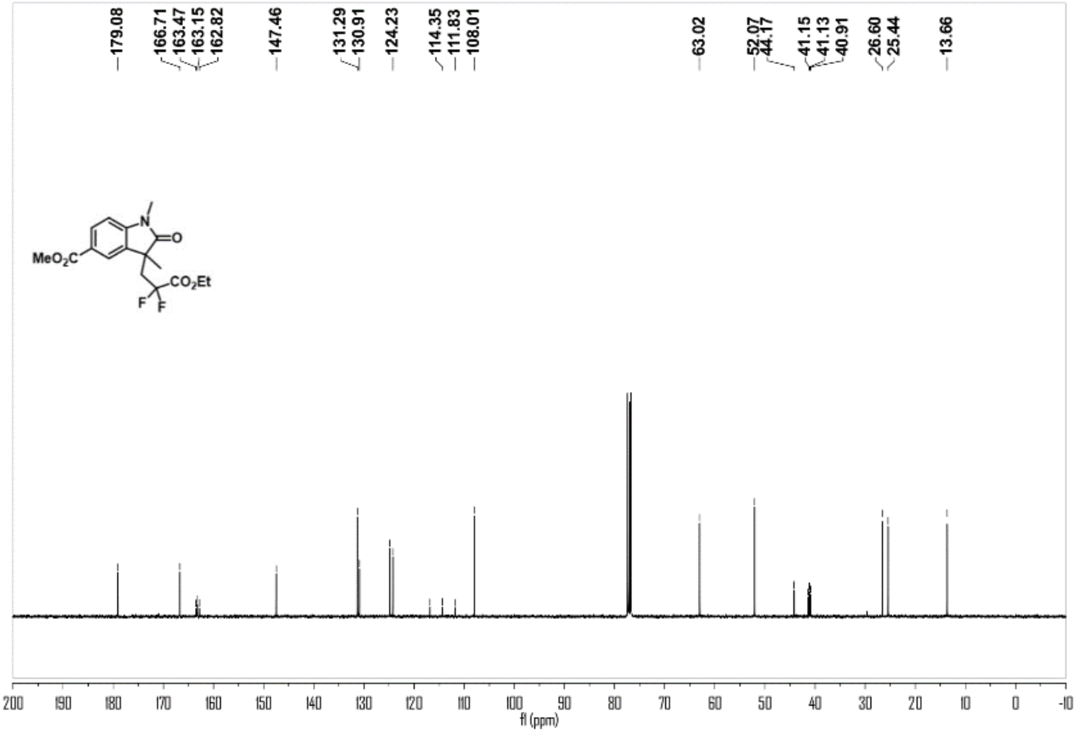

MeO2C
N
O
CO2Et
F
F
179.08
166.71
163.47
163.15
162.82
147.46
131.29
130.91
124.23
114.35
111.83
108.01
63.02
52.07
44.17
41.15
41.13
40.91
26.60
25.44
13.66
fl (ppm)

^{1}H, ^{19}F and ^{13}C NMR spectra of compound 5ac

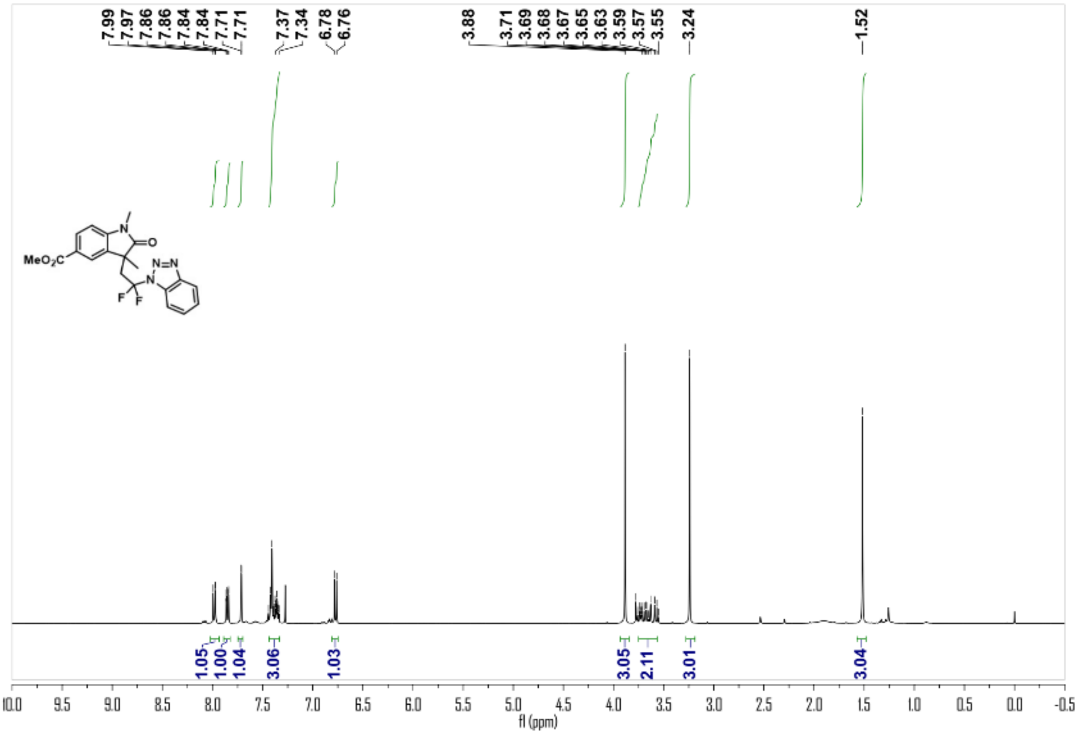

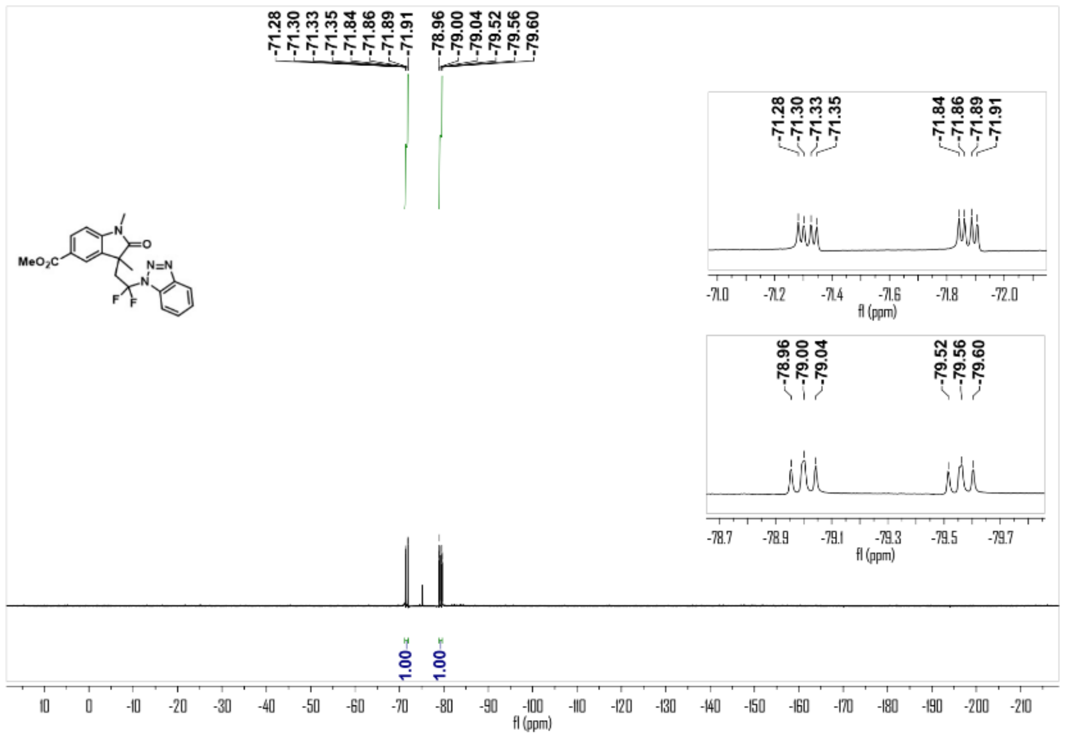

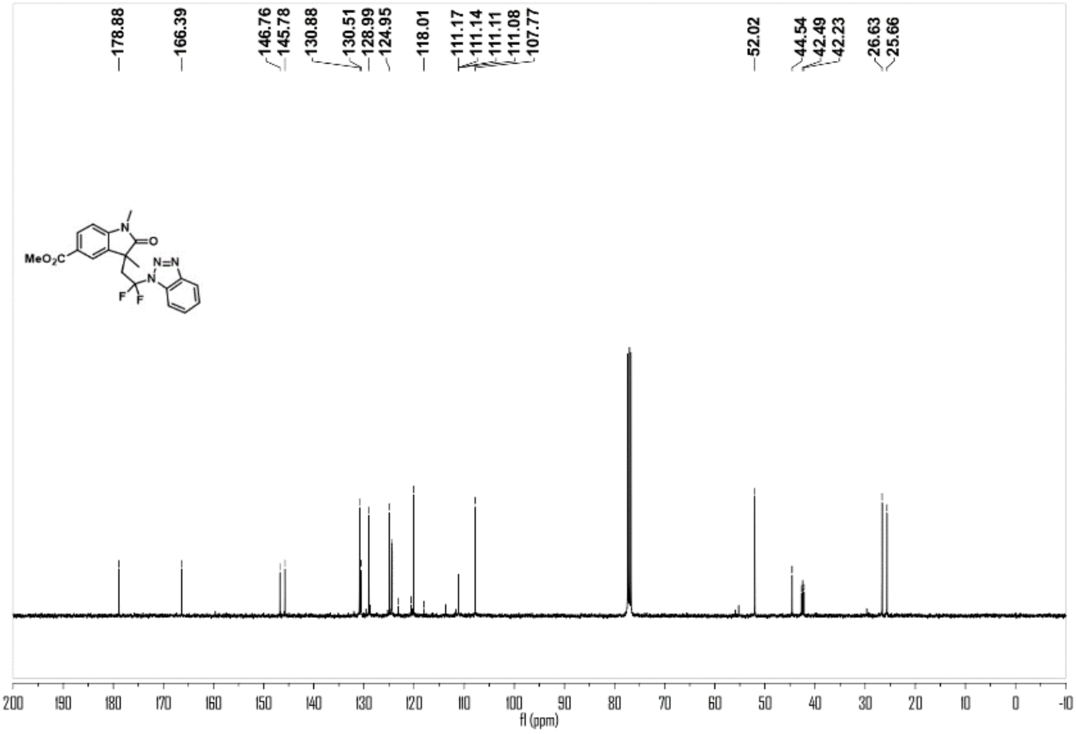

^{1}H, ^{19}F and ^{13}C NMR spectra of compound 5ad

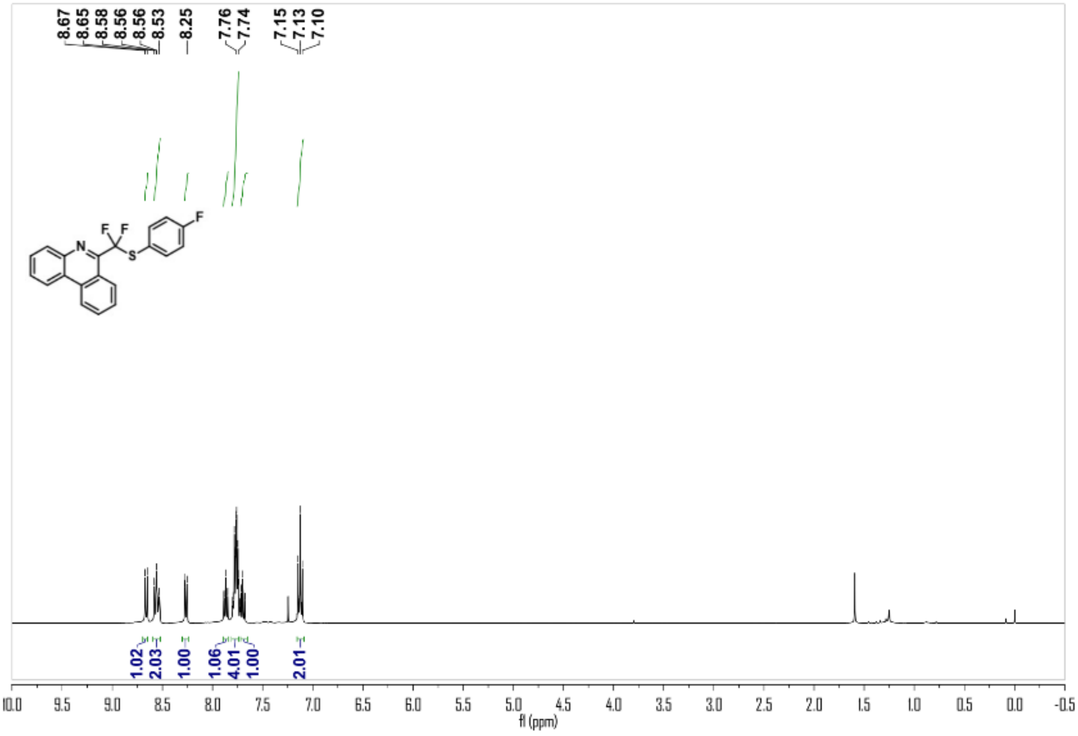

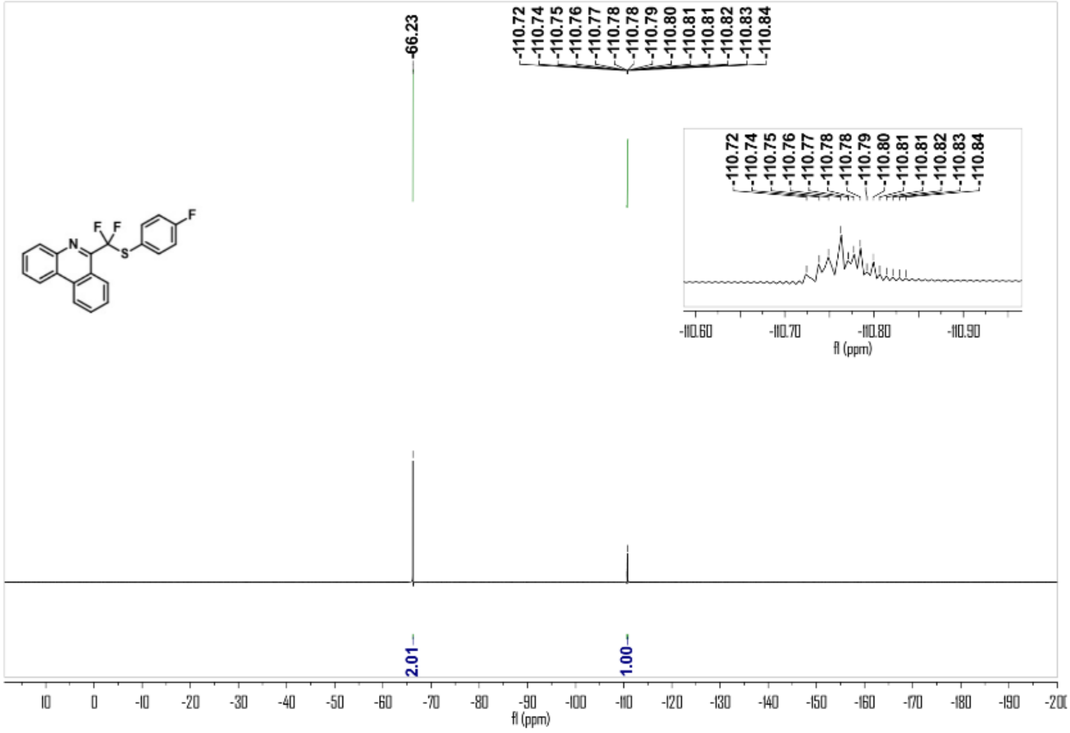
-66.23
-110.72
-110.74
-110.75
-110.76
-110.77
-110.78
-110.78
-110.79
-110.80
-110.81
-110.81
-110.82
-110.83
-110.84
2.01
1.00
f1 (ppm)

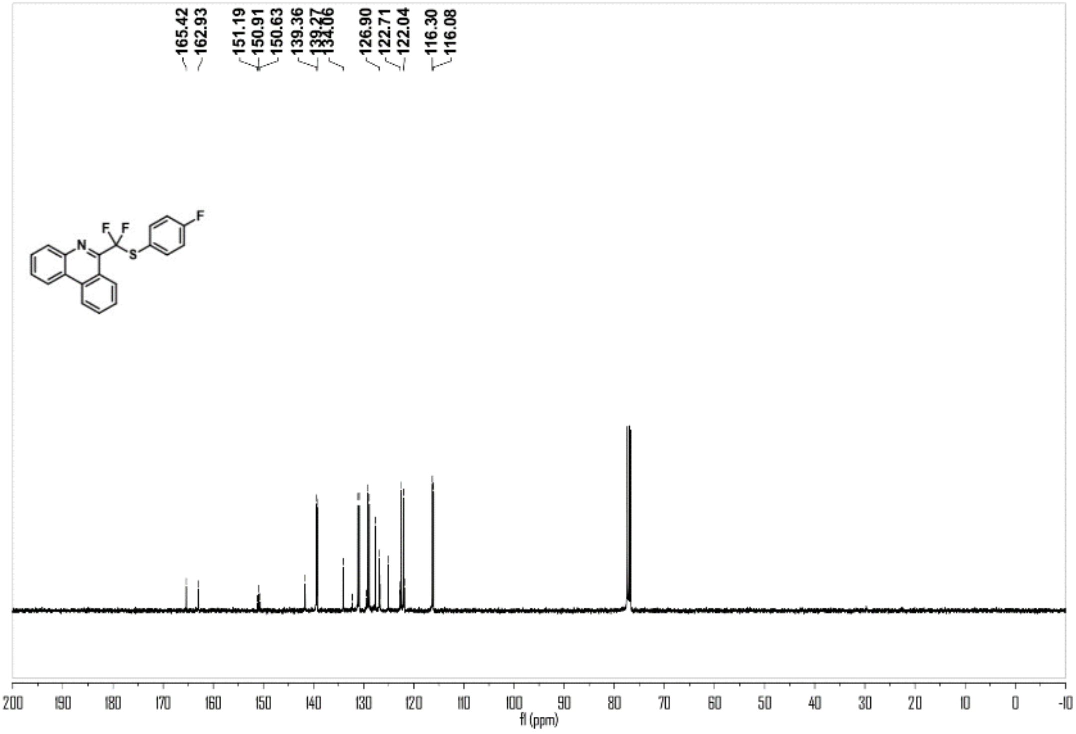
165.42
162.93
151.19
150.91
150.63
139.36
139.27
134.06
126.90
122.71
122.04
116.30
116.08
f1 (ppm)

^{1}H, ^{19}F and ^{13}C NMR spectra of compound 5ae

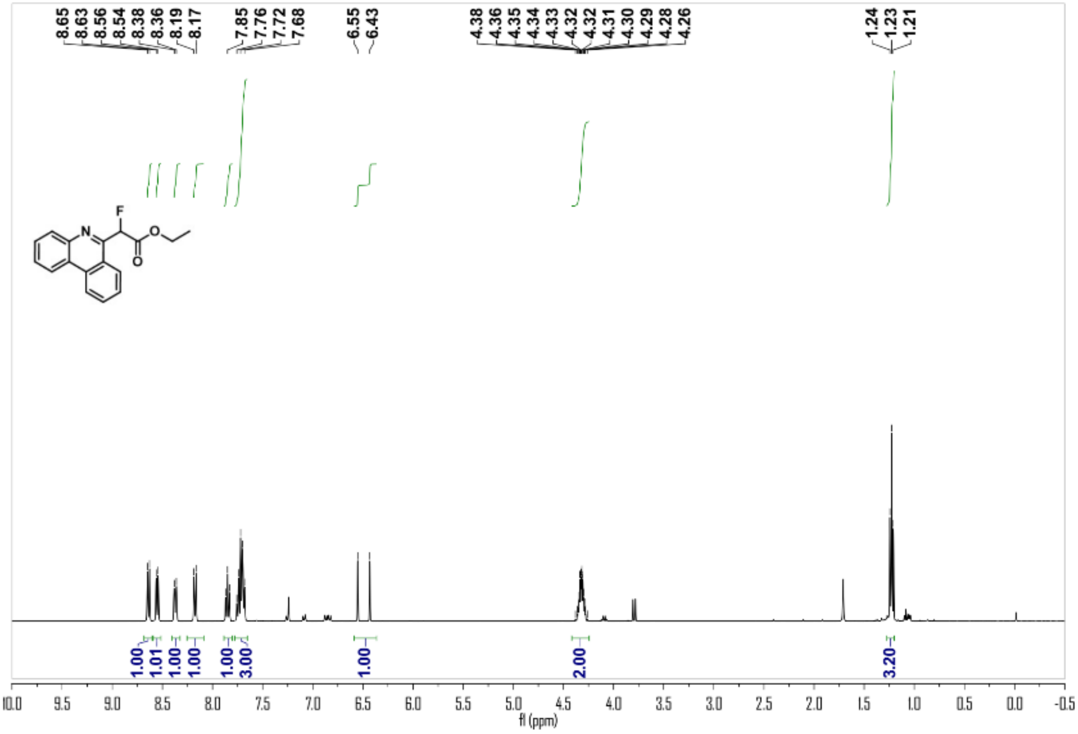

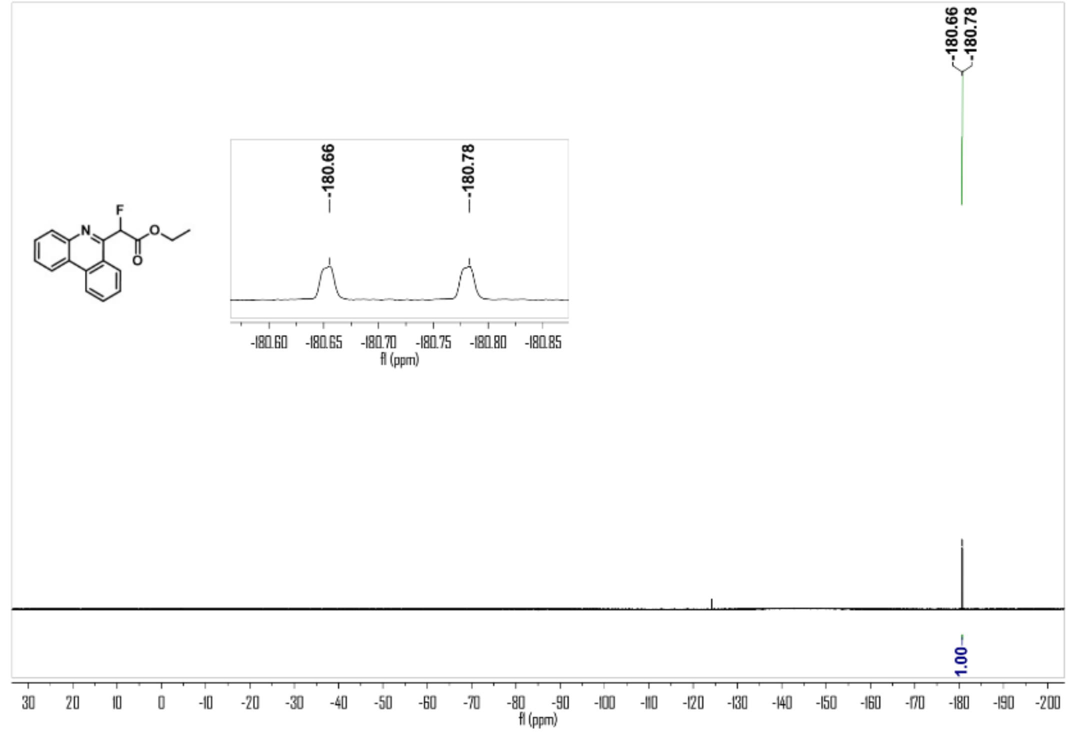

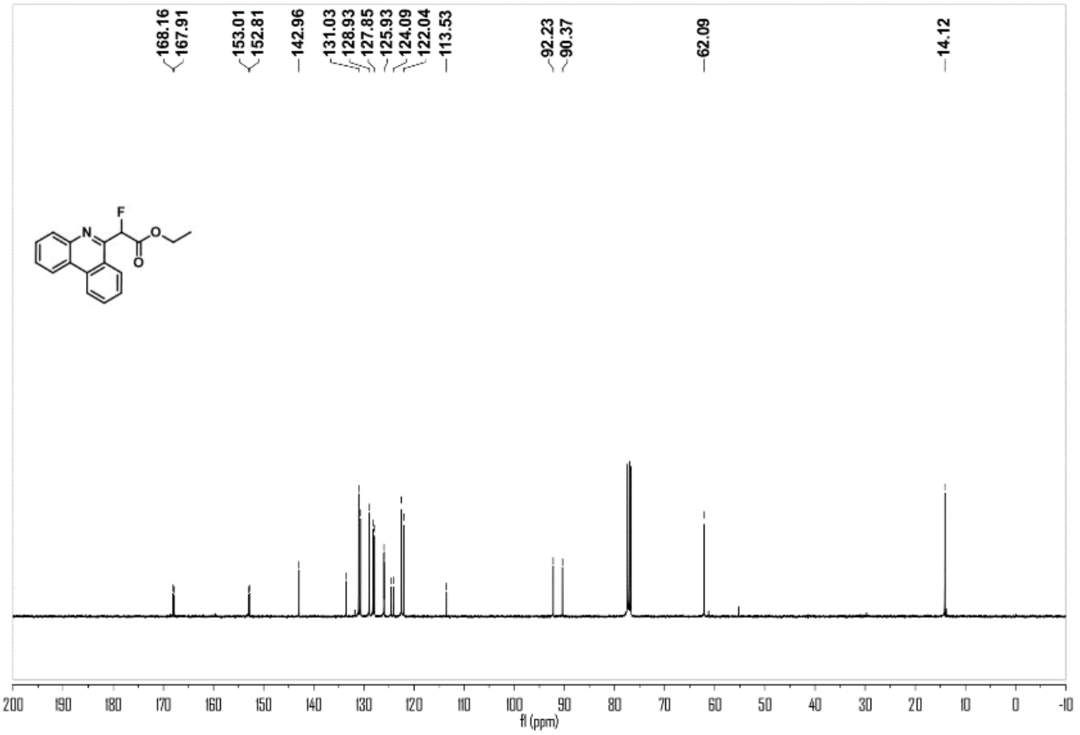

¹H, ¹⁹F and ¹³C NMR spectra of compound 5af

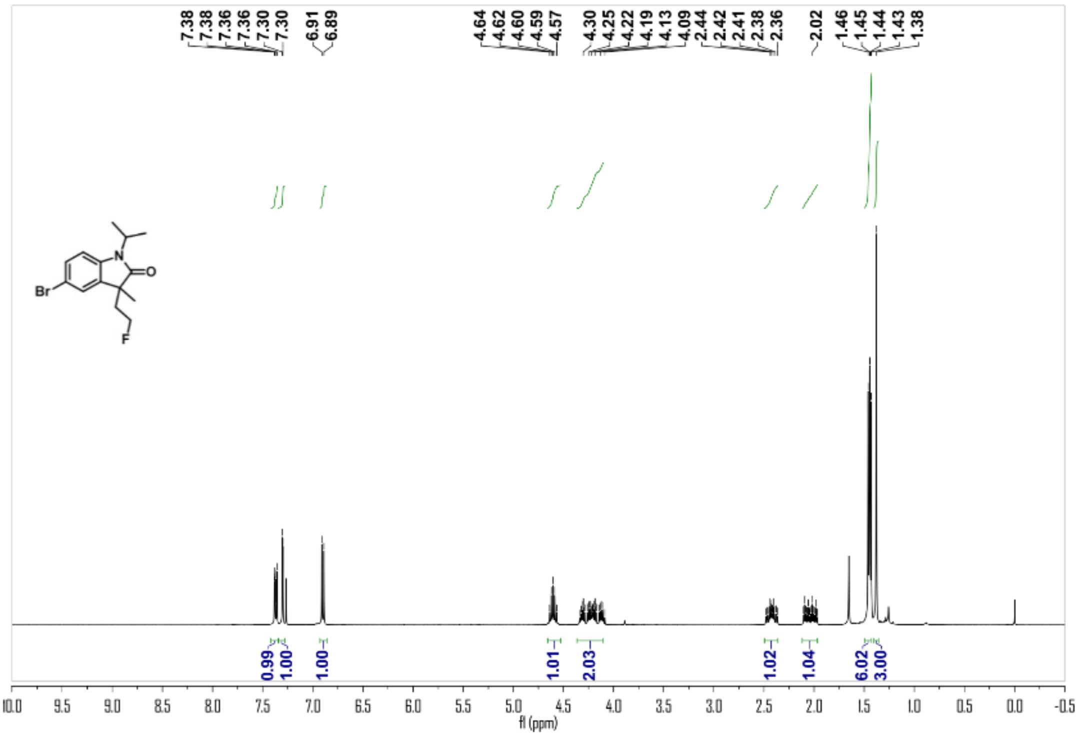

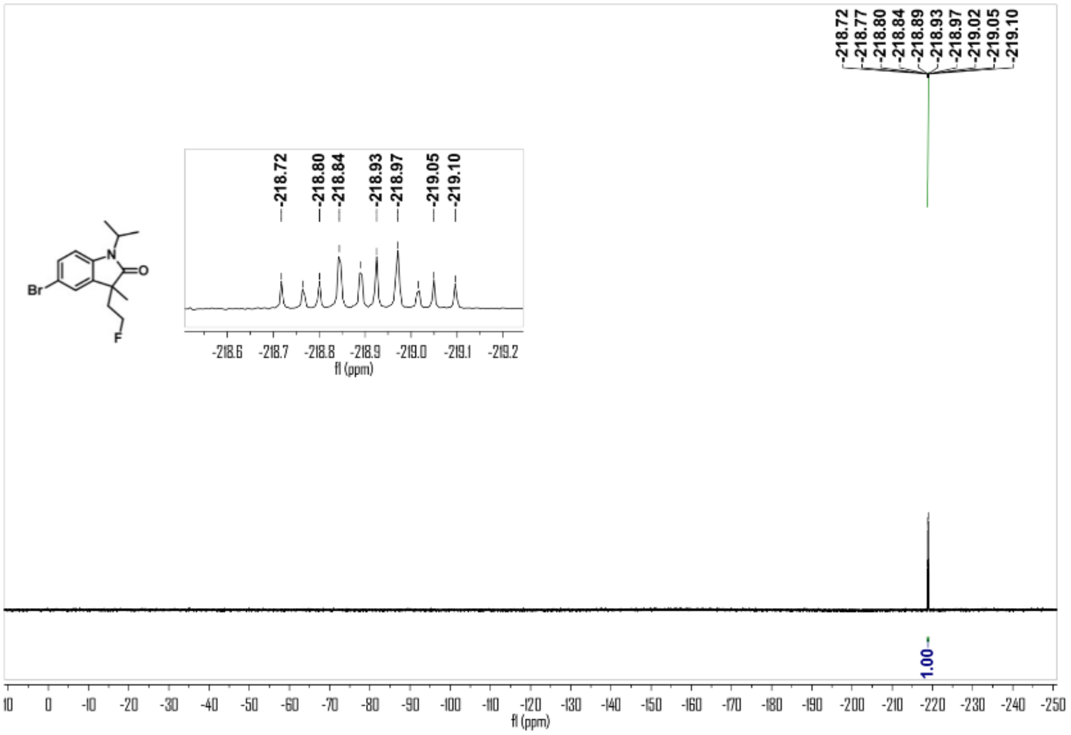

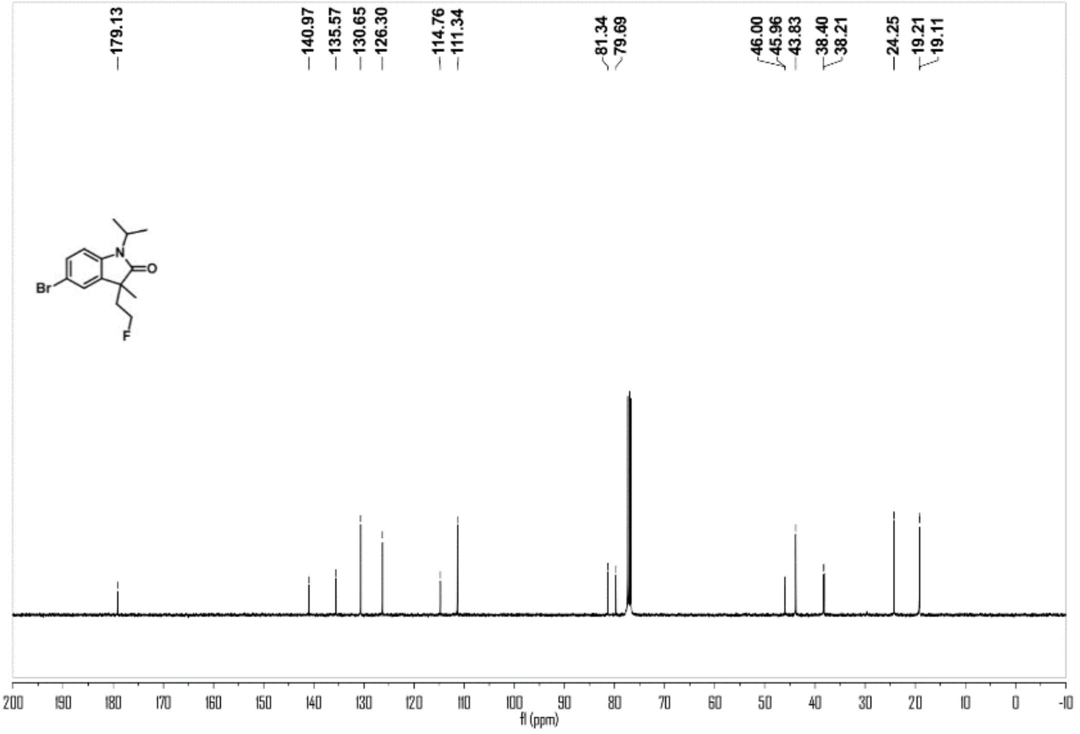

^{1}H, ^{19}F and ^{13}C NMR spectra of compound 5ag-1

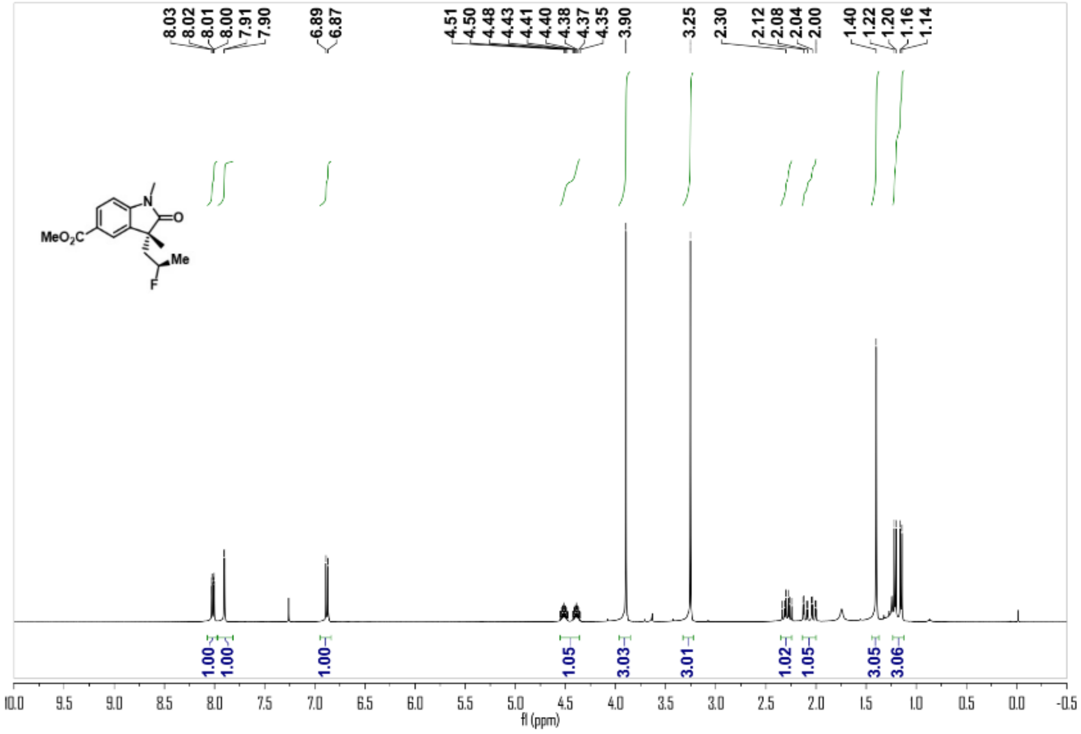

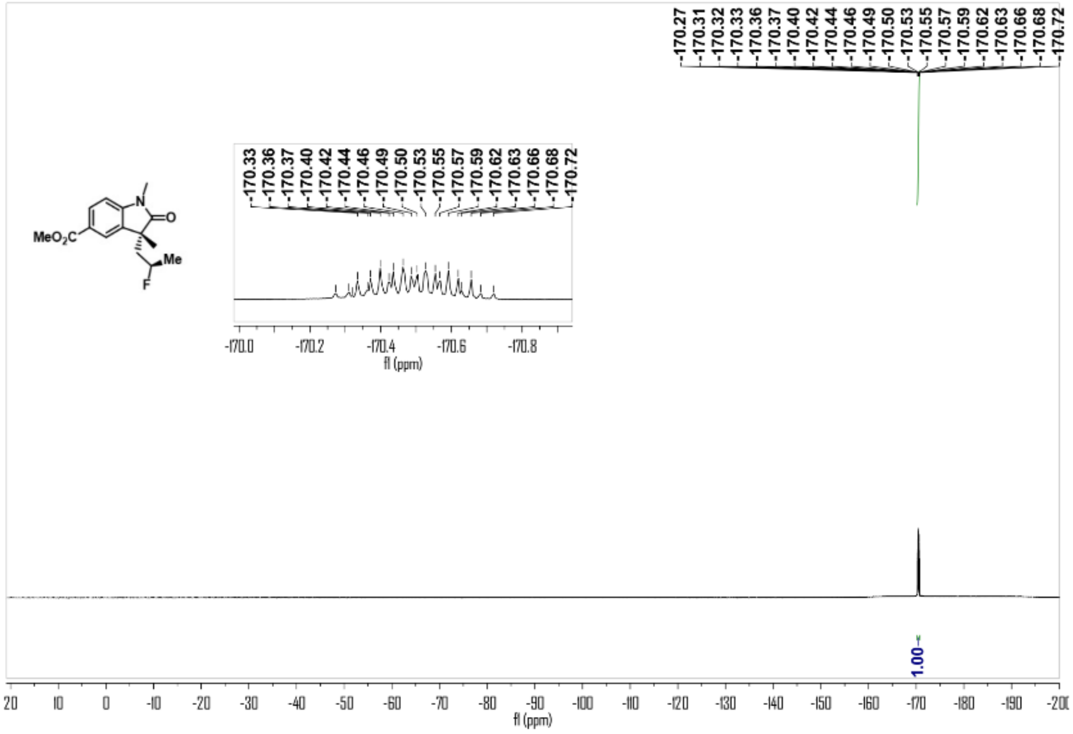

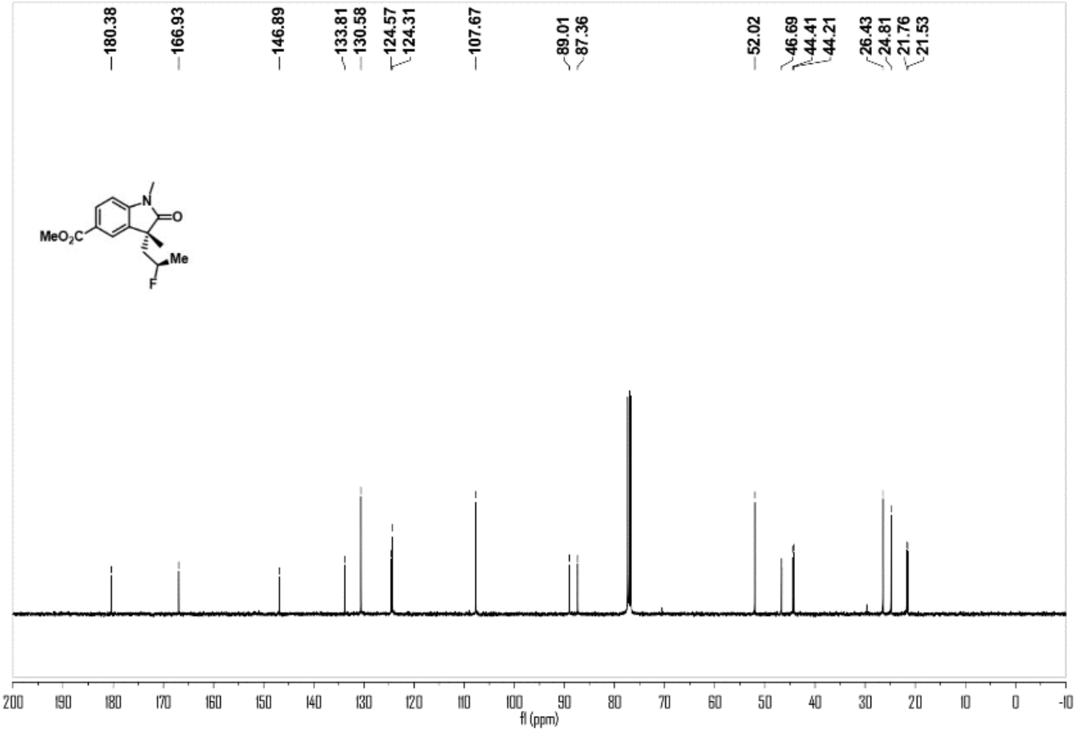

^{1}H, ^{19}F and ^{13}C NMR spectra of compound 5ag-2

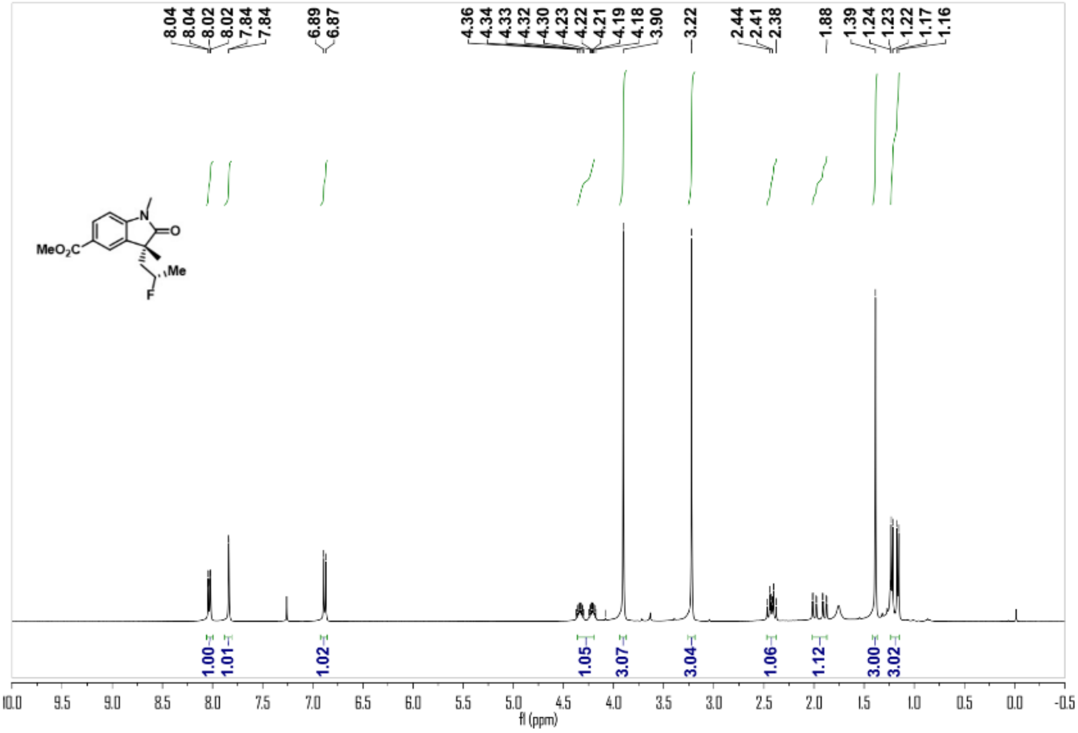

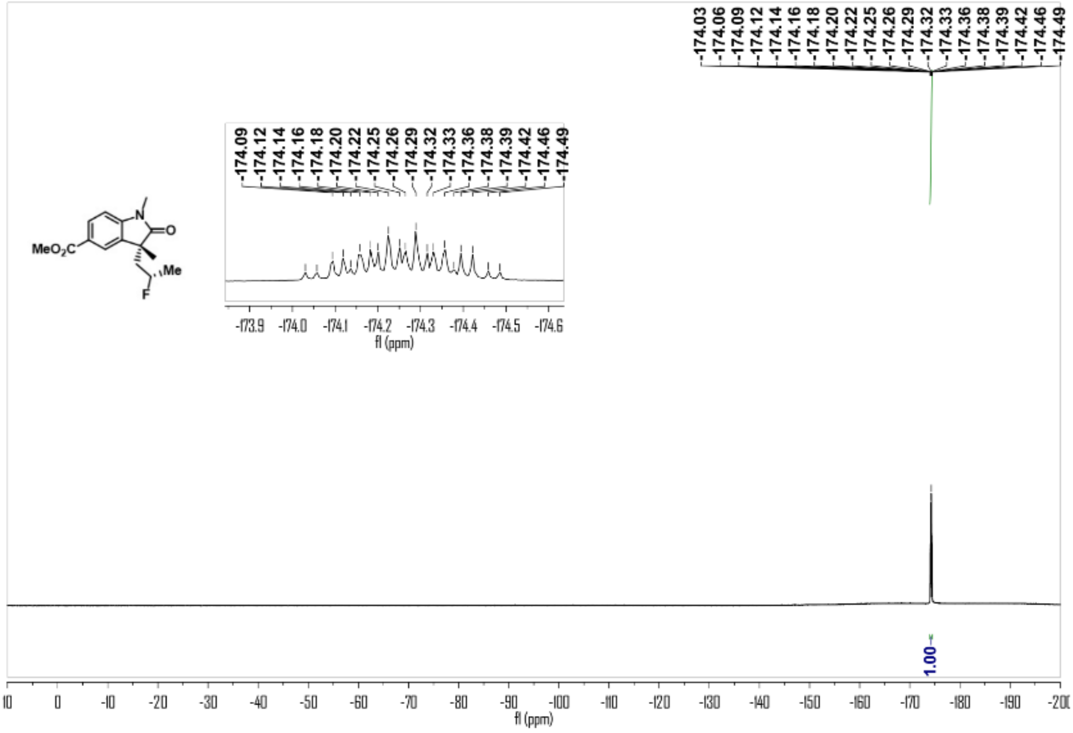

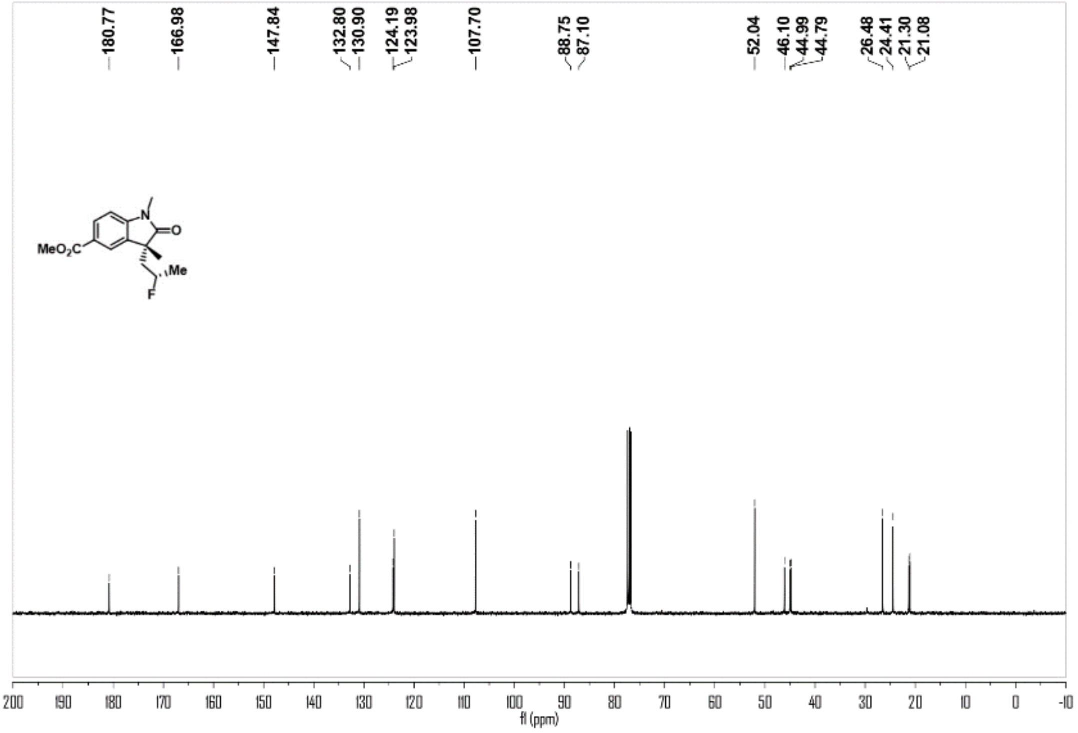

^{1}H, ^{19}F and ^{13}C NMR spectra of compound 5ah

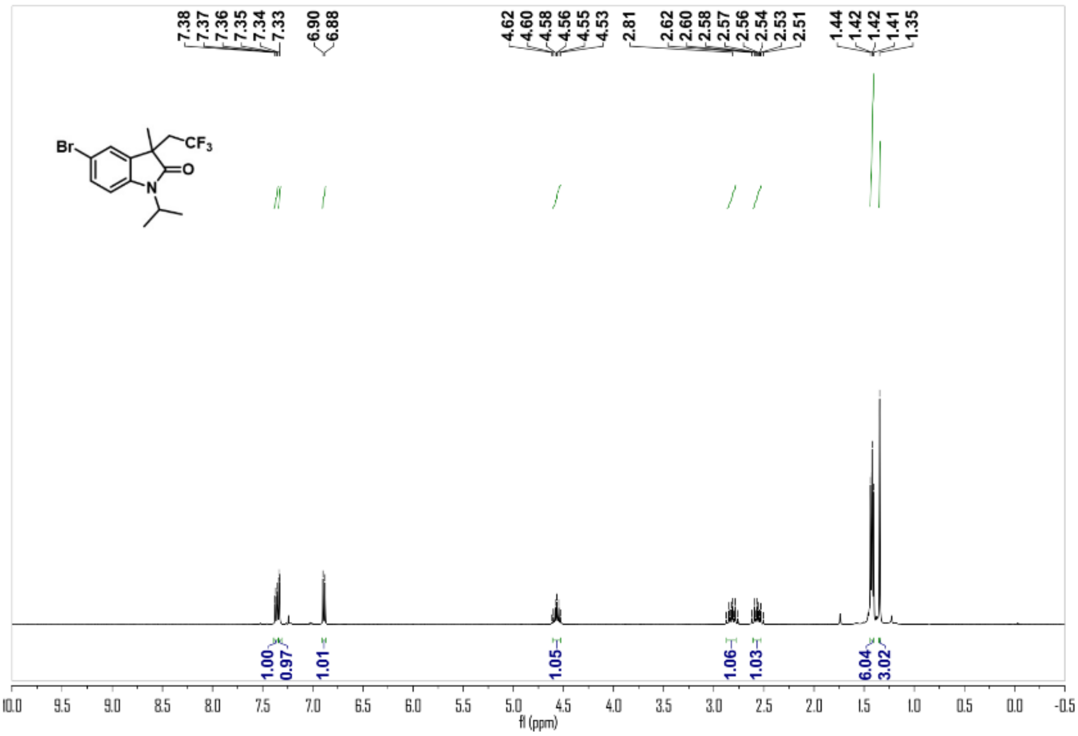

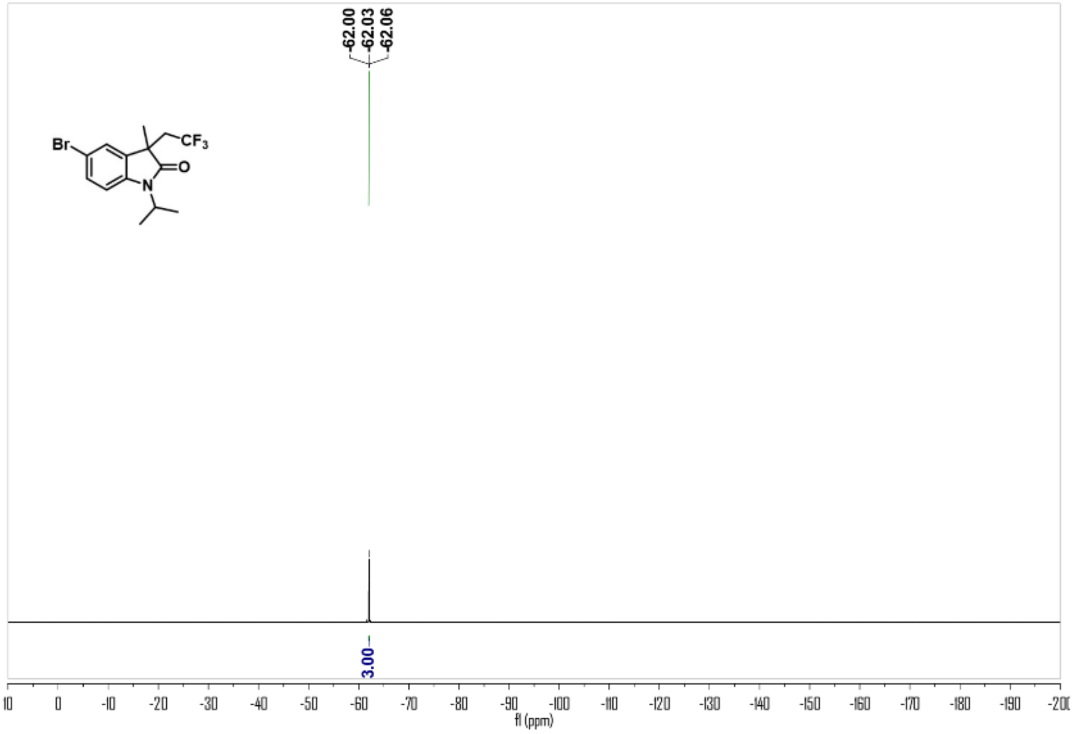

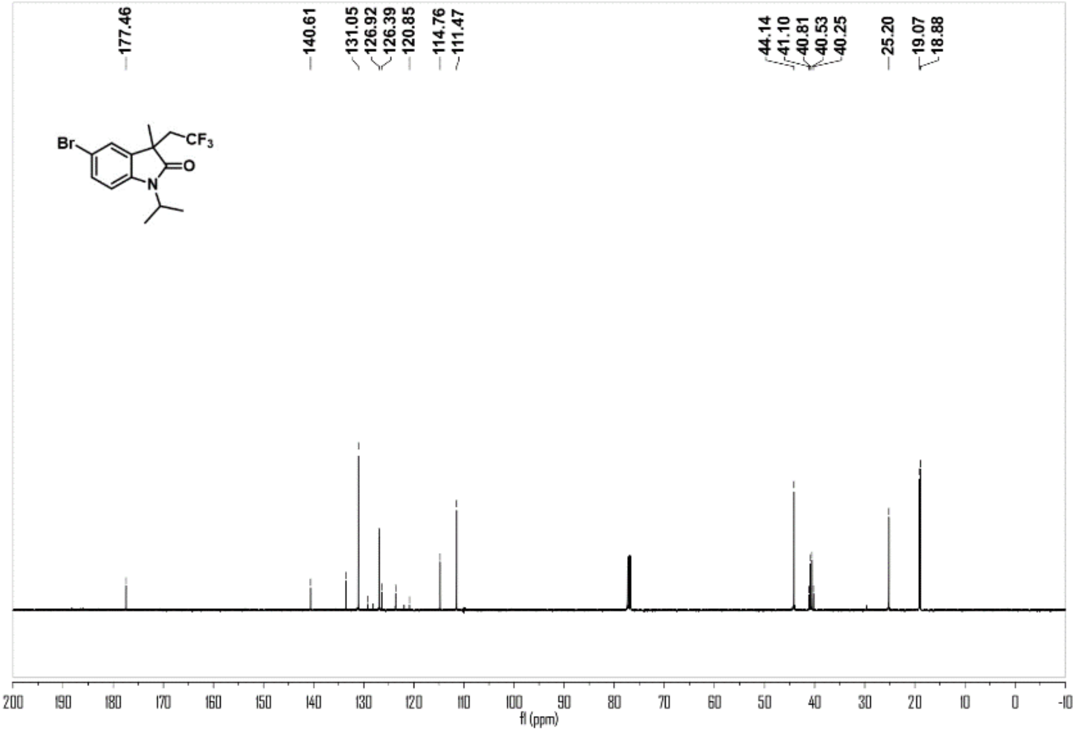

^{1}H, ^{19}F and ^{13}C NMR spectra of compound 5ai

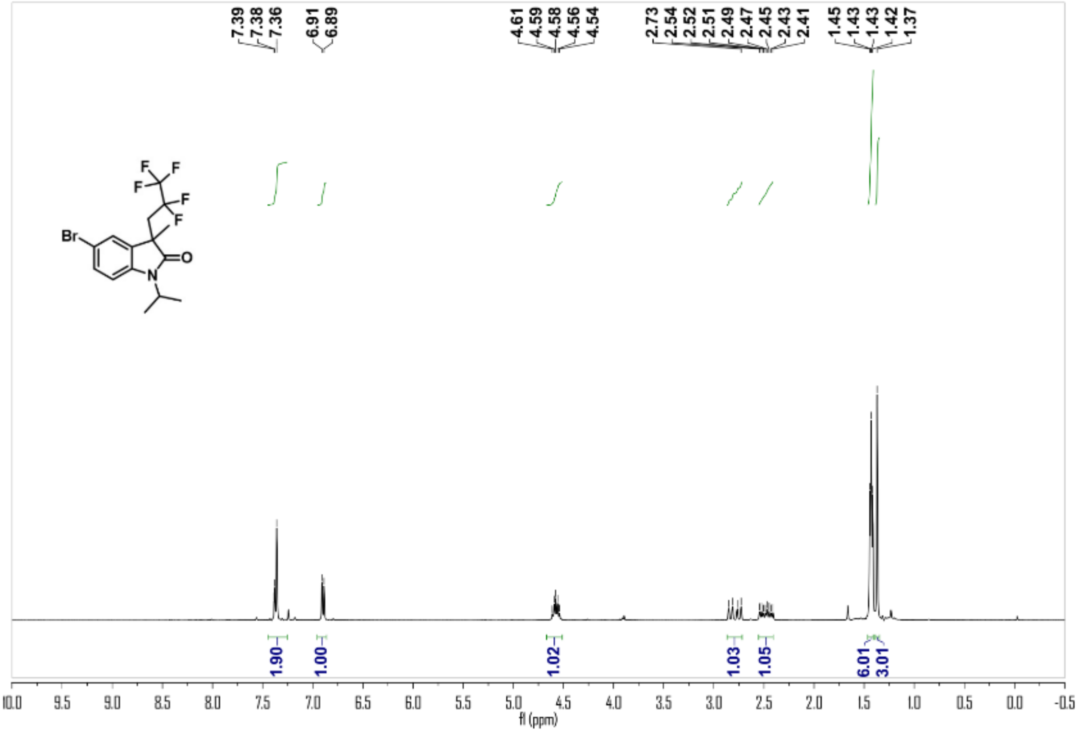

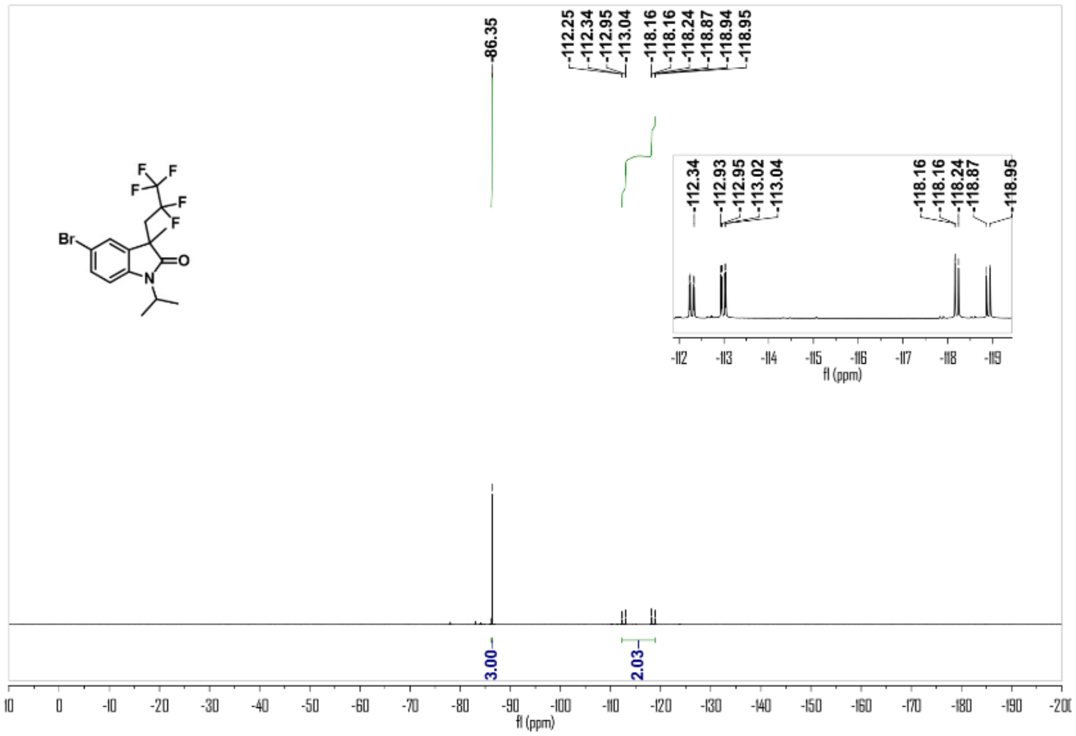

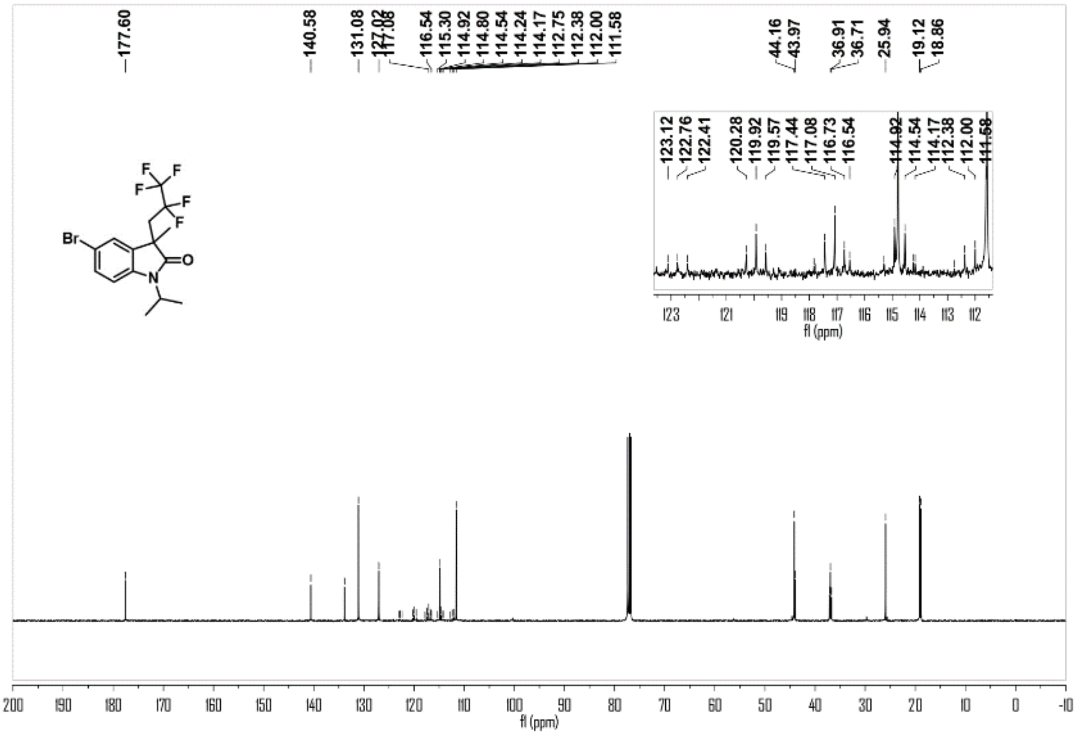

^{1}H, ^{19}F and ^{13}C NMR spectra of compound 5aj

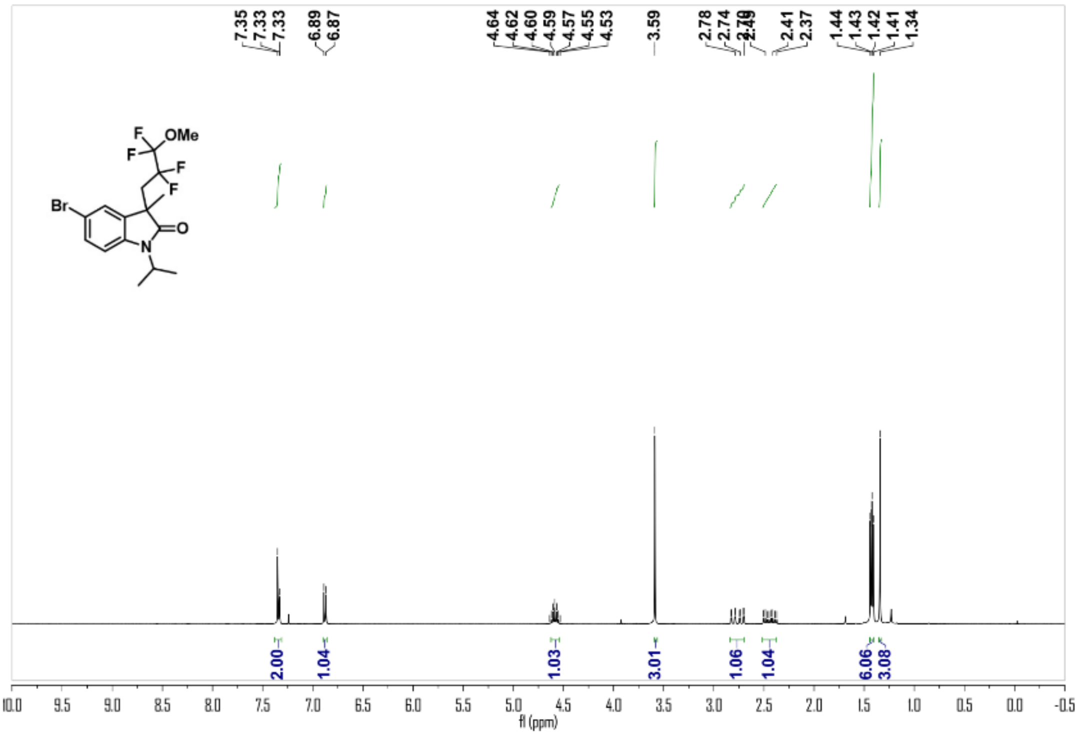

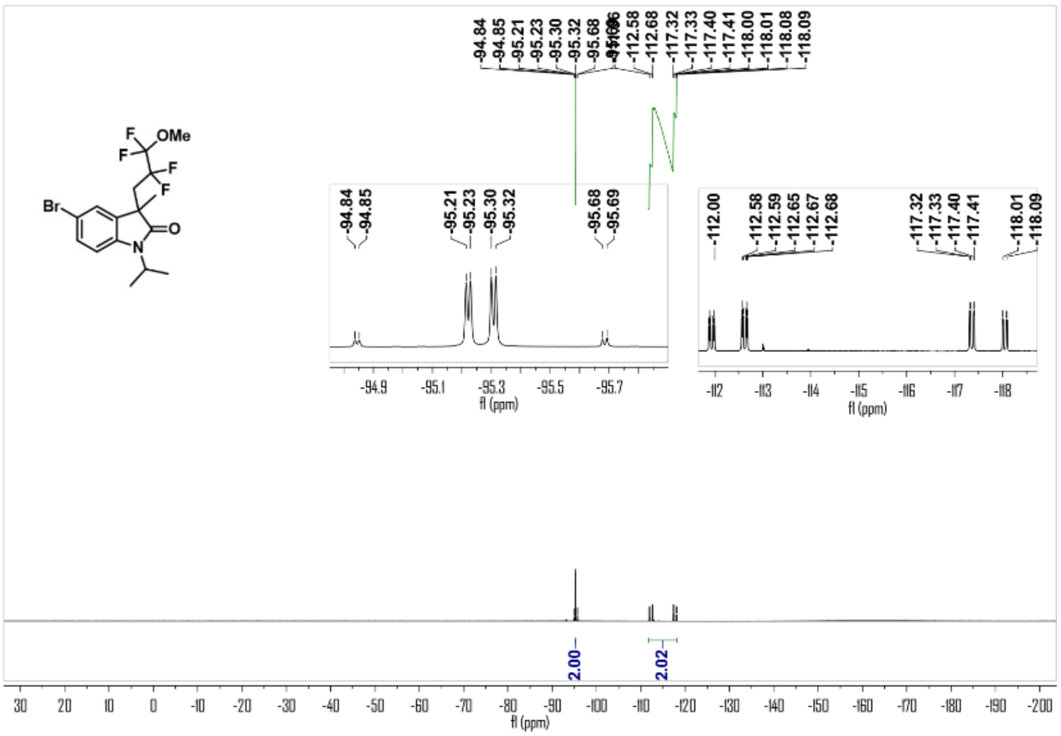

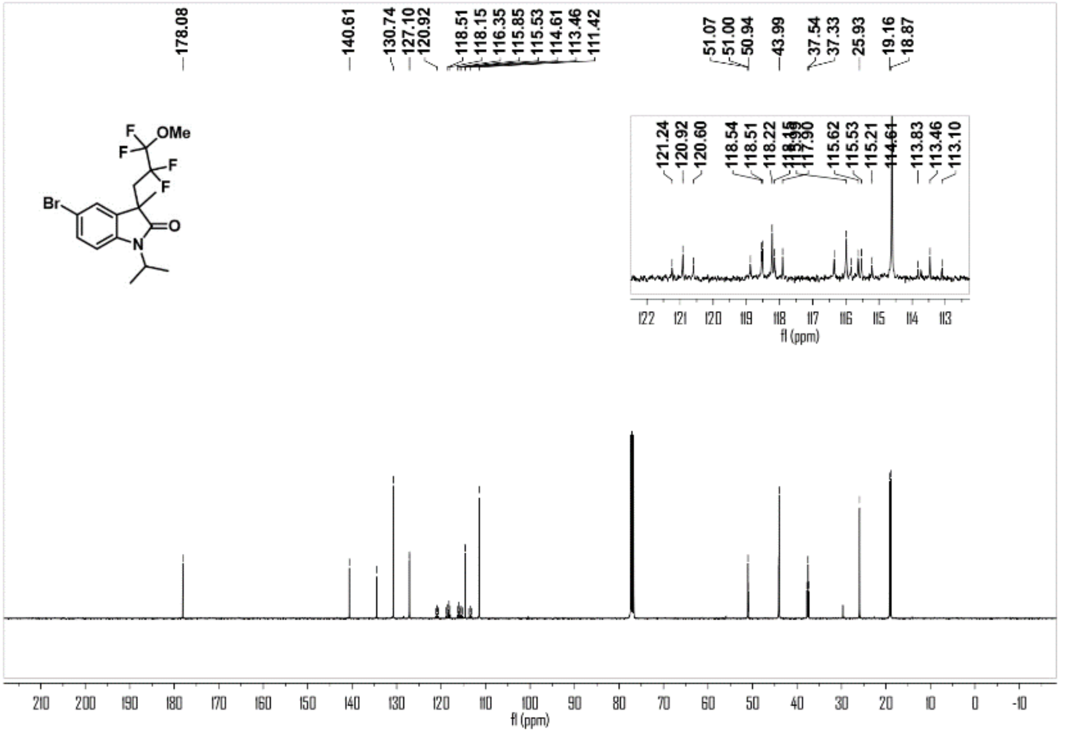

^{1}H, ^{19}F and ^{13}C NMR spectra of compound 5ak

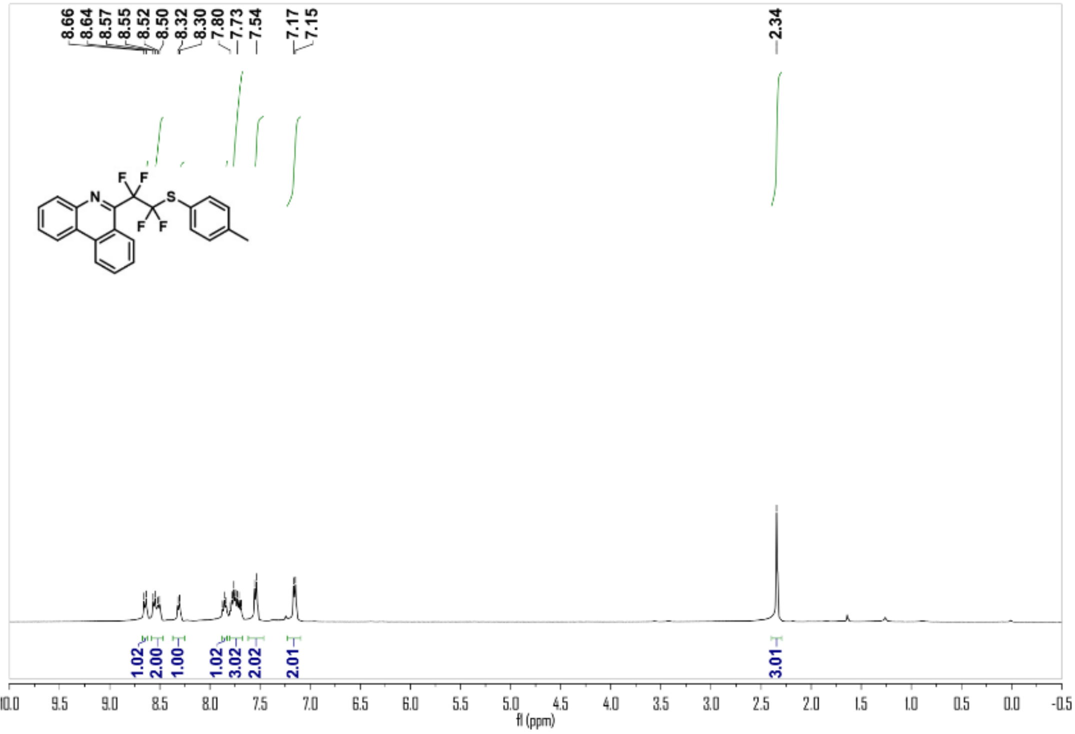

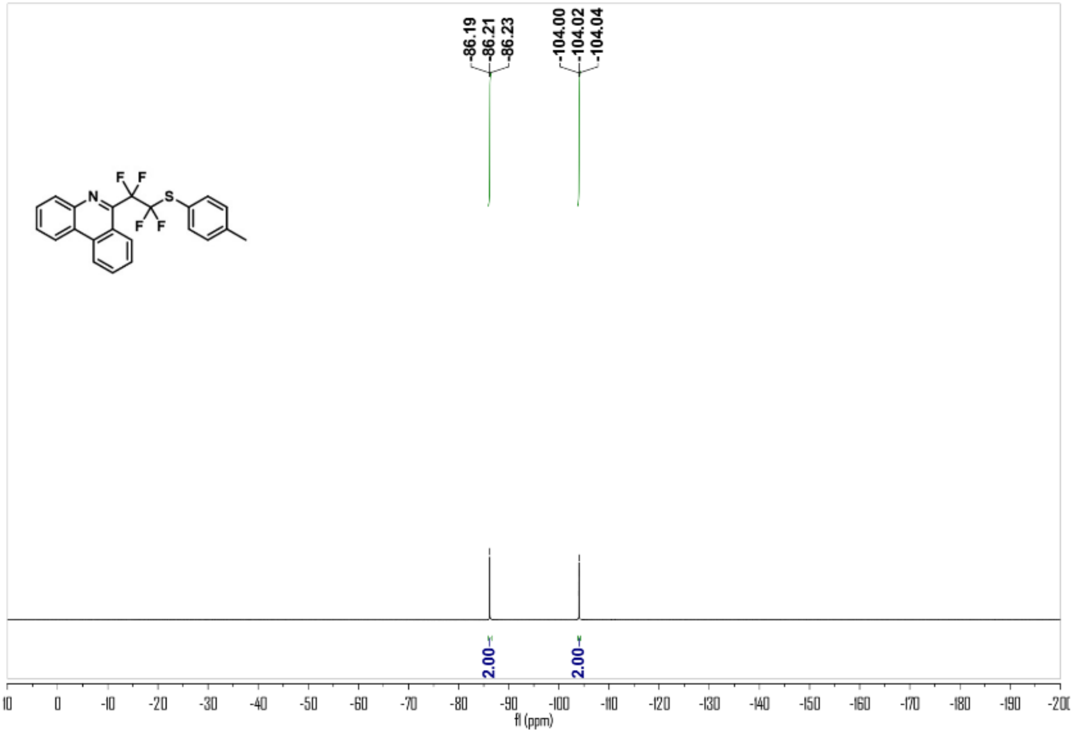
-86.19
-86.21
-86.23
-104.00
-104.02
-104.04
2.00
2.00
f1 (ppm)

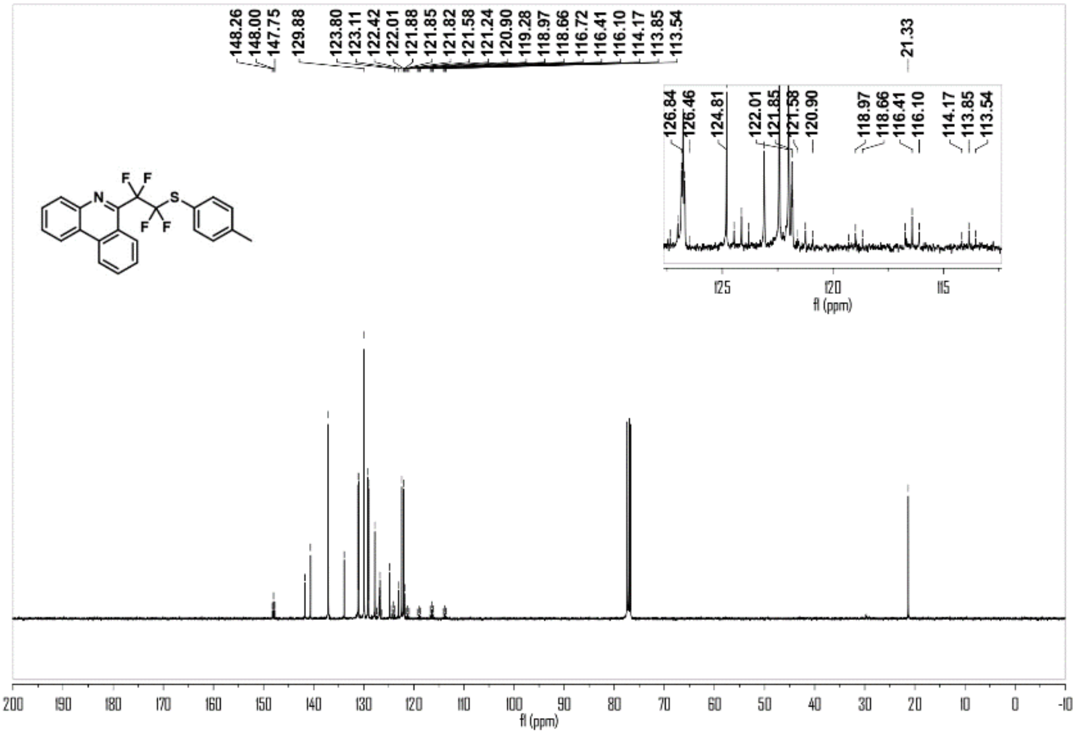
148.26
148.00
147.75
129.88
123.80
123.11
122.42
122.01
121.88
121.85
121.82
121.58
121.24
120.90
119.28
118.97
118.66
116.72
116.41
116.10
114.17
113.85
113.54
21.33
126.84
126.46
124.81
122.01
121.85
121.58
120.90
118.97
118.66
116.41
116.10
114.17
113.85
113.54
f1 (ppm)

^{1}H, ^{19}F and ^{13}C NMR spectra of compound 5al

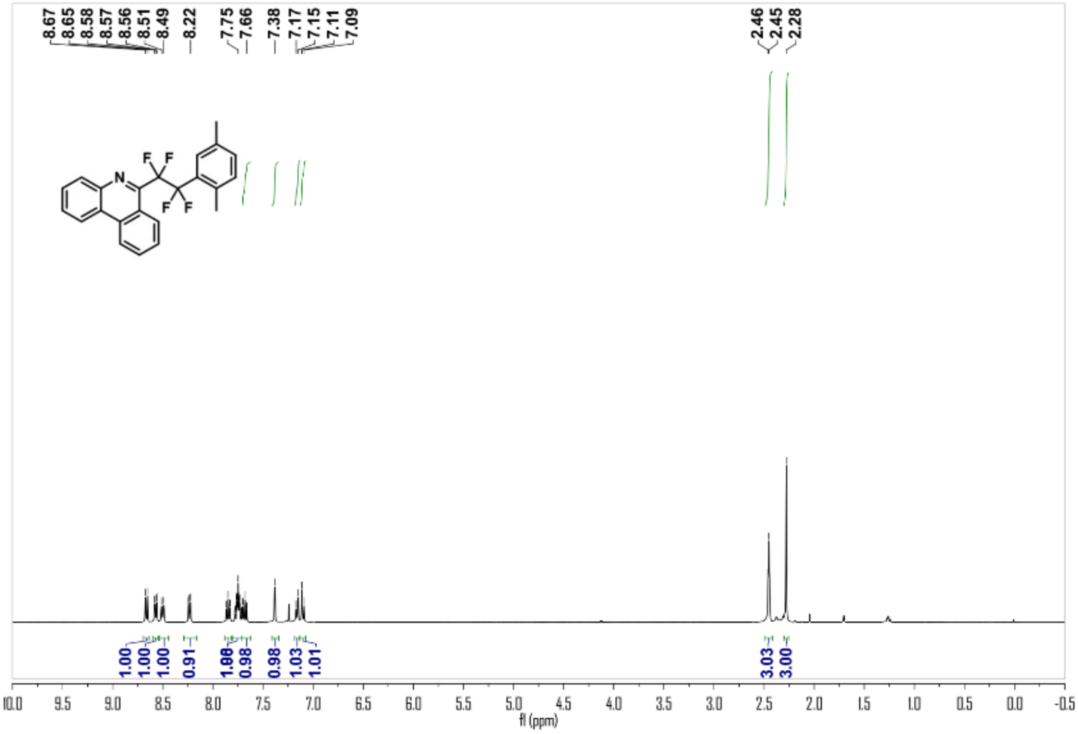

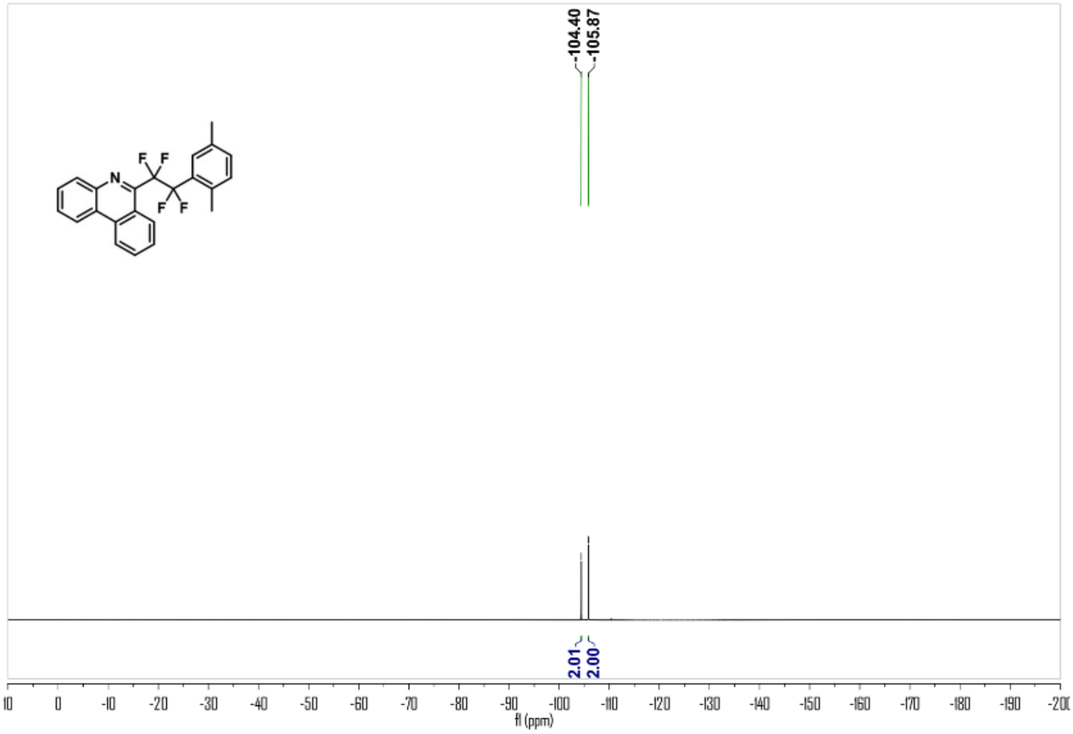

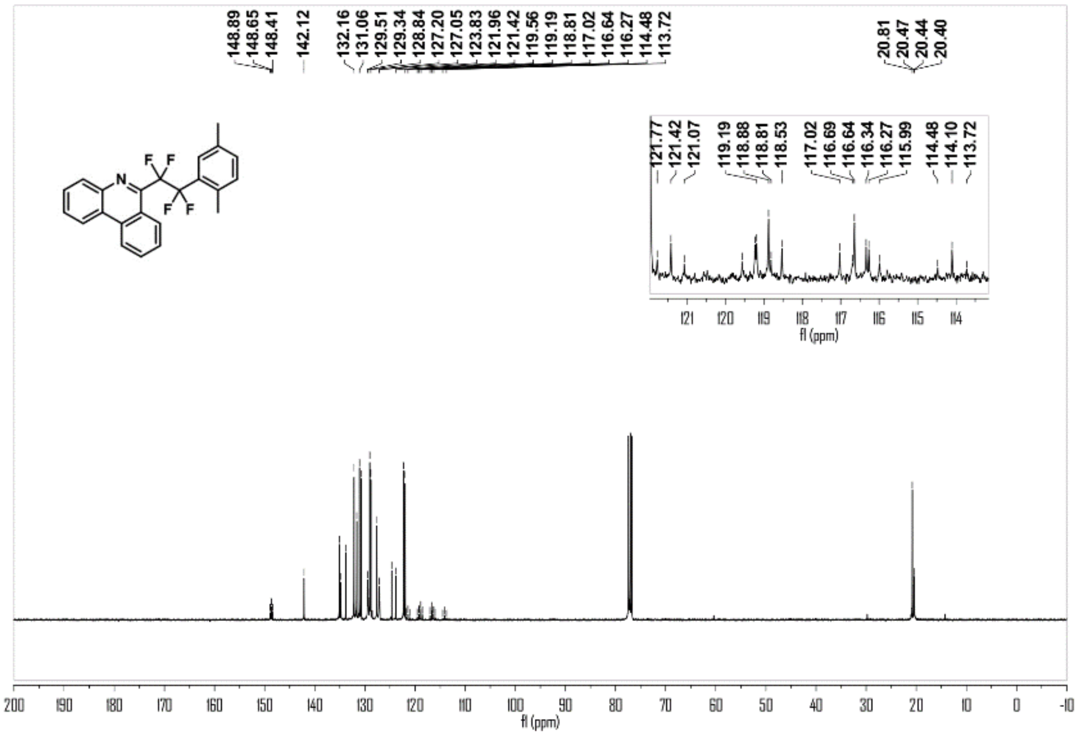

^{1}H, ^{19}F and ^{13}C NMR spectra of compound 5am

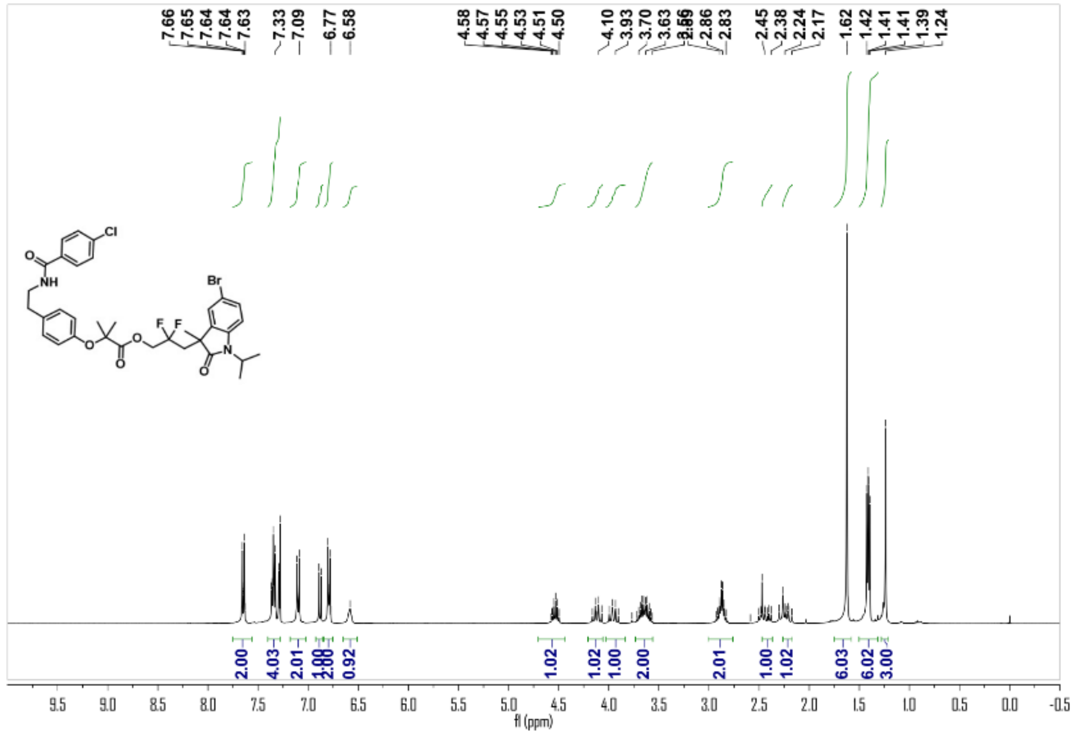

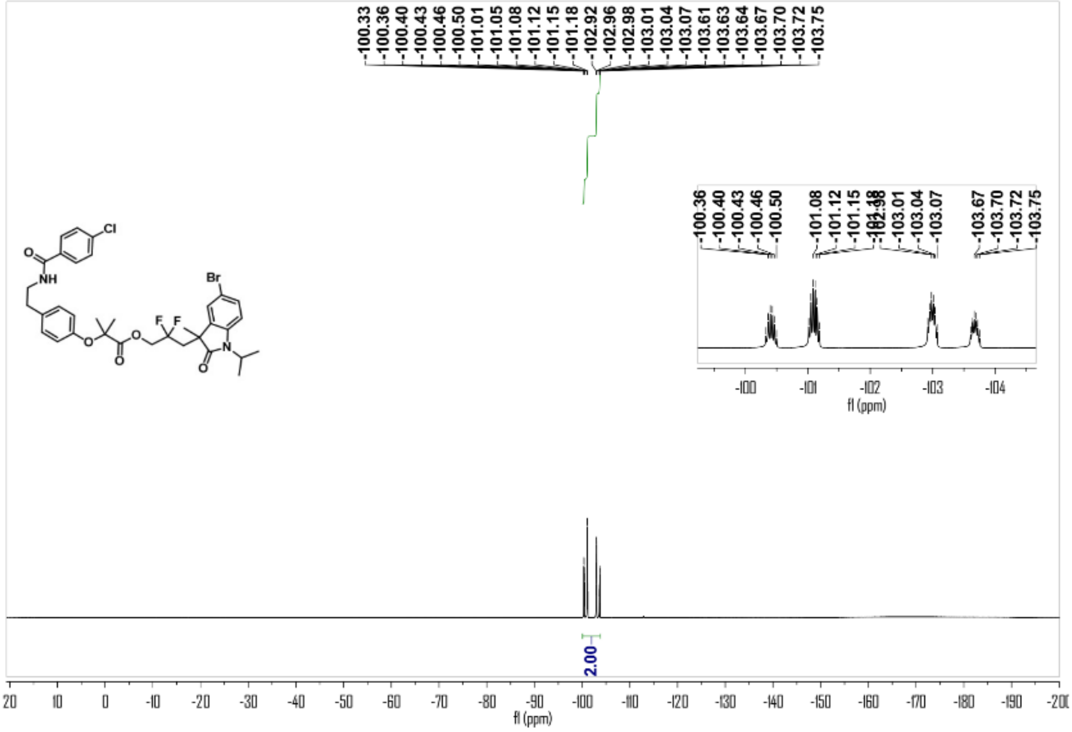

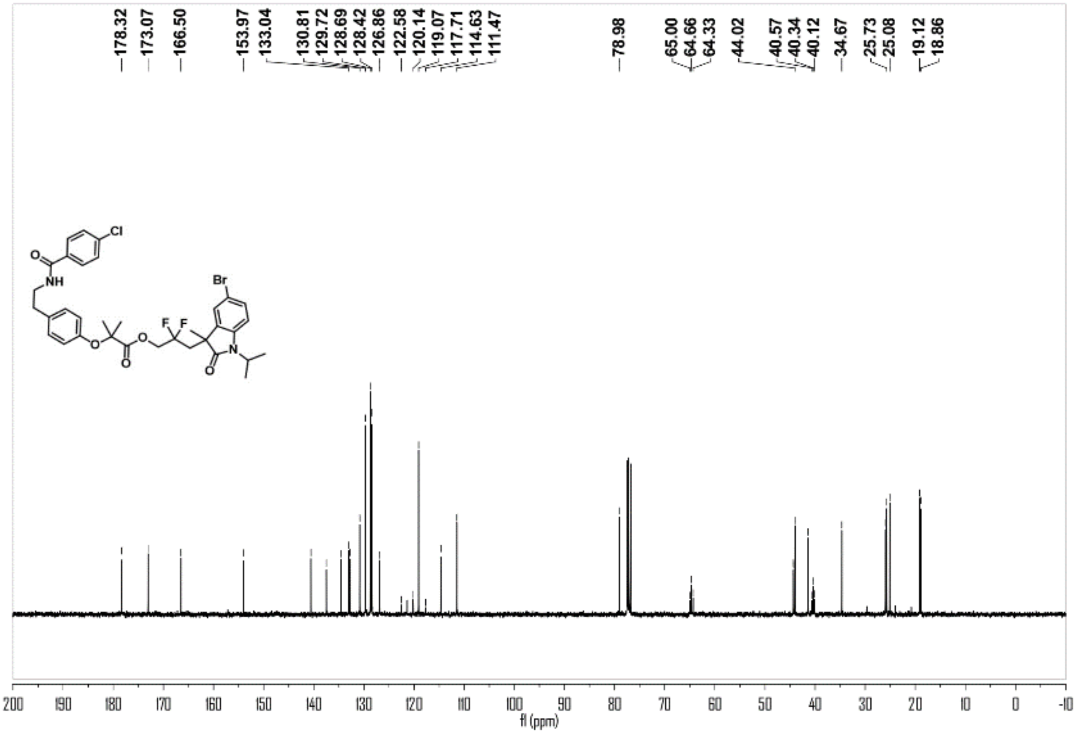

^{1}H, ^{19}F and ^{13}C NMR spectra of compound 5an

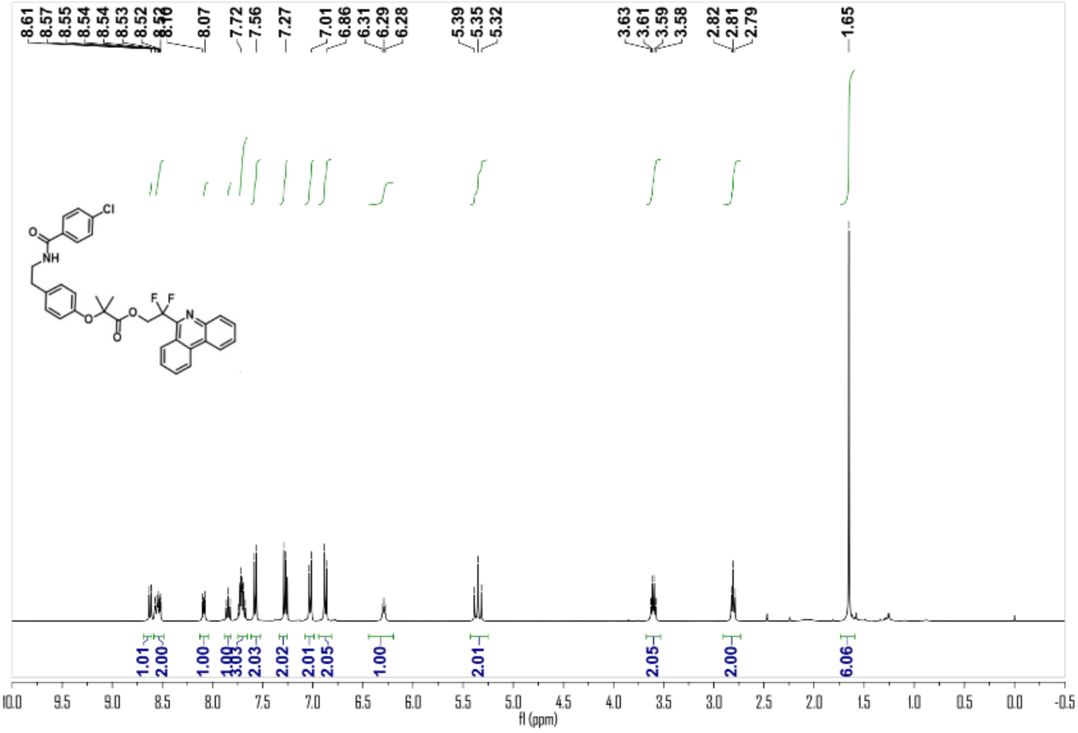

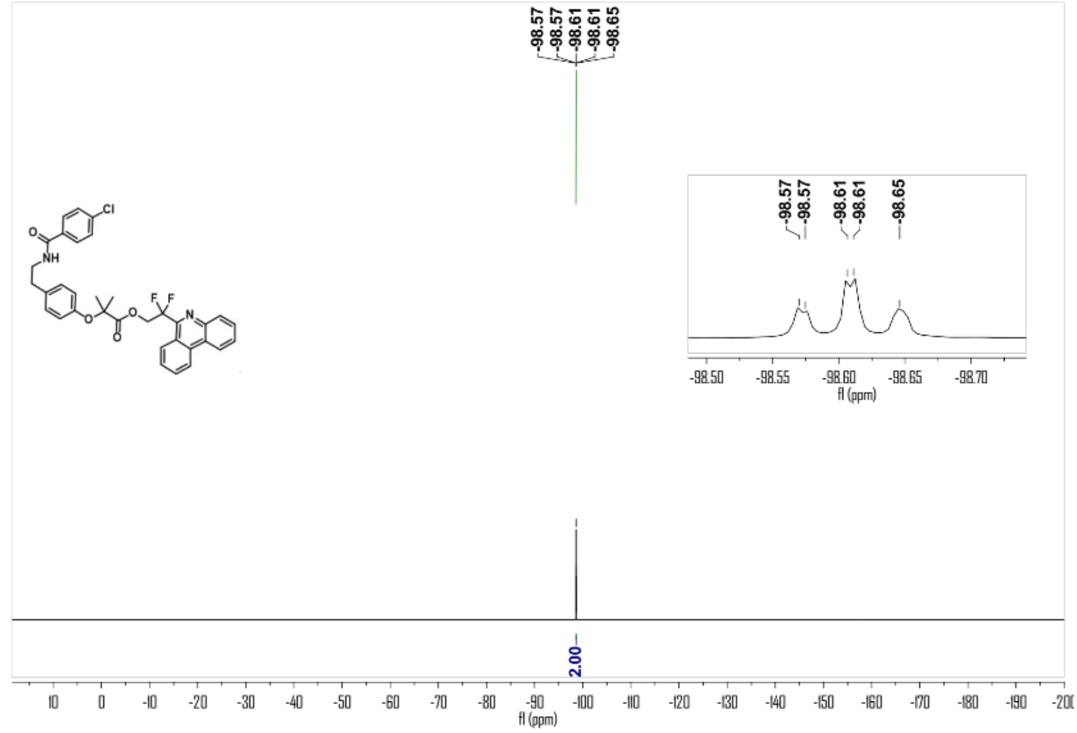

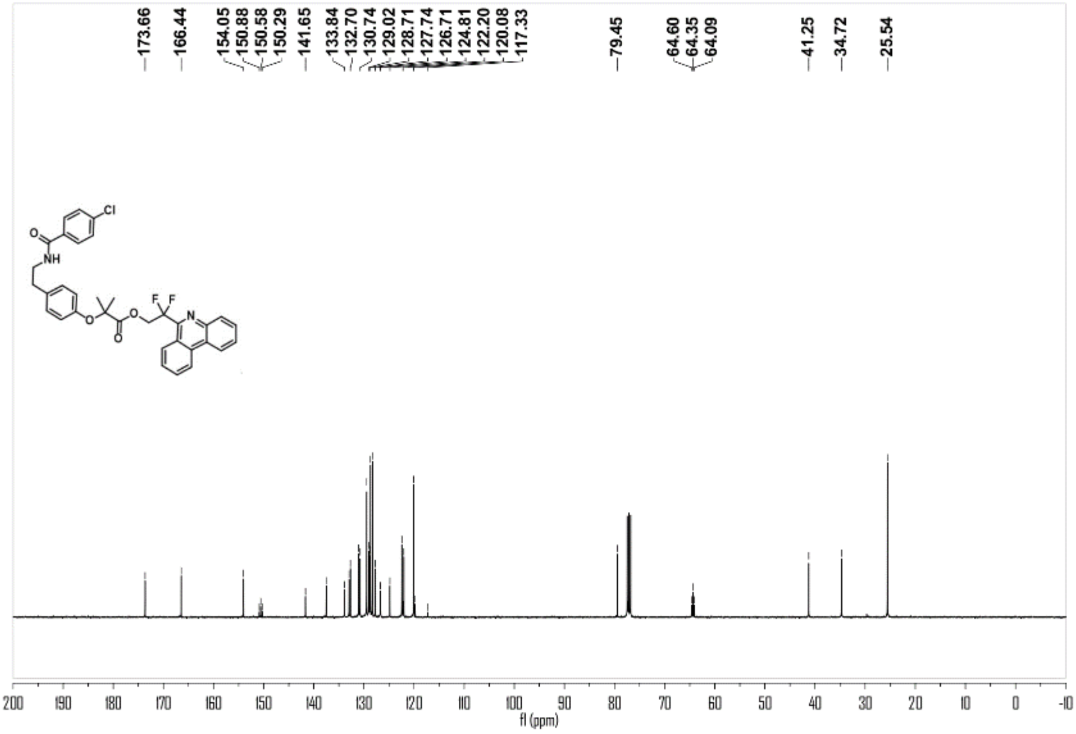

^{1}H, ^{19}F and ^{13}C NMR spectra of compound 5ao

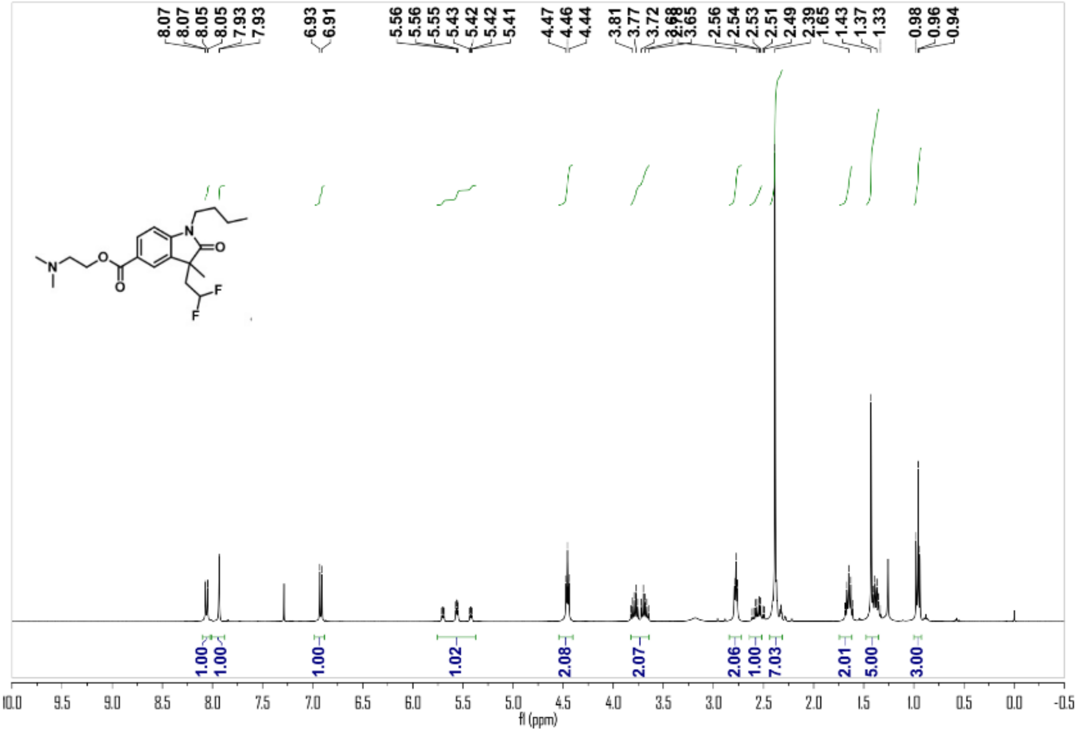

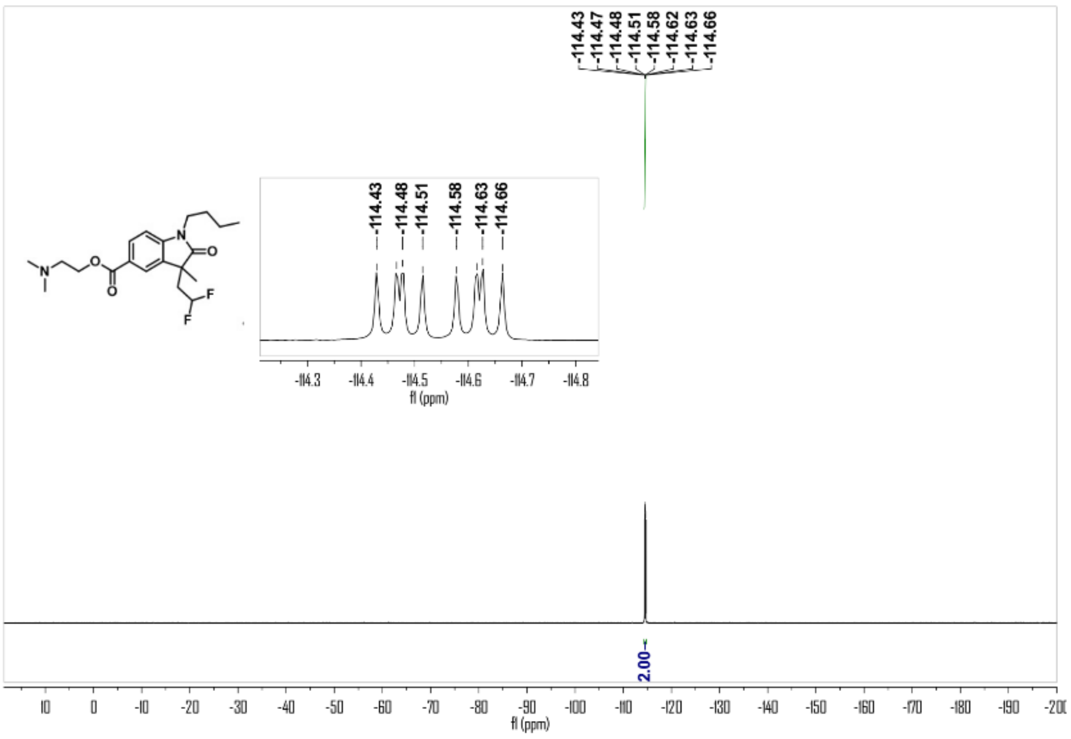
-114.43
-114.47
-114.48
-114.51
-114.58
-114.62
-114.63
-114.66
2.00
10 0 -10 -20 -30 -40 -50 -60 -70 -80 -90 -100 -110 -120 -130 -140 -150 -160 -170 -180 -190 -200
f1 (ppm)
-114.3 -114.4 -114.5 -114.6 -114.7 -114.8
f1 (ppm)

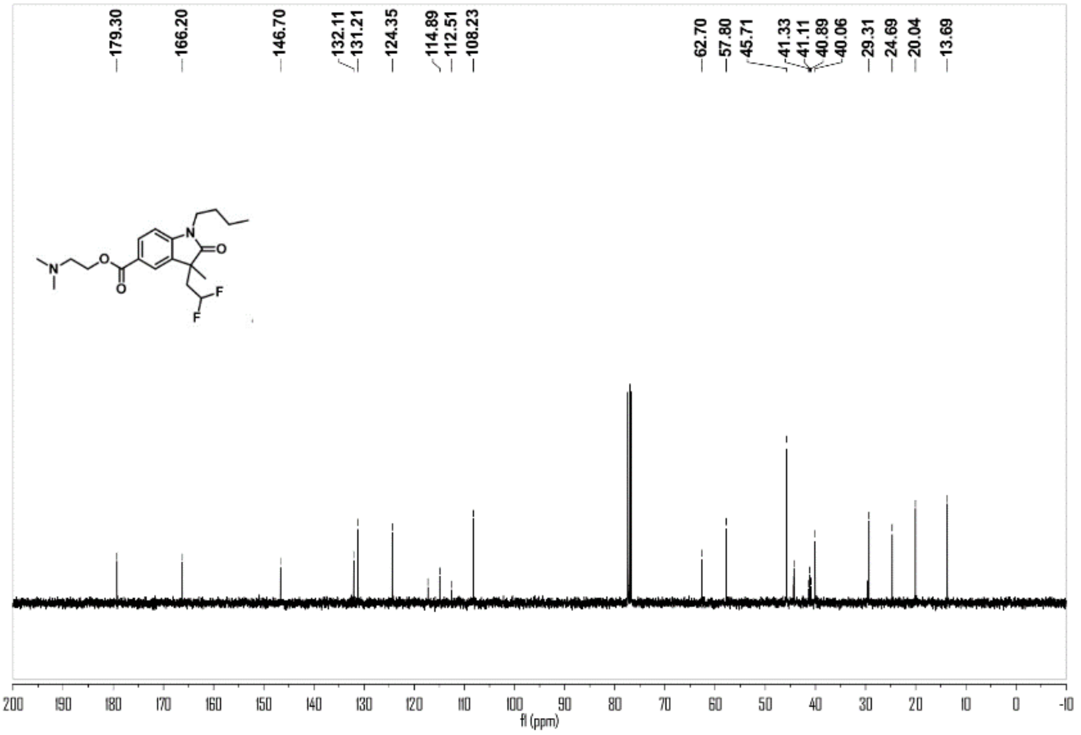
179.30
166.20
146.70
132.11
131.21
124.35
114.89
112.51
108.23
62.70
57.80
45.71
41.33
41.11
40.89
40.06
29.31
24.69
20.04
13.69
200 190 180 170 160 150 140 130 120 110 100 90 80 70 60 50 40 30 20 10 0 -10
f1 (ppm)

^{1}H, ^{19}F and ^{13}C NMR spectra of compound 7

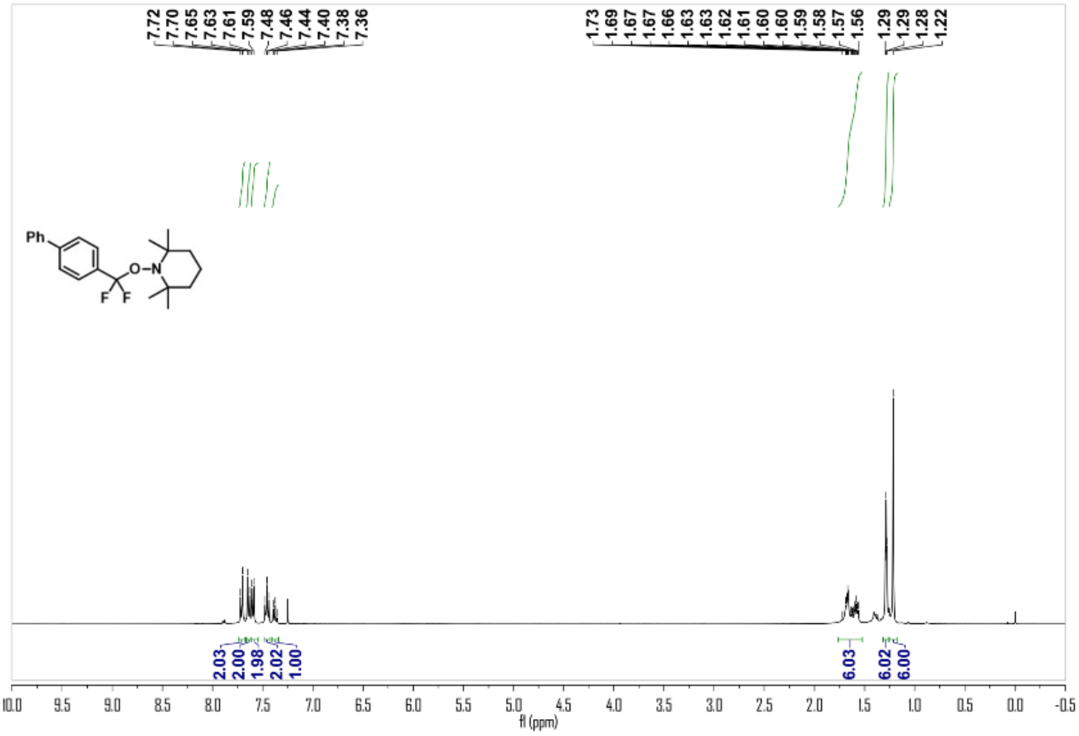

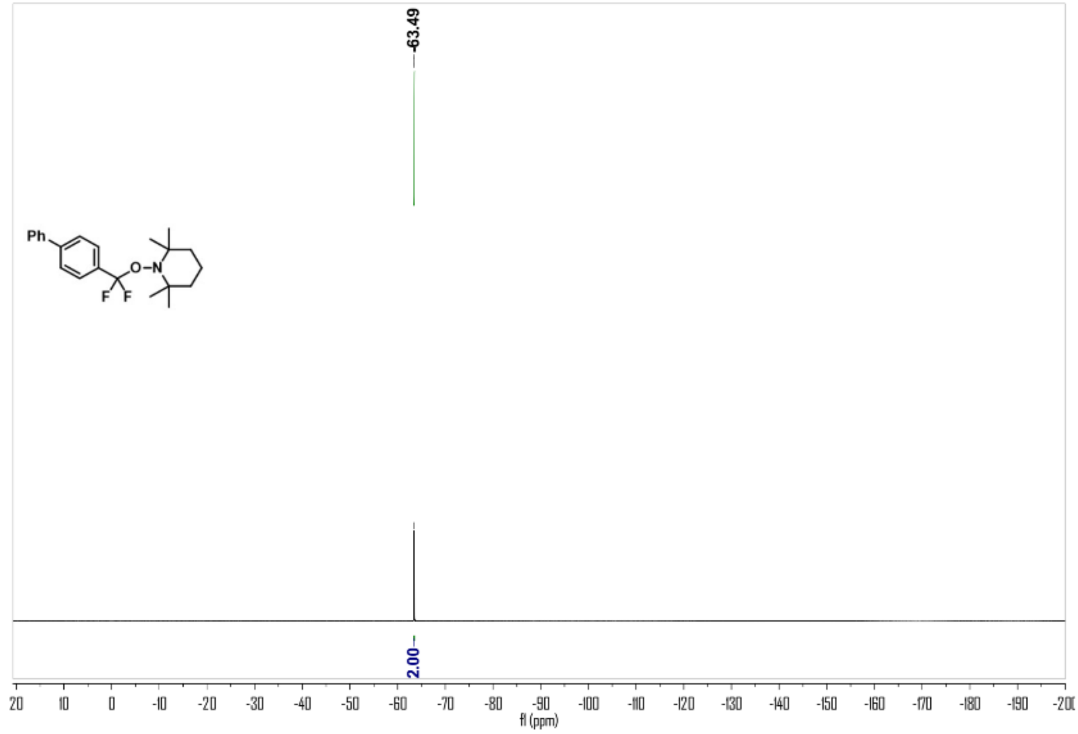

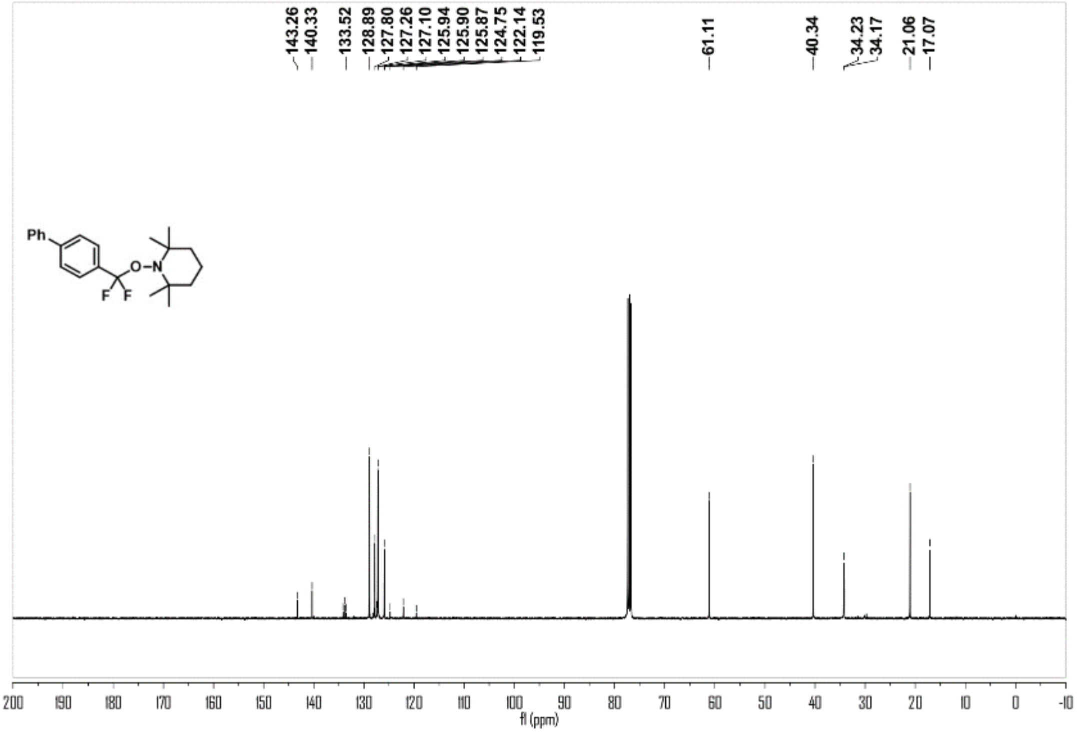

^{1}H, ^{19}F and ^{13}C NMR spectra of compound 9

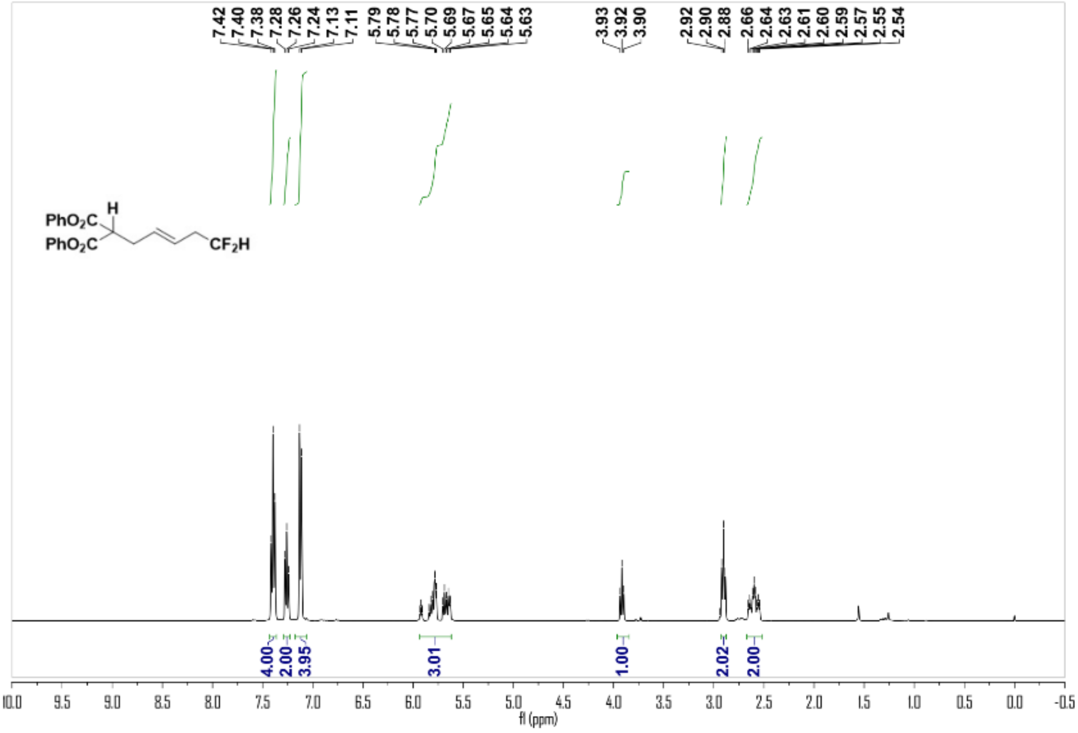

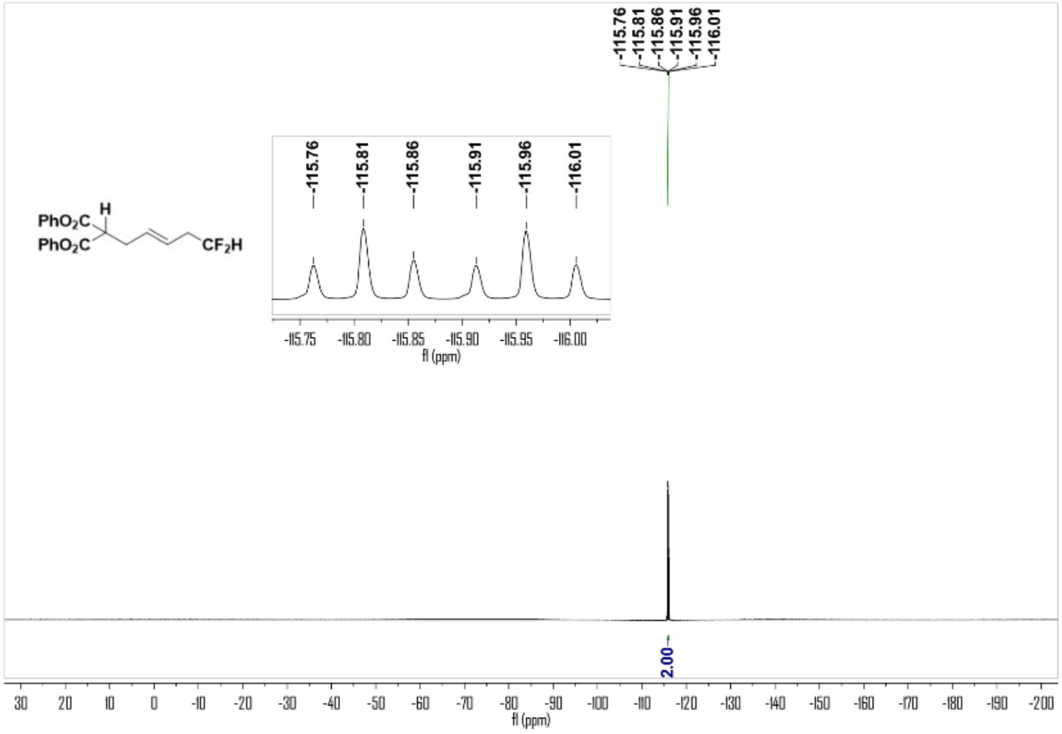
-115.76
-115.81
-115.86
-115.91
-115.96
-116.01
PhO2C
PhO2C
H
CF2H
2.00
f1 (ppm)

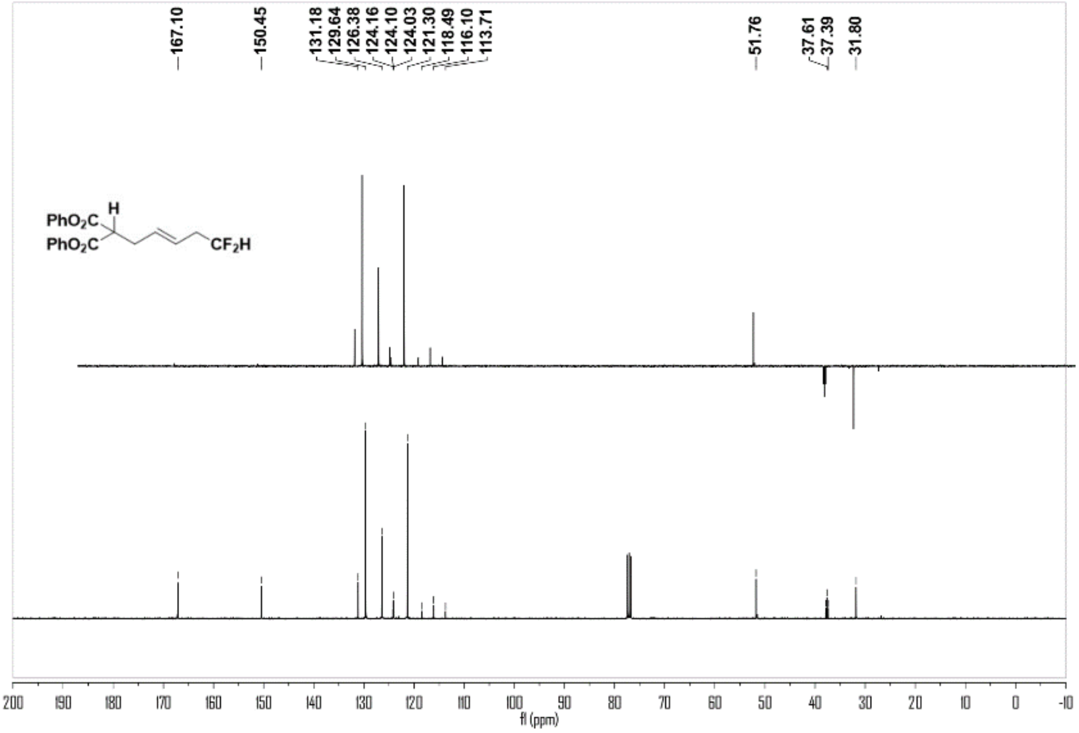
167.10
150.45
131.18
129.64
126.38
124.16
124.10
124.03
121.30
118.49
116.10
113.71
51.76
37.61
37.39
31.80
PhO2C
PhO2C
H
CF2H
f1 (ppm)

10. X-Ray crystallographic data

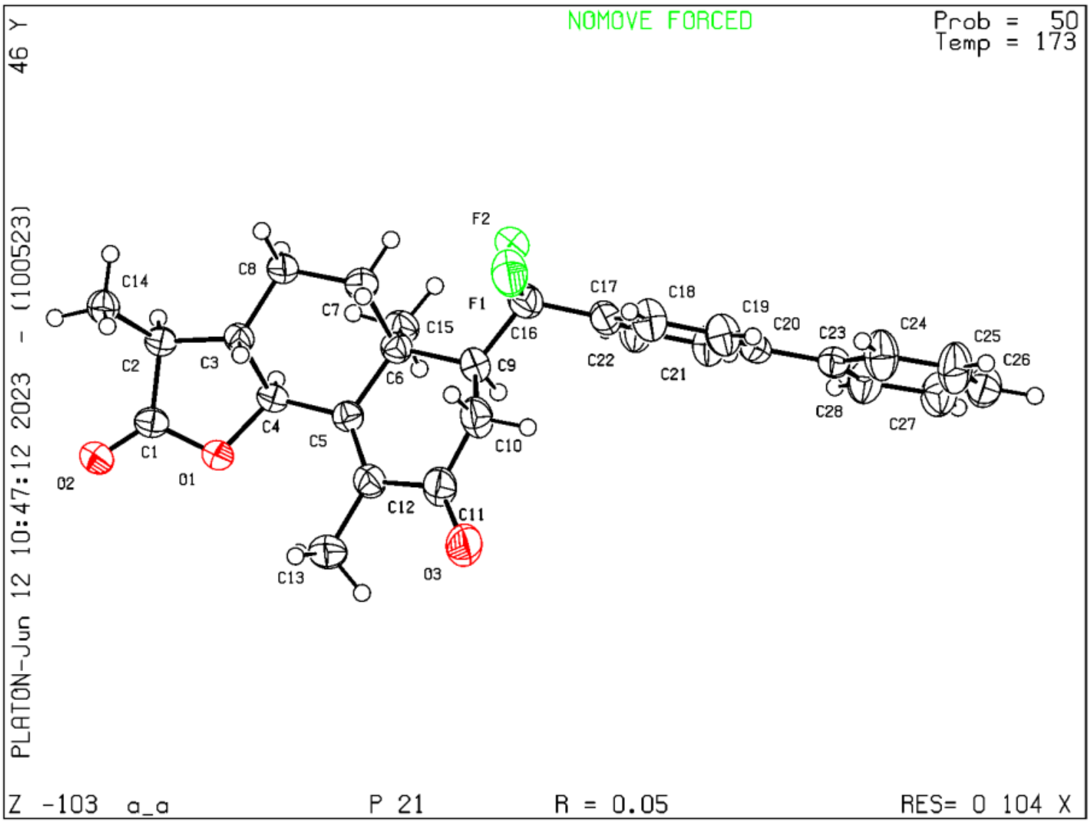

Figure S25. Ortep representation of **3bh-2**

Table S8. Crystal data and structure refinement details for **3bh-2**

**CCDC 2269262**	
Identification code	a_a
Empirical formula	C28 H28 F2 O3
Formula weight	450.50
Temperature	173(2) K
Wavelength	1.54178 A
Crystal system,<br>space group	Monoclinic<br>Pbca
Unit cell dimensions	a = 5.8814(3) A alpha = 90 deg.<br>b = 8.0087(5) A beta = 96.661(4) deg.<br>c = 24.5802(15) A gamma = 90 deg.
Volume/Å3	1149.97(12)
Z	2
Density (calculated)	1.301 Mg/m^3
Absorption coefficient	0.773 mm^-1
F(000)	476

Crystal size	0.120 x 0.100 x 0.100 mm
Theta range for data collection	5.436 to 61.164 deg.
Limiting indices	-6<=h<=6, -8<=k<=9, -27<=l<=27
Reflections collected / unique	12783 / 3452 [R(int) = 0.0503]
Completeness to theta = 61.164	99.9 %
Absorption correction	Semi-empirical from equivalents
Max. and min. transmission	0.7531 and 0.6896
Refinement method	Full-matrix least-squares on F^2
Data / restraints / parameters	3452 / 1 / 301
Goodness-of-fit on F^2	1.057
Final R indices [I>2sigma(I)]	R1 = 0.0464, wR2 = 0.1046
R indices (all data)	R1 = 0.0674, wR2 = 0.1154
Absolute structure parameter	0.05(13)
Extinction coefficient	n/a
Largest diff. peak and hole	0.352 and -0.215 e.A^-3

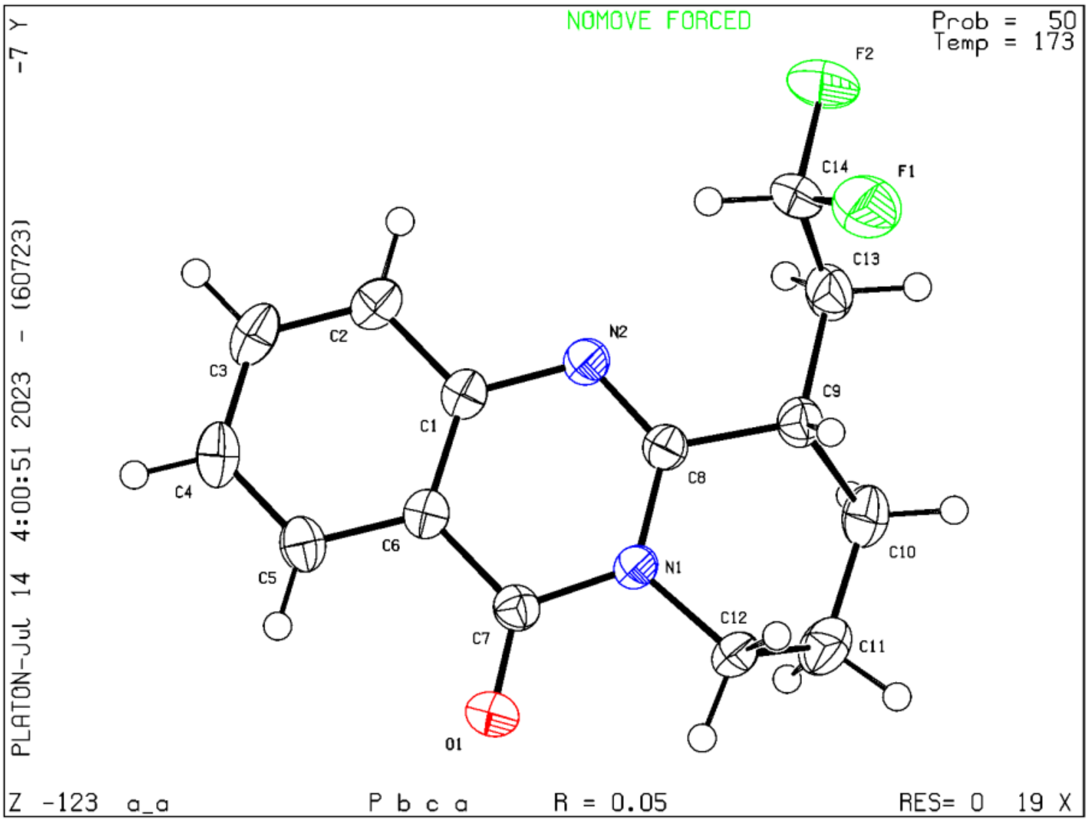

Figure S26. Ortep representation of **5t**

Table S9. Crystal data and structure refinement details for **5t**

CCDC 2281638	
Identification code	a_a
Empirical formula	C14 H14 F2 N2 O
Formula weight	264.27
Temperature	173(2) K
Wavelength	1.54178 Å
Crystal system	Orthorhombic
Space group	Pbca
Unit cell dimensions	a = 13.4213(5) Å alpha = 90 deg.
	b = 10.7867(4) Å beta = 90 deg.
	c = 16.9582(7) Å gamma = 90 deg.
Volume/Å3	2455.06(16)
Z	8
Density (calculated)	1.430 Mg/m^3
Absorption coefficient	0.949 mm^-1
F(000)	1104

Crystal size	0.180 x 0.160 x 0.140 mm
Theta range for data collection	5.216 to 68.719 deg
Limiting indices	-15<=h<=16, -12<=k<=12, -20<=l<=20
Reflections collected / unique	36242 / 2257 [R(int) = 0.0546]
Completeness to theta = 67.679	100.0 %
Absorption correction	Semi-empirical from equivalents
Max. and min. transmission	0.7531 and 0.6931
Refinement method	Full-matrix least-squares on F^2
Data / restraints / parameters	2257 / 0 / 172
Goodness-of-fit on F^2	1.067
Final R indices [I>2sigma(I)]	R1 = 0.0461, wR2 = 0.1264
R indices (all data)	R1 = 0.0491, wR2 = 0.1289
Extinction coefficient	n/a
Largest diff. peak and hole	0.277 and -0.216 e.A^-3

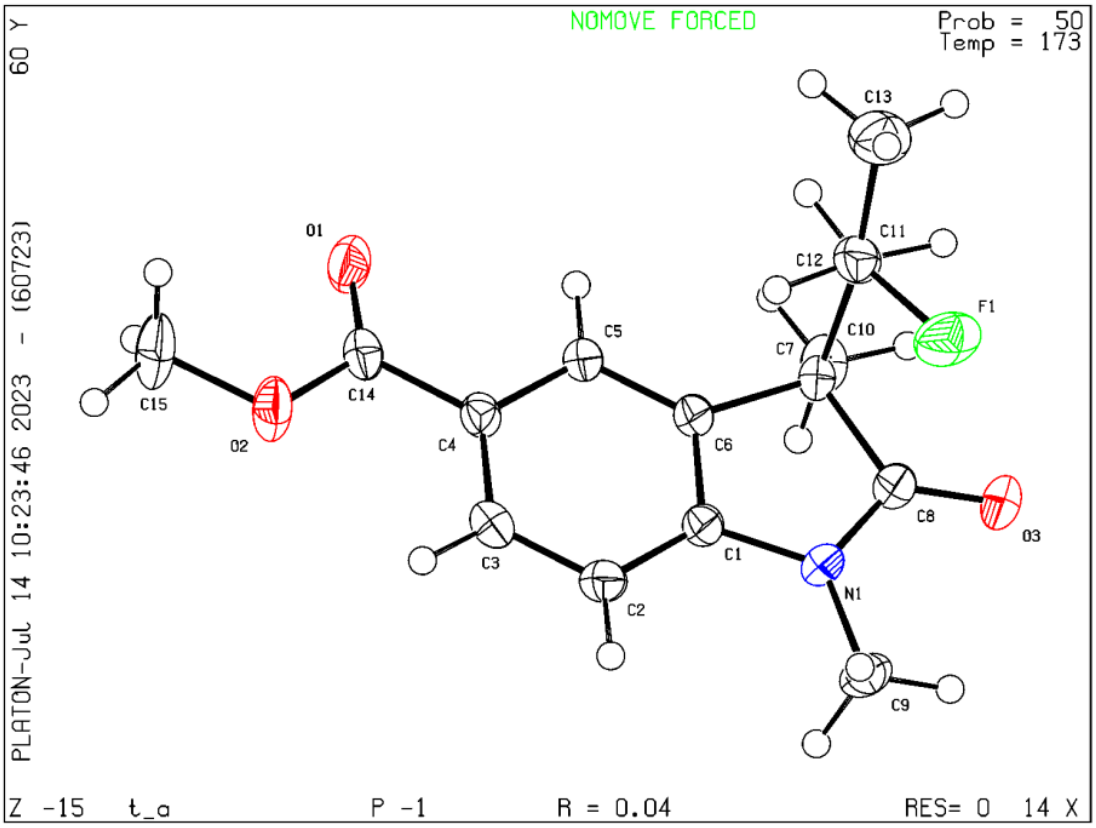

Figure S27. Ortep representation of **5ag-2**

Table S10. Crystal data and structure refinement details for **5ag-2**

CCDC 2281640	
Identification code	t_a
Empirical formula	C15 H18 F N O3
Formula weight	279.30
Temperature	173(2) K
Wavelength	1.54178 Å
Crystal system	Triclinic
Space group	Pbca
Unit cell dimensions	a = 7.9820(2) A alpha = 74.228(2) deg.
	b = 9.0625(2) A beta = 81.456(2) deg.
	c = 10.2331(3) A gamma = 89.004(2) deg.
Volume/Å3	704.25(3)
Z	2
Density (calculated)	1.317 Mg/m^3
Absorption coefficient	0.836 mm^-1
F(000)	296

Crystal size	0.150 x 0.140 x 0.120 mm
Theta range for data collection	4.541 to 68.354 deg
Limiting indices	-9<=h<=9, -10<=k<=10, -12<=l<=11
Reflections collected / unique	7622 / 2549 [R(int) = 0.0627]
Completeness to theta = 67.679	98.7 %
Absorption correction	Semi-empirical from equivalents
Max. and min. transmission	0.7531 and 0.6428
Refinement method	Full-matrix least-squares on F^2
Data / restraints / parameters	2549 / 0 / 185
Goodness-of-fit on F^2	1.059
Final R indices [I>2sigma(I)]	R1 = 0.0434, wR2 = 0.1096
R indices (all data)	R1 = 0.1134, wR2 = 0.1210
Extinction coefficient	n/a
Largest diff. peak and hole	0.243 and -0.290 e.A^-3
